# Modular Synthesis of Complex Benzoxaboraheterocycles through Chelation-Assisted Rh-Catalyzed [2 + 2 + 2] Cycloaddition

**DOI:** 10.1021/acscatal.3c05766

**Published:** 2024-01-24

**Authors:** John M. Halford-McGuff, Marek Varga, David B. Cordes, Aidan P. McKay, Allan J. B. Watson

**Affiliations:** EaStCHEM, School of Chemistry, University of St Andrews, North Haugh, St Andrews KY16 9ST, U.K.

**Keywords:** boron, cycloaddition, heterocycles, mechanism, sensing

## Abstract

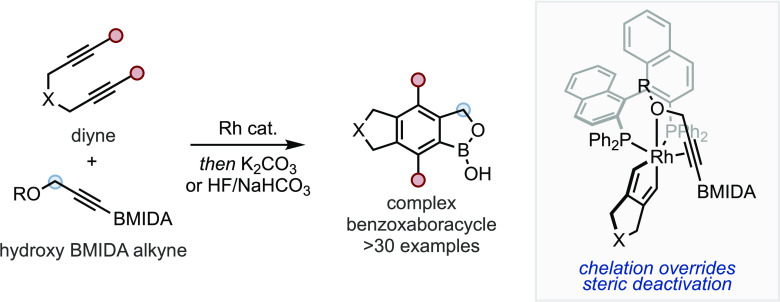

Benzoxaboraheterocycles (BOBs) are moieties of increasing interest in the pharmaceutical industry; however, the synthesis of these compounds is often difficult or impractical due to the sensitivity of the boron moiety, the requirement for metalation–borylation protocols, and lengthy syntheses. We report a straightforward, modular approach that enables access to complex examples of the BOB framework through a Rh-catalyzed [2 + 2 + 2] cycloaddition using MIDA-protected alkyne boronic acids. The key to the development of this methodology was overcoming the steric barrier to catalysis by leveraging chelation assistance. We show the utility of the method through synthesis of a broad range of BOB scaffolds, mechanistic information on the chelation effect, intramolecular alcohol-assisted BMIDA hydrolysis, and linear/cyclic BOB limits as well as comparative binding affinities of the product BOB frameworks for ribose-derived biomolecules.

Boron is a cornerstone element in synthetic chemistry. Classically, organoboron reagents have been used as nontoxic and bench-stable nucleophiles in numerous catalytic methodologies, in particular transition metal-based cross-coupling reactions (*e.g.*, Suzuki–Miyaura,^1,2^ Chan–Lam,^3,4^ and Hayashi^5,6^ reactions). Further applications are broad-ranging including within photocatalysis^7−13^ and materials chemistry;^14−16^ however, the rise of boron in pharmaceutical design is of particular significance.^17−20^

Heteroatoms are prolific in drug discovery with nitrogen, oxygen, and fluorine, especially prevalent.^21^ Borylated heterocycles are becoming key warheads for pharmaceutical development. The first boron-containing drug approved by the FDA was bortezomib (Figure 1a), a treatment for multiple myeloma and the first proteasome inhibitor approved for human use.^22−26^ This was followed by tavaborole (Figure 1a), which is a topical antifungal. Structurally, tavaborole is an example of a benzoxaboraheterocycle (BOB). This motif has important properties that offer unique advantages in drug design (Figure 1b):^27,28^ (1) The vacant *p*-orbital at boron allows for dynamic covalent binding to nucleophiles, for example, to serine residues in serine proteases. (2) They are isolobal to carboxylic acids while having a higher p*K*_a_, which can enhance protein binding.^29,30^ (3) Further heteroatoms can be incorporated into the boraheterocycle to influence overall properties and reactivity (*e.g.*, hydrolysis rate, p*K*_a_, catalytic activity).^31−33^ These attributes have led to new boron-containing drugs (*e.g.*, xeruborbactam, taniborbactam; Figure 1a); however, despite an increase in frequency in drug design, the synthesis of BOB scaffolds remains challenging.

**Figure 1:**
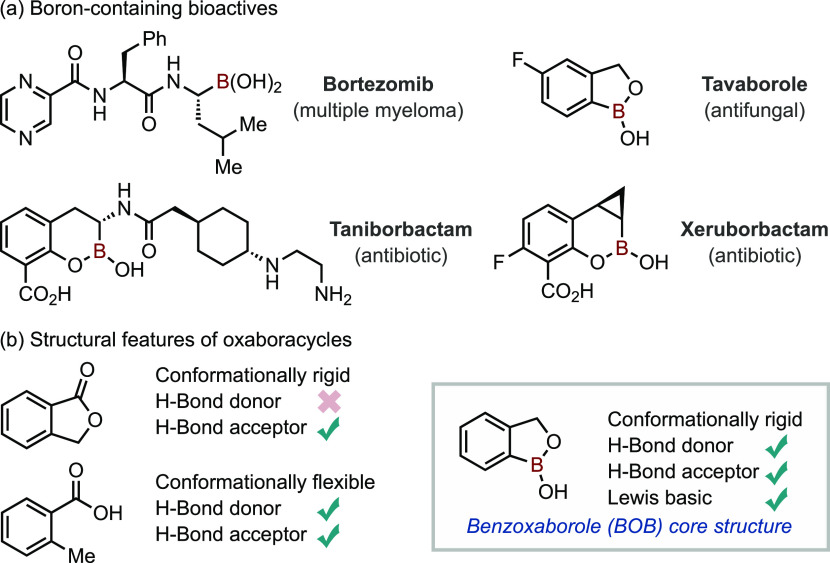
(a) Examples of boron-containing bioactives. (b) Selected structural features of benzoxaboroles.

Classical approaches to the BOB framework have been based on nucleophilic addition of stoichiometric organometallics to a borylated arene bearing an adjacent carbonyl^34^ or borylation of an *ortho*-halo benzyl alcohol derivative using Miyaura-type conditions (Scheme 1a).^35−37^ Contemporary approaches include B-insertion strategies using B–Br reagents (Scheme 1b), such as a dual Ni/Zn catalysis to insert a boron unit into the C(sp^3^)–O bond of benzodihydrofurans by Dong and co-workers^38^ and the electrophilic haloboration approach reported by Ingleson and co-workers to directly access benzoxaboronines from *o*-alkynyl phenols.^39^ A complementary approach that does not rely upon electrophilic borylating agents or C–B bond formation was developed by Sheppard and co-workers, where gold catalysis generated the benzoxaborinine from *o*-alkynyl boronic acids.^40^

**Scheme 1:**
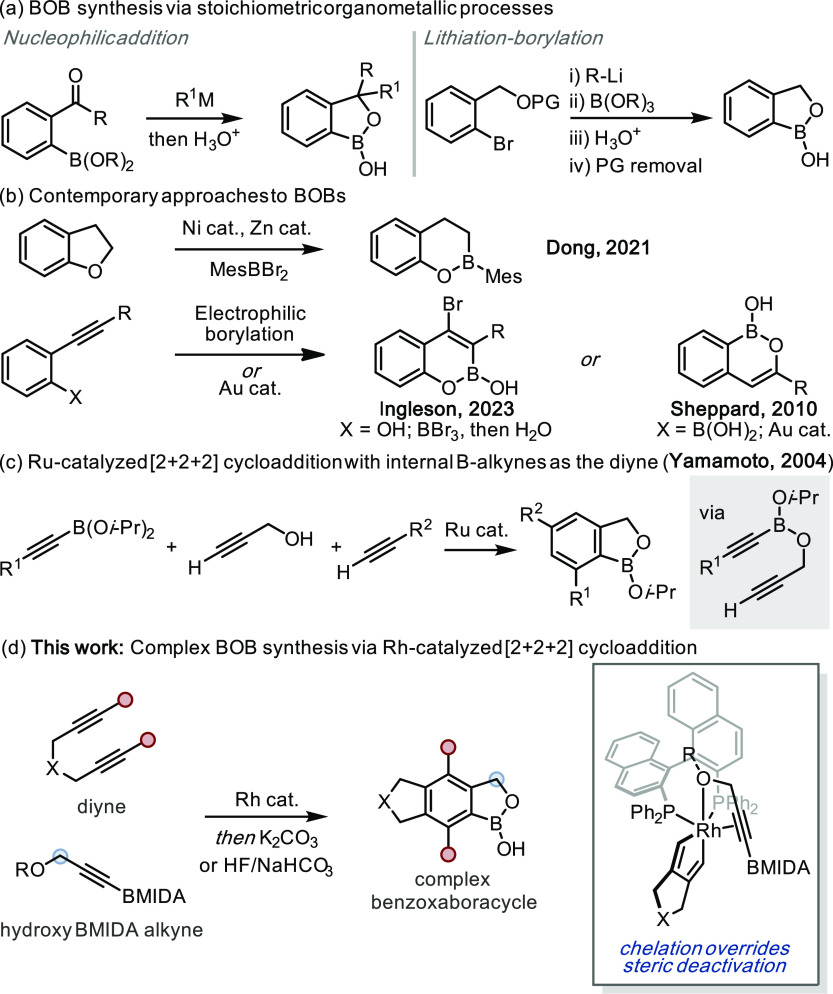
(a) Classical Approaches to BOBs *via* Stoichiometric Organometallics. (b) Selected Contemporary Approaches to BOBs. (c) Yamamoto’s BOB Synthesis *via* Templated Ru-Catalyzed [2 + 2 + 2] Cycloaddition. (d) This work: BOB Synthesis *via* Chelation-Assisted [2 + 2 + 2] Cycloaddition. MIDA, *N*-Methylimidodiacetate; PG, Protecting Group

An attractive synthetic approach to BOB compounds is through [2 + 2 + 2] cycloaddition. The main advantages over other approaches are its high atom efficiency and the rapid generation of molecular complexity using modular components.^41−50^ First disclosed in 1890 by Berthelot,^51^ this procedure has been improved considerably using transition metal catalysis, initially by Reppe,^52^ and it is now extensively used in a variety of fields from pharmaceutical and natural product synthesis to polymer chemistry.^41−50^

In the context of BOB synthesis, the [2 + 2 + 2] cycloaddition approach has seen limited development. Elegant work from Yamamoto and co-workers used ruthenium catalysis to generate benzoxaboroles through trimolecular [2 + 2 + 2] cycloaddition, wherein an alkyne boronic ester was used to template diyne formation by *in situ* transesterification using a propargylic alcohol (Scheme 1c).^53,54^

Here, we report the development of a method for the direct, modular, and regioselective synthesis of complex BOB scaffolds using Rh-catalyzed [2 + 2 + 2] cycloaddition, which uses chelation assistance to overcome an innate steric inhibition (Scheme 1d).

## Design Plan

**Scheme 2:**
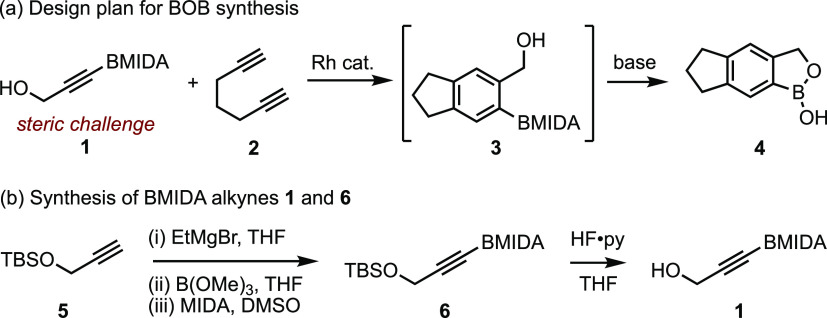
(a) Design Plan for the [2 + 2 + 2] Approach to BOB Scaffolds Using BMIDA Alkynes. (b) Synthesis of BMIDA Alkynes **1** and **6**

Due to facile transmetalation, unprotected alkynyl organoborons (*i.e.*, boronic acids or esters) are incompatible with Rh-catalyzed [2 + 2 + 2] cycloadditions.^55,56^ Consequently, a suitably protected organoboron would be required for this synthetic strategy. We envisioned a process based on the use of a BMIDA-functionalized propargyl alcohol (**1**, Scheme 2a). Cycloaddition with a diyne (*e.g.*, **2**) would generate a BMIDA-functionalized benzyl alcohol derivative (**3**) that, upon treatment with a mild base, would induce BMIDA deprotection,^57^ enabling the formation of the BOB ring system (**4**).

This immediately posed a challenge to the proposed catalysis: the Rh-catalyzed [2 + 2 + 2] cycloaddition is sterically controlled, with catalytic turnover directly related to the steric footprint of the alkyne substituents.^58^ With a combined *A*-value of >6,^58−61^ BMIDA-functionalized alkynes are ostensibly incompatible with this catalysis; however, coordinating functional groups are known to improve turnover.^58,62,63^ Accordingly, we considered that catalysis would be possible based on chelation assistance from the propargyl alcohol offsetting the steric deactivation from the BMIDA (Scheme 1d).

BMIDA alkyne **1** was accessed in two steps from TBS-protected propargyl alcohol **5***via* the borylation/MIDA route developed by Burke^64^ and subsequent desilylation by Kozlowski^65^ (Scheme 2b).

**Figure 2:**
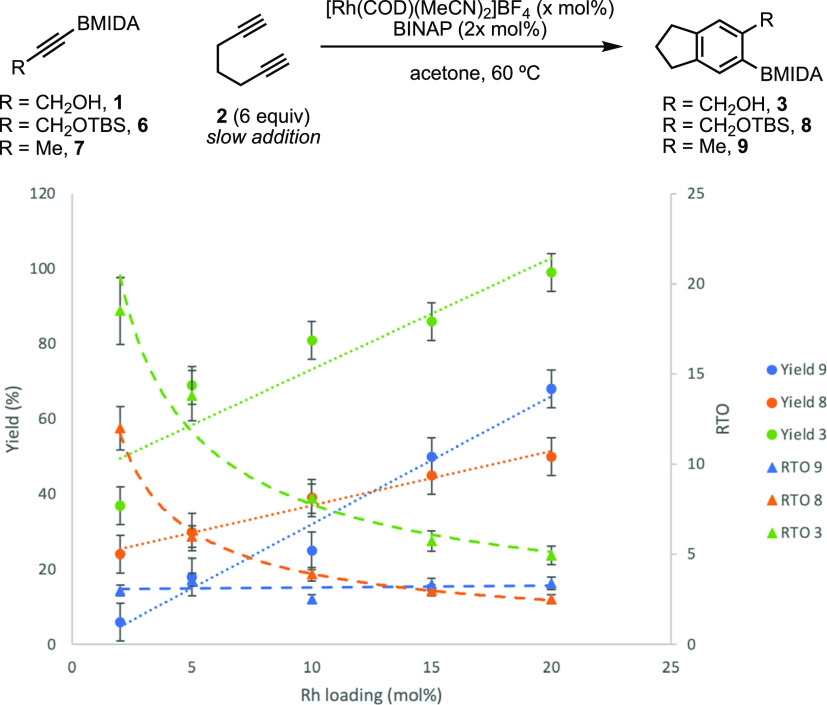
Rhodium turnover (RTO) and reaction yield *vs* catalyst loading for alkynes **1**, **6**, and **7**.

Initial assessment of **1** and benchmark diyne **2** in the Rh-catalyzed [2 + 2 + 2] cycloaddition revealed that catalysis was indeed possible, despite the steric issue of BMIDA, with turnover enhanced by the chelation assistance of the propargyl alcohol (Figure 2).

A comparison of **1***vs* propyne BMIDA (**7**) revealed static rhodium turnover (RTO) for **7** irrespective of the catalyst loading, consistent with the sterically controlled regime;^58^ however, despite the same steric parameters, **1** displayed enhanced turnover due to chelation assistance. Interestingly, the assessment of **6** revealed a similar but slightly diminished chelation assistance despite the presence of the TBS protecting group. This proved advantageous for method development: while **1** could be prepared and isolated, the stability of the neat material was poor and required use immediately. Alkyne **6** had no stability issues and therefore offered a complementary approach to the BOB framework using the same number of overall steps by incorporating TBS deprotection either as workup after cycloaddition or after purification of the aryl BMIDA (*vide infra*).

With chelation-assisted turnover established, complementary protocols were optimized for BOB synthesis *via* [2 + 2 + 2] cycloaddition using alkynes **1** and **6** (Table 1). Using alkyne **1** and combining with one-pot basic (K_2_CO_3_) workup to unmask the BMIDA,^57,66,67^**4** was obtained in 73% yield using 10 mol % [Rh] (entry 1). Increasing the catalyst loading had the expected effect of increasing the yield but decreasing the RTO and *vice versa* (entries 2–5), consistent with the [Rh] *vs* RTO analysis above (Figure 2).

**Table 1:**
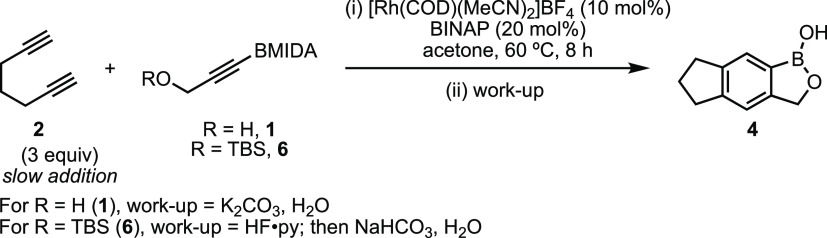
Selected Optimization Data

entry	alkyne	[Rh] (mol %)	BINAP (mol %)	4 (%)^a^	RTO^b^
1	1	10	20	73	7.3
2^c^	1	5	10	69	13.8
3^c^	1	10	20	81	8.1
4^c^	1	15	30	86	5.7
5^c^	1	20	40	>99	≥5.0
6^c^	6	5	10	18	3.6
7^c^	6	10	20	25	2.5
8^c^	6	15	30	50	3.3
9^c^	6	20	40	68	3.4
10^c^^,^^d^	1 or 6	20	40	<5	<0.2

a Determined by ^1^H NMR using an internal standard.

b Rhodium turnover.

c **2** (6 equiv), 16 h.

d Without slow addition. See the SI for full details. [Rh] = [Rh(COD)(MeCN)_2_]BF_4_.

Using TBS-protected alkyne **6** combined with one-pot desilylation (HF·py) and basic (NaHCO_3_) workup enabled formation of **4** using the more stable alkyne **6**. The same response to [Rh] variation was observed (entries 6–9), consistent with **1** and the preceding turnover analysis (Figure 2); however, based on the larger steric parameters of OTBS *vs* OH, **6** required 20 mol % [Rh] for an efficient reaction *vs* 10% for alcohol **1** (entry 1).

It should be noted that an excess of diyne and slow addition were required to offset the kinetics of the significantly more facile homodimer and trimerization of the diyne, consistent with previous studies on this fundamental rate difference (entry 10).^56,58^

**Scheme 3:**
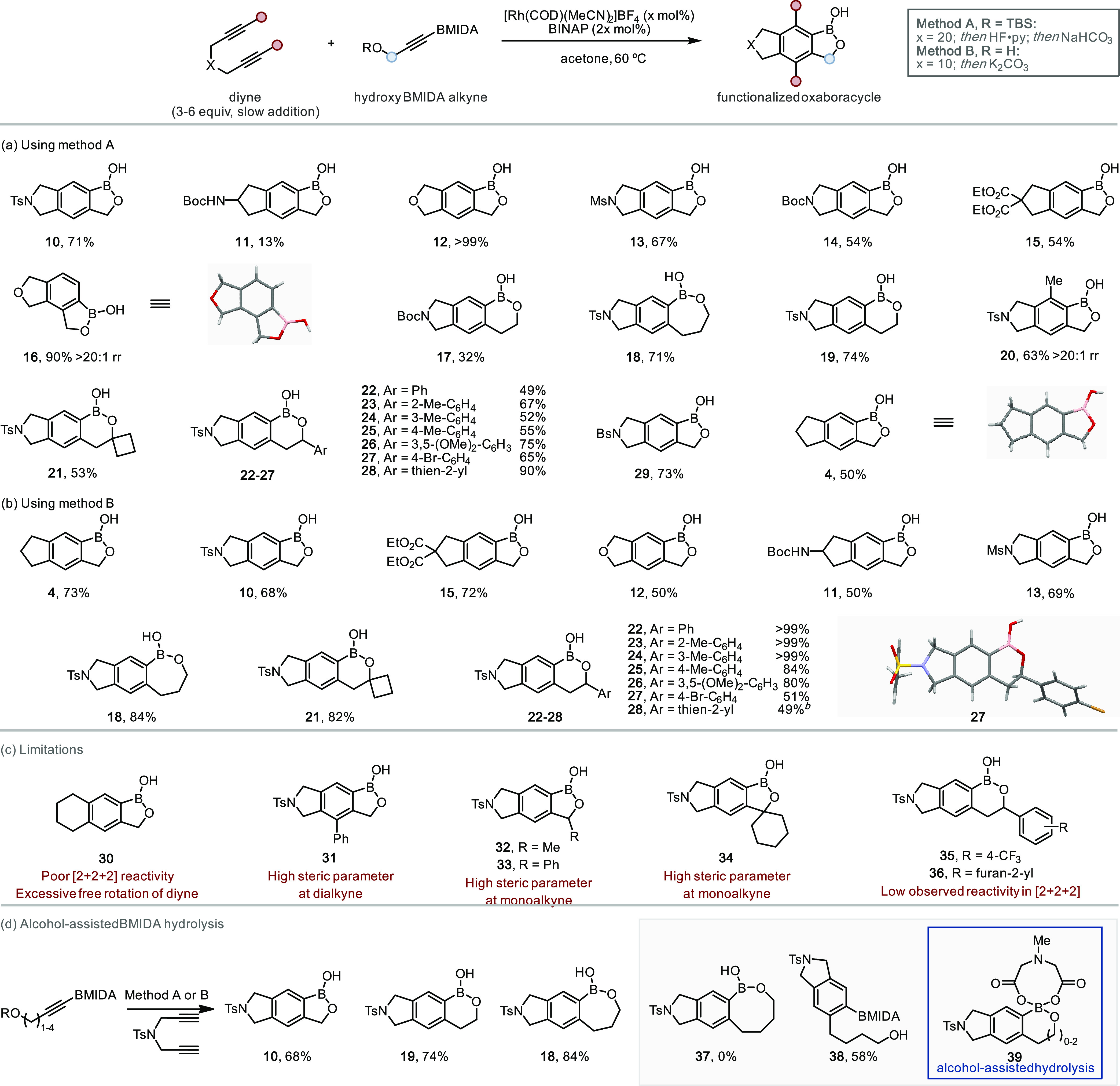
Selected Example Scope of BOB Frameworks Available through Rh-Catalyzed Chelation-Assisted [2 + 2 + 2] Cycloaddition (a) Method A, (b) method B, (c) limitations, and (d) alcohol-assisted BMIDA hydrolysis. See the SI for further details. The hydroxy BMIDA intermediate is unstable and used directly as the crude material, yield over two steps. Bs, brosyl.

The generality of the synthetic process was explored for both protocols, enabling access to a range of novel BOB scaffolds (TBS ether, method A, Scheme 3a; alcohol, method B, Scheme 3b). A variety of functional groups were tolerated, including sulfonamides, carbamates, esters, cyclobutyl groups, and bromides. Modification of the hydroxy BMIDA alkyne component allows for the generation of complex systems with oxaborole ring sizes of 5–7, a specific limitation for alternative methodologies.^39,40^ A surprising result was the high regioselectivity observed for **16** and **20**: the current doctrine in this area is that sterics govern the regioselectivity of [2 + 2 + 2] cycloadditions through kinetic effects; therefore, the isolated regioisomer would be expected to be the minor component; however, the opposite was observed.^68,69^ This origin of this increased regioselectivity likely arises from the enhanced control of alkyne insertion afforded from chelation of the pendant alcohol/ether motifs. Several limitations were encountered throughout the scope, which could be rationalized accordingly (Scheme 3c): first, due to the increased flexibility of 1,7 diynes (*e.g.*, **30**), the formation of the critical intermediate Rh(III) rhodacyclopentadiene is impeded and gave a low yield or no reactivity.^70−73^ Due to the sensitivity of the cycloaddition toward sterics, substitution on the diyne (**31**) and at the propargylic position (**32**–**34**) was not well tolerated.^58^ The remaining monoalkynes were poorly reactive overall in the [2 + 2 + 2] cycloaddition (**35**, **36**).

Regarding oxaborole ring size (Scheme 3d), 5- (**10**), 6- (**19**), and 7-membered (**18**) rings could be accessed in generally good yields; however, the formation of an 8-membered oxaborole (**37**) was not possible and instead, the aryl BMIDA **38** was isolated. Intriguingly, **38** was isolated after the deprotection protocol, implying that the BMIDA cleavage for **10**, **18**, and **19** was facilitated by the presence of the alcohol, for example, *via* dissociation of the *N*-methyl group on the BMIDA and association of the alcohol as shown in proposed intermediate **39**. In the cases of **37** and **38**, increased flexibility/rotation seems to have prevented this hydrolysis.

This suggested that ≥8-membered rings are a limitation for benzoxaboroles, consistent with work by Hall and co-workers, where the formation of an 8-membered BOB was also found to be disfavored.^74^

**Scheme 4:**
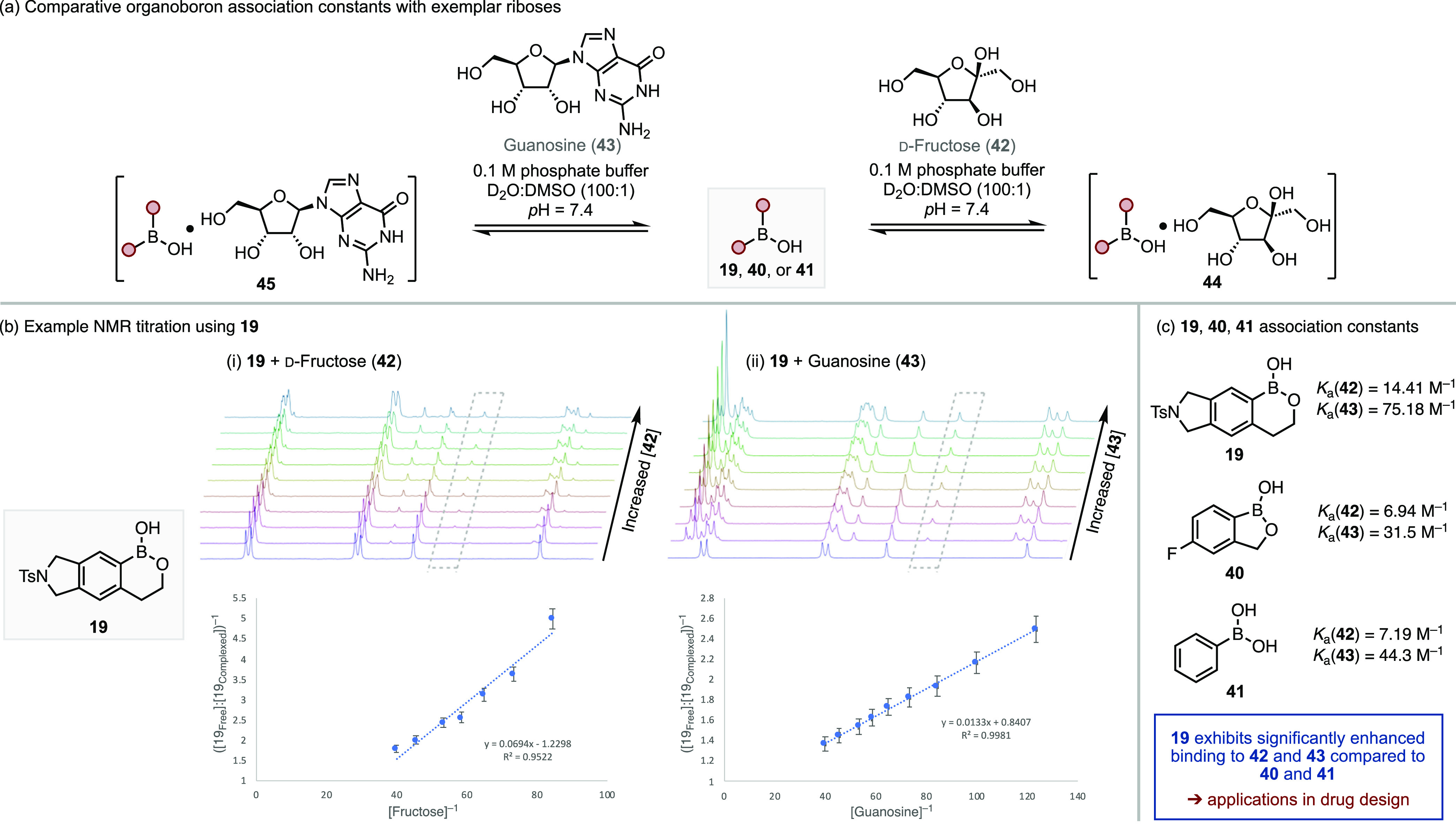
Association Constants (*K*_a_) of Organoborons with a Ribose and Nucleoside (a) Schematic representation of association complex formation. (b) Example ^1^H NMR titration and Benesi–Hildebrand plots using **19** with **42** and **43**. (c) Association constants for complex formation of **19**, **40**, and **41** with **42** and **43**.

The utility of organoboron compounds, including BOBs, within pharmaceutical development is linked with their ability to act as dynamic covalent inhibitors, especially for targets with alcohol-based residues in the active site (*e.g.*, serine proteases).^17−20^ The method developed above allows access to rare BOB frameworks, which have significant potential for exploration of the underdeveloped dynamic covalent inhibitor chemical space. Accordingly, with access to these compounds enabled, we sought to establish how effectively these may bind to exemplar biomolecules by comparison of binding affinity to representative ribose-based biomolecules *vs* known organoboron compounds (Scheme 4). Using the procedure developed by Hall and co-workers,^75,76^ the association constants of representative BOB **19** were compared to those of tavaborole (**40**) and PhB(OH)_2_ (**41**) with d-fructose (**42**) and guanosine (**43**) (Scheme 4b). We observed that the binding of **19** to **42** displayed a *K*_a_ value almost double that of **40** and **41**. More strikingly, **19** showed significantly enhanced binding to **43**, compared to that of either **40** or **41**. Moreover, BOB **19** displayed a high coefficient, >2-fold greater than that of tavaborole **40**, where binding to ribose is the known mode of action.^77−79^ These data emphasize the potential use of these BOB scaffolds as sensors of sugars and nucleosides.

In summary, a method for the synthesis of rare benzoxaboroles has been developed *via* Rh-catalyzed chelation-assisted [2 + 2 + 2] cycloaddition. Leveraging the chelating effect of a local alcohol to offset the steric impact on catalytic turnover, synthetically practical Rh catalyst loading may be used to generate complex BOBs in good to excellent yields. The reaction exhibits good functional group tolerance, and limitations have been disclosed. The dataset has also suggested an intramolecular alcohol-assisted BMIDA hydrolysis. Finally, comparative binding affinities for the new BOB frameworks to ribose-derived biomolecules have suggested utility as warheads for the development of dynamic covalent inhibitors with greater affinity than that of other organoboron derivatives.

## Supporting Information Available

Characterization data; copies of ^1^H and ^13^C NMR spectra; crystal structure data (PDF)

## Abbreviations

Ar: aryl
BOB: benzoxaboraheterocycle
Boc: *tert*-butoxycarbonyl
Bs: brosyl
DMSO: dimethylsulfoxide
MIDA: *N*-methylimidodiacetate
Ms: mesyl
NMR: nuclear magnetic resonance
PG: protecting group
TBS: *tert*-butyldimethylsilyl
Ts: tosyl
