# Supplementary material for: Modular Synthesis of Complex Benzoxaboraheterocycles through Chelation-Assisted Rh-Catalyzed [2 + 2 + 2] Cycloaddition
Source: ACS Catal. 2024 Jan 24;14(3):1846–54. doi: 10.1021/acscatal.3c05766 (PMC10845118; doi:10.1021/acscatal.3c05766)
Supplement: Supplementary file 1 — cs3c05766_si_001.pdf [file cs3c05766_si_001.pdf]

Modular synthesis of complex benzoxaboraheterocycles through chelation-assisted Rh-catalyzed [2+2+2]cycloaddition

John M. Halford-McGuff,[a] Marek Varga,[a] David B. Cordes,[a] Aidan P. McKay,[a] and Allan J. B. Watson*[a]

[a]EaSTCHEM, School of Chemistry, University of St Andrews, North Haugh, St Andrews, Fife, KY16 9ST, U.K.

*Email: aw260@st-andrews.ac.uk

Supporting Information

Contents

1. General S2
2. General Procedures and Synthesis of Starting Materials S3
3. Product Characterization Data S36
4. NMR Association Constant Determination S50
5. Optimization Data S54
6. Chelation Data S55
7. X-ray Diffraction Data S57
8. Spectra S58
9. References S166

1. General

Reagents and solvents were obtained from commercial suppliers and were not purified further unless specified. Purification (where specified) was performed following the standard procedures.[1] Dry solvents (THF, CH_2Cl_2) were provided by a PureSolv SPS-400-5 solvent purification system.

Reactions were carried out in standard borosilicate glassware or 2 mL microwave vials with septum caps. Glassware was either flame-dried under vacuum or allowed to dry in a 180 ºC oven for 24 h before use and then sparged with nitrogen. Room temperature was approximately 18 °C. Reactions at high temperature were heated using a DrySyn metal heating baths or a silicone oil bath. Reactions at 0 °C were performed using and ice/water bath, −5 °C reaction temperatures were achieved with ice/brine mixture, and −78 °C was using dry ice/acetone baths. Degassing was performed by the freeze-pump-thaw technique over three cycles. Additions over times greater than 1 h were performed using either a kdScientific 200 or a World Precision Instruments Aladdin-220 syringe pump.

TLC was carried out using Merck aluminum-backed silica plates coated with F_{254} fluorescent indicator, analyzed under UV light, and developed using aqueous $KMnO_4$ or ethanolic vanillin solutions, where appropriate. Flash column chromatography performed using silica gel (40-62 µm, Fluorochem).

^{1}H, ^{13}C (DEPTQ), and ^{19}F NMR (with or without 1H decoupling) spectra were recorded by either a Bruker AVII 400 (BBFO probe) or AVIII-HD 500 (and AVIII 500 with BBFO+ and Prodigy BBFO probes, respectively) at 400-101-376 MHz or at 500-126-377 MHz, respectively. ^{1}H NMR Spectra recorded at 700 MHz and ^{13}C at 176 MHZ were recorded on a Bruker AVIII-HD 700 with Prodigy TCI probe. ^{11}B NMR spectra were recorded on a Bruker AV300 spectrometer at 96 MHz or a Bruker AV400 at 126 MHz. All spectra were recorded at room temperature with the deuterated solvents used as a lock for spectra and internal reference (*d*-chloroform: ^{1}H, 7.26 ppm; ^{13}C, 77.16 ppm; d_6-acetone: ^{1}H, 2.05 ppm, ^{13}C, 29.8 ppm; d_6-dimethylsulfoxide: ^{1}H 2.50 ppm, ^{13}C 39.5 ppm; d_3-acetonitrile: ^{1}H 1.94 ppm, ^{13}C 1.3 ppm). For ^{11}B NMR, samples were externally referenced to F_3B•OEt_2 in $CDCl_3$. NMR spectra are reported as follows: chemical shift/ppm (multiplicity, coupling constant(s), number of nuclei, assignment). Multiplicity given as br (broad), s (singlet), d (doublet), t (triplet), q (quartet), quint (quintet), h (hextet), m (multiplet), and combinations thereof. Throughout, ^{13}C signals adjacent to boron were not observed. Signals which overlap with one another are described as multiplets. Note that some compounds exhibit extremely poor solubility commonly available NMR solvents (d_4-MeOH, d_3-MeCN, *d*-$CDCl_3$, d_6-acetone) and thus spectra are very weak meaning that HMBC analysis was often required to identify specific signals. Furthermore, some compounds exhibit instability *via* protodeboronation, anhydride formation, or ring opening, the most prevalent issue was the reaction with reactive oxidation species to generate phenols, a well-known trait of similar molecules.[2–4] In general, the OH peak of the oxaboracycle was not observed by ^{1}H NMR; however, it has been assigned where possible. Note that diastereotopicity was observed in some ^{1}H and ^{13}C NMR spectra. IR spectra were recorded using a Shimadzu IT Affinity-1 Fourier transform IR spectrophotometer with a Specac Quest ATR (diamond puck). The spectra were recorded as specified in the procedure as films (using CH_2Cl_2), as solids, or as neat liquids. Transmittance was recorded with maximal absorption wavenumbers given as cm^{-1}. Mass spectra were recorded on a Bruker micrOTOF benchtop ESI with either positive or negative electrospray ionization or EI using a Thermo Mat 900XP, Double Focusing Hi-resolution mass spectrometer. The number of decimal places was determined by the accuracy of the machine (*i.e.*, both 4 and 5 digits given).

2. General Procedures and Synthesis of Starting Materials

General procedure A: Synthesis of alkyne BMIDA through metalation-borylation followed by transligation using MIDA anhydride.

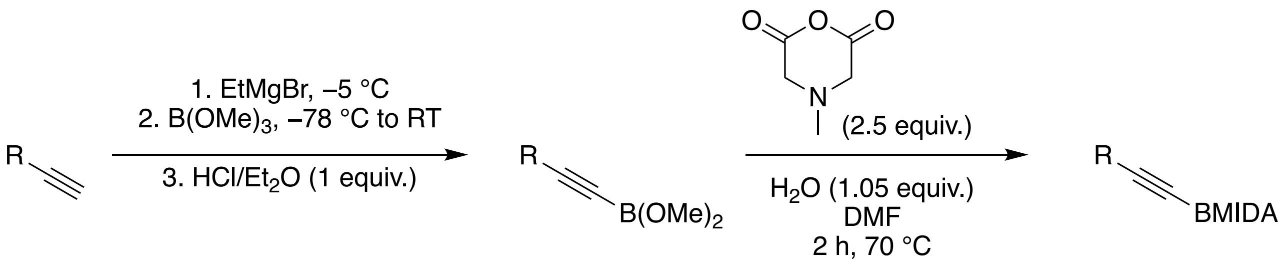

A flame dried Schlenk flask was evacuated and re-filled with N_2 three times and then charged with the alkyne (1.00 equiv.). Anhydrous THF (500 mM) was added, and the mixture was cooled to −5 °C. To this was added, dropwise, EtMgBr (1.05 equiv., ~3.00 M in Et_2O). The mixture was then stirred for 20 min at −5 °C before being gradually warmed to room temperature and stirred for 40 min. In a separate flame-dried flask, freshly distilled $B(OMe)_3$ (1.3 equiv.) was dissolved in anhydrous THF (2.0 mL per mmol of alkyne) under N_2 and cooled to −78 °C. The above Grignard solution was added to the $B(OMe)_3$ solution *via* syringe over 5 min and the mixture was allowed to gradually warm from −78 °C to approximately −20 °C over 1 h. The flask was then allowed to warm to room temperature and stirred for an additional 30 min. HCl (1.00 equiv., 2 M in Et_2O) was then added. The mixture was stirred for 5 min, after which anhydrous DMF (2.0 mL per mmol of alkyne) was added, and the ethereal solvents were removed on a rotary evaporator. The mixture was transferred to a sealed and oven-dried microwave vial containing MIDA anhydride (2.50 equiv.) under N_2. To this was added water (1.05 equiv.), and the mixture was stirred at 70 °C for 3 h. After cooling to room temperature, the mixture was diluted with 10% aq. LiCl (10 mL), which was then neutralized by addition of sat. aq. $NaHCO_3$ solution. The product was extracted with EtOAc/acetone (3:2, 2 × 10 mL per mmol of alkyne), the combined organic layers were washed with 10% aq. LiCl (4 × 10 mL per mmol of alkyne), and dried over anhydrous Na_2SO_4. The solvent was removed *in vacuo*. The product was either purified by dissolving in the minimum volume of CH_2Cl_2, followed by the addition of hexane/Et_2O (3:1, 4 mL per mmol of alkyne), collecting the precipitated product by filtration and washing with hexane/Et_2O (3:1), or flash column chromatography.

General procedure B: [2+2+2]Cycloaddition of diynes and internal borylated alkynes with basic cleavage.

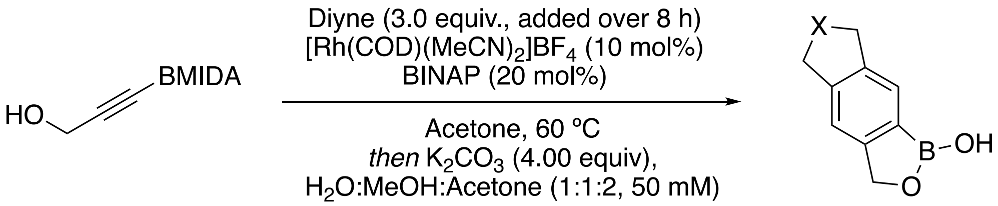

An oven dried microwave vial was charged with the relevant alkyne BMIDA (1.00 equiv.), $[Rh(COD)(MeCN)_2]BF_4$ (10 mol%), and BINAP (20 mol%). The vial was then sealed, evacuated, and backfilled with N_2. Acetone (100 mM/2) was added, and the solution was heated to 60 ºC. A separate flask was charged with the diyne (3.00 equiv.) and acetone (100 mM/2). This solution was added over 8 h using syringe pump addition. Once addition was complete, the mixture was allowed to stir for a further 1 h before allowing to cool to room temperature. The mixture was then filtered through celite and K_2CO_3 (4.00 equiv.) was added followed by MeOH:H_2O (100 mM). The mixture was allowed to stir for 1–5 h at room temperature before concentrating *in vacuo*. The conversion was determined through comparison to an internal standard *via* ^1H NMR spectroscopy using 1,4-dinitrobenzene (0.5 equiv.) as an internal standard.

General procedure C: [2+2+2]Cycloaddition of diynes and internal borylated alkynes with TBS protecting groups.

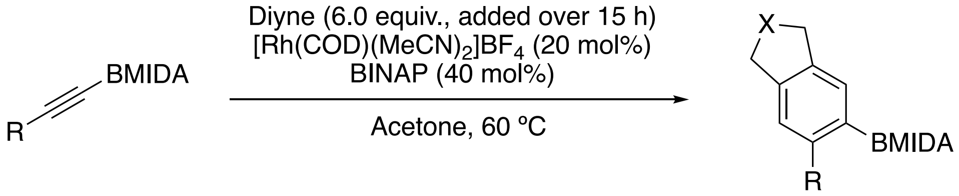

An oven dried microwave vial was charged with the relevant alkyne BMIDA (1.00 equiv.), $[Rh(COD)(MeCN)_2]BF_4$ (20.0 mol%), and BINAP (40.0 mol%). The vial was then sealed, evacuated, and backfilled with N_2. Acetone (100 mM/2) was added, and the solution was heated to 60 ºC. A separate flask was charged with the diyne (6.00 equiv.) and acetone (100 mM/2). This solution was added over 15 h using syringe pump addition. Once addition was complete, the mixture was allowed to stir for a further 1 h before allowing to cool to room temperature. The mixture was then filtered through celite and concentrated *in vacuo* to yield the crude product, which was purified by flash column chromatography.

General procedure D: TBS-MIDA deprotection.

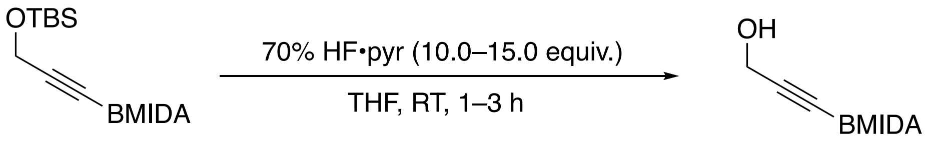

A flask was charged with the required TBS protected alkyne BMIDA (1.00 equiv.) and dry THF (200 mM). 70% HF•pyridine (10.0–15.0 equiv.) was added dropwise and the resulting solution was allowed to stir for 1–3 h before quenching with $NaHCO_3$ (until effervescence ceased). Na_2SO_4 and acetone (two reaction volumes) were then added, and the resulting slurry was stirred for 0.5 h before filtering through cotton and washing with excess acetone. The solution was concentrated *in vacuo* before purification by flash column chromatography, precipitation, or a combination thereof.

General procedure E: Propargylation of aldehydes to form homopropargyl alcohols.

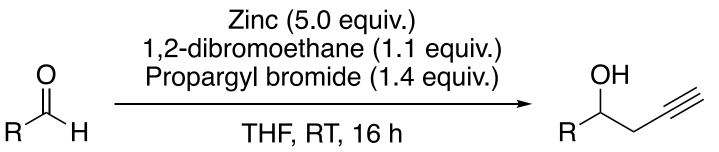

An oven dried microwave vial was charged with zinc (5.0 equiv.) and the aldehyde (1.0 equiv., if solid), sealed, evacuated, and backfilled with N_2. Dry THF (250 mM) was added followed by 1,2-dibromoethane (1.1 equiv.), then propargyl bromide (80% wt% in PhMe, 1.4 equiv.) and the aldehyde (1.0 equiv, if liquid). The resulting mixture was allowed to stir for 16 h at room temperature before unsealing and adding 2 M HCl (25 mL of per mmol of aldehyde). The mixture was filtered and EtOAc (50 mL per mmol of aldehyde) was added. The biphasic system was extracted with EtOAc (3 × 50 mL per mmol of aldehyde), and the combined organic layers were dried over Na_2SO_4, filtered, and concentrated *in vacuo* to yield the crude product, which was purified by flash column chromatography.

General Procedure F: TBS protection of alcohols.

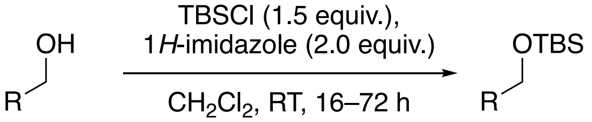

A flask was charged with the alcohol (1.0 equiv.) and dissolved in CH_2Cl_2 (200 mM). 1*H*-Imidazole (2.00 equiv.) and TBSCl (1.5 equiv.) were added, and the resulting solution was stirred for 16–72 h (16 h for primary alcohols, 72 h for secondary alcohols) at room temperature. The mixture was then quenched with the addition of water (50 mL of per mmol of alcohol) and the aqueous phase was extracted with CH_2Cl_2 (3 × 50 mL per mmol of alcohol). The combined organic layers were dried over Na_2SO_4, filtered, and concentrated *in vacuo* to yield the crude product, which was purified by flash column chromatography.

Starting Material Syntheses

T4-Methyl-*N*,*N*-di(prop-2-yn-1-yl)benzenesulfonamide (**S1**)

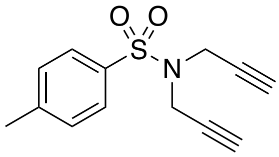

A flask was charged with 4-methylbenzenesulfonamide (8.56 g, 50.0 mmol, 1.00 equiv.), K_2CO_3 (41.5 g, 300 mmol, 6.00 equiv.), and MeCN (250 mL). Propargyl bromide (80 wt% in PhMe, 16.0 mL, 144 mmol, 2.87 equiv.) was added and the solution was then heated to 70 ºC to stir for 16 h. The solution was allowed to cool to room temperature, filtered, and concentrated *in vacuo* to yield the crude product, which was purified by flash column chromatography (silica, Et_2O) to yield the desired product as a pale-yellow solid (12.1 g, 98%).

^{1}H NMR (500 MHz, $CDCl_3$) δ_H 7.75 – 7.66 (m, 2H), 7.30 (d, J = 8.03 Hz, 2H), 4.17 (d, J = 2.35 Hz, 4H), 2.43 (s, 3H), 2.15 (t, J = 2.35 Hz, 2H).

^{13}C NMR (126 MHz, $CDCl_3$) δ_C 144.1, 135.3, 129.7, 128.0, 76.2, 74.2, 36.3, 21.7.

Spectral data in agreement with the literature.[5]

Dimethyl 2,2-di(prop-2-yn-1-yl)malonate (**S2**)

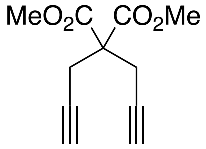

A flame-dried flask under N_2 was charged with dimethyl malonate (1.50 mL, 9.88 mmol, 1.00 equiv.), dry THF (30.0 mL) and propargyl bromide (80 wt% in PhMe, 3.10 mL, 28.8 mmol, 2.91 equiv.) and cooled to 0 ºC. NaH (60 wt% dispersion in oil, 988 mg, 24.7 mmol, 2.50 equiv.) was added in one portion and the reaction was allowed to stir at 0 ºC for 2 hours before allowing to warm to room temperature to stir for a further 4 hours. The reaction was quenched with sat. aq. NH_4Cl, extracted with EtOAc (5 × 20 mL), dried over Na_2SO_4, filtered, and concentrated *in vacuo* to yield the crude product, which was purified by flash column chromatography (silica, 0–5% Et_2O in hexane) to yield the desired product as a white solid (10.3 g, 94%).

^{1}H NMR (500 MHz, $CDCl_3$) δ_H 3.77 (s, 6H), 3.00 (d, J = 2.71 Hz, 4H), 2.03 (t, J = 2.64 Hz, 2H).

^{13}C NMR (126 MHz, $CDCl_3$) δ_C 169.0, 71.7, 56.5, 53.1, 29.7, 22.7.

Spectral data in agreement with the literature.[6]

Methyl 2-(prop-2-yn-1-yl)pent-4-ynoate (**S3**)

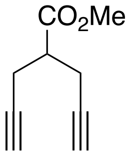

A flask equipped with reflux condenser was charged with dimethyl 2,2-di(prop-2-yn-1-yl)malonate (1.61 g, 7.74 mmol, 1.00 equiv.) and LiCl (984 mg, 23.2 mmol, 3.00 equiv.). DMSO (17.0 mL) and water (350 uL, 19.3 mmol, 2.50 equiv.) were added then the mixture was heated to reflux at 200 ºC for 4 h before allowing to cool to room temperature. Water (~50 mL) and chloroform (~50 mL) were added and the mixture was extracted with chloroform (3 × 50 mL). The organic layers were combined, dried over Na_2SO_4, filtered, and concentrated *in vacuo* to yield the crude product, which was purified by flash column chromatography (silica, 0–10% Et_2O in hexane) to yield the desired product as a yellow oil (483 mg, 38%).

^{1}H NMR (500 MHz, $CDCl_3$) δ_H 3.74 (s, 3H), 2.78 (tt, J = 7.21, 5.98 Hz, 1H), 2.71 – 2.58 (m, 4H), 2.02 (t, J = 2.63 Hz, 2H).

^{13}C NMR (126 MHz, $CDCl_3$) δ_C 172.8, 80.4, 70.5, 52.1, 43.0, 19.9.

Spectral data consistent with the literature.[7]

2-(Prop-2-yn-1-yl)pent-4-ynoic acid (**S4**)

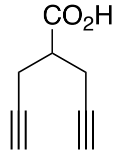

A flask equipped with reflux condenser was charged with methyl 2-(prop-2-yn-1-yl)pent-4-ynoate (314 mg, 2.09 mmol, 1.00 equiv.), MeOH (3.3 mL), and H_2O (3.0 mL). NaOH (234 mg, 5.86 mmol, 2.80 equiv.) was added and the mixture was heated to reflux at 200 ºC for 5 h. The mixture was then allowed to cool to room temperature and conc. HCl was added until no further turbidity appeared to be forming. The mixture was then extracted with EtOAc (3 × 50 mL). The combined organic layers were dried over Na_2SO_4, filtered, and concentrated *in vacuo* to yield the desired product as a thick, yellow oil, which was used directly with no further purification (290 mg, >99%).

^1H NMR (500 MHz, $CDCl_3$) δ_H 2.84 (tt, J = 7.15, 5.87 Hz, 1H), 2.77 – 2.64 (m, 4H), 2.07 (t, J = 2.67 Hz, 2H).

^{13}C NMR (126 MHz, $CDCl_3$) δ_C 178.2, 80.1, 70.8, 42.9, 19.6.

Spectral data consistent with the literature.[7]

tert-Butyl hepta-1,6-diyn-4-ylcarbamate (**S5**)

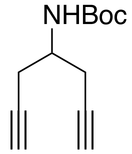

A flame dried flask equipped with a reflux condenser was charged with 2-(prop-2-yn-1-yl)pent-4-ynoic acid (750 mg, 5.51 mmol, 1.00 equiv.) and dry *t*-BuOH (15 mL). Diphenyl phosphoryl azide (1.30 mL, 6.06 mmol, 1.10 equiv.) and NEt_3 (900 µL, 6.45 mmol, 1.17 equiv.) were added and the mixture was heated to 100 ºC for 5 h. After allowing to cool to room temperature, the mixture was filtered through celite and concentrated *in vacuo* to yield the crude product, which was purified by flash column chromatography (silica, 0–5% EtOAc in hexane) to yield the desired product as a colorless oil (627 mg, 55%).

^1H NMR (400 MHz, $CDCl_3$) δ_H 4.85 (brs, 1H), 3.96 (brs, 1H), 2.71 – 2.45 (m, 4H), 2.06 (t, J = 2.65 Hz, 2H), 1.47 (s, 9H).

^{13}C NMR (101 MHz, $CDCl_3$) δ 155.0, 79.8, 79.8, 71.1, 47.6, 28.4, 23.3.

IR (ATR, film): 3298, 1694, 1503, 1366, 1250, 1231, 1163, 1049, 1022, 633 cm^{-1}.

HRMS (ESI): Calculated for 208.1332 *m/z*, found 208.1331 *m/z* $[C_{22}H_{17}N_1O_2+H]^+$.

N,N-Di(prop-2-yn-1-yl)methanesulfonamide (**S6**)

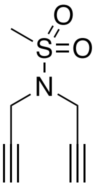

A flask equipped with reflux condenser was charged with methylsulfonamide (476 mg, 5.00 mmol, 1.00 equiv.), K_2CO_3 (4.15 g, 30.0 mmol, 6.00 equiv.), and MeCN (50 mL). Propargyl bromide (80 wt% in PhMe, 1.20 mL, 11.1 mmol, 2.22 equiv.) was added and the solution was then heated to 50 ºC to stir for 72 h. The solution was allowed to cool to room temperature, filtered, and concentrated *in vacuo* to yield the crude

product, which was purified by flash column chromatography (silica, Et_2O) to yield the desired product as a white solid (853 mg, >99%).

^{1}H NMR (400 MHz, $CDCl_3$) δ_H 4.19 (d, J = 2.46 Hz, 4H), 2.98 (s, 3H), 2.39 (t, J = 2.36 Hz, 2H).

^{13}C NMR (101 MHz, $CDCl_3$) δ_C 76.7, 74.7, 38.7, 36.6.

Spectral data in agreement with the literature.[8]

4-Bromo-*N*,*N*-di(prop-2-yn-1-yl)benzenesulfonamide (**S7**)

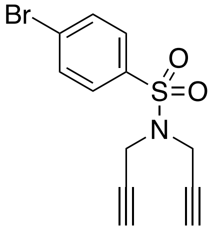

A flask equipped with reflux condenser was charged with 4-bromobenzenesulfonamide (2.48 g, 10.5 mmol, 1.00 equiv.), K_2CO_3 (8.71 g, 63.0 mmol, 6.00 equiv.), and MeCN (100 mL). Propargyl bromide solution (80 wt% in PhMe, 2.50 mL, 23.3 mmol, 2.22 equiv.) was added and the resulting mixture was heated to reflux at 80 ºC for 5 h. After allowing to cool to room temperature, the mixture was filtered, washing with MeCN, then concentrated *in vacuo* to yield the crude product, which was purified by flash column chromatography (silica, 0–25% EtOAc in hexane) to yield the desired product as a yellow solid (2.70 g, 82%)

^{1}H NMR (500 MHz, $CDCl_3$) δ_H 7.78 – 7.70 (m, 2H), 7.70 – 7.65 (m, 2H), 4.19 (d, J = 2.39 Hz, 4H), 2.20 (t, J = 2.38 Hz, 2H).

^{13}C NMR (126 MHz, $CDCl_3$) δ_C 137.2, 132.3, 129.4, 128.3, 75.8, 74.4, 36.3.

Spectral data consistent with the literature.[9]

tert-Butyl di(prop-2-yn-1-yl)carbamate (**S8**)

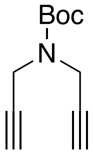

A flame-dried flask was charged with *N*-Boc propargyl amine (5.46 g, 35.2 mmol, 1.00 eqiuv) and dry THF (70 mL) under N_2. After cooling to 0 ºC, NaH (60 wt% dispersion in oil, 1.68 g, 42.0 mmol, 1.20 equiv.) was added followed by dropwise addition of propargyl bromide (80 wt% in PhMe, 5.00 mL, 45.0 mmol, 1.26 equiv.) and the mixture was allowed to stir for 4 h whilst allowing to warm to room temperature. The reaction was quenched with water (100 mL) and extracted with CH_2Cl_2 (3 × 50 mL). The combined organic layers were dried with Na_2SO_4, filtered, and concentrated *in vacuo* to yield the crude product, which was purified by flash column chromatography (silica, 0–5% EtOAc in hexane) to yield the desired product as a yellow oil (5.31 g, 78%).

^{1}H NMR (500 MHz, $CDCl_3$) δ_H 4.17 (brs, 4H), 2.22 (t, J = 2.47 Hz, 2H), 1.48 (s, 9H).

^{13}C NMR (126 MHz, $CDCl_3$) δ_C 154.4, 81.3, 78.9, 72.0, 35.3, 28.4.

Spectral data consistent with the literature.[10]

Diethyl 2,2-di(prop-2-yn-1-yl)malonate (**S9**)

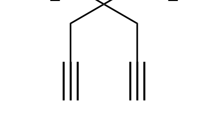

A flame-dried flask was charged with diethyl malonate (3.00 mL, 19.8 mmol, 1.00 equiv.), dry THF (60.0 mL) and propargyl bromide (80 wt% in PhMe, 6.20 mL, 57.5 mmol, 2.91 equiv.) and cooled to 0 ºC under N_2. NaH (60 wt% dispersion in oil, 1.98 g, 49.4 mmol, 2.50 equiv.) was added in one portion and the reaction was allowed to stir at 0 ºC for 2 h before allowing to warm to room temperature to stir for a further 4 h. The reaction was quenched with sat. aq. NH_4Cl (100 mL), extracted with CH_2Cl_2 (5 × 20 mL), dried over Na_2SO_4, filtered, and concentrated *in vacuo* to yield the crude product, which was purified by flash column chromatography (silica, 0–5% EtOAc in hexane) to yield the desired product as a colorless oil, which solidifies on cooling into a white solid (4.67 g, 19.8 mmol, >99%).

^{1}H NMR (500 MHz, $CDCl_3$) δ_H 4.25 (q, J = 7.14 Hz, 4H), 3.01 (d, J = 2.67 Hz, 4H), 2.05 (t, J = 2.63 Hz, 2H), 1.28 (t, J = 7.13 Hz, 6H).

^{13}C NMR (126 MHz, $CDCl_3$) δ_C 168.6, 71.7, 62.1, 56.3, 43.0, 22.5, 14.0.

Spectral data in agreement with the literature.[5]

<u>4-((*tert*-Butyldimethylsilyl)oxy)but-2-yn-1-ol (**S10**)</u>

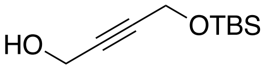

A flame-dried flask was charged with NaH (60 wt% dispersion in oil, 1.85 g, 46.2 mmol, 1.20 equiv.) and THF (25.0 mL) under N_2. After cooling to 0 ºC, but-2-yne-1,4-diol (9.94 g, 115 mmol, 3.00 equiv.) in THF (25 mL) was added dropwise *via* cannula addition. The resulting solution was allowed to stir at 0 ºC for 30 mins before TBSCl (5.80 g, 38.5 mmol, 1.00 equiv.) was added. The solution was allowed to stir for 2 h at 0 ºC then quenched with the addition of sat. aq. NH_4Cl (50 mL). The mixture was extracted with Et_2O (3 × 100 mL). The combined organic layers were dried over Na_2SO_4, filtered, and concentrated *in vacuo* to yield the crude product, which was purified by flash column chromatography (silica, 0–25% EtOAc in hexane) to yield the desired product as a colorless oil (6.54 g, 32.6 mmol, 85%).

^{1}H NMR (400 MHz, $CDCl_3$) δ_H 4.38 (t, J = 1.84 Hz, 2H), 4.32 (t, J = 1.83 Hz, 2H), 0.93 (s, 9H), 0.14 (s, 6H).

^{13}C NMR (126 MHz, $CDCl_3$) δ_C 85.1, 82.4, 81.9, 49.1, 24.3, 16.7, −5.0.

Spectral data consistent with the literature.[11]

<u>*tert*-Butyldimethyl((4-(prop-2-yn-1-yloxy)but-2-yn-1-yl)oxy)silane (**S11**)</u>

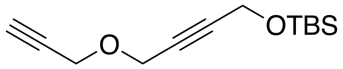

A flask was charged with 4-((*tert*-butyldimethylsilyl)oxy)but-2-yn-1-ol (3.01 g, 15.0 mmol, 1.00 equiv.) and dry THF (100 mL) under N_2. The flask was cooled to 0 ºC before addition of NaH (60 wt% dispersion in oil, 660 mg, 16.5 mmol, 1.10 equiv.) in small portions. The resulting mixture was stirred for 10 mins before propargyl bromide (80 wt% in PhMe, 1.93 mL, 18.0 mmol, 1.20 equiv.) was added. The mixture was allowed to stir for 1 h at room temperature before quenching with MeOH. Water (100 mL) was added and the mixture was extracted with Et_2O (3 × 50 mL). The combined organic layers were dried over Na_2SO_4, filtered, and concentrated *in vacuo* to yield the crude product, which was purified by flash column chromatography (silica, 0–10% Et_2O in hexane) to yield the desired product as a colorless oil (1.74 g, 49%).

^{1}H NMR (500 MHz, $CDCl_3$) δ_H 4.38 (t, J = 1.85 Hz, 2H), 4.32 (t, J = 1.85 Hz, 2H), 4.28 (d, J = 2.42 Hz, 2H), 2.46 (t, J = 2.40 Hz, 1H), 0.93 (s, 9H), 0.15 (s, 6H).

^{13}C NMR (126 MHz, $CDCl_3$) δ_C 85.7, 79.8, 78.9, 74.9, 56.8, 56.4, 51.7, 25.8, 18.3, −5.2.

Spectral data consistent with the literature.[12]

<u>4-Methyl-*N*-(prop-2-yn-1-yl)benzenesulfonamide (**S12**)</u>

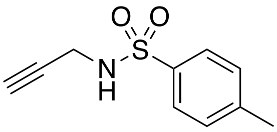

A flask was charged with NEt_3 (5.70 mL, 41.0 mmol, 2.00 equiv.), propargyl amine (1.30 mL, 20.4 mmol, 1.00 equiv.), and CH_2Cl_2 (40.0 mL) under N_2. After cooling to 0 ºC, TsCl (4.28 g, 22.5 mmol, 1.10 equiv.) was added and the mixture was allowed to stir at 0 ºC for 1 h then allowed to warm to room temperature to stir for 5 h. The reaction was quenched with water (50 mL) and extracted with CH_2Cl_2 (3 × 100 mL). The combined organic layers were dried over Na_2SO_4, filtered, and concentrated *in vacuo* to yield the crude product, which was purified by flash column chromatography (silica, Et_2O) to yield the desired product as a pale-yellow solid (988 mg, 94%).

^{1}H NMR (500 MHz, $CDCl_3$) δ_H 7.81 – 7.78 (m, 2H), 7.34 (dt, *J* = 7.86, 0.73 Hz, 2H), 4.57 (s, 1H), 3.86 (dd, *J* = 6.04, 2.54 Hz, 2H), 2.46 (s, 3H), 2.13 (t, *J* = 2.55 Hz, 1H).

^{13}C NMR (126 MHz, $CDCl_3$) δ_C 143.9, 136.5, 129.7, 127.4, 79.4, 73.0, 32.9, 21.6.

Spectral data consistent with the literature.[13]

N-(But-2-yn-1-yl)-4-methyl-*N*-(prop-2-yn-1-yl)benzenesulfonamide (**S13**)

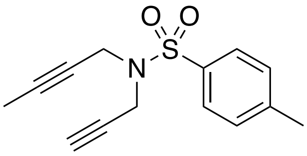

A flask equipped with a reflux condenser was charged with 4-methyl-*N*-(prop-2-yn-1-yl)benzenesulfonamide (250 mg, 1.19 mmol, 1.00 equiv.), K_2CO_3 (495 mg, 3.58 mmol, 3.00 equiv.), and MeCN (6.00 mL). 1-Bromobut-2-yne (160 µL, 1.83 mmol, 1.53 equiv.) was added and the mixture was heated to reflux at 80 ºC for 4 h before allowing to cool to room temperature. The mixture was filtered and concentrated *in vacuo* to yield the crude product, which was purified by flash column chromatography (silica, 0–20% EtOAc in hexane) to yield the desired product as a white solid (311 mg, >99%).

^{1}H NMR (500 MHz, $CDCl_3$) δ_H 7.76 – 7.71 (m, 2H), 7.35 – 7.30 (m, 2H), 4.16 (dd, *J* = 2.55, 0.78 Hz, 2H), 4.12 (q, *J* = 2.36, 0.70 Hz, 2H), 2.45 (s, 3H), 2.15 (t, *J* = 2.48 Hz, 1H), 1.67 (t, *J* = 2.38 Hz, 3H).

^{13}C NMR (126 MHz, $CDCl_3$) δ_C 143.9, 135.5, 129.5, 128.1, 82.1, 76.9, 73.8, 71.4, 36.8, 36.3, 21.7, 3.6.

Spectral data in agreement with literature.[14]

4-Methyl-*N*-(3-phenylprop-2-yn-1-yl)-*N*-(prop-2-yn-1-yl)benzenesulfonamide (**S14**)

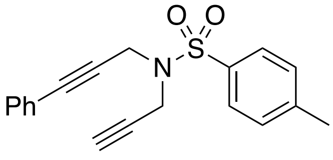

A flask was charged with 4-methyl-*N*-(prop-2-yn-1-yl)benzenesulfonamide (765 mg, 3.66 mmol, 1.20 equiv.), triphenylphosphine (959 mg, 3.66 mmol, 1.20 equiv.) and THF (30.0 mL) and cooled in a water bath. Diisopropyl (*E*)-diazene-1,2-dicarboxylate (830 µL, 4.23 mmol, 1.39 equiv.) and 3-phenylprop-2-yn-1-ol (380 µL, 3.05 mmol, 1.00 equiv.) were added and the reaction mixture was stirred for 3 h at room temperature. The mixture was filtered through celite (washing with Et_2O) and concentrated *in vacuo* to yield the crude product, which was purified by flash column chromatography (silica, 0–20% Et_2O in hexane) to yield the desired product as a white solid (292 mg, 30%).

^{1}H NMR (500 MHz, $CDCl_3$) δ_H 7.81 – 7.68 (m, 2H), 7.33 – 7.22 (m, 5H), 7.17 (dt, *J* = 6.86, 1.53 Hz, 2H), 4.41 (s, 2H), 4.20 (d, *J* = 2.48 Hz, 2H), 2.36 (s, 3H), 2.19 (t, *J* = 2.49 Hz, 1H).

^{13}C NMR (126 MHz, $CDCl_3$) δ_C 143.9, 135.3, 131.6, 129.6, 128.5, 128.2, 128.0, 122.1, 85.9, 81.3, 76.7, 74.0, 37.2, 36.5, 21.5.

Spectral data consistent with the literature. [14]

1-Phenylbut-3-yn-1-ol (**S15**)

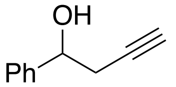

Prepared according to **General Procedure E** from benzaldehyde (400 µL, 3.95 mmol, 1.00 equiv.). Purified by flash column chromatography (silica, 0–15% EtOAc in hexane) to yield the desired product as a colorless oil (222 mg, 38%).

^{1}H NMR (500 MHz, $CDCl_3$) δ_H 7.44 – 7.36 (m, 4H), 7.36 – 7.30 (m, 1H), 4.91 (t, *J* = 6.35 Hz, 1H), 2.68 (d, *J* = 2.67 Hz, 1H), 2.67 (dd, *J* = 2.63, 1.37 Hz, 1H), 2.10 (t, *J* = 2.65 Hz, 1H).

^{13}C NMR (126 MHz, $CDCl_3$) δ_C 142.5, 128.5, 128.0, 125.8, 80.7, 72.4, 71.0, 29.5.

Spectral data consistent with the literature.[15]

tert-Butyldimethyl((1-phenylbut-3-yn-1-yl)oxy)silane (**S16**)

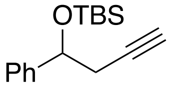

Prepared according to **General Procedure F** from 1-phenylbut-3-yn-1-ol (300 mg, 2.05 mmol, 1.00 equiv.). Purified by flash column chromatography (silica, hexane) to yield the desired product as a colorless oil (483 mg, 90%).

^{1}H NMR (500 MHz, $CDCl_3$) δ_H 7.43 – 7.33 (m, 4H), 7.32 – 7.25 (m, 1H), 4.84 (dd, *J* = 7.12, 5.72 Hz, 1H), 2.61 (ddd, *J* = 16.63, 7.11, 2.63 Hz, 1H), 2.51 (ddd, *J* = 16.65, 5.73, 2.66 Hz, 1H), 1.98 (t, *J* = 2.63 Hz, 1H), 0.91 (s, 9H), 0.10 (s, 3H), −0.05 (s, 3H).

^{13}C NMR (126 MHz, $CDCl_3$) δ_C 144.0, 128.1, 127.5, 125.9, 73.7, 69.9, 31.0, 25.8, 18.3, −4.7, −4.9.

Spectral data consistent with literature.[16]

2-(4-((*tert*-Butyldimethylsilyl)oxy)-4-phenylbut-1-yn-1-yl)-6-methyl-1,3,6,2-dioxazaborocane-4,8-dione (**S17**)

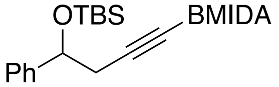

Prepared according to **General Procedure A** from *tert*-butyldimethyl((1-phenylbut-3-yn-1-yl)oxy)silane (651 mg, 696 µL, 1.00 equiv., 2.50 mmol). Purified by precipitation from hexane/Et_2O to afford the desired product as a white powder (356.5 mg, 34%).

^{1}H NMR (500 MHz, Acetone-*d*$_6$) δ_H 7.43 (d, *J* = 7.05 Hz, 2H), 7.34 (t, *J* = 7.53 Hz, 2H), 7.28 – 7.23 (m, 1H), 4.93 (t, *J* = 6.36 Hz, 1H), 4.19 (m, 2H), 3.91 (m, 2H), 2.97 (s, 3H), 2.70 – 2.53 (m, 2H), 0.89 (s, 9H), 0.12 (s, 3H), −0.06 (s, 3H).

^{13}C NMR (126 MHz, Acetone-*d*$_6$) δ_C 168.5, 168.4, 145.1, 128.9, 128.2, 127.0, 98.7, 74.4, 62.0, 62.0, 48.1, 32.2, 26.2, 18.8, −4.6, −4.7.

^{11}B NMR (128 MHz, Acetone-*d*$_6$) δ_B 5.8.

IR (ATR, film): 3213, 2955, 2207, 1769, 1458, 1285, 1254, 1165, 1034, 841, 777 cm^{-1}.

HRMS (ESI): Calculated for 438.1879 *m/z*, found 438,1878 *m/z* $[C_{21}H_{30}BN_1O_5Si+Na]^+$.

2-(4-Hydroxy-4-phenylbut-1-yn-1-yl)-6-methyl-1,3,6,2-dioxazaborocane-4,8-dione (**S18**)

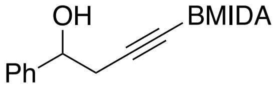

Prepared according to **General Procedure D** from 2-(4-((*tert*-butyldimethylsilyl)oxy)-4-phenylbut-1-yn-1-yl)-6-methyl-1,3,6,2-dioxazaborocane-4,8-dione (83.1 mg, 200 μmol, 1.00 equiv.). Purified by precipitation from acetone-CH_2Cl_2:Et_2O-hexane to yield the desired product as a white solid (11.6 mg, 18%).

^{1}H NMR (400 MHz, Acetone-d_6) δ_H 7.49 – 7.42 (m, 2H), 7.34 (tt, J = 6.69, 0.83 Hz, 2H), 7.29 – 7.23 (m, 1H), 4.87 (t, J = 6.39 Hz, 1H), 4.20 (d, J = 16.92 Hz, 2H), 3.94 (dd, J = 16.93, 9.14 Hz, 2H), 2.99 (s, 3H), 2.78 – 2.58 (m, 2H).

^{13}C NMR (101 MHz, Acetone-d_6) δ_C 167.8, 167.7, 144.4, 128.0, 127.2, 126.1, 98.6, 72.0, 61.1, 61.1, 47.2, 30.2.

^{11}B NMR (128 MHz, Acetone-d_6) δ_B 6.1.

IR (ATR, film): 2205, 1744, 1454, 1290, 1165, 1026, 985, 860, 702 cm^{-1}.

HRMS (ESI): Calculated for 324.1014 *m*/*z*, found 324.1016 *m*/*z* [$C_{15}H_{16}BNO_5$+Na]$^+$.

1-(*o*-Tolyl)but-3-yn-1-ol (**S19**)

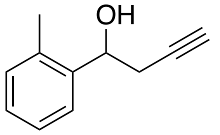

Prepared according to **General Procedure E** from 2-methylbenzaldehyde (1.40 mL,12.1 mmol, 1.00 equiv.). Purified by flash column chromatography (silica, 0-20% EtOAc in hexane) to yield the desired product as a yellow oil (1.45 g, 75%).

^{1}H NMR (500 MHz, $CDCl_3$) δ_H 7.52 (dd, J = 7.63, 1.56 Hz, 1H), 7.24 (dd, J = 7.55, 1.65 Hz, 1H), 7.21 (td, J = 7.36, 1.59 Hz, 1H), 7.16 – 7.14 (m, 1H), 5.12 (dd, J = 7.52, 5.18 Hz, 1H), 2.63 – 2.58 (m, 2H), 2.36 (s, 3H), 2.09 (t, J = 2.63 Hz, 1H).

^{13}C NMR (126 MHz, $CDCl_3$) δ_C 140.5, 134.6, 130.5, 127.8, 126.4, 125.1, 81.0, 70.8, 68.9, 28.3, 19.1.

Spectral data consistent with the literature.[17]

tert-Butyldimethyl((1-(*o*-tolyl)but-3-yn-1-yl)oxy)silane (**S20**)

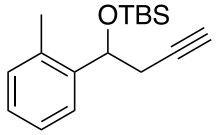

Prepared according to **General Procedure F** from 1-(*o*-tolyl)but-3-yn-1-ol (1.20 g, 7.52 mmol, 1.00 equiv.). Purified by flash column chromatography (silica, 0–5% Et_2O in hexane) to yield the desired compound as a colorless oil (1.76 g, 85%).

^{1}H NMR (500 MHz, $CDCl_3$) δ_H 7.51 (dd, J = 7.64, 1.54 Hz, 1H), 7.22 (td, J = 7.47, 1.54 Hz, 1H), 7.17 (td, J = 7.38, 1.58 Hz, 1H), 7.14 – 7.09 (m, 1H), 5.08 (dd, J = 7.71, 4.86 Hz, 1H), 2.56 (ddd, J = 16.76, 7.72, 2.61 Hz, 1H), 2.48 (ddd, J = 16.75, 4.88, 2.67 Hz, 1H), 2.37 (s, 3H), 1.97 (t, J = 2.64 Hz, 1H), 0.90 (s, 9H), 0.09 (s, 3H), −0.07 (s, 3H).

^{13}C NMR (126 MHz, $CDCl_3$) δ_C 142.2, 133.6, 130.1, 127.2, 126.2, 126.0, 82.0, 70.3, 69.5, 29.8, 25.8, 19.2, 18.3, −4.8, −5.0.

IR (ATR, film): 2928, 1252, 1078, 930, 835, 810, 775, 754, 723, 637, 625 cm^{-1}.

HRMS (ESI): Calculated for 297.1645 *m*/*z*, found 297.1634 *m*/*z* [$C_{17}H_{26}OSi$+Na]$^+$.

2-(4-((*tert*-Butyldimethylsilyl)oxy)-4-(*o*-tolyl)but-1-yn-1-yl)-6-methyl-1,3,6,2-dioxazaborocane-4,8-dione (**S21**)

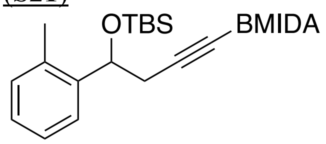

Prepared according to **General Procedure A** from *tert*-butyldimethyl((1-(*o*-tolyl)but-3-yn-1-yl)oxy)silane (549 mg, 2.00 mmol, 1.00 equiv,). Purified by precipitation from hexane/Et_2O to afford the desired product as a white powder (350.0 mg, 41%).

^{1}H NMR (500 MHz, Acetone-d_6) δ_H 7.50 (dd, J = 7.6, 1.7 Hz, 1H), 7.23 – 7.18 (m, 1H), 7.17 – 7.10 (m, 2H), 5.16 (t, J = 6.36 Hz, 1H), 4.19 (m, 2H), 3.93 (d, J = 16.99 Hz, 1H), 3.87 (d, J = 16.88 Hz, 1H), 2.96 (s, 3H), 2.61 (dd, J = 6.41, 5.13 Hz, 2H), 2.38 (s, 3H), 0.88 (s, 9H), 0.11 (s, 3H), −0.08 (s, 3H).

^{13}C NMR (126 MHz, Acetone-d_6) δ_C 168.5, 168.4, 143.1, 134.8, 130.9, 128.0, 127.1, 126.7, 99.3, 70.9, 62.0, 62.0, 48.0, 31.1, 26.1, 19.4, 18.7, −4.7, −4.7.

^{11}B NMR (128 MHz, Acetone-d_6) δ_B 6.0.

IR (ATR, film): 2955, 2205, 1771, 1458, 1287, 1165, 1034, 837, 777 cm^{-1}.

HRMS (ESI): Calculated for 430.2216 *m*/*z*, found 430.2207 *m*/*z* $[C_{22}H_{32}BNO_5Si+H]^+$.

2-(4-Hydroxy-4-(*o*-tolyl)but-1-yn-1-yl)-6-methyl-1,3,6,2-dioxazaborocane-4,8-dione (**S22**)

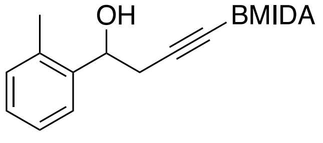

Prepared according **General Procedure D** from 2-(4-((*tert*-butyldimethylsilyl)oxy)-4-(*o*-tolyl)but-1-yn-1-yl)-6-methyl-1,3,6,2-dioxazaborocane-4,8-dione (85.9 mg, 200 µmol, 1.00 equiv.). Purified by flash column chromatography (silica, 0–100% Acetone in CH_2Cl_2) to yield the desired product as a white solid (49.9 mg, 79%).

^{1}H NMR (700 MHz, Acetone-d_6) δ_H 7.54 (d, J = 7.67 Hz, 1H), 7.22 – 7.18 (m, 1H), 7.17 – 7.12 (m, 2H), 5.13 (t, J = 6.56 Hz, 1H), 4.48 (s, 1H), 4.20 (d, J = 16.93 Hz, 2H), 3.97 (d, J = 16.96 Hz, 1H), 3.91 (d, J = 16.93 Hz, 1H), 2.98 (s, 3H), 2.69 – 2.63 (m, 2H), 2.37 (s, 3H).

^{13}C NMR (176 MHz, Acetone-d_6) δ_C 167.8, 167.7, 142.4, 134.7, 130.0, 127.0, 125.8, 125.8, 98.7, 68.3, 61.1, 61.1, 47.2, 29.8, 18.4.

^{11}B NMR (128 MHz, Acetone-d_6) δ_B 6.1.

IR (ATR, film): 2209, 1746, 1705, 1458, 1339, 1290, 1165, 1028, 988, 891, 858, 761, 704 cm^{-1}.

HRMS (ESI): Calculated for 315.1273 *m*/*z*, found 315.1272 *m*/*z* $[C_{16}H_{18}BNO_5]^+$.

1-(*m*-Tolyl)but-3-yn-1-ol (**S23**)

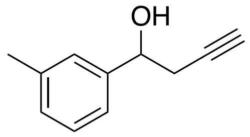

Prepared according to **General Procedure E** from 3-methylbenzaldehyde (1.40 mL, 11.9 mmol, 1.00 equiv.). Purified by flash column chromatography (silica, 0-20% EtOAc in hexane) to yield the desired product as a yellow oil (1.48 g, 78%).

^{1}H NMR (500 MHz, $CDCl_3$) δ_H 7.26 (t, J = 7.54 Hz, 1H), 7.21 (m, 1H), 7.20 – 7.16 (m, 1H), 7.13 (m, 1H), 4.82 (t, J = 6.40 Hz, 1H), 2.63 (dd, J = 6.43, 2.62 Hz, 2H), 2.38 (d, J = 0.89 Hz, 3H), 2.08 (t, J = 2.65 Hz, 1H).

^{13}C NMR (126 MHz, $CDCl_3$) δ_C 142.5, 138.2, 128.8, 128.4, 126.5, 122.9, 80.9, 72.4, 70.9, 29.4, 21.5.

Spectral data consistent with the literature.[17]

tert-Butyldimethyl((1-(*m*-tolyl)but-3-yn-1-yl)oxy)silane (**S24**)

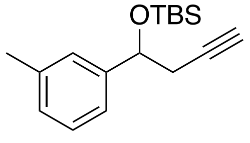

Prepared according to **General Procedure F** from 1-(*m*-tolyl)but-3-yn-1-ol (1.17 g, 7.30 mmol, 1.00 equiv.). Purified by flash column chromatography (silica, 0–5% Et_2O in hexane) to yield the desired compound as a colorless oil (1.56 g, 78%).

^{1}H NMR (400 MHz, $CDCl_3$) δ_H 7.26 – 7.20 (m, 1H), 7.20 – 7.15 (m, 2H), 7.12 – 7.07 (m, 1H), 4.81 (dd, J = 7.22, 5.58 Hz, 1H), 2.60 (ddd, J = 16.58, 7.22, 2.60 Hz, 1H), 2.50 (ddd, J = 16.61, 5.60, 2.67 Hz, 1H), 2.37 (d, J = 0.73 Hz, 3H), 1.98 (t, J = 2.64 Hz, 1H), 0.91 (s, 9H), 0.10 (s, 3H), −0.05 (s, 3H).

^{13}C NMR (101 MHz, $CDCl_3$) δ_C 144.0, 137.6, 128.2, 128.0, 126.5, 123.0, 81.2, 73.8, 69.8, 31.0, 25.8, 21.5, 18.3, −4.7, −4.9.

IR (ATR, film): 2928, 2857, 1472, 1252, 1101, 1082, 936, 835, 810, 775, 702, 665, 633, 446 cm^{-1}.

HRMS (ESI): Calculated for 297.1645 *m/z*, found 297.1645 *m/z* $[C_{17}H_{26}OSi+Na]^+$.

2-(4-((*tert*-Butyldimethylsilyl)oxy)-4-(*m*-tolyl)but-1-yn-1-yl)-6-methyl-1,3,6,2-dioxazaborocane-4,8-dione (**S25**)

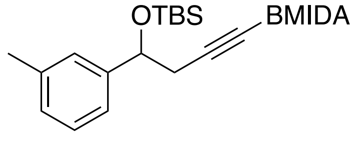

Prepared according to **General Procedure A** from *tert*-butyldimethyl((1-(*m*-tolyl)but-3-yn-1-yl)oxy)silane (477 mg, 2.00 mmol, 1.00 equiv.). Purified by precipitation from hexane/Et_2O to afford a white powder (327.2 mg, 38%).

^{1}H NMR (500 MHz, Acetone-d_6) δ_H 7.25 – 7.24 (m, 1H), 7.21 (dd, J = 5.07, 0.85 Hz, 2H), 7.07 (td, J = 4.18, 1.41 Hz, 1H), 4.89 (t, J = 6.33 Hz, 1H), 4.19 (m, 2H; H[16]), 3.90 (m, 2H), 2.98 (s, 3H), 2.65 (dd, J = 16.67, 6.31 Hz, 1H), 2.57 (dd, J = 16.67, 6.38 Hz, 1H), 2.32 (s, 3H), 0.89 (s, 9H), 0.11 (s, 3H), −0.06 (s, 3H).

^{13}C NMR (126 MHz, Acetone-d_6) δ_C 168.5, 168.4, 145.0, 138.2, 128.9, 128.8, 127.6, 124.0, 99.3, 74.4, 61.9, 61.9, 48.0, 32.2, 26.2, 21.5, 18.7, −4.6, −4.7.

^{11}B NMR (128 MHz, Acetone-d_6) δ 6.0.

IR (ATR, film): 3198, 2955, 2205, 1787, 1458, 1283, 1252, 1165, 1030, 986, 835, 775, 703 cm^{-1}.

HRMS (ESI): Calculated for 452.2035 *m/z*, found 452.2028 *m/z* $[C_{22}H_{32}BNO_5Si+Na]^+$.

2-(4-Hydroxy-4-(*m*-tolyl)but-1-yn-1-yl)-6-methyl-1,3,6,2-dioxazaborocane-4,8-dione (**S26**)

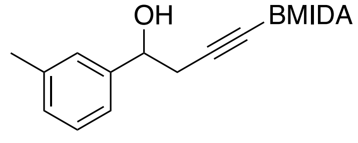

Prepared according **General Procedure D** from 2-(4-((*tert*-butyldimethylsilyl)oxy)-4-(*m*-tolyl)but-1-yn-1-yl)-6-methyl-1,3,6,2-dioxazaborocane-4,8-dione (85.9 mg, 200 μmol, 1.00 equiv.). Purified by flash column

chromatography (silica, 0–100% Acetone in CH_2Cl_2) to yield the desired product as a white solid (48.1 mg, 76%).

^{1}H NMR (700 MHz, Acetone-d_6) δ_H 7.27 (m, 1H), 7.25 – 7.20 (m, 2H), 7.10 – 7.06 (m, 1H), 4.82 (t, J = 6.42 Hz, 1H), 4.53 (brs, 1H), 4.20 (m, 2H), 4.00 – 3.89 (m, 2H), 3.00 (s, 3H), 2.68 (dd, J = 16.73, 6.49 Hz, 1H), 2.63 (dd, J = 16.69, 6.34 Hz, 1H), 2.33 (s, 3H).

^{13}C NMR (176 MHz, Acetone-d_6) δ_C 167.8, 167.7, 144.4, 137.3, 127.9, 127.8, 126.8, 123.2, 98.7, 72.0, 61.1, 61.1, 47.2, 30.2, 20.6.

^{11}B NMR (128 MHz, Acetone-d_6) δ_B 6.0.

IR (ATR, film): 1765, 1339, 1289, 1165, 1146, 1028, 988, 860, 793, 704, 419 cm^{-1}.

HRMS (ESI): Calculated for 338.1170 *m/z*, found 338.1177 *m/z* $[C_{16}H_{18}BNO_5+Na]^+$.

1-(*p*-Tolyl)but-3-yn-1-ol (**S27**)

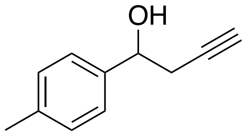

Prepared according to **General Procedure E** from 4-methylbenzaldehyde (1.40 mL, 11.9 mmol, 1.00 equiv.). Purified by flash column chromatography (silica, 0–20% EtOAc in hexane) to yield the desired product as a yellow oil (741 mg, 39%).

^{1}H NMR (400 MHz, $CDCl_3$) δ_H 7.34 – 7.29 (m, 2H), 7.23 – 7.17 (m, 2H), 4.88 (t, J = 6.36 Hz, 1H), 2.66 (dd, J = 6.36, 2.64 Hz, 2H), 2.37 (s, 3H), 2.10 (t, J = 2.63 Hz, 1H).

^{13}C NMR (126 MHz, $CDCl_3$) δ_C 139.5, 137.8, 129.2, 125.7, 80.8, 72.2, 70.9, 29.4, 21.2.

Spectral data consistent with the literature.[17]

tert-Butyldimethyl((1-(*p*-tolyl)but-3-yn-1-yl)oxy)silane (**S28**)

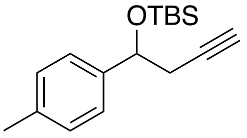

Prepared according to **General Procedure F** from 1-(*p*-tolyl)but-3-yn-1-ol (694 mg, 4.35 mmol, 1.00 equiv.). Purified by flash column chromatography (silica, 0–5% Et_2O in hexane) to yield the desired compound as a colorless oil (1.01 g, 84%).

^{1}H NMR (400 MHz, $CDCl_3$) δ_H 7.28 – 7.19 (m, 2H), 7.19 – 7.10 (m, 2H), 4.84 – 4.72 (m, 1H), 2.60 (ddd, J = 16.59, 7.09, 2.65 Hz, 1H), 2.49 (ddd, J = 16.59, 5.80, 2.65 Hz, 1H), 2.36 (s, 3H), 1.98 (t, J = 2.64 Hz, 1H), 0.91 (s, 9H), 0.09 (s, 3H), −0.05 (s, 3H).

^{13}C NMR (101 MHz, $CDCl_3$) δ_C 141.0, 137.0, 128.8, 125.8, 81.3, 73.6, 69.8, 25.8, 21.2, 18.3, −4.7, −4.9.

IR (ATR, film): 2928, 1252, 1084, 930, 833, 810, 775, 637, 538 cm^{-1}.

HRMS (ESI): Calculated for 297.1645 *m/z*, found 297.1637 *m/z* $[C_{17}H_{26}OSi+Na]^+$.

2-(4-((*tert*-Butyldimethylsilyl)oxy)-4-(*p*-tolyl)but-1-yn-1-yl)-6-methyl-1,3,6,2-dioxazaborocane-4,8-dione (**S29**)

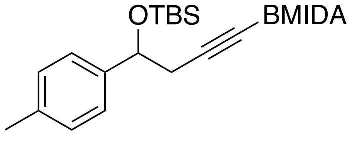

Prepared according to **General Procedure A** from *tert*-butyldimethyl((1-(*p*-tolyl)but-3-yn-1-yl)oxy)silane (549 mg, 607 µL, 1.00 equiv., 2.00 mmol). Purified by precipitation from hexane/Et_2O to afford the desired product as a white powder (411.9 mg, 48%).

^{1}H NMR (400 MHz, Acetone-d_6) δ_H 7.33 – 7.26 (m, 2H), 7.20 – 7.13 (m, 2H), 4.89 (t, *J* = 6.35 Hz, 1H), 4.23 – 4.14 (m, 2H), 3.95 – 3.84 (m, 2H), 2.97 (s, 3H), 2.64 (dd, *J* = 16.61, 6.35 Hz, 1H), 2.55 (dd, *J* = 16.64, 6.38 Hz, 1H), 2.30 (s, 3H), 0.88 (s, 9H), 0.11 (s, 3H), −0.07 (s, 3H).

^{13}C NMR (101 MHz, Acetone-d_6) δ_C 168.5, 168.4, 142.1, 137.7, 129.5, 126.9, 99.4, 74.3, 62.0, 62.0, 48.0, 32.2, 26.2, 21.1, 18.7, −4.6, −4.7.

^{11}B NMR (128 MHz, Acetone-d_6) δ_B 5.8.

IR (ATR, film): 3198, 2955, 2207, 1767, 1458, 1285, 1252, 1163, 1030, 988, 835, 777 cm^{-1}.

HRMS (ESI): Calculated for 452.2035 *m/z*, found 452.2029 *m/z* $[C_{22}H_{32}BNO_5Si+Na]^+$.

2-(4-Hydroxy-4-(*p*-tolyl)but-1-yn-1-yl)-6-methyl-1,3,6,2-dioxazaborocane-4,8-dione (**S30**)

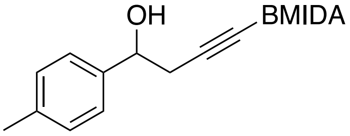

Prepared according **General Procedure D** from 2-(4-((*tert*-butyldimethylsilyl)oxy)-4-(*p*-tolyl)but-1-yn-1-yl)-6-methyl-1,3,6,2-dioxazaborocane-4,8-dione (85.9 mg, 200 µmol, 1.00 equiv.). Purified by precipitation from acetone-CH_2Cl_2:Et_2O-hexane to yield the desired product as a white solid (11.6 mg, 18%).

^{1}H NMR (400 MHz, Acetone-d_6) δ_H 7.33 (d, *J* = 8.03 Hz, 2H), 7.15 (ddd, *J* = 7.37, 1.66, 0.84 Hz, 2H), 4.83 (t, *J* = 6.40 Hz, 1H), 4.47 (s, 1H), 4.20 (dd, *J* = 17.02, 0.78 Hz, 2H), 3.94 (dd, *J* = 16.98, 9.44 Hz, 2H), 2.99 (s, 3H), 2.65 (dd, *J* = 7.94, 6.42 Hz, 2H), 2.31 (s, 3H).

^{13}C NMR (101 MHz, Acetone-d_6) δ_C 167.8, 167.7, 141.5, 136.6, 128.6, 126.1, 86.9, 71.8, 61.1, 61.1, 47.2, 30.2, 20.2.

^{11}B NMR (128 MHz, Acetone-d_6) δ_B 6.0.

IR (ATR, film): 1744, 1638, 1167, 1028, 891, 862, 820, 752, 679 cm^{-1}.

HRMS (ESI): Calculated for 338.1170 *m/z*, found 338.1176 *m/z* $[C_{16}H_{18}BNO_5+Na]^+$.

1-(3,5-Dimethoxyphenyl)but-3-yn-1-ol (**S31**)

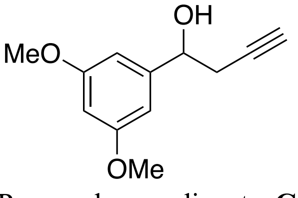

Prepared according to **General Procedure E** from 3,5-dimethoxybenzaldehyde (2.01 g, 12.1 mmol, 1.00 equiv.). Purified by flash column chromatography (silica, 0–20% EtOAc in hexane) to yield the desired product as a colorless oil (2.242 g, 90%).

^{1}H NMR (500 MHz, $CDCl_3$) δ_H 6.58 (dd, *J* = 2.34, 0.53 Hz, 2H), 6.42 (t, *J* = 2.29 Hz, 1H), 4.84 (dd, *J* = 7.15, 5.51 Hz, 1H), 3.82 (s, 6H), 2.66 (ddd, *J* = 5.92, 2.96, 2.12 Hz, 2H), 2.12 (t, *J* = 2.63 Hz, 1H).

^{13}C NMR (126 MHz, $CDCl_3$) δ_C 160.9, 145.0, 103.7, 99.9, 81.3, 72.4, 71.1, 55.4, 29.5.

Spectral data consistent with the literature.[18]

tert-Butyl((1-(3,5-dimethoxyphenyl)but-3-yn-1-yl)oxy)dimethylsilane (**S32**)

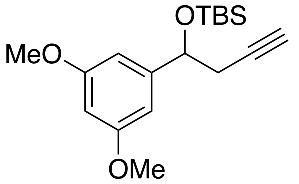

Prepared according to **General Procedure E** from 1-(3,5-dimethoxyphenyl)but-3-yn-1-ol (1.93 g, 9.37 mmol, 1.00 equiv.). Purified by flash column chromatography (silica, 0–5% Et_2O in hexane) to yield the desired compound as a pale-yellow oil (3.01 g, >99%).

^{1}H NMR (500 MHz, $CDCl_3$) δ_H 6.55 (d, *J* = 2.28 Hz, 2H), 6.38 (t, *J* = 2.34 Hz, 1H), 4.77 (dd, *J* = 7.28, 5.43 Hz, 1H), 3.81 (s, 6H), 2.58 (ddd, *J* = 16.66, 7.34, 2.65 Hz, 1H), 2.49 (ddd, *J* = 16.66, 5.43, 2.64 Hz, 1H), 2.00 (t, *J* = 2.64 Hz, 1H), 0.92 (s, 9H), 0.11 (s, 3H), −0.01 (s, 3H).

^{13}C NMR (126 MHz, $CDCl_3$) δ_C 160.5, 146.6, 103.7, 99.4, 83.8, 73.7, 70.0, 55.3, 31.0, 25.8, 14.2, −4.7, −4.9.

IR (ATR, film): 1744, 1638, 1167, 1028, 891, 862, 820, 752, 679 cm^{-1}.

HRMS (ESI): Calculated for 321.1881 *m/z*, found 321.1883 *m/z* $[C_{18}H_{28}O_3Si+H]^+$.

2-(4-((*tert*-Butyldimethylsilyl)oxy)-4-(3,5-dimethoxyphenyl)but-1-yn-1-yl)-6-methyl-1,3,6,2-dioxazaborocane-4,8-dione (**S33**)

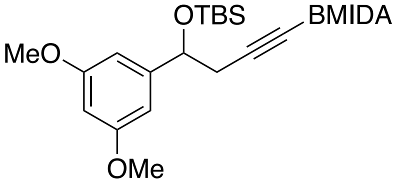

Prepared according to **General Procedure A** from *tert*-butyl((1-(3,5-dimethoxyphenyl)but-3-yn-1-yl)oxy)dimethylsilane (641 mg, 2.00 mmol, 1.00 equiv.). Purified by precipitation from hexane/Et_2O to afford the desired product as a white powder (367.3 mg, 39%).

^{1}H NMR (500 MHz, Acetone-*d*$_6$) δ_H 6.61 (d, *J* = 2.29 Hz, 2H), 6.38 (t, *J* = 2.35 Hz, 1H), 4.87 (t, *J* = 6.20 Hz, 1H), 4.21 (m, 2H), 3.94 (m, 2H), 3.78 (s, 6H), 3.03 (s, 3H), 2.63 (dd, *J* = 16.67, 6.41 Hz, 1H), 2.56 (dd, *J* = 16.67, 6.09 Hz, 1H), 0.91 (s, 9H), 0.13 (s, 3H), -0.02 (s, 3H).

^{13}C NMR (126 MHz, Acetone-*d*$_6$) δ_C 168.5, 168.4, 161.6, 147.6, 104.8, 99.8, 99.4, 74.3, 62.0, 62.0, 55.5, 48.1, 32.2, 26.2, 18.8, −4.6, −4.7.

^{11}B NMR (128 MHz, Acetone-*d*$_6$) δ_B 6.0.

IR (ATR, film): 2953, 2205, 1767, 1597, 1458, 1287, 1153, 1030, 835, 777 cm^{-1}.

HRMS (ESI): Calculated for 476.2270 *m/z*, found 476.2270 *m/z* $[C_{23}H_{34}BNO_7Si+H]^+$.

2-(4-(3,5-Dimethoxyphenyl)-4-hydroxybut-1-yn-1-yl)-6-methyl-1,3,6,2-dioxazaborocane-4,8-dione (**S34**)

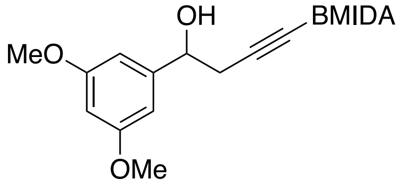

Prepared according **General Procedure D** from 2-(4-((*tert*-butyldimethylsilyl)oxy)-4-(3,5-dimethoxyphenyl)but-1-yn-1-yl)-6-methyl-1,3,6,2-dioxazaborocane-4,8-dione (95.1 mg, 200 µmol, 1.00 equiv.). Purified by flash column chromatography (silica, 0–100% Acetone in CH_2Cl_2) to yield the desired product as a white solid (56.9 mg, 79%).

^{1}H NMR (500 MHz, Acetone-d_6) δ_H 6.66 – 6.63 (m, 2H), 6.38 (t, J = 2.34 Hz, 1H), 4.80 (td, J = 6.22, 3.25 Hz, 1H), 4.58 (d, J = 4.41 Hz, 1H), 4.22 (dd, J = 16.93, 1.11 Hz, 2H), 3.98 (dd, J = 16.95, 7.48 Hz, 2H), 3.79 (s, 6H), 3.05 (s, 3H), 2.69 – 2.59 (m, 2H).

^{13}C NMR (126 MHz, Acetone-d_6) δ_C 167.7, 167.7, 160.8, 146.9, 103.9, 98.9, 98.6, 72.0, 61.1, 61.1, 54.7, 47.2, 30.2.

^{11}B NMR (96 MHz, Acetone-d_6) δ_B 6.1.

IR (ATR, film): 2209, 1763, 1707, 1597, 1458, 1290, 1157, 1028, 986, 858, 704 cm^{-1}.

HRMS (ESI): Calculated for 362.1406 *m/z*, found 362.1417 *m/z* [$C_{17}H_{20}BNO_7$+H]$^+$.

tert-Butyldimethyl((1-(thiophen-2-yl)but-3-yn-1-yl)oxy)silane (**S35**)

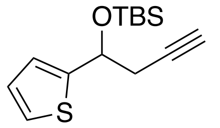

Prepared according to **General Procedure E** from thiophene-2-carbaldehyde (1.00 mL, 11.0 mmol, 1.00 equiv.). Modified work up procedure employed, no acid was added. Mixture was quenched with EtOAc (20 mL), filtered, and concentrated *in vacuo* to yield the crude product, which was purified by flash column chromatography (silica, 0–20% EtOAc in hexane) to yield the desired product with a contaminant. This was then subjected to **General Procedure F**. The crude product was purified by flash column chromatography (silica, 100% hexane) to yield the desired product as a colorless oil (804.7 mg, 30%).

^{1}H NMR (500 MHz, $CDCl_3$) δ_H 7.23 (dd, J = 4.95, 1.28 Hz, 1H), 6.99 (ddd, J = 3.46, 1.29, 0.74 Hz, 1H), 6.96 (dd, J = 4.97, 3.48 Hz, 1H), 5.11 (td, J = 6.44, 0.75 Hz, 1H), 2.71 (ddd, J = 16.57, 6.64, 2.64 Hz, 1H), 2.61 (ddd, J = 16.59, 6.18, 2.65 Hz, 1H), 2.03 (t, J = 2.65 Hz, 1H), 0.92 (s, 9H), 0.13 (s, 3H), 0.02 (s, 3H).

^{13}C NMR (126 MHz, $CDCl_3$) δ_C 148.3, 126.3, 124.2, 123.3, 81.1, 70.4, 70.0, 31.4, 25.7, 18.2, −4.9, −4.9.

Spectral data consistent with the literature.[19]

2-(4-((*tert*-Butyldimethylsilyl)oxy)-4-(thiophen-2-yl)but-1-yn-1-yl)-6-methyl-1,3,6,2-dioxazaborocane-4,8-dione (**S36**)

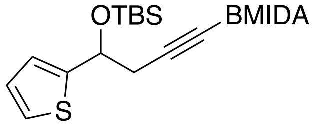

Prepared according to **General Procedure A** from *tert*-butyldimethyl((1-(thiophen-2-yl)but-3-yn-1-yl)oxy)silane (533 mg, 539 µL, 1.00 equiv., 2.00 mmol). Purified by precipitation from hexane/Et_2O to afford the desired product as a white powder (309.5 mg, 37%).

^{1}H NMR (500 MHz, Acetone-d_6) δ_H 7.35 (dd, J = 5.08, 1.23 Hz, 1H), 7.07 (dt, J = 3.53, 1.18 Hz, 1H), 6.96 (dd, J = 5.08, 3.47 Hz, 1H), 5.22 (t, J = 6.18 Hz, 1H), 4.21 (m, 2H), 3.93 (m, 2H), 3.03 (s, 3H), 2.74 (dd, J = 16.67, 5.98 Hz, 1H), 2.65 (dd, J = 16.67, 6.52 Hz, 1H), 0.90 (s, 9H), 0.15 (s, 3H), 0.01 (s, 3H).

^{13}C NMR (126 MHz, Acetone-d_6) δ_C 168.5, 168.4, 149.3, 127.1, 125.2, 124.6, 98.6, 70.7, 62.0, 62.0, 48.1, 32.7, 26.1, 18.7, −4.7, −4.8.

^{11}B NMR (96 MHz, Acetone-d_6) δ_B 6.0.

IR (ATR, film): 2955, 2930, 2207, 1749, 1252, 1165, 1030, 984, 837, 777, 700 cm^{-1}.

HRMS (ESI): Calculated for 444.1443 *m/z*, found 444.1435 *m/z* [$C_{19}H_{28}BNO_5SSi$+Na]$^+$.

2-(4-Hydroxy-4-(thiophen-2-yl)but-1-yn-1-yl)-6-methyl-1,3,6,2-dioxazaborocane-4,8-dione (**S37**)

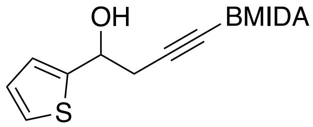

Prepared according **General Procedure D** from 2-(4-((*tert*-butyldimethylsilyl)oxy)-4-(thiophen-2-yl)but-1-yn-1-yl)-6-methyl-1,3,6,2-dioxazaborocane-4,8-dione (84.3 mg, 200 µmol, 1.00 equiv.). However, the desired product was very unstable and thus was used directly in **General Procedure C**.

1-(4-Bromophenyl)but-3-yn-1-ol (**S38**)

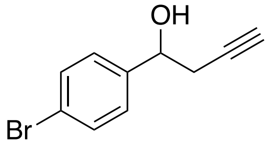

Prepared according to **General Procedure E** from 4-bromobenzaldehyde (925 mg, 5.00 mmol, 1.00 equiv.). Purified by flash column chromatography (silica, 0–10% EtOAc in hexane) to yield the desired product as a pale-yellow oil (403 mg, 36%).

^{1}H NMR (400 MHz, $CDCl_3$) δ_H 7.58 – 7.48 (m, 2H), 7.34 – 7.26 (m, 2H), 4.86 (td, J = 6.26, 2.79 Hz, 1H), 2.70 – 2.55 (m, 2H), 2.10 (t, J = 2.64 Hz, 1H).

^{13}C NMR (101 MHz, $CDCl_3$) δ_C 141.4, 131.6, 127.5, 121.9, 80.2, 71.7, 71.4, 29.5.

Spectral data consistent with the literature.[18]

((1-(4-Bromophenyl)but-3-yn-1-yl)oxy)(*tert*-butyl)dimethylsilane (**S39**)

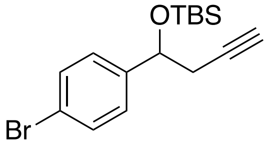

Prepared according to **General Procedure F** from 1-(4-bromophenyl)but-3-yn-1-ol (300 mg, 1.33 mmol, 1.00 equiv.). Purified by flash column chromatography (silica, 0–5% Et_2O in hexane) to yield the desired product as a colorless oil (443 mg, 1.31 mmol, 98%).

^{1}H NMR (500 MHz $CDCl_3$) δ_H 7.52 – 7.44 (m, 2H), 7.28 – 7.24 (m, 2H), 4.79 (t, J = 6.41 Hz, 1H), 2.59 (ddd, J = 16.58, 6.63, 2.63 Hz, 1H), 2.47 (ddd, J = 16.60, 6.17, 2.63 Hz, 1H), 1.98 (t, J = 2.64 Hz, 1H), 0.91 (s, 9H), 0.10 (s, 3H), −0.05 (s, 3H).

^{13}C NMR (126 MHz, $CDCl_3$) δ_C 143.0, 131.2, 127.7, 121.3, 73.1, 70.3, 30.9, 25.8, 18.2, −4.8, −4.9.

IR (ATR, film): 2928, 1252, 1107, 1086, 1071, 1011, 928, 858, 835, 775, 629, 536 cm^{-1}.

HRMS (ESI): Calculated for 361.0594 *m/z*, found 361.0597 *m/z* $[C_{16}H_{23}BrOSi+Na]^+$.

2-(4-(4-Bromophenyl)-4-((*tert*-butyldimethylsilyl)oxy)but-1-yn-1-yl)-6-methyl-1,3,6,2-dioxazaborocane-4,8-dione (**S40**)

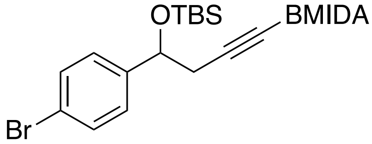

Prepared according to **General Procedure A** from ((1-(4-bromophenyl)but-3-yn-1-yl)oxy)(*tert*-butyl)dimethylsilane (450 mg, 1.00 equiv., 1.33 mmol). Purified by precipitation from hexane/Et_2O to afford the desired product as a white powder (380.8 mg, 58%).

^{1}H NMR (400 MHz, Acetone-d_6) δ_H 7.54 (d, J = 8.33 Hz, 2H), 7.42 (d, J = 8.38 Hz, 2H), 4.96 (t, J = 6.21 Hz, 1H), 4.22 (m, 2H), 3.95 (m, 2H), 3.04 (s, 3H), 2.68 (dd, J = 16.66, 6.12 Hz, 1H), 2.59 (dd, J = 16.71, 6.36 Hz, 1H), 0.91 (s, 9H), 0.14 (s, 3H), −0.03 (s, 3H).

^{13}C NMR (101 MHz, Acetone-d_6) δ_C 168.4, 144.4, 131.9, 129.1, 121.5, 98.8, 73.6, 62.0, 48.1, 32.0, 26.1, 18.7, −4.7, −4.7.

^{11}B NMR (128 MHz, Acetone-d_6) δ_B 6.3.

IR (ATR, film): 2955, 2205, 1767, 1281, 1252, 1163, 1070, 1030, 1009, 986, 835, 777 cm^{-1}.

HRMS (ESI): Calculated for 516.0984 *m/z*, found 516.0990 *m/z* $[C_{21}H_{29}BBrNO_5Si+Na]^+$.

2-(4-(4-Bromophenyl)-4-hydroxybut-1-yn-1-yl)-6-methyl-1,3,6,2-dioxazaborocane-4,8-dione (**S41**)

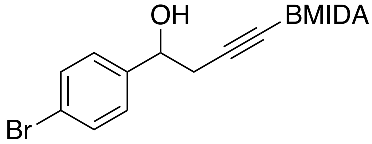

Prepared according **General Procedure D** from 2-(4-(4-bromophenyl)-4-((*tert*-butyldimethylsilyl)oxy)but-1-yn-1-yl)-6-methyl-1,3,6,2-dioxazaborocane-4,8-dione (98.9 mg, 200 μmol, 1.00 equiv.). Purified by flash column chromatography (silica, 0–10% MeCN in CH_2Cl_2) to yield the desired product as a white solid (66.5 mg, 88%).

^{1}H NMR (400 MHz, Acetone-d_6) δ_H 7.56 – 7.47 (m, 2H), 7.46 – 7.36 (m, 2H), 4.88 (t, J = 6.32 Hz, 1H), 4.22 (m, 2H), 3.98 (m, 2H), 3.05 (s, 3H), 2.73 – 2.57 (m, 2H).

^{13}C NMR (101 MHz, Acetone-d_6) δ_C 167.7, 167.7, 143.8, 131.0, 128.2, 120.5, 98.3, 71.2, 61.2, 61.2, 47.3, 30.0.

^{11}B NMR (128 MHz, Acetone-d_6) δ_B 6.0.

IR (ATR, film): 1744, 1289, 1163, 1146, 1028, 1009, 988, 963, 860, 527, 403 cm^{-1}.

HRMS (ESI): Calculated for 402.0119 *m/z*, found 402.0112 *m/z* $[C_{15}H_{15}BrBNO_5+Na]^+$.

1-(4-(Trifluoromethyl)phenyl)but-3-yn-1-ol (**S42**)

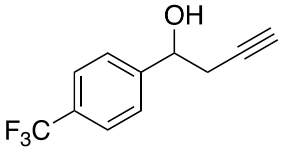

Prepared according to **General Procedure E** from 4-trifluoromethylbenzaldehyde (680 μL, 4.98 mmol, 1.00 equiv.). Purified by flash column chromatography (silica, 0–10% EtOAc in hexane) to yield the desired product as a pale-yellow oil (301 mg, 28%).

^{1}H NMR (500 MHz, $CDCl_3$) δ_H 7.68 – 7.63 (m, 2H), 7.55 (d, J = 8.07 Hz, 2H), 5.01 – 4.94 (m, 1H), 2.74 – 2.62 (m, 2H), 2.13 (t, J = 2.63 Hz, 1H).

^{13}C NMR (126 MHz, $CDCl_3$) δ_C 146.2, 130.2 (q, $^2J_{CF}$ = 32.4 Hz), 126.1, 125.5 (q, $^3J_{CF}$ = 3.8 Hz), 124.1 (q, $^1J_{CF}$ = 272.0 Hz), 79.9, 71.7, 71.6, 29.5

^{19}F NMR (471 MHz, $CDCl_3$) δ_F −62.5.

Spectral data consistent with the literature.[20]

tert-Butyldimethyl((1-(4-(trifluoromethyl)phenyl)but-3-yn-1-yl)oxy)silane (**S43**)

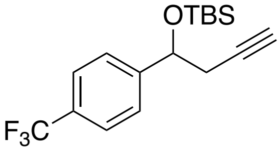

Prepared according to **General Procedure F** from 1-(4-(trifluoromethyl)phenyl)but-3-yn-1-ol (200 mg, 934 µmol, 1.00 equiv.). Purified by flash column chromatography (silica, 0–5% Et_2O in hexane) to yield the desired product as a colorless oil (295 mg, 96%).

^{1}H NMR (500 MHz $CDCl_3$) δ_H 7.61 (d, J = 8.09 Hz, 2H), 7.51 (d, J = 8.01 Hz, 2H), 4.89 (t, J = 6.38 Hz, 1H), 2.62 (ddd, J = 16.59, 6.54, 2.67 Hz, 1H), 2.51 (ddd, J = 16.64, 6.23, 2.66 Hz, 1H), 2.00 (t, J = 2.66 Hz, 1H), 0.92 (s, 9H), 0.12 (s, 3H), −0.03 (s, 3H).

^{13}C NMR (126 MHz, $CDCl_3$) δ_C 147.8, 129.7 (q, $^2J_{CF}$ = 32.3 Hz), 126.2, 124.23 (q, $^1J_{CF}$ = 275.5 Hz), 125.1 (q, $^3J_{CF}$ = 3.6 Hz), 80.8, 73.1, 70.6, 30.8, 25.7, 18.2, −4.8, −4.9.

^{19}F{^{1}H} (470 MHz, $CDCl_3$) δ_F −62.4.

IR (ATR, film): 1323, 1258, 1163, 1125, 1094, 1067, 1017, 1007, 932, 860, 835, 810, 777, 669, 606 cm^{-1}.

HRMS (ESI): Calculated for 351.1363 *m/z*, found 351.1364 *m/z* $[C_{17}H_{23}F_3OSi+Na]^+$.

2-(4-((*tert*-Butyldimethylsilyl)oxy)-4-(4-(trifluoromethyl)phenyl)but-1-yn-1-yl)-6-methyl-1,3,6,2-dioxazaborocane-4,8-dione (**S44**)

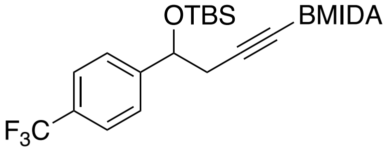

Prepared according to **General Procedure A** from *tert*-butyldimethyl((1-(4-(trifluoromethyl)phenyl)but-3-yn-1-yl)oxy)silane (391 mg, 1.19 mmol, 1.00 equiv.). Purified by column chromatography (silica, 0–5% MeCN in CH_2Cl_2) to afford the desired product as a colorless solid (304.8 mg, 53%).

^{1}H NMR (400 MHz, Acetone-d_6) δ_H 7.71 – 7.67 (m, 4H), 5.07 (t, J = 6.16 Hz, 1H), 4.24 – 4.16 (m, 2H), 3.97 – 3.89 (m, 2H), 3.01 (s, 3H), 2.71 (dd, J = 16.74, 6.05 Hz, 1H), 2.63 (dd, J = 16.74, 6.34 Hz, 1H), 0.91 (s, 9H), 0.15 (s, 3H), −0.03 (s, 3H).

^{13}C NMR (101 MHz, Acetone-d_6) δ_C 168.4, 168.4, 149.6, 129.9 (q, $^2J_{CF}$ = 32.0 Hz), 127.8, 125.8 (q, $^3J_{CF}$ = 3.8 Hz), 125.4 (q, $^1J_{CF}$ = 271.2 Hz), 98.5, 73.6, 62.1, 62.1, 48.1, 32.0, 26.2, 18.7, −4.7, 4.7.

^{11}B NMR (96 MHz, Acetone-d_6) δ_B 6.3.

^{19}F{^{1}H} NMR (376 MHz, $CDCl_3$) δ_F −57.6.

IR (ATR, film): 2955, 2208, 1771, 1749, 1325, 1163, 1034, 837, 779 cm^{-1}.

HRMS (ESI): Calculated for 506.1752 *m/z*, found 506.1743 *m/z* $[C_{22}H_{29}BF_3NO_5Si+Na]^+$.

tert-Butyldimethyl(1-(prop-2-yn-1-yl)cyclobutoxy)silane (**S45**)

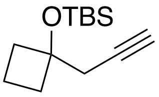

A flame-dried flask was charged with zinc (3.28 g, 50.2 mmol, 5.00 equiv.) under N_2 followed by (in order) dry THF (50.0 mL), 1,2-dibromoethane (950 µL, 11.0 mmol, 1.10 equiv.), and cyclobutanone (750 µL, 10.0 mmol, 1.00 equiv.) then cooled to 0 ºC. 3-Bromoprop-1-yne (80 wt% in PhMe, 1.90 mL, 17.1 mmol, 1.70 equiv.) was added dropwise and the resulting solution was allowed to stir for 16 h at room temperature before unsealing and quenching with dilute aq. HCl (2 M, ~100 mL) and filtering the solution. The mixture was extracted with CH_2Cl_2 (3 × 100 mL) and the combined organic layers were dried over Na_2SO_4, filtered, and concentrated *in vacuo* to yield the crude product. To this, DMF (50.0 mL) was added followed by 1*H*-imidazole (2.04 g, 30.0 mmol, 3.00 equiv.) and TBSCl (3.01 g, 20.0 mmol, 2.00 equiv.) then the mixture was heated to 70 ºC to stir for 16 h. The mixture was allowed to cool to room temperature then quenched with the addition of water (~100 mL). The mixture was extracted with CH_2Cl_2 (3 ×100 mL) and the combined organic layers

were washed with 10% aq. LiCl (~50 mL) and then washed with Na_2SO_4, filtered, and concentrated *in vacuo* to yield the crude product, which was purified by flash column chromatography (silica, hexane) to yield the desired product as a colorless oil (739 mg, 33%).

^{1}H NMR (500 MHz, $CDCl_3$) δ_H 2.49 (d, J = 2.58 Hz, 2H), 2.20 – 2.13 (m, 4H), 1.97 (t, J = 2.63 Hz, 1H), 1.79 – 1.67 (m, 1H), 1.57 – 1.47 (m, 1H), 0.91 (s, 9H), 0.13 (s, 6H).

^{13}C NMR (126 MHz, $CDCl_3$) δ_C 82.0, 75.2, 69.3, 36.2, 30.9, 25.8, 18.0, 12.4, −2.7.

IR (ATR, film): 1740, 1736, 1302, 1169, 1057, 1042, 1028, 993, 949, 874, 702 cm^{-1}.

HRMS (ESI): Calculated for 247.1489 *m/z*, found 247.1496 *m/z* $[C_{13}H_{24}OSi+Na]^+$.

2-(3-(1-((*tert*-Butyldimethylsilyl)oxy)cyclobutyl)prop-1-yn-1-yl)-6-methyl-1,3,6,2-dioxazaborocane-4,8-dione (**S46**)

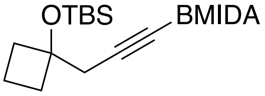

Prepared according to **General Procedure A** from *tert*-butyldimethyl(1-(prop-2-yn-1-yl)cyclobutoxy)silane (561 mg, 625 µL, 2.50 mmol, 1.00 equiv.). Purified by precipitation from hexane/Et_2O to afford the desired product as a white powder (367.3 mg, 39%).

^{1}H NMR (500 MHz, Acetone-*d*$_6$) δ_H 4.25 (d, J = 16.88 Hz, 2H), 4.02 (d, J = 16.88 Hz, 2H), 3.22 (s, 3H), 2.56 (s, 2H), 2.18 – 2.10 (m, 4H), 1.75 – 1.66 (m, 1H), 1.57 (m, 1H), 0.89 (s, 9H), 0.14 (s, 6H).

^{13}C NMR (126 MHz, Acetone-*d*$_6$) δ_C 168.5, 100.0, 76.3, 62.1, 48.4, 36.9, 32.3, 26.2, 18.5, 12.9, −2.5.

^{11}B NMR (128 MHz, Acetone-*d*$_6$) δ_B 6.1.

IR (ATR, film): 2955, 2934, 2297, 1765, 1283, 1252, 1161, 1032, 991, 835, 773 cm^{-1}.

HRMS (ESI): Calculated for 380.2059 *m/z*, found 380.2043 *m/z* $[C_{18}H_{30}BN_1O_5Si+H]^+$.

2-(3-(1-Hydroxycyclobutyl)prop-1-yn-1-yl)-6-methyl-1,3,6,2-dioxazaborocane-4,8-dione (**S47**)

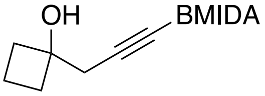

Prepared according to **General Procedure D** from 2-(3-(1-((*tert*-butyldimethylsilyl)oxy)cyclobutyl)prop-1-yn-1-yl)-6-methyl-1,3,6,2-dioxazaborocane-4,8-dione (75.9 mg, 1.33 mmol, 1.00 equiv.). Purified by flash column chromatography (silica, 0–100% acetone in CH_2Cl_2) to yield the desired product as a white solid (28.3 mg, 53%).

^{1}H NMR (700 MHz, Acetone-*d*$_6$) δ_H 4.23 (d, J = 16.97 Hz, 2H), 4.05 (d, J = 16.92 Hz, 2H), 3.21 (s, 3H), 2.50 (s, 2H), 2.12 (m, 2H), 2.07 – 2.00 (m, 2H), 1.69 (m, 1H), 1.57 (m, 1H).

^{13}C NMR (176 MHz, Acetone-*d*$_6$) δ_C 167.9, 99.0, 73.4, 61.3, 47.5, 35.1, 30.8, 11.5.

^{11}B NMR (96 MHz, Acetone-*d*$_6$) δ_B 6.1.

IR (ATR, film): 2205, 1744, 1707, 1452, 1339, 1283, 1165, 1026, 984, 963, 889, 860, 704 cm^{-1}.

HRMS (ESI): Calculated for 266.1194 *m/z*, found 266.1201 *m/z* $[C_{12}H_{16}BNO_5+H]^+$.

2-(4-((*tert*-Butyldimethylsilyl)oxy)but-1-yn-1-yl)-6-methyl-1,3,6,2-dioxazaborocane-4,8-dione (**S48**)

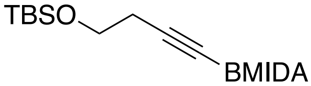

Prepared as previously reported.[21]

2-(4-Hydroxybut-1-yn-1-yl)-6-methyl-1,3,6,2-dioxazaborocane-4,8-dione (**S49**)

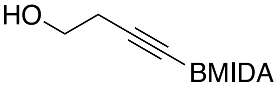

Prepared according to **General Procedure D** from 2-(4-((*tert*-butyldimethylsilyl)oxy)but-1-yn-1-yl)-6-methyl-1,3,6,2-dioxazaborocane-4,8-dione (2.00 g, 5.90 mmol, 1.00 equiv.). Purified by flash column chromatography (silica, 20–50% MeCN in CH_2Cl_2) to give a solid, which was further purified by trituration with Et_2O and EtOAc to yield the desired product as a white solid (665 mg, 50%).

^{1}H NMR (400 MHz, Acetone-d_6) δ_H 4.25 (d, J = 16.90 Hz, 2H), 4.07 (d, J = 16.83 Hz, 2H), 3.66 (t, J = 6.85 Hz, 2H), 2.43 (t, J = 6.83 Hz, 2H).

^{13}C NMR (101 MHz, Acetone) δ_c 167.5, 100.0, 61.3, 60.5, 47.4, 23.5.

^{11}B NMR (128 MHz, Acetone-d_6) δ_B 6.1.

IR (ATR, film): 3447, 2988, 2209, 1740, 1630, 1304, 1171, 1057, 1044, 1028, 995, 949, 874, 702 cm^{-1}.

HRMS (ESI): Calculated for 226.0881 *m*/*z*, found 226.0875 *m*/*z* $[C_9H_{12}BNO_5+H]^+$.

tert-Butyl(pent-4-yn-1-yloxy)diphenylsilane (**S50**)

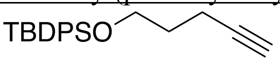

A flask was charged with 1*H*-imidazole (1.10 g, 16.2 mmol, 1.50 equiv.), TBDPSCl (4.20 mL, 16.2 mmol, 1.50 equiv.), and CH_2Cl_2 (50.0 mL). 4-Pentyn-1-ol (1.00 mL, 10.8 mmol, 1.00 equiv.) was added and the solution was stirred for 5 h at room temperature before quenching with water (~100 mL). The biphasic system was extracted with CH_2Cl_2 (3 × 50 mL) and the combined organic layers were dried over Na_2SO_4, filtered, and concentrated *in vacuo* to yield the crude product, which was purified by flash column chromatography (silica, hexane) to yield the desired product as a colorless oil (3.12 g, 90%).

^{1}H NMR (400 MHz, $CDCl_3$) δ_H 7.73 – 7.68 (m, 4H), 7.50 – 7.37 (m, 6H), 3.78 (t, J = 5.98 Hz, 2H), 2.45 – 2.36 (m, 2H), 1.95 (t, J = 2.66 Hz, 1H), 1.86 – 1.76 (m, 2H), 1.08 (s, 9H).

^{13}C NMR (101 MHz, $CDCl_3$) δ_C 135.6, 133.8, 129.6, 127.7, 84.3, 68.3, 62.3, 31.4, 26.9, 19.3, 15.0.

Spectral data consistent with the literature.[22]

2-(5-((*tert*-Butyldiphenylsilyl)oxy)pent-1-yn-1-yl)-6-methyl-1,3,6,2-dioxazaborocane-4,8-dione (**S51**)

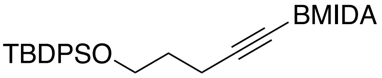

Prepared according to **General Procedure A** from *tert*-butyl(pent-4-yn-1-yloxy)diphenylsilane (1.29 g, 4.00 mmol, 1.00 equiv.). The product was purified by precipitation from hexane/Et_2O to afford the desired product as a white powder (756 mg, 40%).

^{1}H NMR (400 MHz, Acetone-d_6) δ 7.70 (dt, J = 4.70, 2.31 Hz, 4H), 7.48 – 7.39 (m, 6H), 4.23 (d, J = 16.94 Hz, 2H), 4.01 (d, J = 16.85 Hz, 2H), 3.80 (t, J = 6.20 Hz, 2H), 3.11 (s, 3H), 2.41 (t, J = 7.10 Hz, 2H), 1.79 (h, J = 6.97 Hz, 2H), 1.04 (s, 9H).

^{13}C NMR (101 MHz, Acetone-d_6) δ_C 168.6, 136.2, 134.4, 130.6, 128.6, 101.9, 63.2, 62.1, 48.2, 32.3, 27.2, 19.7, 16.3.

^{11}B NMR (96 MHz, Acetone-d_6) δ 6.3.

IR (ATR, film): 3213, 2955, 2203, 1767, 1462, 1285, 1105, 1032, 988, 702, 503 cm^{-1}.

HRMS (ESI): Calculated for 476.2216 *m*/*z*, found 476.2217 *m*/*z* $[C_{26}H_3BNO_5Si+Na]^+$.

2-(5-Hydroxypent-1-yn-1-yl)-6-methyl-1,3,6,2-dioxazaborocane-4,8-dione (**S52**)

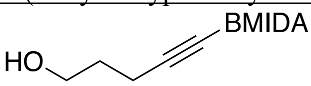

Prepared according **General Procedure D** from 2-(5-((*tert*-butyldiphenylsilyl)oxy)pent-1-yn-1-yl)-6-methyl-1,3,6,2-dioxazaborocane-4,8-dione (95.5 mg, 200 µmol, 1.00 equiv.). Purified by precipitation from acetone-CH_2Cl_2:Et_2O-hexane to yield the desired product as a white solid (23.2 mg, 49%, N.B. this compound readily degrades in solution).

^{1}H NMR (400 MHz, Acetone-d_6) δ_H 4.39 (s, 1H), 4.24 (d, J = 16.93 Hz, 2H), 4.07 (d, J = 16.92 Hz, 2H), 3.64 (t, J = 6.20 Hz, 2H), 3.21 (s, 3H), 2.33 (t, J = 7.12 Hz, 2H), 1.71 (tt, J = 7.13, 6.17 Hz, 2H).

^{13}C NMR (101 MHz, Acetone-d_6) δ_C 167.8, 79.2, 61.3, 60.2, 47.4, 31.7, 15.5.

^{11}B NMR (128 MHz, Acetone-d_6) δ_B 6.0.

IR (ATR, film): 3196, 1744, 1634, 1456, 1418, 1254, 1167, 1030 cm^{-1}.

HRMS (ESI): Calculated for 240.1038 *m/z*, found 240.1028 *m/z* $[C_{10}H_{14}BNO_5+H]^+$.

(But-3-yn-2-yloxy)(*tert*-butyl)dimethylsilane (**S53**)

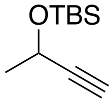

Prepared according to **General Procedure F** from 3-butyn-2-ol (1.00 mL, 12.8 mmol, 1.00 equiv.). Used in subsequent steps without further purification due to volatility.

2-(3-((*tert*-Butyldimethylsilyl)oxy)but-1-yn-1-yl)-6-methyl-1,3,6,2-dioxazaborocane-4,8-dione (**S54**)

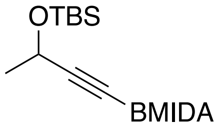

Prepared according to **General Procedure A** from (but-3-yn-2-yloxy)(*tert*-butyl)dimethylsilane (369 mg, 2.00 mmol, 1.00 equiv.). The product was purified by precipitation from hexane/Et_2O to afford the desired product as a white powder (109.5 mg, 16%).

^{1}H NMR (500 MHz, Acetone-d_6) δ_H 4.61 (q, J = 6.46 Hz, 1H), 4.28 (d, J = 16.88 Hz, 2H), 4.08 (d, J = 16.88 Hz, 2H), 3.23 (s, 3H), 1.37 (d, J = 6.52 Hz, 3H), 0.89 (s, 9H), 0.14 (s, 3H), 0.12 (s, 3H).

^{13}C NMR (126 MHz, Acetone-d_6) δ_C 168.4, 168.4, 104.1, 62.3, 62.3, 59.9, 48.4, 26.1, 25.8, 18.7, −4.4, −4.8.

^{11}B NMR (96 MHz Acetone-d_6) δ_B 11.6.

IR (ATR, film): 2930, 1757, 1339, 1252, 1163, 1022, 837, 777 cm^{-1}.

HRMS (ESI): Calculated for 362.1566 *m/z*, found 362.1562 *m/z* $[C_{15}H_{26}BNO_5Si+Na]^+$.

tert-Butyldimethyl((1-phenylprop-2-yn-1-yl)oxy)silane (**S55**)

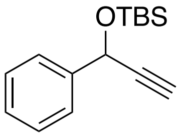

Prepared according to **General Procedure F** from 1-phenyl-2-propyn-1-ol (730 µL, 5.00 mmol, 1.00 equiv.). Purified by flash column chromatography (silica, hexane) to yield the desired product as a colorless oil (726 mg, 59%).

^{1}H NMR (400 MHz, $CDCl_3$) δ_H 7.56 – 7.50 (m, 2H), 7.38 (tt, *J* = 6.63, 0.96 Hz, 2H), 7.34 – 7.29 (m, 1H), 5.52 (d, *J* = 2.21 Hz, 1H), 2.58 (d, *J* = 2.24 Hz, 1H), 0.97 (s, 9H), 0.21 (s, 3H), 0.17 (s, 3H).

^{13}C NMR (126 MHz, $CDCl_3$) δ_C 141.3, 128.4, 127.8, 126.0, 84.8, 73.7, 64.6, 25.8, 18.3, −4.6, −4.9.

Spectral data consistent with the literature.[23]

2-(3-((*tert*-Butyldimethylsilyl)oxy)-3-phenylprop-1-yn-1-yl)-6-methyl-1,3,6,2-dioxazaborocane-4,8-dione **(S56)**

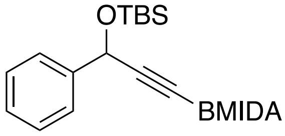

Prepared according to **General Procedure A** from *tert*-butyldimethyl((1-phenylprop-2-yn-1-yl)oxy)silane (493 mg, 2.00 mmol, 1.00 equiv.). The product was purified by column chromatography (silica, 0 to 5% MeCN in CH_2Cl_2) to afford the desired product as a white powder (128 mg, 16%).

^{1}H NMR (400 MHz, Acetone-d_6) δ_H 7.53 (d, *J* = 7.44 Hz, 2H), 7.36 (t, *J* = 7.44 Hz, 2H), 7.32 – 7.26 (m, 1H), 5.63 (s, 1H), 4.29 (d, *J* = 16.94 Hz, 2H), 4.08 (d, *J* = 16.94 Hz, 2H), 3.21 (s, 3H), 0.93 (s, 9H), 0.21 (s, 3H), 0.16 (s, 3H).

^{13}C NMR (101 MHz, Acetone-d_6) δ_C 168.4, 168.4, 142.7, 129.1, 128.5, 127.1, 102.0, 65.8, 62.3, 62.3, 48.5, 26.1, 18.8, −4.3, −4.7.

^{11}B NMR (96 MHz, Acetone-d_6) δ_B 6.3.

IR (ATR, film): 3200, 2934, 1767, 1462, 1427, 1283, 1103, 1032, 986, 700, 611, 498 cm^{-1}.

HRMS (ESI): Calculated for 424.1722 *m*/*z*, found 424.1720 *m*/*z* $[C_{20}H_{28}BNO_5Si+Na]^+$.

tert-Butyl((*trans*-2-ethynylcyclohexyl)oxy)dimethylsilane (**S57**)

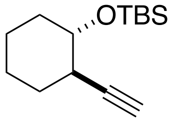

A flame-dried flask was charged with ethynyltrimethylsilane (4.30 mL, 30.0 mmol, 1.50 equiv.) and THF (25.0 mL) then cooled to −78 ºC. *n*-Butyllithium (1.93 g, 17.0 mL, 1.77 M in hexane, 30.1 mmol, 1.50 equiv.) was added dropwise and the resulting solution was stirred for 15 mins before BF_3•OEt_2 (4.54 g, 4.06 mL, 32.0 mmol, 1.60 equiv.) was added. This solution was also stirred for 15 mins at −78 ºC before 7-oxabicyclo[4.1.0]heptane (2.00 mL, 20.0 mmol, 1.00 equiv.) was added. This solution was stirred for 30 mins at −78 ºC then allowed to warm to room temperature to stir for 4 h. The resulting solution was quenched with the addition of sat. aq. NH_4Cl (~100 mL) and the mixture was then extracted CH_2Cl_2. (3 × 50 mL). The combined organic layers were dried over Na_2SO_4, filtered, and concentrated *in vacuo* to yield the crude product, which was purified by filtration through a pad of silica gel eluting with 0–10% EtOAc in hexane, to yield crude *trans*-2-((trimethylsilyl)ethynyl)cyclohexan-1-ol, which was used directly in the next step.
A flask was charged with *trans*-2-((trimethylsilyl)ethynyl)cyclohexan-1-ol (3.93 g, 20.0 mmol, 1.00 equiv., 100% yield assumed) and MeOH (100 mL). K_2CO_3 (5.53 g, 40.0 mmol, 2.00 equiv.) was added and the mixture was stirred for 16 h at room temperature. Water (200 mL) was then added and the mixture was extracted with CH_2Cl_2 (3 × 100 mL). The combined organic layers were dried over Na_2SO_4, filtered, and concentrated *in vacuo* to yield crude *trans*-2-(ethynyl)cyclohexan-1-ol, which was used directly in the next step.
trans-2-Ethynylcyclohexan-1-ol (2.48 g, 20.0 mmol, 1.00 equiv., 100% yield assumed) was added to a flask along with CH_2Cl_2 (40 mL). 1*H*-imidazole (4.08 g, 60.0 mmol, 3.00 equiv.) and TBSCl (7.54 g, 50.0 mmol, 2.50 equiv.) were added in one portion and the resulting solution was stirred at room temperature for 4 h before adding water (~100 mL). The biphasic system was extracted with CH_2Cl_2 (3 × 50 mL) and the combined organic layers were dried over Na_2SO_4, filtered, and concentrated *in vacuo* to yield the crude product, which was purified by flash column chromatography to yield the desired product as a pale-yellow oil (989.6 mg, 21%).

^{1}H NMR (500 MHz, $CDCl_3$) δ_H 3.63 (td, J = 7.85, 3.82 Hz, 1H), 2.34 (dddd, J = 9.56, 7.52, 3.95, 2.44 Hz, 1H), 2.07 (d, J = 2.37 Hz, 1H), 2.05 – 1.95 (m, 1H), 1.95 – 1.86 (m, 1H), 1.76 – 1.68 (m, 1H), 1.65 (dddd, J = 9.28, 8.29, 5.05, 2.40 Hz, 1H), 1.50 – 1.40 (m, 1H), 1.39 – 1.21 (m, 2H), 0.92 (s, 9H), 0.12 (s, 3H), 0.10 (s, 3H).

^{13}C NMR (126 MHz, $CDCl_3$) δ_C 87.3, 72.8, 69.3, 37.3, 33.8, 29.8, 25.9, 23.9, 23.1, 18.2, −4.5, −4.6.

Spectral data consistent with the literature.[24]

2-((*trans*-2-((*tert*-Butyldimethylsilyl)oxy)cyclohexyl)ethynyl)-6-methyl-1,3,6,2-dioxazaborocane-4,8-dione (**S58**)

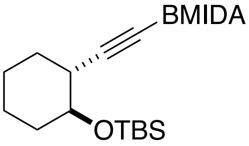

Prepared according to **General Procedure A** from *tert*-butyl((*trans*-2-ethynylcyclohexyl)oxy)dimethylsilane (353 mg, 1.48 mmol, 1.00 equiv.). Purified by precipitation from hexane/Et_2O to yield the desired product as a white powder (167.0 mg, 29%).

^{1}H NMR (500 MHz, Acetone-d_6) δ_H 4.23 (m, 2H), 4.06 – 4.00 (m, 2H), 3.74 – 3.68 (m, 1H), 3.20 (s, 3H), 2.37 (td, J = 8.33, 4.01 Hz, 1H), 1.98 – 1.86 (m, 2H), 1.72 – 1.57 (m, 2H), 1.44 (m, 1H), 1.32 – 1.23 (m, 3H), 0.90 (s, 9H), 0.12 (s, 3H), 0.09 (s, 3H).

^{13}C NMR (126 MHz, Acetone-d_6) δ_C 168.6, 168.6, 104.7, 73.1, 62.1, 62.1, 48.3, 38.5, 34.1, 30.2, 26.2, 24.3, 23.3, 18.6, −4.4, −4.4.

^{11}B NMR (96 MHz, Acetone-d_6) δ_B 6.3.

IR (ATR, film): 2932, 2197, 1771, 1506, 1458, 1169, 1101, 1030 cm^{-1}.

HRMS (ESI): Calculated for 394.2216 *m*/*z*, found 394.2197 *m*/*z* $[C_{19}H_{32}BNO_5Si+H]^+$.

1-(Furan-2-yl)but-3-yn-1-ol (**S59**)

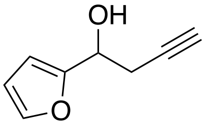

Prepared according to **General Procedure E** from furan-2-carbaldehyde (1.00 mL, 12.1 mmol, 1.00 equiv.). Modified work up procedure employed; no acid added. The mixture was quenched with EtOAc (~100 mL), filtered, and concentrated *in vacuo*. Purified by flash column chromatography (silica, 0–20% EtOAc in hexane) to yield the desired product as a yellow oil (310 mg, 19%).

^{1}H NMR (500 MHz, $CDCl_3$) δ_H 7.41 (dd, J = 1.62, 1.06 Hz, 1H), 6.38 – 6.35 (m, 2H), 4.90 (t, J = 6.25 Hz, 1H), 2.80 (dd, J = 6.28, 2.65 Hz, 2H), 2.10 (t, J = 2.65 Hz, 1H).

^{13}C NMR (126 MHz, $CDCl_3$) δ_C 154.6, 142.3, 110.3, 106.7, 79.9, 71.2, 66.1, 26.1.

Spectral data consistent with the literature.[18]

tert-Butyl((1-(furan-2-yl)but-3-yn-1-yl)oxy)dimethylsilane (**S60**)

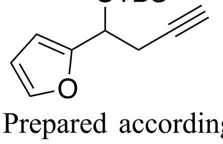

Prepared according to **General Procedure F** from 1-(furan-2-yl)but-3-yn-1-ol (233 mg, 1.71 mmol, 1.00 equiv.). Purified by flash column chromatography (silica, 0–5% Et_2O in hexane) to yield the desired compound as a pale-yellow oil (131 mg, 31%).

^{1}H NMR (500 MHz, $CDCl_3$) δ_H 7.38 (dd, J = 1.81, 0.86 Hz, 1H), 6.34 (dd, J = 3.28, 1.81 Hz, 1H), 6.29 – 6.24 (m, 1H), 4.87 (t, J = 6.64 Hz, 1H), 2.77 – 2.66 (m, 2H), 1.99 (t, J = 2.67 Hz, 1H), 0.90 (s, 9H), 0.12 (s, 3H), 0.00 (s, 3H).

^{13}C NMR (126 MHz, $CDCl_3$) δ_C 155.6, 141.7, 110.1, 106.4, 81.0, 70.0, 67.4, 27.3, 25.7, 18.2, −5.0, −5.0.

IR (ATR, film): 2930, 1759, 1254, 1107, 930, 837, 812, 779, 642 cm^{-1}.

HRMS (ESI): Calculated for 273.1462 *m/z*, found 273.1279 *m/z* $[C_{14}H_{22}O_2Si+Na]^+$.

2-(4-((*tert*-Butyldimethylsilyl)oxy)-4-(furan-2-yl)but-1-yn-1-yl)-6-methyl-1,3,6,2-dioxazaborocane-4,8-dione (**S61**)

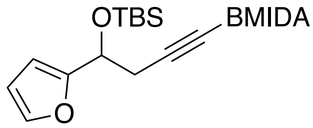

Prepared according to **General Procedure A** from *tert*-butyl((1-(furan-2-yl)but-3-yn-1-yl)oxy)dimethylsilane (142 mg, 568 µmol, 1.00 equiv.). Purified by precipitation from hexane/Et_2O to afford the desired compound as a red-brown solid (52.4 mg, 23%).

^{1}H NMR (500 MHz, Acetone-d_6) δ_H 7.49 (d, J = 1.34 Hz, 1H), 6.37 (m, 2H), 4.93 (t, J = 6.69 Hz, 1H), 4.21 (m, 2H), 3.94 (m, 2H), 3.04 (s, 3H), 2.77 (dd, J = 16.66, 7.02 Hz, 1H), 2.71 (dd, J = 16.64, 6.38 Hz, 1H), 0.87 (s, 9H), 0.12 (s, 3H), −0.03 (s, 3H).

^{13}C NMR (126 MHz, Acetone-d_6) δ_C 168.5, 168.5, 156.6, 142.8, 110.9, 107.6, 98.5, 68.1, 62.0, 62.0, 48.2, 28.6, 26.1, 18.7, −4.8, −4.8.

^{11}B NMR (96 MHz, Acetone-d_6) δ_B 6.1.

IR (ATR, film): 2955, 2207, 1771, 1749, 1541, 1508, 1032, 837, 779 cm^{-1}.

HRMS (ESI): Calculated for 428.1671 *m/z*, found 428.1677 *m/z* $[C_{19}H_{28}BNO_6Si+Na]^+$.

tert-Butyl((1-ethynylcyclohexyl)oxy)dimethylsilane (**S62**)

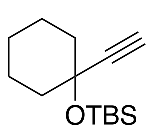

Prepared according to a modified **General Procedure F** from ethynylcyclohexanol (6.16 g, 49.6 mmol, 1.00 equiv.) in DMF (25.0 mL) at 60 ºC. Purified by flash column chromatography (silica, hexane) to yield the desired product as a colorless oil (9.43 g, 80%).

^{1}H NMR (400 MHz, $CDCl_3$) δ_H 2.48 (s, 1H), 1.82 (m, 2H), 1.66 (m 4H), 1.57 – 1.23 (m, 4H), 0.91 (s, 9H), 0.19 (s, 6H).

^{13}C NMR (101 MHz, $CDCl_3$) δ_C 88.5, 72.7, 69.0, 41.0, 25.8, 25.3, 22.7, 18.2, −2.8.

Spectral data consistent with the literature.[25]

2-((1-((*tert*-Butyldimethylsilyl)oxy)cyclohexyl)ethynyl)-6-methyl-1,3,6,2-dioxazaborocane-4,8-dione (**S63**)

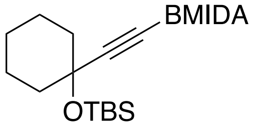

Prepared according to **General Procedure A** from *tert*-butyl((1-ethynylcyclohexyl)oxy)dimethylsilane (954 mg, 4.00 mmol, 1.00 equiv.). The product was purified by precipitation from hexane/Et_2O to afford the desired compound as a white powder (796 mg, 51%).

^{1}H NMR (400 MHz, Acetone-d_6) δ_H 4.30 (d, J = 17.00 Hz, 2H), 4.09 (d, J = 16.94 Hz, 2H), 3.26 (s, 3H), 1.80 (dd, J = 9.2, 5.0 Hz, 2H), 1.69 – 1.58 (m, 4H), 1.58 – 1.49 (m, 2H), 1.45 – 1.27 (m, 1H), 0.89 (s, 9H), 0.21 (s, 6H).

^{13}C NMR (101 MHz, Acetone-d_6) δ_C 168.5, 105.9, 70.2, 62.3, 48.5, 41.8, 26.2, 25.9, 23.5, 18.7, −2.5.

^{11}B NMR (96 MHz, Acetone-d_6) δ_B 6.3.

IR (ATR, film): 2932, 1773, 1273, 1258, 1090, 1032, 835, 773 cm^{-1}.

HRMS (ESI): Calculated for 416.2035 *m/z*, found 416.2041 *m/z* [$C_{19}H_{32}BNO_5Si$+Na]$^+$.

2-(6-(((*tert*-Butyldimethylsilyl)oxy)methyl)-2-tosylisoindolin-5-yl)-6-methyl-1,3,6,2-dioxazaborocane-4,8-dione (**S64**)

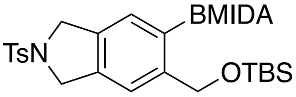

Prepared as previously reported.[21]

tert-Butyl (5-(((*tert*-butyldimethylsilyl)oxy)methyl)-6-(6-methyl-4,8-dioxo-1,3,6,2-dioxazaborocan-2-yl)-2,3-dihydro-1*H*-inden-2-yl)carbamate (**S65**)

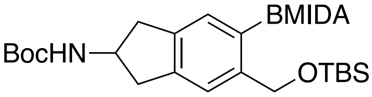

Prepared according to **General Procedure C** from 2-(3-((*tert*-butyldimethylsilyl)oxy)prop-1-yn-1-yl)-6-methyl-1,3,6,2-dioxazaborocane-4,8-dione (32.5 mg, 100 µmol, 1.00 equiv.). Purified by flash column chromatography (silica, 0–20% MeCN in CH_2Cl_2) to yield the desired product as a yellow solid (12.6 mg, 24%).

^{1}H NMR (500 MHz, Acetone-d_6) δ_H 7.37 (s, 1H), 7.30 (s, 1H), 6.31 (brs, 1H), 4.94 – 4.83 (m, 2H), 4.37 (d, J = 17.12 Hz, 3H), 4.18 (dd, J = 17.15, 2.84 Hz, 2H), 3.21 (td, J = 15.91, 7.40 Hz, 2H), 2.87 (dt, J = 15.26, 7.62 Hz, 2H), 2.79 (s, 3H), 1.42 (s, 9H), 0.95 (s, 9H), 0.15 (s, 3H), 0.14 (s, 3H).

^{13}C NMR (126 MHz, Acetone-d_6) δ_C 168.5, 168.5, 143.5, 142.6, 140.0, 130.3, 125.1, 77.7, 65.6, 62.9, 62.9, 48.3, 39.5, 39.5, 39.2, 39.1, 27.8, 25.7, 18.4, −5.8, −5.8.

^{11}B NMR (96 MHz, Acetone-d_6) δ_B 12.6.

IR (ATR, film): 2955, 2930, 2856, 1767, 1701, 1516, 1364, 1252, 1171, 1044, 835, 777 cm^{-1}.

HRMS (ESI): Calculated for 555.2668 *m/z*, found 555.2691 *m/z* [$C_{26}H_{41}BN_2O_7Si$+Na]$^+$.

2-(6-(((*tert*-Butyldimethylsilyl)oxy)methyl)-1,3-dihydroisobenzofuran-5-yl)-6-methyl-1,3,6,2-dioxazaborocane-4,8-dione (**S66**)

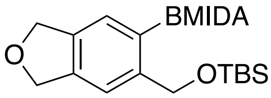

Prepared according to **General Procedure C** from 2-(3-((*tert*-butyldimethylsilyl)oxy)prop-1-yn-1-yl)-6-methyl-1,3,6,2-dioxazaborocane-4,8-dione (32.5 mg, 100 µmol, 1.00 equiv.). Purified by flash column chromatography (silica, 0–10% MeCN in CH_2Cl_2) to yield the desired product as a yellow solid (33.2 mg, 79%).

^{1}H NMR (500 MHz, Acetone-d_6) δ_H 7.46 (s, 1H), 7.43 (s, 1H), 5.05 (d, J = 2.18 Hz, 2H), 5.03 (d, J = 2.16 Hz, 2H), 4.94 (s, 2H), 4.39 (d, J = 17.14 Hz, 2H), 4.20 (d, J = 17.17 Hz, 2H), 2.80 (s, 3H), 0.95 (s, 9H), 0.15 (s, 6H).

^{13}C NMR (126 MHz, Acetone-d_6) δ_C 168.4, 144.5, 140.7, 138.0, 126.7, 121.2, 72.9, 72.8, 65.4, 62.9, 48.3, 25.7, 18.4, −5.9.

^{11}B NMR (96 MHz, Acetone-d_6) δ_B 12.4.

IR (ATR, film): 2953, 2930, 2855, 1763, 1462, 1339, 1290, 1194, 1044, 835, 779 cm^{-1}.

HRMS (ESI): Calculated for 442.18317 *m/z*, found 442.18271 *m/z* $[C_{20}H_{30}BNO_6Si+Na]^+$.

2-(6-(((*tert*-Butyldimethylsilyl)oxy)methyl)-2-(methylsulfonyl)isoindolin-5-yl)-6-methyl-1,3,6,2-dioxazaborocane-4,8-dione (**S67**)

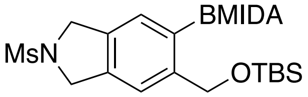

Prepared according to **General Procedure C** from 2-(3-((*tert*-butyldimethylsilyl)oxy)prop-1-yn-1-yl)-6-methyl-1,3,6,2-dioxazaborocane-4,8-dione (32.5 mg, 100 µmol, 1.00 equiv.). Purified by flash column chromatography (silica, 0–20% MeCN in CH_2Cl_2) to yield the desired product as a yellow solid (19.4 mg, 53%).

^1H NMR (500 MHz, Acetone-d_6) δ_H 7.50 (s, 1H), 7.48 (s, 1H), 4.94 (s, 2H), 4.71 (t, J = 2.11 Hz, 2H), 4.68 (t, J = 1.98 Hz, 2H), 4.40 (d, J = 17.15 Hz, 2H), 4.20 (d, J = 17.12 Hz, 2H), 2.94 (s, 3H), 2.81 (s, 3H), 0.96 (s, 9H), 0.16 (s, 6H).

^{13}C NMR (126 MHz, Acetone-d_6) δ_C 168.3, 145.0, 137.9, 135.3, 128.6, 122.7, 65.2, 62.9, 53.6, 53.4, 48.3, 33.3, 25.7, 18.3, −5.9.

^{11}B NMR (96 MHz, Acetone-d_6) δ_B 11.6.

IR (ATR, film): 2955, 2930, 2857, 1765, 1329, 1252, 1152, 1038, 835, 777, 519 cm^{-1}.

HRMS (ESI): Calculated for 519.17630 *m/z*, found 519.17580 *m/z* $[C_{21}H_{33}BN_2O_7Si+Na]^+$.

tert-Butyl 5-(((*tert*-butyldimethylsilyl)oxy)methyl)-6-(6-methyl-4,8-dioxo-1,3,6,2-dioxazaborocan-2-yl)isoindoline-2-carboxylate (**S68**)

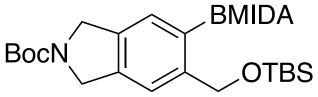

Prepared according to **General Procedure C** from 2-(3-((*tert*-butyldimethylsilyl)oxy)prop-1-yn-1-yl)-6-methyl-1,3,6,2-dioxazaborocane-4,8-dione (32.5 mg, 100 µmol, 1.00 equiv.). Purified by flash column chromatography (silica, 0–20% MeCN in CH_2Cl_2) to yield the desired product as a yellow solid (23.3 mg, 45%).

1:1 ratio of rotamers observed. Equivalent rotameric ^1H signals reported as ranges. All observed ^{13}C NMR signals reported.

^1H NMR (500 MHz, Acetone-d_6) δ 7.57 – 7.41 (m, 2H), 4.94 – 4.94 (m, 2H), 4.67 – 4.62(m, 4H), 4.39 (m, 2H), 4.20 (m, 2H), 2.80 – 2.80 (m, 3H), 1.51 – 1.51 (m, 9H), 1.21 – 1.21 (m, 3H), 0.96 – 0.96 (m, 9H), 0.16 – 0.16 (m, 6H).

^{13}C NMR (126 MHz, Acetone-d_6) δ 168.3, 168.3, 153.9, 144.6, 138.7, 138.2, 136.1, 135.6, 128.6, 128.5, 122.9, 122.8, 78.7, 78.6, 65.3, 62.9, 62.8, 54.6, 52.1, 51.9, 51.7, 48.3, 48.2, 27.8, 25.7, 17.9, −5.9, −5.9.

^{11}B NMR (96 MHz, Acetone-d_6) δ_B 12.4.

IR (ATR, film): 2928, 1765, 1702, 1402, 1366, 1252, 1169, 1101, 1034, 878, 835, 775 cm^{-1}.

HRMS (ESI): Calculated for 541.25167 *m/z*, found 541.25121 *m/z* $[C_{25}H_{39}BN2O_7Si+Na]^+$.

Diethyl 5-(((*tert*-butyldimethylsilyl)oxy)methyl)-6-(6-methyl-4,8-dioxo-1,3,6,2-dioxazaborocan-2-yl)-1,3-dihydro-2*H*-indene-2,2-dicarboxylate (**S69**)

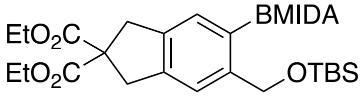

Prepared according to **General Procedure C** from 2-(3-((*tert*-butyldimethylsilyl)oxy)prop-1-yn-1-yl)-6-methyl-1,3,6,2-dioxazaborocane-4,8-dione (32.5 mg, 100 µmol, 1.00 equiv.). Purified by flash column chromatography (silica, 0–20% MeCN in CH_2Cl_2) to yield the desired product as a yellow solid (21.4 mg, 39%).

^{1}H NMR (500 MHz, Acetone-d_6) δ_H 7.39 (s, 1H), 7.34 (s, 1H), 4.89 (s, 2H), 4.36 (d, J = 17.08 Hz, 2H), 4.25 – 4.14 (m, 6H), 3.58 (m, 4H), 2.78 (s, 3H), 1.24 (t, J = 7.09 Hz, 6H), 0.95 (s, 9H), 0.15 (s, 6H).

^{13}C NMR (176 MHz, Acetone-d_6) δ_C 171.2, 168.4, 144.0, 141.3, 138.6, 129.9, 124.6, 65.4, 62.9, 61.3, 59.8 48.3, 40.3, 40.0, 25.7, 18.5, 13.4, −5.9.

^{11}B NMR (96 MHz, Acetone-d_6) δ_B 13.3.

IR (ATR, film): 2930, 1789, 1732, 1292, 1254, 1184, 1051, 837, 419, 411, 403 cm^{-1}.

HRMS (ESI): Calculated for 562.2638 *m/z*, found 562.2635 *m/z* $[C_{27}H_{40}BNO_9Si+H]^+$.

2-(4-(((*tert*-Butyldimethylsilyl)oxy)methyl)-1,3-dihydroisobenzofuran-5-yl)-6-methyl-1,3,6,2-dioxazaborocane-4,8-dione (**S70**)

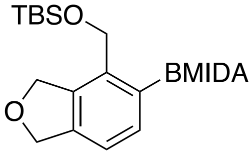

Prepared according to **General Procedure C** from 2-ethynyl-6-methyl-1,3,6,2-dioxazaborocane-4,8-dione (18.1 mg, 100 µmol, 1.00 equiv.). Purified by flash column chromatography (silica, 0–2.5% MeCN in CH_2Cl_2) to yield the desired product as a yellow solid (40.3 mg, 96%).

^{1}H NMR (400 MHz, Acetone-d_6) δ_H 7.44 (d, J = 7.64 Hz, 1H), 7.25 (d, J = 7.63 Hz, 1H), 5.20 (t, J = 2.08 Hz, 2H), 5.11 – 5.03 (m, 2H), 4.90 (s, 2H), 4.39 (d, J = 17.07 Hz, 2H), 4.17 (d, J = 17.08 Hz, 2H), 2.79 (s, 3H), 0.94 (s, 9H), 0.18 (s, 6H).

^{13}C NMR (101 MHz, Acetone-d_6) δ_C 168.3, 141.2, 139.8, 138.5, 133.7, 119.7, 72.9, 72.9, 62.5, 62.2, 48.0, 25.6, 18.1, −6.1.

^{11}B NMR (128 MHz, Acetone-d_6) δ 11.9.

IR (ATR, film): 2928, 2855, 1769, 1287, 1252, 1233, 1057, 1009, 837, 816, 777 cm^{-1}.

HRMS (ESI): Calculated for 420.2008 *m/z*, found 420.2001 *m/z* $[C_{20}H_{30}BNO_6Si+H]^+$.

2-(6-(2-((*tert*-Butyldimethylsilyl)oxy)-2-phenylethyl)-2-tosylisoindolin-5-yl)-6-methyl-1,3,6,2-dioxazaborocane-4,8-dione (**S71**)

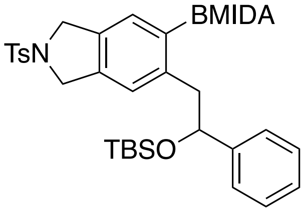

Prepared according to **General Procedure C** from 2-(4-((*tert*-butyldimethylsilyl)oxy)-4-phenylbut-1-yn-1-yl)-6-methyl-1,3,6,2-dioxazaborocane-4,8-dione (41.5 mg, 100 µmol, 1.00 equiv.). Purified by flash column

chromatography (silica, 0–2% MeCN in CH_2Cl_2) to yield the desired product as an off-white solid (19.1 mg, 29%).

^{1}H NMR (500 MHz, Acetone-d_6) δ_H 7.83 (d, J = 8.30 Hz, 2H), 7.65 – 7.58 (m, 2H), 7.47 – 7.41 (m, 2H), 7.36 – 7.27 (m, 3H), 7.26 – 7.19 (m, 1H), 7.18 (s, 1H), 6.81 (brs, 1H), 4.99 (dd, J = 10.25, 2.65 Hz, 1H), 4.74 – 4.48 (m, 4H), 4.40 (d, J = 17.58 Hz, 1H), 4.31 (d, J = 16.85 Hz, 1H), 4.23 (d, J = 17.55 Hz, 1H), 4.09 (d, J = 16.89 Hz, 1H), 3.11 (dd, J = 13.09, 2.71 Hz, 1H), 2.68 (s, 3H), 2.67 – 2.63 (m, 1H), 2.41 (s, 3H), 1.22 (s, 9H), 0.66 (m, 6H).

^{13}C NMR (126 MHz, Acetone-d_6) δ_C 168.9, 168.0, 146.4, 143.6, 143.6, 136.5, 134.0, 133.8, 129.9, 128.0, 127.9, 127.9, 127.7, 126.7, 126.0, 75.7, 62.2, 62.1, 54.6, 53.7, 53.7, 48.0, 46.9, 25.2, 24.0, 17.7, −5.9, −6.5.

^{11}B NMR (96 MHz, Acetone-d_6) δ_B 22.5.

IR (ATR, film): 1771, 1339, 1163, 1098, 1067, 1030, 702, 667, 424, 415, 405 cm^{-1}.

HRMS (ESI): Calculated for 663.2726 m/z, found 663.2727 m/z $[C_{34}H_{43}BN_2O_7SSi+H]^+$.

2-(6-(2-((*tert*-Butyldimethylsilyl)oxy)-2-(*o*-tolyl)ethyl)-2-tosyl-2,3-dihydro-1*H*-inden-5-yl)-6-methyl-1,3,6,2-dioxazaborocane-4,8-dione (**S72**)

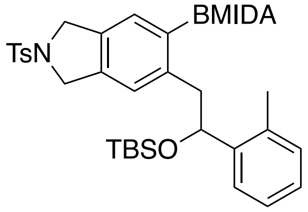

Prepared according to **General Procedure C** from 2-(4-((*tert*-butyldimethylsilyl)oxy)-4-(*o*-tolyl)but-1-yn-1-yl)-6-methyl-1,3,6,2-dioxazaborocane-4,8-dione (42.9 mg, 100 µmol, 1.00 equiv.). Purified by flash column chromatography (silica, 0–3% MeCN in CH_2Cl_2) to yield the desired product as an off-white solid (23.7 mg, 36%).

^{1}H NMR (500 MHz, Acetone-d_6) δ_H 7.85 – 7.77 (m, 2H), 7.57 (dd, J = 7.49, 1.47 Hz, 1H), 7.51 – 7.42 (m, 2H), 7.31 (s, 1H), 7.21 – 7.15 (m, 1H), 7.15 – 7.10 (m, 2H), 7.08 – 7.05 (m, 1H), 5.14 (t, J = 6.79 Hz, 1H), 4.62 (appq, J = 1.61 Hz, 2H), 4.59 – 4.48 (m, 2H), 4.33 (d, J = 10.63 Hz, 1H), 4.29 (d, J = 10.27 Hz, 1H), 4.11 (m, 2H), 3.02 (mf, 2H), 2.65 (s, 3H), 2.42 (s, 3H), 2.39 (s, 3H), 0.63 (s, 9H), −0.41 (s, 3H), −0.45 (s, 3H).

^{13}C NMR (126 MHz, Acetone-d_6) δ_C 168.6, 168.2, 143.6, 143.6, 143.3, 136.6, 134.6, 133.8, 133.8, 130.1, 129.9, 127.7, 127.7, 127.4, 127.3, 126.7, 125.7, 81.2, 62.1, 62.0, 53.7, 53.6, 46.9, 45.1, 25.2, 20.5, 18.8, 17.7, −5.8, −6.1.

^{11}B NMR (96 MHz, Acetone-d_6) δ_B 11.2.

IR (ATR, film): 2957, 1767, 1711, 1341, 1287, 1254, 1161, 1067, 1030, 835, 777, 667, 550 cm^{-1}.

HRMS (ESI): Calculated for 675.2726 m/z, found 675.2767 m/z $[C_{34}H_{43}BN_2O_7SSi-H]^-$.

2-(6-(2-((*tert*-Butyldimethylsilyl)oxy)-2-(*m*-tolyl)ethyl)-2-tosylisoindolin-5-yl)-6-methyl-1,3,6,2-dioxazaborocane-4,8-dione (**S73**)

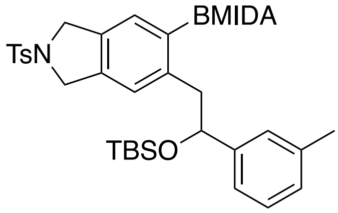

Prepared according to **General Procedure C** from 2-(4-((*tert*-butyldimethylsilyl)oxy)-4-(*m*-tolyl)but-1-yn-1-yl)-6-methyl-1,3,6,2-dioxazaborocane-4,8-dione (42.9 mg, 100 µmol, 1.00 equiv.). Purified by flash column chromatography (silica, 0–5% MeCN in CH_2Cl_2) to yield the desired product as a yellow solid (17.3 mg, 26%).

^{1}H NMR (500 MHz, Acetone-d_6) δ_H 7.85 – 7.79 (m, 2H), 7.50 (s, 1H), 7.45 (d, J = 8.01 Hz, 2H), 7.40 (d, J = 7.70 Hz, 1H), 7.33 (s, 1H), 7.23 – 7.15 (m, 2H), 7.04 (d, J = 7.46 Hz, 1H), 4.93 (dd, J = 10.25, 2.53 Hz, 1H), 4.72 – 4.50 (m, 4H), 4.39 (d, J = 17.57 Hz, 1H), 4.31 (d, J = 16.88 Hz, 1H), 4.22 (d, J = 17.55 Hz, 1H), 4.08 (d, J = 16.84 Hz, 1H), 3.10 (dd, J = 13.11, 2.60 Hz, 1H), 2.67 (s, 3H), 2.62 (dd, J = 13.12, 10.18 Hz, 1H), 2.41 (s, 3H), 2.34 (s, 3H), 0.67 (s, 9H), −0.46 (s, 3H), −0.70 (s, 3H).

^{13}C NMR (126 MHz, Acetone-d_6) δ_C 168.8, 168.0, 146.3, 143.7, 143.6, 137.2, 136.5, 133.9, 133.9, 129.9, 127.9, 127.9, 127.8, 127.7, 127.2, 126.8, 122.8, 75.8, 62.2, 62.1, 53.7, 53.7, 48.1, 46.9, 25.2, 25.2, 20.5, 17.1, −5.9, −6.5.

^{11}B NMR (96 MHz, Acetone-d_6) δ_B 11.8.

IR (ATR, film): 2926, 1773, 1707, 1331, 1287, 1161, 1092, 1032, 835, 775, 667, 550 cm^{-1}.

HRMS (ESI): Calculated for 699.2702 *m*/*z*, found 699.2456 *m*/*z* $[C_{35}H_{45}BN_2O_7SSi+Na]^+$.

2-(6-(2-((*tert*-Butyldimethylsilyl)oxy)-2-(3,5-dimethoxyphenyl)ethyl)-2-tosylisoindolin-5-yl)-6-methyl-1,3,6,2-dioxazaborocane-4,8-dione (**S74**)

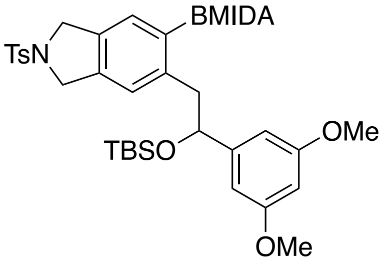

Prepared according to **General Procedure C** from 2-(4-((*tert*-butyldimethylsilyl)oxy)-4-(3,5-dimethoxyphenyl)but-1-yn-1-yl)-6-methyl-1,3,6,2-dioxazaborocane-4,8-dione (47.5 mg, 100 µmol, 1.00 equiv.). Purified by flash column chromatography (silica, 0–5% MeCN in CH_2Cl_2) to yield the desired product as a pale-pink solid (35.5 mg, 50%).

^{1}H NMR (500 MHz, Acetone-d_6) δ_H 7.90 – 7.79 (m, 2H), 7.51 – 7.37 (m, 2H), 7.32 (s, 1H), 7.18 (s, 1H), 6.81 (d, J = 2.29 Hz, 2H), 6.32 (t, J = 2.30 Hz, 1H), 4.93 (dd, J = 10.36, 2.64 Hz, 1H), 4.73 – 4.48 (m, 4H), 4.41 (d, J = 17.63 Hz, 1H), 4.32 (d, J = 16.81 Hz, 1H), 4.26 (d, J = 17.57 Hz, 1H), 4.10 (d, J = 16.85 Hz, 1H), 3.81 (s, 6H), 3.13 (dd, J = 13.05, 2.67 Hz, 1H), 2.69 (s, 3H), 2.62 (dd, J = 12.93, 10.48 Hz, 1H), 2.41 (s, 3H), 0.68 (s, 9H), −0.42 (s, 3H), −0.67 (s, 3H).

^{13}C NMR (126 MHz, Acetone-d_6) δ_C 169.0, 168.0, 160.8, 149.1, 143.6, 143.5, 136.5, 134.0, 133.8, 129.9, 128.2, 127.8, 127.7, 103.3, 99.4, 75.6, 62.2, 62.1, 54.7, 53.7, 53.7, 47.8, 46.9, 25.2, 20.5, 17.7, −5.9, −6.6.

^{11}B NMR (96 MHz, Acetone-d_6) δ_B 12.1.

IR (ATR, film): 1771, 1597, 1343, 1289, 1157, 1088, 1061, 1032, 835, 667 cm^{-1}.

HRMS (ESI): Calculated for 723.2937 *m*/*z*, found 723.2963 *m*/*z* $[C_{36}H_{47}BN_2O_9SSi+H]^+$.

2-(6-(2-((*tert*-Butyldimethylsilyl)oxy)-2-(thiophen-2-yl)ethyl)-2-tosylisoindolin-5-yl)-6-methyl-1,3,6,2-dioxazaborocane-4,8-dione (**S75**)

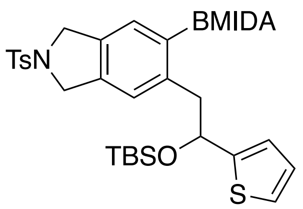

Prepared according to **General Procedure C** from 2-(4-((*tert*-butyldimethylsilyl)oxy)-4-(thiophen-2-yl)but-1-yn-1-yl)-6-methyl-1,3,6,2-dioxazaborocane-4,8-dione (42.1 mg, 100 μmol, 1.00 equiv.). Purified by flash column chromatography (silica, 0–7.5% MeCN in CH_2Cl_2) followed by precipitation from CH_2Cl_2-Hexane-Et_2O to yield the desired product as a white solid (38.0 mg, 57%).

^{1}H NMR (500 MHz, Acetone-d_6) δ_H 7.82 (d, J = 8.33 Hz, 2H), 7.51 – 7.40 (m, 2H), 7.33 (s, 1H), 7.30 (dd, J = 5.02, 1.21 Hz, 1H), 7.23 (dt, J = 3.46, 0.98 Hz, 1H), 7.14 (s, 1H), 6.95 (dd, J = 5.03, 3.48 Hz, 1H), 5.30 (dd, J = 10.05, 2.62 Hz, 1H), 4.79 – 4.48 (m, 4H), 4.41 (d, J = 17.57 Hz, 1H), 4.33 (d, J = 16.87 Hz, 1H), 4.26 (d, J = 17.57 Hz, 1H), 4.11 (d, J = 16.86 Hz, 1H), 3.18 (dd, J = 13.12, 2.71 Hz, 1H), 2.76 (dd, J = 13.18, 10.09 Hz, 1H), 2.71 (s, 3H), 2.41 (s, 3H), 0.70 (s, 9H), −0.36 (s, 3H), −0.69 (s, 3H).

^{13}C NMR (126 MHz, Acetone-d_6) δ_C 168.8, 168.0, 143.6, 142.9, 137.8, 136.7, 134.2, 133.8, 129.9, 128.0, 127.9, 127.7, 126.4, 123.3, 122.9, 72.5, 62.3, 62.2, 53.7, 53.7, 49.0, 47.0, 25.2, 20.5, 17.7, −6.1, −6.7.

^{11}B NMR (96 MHz, Acetone-d_6) δ_B 12.2.

IR (ATR, film): 2928, 2361, 1771, 1335, 1161, 1026, 667 cm^{-1}.

HRMS (ESI): Calculated for 669.2290 *m*/*z*, found 669.2273 *m*/*z* [$C_{32}H_{41}BN_2O_7S_2Si$+H]$^+$.

2-(6-(2-(4-Bromophenyl)-2-((*tert*-butyldimethylsilyl)oxy)ethyl)-2-tosylisoindolin-5-yl)-6-methyl-1,3,6,2-dioxazaborocane-4,8-dione (**S76**)

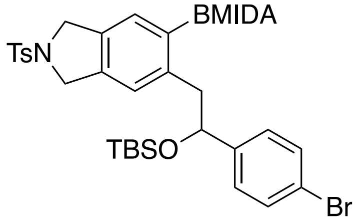

Prepared according to **General Procedure C** from 2-(4-((*tert*-butyldimethylsilyl)oxy)-4-(4-bromophenyl)but-1-yn-1-yl)-6-methyl-1,3,6,2-dioxazaborocane-4,8-dione (49.4 mg, 100 μmol, 1.00 equiv.). Purified by flash column chromatography (silica, 0–3% MeCN in CH_2Cl_2) to yield the desired product as an off-white solid (36.4 mg, 49%).

^{1}H NMR (400 MHz, Acetone-d_6) δ_H 7.84 – 7.79 (m, 2H), 7.61 – 7.57 (m, 2H), 7.52 – 7.47 (m, 2H), 7.47 – 7.42 (m, 2H), 7.33 (s, 1H), 7.17 (s, 1H), 4.96 (dd, J = 10.20, 2.49 Hz, 1H), 4.73 – 4.50 (m, 4H), 4.40 (d, J = 17.57 Hz, 1H), 4.32 (d, J = 16.84 Hz, 1H), 4.22 (d, J = 17.56 Hz, 1H), 4.10 (d, J = 16.83 Hz, 1H), 3.10 (dd, J = 13.08, 2.57 Hz, 1H), 2.68 (s, 3H), 2.62 (dd, J = 13.01, 10.32 Hz, 1H), 2.41 (s, 3H), 0.67 (s, 9H), −0.46 (s, 3H), −0.65 (s, 3H).

^{13}C NMR (101 MHz, Acetone-d_6) δ_C 168.9, 167.9, 145.8, 143.6, 143.2, 136.6, 134.1, 133.9, 130.9, 129.9, 128.2, 128.0, 127.9, 127.7, 120.0, 75.3, 62.2, 62.1, 53.7, 53.7, 47.9, 46.9, 25.2, 20.5, 17.7, −6.0, −6.5.

^{11}B NMR (128 MHz, Acetone-d_6) δ_B 11.5.

IR (ATR, film): 1767, 1341, 1287, 1252, 1161, 1082, 1030, 835, 667, 548 cm^{-1}.

HRMS (ESI): Calculated for 741.1831 *m*/*z*, found 741.1807 *m*/*z* [$C_{34}H_{42}BBrN_2O_7SSi$+H]$^+$.

2-(6-((1-((*tert*-Butyldimethylsilyl)oxy)cyclobutyl)methyl)-2-tosylisoindolin-5-yl)-6-methyl-1,3,6,2-dioxazaborocane-4,8-dione (**S77**)

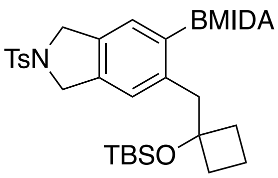

Prepared according to **General Procedure C** from 2-(3-(1-((*tert*-butyldimethylsilyl)oxy)cyclobutyl)prop-1-yn-1-yl)-6-methyl-1,3,6,2-dioxazaborocane-4,8-dione (37.9 mg, 100 µmol, 1.00 equiv.). Purified by flash column chromatography (silica, 0–5% MeCN in CH_2Cl_2) to yield the desired product as a white solid (33.9 mg, 54%).

^{1}H NMR (500 MHz, Acetone-d_6) δ_H 7.81 (d, J = 8.35 Hz, 2H), 7.51 (s, 1H), 7.43 (d, J = 8.07 Hz, 2H), 7.34 (s, 1H), 4.59 (appd, J = 2.20 Hz, 2H), 4.56 (appd, J = 2.14 Hz, 2H), 4.34 (d, J = 17.14 Hz, 2H), 4.13 (d, J = 17.12 Hz, 2H), 3.14 (s, 2H), 2.68 (s, 3H), 2.40 (s, 3H), 2.27 – 2.03 (m, 4H), 1.86 (m, 1H), 1.74 – 1.62 (m, 1H), 0.88 (s, 9H), 0.05 (s, 6H).

^{13}C NMR (126 MHz, Acetone-d_6) δ_C 168.3, 143.6, 142.9, 136.4, 133.9, 133.5, 129.8, 127.7, 127.7, 125.2, 76.8, 62.3, 53.6, 47.0, 43.8, 36.4, 25.5, 25.2, 20.5, 11.8, −3.2.

^{11}B NMR (128 MHz, Acetone-d_6) δ_B 11.5.

IR (ATR, film): 2953, 1771, 1341, 1263, 1163, 1098, 1032, 999, 835, 667, 419, 407 cm^{-1}.

HRMS (ESI): Calculated for 627.2726 *m/z*, found 627.2709 *m/z* $[C_{34}H_{42}BBrN_2O_7SSi+H]^+$.

2-(6-(2-((*tert*-Butyldimethylsilyl)oxy)ethyl)-2-tosylisoindolin-5-yl)-6-methyl-1,3,6,2-dioxazaborocane-4,8-dione (**S78**)

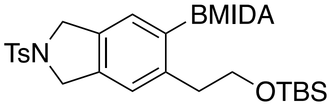

Prepared according to **General Procedure C** from 2-(3-((*tert*-butyldimethylsilyl)oxy)but-1-yn-1-yl)-6-methyl-1,3,6,2-dioxazaborocane-4,8-dione (339 mg, 1.00 mmol, 1.00 equiv.). Purified by flash column chromatography (silica, 0–15% MeCN in CH_2Cl_2) followed by precipitation from hexane:acetone:Et_2O to yield the desired product as an off-white solid (328 mg, 56%).

^{1}H NMR (500 MHz, Acetonitrile-d_3) δ_H 7.80 – 7.71 (m, 2H), 7.42 – 7.34 (m, 2H), 7.23 (s, 1H), 7.11 (s, 1H), 4.59 – 4.54 (m, 4H), 4.06 (d, J = 17.21 Hz, 2H), 3.89 (d, J = 17.20 Hz, 2H), 3.78 (t, J = 6.57 Hz, 2H), 2.80 (t, J = 6.59 Hz, 2H), 2.46 (s, 3H), 2.39 (s, 3H), 0.82 (s, 8H), −0.07 (s, 6H).

^{13}C NMR (126 MHz, Acetonitrile-d_3) δ_C 169.4, 145.0, 144.6, 138.1, 134.8, 134.4, 130.8, 129.1, 128.5, 126.6, 65.4, 63.3, 54.6, 54.5, 48.3, 39.8, 26.2, 21.5, 18.9, −5.3.

^{11}B NMR (96 MHz, CD_3CN) δ 12.4.

Spectral data consistent with the literature.[21]

2-(6-(3-((*tert*-Butyldiphenylsilyl)oxy)propyl)-2-tosylisoindolin-5-yl)-6-methyl-1,3,6,2-dioxazaborocane-4,8-dione (**S79**)

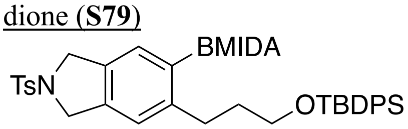

Prepared according to **General Procedure C** from 2-(5-((*tert*-butyldiphenylsilyl)oxy)pent-1-yn-1-yl)-6-methyl-1,3,6,2-dioxazaborocane-4,8-dione (47.7 mg, 100 µmol, 1.00 equiv.). Purified by flash column chromatography (silica, 0–5% MeCN in CH_2Cl_2) to yield the desired product as a white solid (33.3 mg, 46%).

^{1}H NMR (500 MHz, Acetonitrile-d_3) δ_H 7.79 – 7.73 (m, 2H), 7.70 – 7.64 (m, 4H), 7.51 – 7.45 (m, 2H), 7.45 – 7.36 (m, 6H), 7.22 (s, 1H), 7.03 (s, 1H), 4.59 – 4.54 (m, 2H), 4.54 – 4.52 (m, 2H), 4.05 (d, J = 17.31 Hz, 2H),

3.85 (d, J = 17.27 Hz, 2H), 3.71 (t, J = 6.39 Hz, 2H), 2.66 – 2.58 (m, 2H), 2.43 (s, 3H), 2.39 (s, 3H), 1.89 – 1.75 (m, 2H), 1.04 (s, 9H).

^{13}C NMR (126 MHz, Acetonitrile-d_3) δ_C 168.4, 146.8, 144.1, 137.5, 135.4, 133.9, 133.5, 133.4, 129.9, 129.8, 128.3, 127.8, 127.6, 124.2, 63.3, 62.4, 53.8, 53.5, 47.5, 35.4, 32.1, 26.3, 20.5, 18.8.

^{11}B NMR (96 MHz, Acetonitrile-d_3) δ_B 12.3.

IR (ATR, film): 2928, 1765, 1335, 1304, 1163, 1096, 1032, 704, 667, 550, 505 cm^{-1}.

HRMS (ESI): Calculated for 747.2702 *m/z*, found 747.2702 *m/z* [$C_{39}H_{45}BN_2O_7SSi+Na]^+$.

tert-Butyl 5-(((*tert*-butyldimethylsilyl)oxy)ethyl)-6-(6-methyl-4,8-dioxo-1,3,6,2-dioxazaborocan-2-yl)isoindoline-2-carboxylate (**S80**)

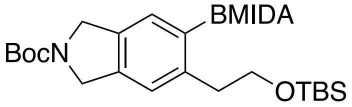

Prepared according to **General Procedure C** from 2-(3-((*tert*-butyldimethylsilyl)oxy)but-1-yn-1-yl)-6-methyl-1,3,6,2-dioxazaborocane-4,8-dione (33.9 mg, 100 μmol, 1.00 equiv.). Purified by flash column chromatography (silica, 0–20% MeCN in CH_2Cl_2) to yield the desired product as a yellow solid (21.4 mg, 40%).

1:1 ratio of rotamers observed. Equivalent rotameric ^{1}H signals reported as ranges. All observed ^{13}C NMR signals reported.

^{1}H NMR (500 MHz, Acetone-d_6) δ_H 7.42 – 7.38 (m, 1H), 7.27 – 7.24 (m, 1H), 4.65 – 4.56 (m, 4H), 4.37 (m, 2H), 4.19 (m, 2H), 3.89 – 3.87 (m, 2H), 2.96 – 2.92 (m, 2H), 2.79 – 2.79 (m, 3H), 1.51 – 1.51 (m, 9H), 0.89 – 0.89 (m, 9H), 0.01 – 0.01 (m, 6H).

^{13}C NMR (126 MHz, Acetone-d_6) δ_C 168.3, 168.3, 153.9, 143.1, 143.1, 138.4, 137.8, 135.0, 134.5, 128.3, 128.2, 125.6, 125.5, 78.6, 64.8, 64.8, 62.4, 62.4, 52.0, 51.9, 51.7, 51.7, 47.4, 47.3, 39.2, 39.2, 27.8, 25.5, 18.0, −6.1.

^{11}B NMR (96 MHz, Acetone-d_6) δ_B 12.3.

IR (ATR, film): 3435, 2928, 2857, 2359, 1686, 1404, 1366, 1254, 1171, 1098, 1030, 837, 775 cm^{-1}.

HRMS (ESI): Calculated for 278.1371 *m/z*, found 278.1398 *m/z* [$C_{26}H_{41}BN_2O_7Si+Na+H]^{2+}$.

2-(2-((4-Bromophenyl)sulfonyl)-6-(2-((*tert*-butyldimethylsilyl)oxy)ethyl)isoindolin-5-yl)-6-methyl-1,3,6,2-dioxazaborocane-4,8-dione (**S81**)

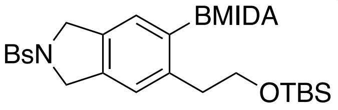

Prepared according to **General Procedure C** from 2-(3-((*tert*-butyldimethylsilyl)oxy)but-1-yn-1-yl)-6-methyl-1,3,6,2-dioxazaborocane-4,8-dione (67.9 mg, 200 μmol, 1.00 equiv.). Purified by flash column chromatography (silica, 0–5% MeCN in CH_2Cl_2) followed by precipitation from hexane:acetone:Et_2O to yield the desired product as a yellow solid (4.6 mg, 4%).

^{1}H NMR (500 MHz, Acetone-d_6) δ_H 7.90 – 7.86 (m, 2H), 7.85 – 7.81 (m, 2H), 7.33 (s, 1H), 7.18 (s, 1H), 4.66 – 4.61 (m, 4H), 4.35 (d, J = 17.16 Hz, 2H), 4.15 (d, J = 17.12 Hz, 2H), 3.82 (t, J = 6.65 Hz, 2H), 2.88 (t, J = 6.64 Hz, 2H), 2.73 (s, 3H), 0.84 (s, 9H), −0.06 (s, 6H).

^{13}C NMR (126 MHz, Acetone-d_6) δ_C 168.2, 143.7, 136.8, 136.1, 133.6, 132.5, 129.5, 128.2, 127.2, 125.6, 64.6, 62.4, 53.6, 53.6, 47.3, 39.1, 25.4, 18.0, −6.2.
^{11}B NMR (96 MHz, Acetone-d_6) δ_B 11.5.

IR (ATR, film): 2970, 1765, 1169, 1096, 1067, 1038, 1009, 837, 739, 619, 424 cm^{-1}.

HRMS (ESI): Calculated for 651.1362 *m/z*, found 651.1355 *m/z* $[C_{27}H_{36}BBrN_2O_7SSi+H]^+$.

tert-Butyl(hex-5-yn-1-yloxy)dimethylsilane (**S82**)

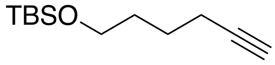

Prepared according to **General Procedure F** from 5-hexyn-1-ol (1.10 mL, 10.0 mmol, 1.00 equiv.). Purified by flash column chromatography (silica, hexane) to yield the desired product as a colorless oil (2.01 g, 95%).

^{1}H NMR (500 MHz CDCl$_3$) δ_H 3.66 (t, *J* = 5.89 Hz, 2H), 2.24 (td, *J* = 6.80, 2.62 Hz, 2H), 1.97 (t, *J* = 2.65 Hz, 1H), 1.70 – 1.56 (m, 4H), 0.92 (s, 9H), 0.07 (s, 6H).

^{13}C NMR (126 MHz, CDCl$_3$) δ_C 84.6, 68.3, 62.6, 31.8, 26.0, 25.7, 25.0, 18.2, −5.3.

Spectral data consistent with the literature.[26]

2-(6-((*tert*-Butyldimethylsilyl)oxy)hex-1-yn-1-yl)-6-methyl-1,3,6,2-dioxazaborocane-4,8-dione (**S83**)

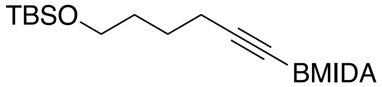

Prepared according to **General Procedure A** from *tert*-butyl(hex-5-yn-1-yloxy)dimethylsilane (425 mg, 0.500 mL, 2.00 mmol, 1.00 equiv.). Purified by precipitation from hexane/Et$_2$O to afford the desired compound as a white powder (78.9 mg, 11%).

^{1}H NMR (500 MHz, Acetone-*d*$_6$) δ_H 4.23 (d, *J* = 16.89 Hz, 2H), 4.05 (d, *J* = 16.88 Hz, 2H), 3.65 (t, *J* = 5.88 Hz, 2H), 3.20 (s, 3H), 2.30 – 2.22 (d, *J* = 6.58 Hz, 2H), 1.65 – 1.54 (m, 4H), 0.89 (s, 9H), 0.05 (s, 6H).

^{13}C NMR (126 MHz, Acetone-*d*$_6$) δ_C 168.6, 102.5, 63.1, 62.1, 48.3, 32.7, 26.3, 25.9, 19.6, 18.8, −5.2.

^{11}B NMR (128 MHz, Acetone-*d*$_6$) δ_B 6.2.

IR (ATR, film): 2953, 2203, 1749, 1458, 1287, 1252, 1165, 1030, 1009, 991, 837, 777 cm^{-1}.

HRMS (ESI): Calculated for 368.2059 *m/z*, found 368.2059 *m/z* $[C_{17}H_{30}BNO_5Si+H]^+$.

2-(6-(4-((*tert*-Butyldimethylsilyl)oxy)butyl)-2-tosylisoindolin-5-yl)-6-methyl-1,3,6,2-dioxazaborocane-4,8-dione (**S84**)

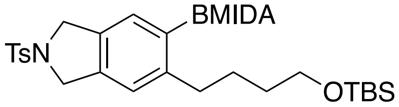

Prepared according to **General Procedure C** from 2-(6-((*tert*-butyldimethylsilyl)oxy)hex-1-yn-1-yl)-6-methyl-1,3,6,2-dioxazaborocane-4,8-dione (36.7 mg, 100 µmol, 1.00 equiv.). Purified by flash column chromatography (silica, 0–3% MeCN in CH$_2$Cl$_2$) to yield the desired product as a yellow solid (25.3 mg, 41%).

^{1}H NMR (400 MHz, Acetone-*d*$_6$) δ_H 7.85 – 7.77 (m, 2H), 7.47 – 7.39 (m, 2H), 7.32 (s, 1H), 7.14 (s, 1H), 4.57 (m, 4H), 4.36 (d, *J* = 17.20 Hz, 2H), 4.15 (d, *J* = 17.18 Hz, 2H), 3.65 (t, *J* = 6.06 Hz, 2H), 2.72 (s, 3H), 2.67 (t, *J* = 7.50 Hz, 2H), 2.40 (s, 3H), 1.69 – 1.52 (m, 4H), 0.89 (s, 9H), 0.05 (s, 6H).

^{13}C NMR (101 MHz, Acetone-*d*$_6$) δ_C 168.3, 147.5, 143.7, 137.4, 134.0, 133.4, 129.8, 128.3, 127.7, 124.0, 62.8, 62.6, 53.6, 53.5, 47.5, 35.7, 32.8, 29.3, 25.5, 20.5, 17.9, −6.0.

^{11}B NMR (128 MHz, Acetone-*d*$_6$) δ 11.5.

IR (ATR, film): 2928, 1767, 1341, 1290, 1254, 1163, 1096, 1059, 1032, 837, 667 cm^{-1}.
HRMS (ESI): Calculated for 615.2762 *m/z*, found 615.2715 *m/z* $[C_{30}H_{43}BN_2O_7SSi+H]^+$.

3. Product Characterization Data

2-(3-Hydroxyprop-1-yn-1-yl)-6-methyl-1,3,6,2-dioxazaborocane-4,8-dione (**1**)

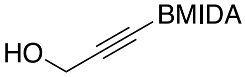

Prepared according to **General Procedure D** from 2-(3-((*tert*-butyldimethylsilyl)oxy)prop-1-yn-1-yl)-6-methyl-1,3,6,2-dioxazaborocane-4,8-dione (650 mg, 2.00 mmol, 1.00 equiv.). Purified by flash column chromatography (silica, 0–50% acetone in CH_2Cl_2) to yield the desired product as a white solid (338 mg, 80%).

^{1}H NMR (500 MHz, DMSO-d_6) δ_H 5.20 (brs, 1H), 4.26 (d, J = 17.14 Hz, 2H), 4.11 – 3.99 (m, 4H), 2.98 (s, 3H).

^{13}C NMR (126 MHz, DMSO-d_6) δ_C 169.1, 101.2, 61.8, 49.9, 48.2.

^{11}B NMR (96 MHz, DMSO-d_6) δ_B 6.1.

Spectral data consistent with the literature.[27]

3,5,6,7-Tetrahydro-1*H*-indeno[5,6-*c*][1,2]oxaborol-1-ol (**4**)

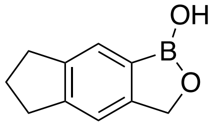

Prepared according to **General Procedure D** from 2-(3-((*tert*-butyldimethylsilyl)oxy)prop-1-yn-1-yl)-6-methyl-1,3,6,2-dioxazaborocane-4,8-dione (21.1 mg, 100 µmol, 1.00 equiv.) and heptadiyne (55.3 mg, 600 µmol, 6.00 equiv.). Purified by flash column chromatography (silica, 0–5% acetone in CH_2Cl_2) before precipitation from CH_2Cl_2:Et_2O to yield the desired product as a white powder (13.0 mg, 68%).

Prepared according to **General Procedure B** from 2-(3-hydroxyprop-1-yn-1-yl)-6-methyl-1,3,6,2-dioxazaborocane-4,8-dione (10.5 mg, 50.0 µmol, 1.00 equiv.). ^{1}H NMR using 1,4-dinitrobenzene (4.2 mg, 25.0 µmol, 0.50 equiv.) as internal standard revealed a yield of 73%.

^{1}H NMR (500 MHz, Acetonitrile-d_3) δ_H 7.56 (s, 1H), 7.25 (d, J = 1.49 Hz, 1H), 6.54 (s, 1H), 4.99 (s, 2H), 2.95 (ddd, J = 9.67, 5.47, 2.39 Hz, 4H), 2.14 – 2.06 (m, 2H).

^{13}C NMR (126 MHz, Acetonitrile-d_3) δ_C 156.8, 148.2, 130.9, 125.7, 117.1, 70.4, 32.5, 31.9, 25.5.

^{11}B NMR (96 MHz, Acetonitrile-d_3) δ_B 32.7.

IR: (ATR, film): 3310, 2361, 1410, 991 cm^{-1}.

HRMS (ESI): Calculated for 197.0744 *m/z*, found 197.0748 *m/z* $[C_{10}H_{11}BO_2+Na]^+$.

tert-Butyldimethyl(prop-2-yn-1-yloxy)silane (**5**)

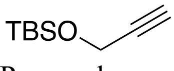

Prepared according to **General Procedure F** from propargyl alcohol (1.00 mL, 17.2 mmol, 1.00 equiv.). Used without further purification due to volatility of product, obtained as yellow oil (2.81 g, 96%).

^{1}H NMR (500 MHz, $CDCl_3$) δ_H 4.34 (d, J = 2.39 Hz, 2H), 2.40 (t, J = 2.40 Hz, 1H), 0.94 (s, 9H), 0.15 (s, 6H).

^{13}C NMR (126 MHz, CDCl3) δ_C 82.5, 72.8, 51.5, 25.8, 18.3, −5.2.

Spectral data consistent with the literature.[28]

2-(3-((*tert*-Butyldimethylsilyl)oxy)prop-1-yn-1-yl)-6-methyl-1,3,6,2-dioxazaborocane-4,8-dione (**6**)

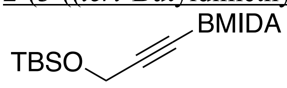

Prepared as previously described.[29]

6-Methyl-2-(prop-1-yn-1-yl)-1,3,6,2-dioxazaborocane-4,8-dione (**7**)

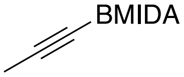

Prepared as previous described.[30]

2-(6-(4-((*tert*-Butyldimethylsilyl)oxy)butyl)-2-tosylisoindolin-5-yl)-6-methyl-1,3,6,2-dioxazaborocane-4,8-dione (**8**)

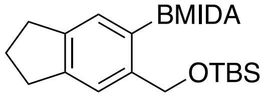

Prepared according to **General Procedure C** 2-(3-((*tert*-butyldimethylsilyl)oxy)prop-1-yn-1-yl)-6-methyl-1,3,6,2-dioxazaborocane-4,8-dione (32.5 mg, 200 µmol, 1.00 equiv.). Purified by flash column chromatography (silica, 0–2.5% MeCN in CH_2Cl_2) followed by precipitation of an impurity. The filtrate was concentrated *in vacuo* to yield the desired product as an orange solid (25.3 mg, 31%).

^{1}H NMR (400 MHz, $CDCl_3$) δ_H 7.42 (s, 1H), 7.16 (s, 1H), 4.82 (s, 2H), 4.02 (d, *J* = 16.54 Hz, 2H), 3.89 (d, *J* = 16.59 Hz, 2H), 2.92 (q, *J* = 7.89 Hz, 4H), 2.69 (s, 3H), 2.17 – 2.00 (m, 2H), 0.93 (s, 9H), 0.14 (s, 6H).

^{13}C NMR (101 MHz, $CDCl_3$) δ_C 167.7, 146.3, 143.7, 142.5, 130.0, 127.0, 66.5, 63.3, 48.9, 32.8, 32.6, 26.2, 25.2, 18.8, -5.0.

^{11}B NMR (128 MHz, $CDCl_3$) δ_B 12.0.

IR (ATR, film): 2928, 1759, 1462, 1337, 1296, 1250, 1180, 1028, 818 cm^{-1}.

HRMS (ESI): Calculated for 440.2035 *m*/*z*, found 440.2031 *m*/*z* $[C_{21}H_{32}BNO_5Si+Na]^+$.

6-Methyl-2-(6-methyl-2,3-dihydro-1*H*-inden-5-yl)-1,3,6,2-dioxazaborocane-4,8-dione (**9**)

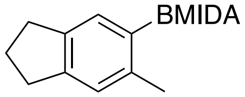

Prepared as previously described.[21]

6-Tosyl-3,5,6,7-tetrahydro-1*H*-[1,2]oxaborolo[3,4-*f*]isoindol-1-ol (**10**)

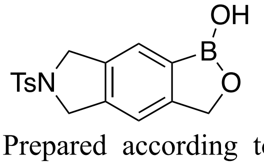

Prepared according to **General Procedure D** from 2-(3-((*tert*-butyldimethylsilyl)oxy)prop-1-yn-1-yl)-6-methyl-1,3,6,2-dioxazaborocane-4,8-dione (21.1 mg, 100 µmol, 1.00 equiv.) (148 mg, 600 µmol). Purified by flash column chromatography (silica, 0–5% acetone in CH_2Cl_2) before precipitation from CH_2Cl_2:Et_2O to yield the desired product as a white powder (7.9 mg, 23%).

Prepared according to **General Procedure B** from 2-(3-hydroxyprop-1-yn-1-yl)-6-methyl-1,3,6,2-dioxazaborocane-4,8-dione (10.5_mg, 50.0 µmol, 1.00 equiv.). ^{1}H NMR using 1,4-dinitrobenzene (4.2 mg, 25.0 µmol, 0.50 equiv.) as internal standard revealed a yield of 68%.

^{1}H NMR (500 MHz, Acetone-d_6) δ_H 7.85 – 7.78 (m, 2H), 7.59 (s, 1H), 7.49 – 7.42 (m, 2H), 7.31 (m, 1H), 4.98 (s, 2H), 4.63 (dt, *J* = 5.35, 1.96 Hz, 4H), 2.40 (s, 3H).

^{13}C NMR (126 MHz, Acetone-d_6) δ_C 154.2, 143.8, 139.5, 135.4, 133.8, 129.8, 127.7, 124.5, 115.7, 70.1, 53.4, 53.1, 20.5.

^{11}B NMR (96 MHz, Acetone-d_6) δ_B 32.6.

IR (ATR, film): 1163, 667, 600, 457, 419, 411, 403 cm^{-1}.

HRMS (ESI): Calculated for 352.07853 *m/z*, found 352.07817 *m/z* $[C_{16}H_{16}BNO_4S+Na]^+$.

tert-Butyl (1-hydroxy-3,5,6,7-tetrahydro-1*H*-indeno[5,6-*c*][1,2]oxaborol-6-yl)carbamate (**11**)

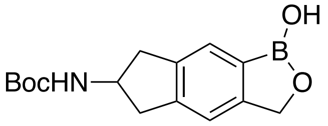

Prepared according to **General Procedure D** from *tert*-butyl (5-(((*tert*-butyldimethylsilyl)oxy)methyl)-6-(6-methyl-4,8-dioxo-1,3,6,2-dioxazaborocan-2-yl)-2,3-dihydro-1*H*-inden-2-yl)carbamate (20.6_mg, 38.7 µmol, 1.00 equiv.). Purified by flash column chromatography (silica, 0–50% MeCN in CH_2Cl_2) to yield the desired product as a yellow solid (7.0 mg, 63%).

Prepared according to **General Procedure B** from 2-(3-hydroxyprop-1-yn-1-yl)-6-methyl-1,3,6,2-dioxazaborocane-4,8-dione (10.5_mg, 50.0 µmol, 1.00 equiv.). ^{1}H NMR using 1,4-dinitrobenzene (4.2 mg, 25.0 µmol, 0.50 equiv.) as internal standard revealed a yield of 50%.

1:0.38 ratio of rotamers observed. Peaks of only major rotamer assigned.

^{1}H NMR (500 MHz, Acetone-*d*$_6$) δ_H 7.55 (s, 1H), 7.24 (s, 1H), 6.25 (brs, 1H), 4.97 (s, 2H), 4.39 (p, *J* = 7.08 Hz, 1H), 3.23 (dt, *J* = 14.52, 6.93 Hz, 2H), 2.89 (td, *J* = 15.16, 6.67 Hz, 2H), 1.42 (s, 9H).

^{13}C NMR (126 MHz, Acetone-*d*$_6$) δ_C 155.5, 153.4, 145.0, 140.5, 126.1, 117.4, 77.7, 70.2, 52.3, 39.4, 38.8, 27.8.

^{11}B NMR (96 MHz, Acetone-*d*$_6$) δ_B 32.4.

IR (ATR, film): 3337, 2930, 1682, 1520, 1478, 1408, 1393, 1364, 1254, 1167, 1047, 405 cm^{-1}.

Mass of Brown-type oxidation product detected.
HRMS (ESI): Calculated for 302.1366 *m/z*, found 302.1372 *m/z* $[C_{15}H_{21}NO_4+Na]^+$.

5,7-Dihydro-1*H*,3*H*-[1,2]oxaborolo[3,4-*f*]isobenzofuran-1-ol (**12**)

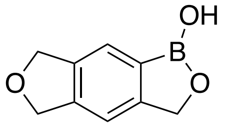

Prepared according to **General Procedure C** from 2-(3-((*tert*-butyldimethylsilyl)oxy)prop-1-yn-1-yl)-6-methyl-1,3,6,2-dioxazaborocane-4,8-dione (32.5 mg, 100 µmol, 1.00 equiv.) followed by **General Procedure D**. Purified by flash column chromatography (silica, 0–5% acetone in CH_2Cl_2) to yield the desired product as a white solid (17.4 mg, 98% over two steps).

Prepared according to **General Procedure B** from 2-(3-hydroxyprop-1-yn-1-yl)-6-methyl-1,3,6,2-dioxazaborocane-4,8-dione (10.5_mg, 50.0 µmol, 1.00 equiv.). ^{1}H NMR using 1,4-dinitrobenzene (4.2 mg, 25.0 µmol, 0.50 equiv.) as internal standard revealed a yield of 50%.

^{1}H NMR (500 MHz, Acetone-*d*$_6$) δ_H 8.08 (s, 1H), 7.62 (s, 1H), 7.40 – 7.29 (m, 1H), 5.08 – 5.02 (m, 4H), 5.03 (s, 2H).

^{13}C NMR (126 MHz, Acetone-*d*$_6$) δ_C 154.1, 143.0, 138.8, 122.6, 113.9, 72.6, 72.4, 70.2.

^{11}B NMR (128 MHz, Acetone-*d*$_6$) δ_B 32.3.

IR (ATR, film): 2924, 1630, 1416, 1373, 1059, 1034, 964, 901 cm^{-1}.

HRMS (ESI): Calculated for 176.06393 *m/z*, found 176.064594 *m/z* $[C_9H_9BO_3]^+$.

6-(Methylsulfonyl)-3,5,6,7-tetrahydro-1*H*-[1,2]oxaborolo[3,4-*f*]isoindol-1-ol (**13**)

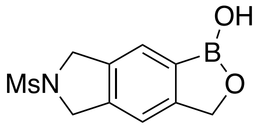

Prepared according to **General Procedure D** from 2-(6-(((*tert*-butyldimethylsilyl)oxy)methyl)-2-(methylsulfonyl)isoindolin-5-yl)-6-methyl-1,3,6,2-dioxazaborocane-4,8-dione (35.6_mg, 71.7 µmol, 1.00 equiv.). Purified by flash column chromatography (silica, 0–100% MeCN in CH_2Cl_2) to yield the desired product as a yellow solid (16.0 mg, 77%).

Prepared according to **General Procedure B** from 2-(3-hydroxyprop-1-yn-1-yl)-6-methyl-1,3,6,2-dioxazaborocane-4,8-dione (10.5_mg, 50.0 µmol, 1.00 equiv.). ^{1}H NMR using 1,4-dinitrobenzene (4.2 mg, 25.0 µmol, 0.50 equiv.) as internal standard revealed a yield of 69%.

^{1}H NMR (500 MHz, Acetone-d_6 + 1 drop D_2O) δ_H 7.70 (s, 1H), 7.38 (s, 1H), 5.02 (s, 2H), 4.72 – 4.70 (m, 2H), 4.69 (m, 2H), 2.95 (s, 3H).

^{13}C NMR (126 MHz, Acetone-d_6) δ_C 154.2, 139.9, 135.8, 124.7, 115.8, 70.1, 53.3, 53.0, 33.2.

^{11}B NMR (96 MHz, Acetone-d_6) δ_B 31.6.

IR: (ATR, solid): 3568, 3379, 2363, 2338, 1061 cm^{-1}.

HRMS (ESI): Calculated for 254.0653 *m*/*z*, found 254.0646 *m*/*z* $[C_{10}H_{12}BNO_4S+H]^+$.

tert-Butyl 1-hydroxy-5,7-dihydro-1*H*-[1,2]oxaborolo[3,4-*f*]isoindole-6(3*H*)-carboxylate (**14**)

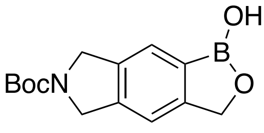

Prepared according to **General Procedure D** from *tert*-butyl 5-(((*tert*-butyldimethylsilyl)oxy)methyl)-6-(6-methyl-4,8-dioxo-1,3,6,2-dioxazaborocan-2-yl)isoindoline-2-carboxylate (49.3_mg, 95.1 µmol, 1.00 equiv.). Purified by flash column chromatography (silica, 0–2% Acetone in CH_2Cl_2) to yield the desired product as a yellow solid (20.7 mg, 77%).

1:1 ratio of rotamers observed. Equivalent rotameric ^{1}H signals reported as ranges. All observed ^{13}C NMR signals reported.

^{1}H NMR (500 MHz, Acetone-d_6) δ_H 8.13 – 8.13 (m, 1H), 7.67 – 7.65 (m, 1H), 7.38 – 7.36 (m, 1H), 5.10 – 5.00 (m, 2H), 4.66 (m, 4H), 1.52 – 1.52 (m, 9H).

^{13}C NMR (176 MHz, Acetone-d_6) δ_C 153.9, 140.8, 140.2, 136.6, 136.1, 124.5, 124.5, 115.7, 115.6, 78.7, 78.7, 70.2, 70.2, 51.9, 51.7, 51.5, 51.3, 27.8.

^{11}B NMR (96 MHz, Acetone-d_6) δ_B 32.5.

IR: (ATR, film): 3196, 1693, 1410, 1364, 1254, 1165, 1115, 855, 702, 527 cm^{-1}.

HRMS (ESI): Calculated for 298.1221 *m*/*z*, found 298.1218 *m*/*z* $[C_{10}H_{12}BNO_4S+H]^+$.

Diethyl 1-hydroxy-5,7-dihydro-1*H*-indeno[5,6-*c*][1,2]oxaborole-6,6(3*H*)-dicarboxylate (**15**)

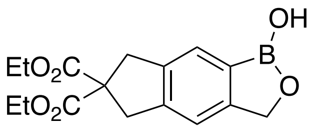

Prepared according to **General Procedure D** from diethyl 5-(((*tert*-butyldimethylsilyl)oxy)methyl)-6-(6-methyl-4,8-dioxo-1,3,6,2-dioxazaborocan-2-yl)-1,3-dihydro-2*H*-indene-2,2-dicarboxylate (56.2 mg, 100 µmol, 1.00 equiv.). Purified by flash column chromatography (silica, 0–5% Acetone in CH_2Cl_2) to yield the desired product as a yellow solid (13.4 mg, 42%).

Prepared according to **General Procedure B** from 2-(3-hydroxyprop-1-yn-1-yl)-6-methyl-1,3,6,2-dioxazaborocane-4,8-dione (10.5 mg, 50.0 µmol, 1.00 equiv.). ^{1}H NMR using 1,4-dinitrobenzene (4.2 mg, 25.0 µmol, 0.50 equiv.) as internal standard revealed a yield of 72%.

^{1}H NMR (500 MHz, Acetone-d_6) δ_H 8.00 (s, 1H), 7.56 (d, *J* = 1.38 Hz, 1H), 7.26 (d, *J* = 1.12 Hz, 1H), 4.98 (d, *J* = 1.20 Hz, 2H), 4.20 (q, *J* = 7.07 Hz, 4H), 3.59 (s, 2H), 3.59 – 3.57 (m, 2H), 1.25 (t, *J* = 7.10 Hz, 6H).

^{13}C NMR (126 MHz, Acetone-d_6) δ_C 171.0, 153.8, 143.6, 139.2, 125.8, 117.1, 70.1, 61.3, 60.3, 40.0, 39.5, 13.4.

^{11}B NMR (128 MHz, Acetone-d_6) δ_B 32.7.

IR: (ATR, film): 2922, 1732, 1410, 1366, 1279, 1256, 1240, 1190, 1067 cm^{-1}.

HRMS (ESI): Calculated for 319.1348 *m/z*, found 319.1350 *m/z* $[C_{16}H_{19}BO_6+H]^+$.

<u>6,8-Dihydro-[1,2]oxaborolo[4,3-*e*]isobenzofuran-3(1*H*)-ol (**16**)</u>

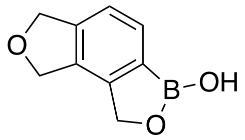

Prepared according to **General Procedure D** from 2-(4-(((*tert*-butyldimethylsilyl)oxy)methyl)-1,3-dihydroisobenzofuran-5-yl)-6-methyl-1,3,6,2-dioxazaborocane-4,8-dione (41.9 mg, 100 µmol, 1.00 equiv.). Purified by flash column chromatography (silica, 0–2% Acetone in CH_2Cl_2) followed by precipitation from CH_2Cl_2-acetone:Et_2O-hexane to yield the desired product as a white solid (13.4 mg, 76%).

^{1}H NMR (500 MHz, Acetone-d_6) δ_H 8.36 (s, 1H), 7.69 (d, *J* = 7.47 Hz, 1H), 7.32 (d, *J* = 7.45 Hz, 1H), 5.11 – 5.07 (m, 2H), 5.06 – 5.03 (m, 2H), 4.99 (s, 2H).

^{13}C NMR (126 MHz, Acetone-d_6) δ_C 147.5, 142.6, 132.4, 129.6, 119.9, 72.9, 71.1, 69.0.

^{11}B NMR (128 MHz, Acetone-d_6) δ_B 32.5.

IR: (ATR, film): 3381, 2924, 1618, 1443, 1402, 1385, 1354, 1042, 892, 729, 681, 640, 586, 538, 502 cm^{-1}.

HRMS (EI): Calculated for 176.06393 *m/z*, found 176.063962 *m/z* $[C_9H_9BO_3]^+$.

<u>*tert*-Butyl 1-hydroxy-3,4,6,8-tetrahydro-[1,2]oxaborinino[3,4-*f*]isoindole-7(1*H*)-carboxylate (**17**)</u>

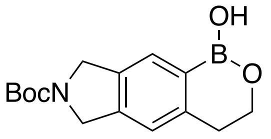

Prepared according to **General Procedure D** from *tert*-butyl 5-(2-((*tert*-butyldimethylsilyl)oxy)ethyl)-6-(6-methyl-4,8-dioxo-1,3,6,2-dioxazaborocan-2-yl)isoindoline-2-carboxylate (5.96 mg, 11.2 µmol, 1.00 equiv.). Purified by flash column chromatography (silica, 0–2% Acetone in CH_2Cl_2) to yield the desired product as a white solid (2.90 mg, 90%).

1:1 ratio of rotamers observed. Equivalent rotameric ^{1}H signals reported as ranges. All observed ^{13}C NMR signals reported.

^{1}H NMR (500 MHz, Acetone-d_6) δ_H 7.69 – 7.66 (m, 1H), 7.19 – 7.17 (m, 1H), 4.67 – 4.58 (m, 4H), 4.14 (m, 2H), 2.98 – 2.90 (m, 2H), 1.51 – 1.51 (m, 9H).

^{13}C NMR (176 MHz, Acetone-d_6) δ_C 153.9, 145.3, 140.6, 140.1, 127.2, 127.2, 121.1, 121.0, 78.7, 78.5, 63.6, 52.1, 51.9, 51.7, 51.5, 32.4, 22.5, 19.2, 13.5.

^{11}B NMR (96 MHz, Acetone-d_6) δ_B 28.4.

IR: (ATR, film): 3368, 2924, 2855, 2361, 2342, 1670, 1420, 1171, 419 cm^{-1}.

HRMS (ESI): Calculated for 312.13776 *m/z*, found 312.13753 *m/z* $[C_{15}H_{20}BNO_4+Na]^+$.

Compound spontaneously undergoes Brown-type oxidation to the corresponding phenol.

8-Tosyl-3,4,5,7,8,9-hexahydro-1*H*-[1,2]oxaborepino[3,4-*f*]isoindol-1-ol (**18**)

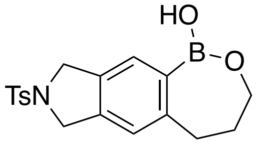

Prepared according to **General Procedure D** from 2-(6-(3-((*tert*-butyldiphenylsilyl)oxy)propyl)-2-tosylisoindolin-5-yl)-6-methyl-1,3,6,2-dioxazaborocane-4,8-dione (72.5 mg, 100 µmol, 1.00 equiv.). Purified by flash column chromatography (silica, 0–5% Acetone in CH_2Cl_2) to yield the desired product as a white solid (28.2 mg, 79%).

Prepared according to **General Procedure B** from 2-(5-hydroxypent-1-yn-1-yl)-6-methyl-1,3,6,2-dioxazaborocane-4,8-dione (12.0 mg, 50.0 µmol, 1.00 equiv.). ^1H NMR with 1,4-dinitrobenzene (4.2 mg, 25.0 µmol, 0.50 equiv.) as internal standard revealed a yield of 84%.

^1H NMR (500 MHz, Acetone-d_6) δ_H 7.87 – 7.77 (m, 2H), 7.47 (s, 1H), 7.44 (d, J = 8.03 Hz, 2H), 7.08 (s, 1H), 4.59 (s, 4H), 3.78 (t, J = 6.05 Hz, 2H), 2.80 (t, J = 6.91 Hz, 2H), 2.40 (s, 4H), 1.96 (p, J = 6.52 Hz, 2H).

^{13}C NMR (126 MHz, Acetone-d_6) δ_C 144.5, 143.7, 138.4, 133.9, 133.3, 129.8, 127.7, 127.7, 122.3, 62.7, 53.7, 53.4, 31.7, 30.0, 20.5.

^{11}B NMR (96 MHz, Acetone-d_6) δ_B 31.2.

IR: (ATR, solid): 2934, 1427, 1341, 1312, 1163, 1098, 667, 596, 550 cm^{-1}.

HRMS (ESI): Calculated for 358.1279 *m/z*, found 358.1284 *m/z* $[C_{18}H_{20}BNO_4S+H]^+$.

7-Tosyl-4,6,7,8-tetrahydro-[1,2]oxaborinino[3,4-*f*]isoindol-1(3*H*)-ol (**19**)

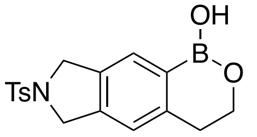

Prepared according to **General Procedure D** from 2-(6-(2-((tert-butyldimethylsilyl)oxy)ethyl)-2-tosylisoindolin-5-yl)-6-methyl-1,3,6,2-dioxazaborocane-4,8-dione (58.7 mg, 100 µmol, 1.00 equiv.). Purified by flash column chromatography (silica, 0–5% Acetone in CH_2Cl_2) to yield the desired product as a white solid (23.7 mg, 69%).

^1H NMR (500 MHz, Acetone-d_6) δ_H 7.81 (dd, J = 8.38, 2.04 Hz, 2H), 7.60 (s, 1H), 7.44 (d, J = 7.95 Hz, 2H), 7.10 (s, 1H), 4.69 – 4.58 (m, 4H), 4.08 (t, J = 5.93 Hz, 2H), 2.88 (t, J = 5.93 Hz, 2H), 2.40 (s, 3H).

^{13}C NMR (126 MHz, Acetone-d_6) δ_C 145.7, 143.7, 139.3, 133.9, 133.8, 129.8, 127.7, 127.1, 121.0, 63.5, 53.7, 53.3, 32.3, 20.5.

^{11}B NMR (96 MHz, Acetone-d_6) δ_B 28.3.

IR: (ATR, solid): 2970, 2901, 1435, 1408, 1387, 1423, 1314, 1157, 1101, 1067, 667, 600, 550, 411, 405 cm^{-1}.

HRMS (ESI): Calculated for 366.09418 *m/z*, found 366.09382 *m/z* [$C_{17}H_{18}BNO_4S+Na]^+$.

8-Methyl-6-tosyl-3,5,6,7-tetrahydro-1*H*-[1,2]oxaborolo[3,4-*f*]isoindol-1-ol (**20**)

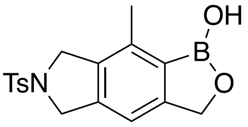

Prepared according to **General Procedure C** from 2-(3-((*tert*-butyldimethylsilyl)oxy)prop-1-yn-1-yl)-6-methyl-1,3,6,2-dioxazaborocane-4,8-dione (32.5 mg, 100 µmol, 1.00 equiv.). Purified by flash column chromatography (silica, 0–5% MeCN in CH_2Cl_2) followed by deprotection under **General Procedure D** to yield the crude product, which was purified by flash column chromatography (silica, 0–2% Acetone in CH_2Cl_2) followed by precipitation from CH_2Cl_2-acetone:Et_2O-hexane to yield the desired product as a white solid (22.8 mg, 66%, note this compound has very poor solubility characteristics thus HMBC data required in order to determine all ^{13}C signals).

^1H NMR (700 MHz, Acetone-d_6) δ_H 7.88 (s, 1H), 7.86 – 7.82 (m, 2H), 7.44 (d, *J* = 8.21 Hz, 2H), 7.09 (s, 1H), 4.92 (s, 2H), 4.64 (d, *J* = 2.05 Hz, 2H), 4.59 (s, 2H), 2.41 (s, 6H).

^{13}C NMR (176 MHz, Acetone-d_6) δ_C 154.7, 143.7, 143.7, 139.5, 136.7, 133.5, 129.8, 127.7, 112.8, 69.7, 53.8, 52.3, 20.5, 16.0.

^{11}B NMR (128 MHz, Acetone-d_6) δ_B 32.4.

IR (ATR, film): 2845, 1451, 1339, 1158, 1099, 1018, 818, 664, 654, 604, 548, 540 cm^{-1}.

HRMS (ESI): Calculated for 344.1122 *m/z*, found 344.1129 *m/z* [$C_{17}H_{18}BNO_4S+H]^+$.

7'-Tosyl-4',6',7',8'-tetrahydro-1'*H*-spiro[cyclobutane-1,3'-[1,2]oxaborinino[3,4-*f*]isoindol]-1'-ol (**21**)

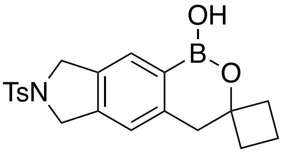

Prepared according to **General Procedure D** from 2-(6-((1-((*tert*-butyldimethylsilyl)oxy)cyclobutyl)methyl)-2-tosylisoindolin-5-yl)-6-methyl-1,3,6,2-dioxazaborocane-4,8-dione (20.1 mg, 32.1 µmol, 1.00 equiv.). Purified by flash column chromatography (silica, 0–2% Acetone in CH_2Cl_2) to yield the desired product as a yellow solid (11.5 mg, 94%).

Prepared according to **General Procedure B** from 2-(3-(1-hydroxycyclobutyl)prop-1-yn-1-yl)-6-methyl-1,3,6,2-dioxazaborocane-4,8-dione (13.3 mg, 50.0 µmol, 1.00 equiv.). ^1H NMR with 1,4-dinitrobenzene (4.2 mg, 25.0 µmol, 0.50 equiv.) as internal standard revealed a yield of 82%.

^1H NMR (500 MHz, Acetone-d_6) δ_H 7.84 – 7.77 (m, 2H), 7.57 (s, 1H), 7.45 – 7.41 (m, 2H), 7.34 (s, 1H), 7.16 – 7.13 (m, 1H), 4.60 (d, *J* = 1.35 Hz, 4H), 3.02 (s, 2H), 2.40 (s, 3H), 2.13 – 2.03 (m, 2H), 1.97 (ddt, *J* = 10.07, 8.73, 3.11 Hz, 2H), 1.75 (dtt, *J* = 11.31, 9.92, 3.32 Hz, 1H), 1.67 – 1.58 (m, 1H).

^{13}C NMR (126 MHz, Acetone-d_6) δ_C 144.1, 143.7, 139.4, 134.0, 133.8, 129.8, 127.7, 126.9, 122.0, 76.4, 53.7, 53.3, 40.5, 34.8, 20.5, 12.2.

^{11}B NMR (128 MHz, Acetone-d_6) δ_B 28.0.

IR: (ATR, film): 1435, 1389, 1343, 1290, 1273, 1163, 1098, 1063, 667, 583, 550 cm^{-1}.

HRMS (ESI): Calculated for 384.1435 *m/z*, found 384.1428 *m/z* [$C_{20}H_{23}BNO_4S+H]^+$.

3-Phenyl-7-tosyl-4,6,7,8-tetrahydro-[1,2]oxaborinino[3,4-*f*]isoindol-1(3*H*)-ol (**22**)

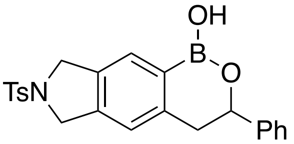

Prepared according to **General Procedure D** from 2-(6-(2-((*tert*-butyldimethylsilyl)oxy)-2-phenylethyl)-2-tosylisoindolin-5-yl)-6-methyl-1,3,6,2-dioxazaborocane-4,8-dione (18.7 mg, 28.2 µmol, 1.00 equiv.). Purified by flash column chromatography (silica, 0–5% Acetone in CH_2Cl_2) to yield the desired product as a yellow solid (10.1 mg, 85%).

Prepared according to **General Procedure B** from 2-(4-hydroxy-4-phenylbut-1-yn-1-yl)-6-methyl-1,3,6,2-dioxazaborocane-4,8-dione (15.1 mg, 50.0 µmol, 1.00 equiv.). ^{1}H NMR with 1,4-dinitrobenzene (4.2 mg, 25.0 µmol, 0.50 equiv.) as internal standard revealed a yield of >99%.

^{1}H NMR (500 MHz, Acetone-d_6) δ_H 7.87 – 7.79 (m, 2H), 7.66 (s, 1H), 7.53 (s, 1H), 7.50 – 7.42 (m, 4H), 7.40 – 7.34 (m, 2H), 7.31 – 7.26 (m, 1H), 7.12 (s, 1H), 5.24 (dd, J = 10.19, 4.19 Hz, 1H), 4.64 – 4.60 (m, 4H), 3.19 – 2.97 (m, 2H), 2.41 (s, 3H).

^{13}C NMR (126 MHz, Acetone-d_6) δ_C 145.0, 143.7, 143.3, 139.6, 134.2, 133.8, 129.8, 128.2, 127.7, 127.3, 127.1, 125.7, 121.2, 75.6, 53.7, 53.3, 40.3, 20.5.

^{11}B NMR (128 MHz, Acetone-d_6) δ_B 28.7.

IR: (ATR, film): 1435, 1393, 1339, 1308, 1279, 1161, 1096, 1059, 754, 700, 665, 596, 546, 530 cm^{-1}.

HRMS (ESI): Calculated for 420.1435 *m*/*z*, found 420.1436 *m*/*z* $[C_{16}H_{19}BO_6+H]^+$.

3-(*o*-Tolyl)-7-tosyl-4,6,7,8-tetrahydro-[1,2]oxaborinino[3,4-*f*]isoindol-1(3*H*)-ol (**23**)

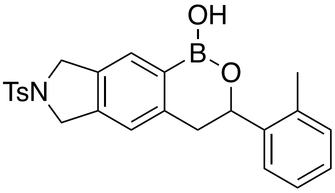

Prepared according to **General Procedure D** from 2-(6-(2-((*tert*-butyldimethylsilyl)oxy)-2-(*o*-tolyl)ethyl)-2-tosylisoindolin-5-yl)-6-methyl-1,3,6,2-dioxazaborocane-4,8-dione (30.0 mg, 44.3 µmol, 1.00 equiv.). Purified by flash column chromatography (silica, 0–5% Acetone in CH_2Cl_2) to yield the desired product as a yellow solid (10.1 mg, 53%).

Prepared according to **General Procedure B** from 2-(4-hydroxy-4-(*o*-tolyl)but-1-yn-1-yl)-6-methyl-1,3,6,2-dioxazaborocane-4,8-dione (15.8 mg, 50.0 µmol, 1.00 equiv.). ^{1}H NMR with 1,4-dinitrobenzene (4.2 mg, 25.0 µmol, 0.50 equiv.) as internal standard revealed a yield of >99%.

^{1}H NMR (700 MHz, Acetone-d_6) δ_H 7.85 – 7.81 (m, 2H), 7.67 (s, 1H), 7.55 – 7.53 (m, 1H), 7.51 (s, 1H), 7.44 (d, J = 8.00 Hz, 2H), 7.23 (td, J = 7.24, 2.03 Hz, 1H), 7.20 – 7.16 (m, 2H), 7.13 (s, 1H), 5.41 (dd, J = 10.44, 4.12 Hz, 1H), 4.64 – 4.60 (m, 4H), 3.08 – 2.93 (m, 2H), 2.41 (s, 3H), 2.35 (s, 3H).

^{13}C NMR (176 MHz, Acetone-d_6) δ_C 145.3, 143.7, 141.0, 139.5, 134.3, 134.2, 133.8, 130.2, 129.8, 127.7, 127.2, 127.1, 125.9, 125.6, 121.2, 73.0, 53.7, 53.3, 38.9, 20.5, 18.2.

^{11}B NMR (128 MHz, Acetone-d_6) δ_B 29.3.

IR (ATR, film): 1435, 1339, 1161, 1098, 667, 602, 548 cm^{-1}.

HRMS (ESI): Calculated for 456.1411 *m*/*z*, found 456.1431 *m*/*z* $[C_{24}H_{24}BNO_4S+Na]^+$.

3-(*m*-Tolyl)-7-tosyl-4,6,7,8-tetrahydro-[1,2]oxaborinino[3,4-*f*]isoindol-1(3*H*)-ol (**24**)

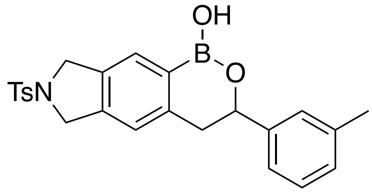

Prepared according to **General Procedure D** from 2-(6-(2-((*tert*-butyldimethylsilyl)oxy)-2-(*m*-tolyl)ethyl)-2-tosylisoindolin-5-yl)-6-methyl-1,3,6,2-dioxazaborocane-4,8-dione (31.6 mg, 46.7 µmol, 1.00 equiv.). Purified by flash column chromatography (silica, 0–5% Acetone in CH_2Cl_2) to yield the desired product as a yellow solid (11.6 mg, 85%).

Prepared according to **General Procedure B** from 2-(4-hydroxy-4-(*m*-tolyl)but-1-yn-1-yl)-6-methyl-1,3,6,2-dioxazaborocane-4,8-dione (15.8 mg, 50.0 µmol, 1.00 equiv.). ^{1}H NMR with 1,4-dinitrobenzene (4.2 mg, 25.0 µmol, 0.50 equiv.) as internal standard revealed a yield of >99%.

^{1}H NMR (500 MHz, Acetone-d_6) δ_H 7.79 (dd, J = 8.40, 2.43 Hz, 2H), 7.51 – 7.41 (m, 2H), 7.38 (d, J = 7.97 Hz, 1H), 7.23 – 7.17 (m, 2H), 7.10 – 7.01 (m, 1H), 6.92 (s, 1H), 6.73 (s, 1H), 4.95 – 4.88 (m, 1H), 4.54 – 4.49 (m, 2H), 4.46 (m, 2H), 3.01 – 2.87 (m, 2H), 2.41 (s, 3H), 2.31 (s, 3H).

^{13}C NMR (126 MHz, Acetone-d_6) δ_C155.6, 145.2, 143.7, 137.4, 135.7, 133.9, 129.8, 129.3, 128.0, 127.7, 126.7, 126.3, 125.4, 122.7, 109.9, 74.6, 53.6, 53.1, 41.6, 20.6, 20.5.

^{11}B NMR (128 MHz, Acetone-d_6) δ_B 27.2.

IR: (ATR, film): 2361, 1645, 1339, 1161, 667, 411 cm^{-1}.

HRMS (ESI): Calculated for 406.1471 *m*/*z*, found 406.1496 *m*/*z* $[C_{24}H_{24}BNO_4S-BO]^+$.

Compound spontaneously undergoes Brown-type oxidation to the corresponding phenol.

3-(*p*-Tolyl)-7-tosyl-4,6,7,8-tetrahydro-[1,2]oxaborinino[3,4-*f*]isoindol-1(3*H*)-ol (**25**)

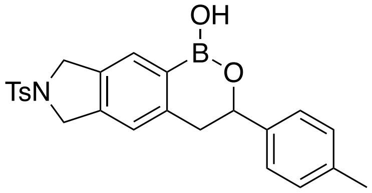

Prepared according to **General Procedure C** from 2-(6-(2-((*tert*-butyldimethylsilyl)oxy)-2-(*p*-tolyl)ethyl)-2-tosylisoindolin-5-yl)-6-methyl-1,3,6,2-dioxazaborocane-4,8-dione (85.9 mg, 200 µmol, 1.00 equiv.). Purified by flash column chromatography (silica, 0–5% MeCN in CH_2Cl_2) followed by deprotection under **General Procedure D** to yield the crude product which was purified by flash column chromatography (silica, 0–3% Acetone in CH_2Cl_2) followed by precipitation from CH_2Cl_2:Et_2O:hexane to yield the desired product as a yellow solid (41.3 mg, 54%).

Prepared according to **General Procedure B** from 2-(4-hydroxy-4-(*p*-tolyl)but-1-yn-1-yl)-6-methyl-1,3,6,2-dioxazaborocane-4,8-dione (15.8 mg, 50.0 µmol, 1.00 equiv.). ^{1}H NMR with 1,4-dinitrobenzene (4.2 mg, 25.0 µmol, 0.50 equiv.) as internal standard revealed a yield of 84%.

^{1}H NMR (500 MHz, Acetone-d_6) δ_H 7.87 – 7.79 (m, 2H), 7.65 (s, 1H), 7.51 (s, 1H), 7.44 (dt, J = 7.88, 0.73 Hz, 2H), 7.36 – 7.31 (m, 2H), 7.21 – 7.15 (m, 2H), 7.11 (d, J = 1.15 Hz, 1H), 5.20 (dd, J = 9.63, 4.62 Hz, 1H), 4.67 – 4.54 (m, 4H), 3.11 – 2.99 (m, 2H), 2.41 (s, 3H), 2.32 (s, 3H).

^{13}C NMR (126 MHz, Acetone-d_6) δ_C 145.1, 143.7, 140.3, 139.5, 136.7, 134.1, 133.8, 129.8, 128.7, 127.7, 127.1, 125.6, 121.2, 75.5, 53.7, 53.3, 40.3, 20.5, 20.2.

^{11}B NMR (128 MHz, Acetone-d_6) δ_B 29.0.

IR: (ATR, film): 2988, 1437, 1393, 1341, 1308, 1163, 1098, 667, 442, 415 cm^{-1}.

HRMS (ESI): Calculated for 434.1592 *m/z*, found 434.1589 *m/z* $[C_{24}H_{24}BNO_4S+H]^+$.

3-(3,5-Dimethoxyphenyl)-7-tosyl-4,6,7,8-tetrahydro-[1,2]oxaborinino[3,4-*f*]isoindol-1(3H)-ol (**26**)

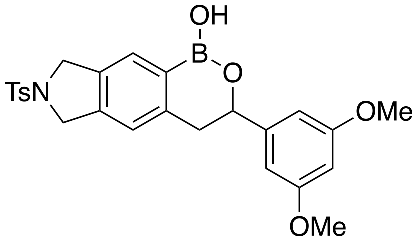

Prepared according to **General Procedure D** from 2-(6-(2-((*tert*-butyldimethylsilyl)oxy)-2-(3,5-dimethoxyphenyl)ethyl)-2-tosylisoindolin-5-yl)-6-methyl-1,3,6,2-dioxazaborocane-4,8-dione (8.1 mg, 11.0 µmol, 1.00 equiv.). Purified by flash column chromatography (silica, 0–2% Acetone in CH_2Cl_2) to yield the desired product as a yellow solid (5.3 mg, 99%).

Prepared according to **General Procedure B** from 2-(4-hydroxy-4-(3,5-dimethoxyphenyl)but-1-yn-1-yl)-6-methyl-1,3,6,2-dioxazaborocane-4,8-dione (18.1_mg, 50.0 µmol, 1.00 equiv.). ^{1}H NMR with 1,4-dinitrobenzene (4.2 mg, 25.0 µmol, 0.50 equiv.) as internal standard revealed a yield of 80%.

^{1}H NMR (500 MHz, Acetone-d_6) δ_H 7.81 (d, J = 8.34 Hz, 2H), 7.45 (d, J = 8.05 Hz, 2H), 7.13 (m, 2H), 6.52 (d, J = 2.31 Hz, 2H), 6.34 (t, J = 2.30 Hz, 1H), 4.79 (dt, J = 9.32, 5.15 Hz, 1H), 4.56 (m, 4H), 3.73 (s, 6H), 2.98 – 2.87 (m, 2H), 2.41 (s, 3H).

^{13}C NMR (126 MHz, Acetone-d_6) δ_C 160.7, 148.1, 143.7, 139.1, 135.9, 133.8, 129.8, 129.2, 127.7, 123.8, 122.1, 103.8, 98.6, 74.6, 54.6, 53.5, 53.4, 45.8, 20.5.

^{11}B NMR (96 MHz, Acetone-d_6) δ_B 26.3.

IR: (ATR, film): 1597, 1344, 1204, 1159, 1098, 1061, 667, 548, 430, 407 cm^{-1}.

Mass of protodeboronated material detected.
HRMS (ESI): Calculated for 454.1683 *m/z*, found 454.1677 *m/z* $[C_{25}H_{26}BNO_6S+H]^+$.

3-(4-Bromophenyl)-7-tosyl-4,6,7,8-tetrahydro-[1,2]oxaborinino[3,4-*f*]isoindol-1(3*H*)-ol (**27**)

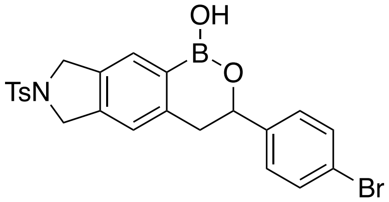

Prepared according to **General Procedure D** from 2-(6-(2-(4-bromophenyl)-2-((*tert*-butyldimethylsilyl)oxy)ethyl)-2-tosylisoindolin-5-yl)-6-methyl-1,3,6,2-dioxazaborocane-4,8-dione (37.1 mg, 50.0 µmol, 1.00 equiv.). Purified by flash column chromatography (silica, 0–2% Acetone in CH_2Cl_2) to yield the desired product as a yellow solid (22.1 mg, 89%).

Prepared according to **General Procedure B** from 2-(4-hydroxy-4-(4-bromophenyl)but-1-yn-1-yl)-6-methyl-1,3,6,2-dioxazaborocane-4,8-dione (19.0 mg, 50.0 µmol, 1.00 equiv.). ^{1}H NMR with 1,4-dinitrobenzene (4.2 mg, 25.0 µmol, 0.50 equiv.) as internal standard revealed a yield of 49%.

^{1}H NMR (500 MHz, Acetone-d_6) 7.82 (d, J = 8.22 Hz, 2H), 7.66 (s, 1H), 7.58 – 7.52 (m, 2H), 7.44 (d, J = 8.61 Hz, 5H), 7.12 (s, 1H), 5.24 (dd, J = 10.88, 3.54 Hz, 1H), 4.69 – 4.53 (m, 4H), 3.17 – 2.97 (m, 2H), 2.41 (s, 3H).

^{13}C NMR (126 MHz, Acetone-d_6) δ_C 144.8, 143.7, 142.7, 139.7, 134.3, 133.8, 131.2, 129.8, 127.8, 127.7, 127.1, 121.2, 120.6, 75.0, 53.7, 53.3, 40.1, 20.5.

^{11}B NMR (128 MHz, Acetone-d_6) δ_B 28.7.

IR: (ATR, film): 2924, 2853, 1489, 1437, 1387, 1341, 1308, 1203, 1098, 1068, 1089, 816 cm^{-1}.

Mass of protodeboronated material detected.
HRMS (ESI): Calculated for 454.1683 *m/z*, found 454.1677 *m/z* [$C_{25}H_{27}NO_5S$+H]$^+$.

Compound spontaneously undergoes Brown-type oxidation to the corresponding phenol.

3-(Thiophen-2-yl)-7-tosyl-4,6,7,8-tetrahydro-[1,2]oxaborinino[3,4-*f*]isoindol-1(3*H*)-ol (**28**)

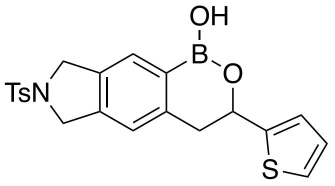

Prepared according to **General Procedure C** from 2-(4-((*tert*-butyldimethylsilyl)oxy)-4-(thiophen-2-yl)but-1-yn-1-yl)-6-methyl-1,3,6,2-dioxazaborocane-4,8-dione (84.3 mg, 200 µmol, 1.00 equiv.). Purified by flash column chromatography (silica, 0–5% MeCN in CH_2Cl_2) followed by **General Procedure D**. Purified by flash column chromatography (0–5% acetone in CH_2Cl_2) to yield the desired product as a white solid (24.3 mg, 29%).

Prepared according to **General Procedure D** from 2-(4-hydroxy-4-(thiophen-2-yl)but-1-yn-1-yl)-6-methyl-1,3,6,2-dioxazaborocane-4,8-dione (61.4 mg, 200 µmol, 1.00 equiv.) followed by **General Procedure B** on the crude product (due to instability of the intermediate alcohol). ^{1}H NMR with 1,4-dinitrobenzene (4.2 mg, 25.0 µmol, 0.50 equiv.) as internal standard revealed a yield of 51%.

^{1}H NMR (700 MHz, Acetone-d_6) δ_H 7.82 (d, *J* = 8.28 Hz, 2H), 7.64 (s, 1H), 7.60 (s, 1H), 7.44 (d, *J* = 8.05 Hz, 2H), 7.35 (dd, *J* = 5.07, 1.18 Hz, 1H), 7.16 (s, 1H), 7.06 (dt, *J* = 3.49, 1.14 Hz, 1H), 6.98 (dd, *J* = 5.01, 3.48 Hz, 1H), 5.51 (ddd, *J* = 9.54, 3.82, 0.97 Hz, 1H), 4.62 (s, 4H), 3.25 (dd, *J* = 16.02, 3.84 Hz, 1H), 3.17 (dd, *J* = 16.00, 9.54 Hz, 1H), 2.41 (s, 3H).

^{13}C NMR (176 MHz, Acetone-d_6) δ_C 146.9, 144.3, 143.7, 139.7, 134.3, 133.8, 129.8, 127.7, 127.1, 126.5, 124.5, 123.7, 121.4, 71.8, 53.7, 53.3, 39.7, 20.5.

^{11}B NMR (128 MHz, Acetone-d_6) δ_B 28.0.

IR (ATR, film): 1707, 1437, 1339, 1306, 1221, 1159, 1096, 1059, 814, 708, 665, 600, 548, 415, 407 cm^{-1}.

HRMS (ESI): Calculated for 426.1000 *m/z*, found 426.0999 *m/z* [$C_{21}H_{20}BNO_4S_2$+H]$^+$.

7-((4-Bromophenyl)sulfonyl)-4,6,7,8-tetrahydro-[1,2]oxaborinino[3,4-*f*]isoindol-1(3*H*)-ol (**29**)

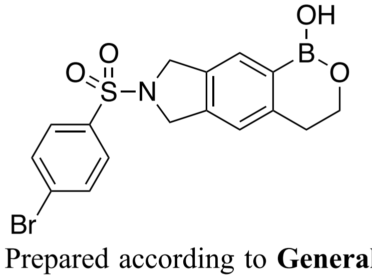

Prepared according to **General Procedure D** from *tert*-butyl 5-(2-((*tert*-butyldimethylsilyl)oxy)ethyl)-6-(6-methyl-4,8-dioxo-1,3,6,2-dioxazaborocan-2-yl)isoindoline-2-carboxylate (20.3 mg, 31.2 µmol, 1.00 equiv.). Purified by flash column chromatography (silica, 0–2% Acetone in CH_2Cl_2) to yield the desired product as a white solid (11.4 mg, 90%).

Prepared according to **General Procedure B** from 2-(4-hydroxypent-1-yn-1-yl)-6-methyl-1,3,6,2-dioxazaborocane-4,8-dione (11.3 mg, 50.0 µmol, 1.00 equiv.). ^{1}H NMR with 1,4-dinitrobenzene (4.2 mg, 25.0 µmol, 0.50 equiv.) as internal standard revealed a yield of 73%.

^{1}H NMR (500 MHz, Acetone-d_6) δ_H 7.92 – 7.86 (m, 2H), 7.83 (d, *J* = 8.66 Hz, 2H), 7.60 (s, 1H), 7.33 (s, 1H), 7.11 (s, 1H), 4.64 (s, 4H), 4.09 (t, *J* = 5.94 Hz, 2H), 2.89 (t, *J* = 6.01 Hz, 2H).

^{13}C NMR (176 MHz, Acetone-d_6) δ_C 145.8, 139.1, 136.2, 133.8, 132.5, 129.5, 127.2, 127.1, 121.0, 63.5, 53.7, 53.3, 32.3.

^{11}B NMR (96 MHz, Acetone-d_6) δ_B 29.5.

IR (ATR, film): 2924, 2855, 1736, 1343, 1167, 1096, 1069, 1009, 741, 621, 419 cm^{-1}.

HRMS (ESI): Calculated for 408.0071 *m*/*z*, found 408.0072 *m*/*z* $[C_{16}H_{15}BbrNO_4S+H]^+$.

Compound spontaneously undergoes Brown-type oxidation to the corresponding phenol.

2-(6-(4-Hydroxybutyl)-2-tosylisoindolin-5-yl)-6-methyl-1,3,6,2-dioxazaborocane-4,8-dione (**38**)

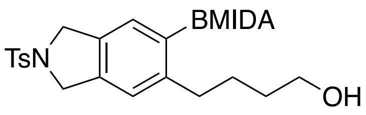

Prepared according to **General Procedure D** from 2-(6-(4-((*tert*-butyldimethylsilyl)oxy)butyl)-2-tosylisoindolin-5-yl)-6-methyl-1,3,6,2-dioxazaborocane-4,8-dione (30.7 mg, 50.0 µmol, 1.00 equiv.). Purified by flash column chromatography (silica, 0–2% Acetone in CH_2Cl_2) to yield the desired product as a white solid (3.0 mg, 12%).

^{1}H NMR (700 MHz, Acetone-d_6) δ_H 7.81 (d, *J* = 8.14 Hz, 2H), 7.43 (d, *J* = 7.97 Hz, 2H), 7.31 (s, 1H), 7.13 (s, 1H), 4.59 (d, *J* = 2.16 Hz, 2H), 4.57 (d, *J* = 2.07 Hz, 2H), 4.37 – 4.32 (m, 2H), 4.16 (d, *J* = 17.29 Hz, 2H), 3.55 (q, *J* = 5.54, 5.10 Hz, 2H), 3.43 (s, 1H), 2.72 (d, *J* = 1.88 Hz, 3H), 2.68 – 2.64 (m, 2H), 2.40 (s, 3H), 1.67 – 1.52 (m, 4H).

^{13}C NMR (176 MHz, Acetone-d_6) δ_C 168.5, 147.6, 143.7, 137.4, 133.9, 133.3, 129.8, 128.2, 127.7, 124.0, 62.6, 61.4, 53.6, 53.5, 47.5, 35.7, 32.9, 29.4, 20.5.

^{11}B NMR (128 MHz, Acetone-d_6) δ_B 11.8.

IR: (ATR, film): 2924, 2361, 1749, 1339, 1161, 1095, 889 cm^{-1}.

HRMS (ESI): Calculated for 523.1681 *m*/*z*, found 523.1676 *m*/*z* $[C_{24}H_{29}BN_2O_7S+Na]^+$.

Compound decomposes readily on silica.

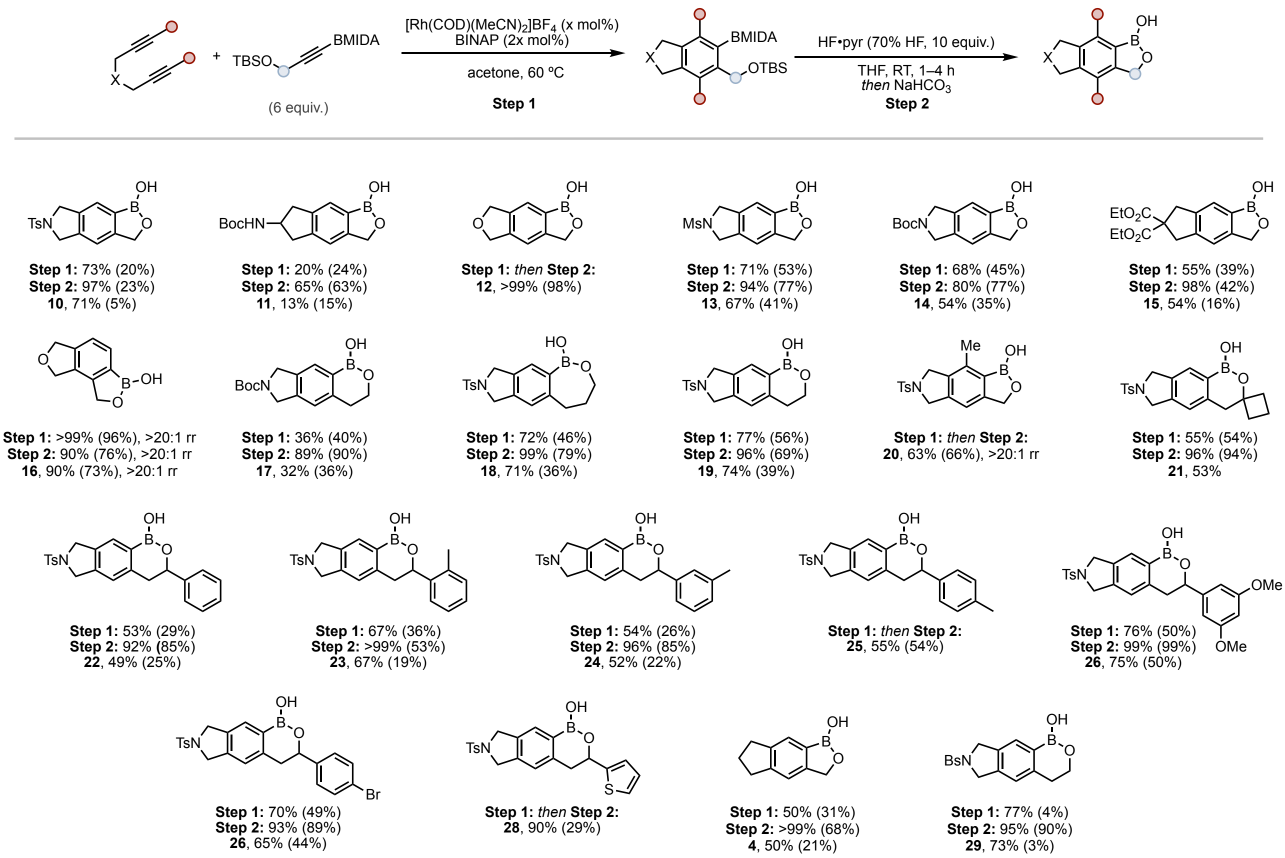

Scheme S1: ^{1}H NMR yield of [2+2+2] cycloaddition (Step 1). ^{1}H NMR yield of desilylation (Step 2). Yield over two steps. Isolated yields shown in brackets. All ^{1}H NMR yields determined using 1,4-dinitrobenzene (0.5 equiv.) as internal standard.

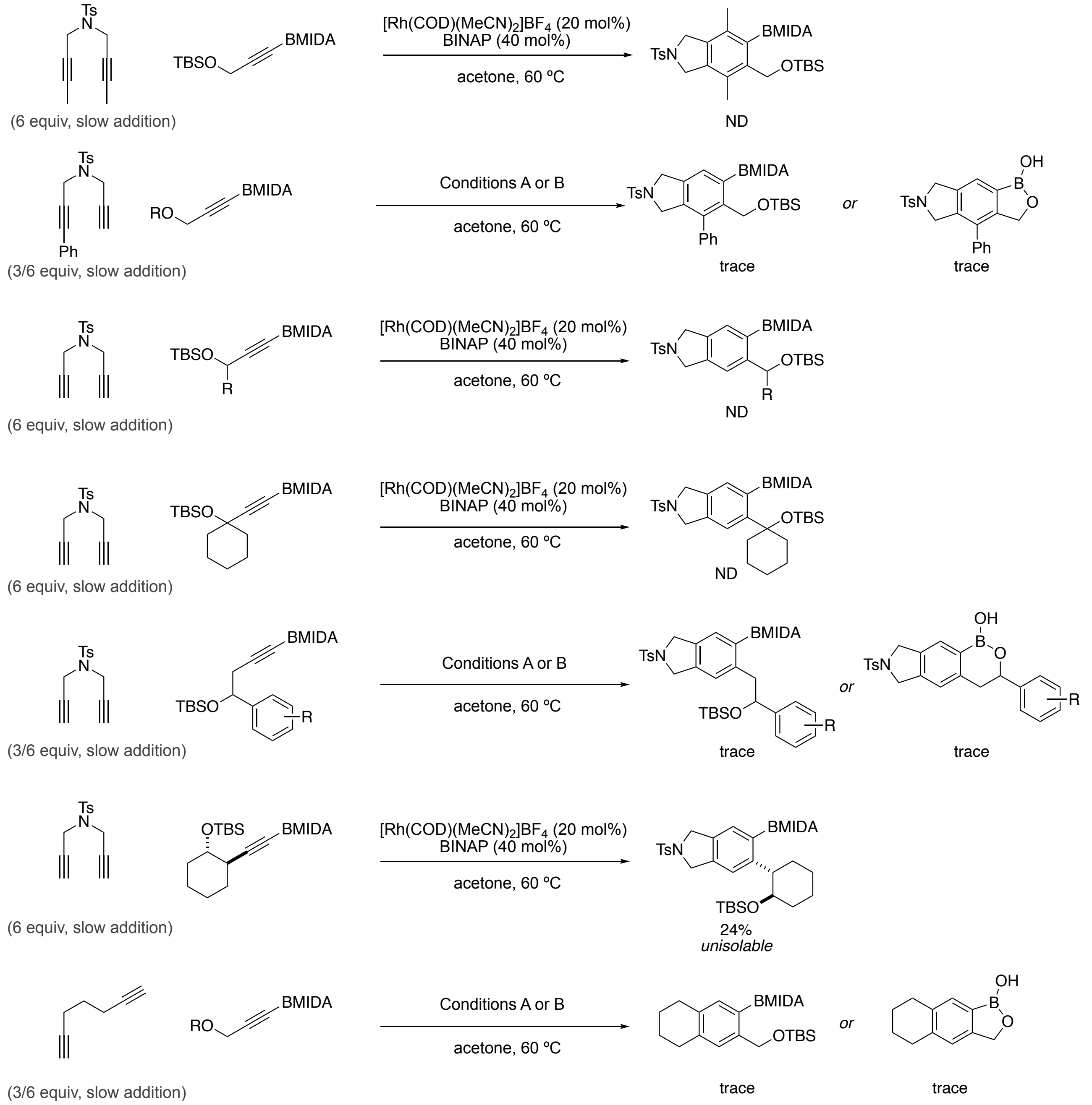

Scheme S2: Extended list of unsuccessful substrates and further information.

4. NMR Association Constant Determination

Association constants were determined using a procedure disclosed by Hall and coworkers.[30, 31]

A buffer solution of 0.1 M KH_2PO_4 was made in D_2O, and the pH was adjusted to 7.4 by addition of 4 M NaOH in D_2O. 47.5 µmol of the boronic acid/ester was dissolved in 3.125 mL of buffer solution (a few drops of DMSO were added if not fully solubilized). A solution of the sugar (0.15 M) was made by dissolving 0.15 mmol of the sugar in 1 mL of the boronic acid solution, and the pH was again adjusted to 7.4. To 700 µL of boronic acid solution was added 40 µL of the sugar solution, and afterwards aliquots of 10 µL were added to obtain a range of sugar concentrations in the range of 8 – 26 mM.

Table S1: Fructose tavaborole titration.

volume fruct solution added	Int free Tavaborole	int complexed Tavaborole	ratio	Amount [S] added	total volume	[S]	1/ratio	1/[S]
40	1	0.7	0.41176471	0.000006	740	0.008108108	2.42857143	123.333333
50	1	0.92	0.47916667	0.0000075	750	0.01	2.08695652	100
60	1	1.15	0.53488372	0.000009	760	0.011842105	1.86956522	84.4444444
70	1	1.58	0.6124031	0.0000105	770	0.013636364	1.63291139	73.3333333
80	1	1.66	0.62406015	0.000012	780	0.015384615	1.60240964	65
90	1	2.27	0.6941896	0.0000135	790	0.017088608	1.44052863	58.5185185
100	1	2.58	0.72067039	0.000015	800	0.01875	1.3875969	53.3333333
120	1	3.42	0.77375566	0.000018	820	0.02195122	1.29239766	45.5555556
140	1	3.72	0.78813559	0.000021	840	0.025	1.2688172	40

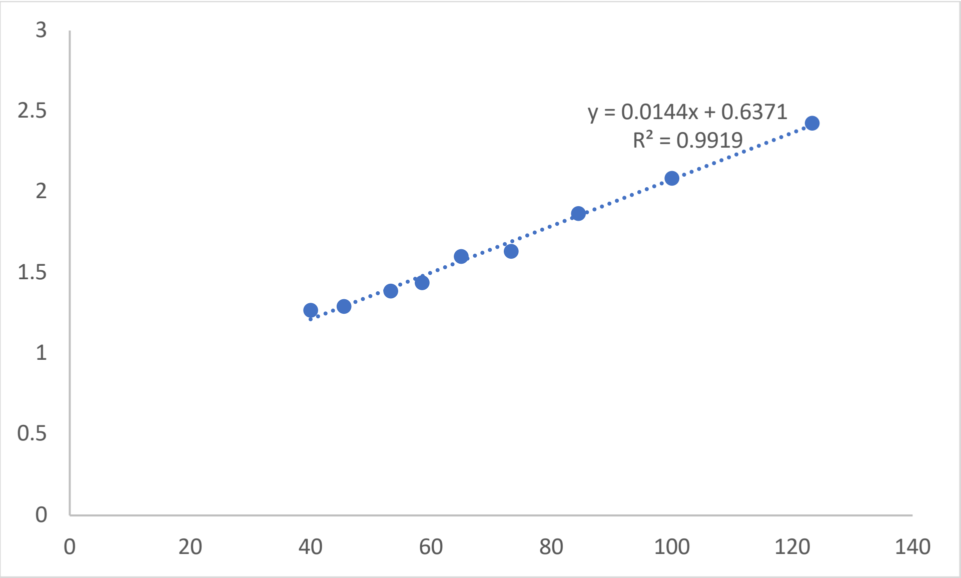

Figure S1: Benesi-Hildebrand plot of the tavaborole-fructose complex.

Table S2: Guanosine tavaborole titration.

volume guan solution added	Int free Tavaborole	int complexed Tavaborole	ratio	Amount [S] added	total volume	[S]	1/ratio	1/[S]
40	1	0.39	0.28057554	0.000006	640	0.009375	3.564102564	106.6666667
50	1	0.47	0.319727891	0.0000075	650	0.011538462	3.127659574	86.66666667
60	1	0.59	0.371069182	0.000009	660	0.013636364	2.694915254	73.33333333
70	1	0.79	0.441340782	0.0000105	670	0.015671642	2.265822785	63.80952381
80	1	1.03	0.507389163	0.000012	680	0.017647059	1.970873786	56.66666667
90	1	1.12	0.528301887	0.0000135	690	0.019565217	1.892857143	51.11111111
100	1	1.33	0.570815451	0.000015	700	0.021428571	1.751879699	46.66666667
120	1	2.6	0.722222222	0.000018	720	0.025	1.384615385	40
140	1	1.94	0.659863946	0.000021	740	0.028378378	1.515463918	35.23809524

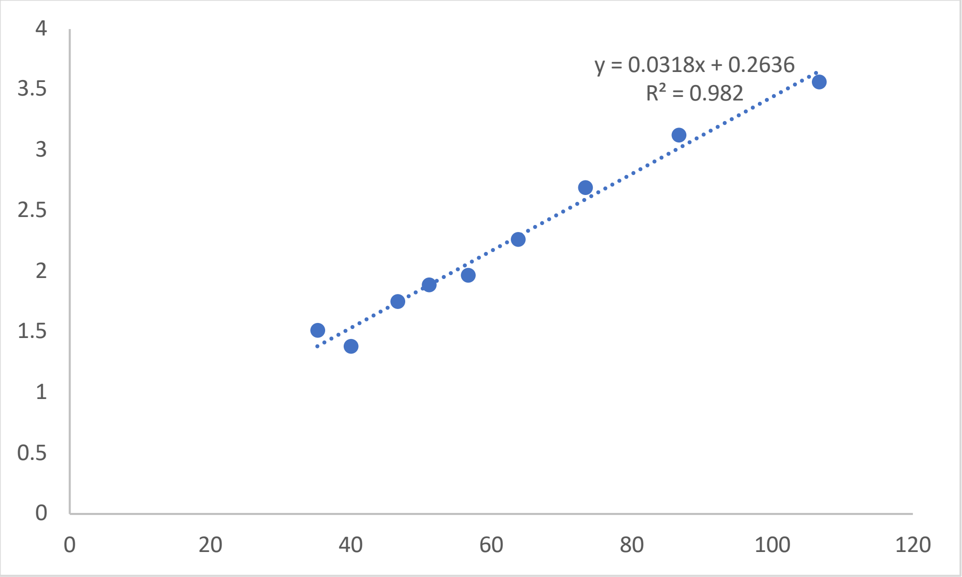

Figure S2: Benesi-Hildebrand plot of the tavaborole-guanosine complex.

Table S3: Phenylboronic acid fructose titration.

volume fruct solution added	Int free PhB	int complexed PhB	ratio	Amount [S] added	total volume	[S]	1/ratio	1/[S]
40	1	0.82	0.450549451	0.000006	540	0.011111111	2.219512195	90
50	1	1.02	0.504950495	0.0000075	550	0.013636364	1.980392157	73.33333333
60	1	1.38	0.579831933	0.000009	560	0.016071429	1.724637681	62.22222222
70	1	1.5	0.6	0.0000105	570	0.018421053	1.666666667	54.28571429
80	1	1.8	0.642857143	0.000012	580	0.020689655	1.555555556	48.33333333
90	1	1.93	0.658703072	0.0000135	590	0.022881356	1.518134715	43.7037037
100	1	2.03	0.669966997	0.000015	600	0.025	1.492610837	40
120	1	2.32	0.698795181	0.000018	620	0.029032258	1.431034483	34.44444444
140	1	2.4	0.705882353	0.000021	640	0.0328125	1.416666667	30.47619048

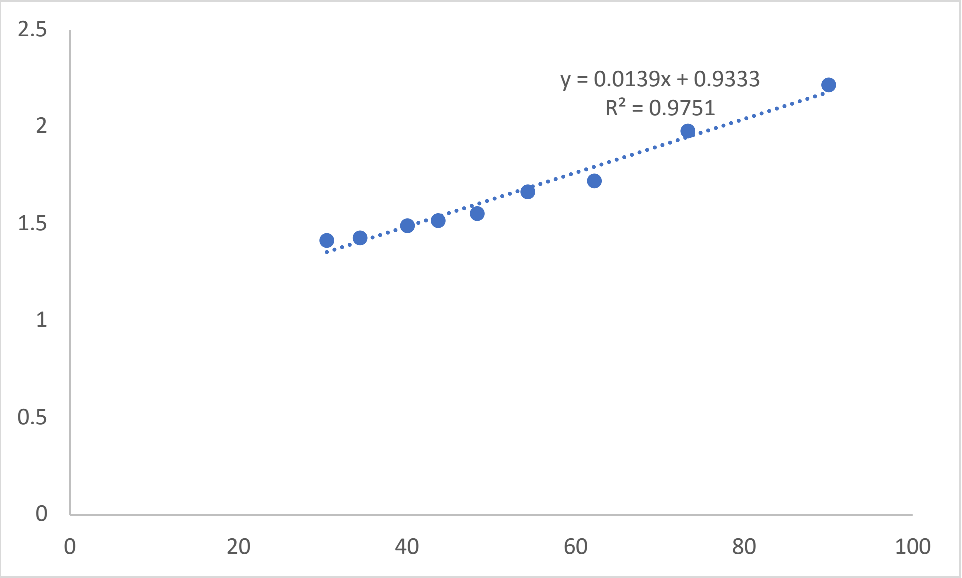

Figure S3: Benesi-Hildebrand plot of the phenylboronic acid-fructose complex.

Table S4: Phenylboronic acid guanosine titration.

volume guan solution added	Int free PhB	int complexed PhB	ratio	Amount [S] added	total volume	[S]	1/ratio	1/[S]
40	1	0.63	0.386503067	0.000006	540	0.011111111	2.587301587	90
50	1	0.77	0.435028249	0.0000075	550	0.013636364	2.298701299	73.33333333
60	1	0.89	0.470899471	0.000009	560	0.016071429	2.123595506	62.22222222
70	1	1.22	0.54954955	0.0000105	570	0.018421053	1.819672131	54.28571429
80	1	1.68	0.626865672	0.000012	580	0.020689655	1.595238095	48.33333333
90	1	2.07	0.674267101	0.0000135	590	0.022881356	1.483091787	43.7037037
100	1	1.66	0.62406015	0.000015	600	0.025	1.602409639	40
120	1	2.66	0.726775956	0.000018	620	0.029032258	1.37593985	34.44444444
140	1	3.28	0.76635514	0.000021	640	0.0328125	1.304878049	30.47619048

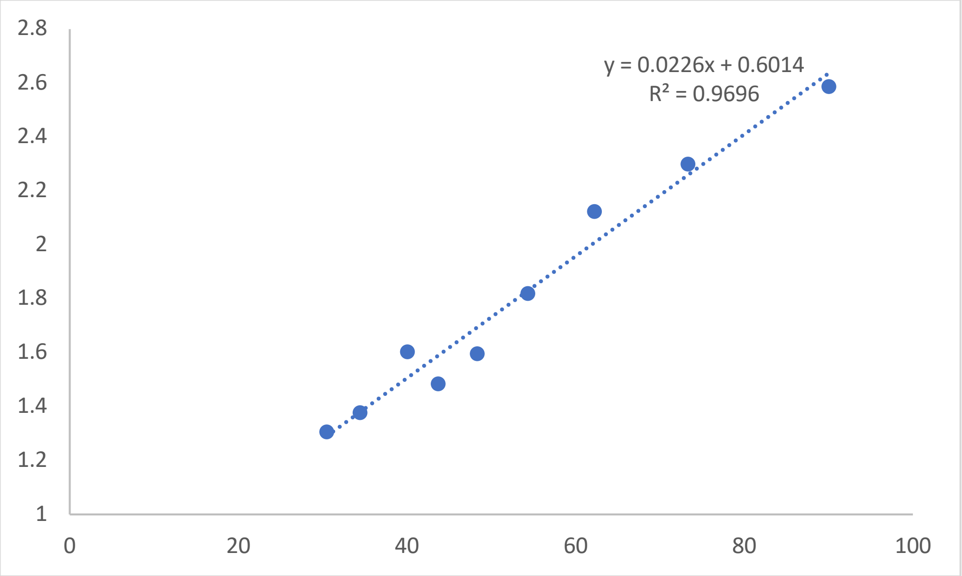

Figure S4: Benesi-Hildebrand plot of the phenylboronic acid-guanosine complex.

Table S5: 19-fructose titration.

volume fruct solution added	Int free 19	int complexed 19	ratio	Amount [S] added	total volume	[S]	1/ratio	1/[S]
40	1	0.08	0.074074074	0.000006	740	0.008108108	13.5	123.3333333
50	1	0.12	0.107142857	0.0000075	750	0.01	9.333333333	100
60	1	0.25	0.2	0.000009	760	0.011842105	5	84.44444444
70	1	0.38	0.275362319	0.0000105	770	0.013636364	3.631578947	73.33333333
80	1	0.47	0.319727891	0.000012	780	0.015384615	3.127659574	65
90	1	0.64	0.390243902	0.0000135	790	0.017088608	2.5625	58.51851852
100	1	0.7	0.411764706	0.000015	800	0.01875	2.428571429	53.33333333
120	1	1	0.5	0.000018	820	0.02195122	2	45.55555556
140	1	1.26	0.557522124	0.000021	840	0.025	1.793650794	40

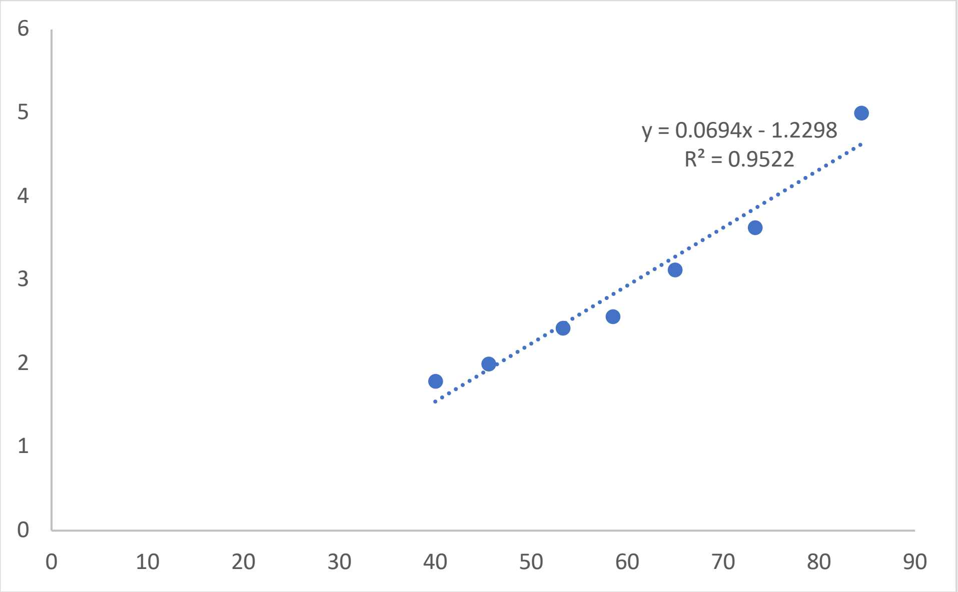

Figure S5: Benesi-Hildebrand plot of the **19**-fructose complex.

Table S6: 19-guanosine titration.

volume guan solution added	Int free 19	int complexed 19	ratio	Amount [S] added	total volume	[S]	1/ratio	1/[S]
40	1	0.67	0.401197605	0.000006	740	0.008108108	2.492537313	123.3333333
50	1	0.86	0.462365591	0.0000075	750	0.01	2.162790698	100
60	1	1.07	0.516908213	0.000009	760	0.011842105	1.934579439	84.44444444
70	1	1.21	0.547511312	0.0000105	770	0.013636364	1.826446281	73.33333333
80	1	1.37	0.578059072	0.000012	780	0.015384615	1.729927007	65
90	1	1.61	0.616858238	0.0000135	790	0.017088608	1.621118012	58.51851852
100	1	1.86	0.65034965	0.000015	800	0.01875	1.537634409	53.33333333
120	1	2.21	0.68847352	0.000018	820	0.02195122	1.452488688	45.55555556
140	1	2.71	0.730458221	0.000021	840	0.025	1.36900369	40

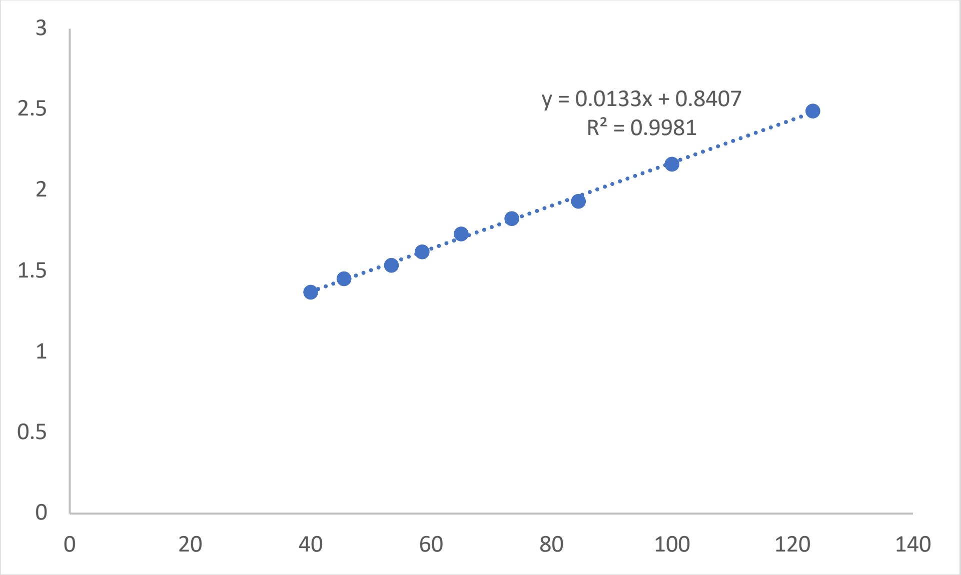

Figure S6: Benesi-Hildebrand plot of the **19**-guanosine complex.

5. Optimization Data

Table S7: Optimization data.

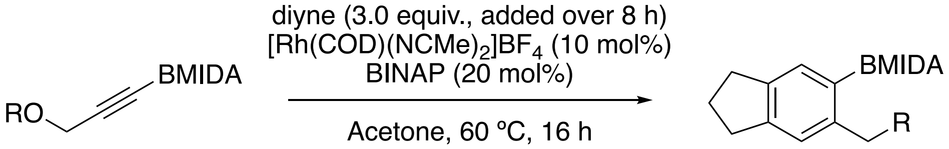

**Entry**	**R**	**Deviation from standard**	**NMR yield**[a]	**RTO**
**1**	H	-	73	7.3
**2**	H	15 mol% Rh, 30 mol% BINAP, 6.0 equiv. diyne, 16 h addn	86	5.7
**3**	H	10 mol% Rh, 20 mol% BINAP, 6.0 equiv. diyne, 16 h addn	81	8.1
**4**	H	5 mol% Rh, 10 mol% BINAP, 6.0 equiv. diyne, 16 h addn	69	13.8
**5**	H	20 mol% Rh, 40 mol% BINAP, 6.0 equiv. diyne, 16 h addn	>99	≥5.0
**6**	H	20 mol% Rh, 40 mol% BINAP, 3.0 equiv. diyne, 16 h addn	90	4.5
**6**	TBS	20 mol% Rh, 40 mol% BINAP, 6.0 equiv. diyne, 16 h addn	68	3.4
**7**	TBS	15 mol% Rh, 30 mol% BINAP, 6.0 equiv. diyne, 16 h addn	50	3.3
**8**	TBS	10 mol% Rh, 20 mol% BINAP, 6.0 equiv. diyne, 16 h addn	25	2.5
**9**	TBS	5 mol% Rh, 10 mol% BINAP, 6.0 equiv. diyne, 16 h addn	18	3.6
**10**	TBS	No Rh, No ligand	0	0
**11**	TBS	No slow addn	<5	<0.25

[a]Determined using 1,4-dinitrobenzene (4.2 mg, 50 µmol, 0.50 equiv.) as an internal standard.

6. Chelation Data

Table S8: Chelation data of various alkynyl BMIDAs.

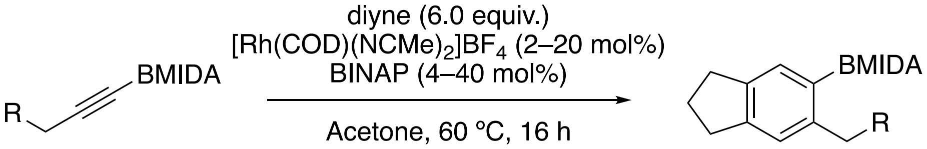

**Entry**	**R**	**Rh loading (mol%)**	**NMR yield**[a]	**RTO**
**1**	H	20	68	3.40
**2**	H	15	50	3.33
**3**	H	10	25	2.50
**4**	H	5	18	3.60
**5**	H	2	6	3.00
**6**	OTBS	20	50	2.50
**7**	OTBS	15	45	3.00
**8**	OTBS	10	39	3.90
**9**	OTBS	5	30	6.00
**10**	OTBS	2	24	12.0
**11**	OH	20	99	4.95
**12**	OH	15	86	5.73
**13**	OH	10	81	8.10
**14**	OH	5	69	13.80
**15**	OH	2	37	18.50

[a]Determined using 1,4-dinitrobenzene (4.2 mg, 50 µmol, 0.50 equiv.) as an internal standard.

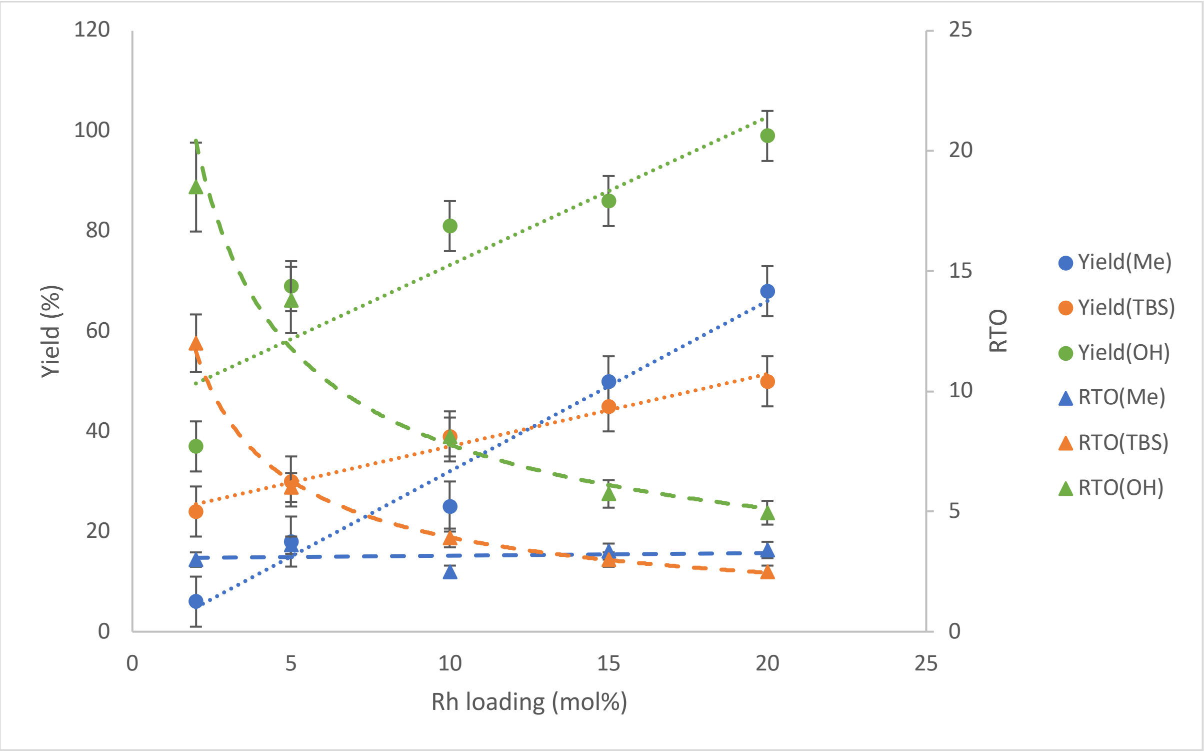

Figure S7: Graph of yield and RTO *vs.* Rh loading (errors bars refer to standardized 5% error).

7. X-ray Diffraction Data

X-ray diffraction data for compounds **4**, **16**, and **27** were collected at 173 K or 125 K using a Rigaku FR-X Ultrahigh Brilliance Microfocus RA generator/confocal optics with XtaLAB P200 diffractometer [Mo Kα radiation (λ = 0.71073 Å)]. Data for all compounds analyzed were collected (using a calculated strategy) and processed (including correction for Lorentz, polarization and absorption) using CrysAlisPro.[32] Structures were solved by either direct methods (SIR2011[33]) or dual-space methods (SHELXT[34]) and refined by full-matrix least-squares against F^2 (SHELXL-2019/3[35]). Non-hydrogen atoms were refined anisotropically, and hydrogen atoms were refined using a riding model except for the hydrogen atoms on O1 in **4** and **27** which were located from the difference Fourier map and refined isotropically subject to a distance restraint. Crystals of **16** were affected by non-merohedral twinning showing a twin law of [-1.000 0.000 0.000 -0.007 0.055 -0.945 0.007 -1.055 -0.055] and a refined twin fraction of 0.171. Non-merohedral twinning of **16** was identified using PLATON[36] and all other calculations were performed using the Olex2[37] interface. CCDC 2306555–2306557 contains the supplementary crystallographic data for this paper. These data can be obtained free of charge from The Cambridge Crystallographic Data Centre via www.ccdc.cam.ac.uk/structures. Selected crystallographic data are presented in Table S9.

Table S9: Selected crystallographic data.

	**4**	**16**	**27**
formula	$C_{10}H_{11}BO_2$	$C_9H_9BO_3$	$C_{26}H_{27}BbrNO_5S$
fw	174.00	175.97	556.26
crystal description	Colorless prism	Colorless prism	Colorless block
crystal size [mm^3]	0.11 x 0.03 x 0.01	0.15 x 0.09 x 0.08	0.30 x 0.15 x 0.04
temperature [K]	100	125	125
space group	$P\bar{1}$	$P\bar{1}$	$P2_1/n$
a [Å]	4.6117(3)	8.8417(3)	17.1276(4)
b [Å]	6.0994(3)	9.4609(5)	9.7083(2)
c [Å]	15.6870(8)	9.9015(4)	17.5883(4)
α [°]	98.807(4)	101.067(4)	
β [°]	93.366(4)	99.509(3)	118.782(3)
γ [°]	102.254(5)	98.541(4)	
vol [Å]^3	424.22(4)	787.67(6)	2563.27(12)
Z	2	4	4
ρ (calc) [g/cm^3]	1.362	1.484	1.441
μ [mm^{-1}]	0.091	0.108	1.722
F(000)	184.0	368.0	1144.0
reflections collected	5252	10575	49758
independent reflections (R_{int})	1896 (0.0341)	3686 (0.0362)	6107 (0.0331)
parameters, restraints	122, 1	238, 0	323, 1
GoF on F^2	1.041	1.070	1.059
R_1 [$I > 2\sigma(I)$]s	0.0479	0.0548	0.0357
wR_2 (all data)	0.1288	0.1655	0.0823
largest diff. peak/hole [e/Å^3]	0.34/-0.20	0.46/-0.31	1.00/-0.68

8. NMR Spectra

(S1)

^{1}H

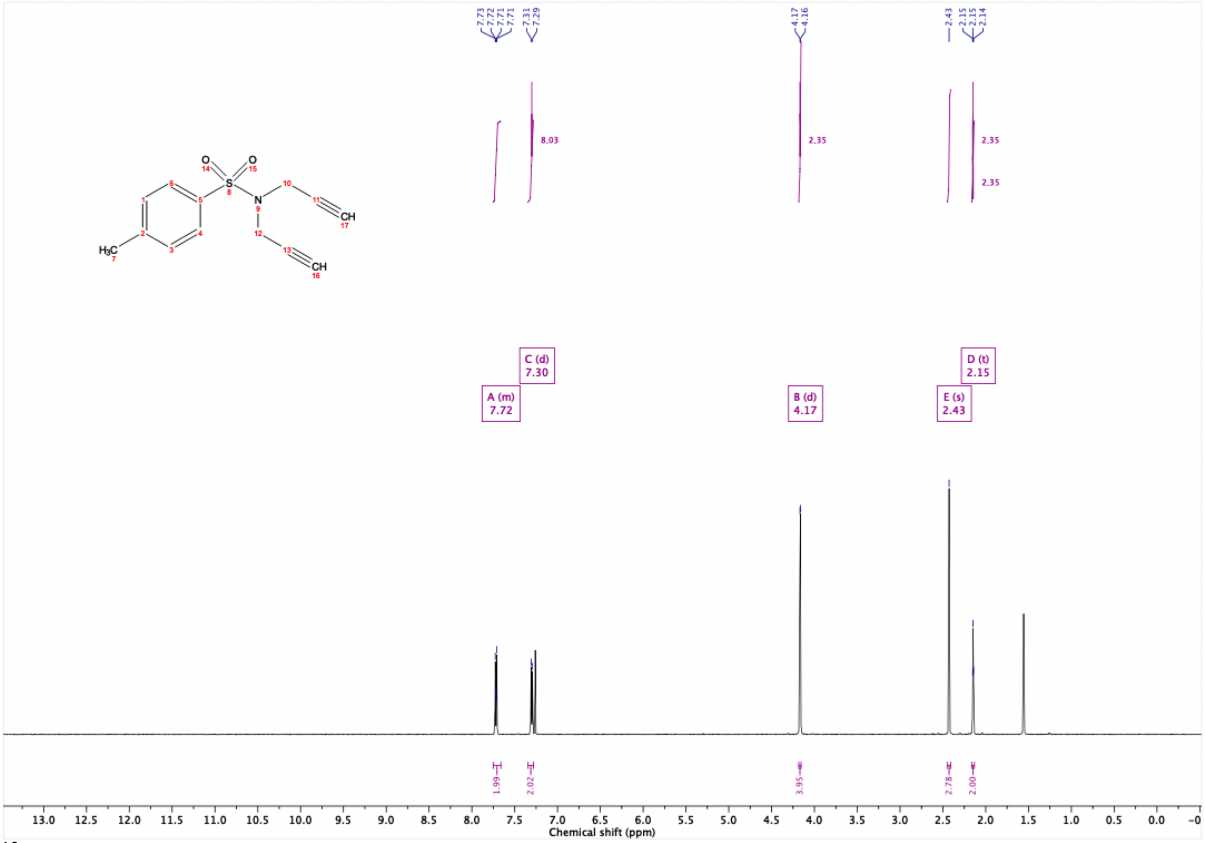

^{13}C DEPTQ

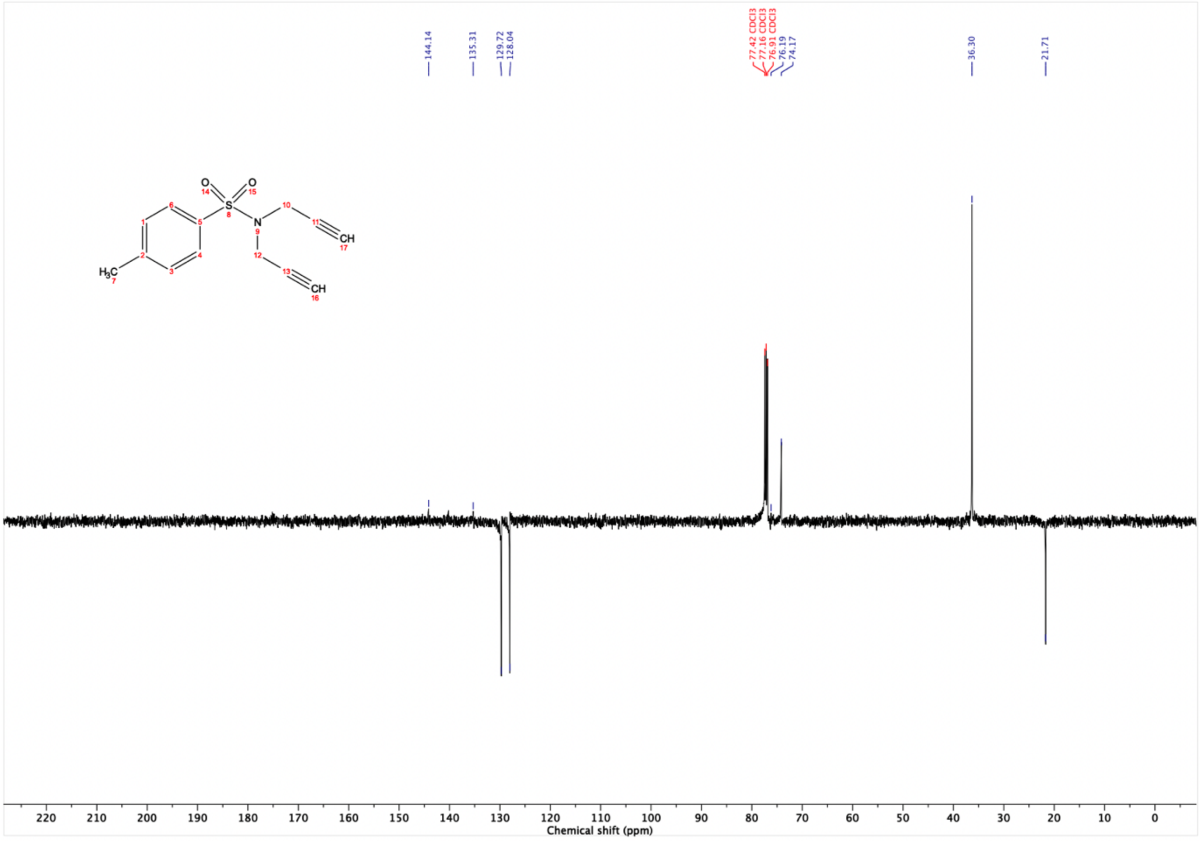

(**S2**)

^{1}H

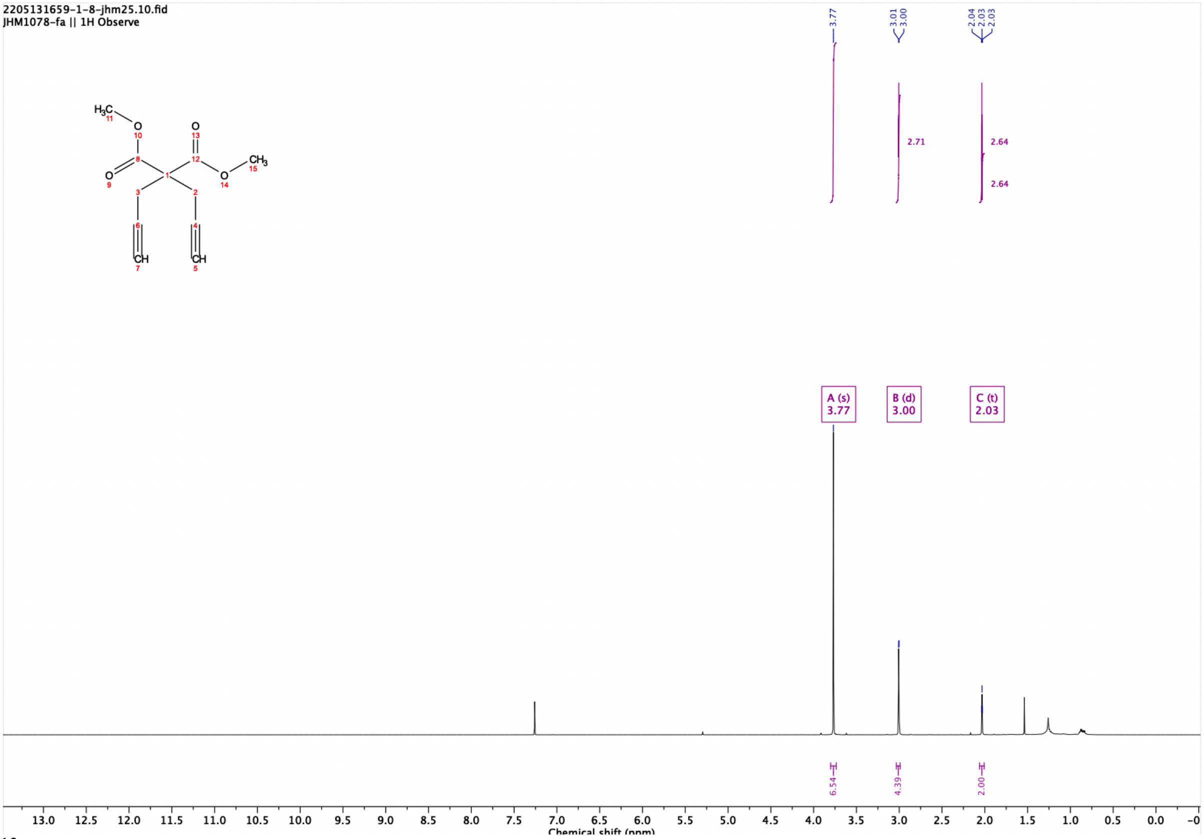

^{13}C DEPTQ

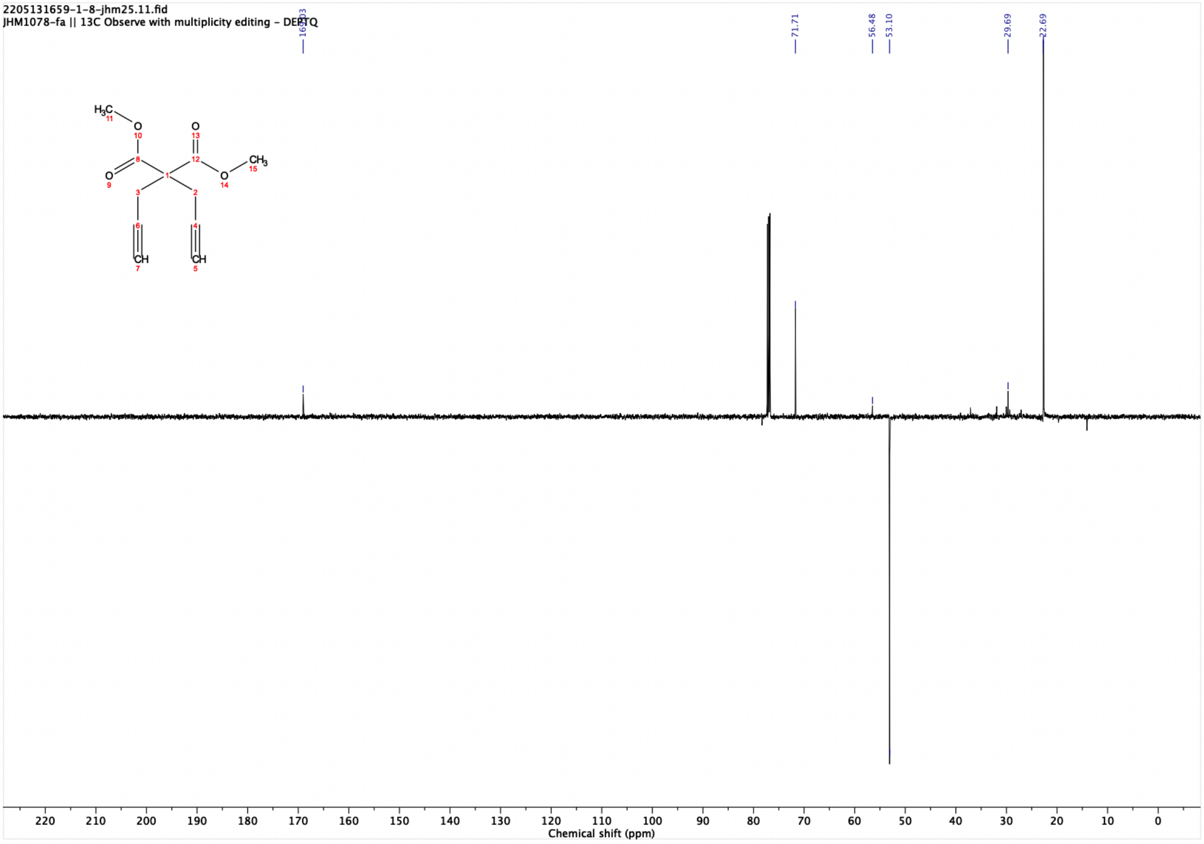

(S3)

^{1}H

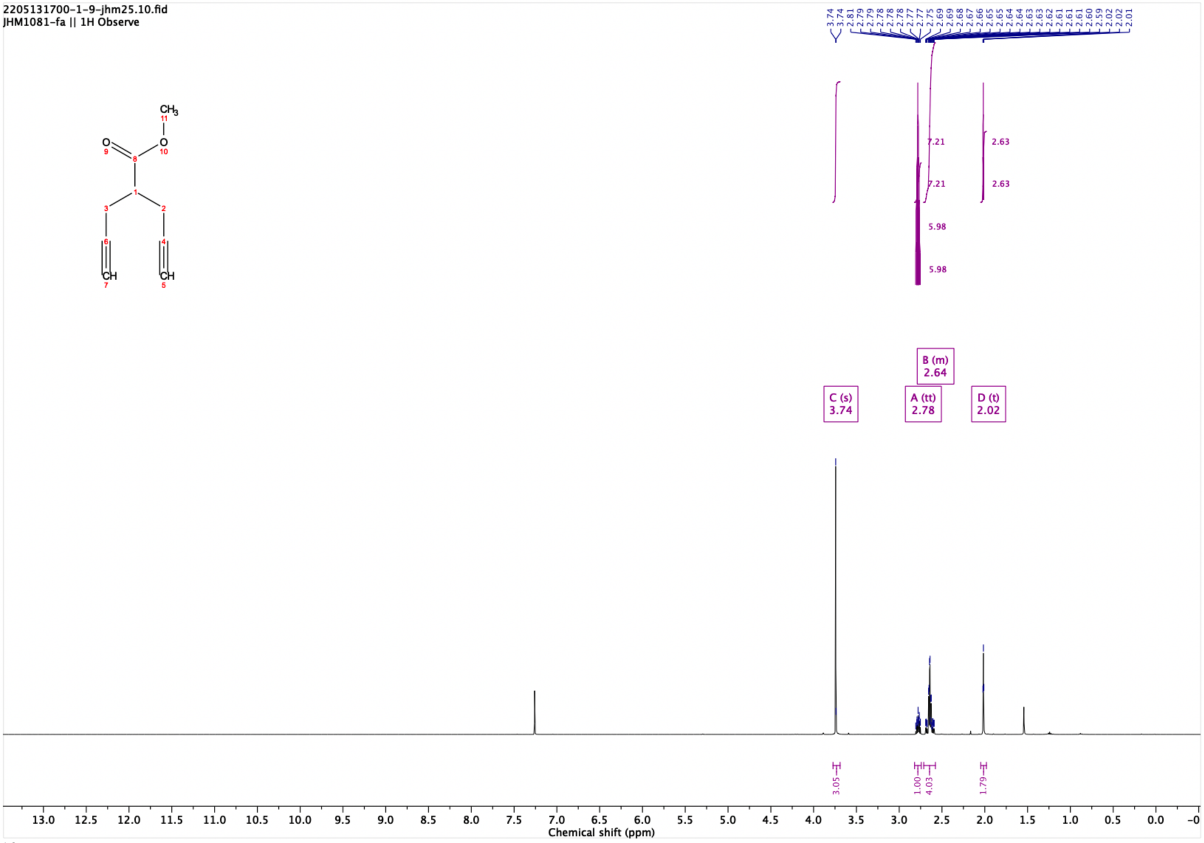

^{13}C DEPTQ

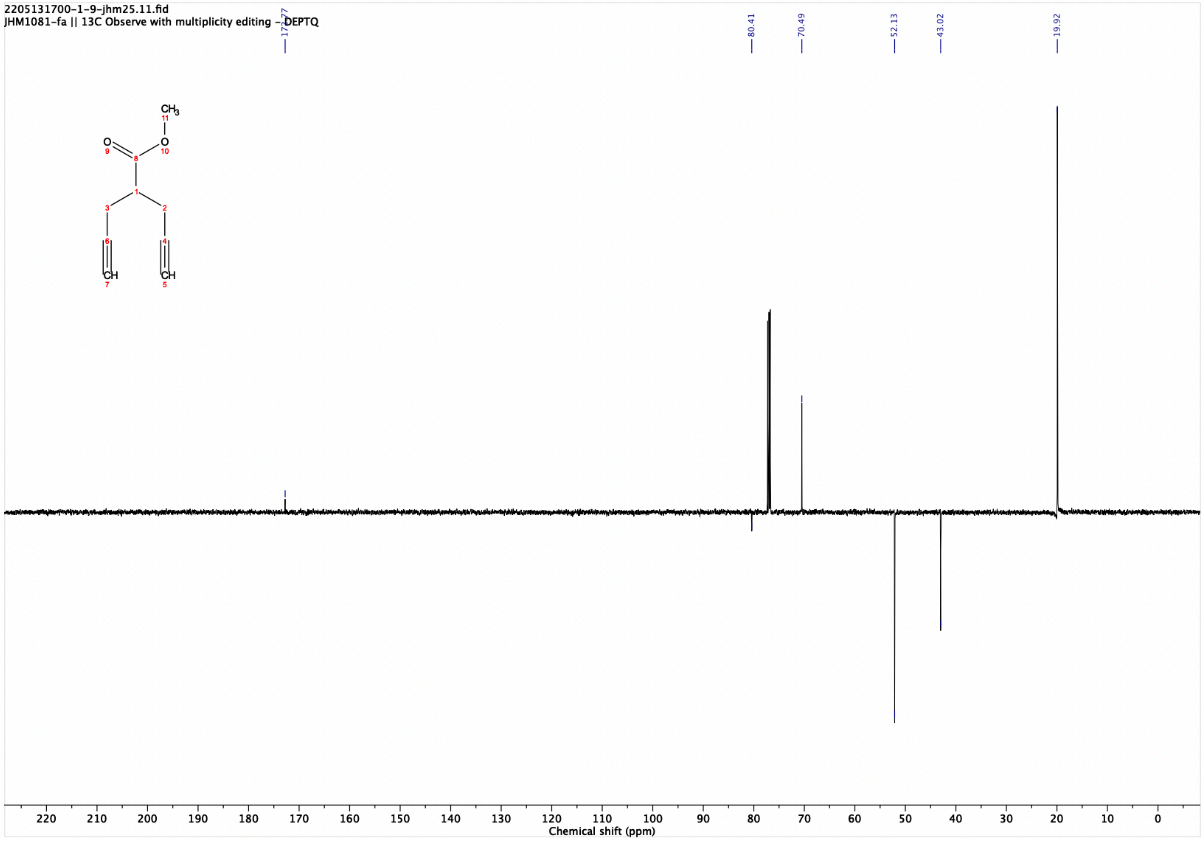

(S4)

^{1}H

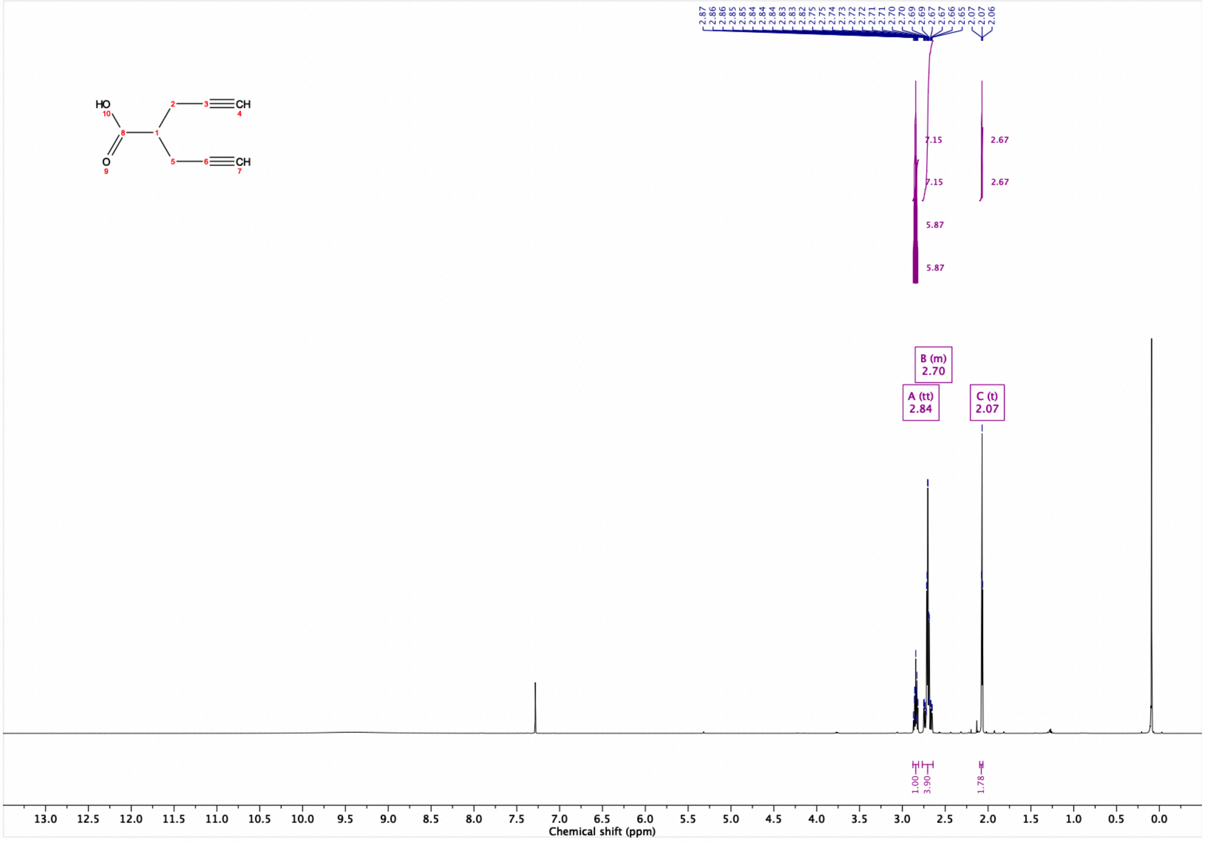

^{13}C DEPTQ

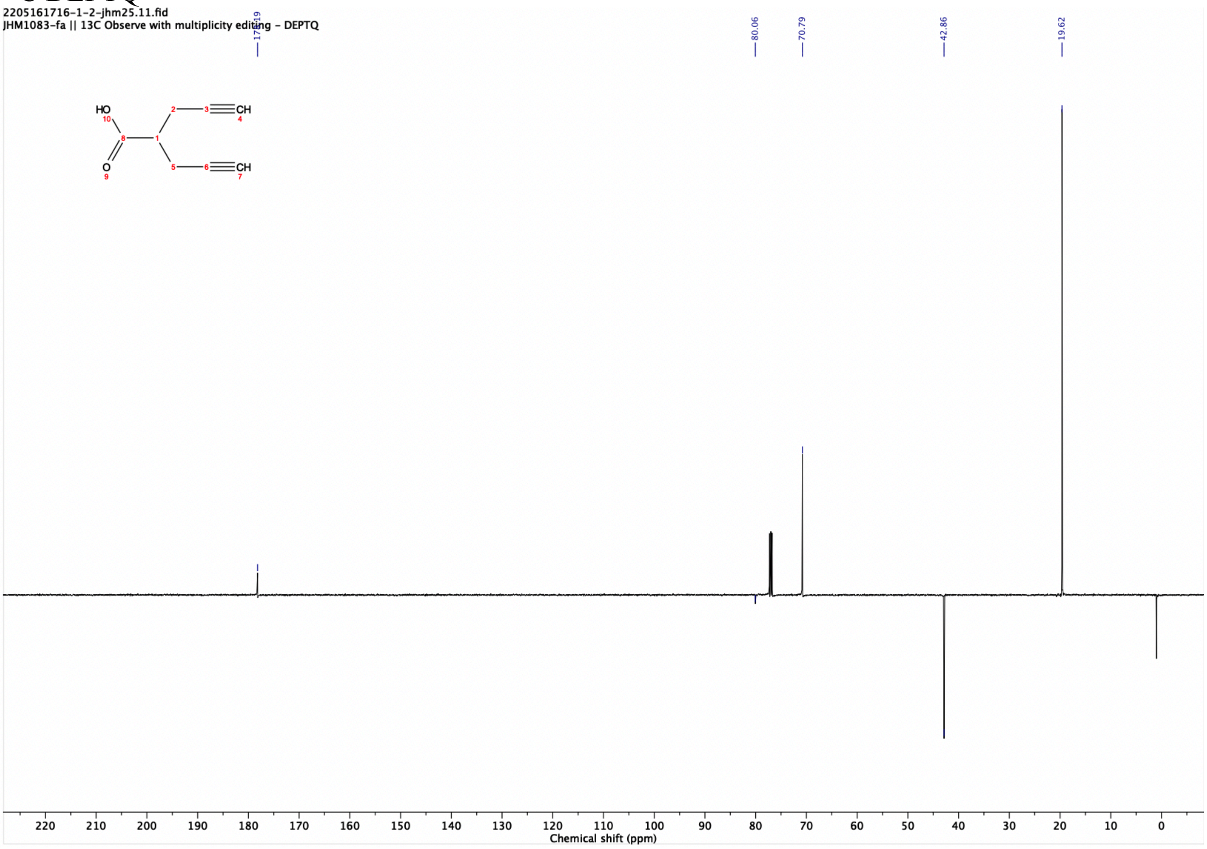

(S5)

^{1}H

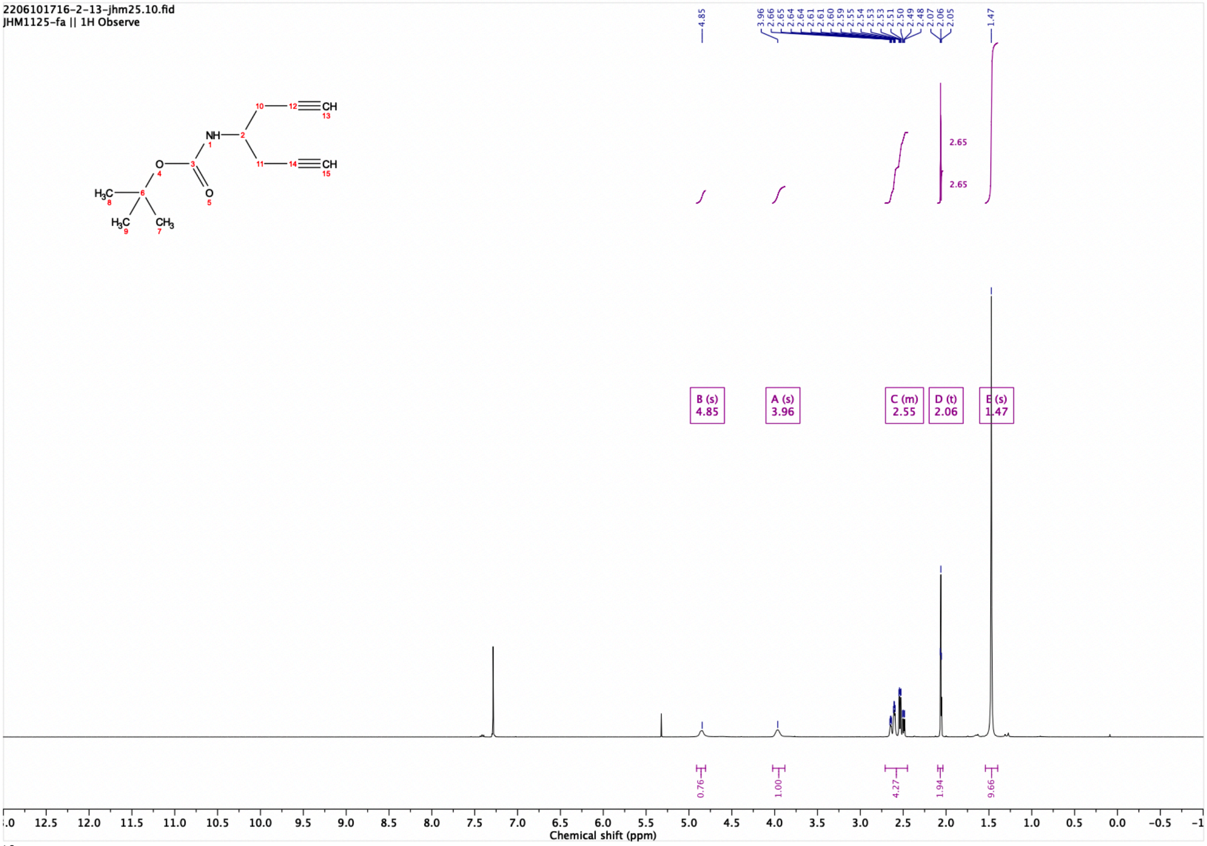

^{13}C DEPTQ

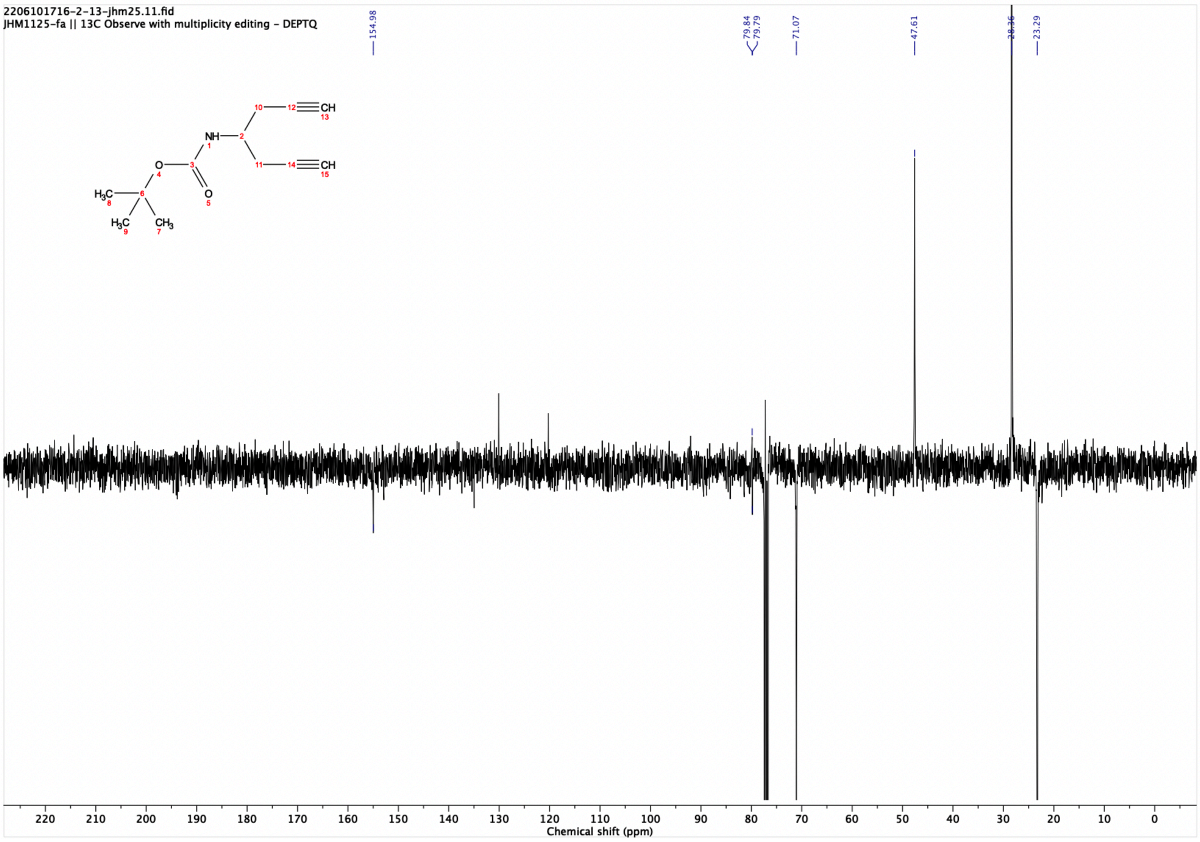

(S6)

^{1}H

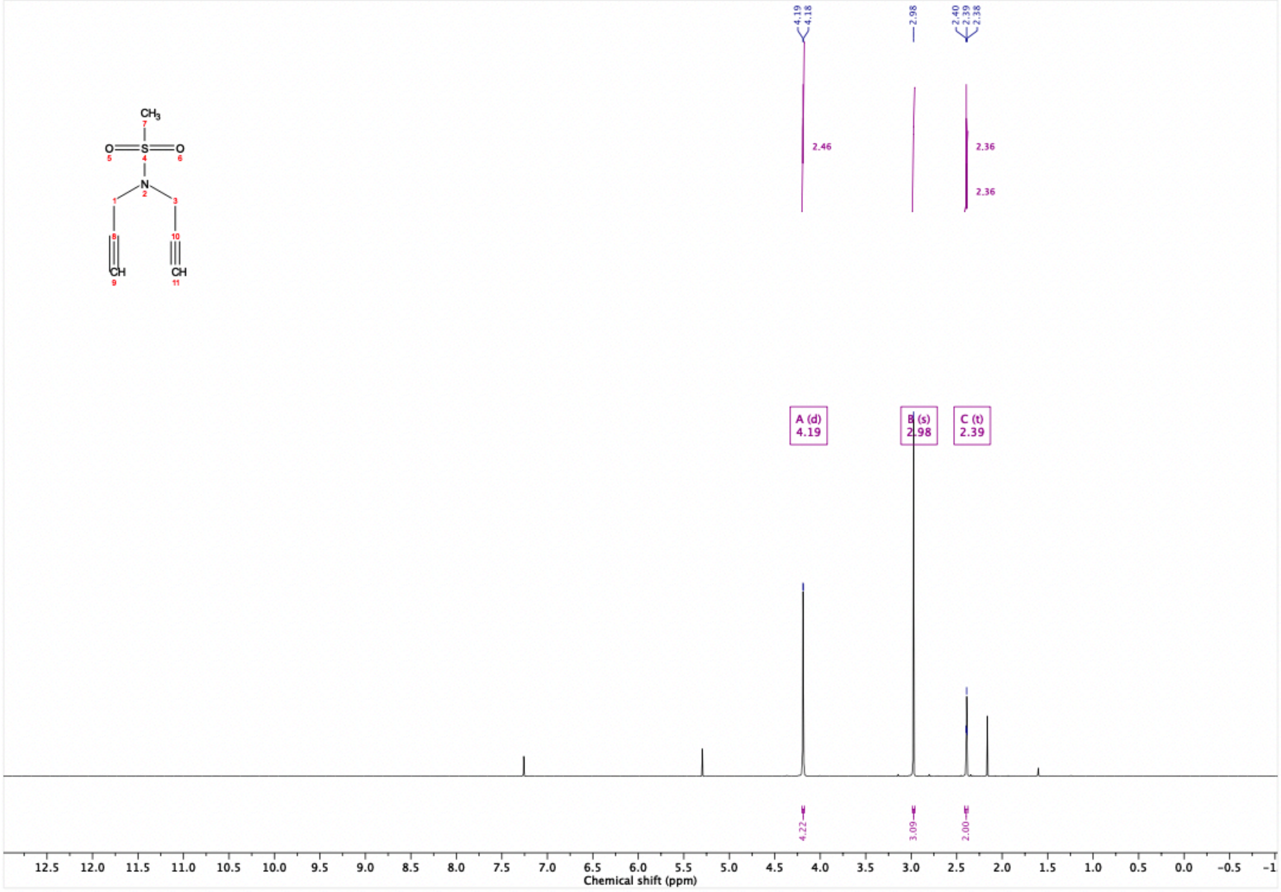

^{13}C DEPTQ

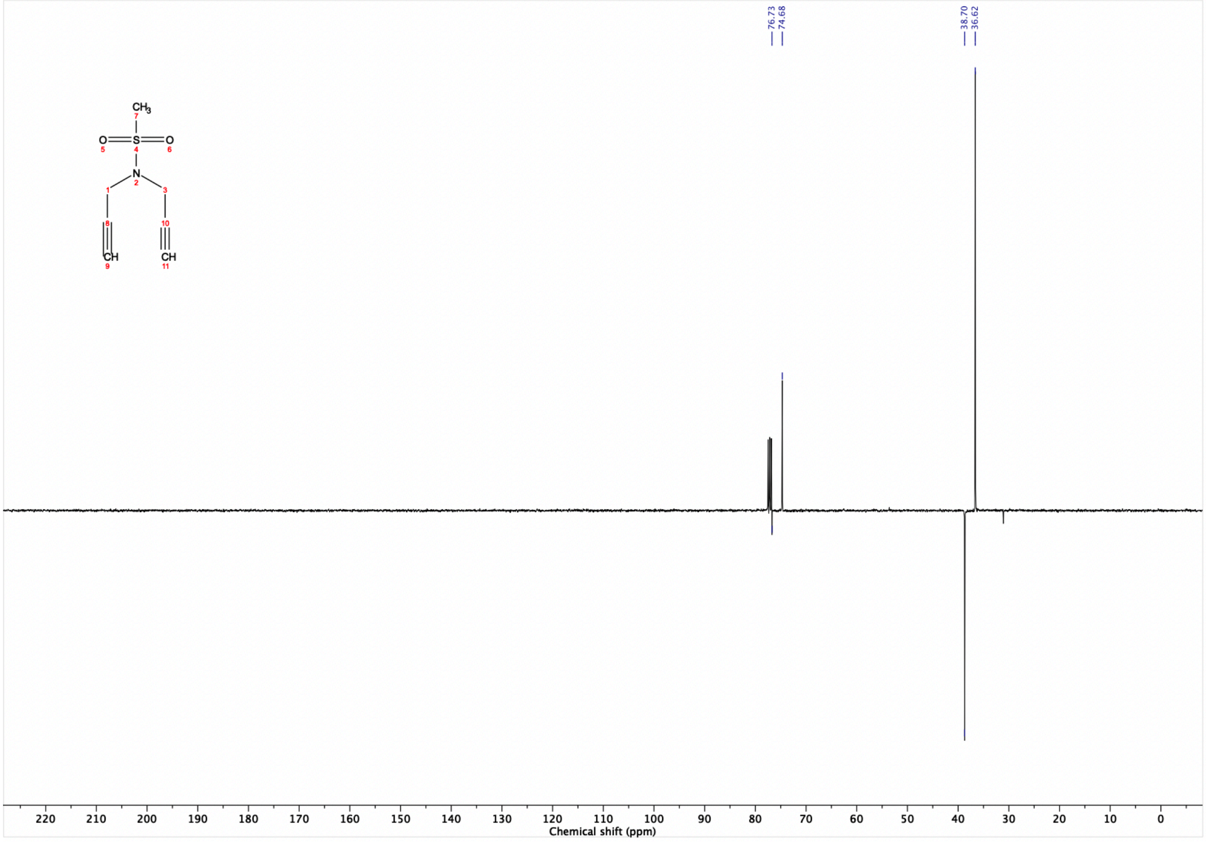

(S7)

^{1}H

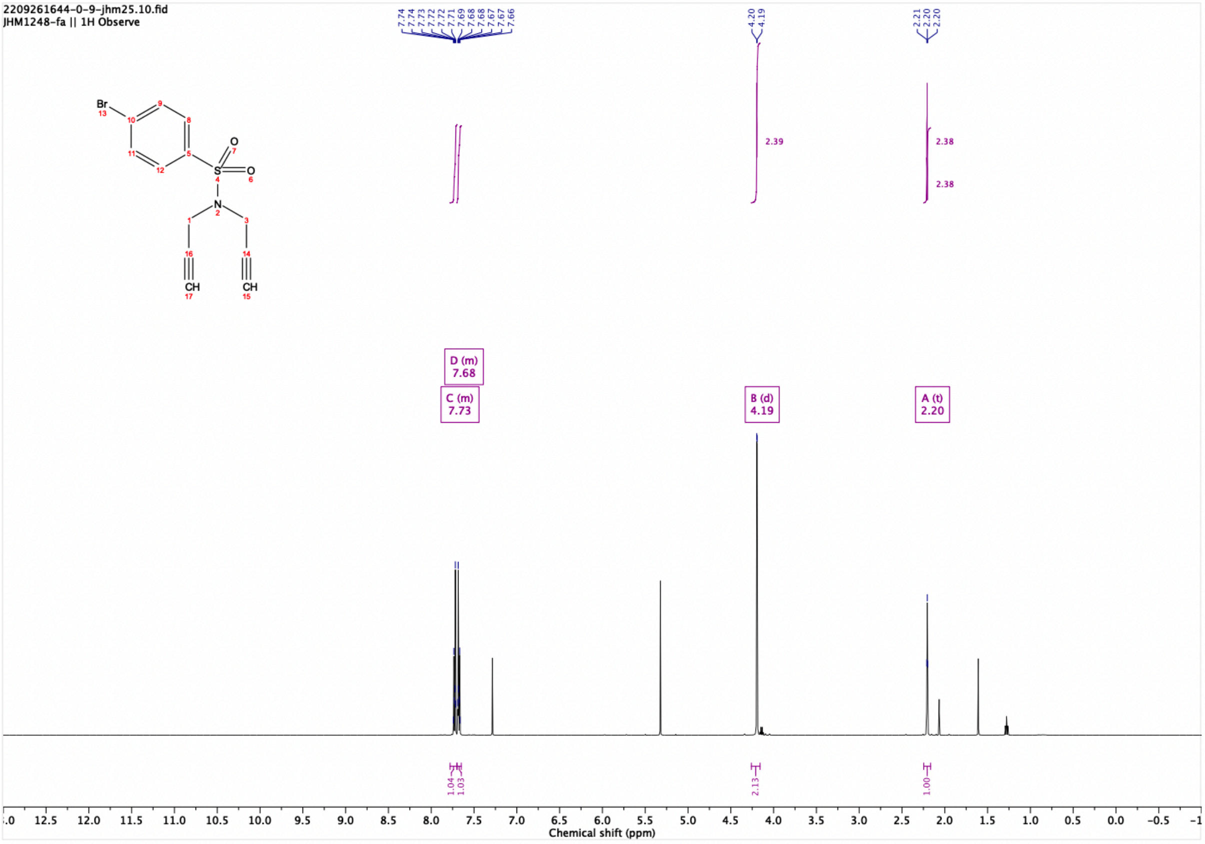

^{13}C DEPTQ

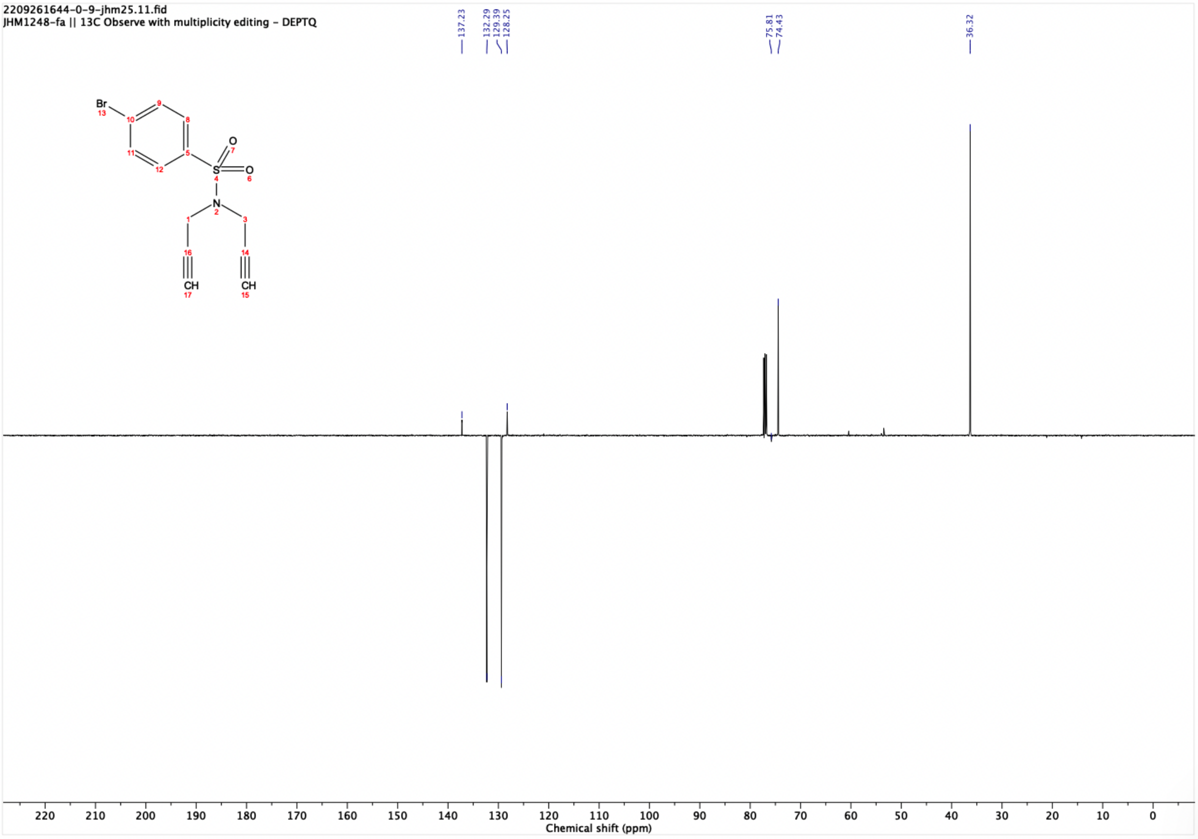

(S8)

^{1}H

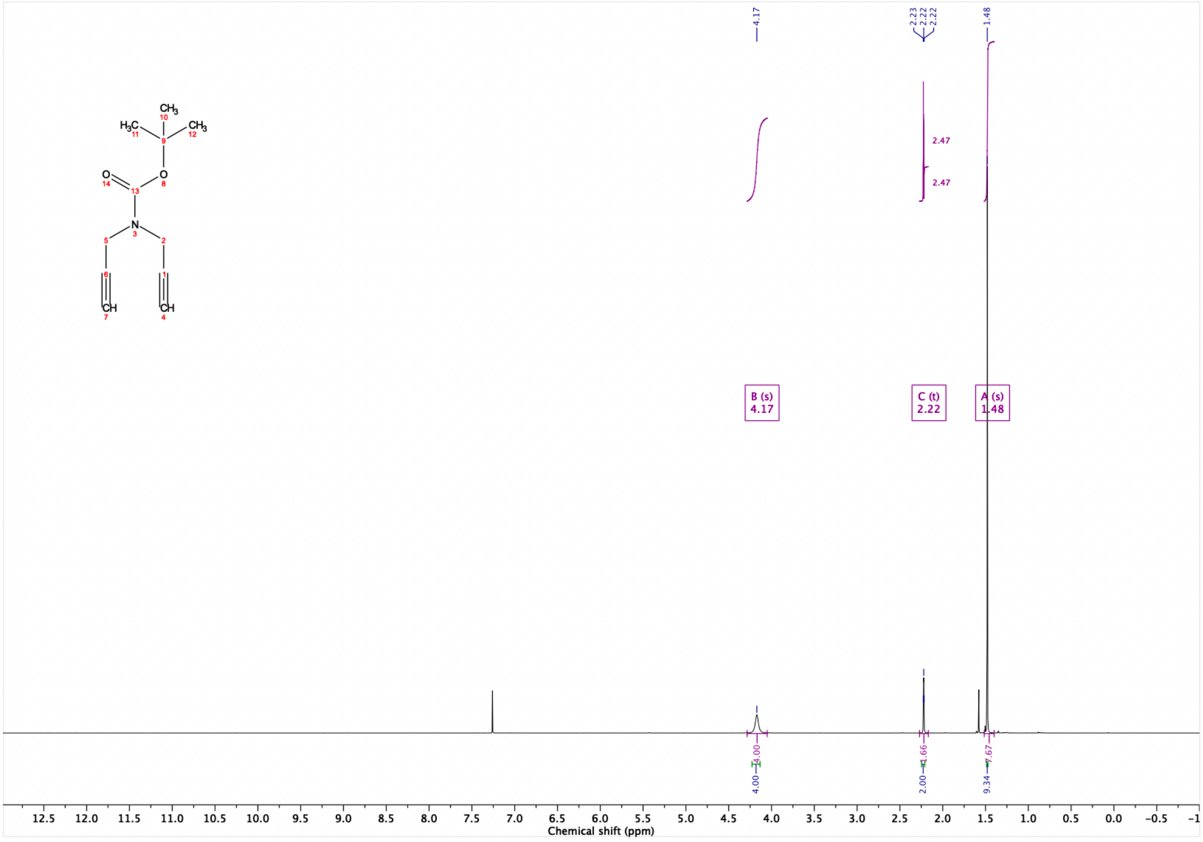

^{13}C DEPTQ

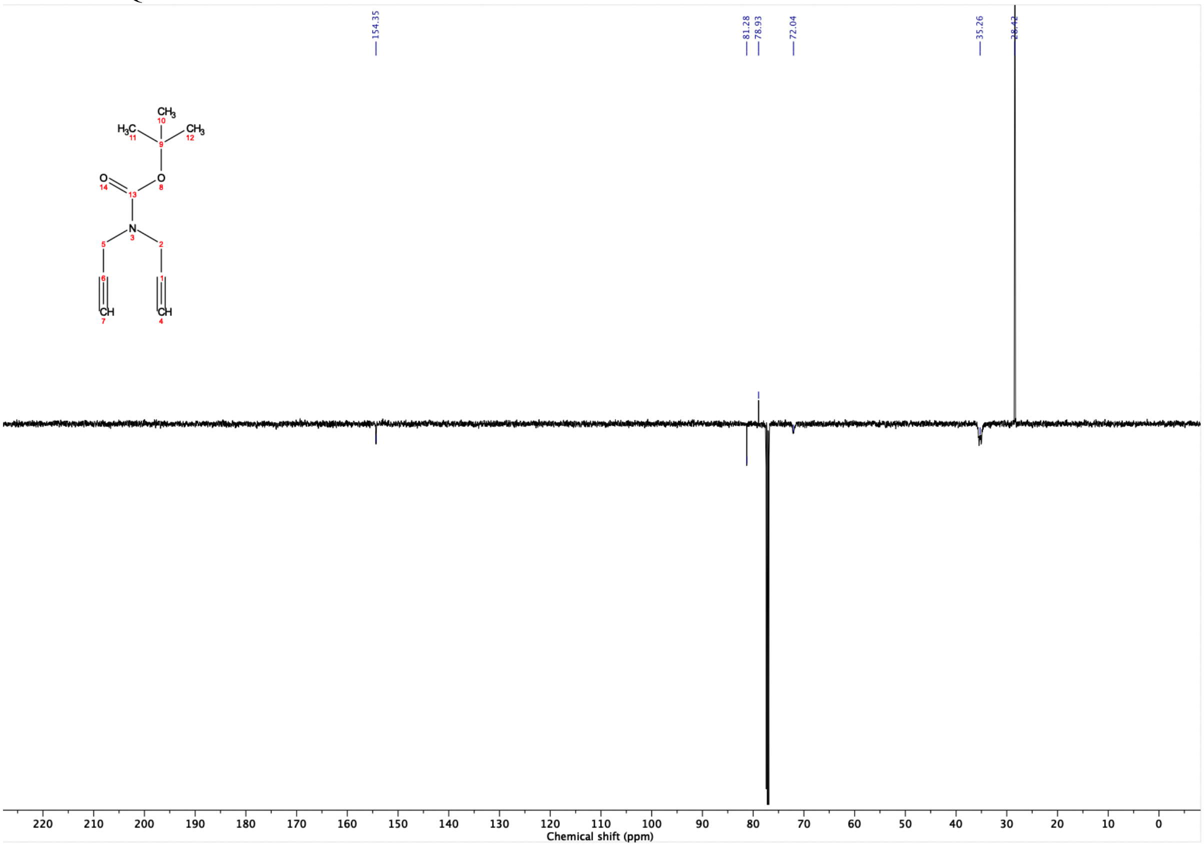

(S9)

^{1}H

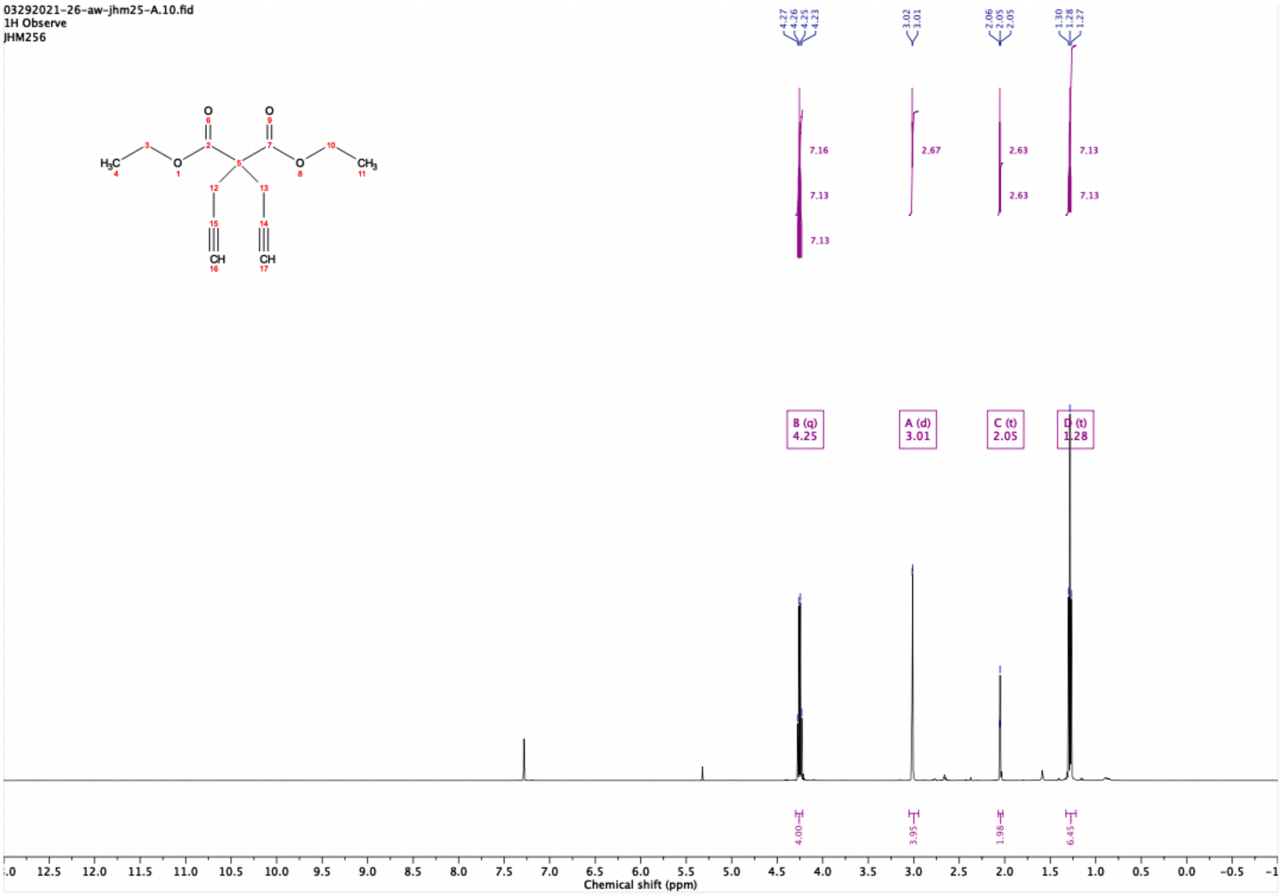

^{13}C DEPTQ

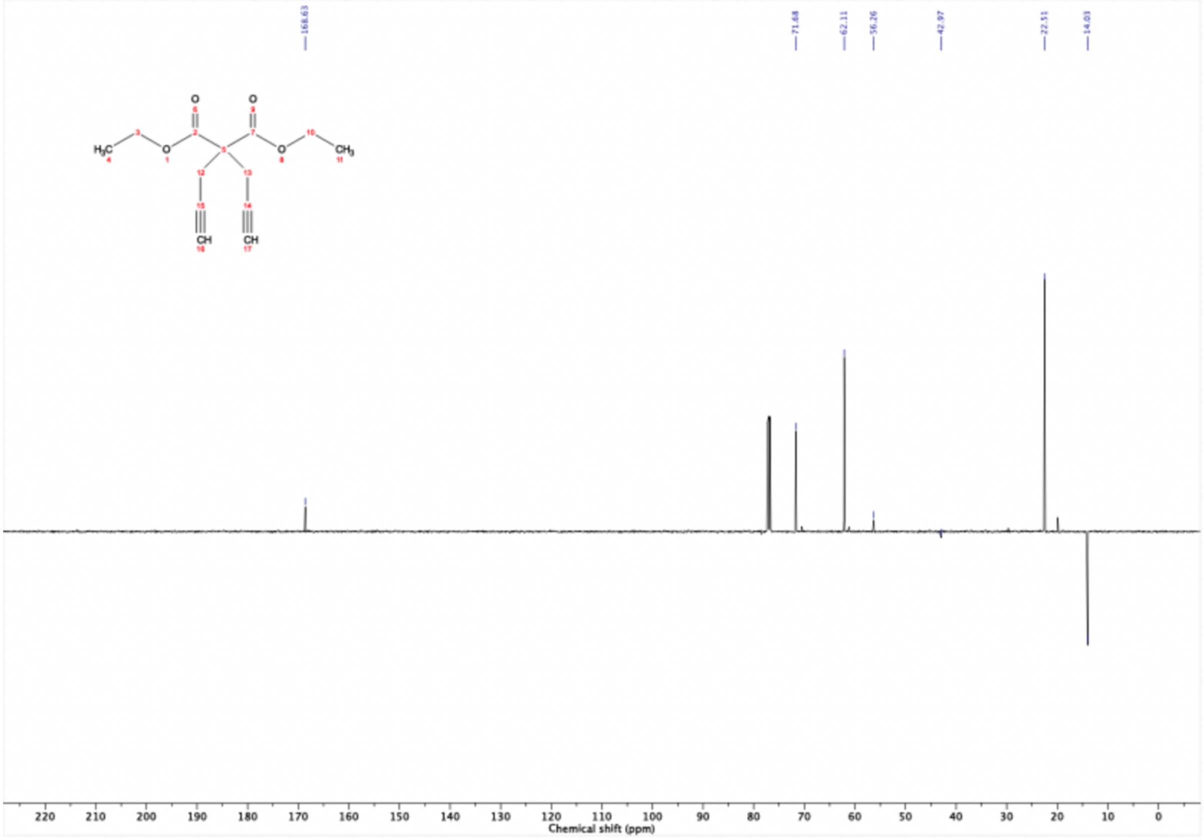

(S10)

^{1}H

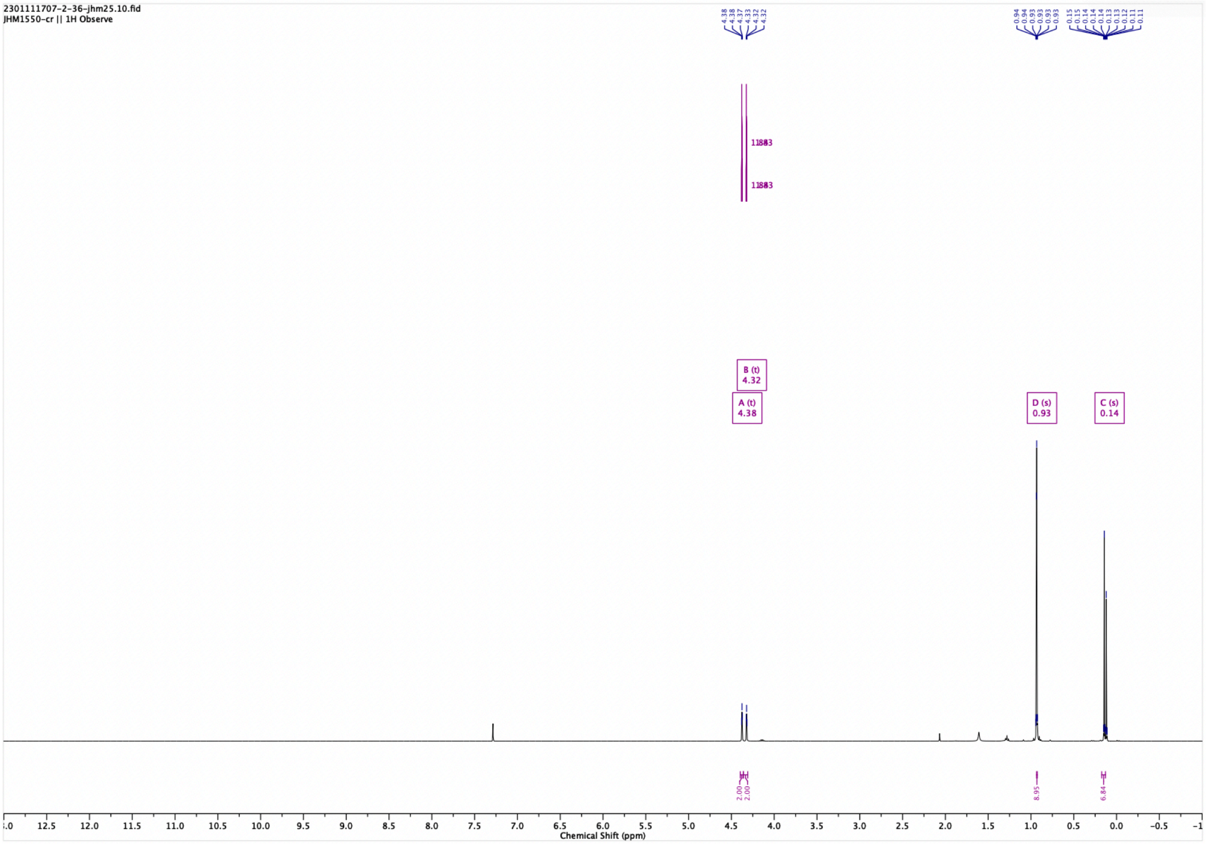

^{13}C DEPTQ

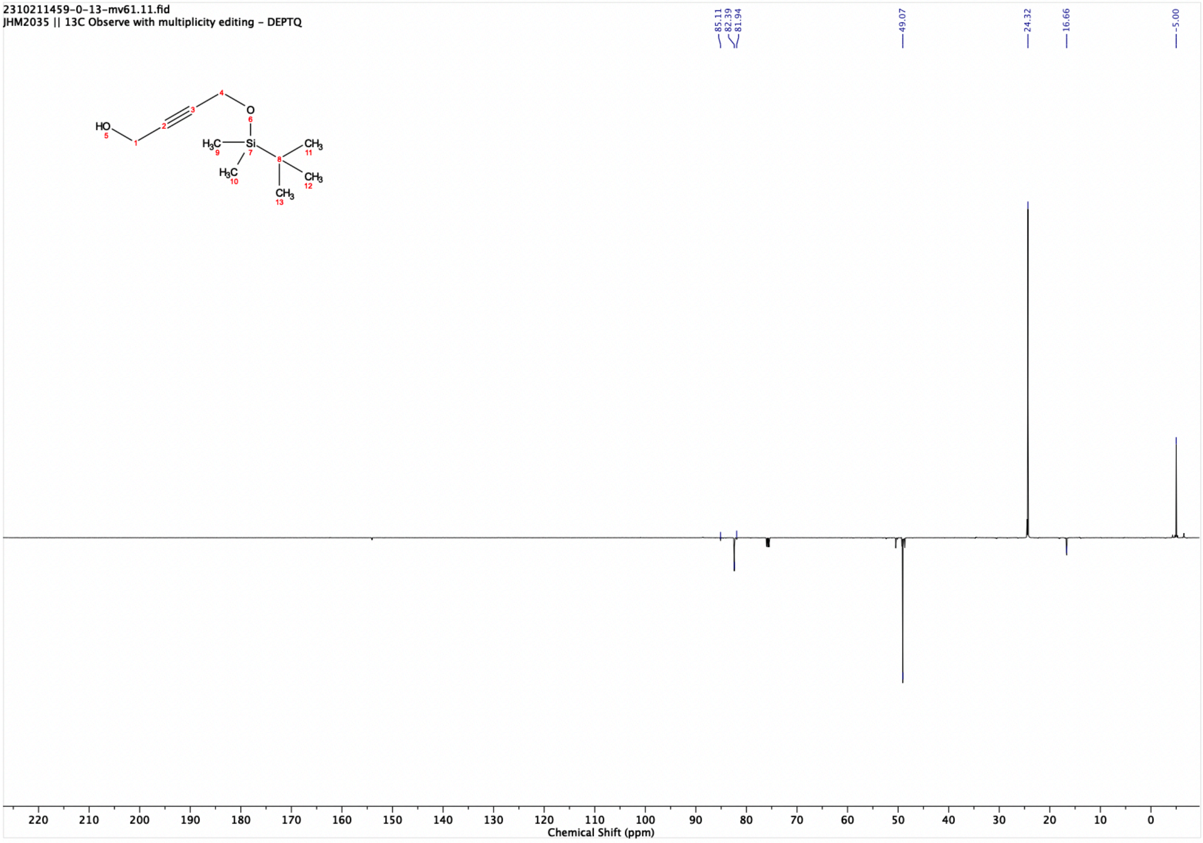

(S11)

^{1}H

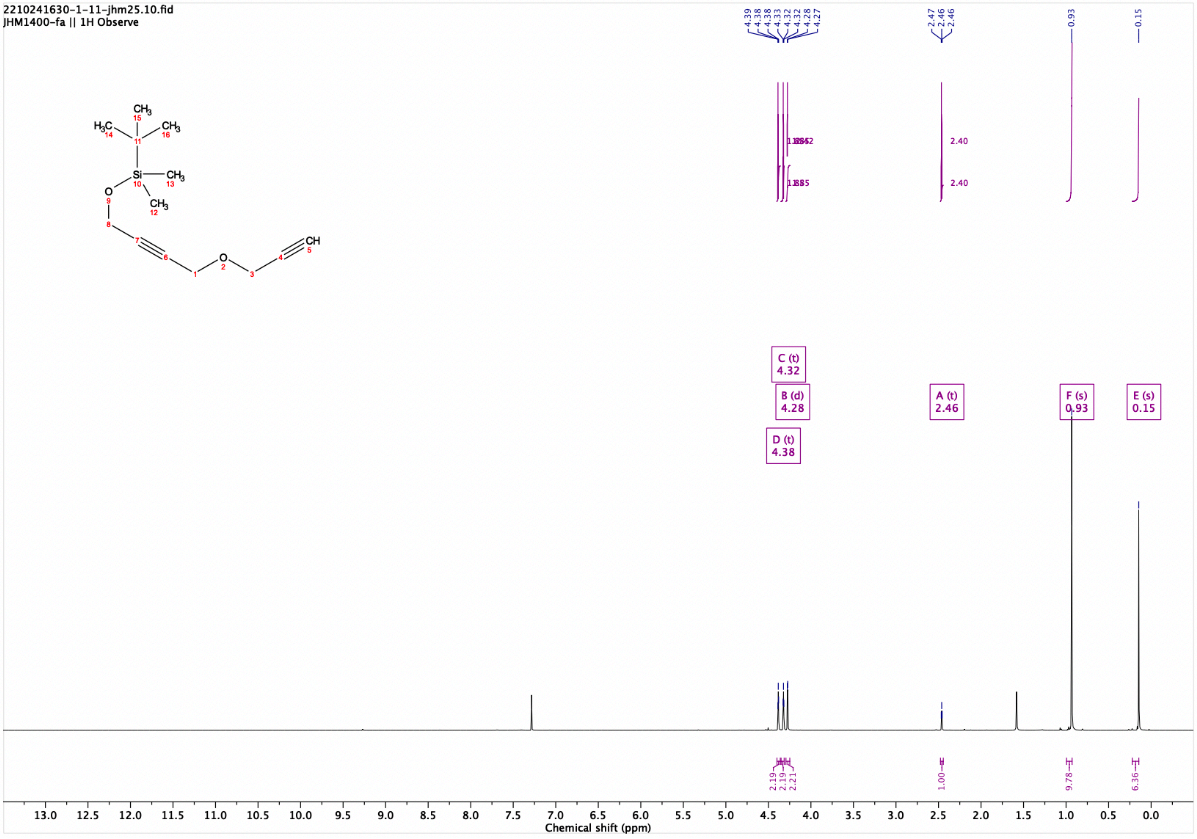

^{13}C DEPTQ

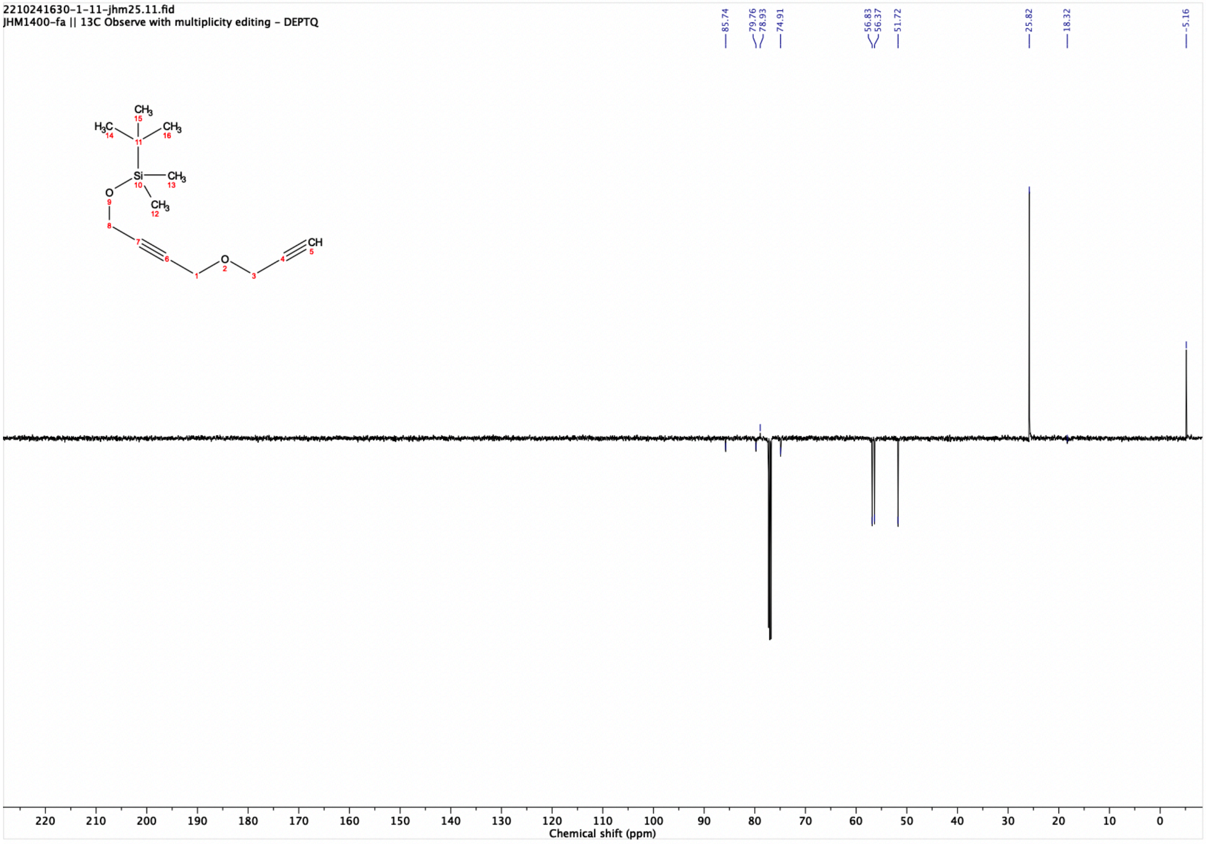

(S12)

^{1}H

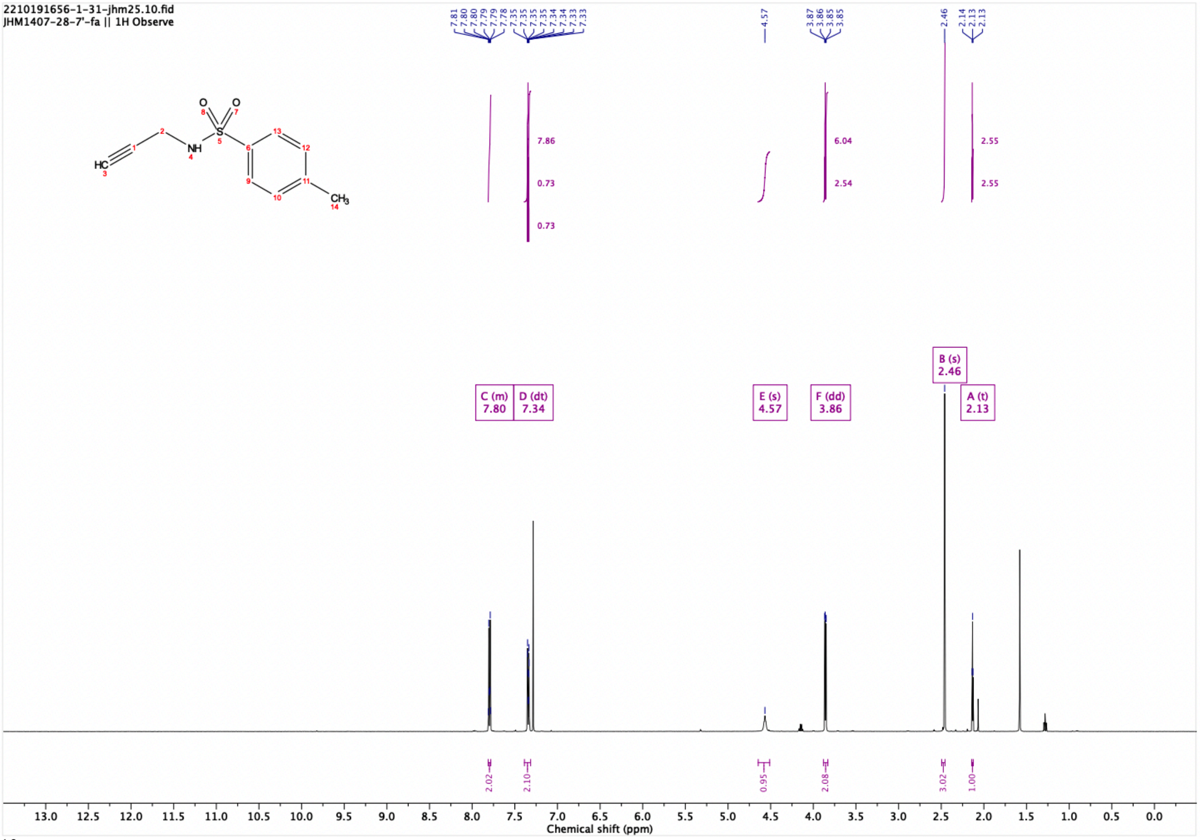

^{13}C DEPTQ

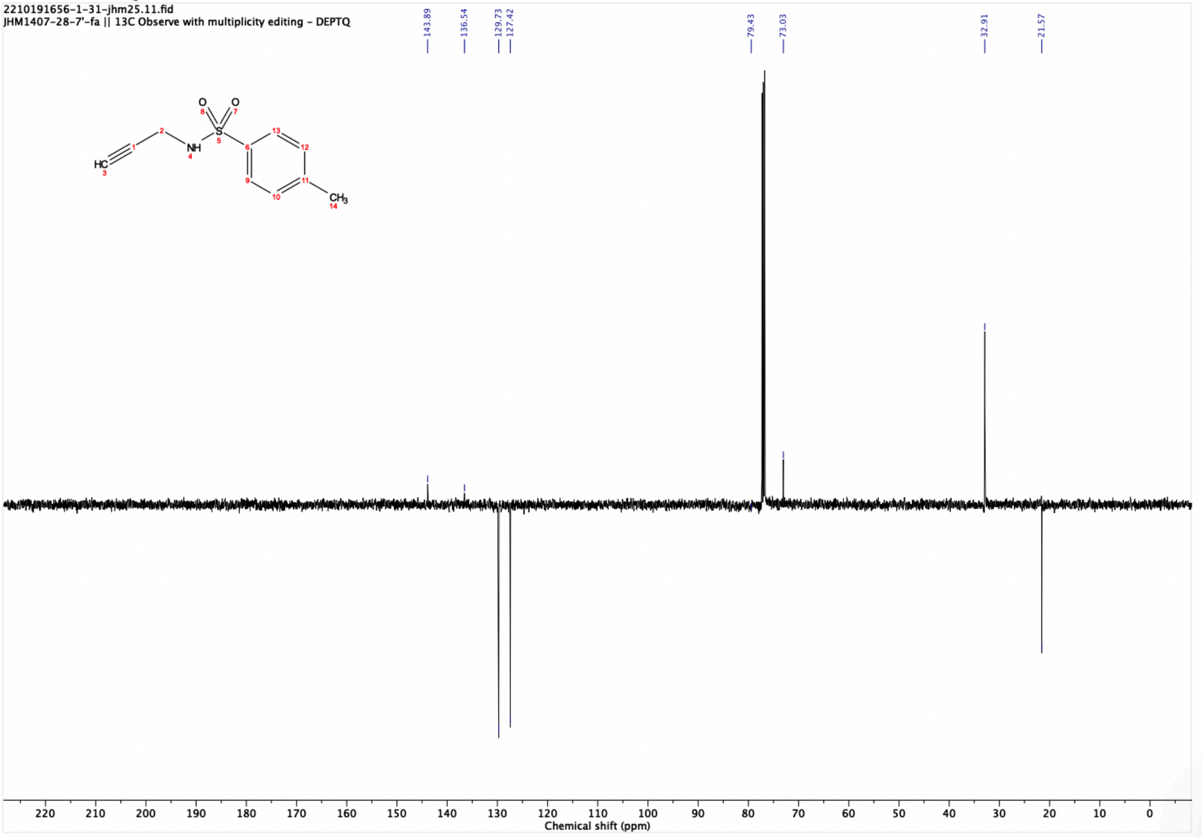

(S13)

^{1}H

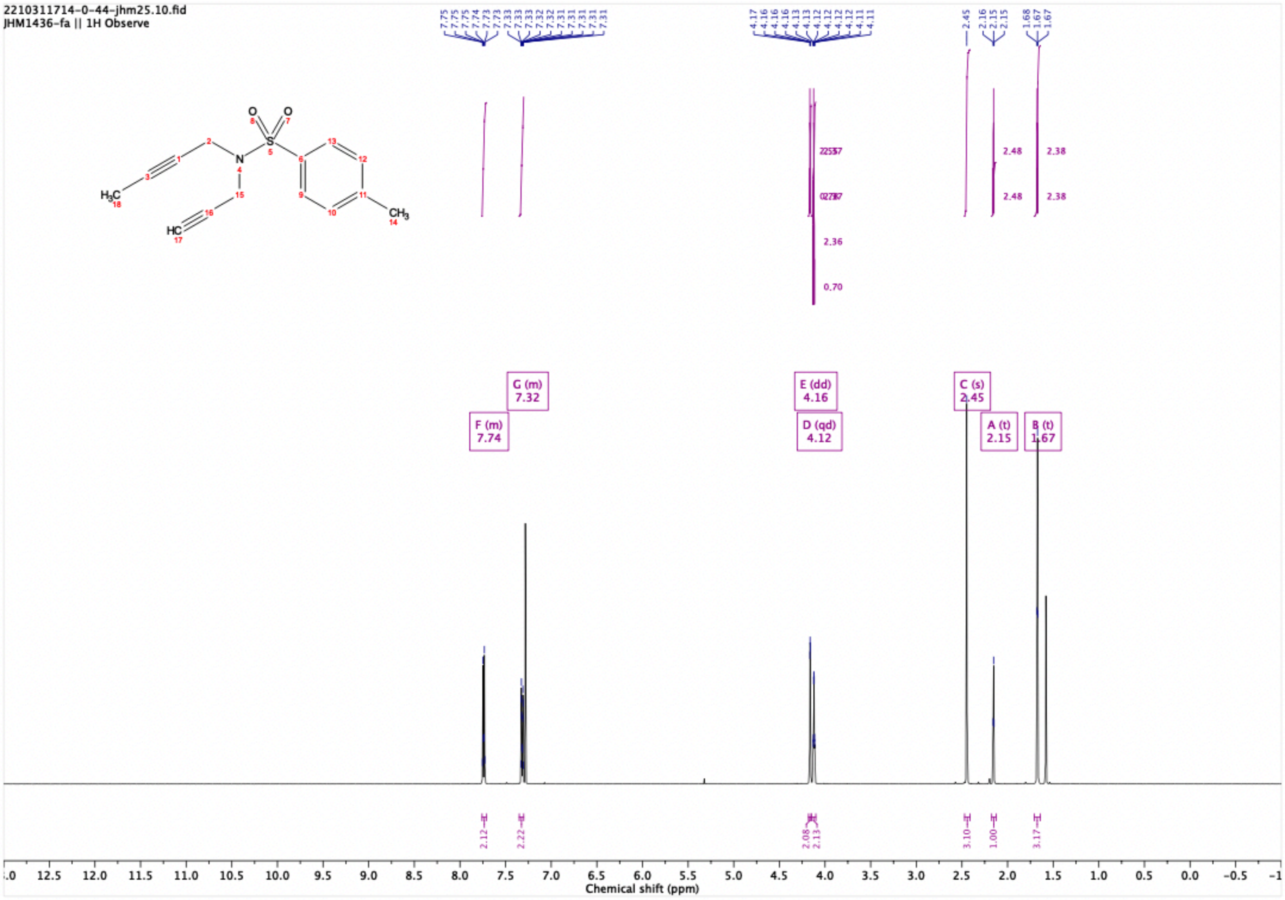

^{13}C DEPTQ

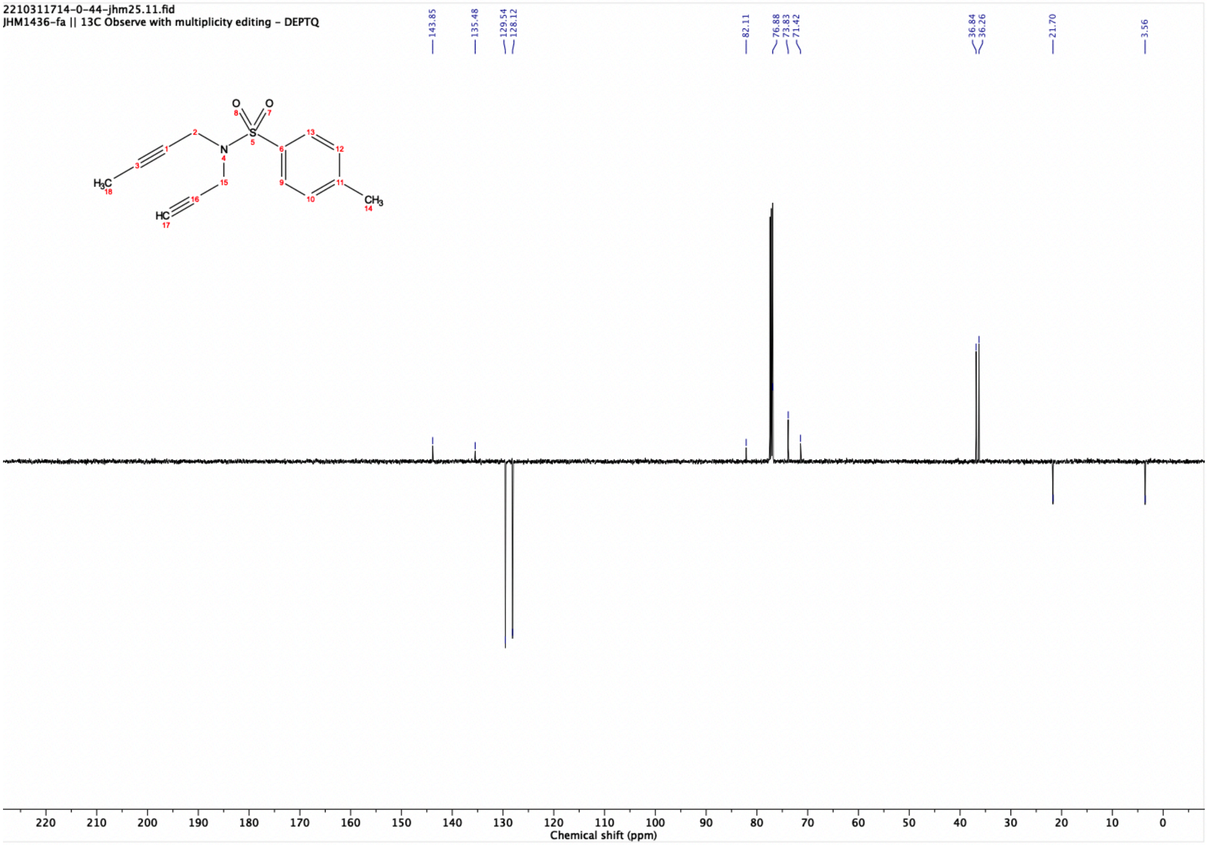

(S14)

^{1}H

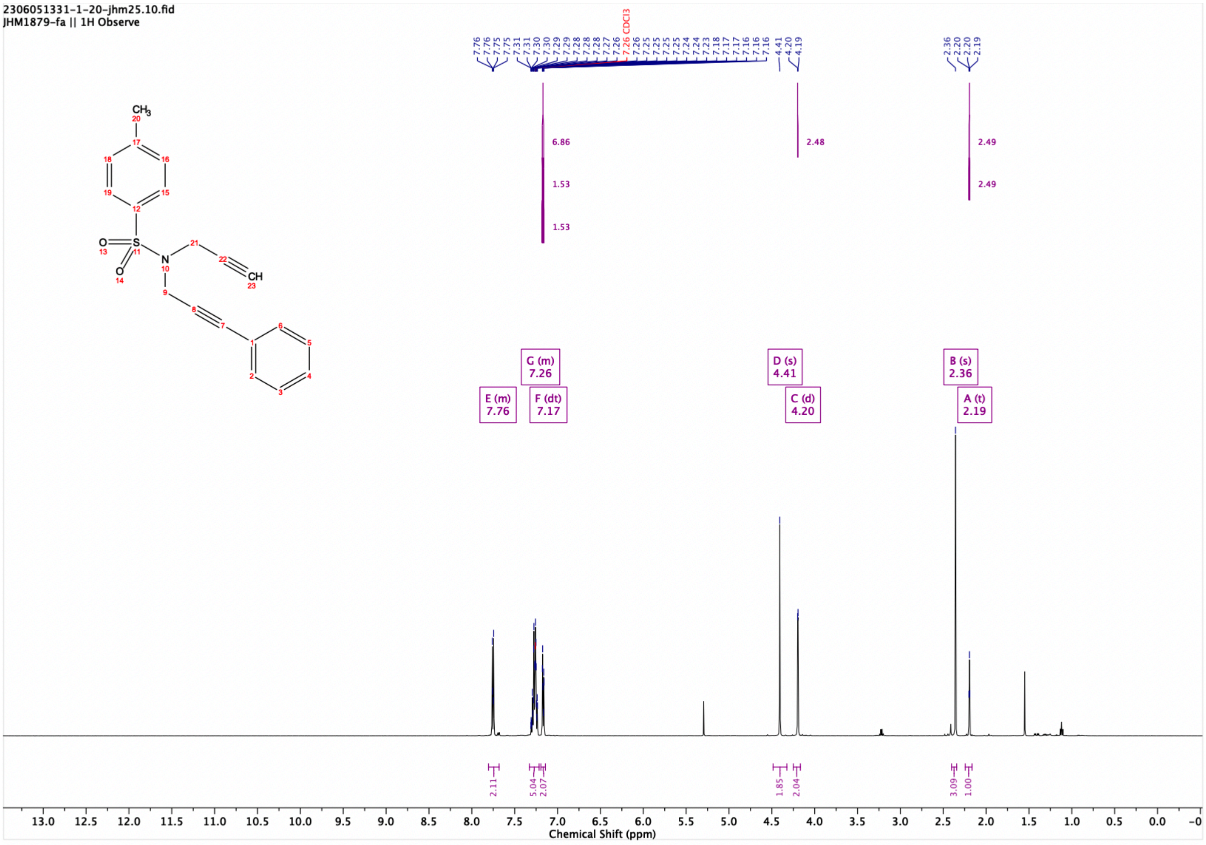

^{13}C DEPTQ

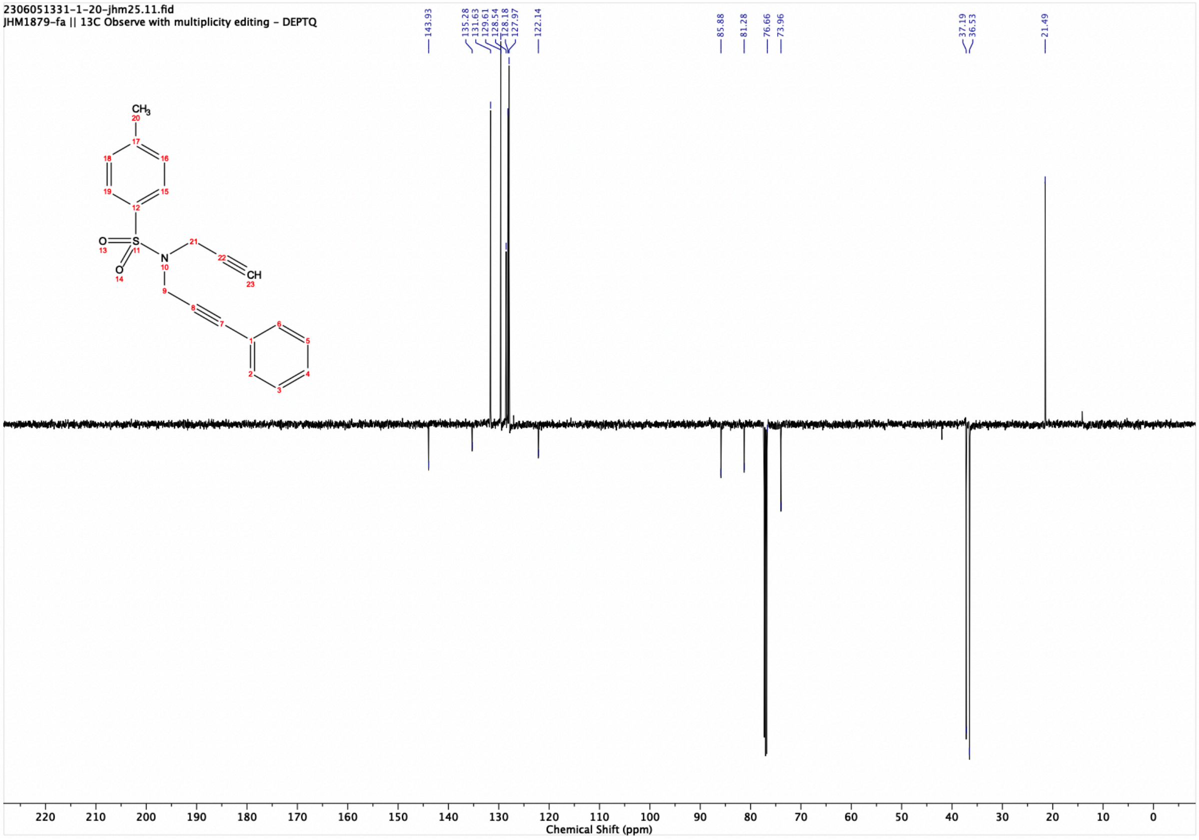

(S15)

^{1}H

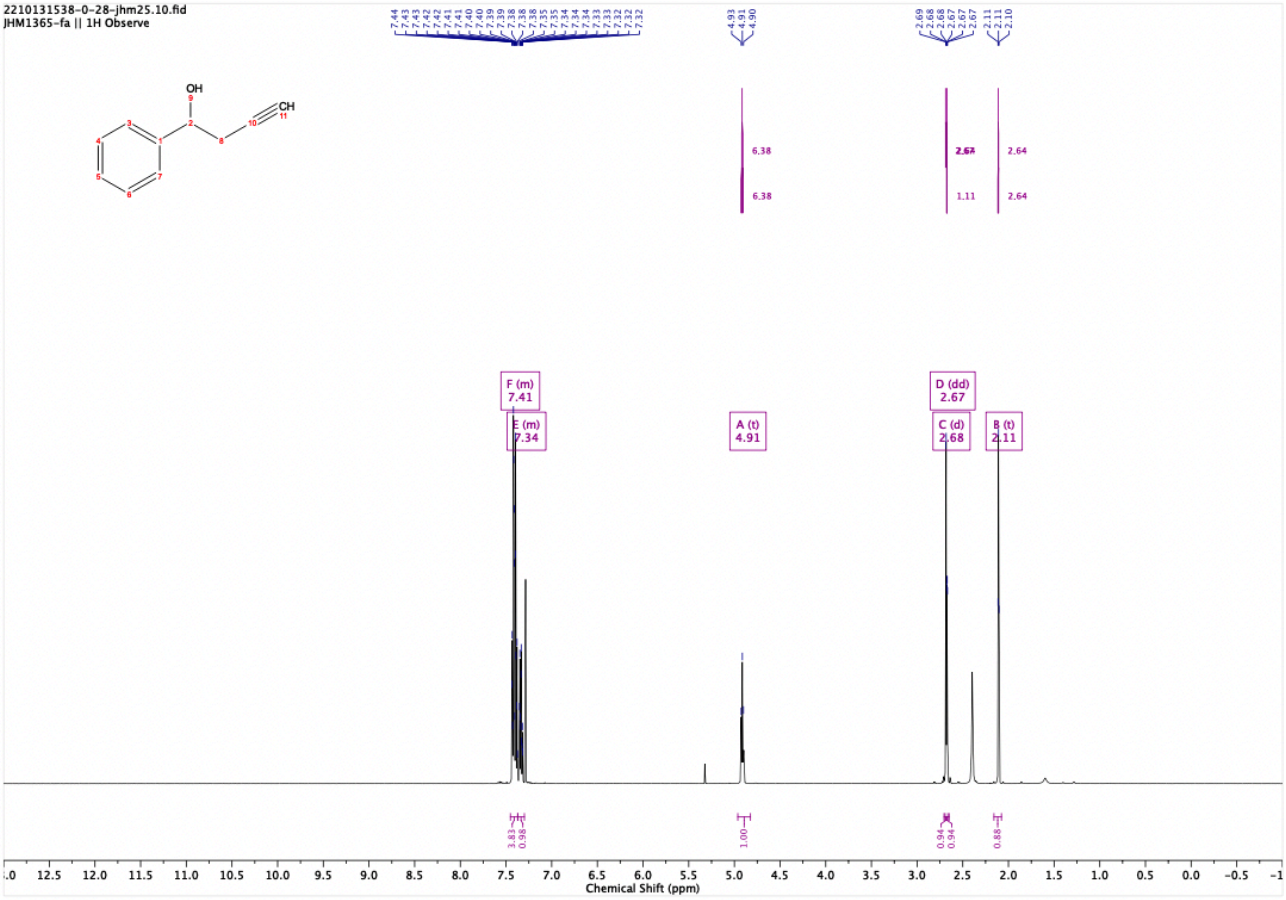

^{13}C DEPTQ

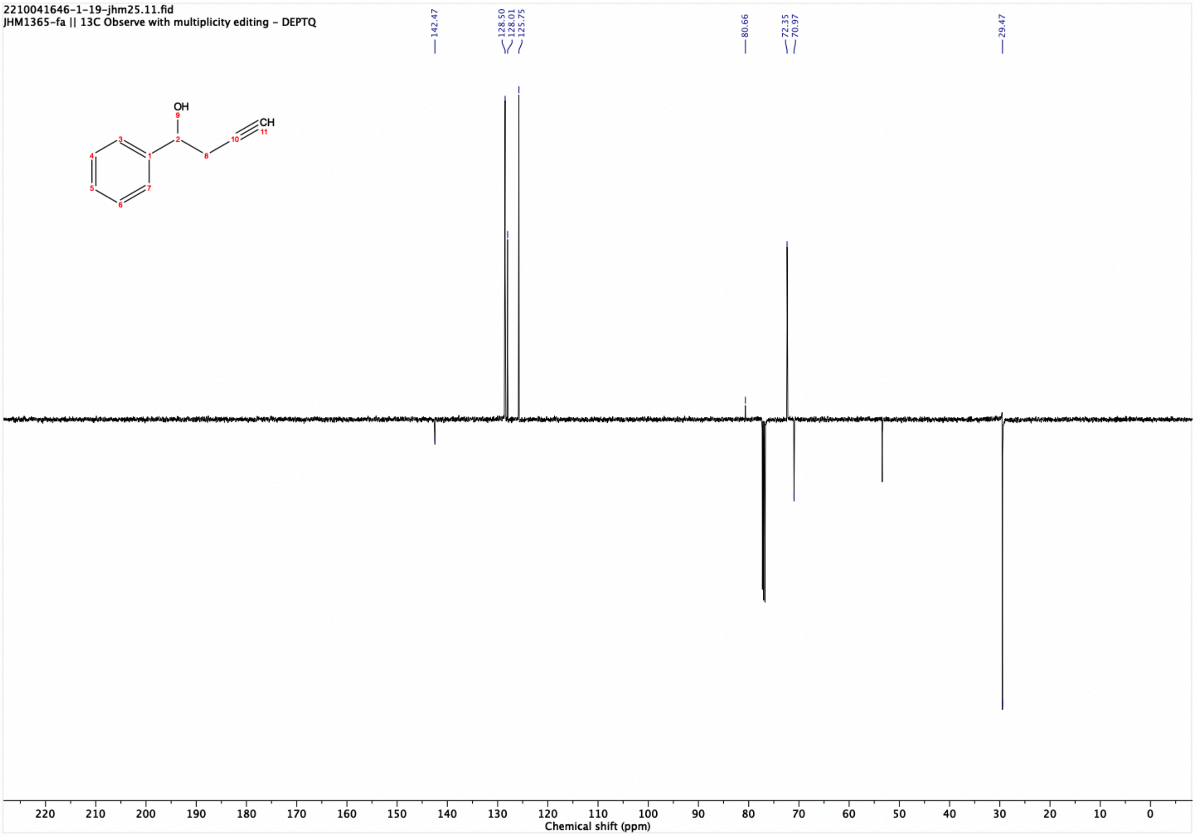

(S16)

^{1}H

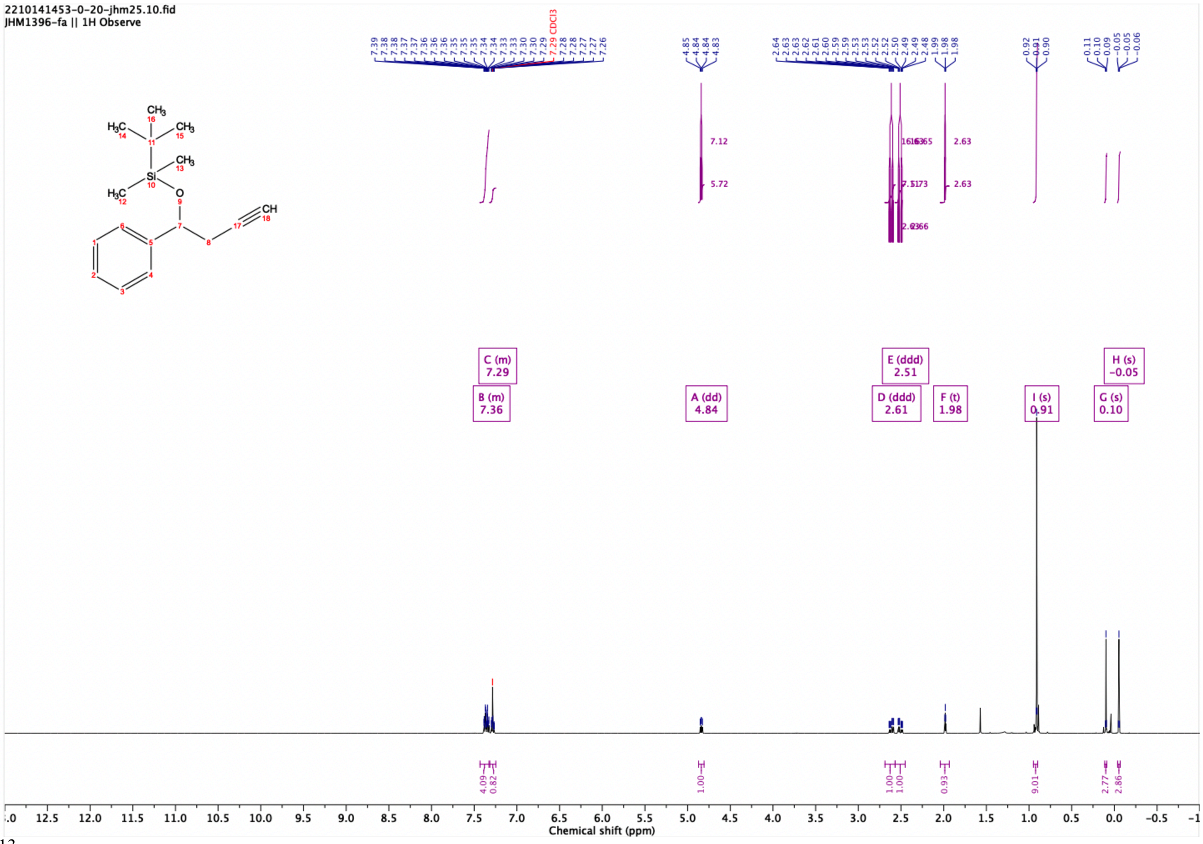

^{13}C DEPTQ

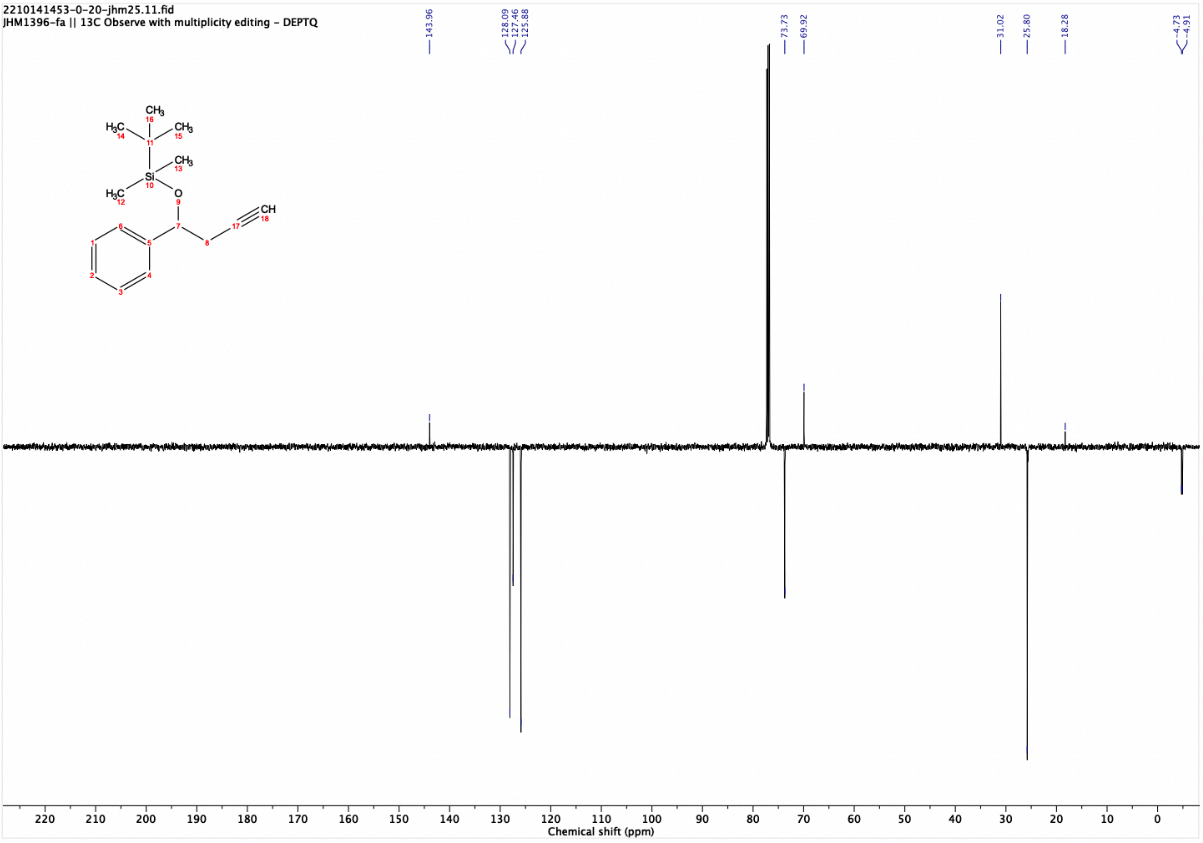

(S17)

^{1}H

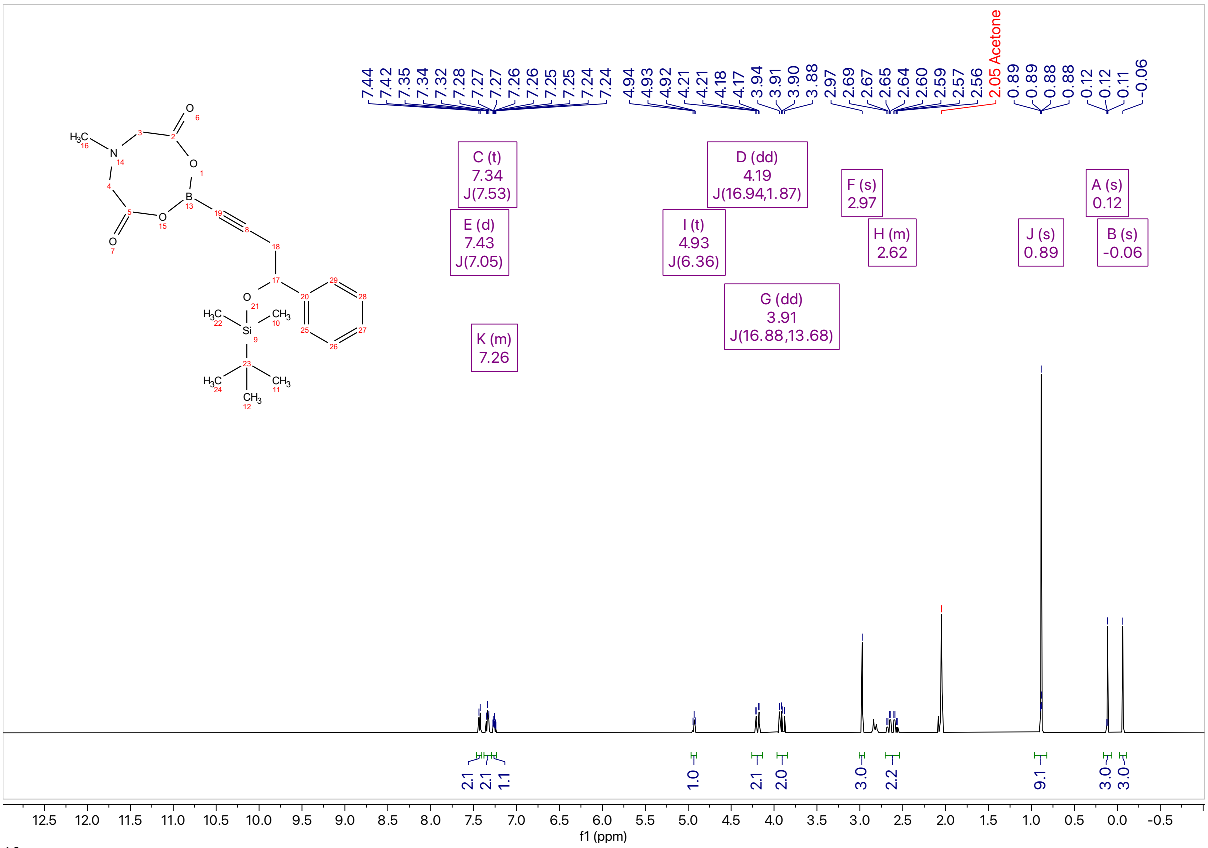

7.44
7.42
7.35
7.34
7.32
7.28
7.27
7.27
7.26
7.26
7.25
7.25
7.24
7.24
4.94
4.93
4.92
4.21
4.21
4.18
4.17
3.94
3.91
3.90
3.88
2.97
2.69
2.67
2.65
2.64
2.60
2.59
2.57
2.56
2.05 Acetone
0.89
0.89
0.88
0.88
0.12
0.12
0.11
-0.06
C (t) 7.34 J(7.53)
E (d) 7.43 J(7.05)
K (m) 7.26
I (t) 4.93 J(6.36)
D (dd) 4.19 J(16.94,1.87)
G (dd) 3.91 J(16.88,13.68)
F (s) 2.97
H (m) 2.62
J (s) 0.89
A (s) 0.12
B (s) -0.06
2.1
2.1
1.1
1.0
2.1
2.0
3.0
2.2
9.1
3.0
3.0
f1 (ppm)

^{13}C

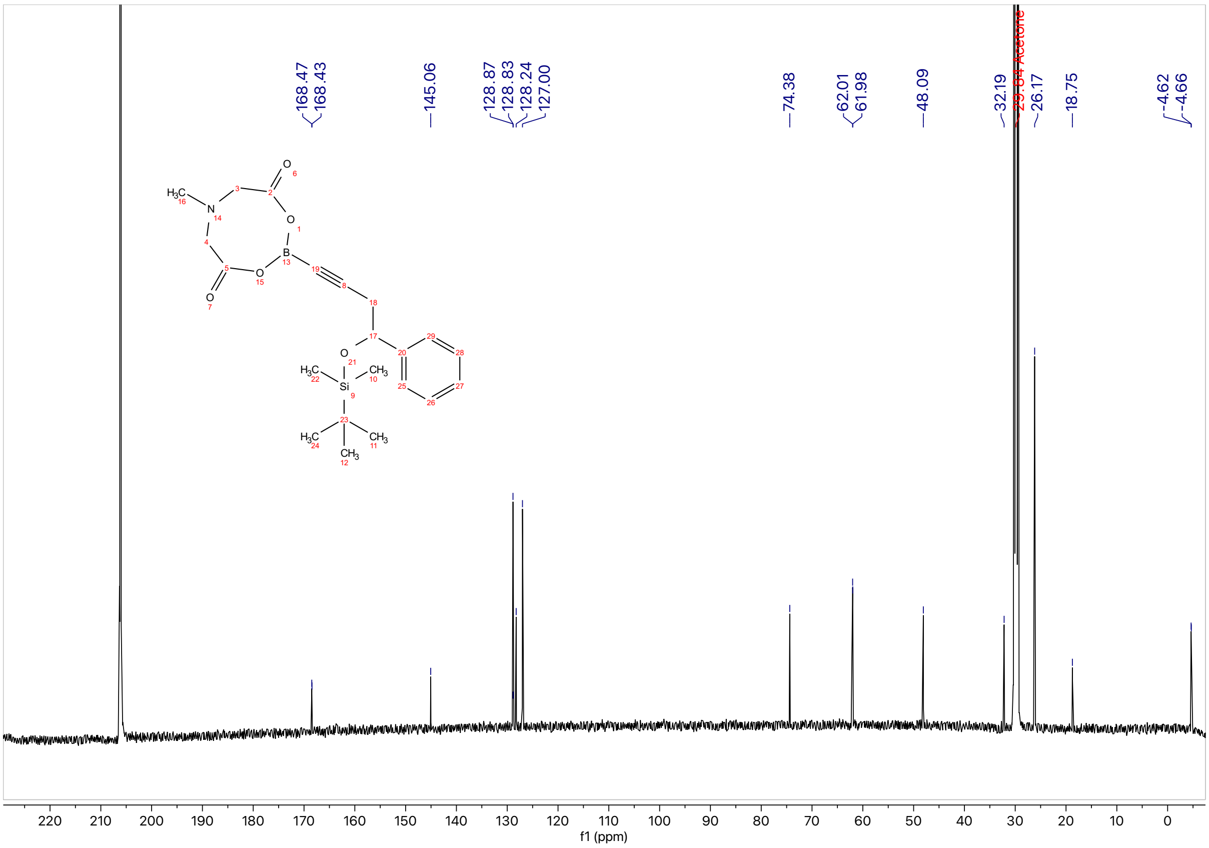

168.47
168.43
145.06
128.87
128.83
128.24
127.00
74.38
62.01
61.98
48.09
32.19
29.84 Acetone
26.17
18.75
-4.62
-4.66
f1 (ppm)

(S18)

^{1}H

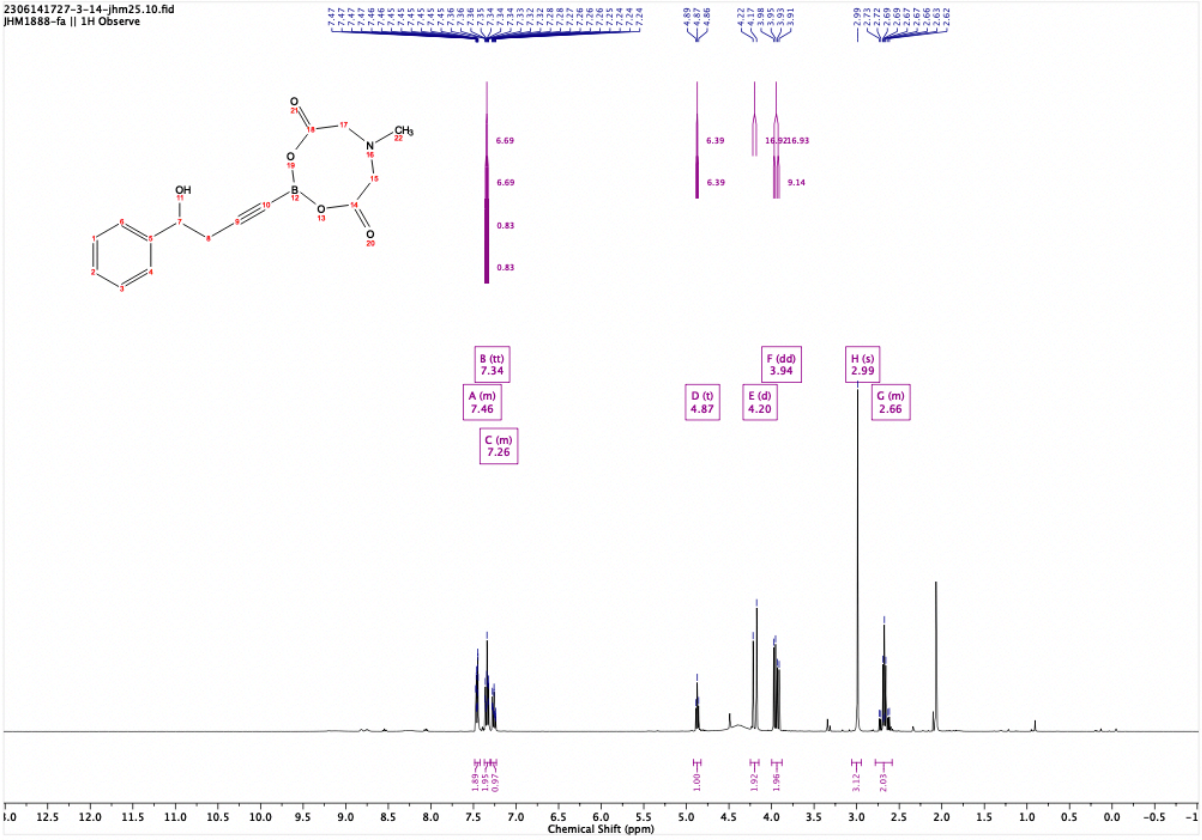

^{13}C DEPTQ

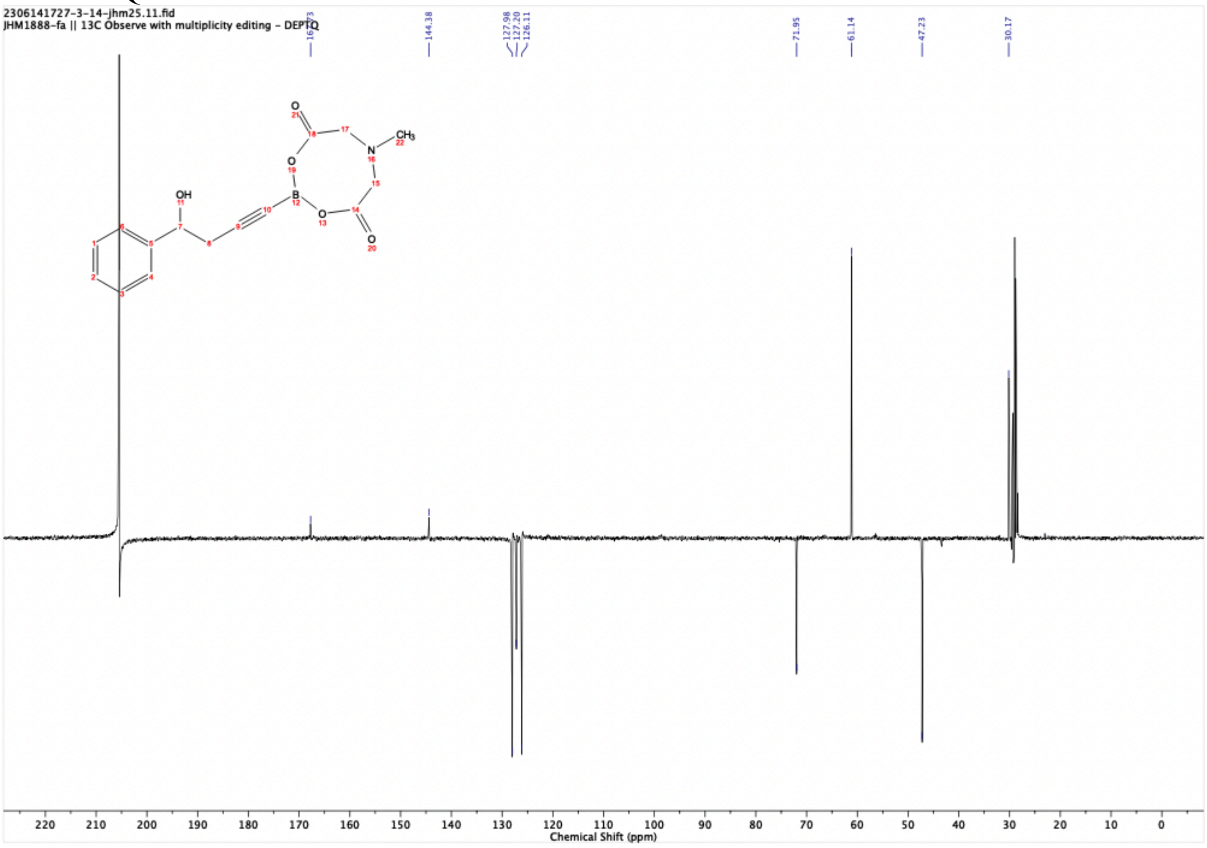

(S19)

^{1}H

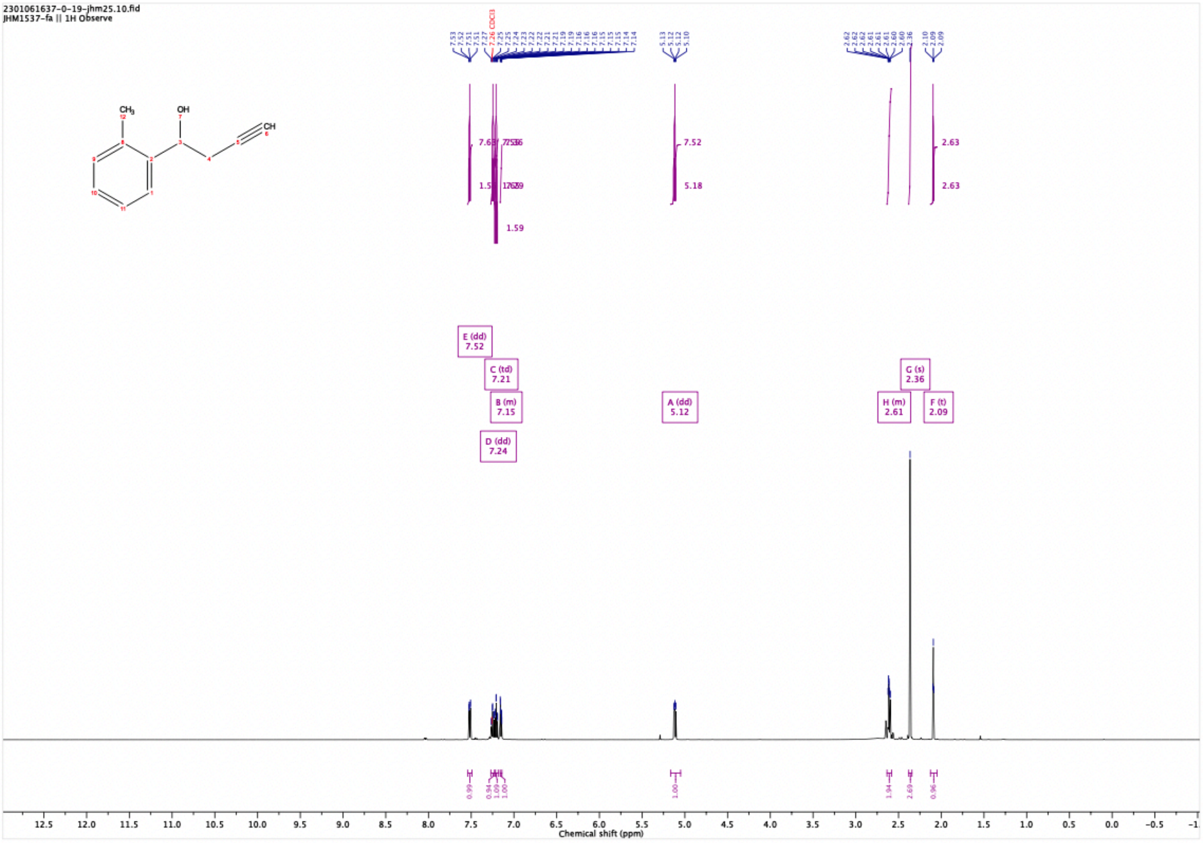

^{13}C DEPTQ

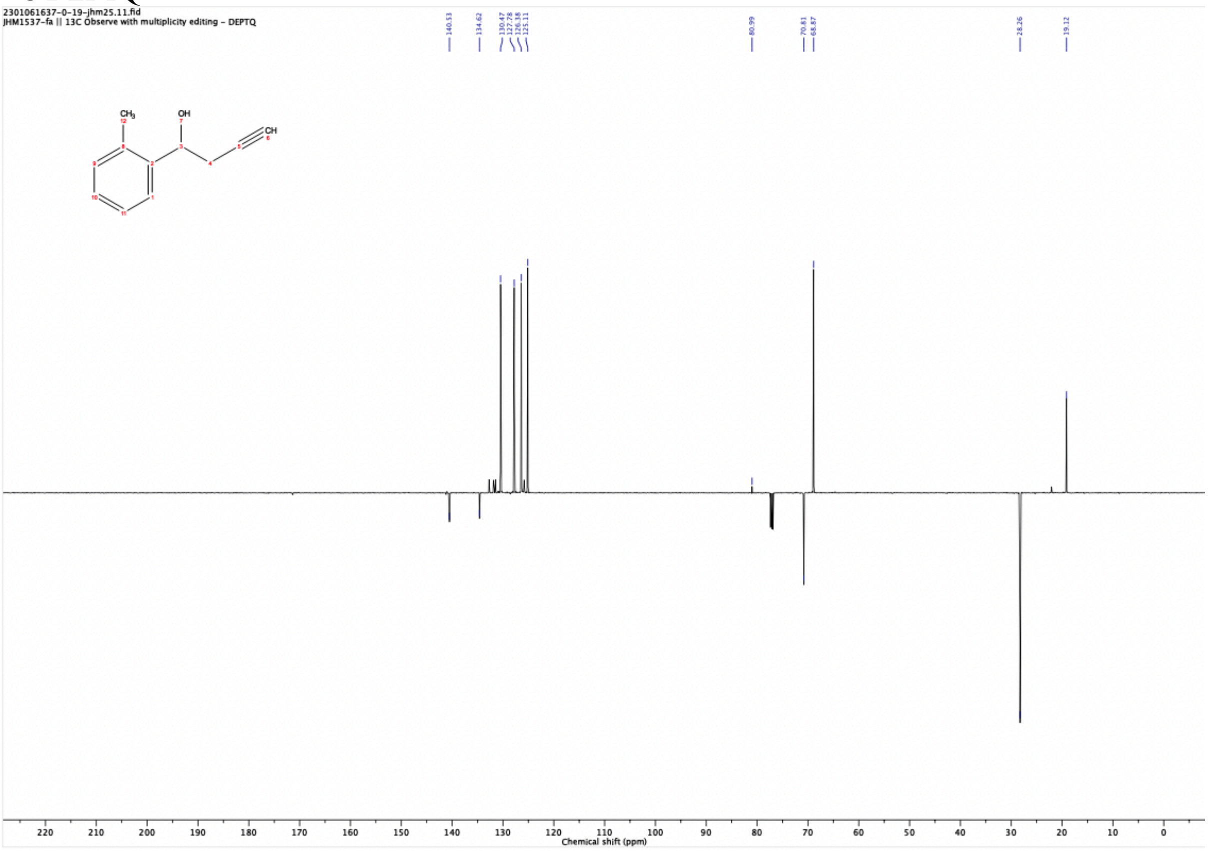

(S20)

^{1}H

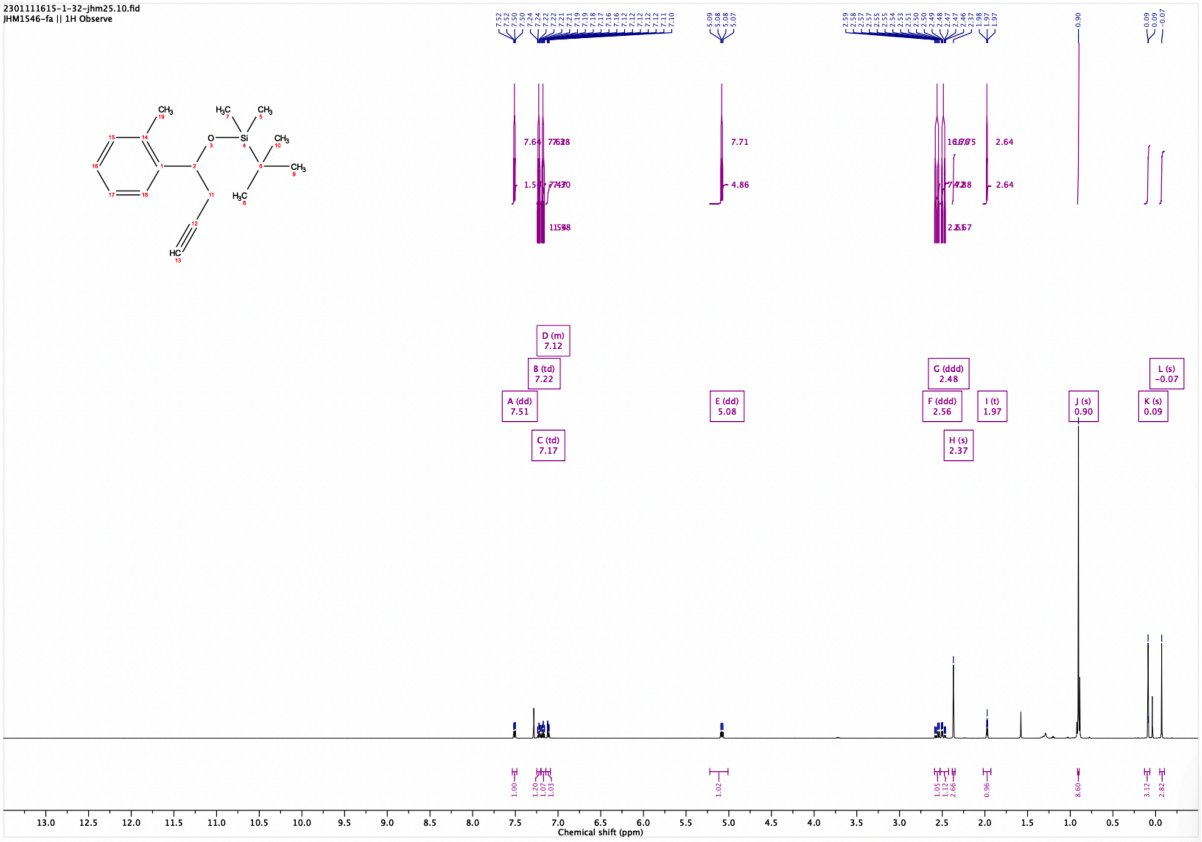

^{13}C DEPTQ

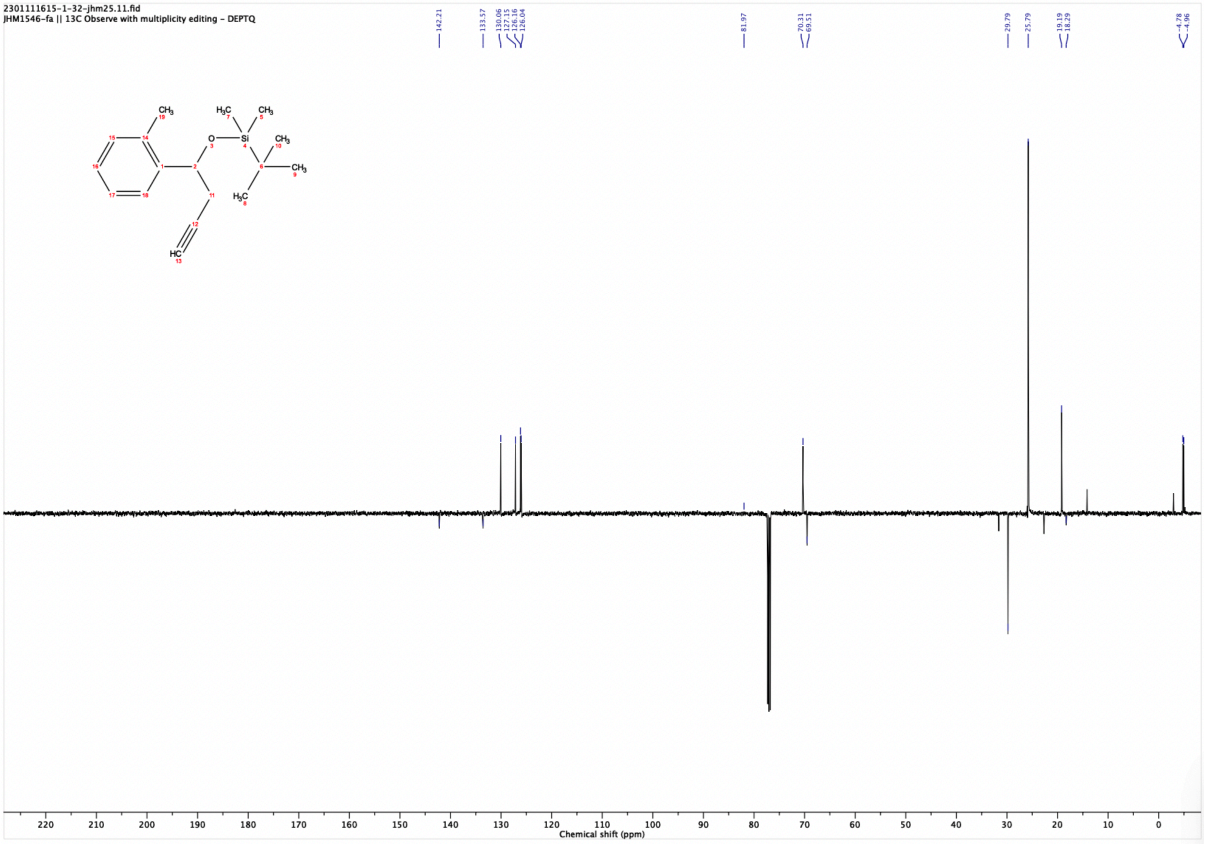

(S21)

^{1}H

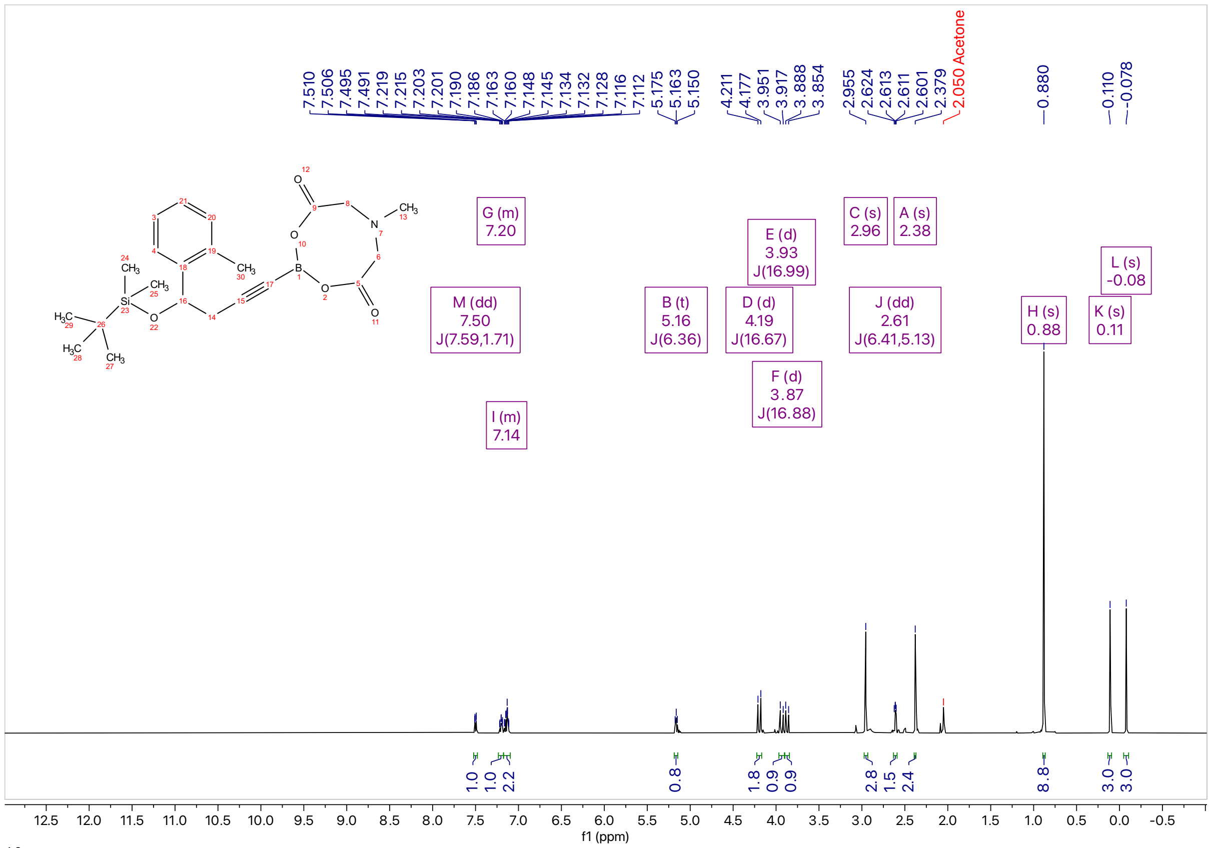
7.510
7.506
7.495
7.491
7.219
7.215
7.203
7.201
7.190
7.186
7.163
7.160
7.148
7.145
7.134
7.132
7.128
7.116
7.112
5.175
5.163
5.150
4.211
4.177
3.951
3.917
3.888
3.854
2.955
2.624
2.613
2.611
2.601
2.379
2.050 Acetone
0.880
0.110
-0.078
G (m) 7.20
C (s) 2.96
A (s) 2.38
E (d) 3.93 J(16.99)
L (s) -0.08
M (dd) 7.50 J(7.59,1.71)
B (t) 5.16 J(6.36)
D (d) 4.19 J(16.67)
J (dd) 2.61 J(6.41,5.13)
H (s) 0.88
K (s) 0.11
F (d) 3.87 J(16.88)
I (m) 7.14
1.0
1.0
2.2
0.8
1.8
0.9
0.9
2.8
1.5
2.4
8.8
3.0
3.0
f1 (ppm)

^{13}C

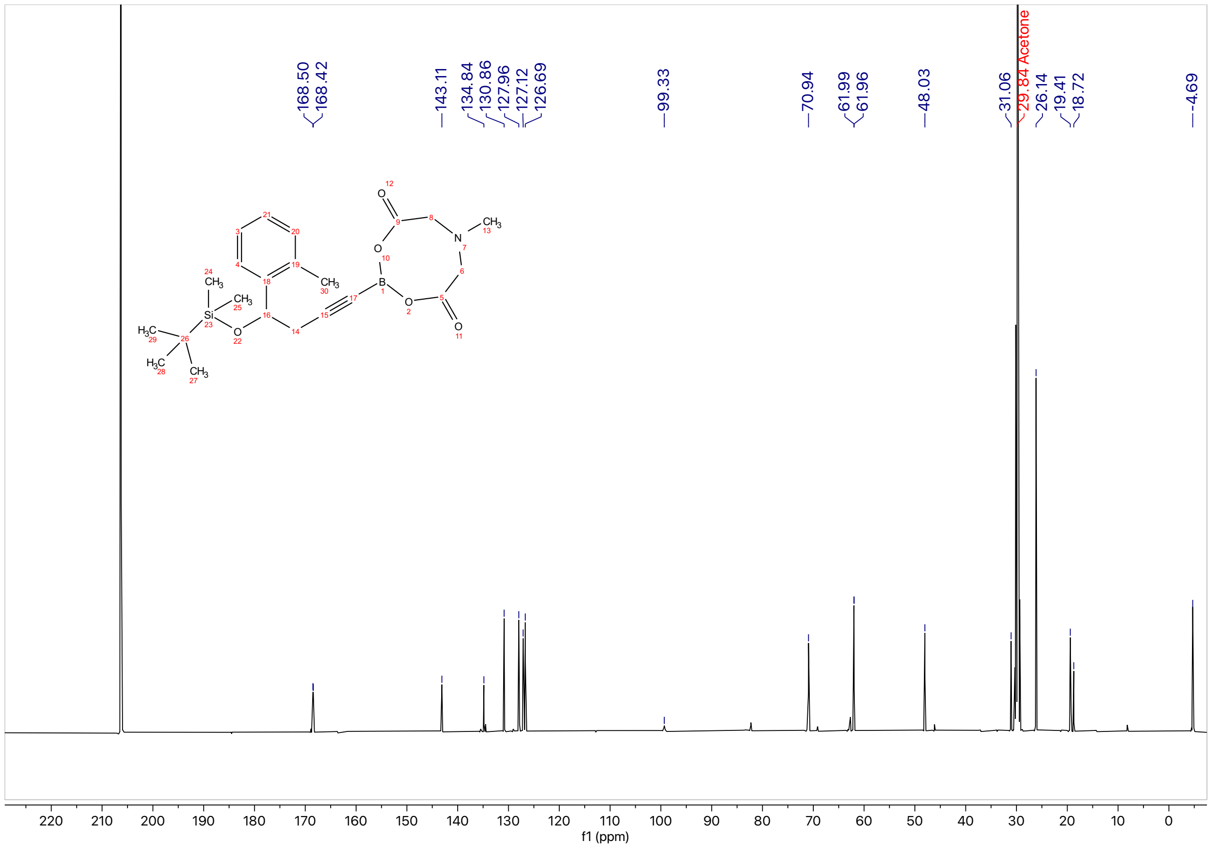
168.50
168.42
143.11
134.84
130.86
127.96
127.12
126.69
99.33
70.94
61.99
61.96
48.03
31.06
29.84 Acetone
26.14
19.41
18.72
-4.69
f1 (ppm)

(S22)

^{1}H

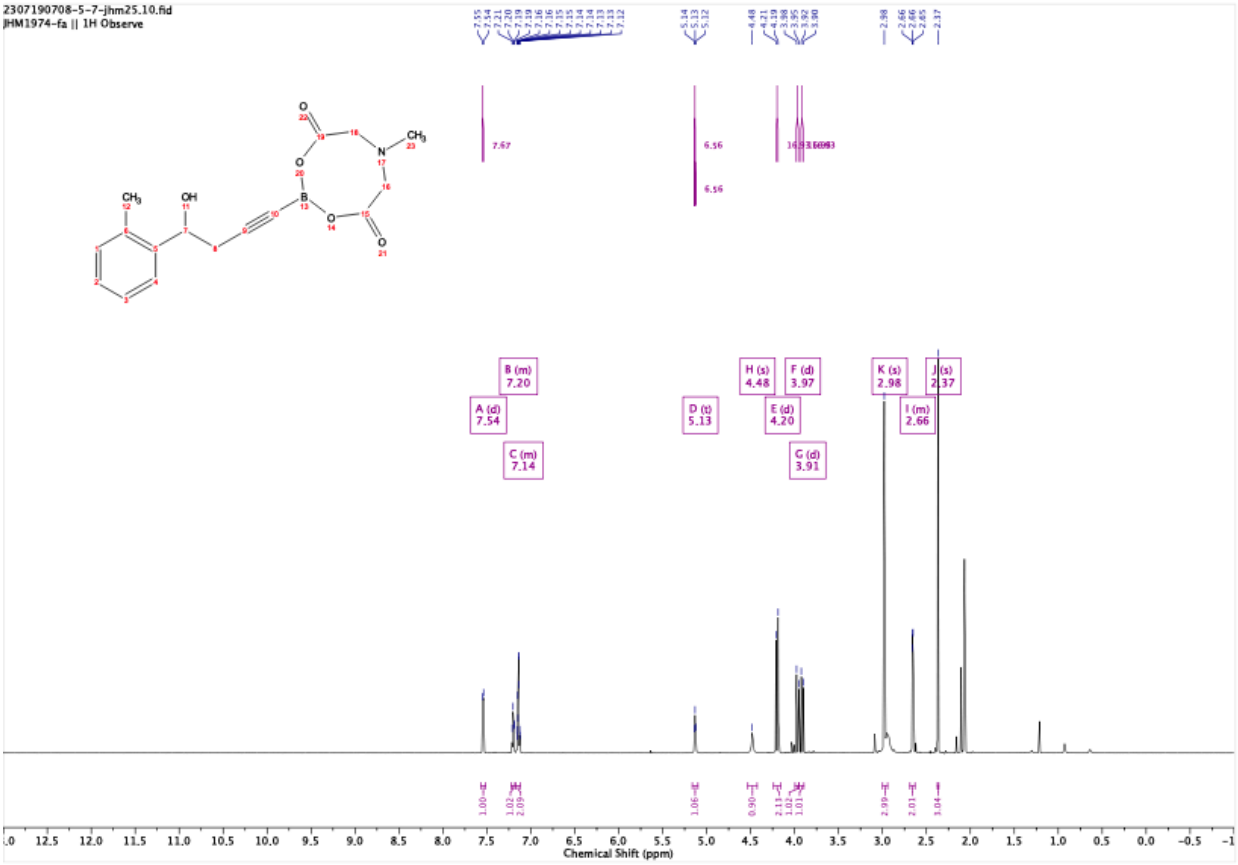

^{13}C DEPTQ

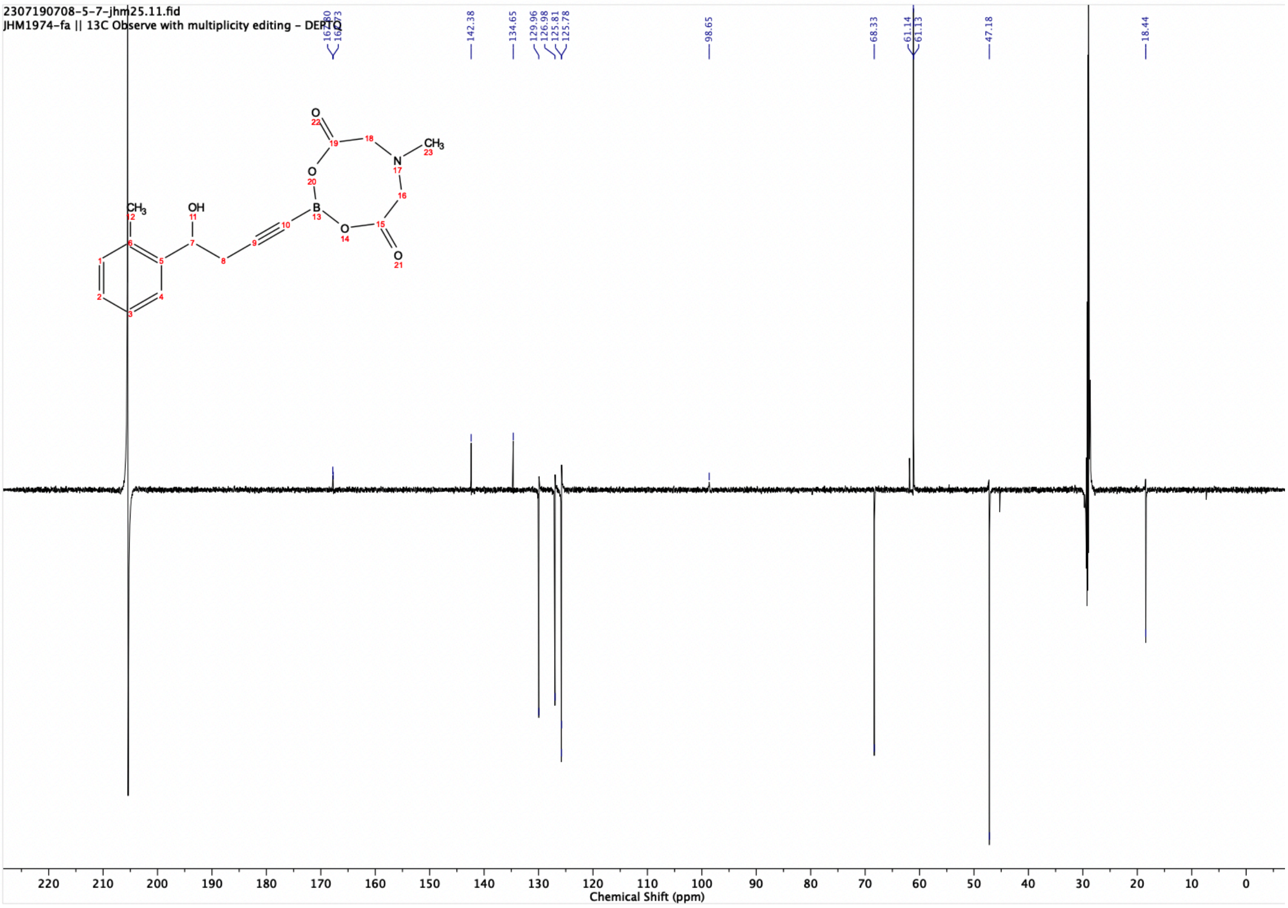

(S23)

^{1}H

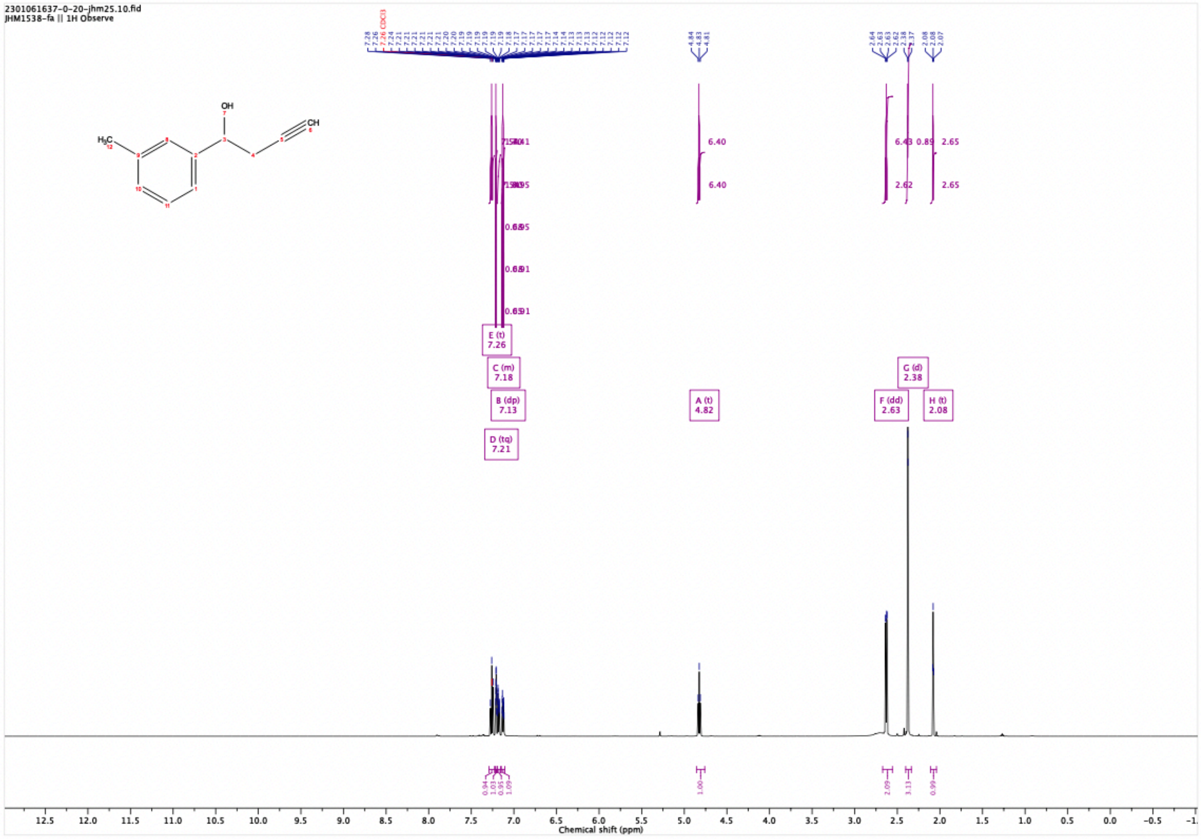

^{13}C DEPTQ

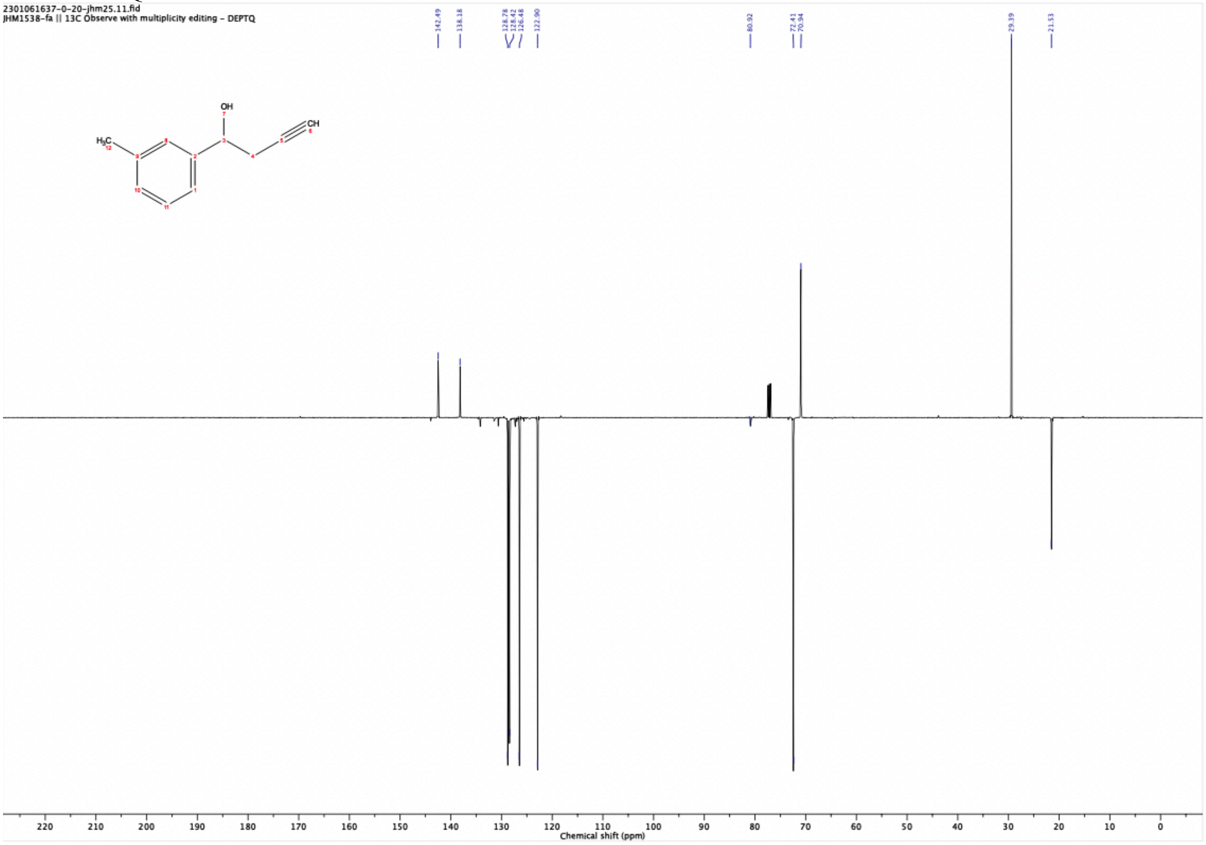

(S24)

^{1}H

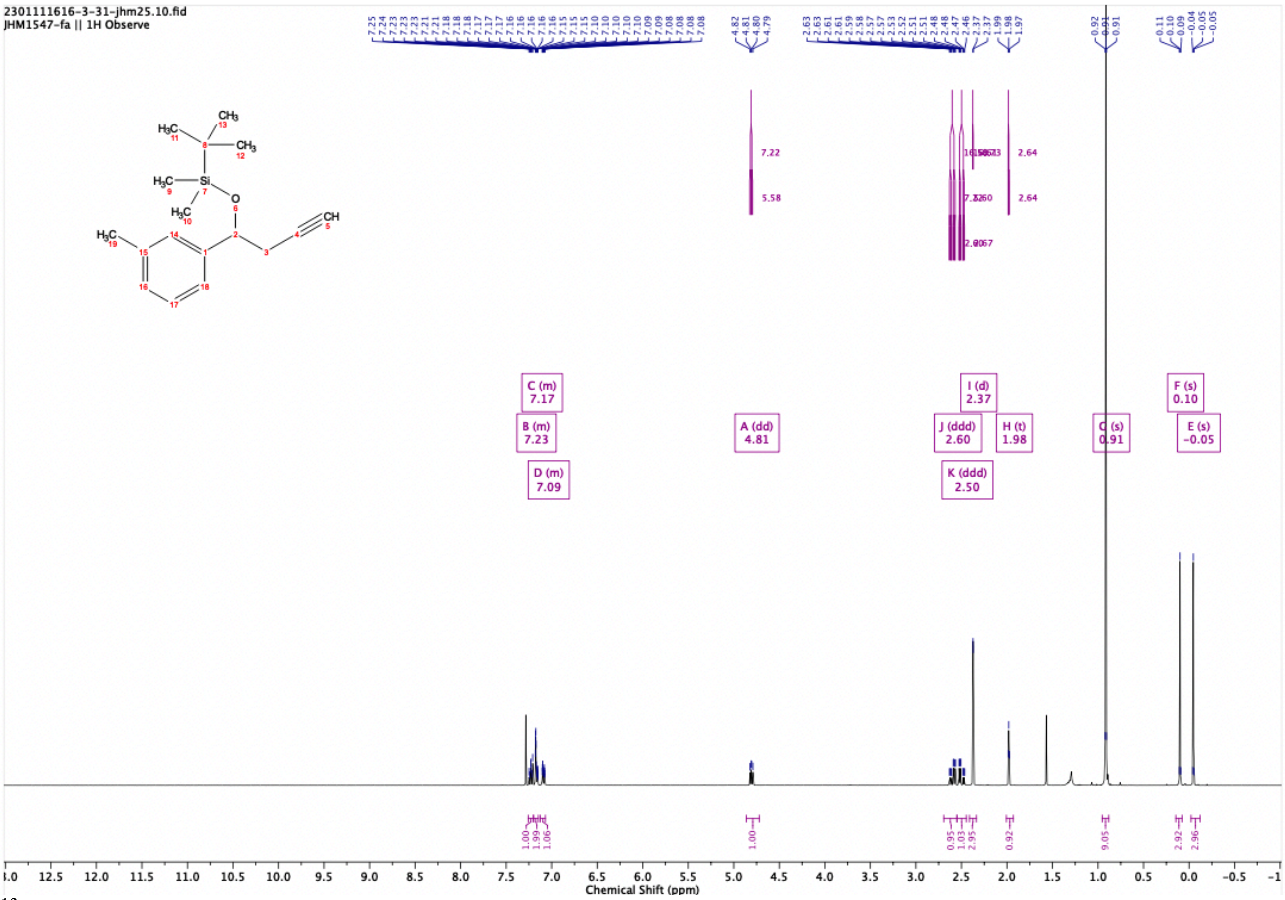

^{13}C DEPTQ

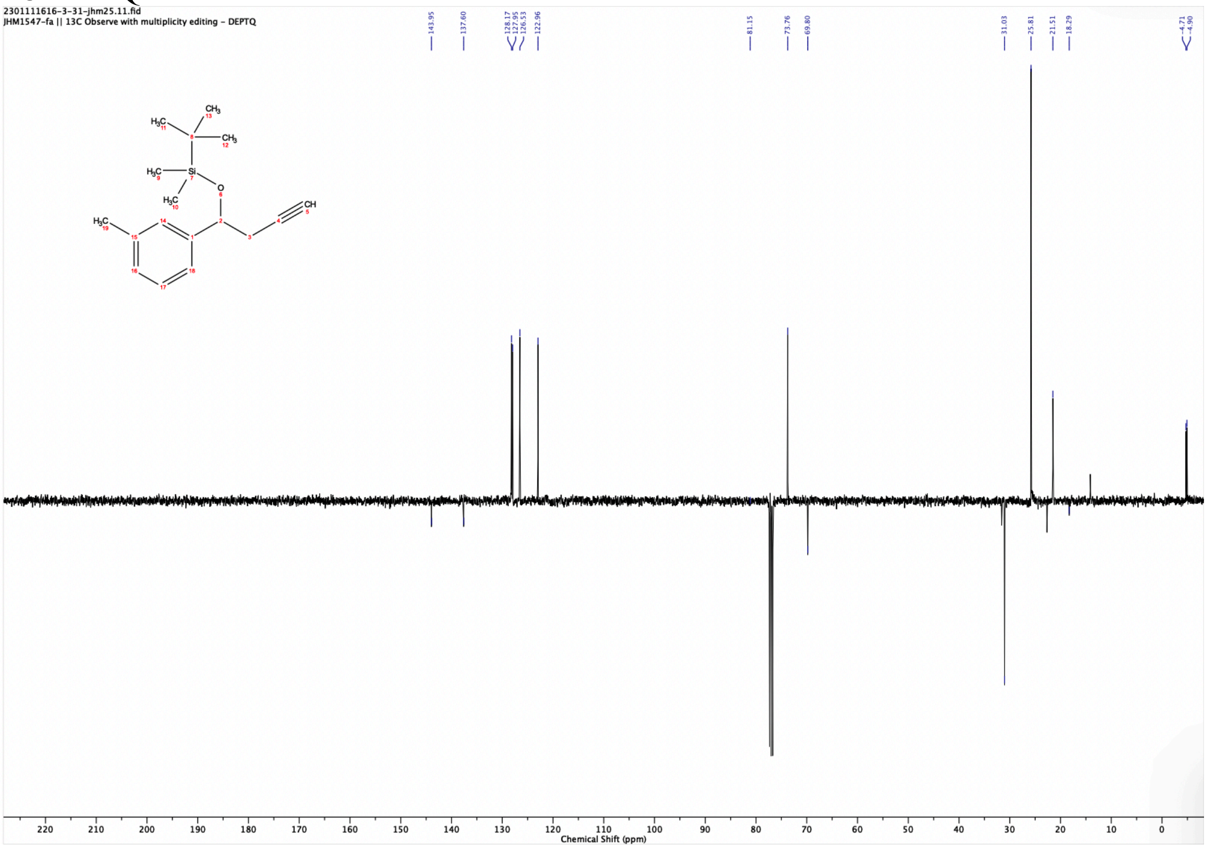

(S25)

^{1}H

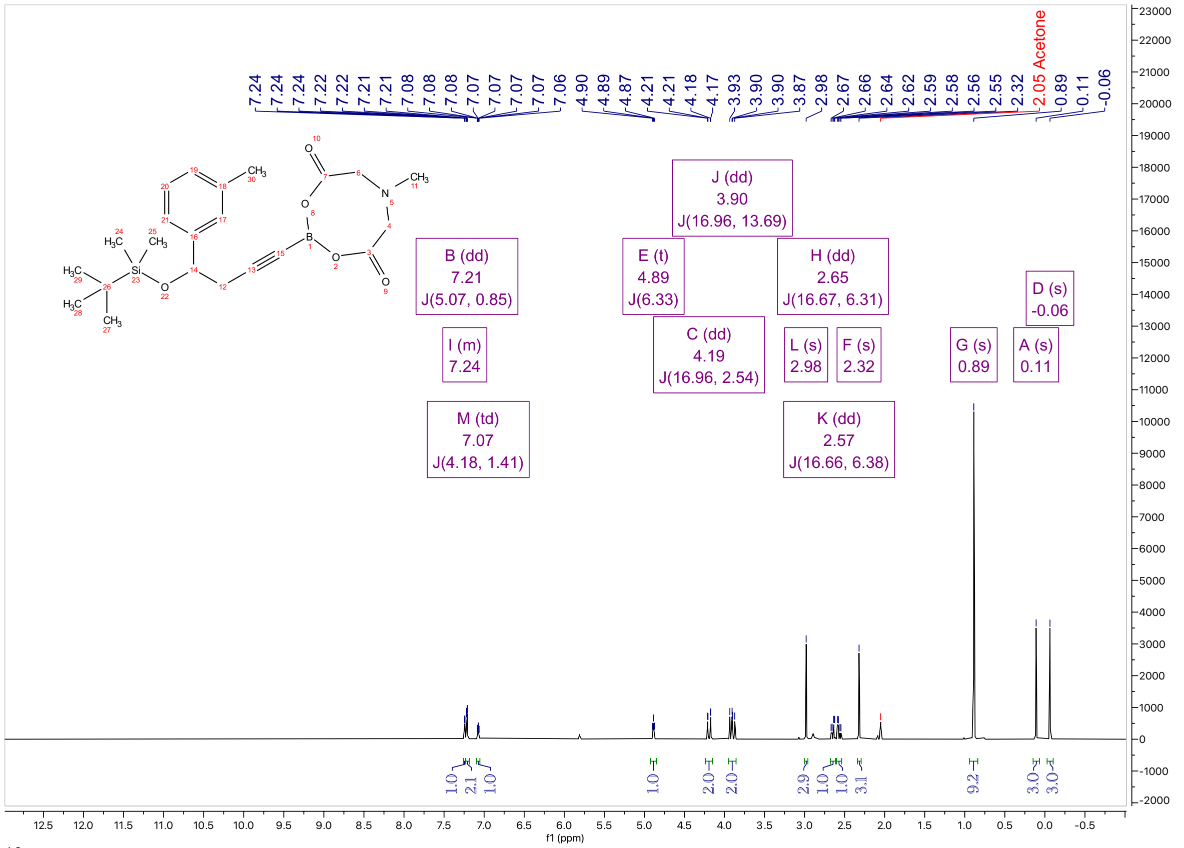

7.24
7.24
7.24
7.22
7.22
7.21
7.21
7.08
7.08
7.08
7.07
7.07
7.07
7.07
7.06
4.90
4.89
4.87
4.21
4.21
4.18
4.17
3.93
3.90
3.90
3.87
2.98
2.67
2.66
2.64
2.62
2.59
2.58
2.56
2.55
2.32
2.05 Acetone
0.89
0.11
-0.06
J (dd)
3.90
J(16.96, 13.69)
B (dd)
7.21
J(5.07, 0.85)
E (t)
4.89
J(6.33)
H (dd)
2.65
J(16.67, 6.31)
D (s)
-0.06
I (m)
7.24
C (dd)
4.19
J(16.96, 2.54)
L (s)
2.98
F (s)
2.32
G (s)
0.89
A (s)
0.11
M (td)
7.07
J(4.18, 1.41)
K (dd)
2.57
J(16.66, 6.38)
1.0
2.1
1.0
1.0
2.0
2.0
2.9
1.0
1.0
3.1
9.2
3.0
3.0
f1 (ppm)

^{13}C

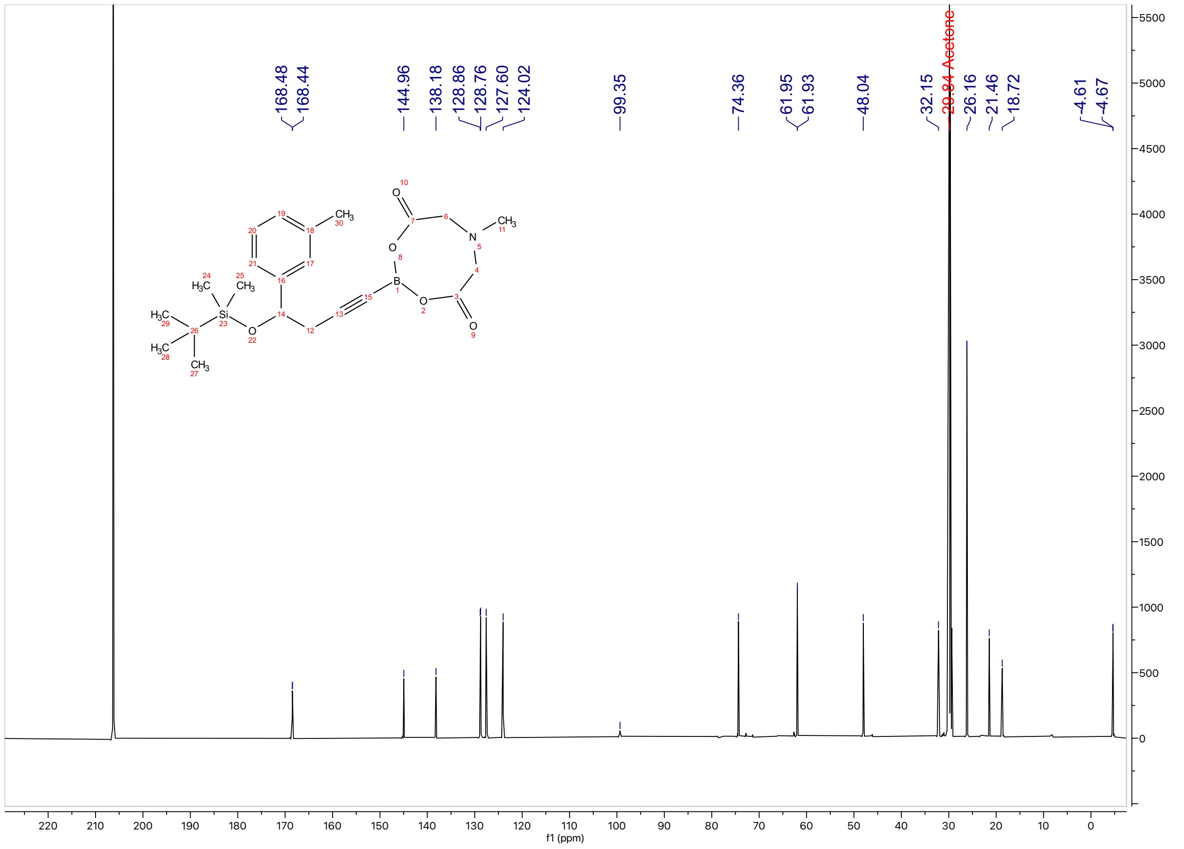

168.48
168.44
144.96
138.18
128.86
128.76
127.60
124.02
99.35
74.36
61.95
61.93
48.04
32.15
29.84 Acetone
26.16
21.46
18.72
-4.61
-4.67
f1 (ppm)

(S26)

^{1}H

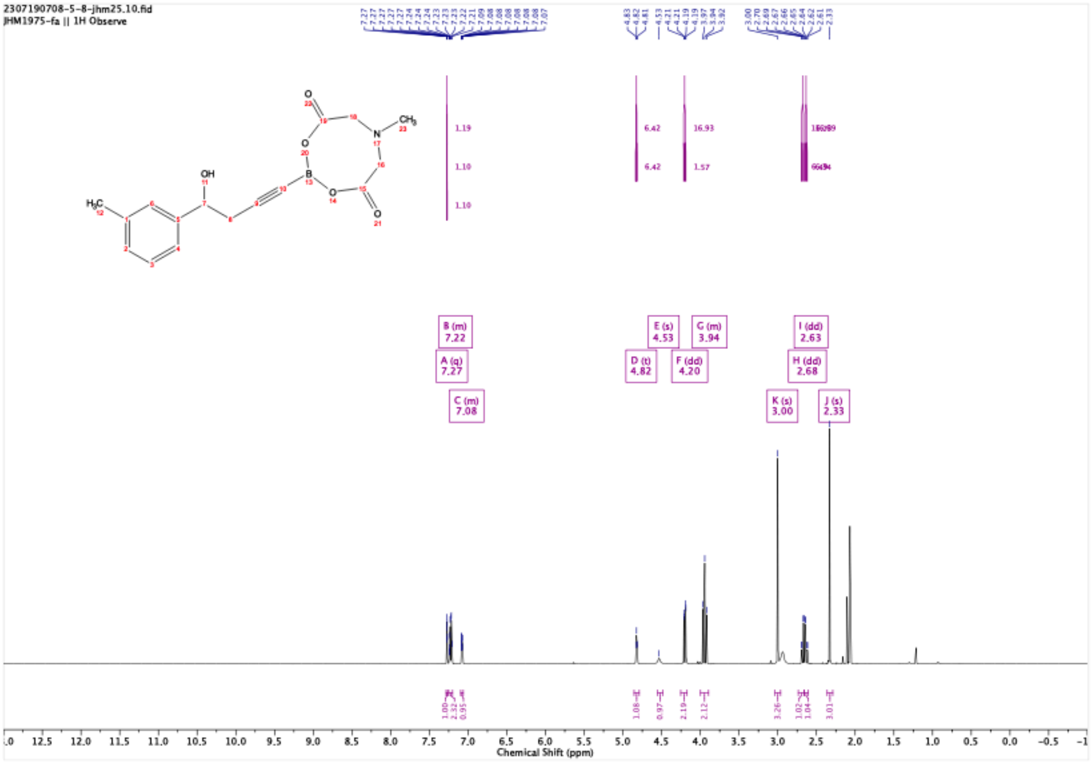

^{13}C DEPTQ

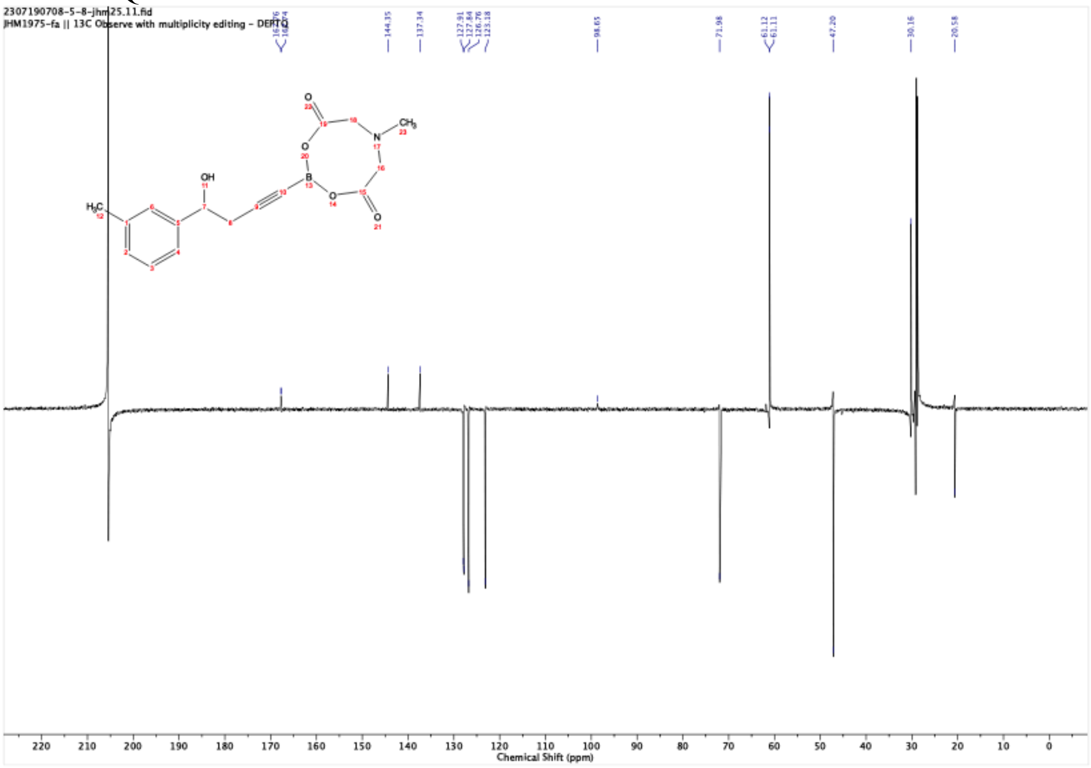

(S27)

^{1}H

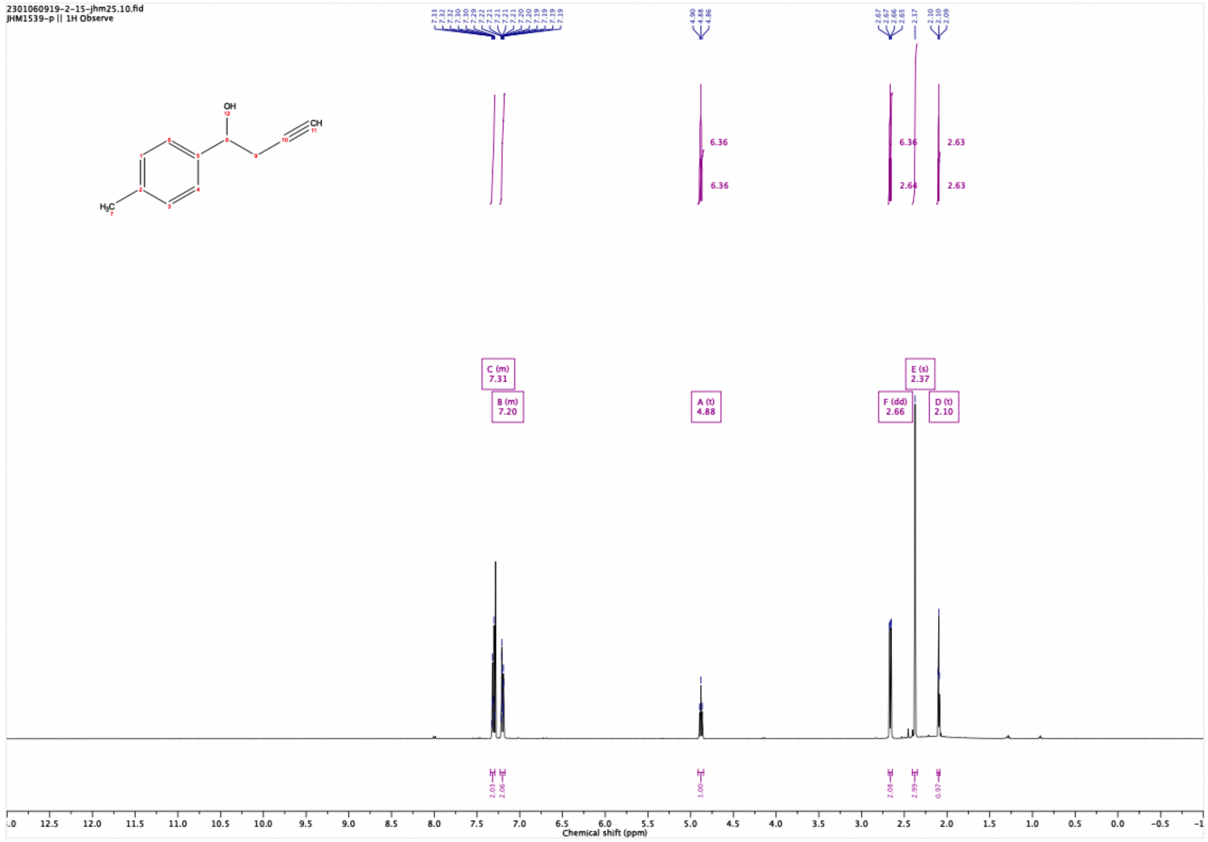

^{13}C DEPTQ

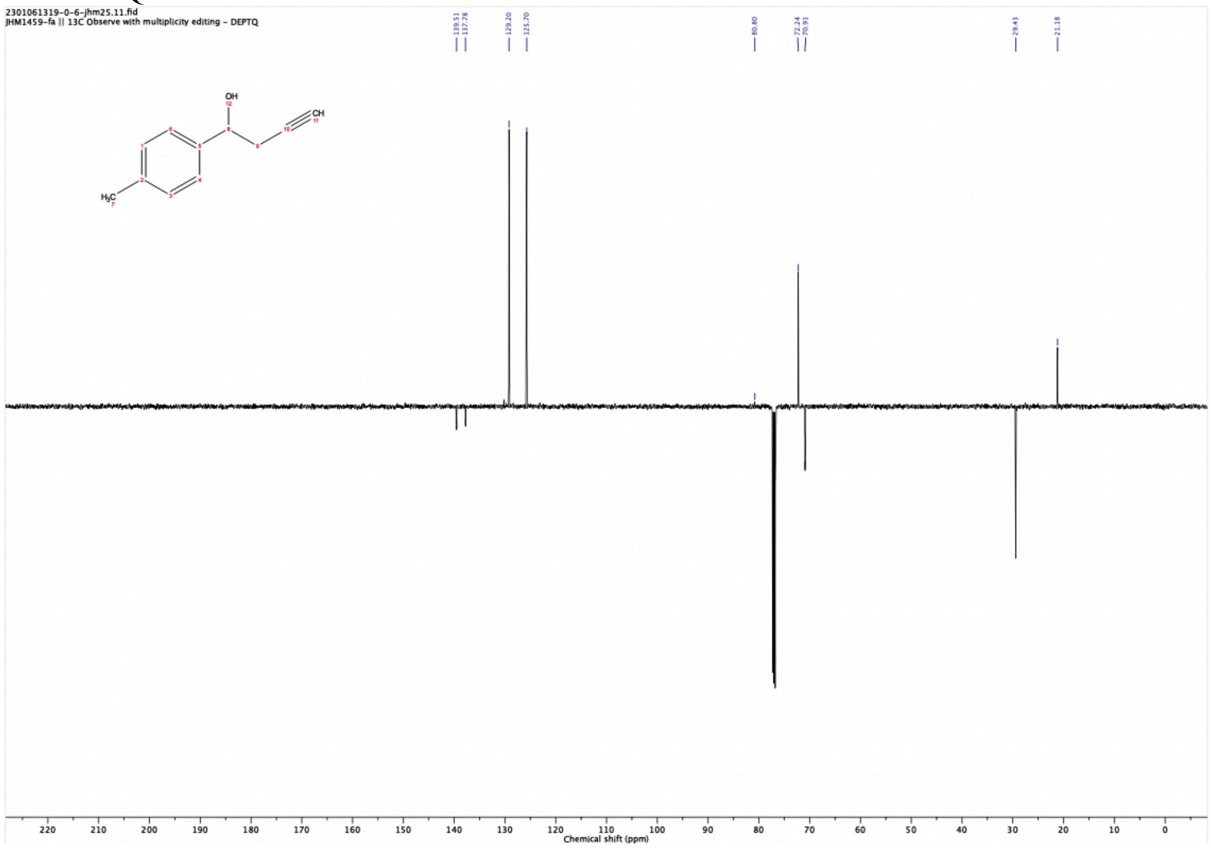

(S28)

^{1}H

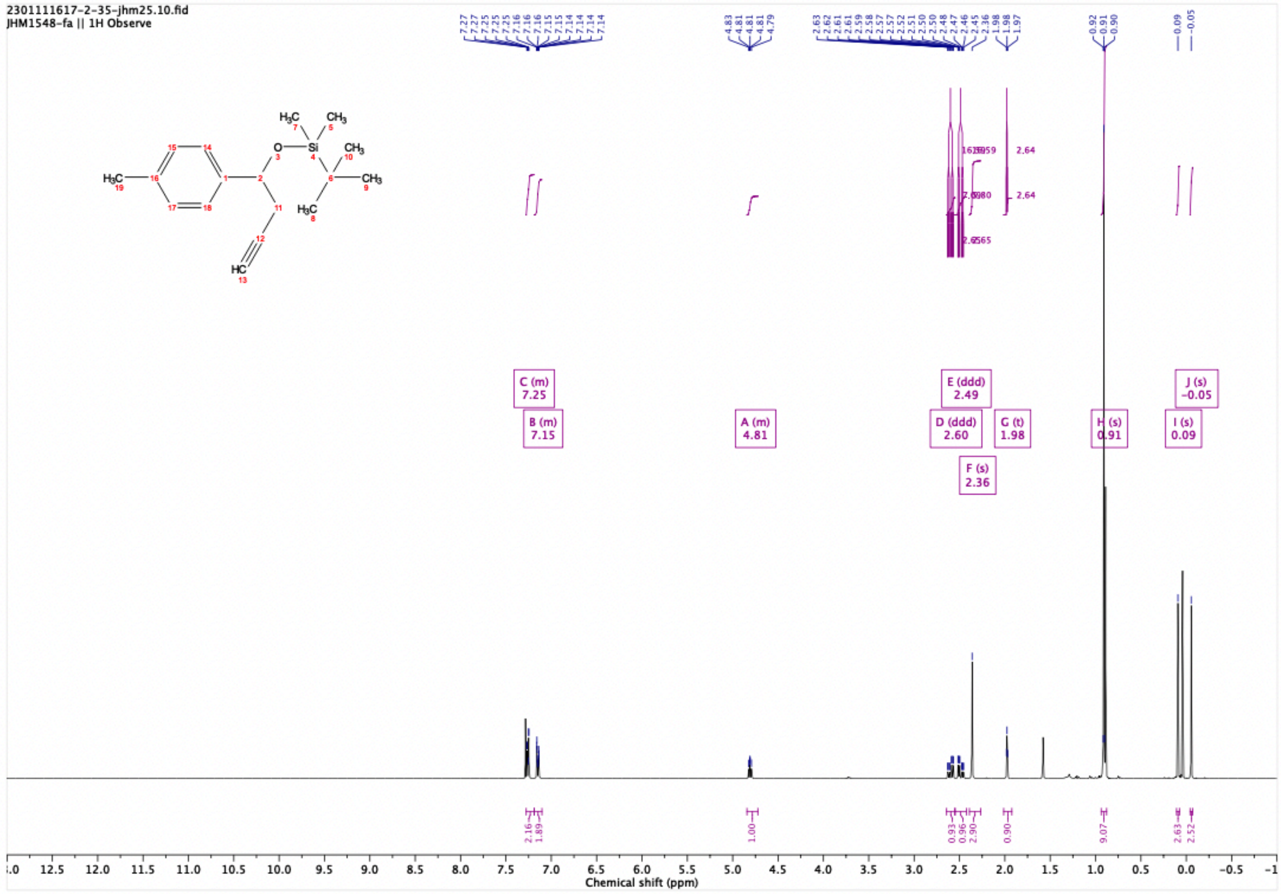

^{13}C DEPTQ

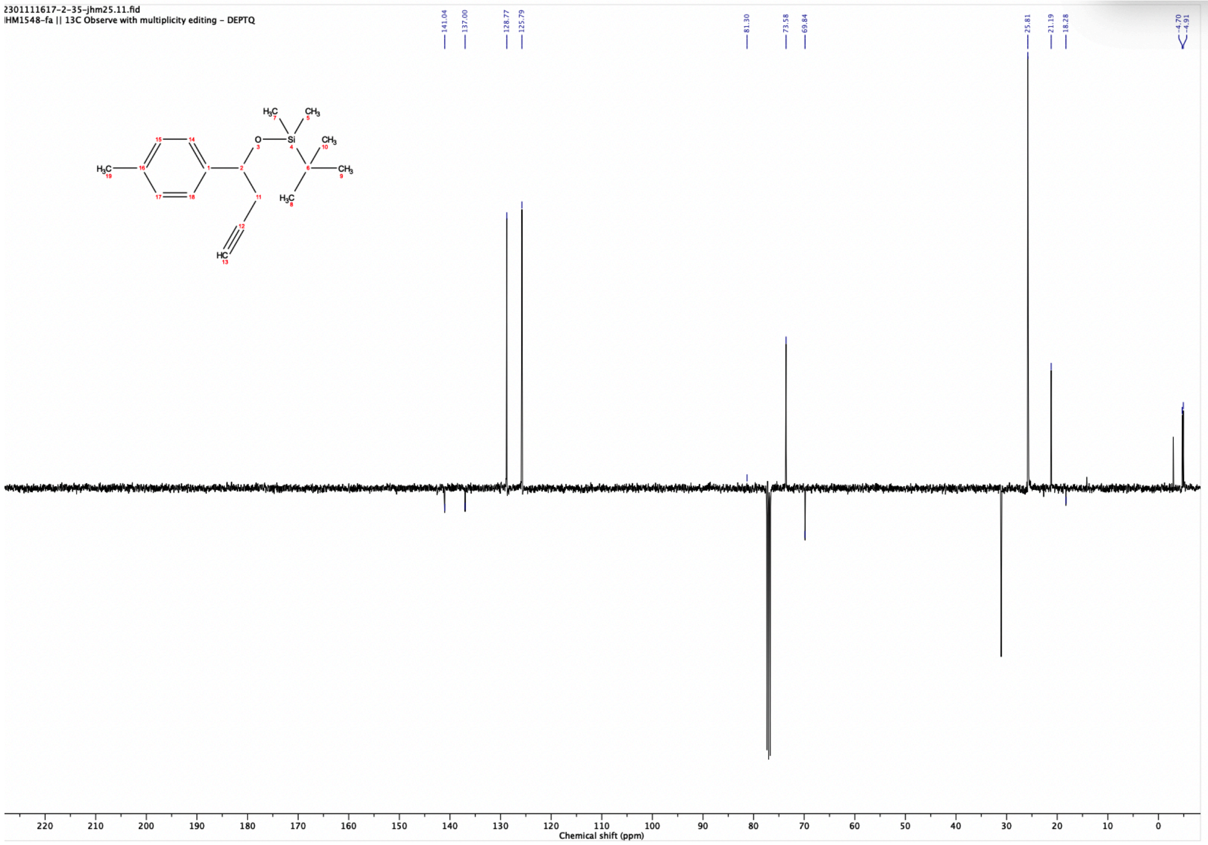

(S29)

^{1}H

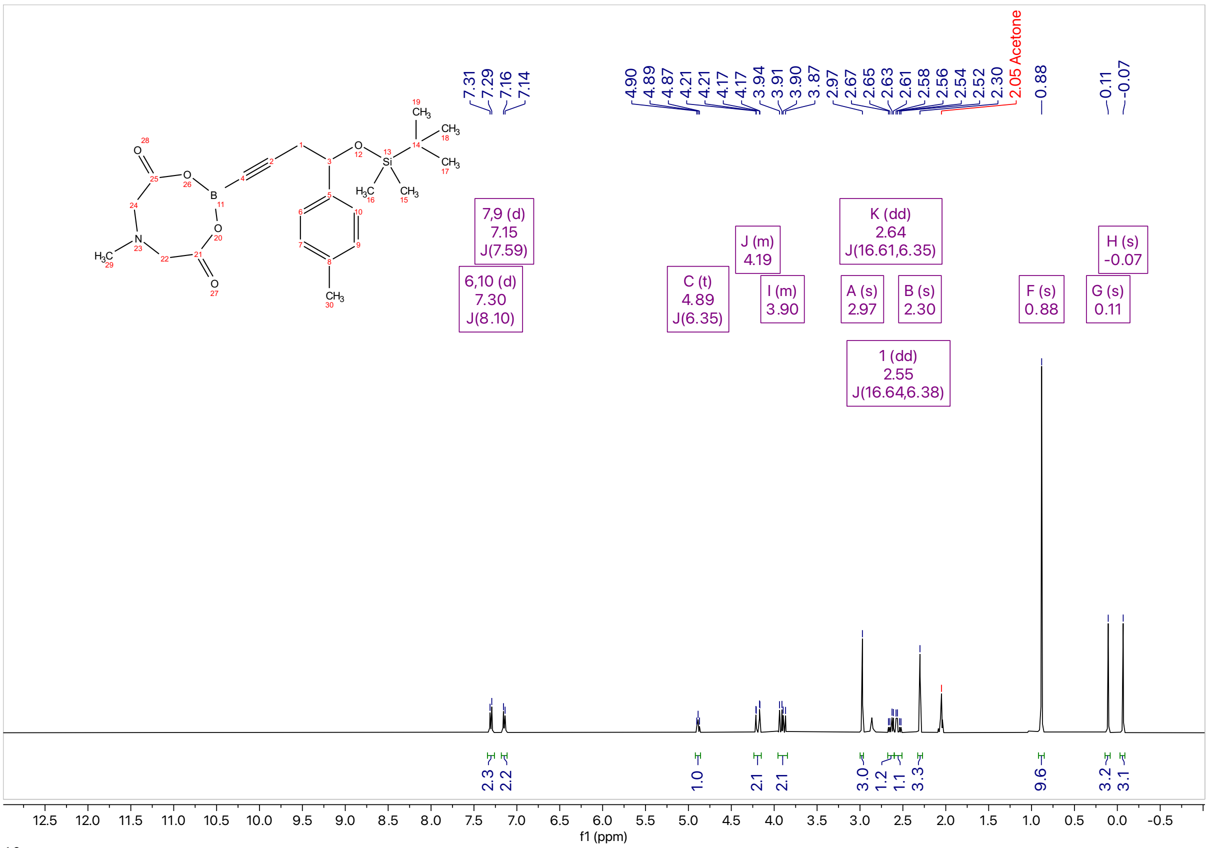
7.31
7.29
7.16
7.14
4.90
4.89
4.87
4.21
4.21
4.17
4.17
3.94
3.91
3.90
3.87
2.97
2.67
2.65
2.63
2.61
2.58
2.56
2.54
2.52
2.30
2.05 Acetone
0.88
0.11
-0.07
7,9 (d) 7.15 J(7.59)
6,10 (d) 7.30 J(8.10)
C (t) 4.89 J(6.35)
J (m) 4.19
I (m) 3.90
K (dd) 2.64 J(16.61,6.35)
A (s) 2.97
B (s) 2.30
1 (dd) 2.55 J(16.64,6.38)
F (s) 0.88
H (s) -0.07
G (s) 0.11
2.3
2.2
1.0
2.1
2.1
3.0
1.2
1.1
3.3
9.6
3.2
3.1
f1 (ppm)

^{13}C

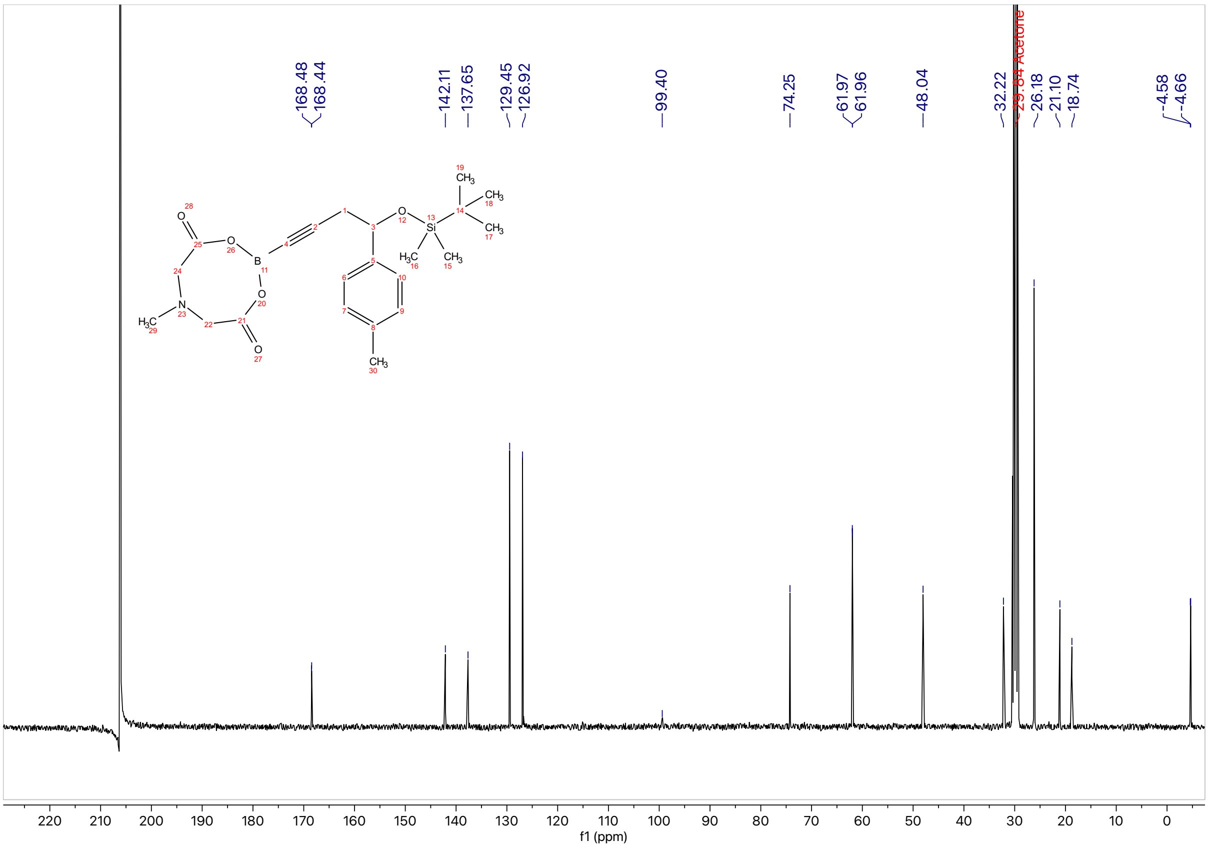
168.48
168.44
142.11
137.65
129.45
126.92
99.40
74.25
61.97
61.96
48.04
32.22
29.84 Acetone
26.18
21.10
18.74
-4.58
-4.66
f1 (ppm)

(S30)

^{1}H

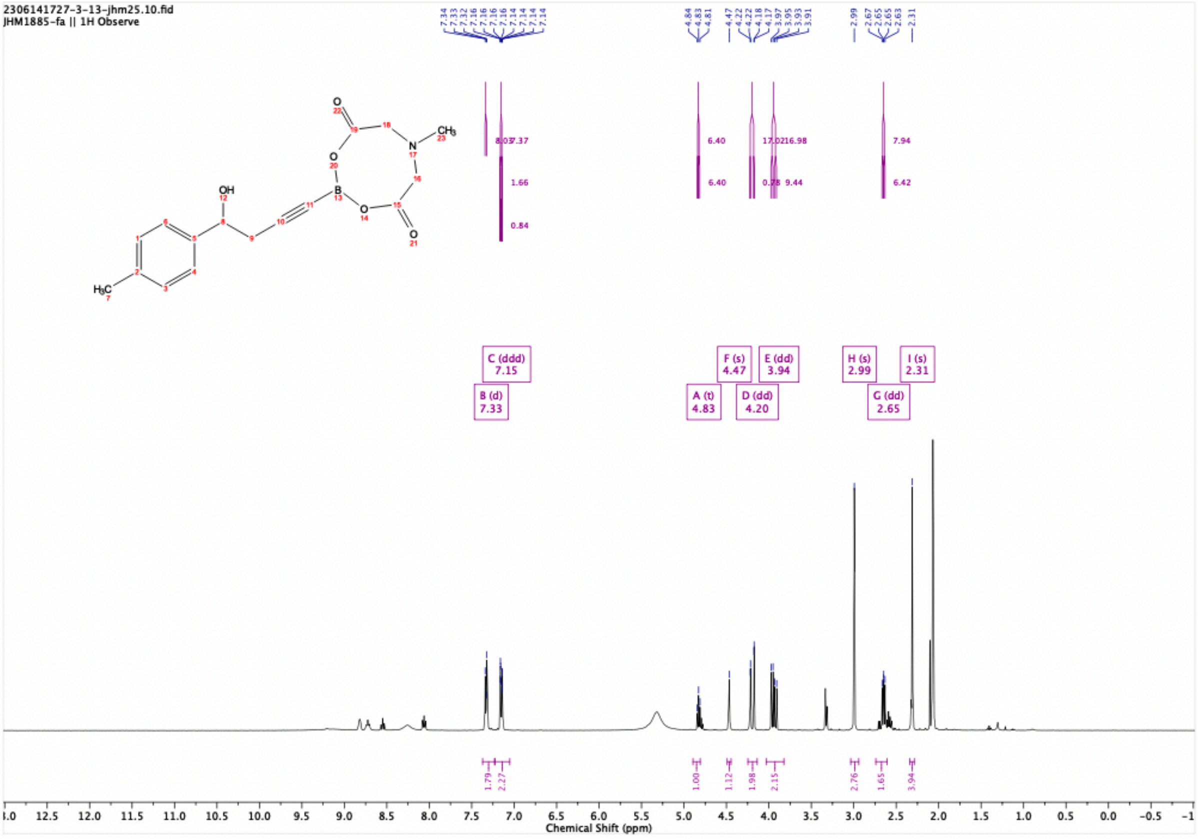

^{13}C DEPTQ

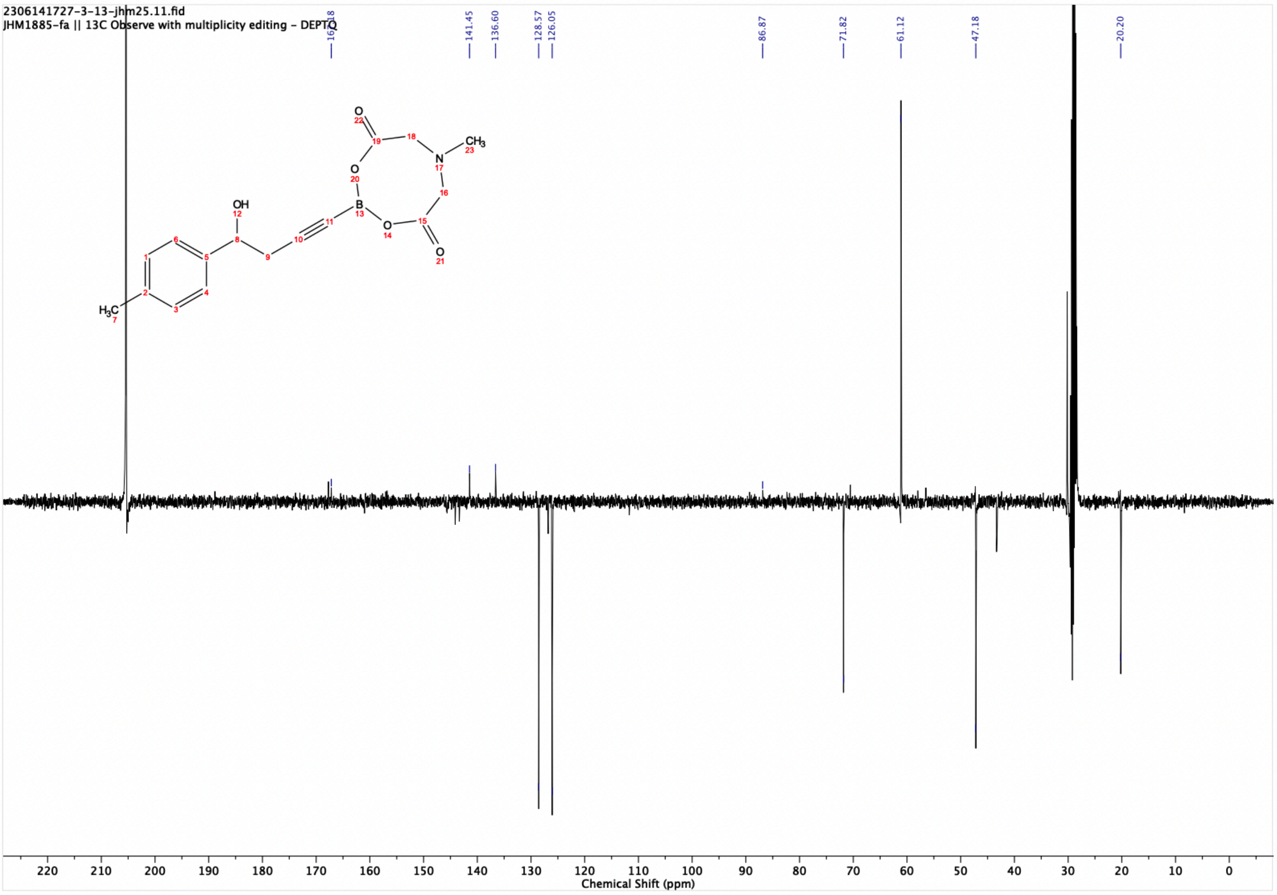

(S31)

^{1}H

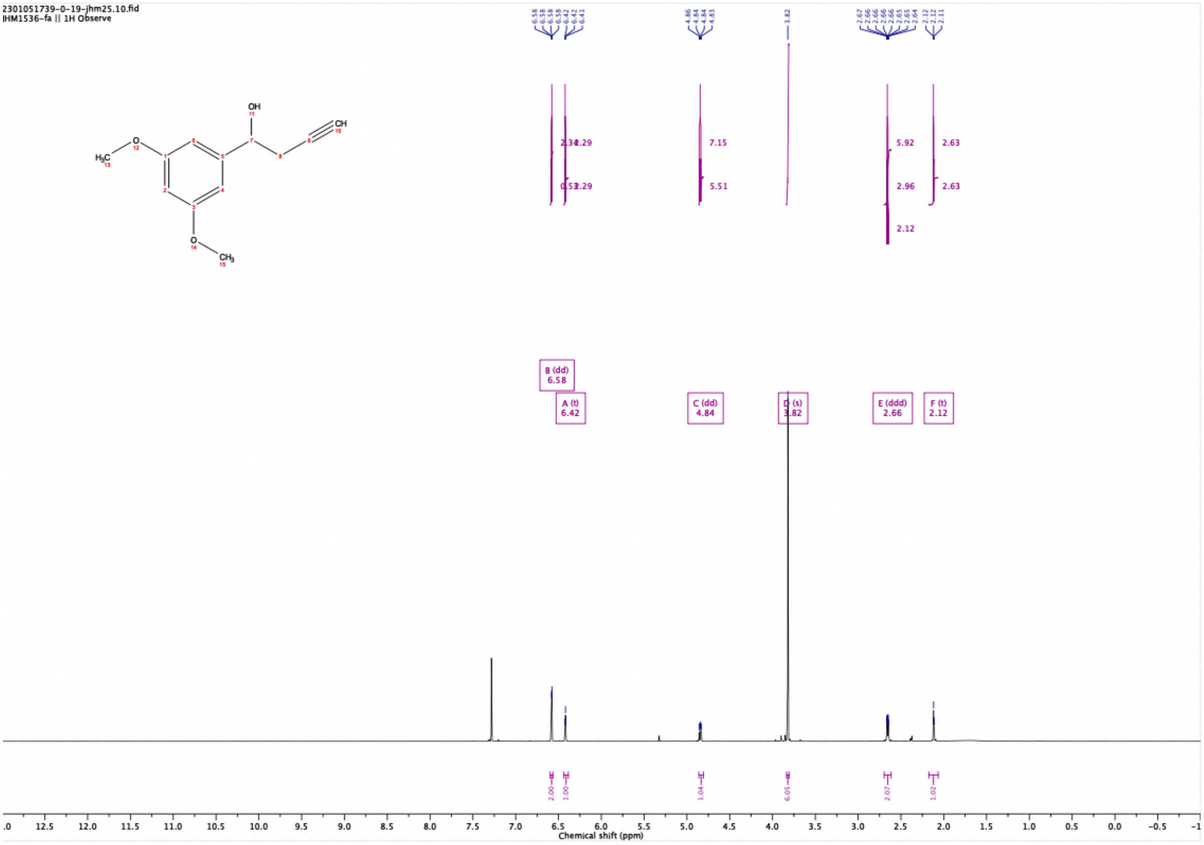

^{13}C DEPTQ

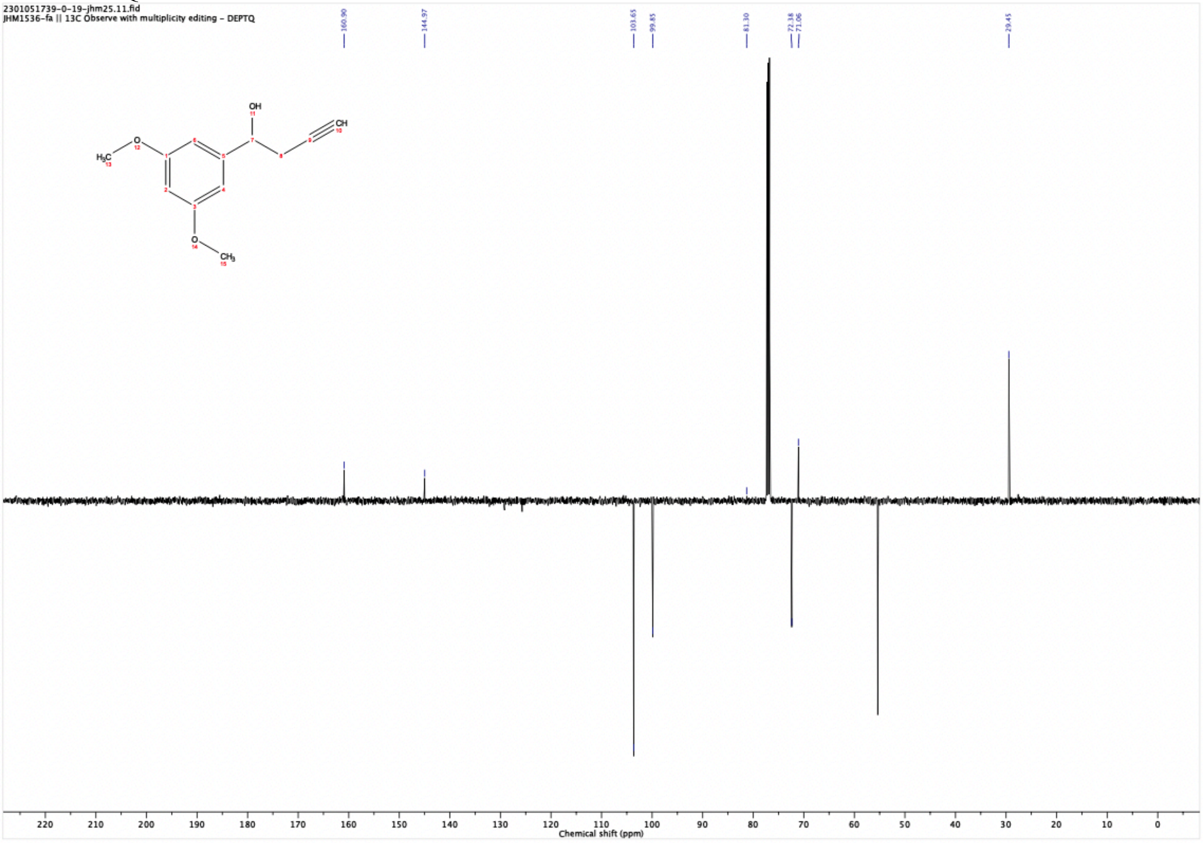

(S32)

^{1}H

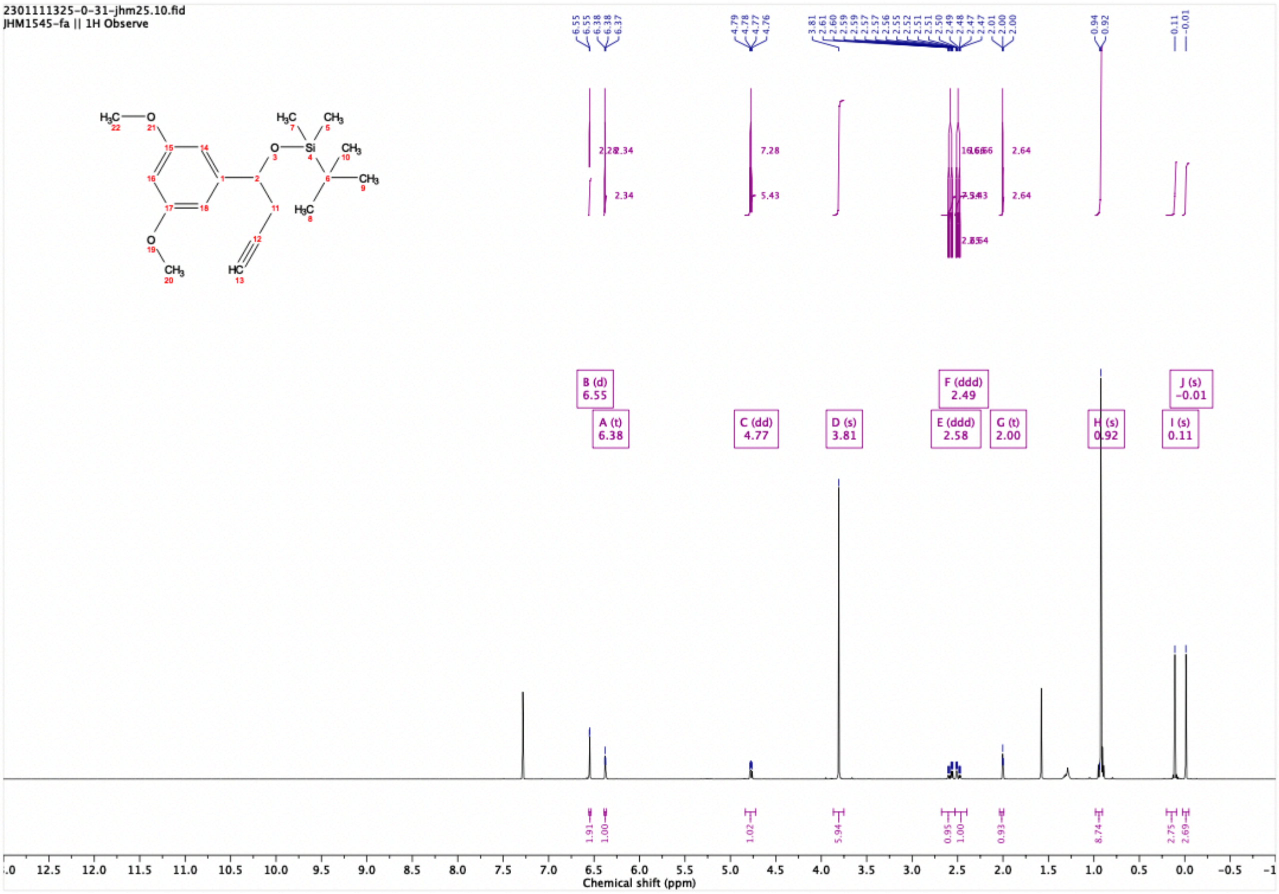

^{13}C DEPTQ

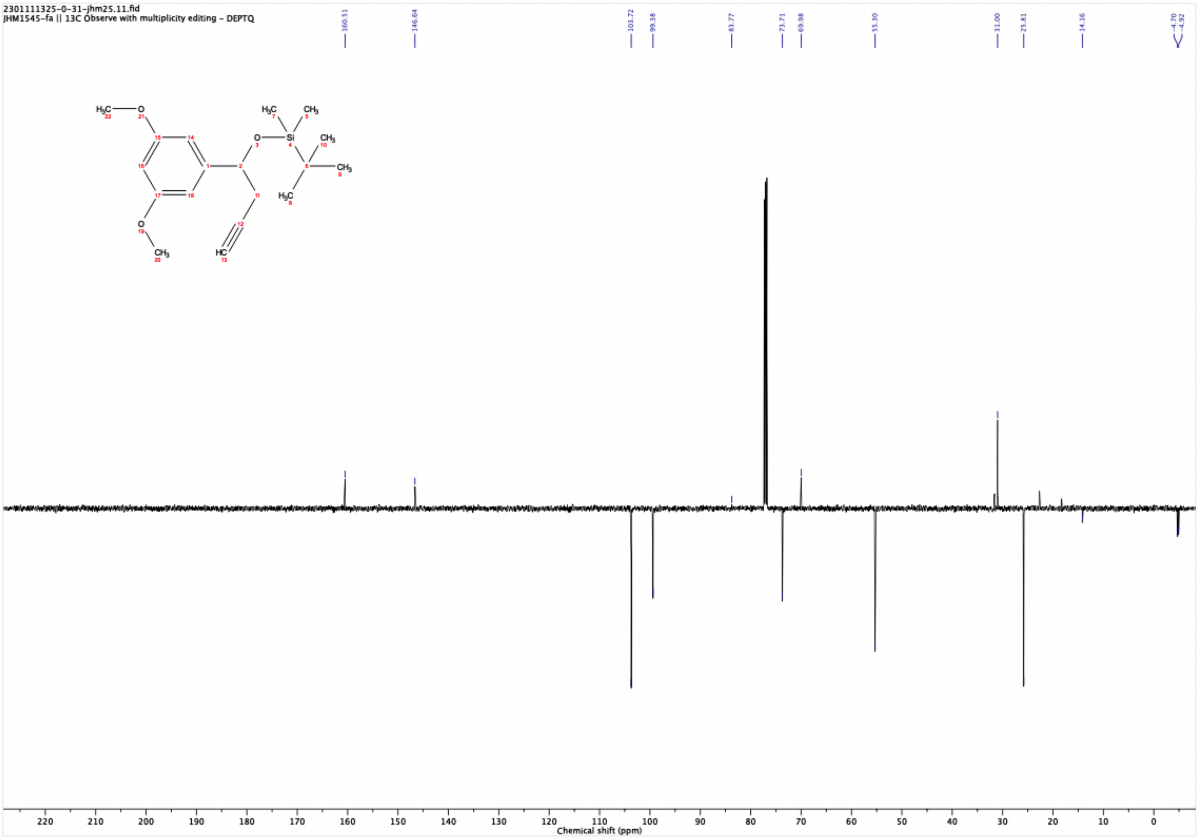

(S33)

^{1}H

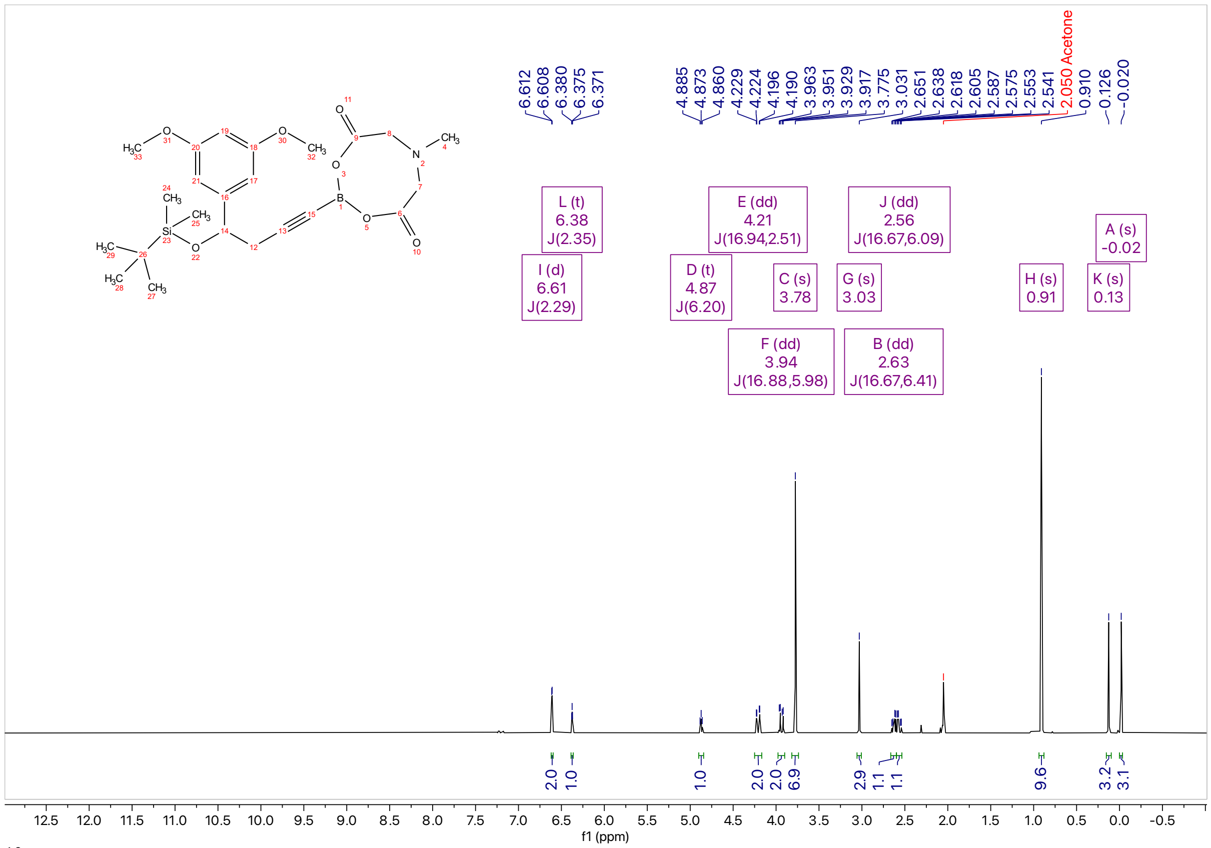
6.612
6.608
6.380
6.375
6.371
4.885
4.873
4.860
4.229
4.224
4.196
4.190
3.963
3.951
3.929
3.917
3.775
3.031
2.651
2.638
2.618
2.605
2.587
2.575
2.553
2.541
2.050 Acetone
0.910
0.126
-0.020
L (t) 6.38 J(2.35)
I (d) 6.61 J(2.29)
D (t) 4.87 J(6.20)
E (dd) 4.21 J(16.94,2.51)
F (dd) 3.94 J(16.88,5.98)
C (s) 3.78
G (s) 3.03
J (dd) 2.56 J(16.67,6.09)
B (dd) 2.63 J(16.67,6.41)
H (s) 0.91
A (s) -0.02
K (s) 0.13
2.0
1.0
1.0
2.0
2.0
6.9
2.9
1.1
1.1
9.6
3.2
3.1
f1 (ppm)

^{13}C

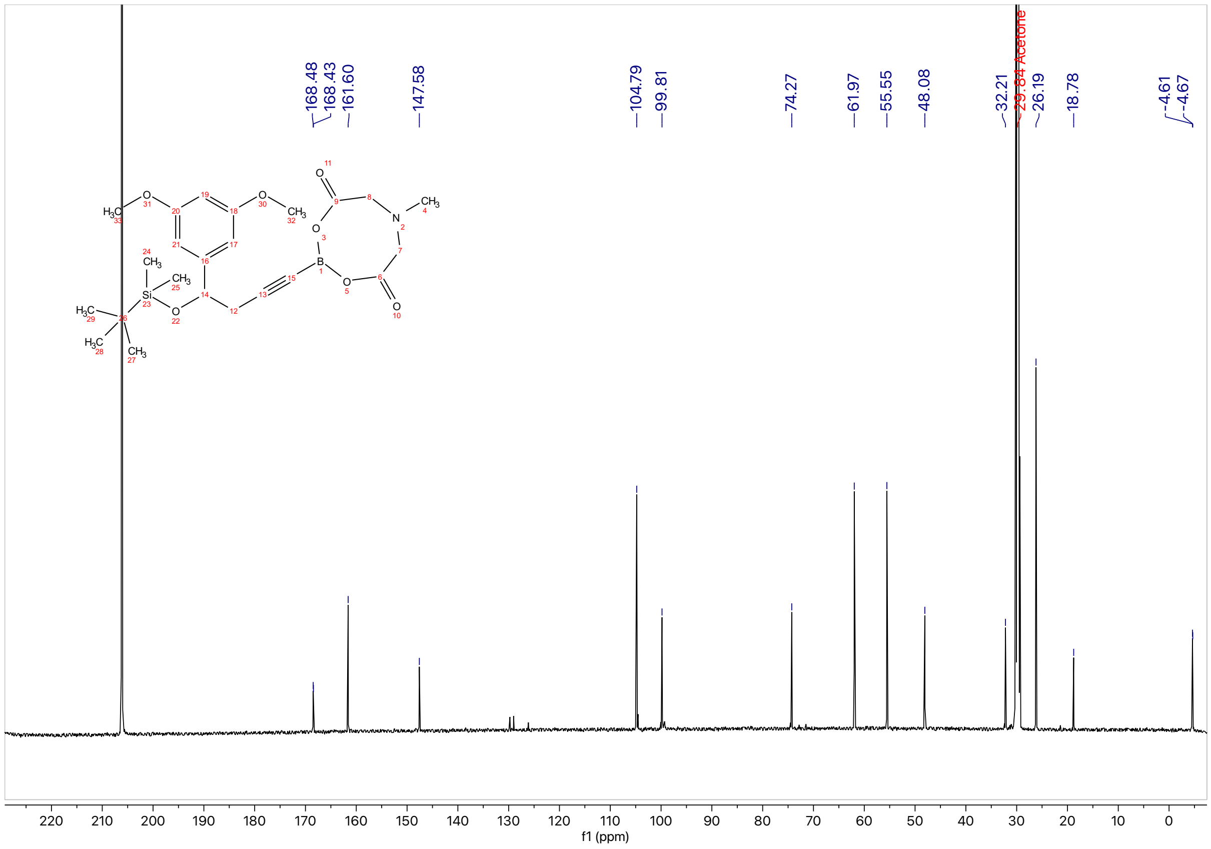
168.48
168.43
161.60
147.58
104.79
99.81
74.27
61.97
55.55
48.08
32.21
29.84 Acetone
26.19
18.78
-4.61
-4.67
f1 (ppm)

(S34)

^{1}H

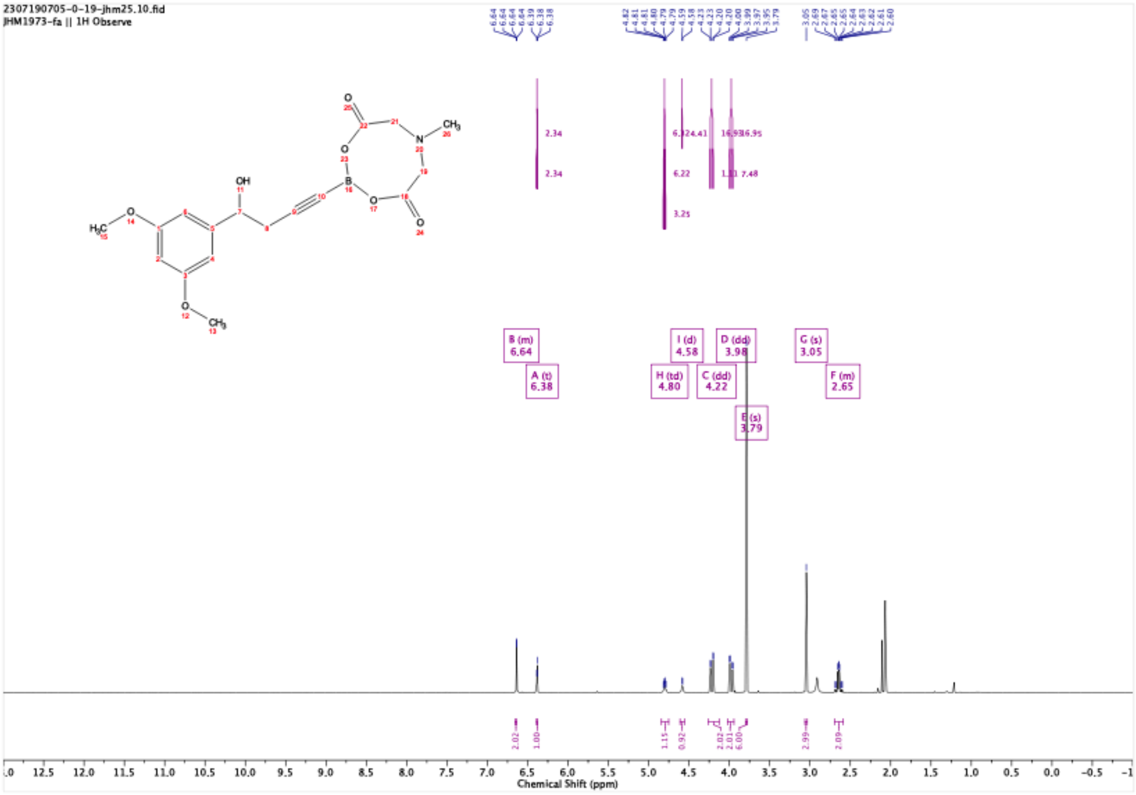

^{13}C DEPTQ

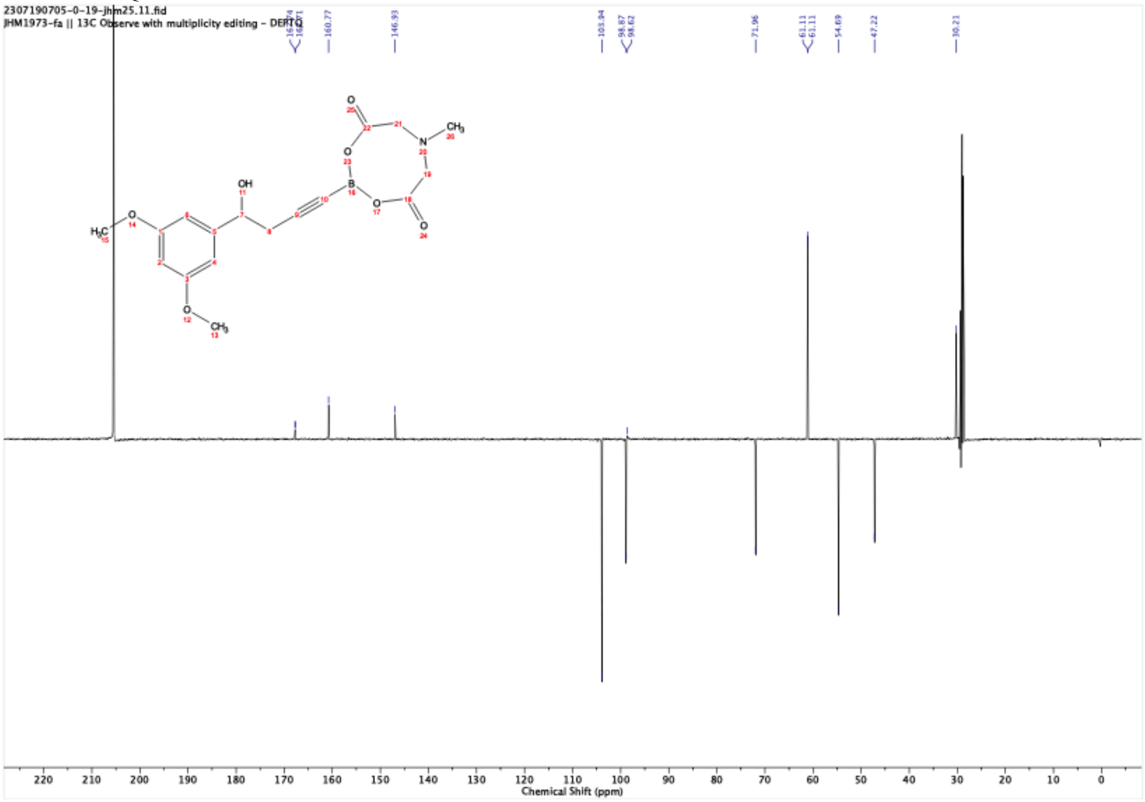

(S35)

^{1}H

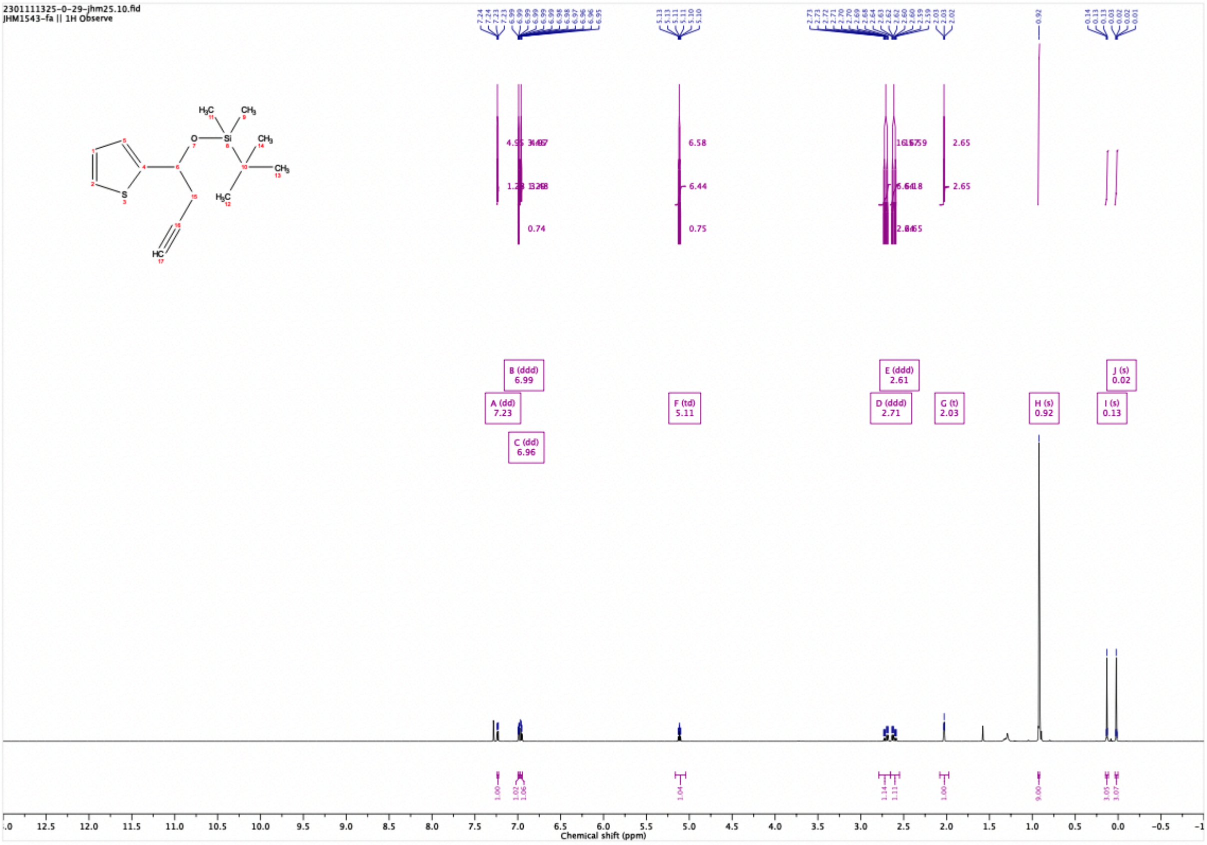

^{13}C DEPTQ

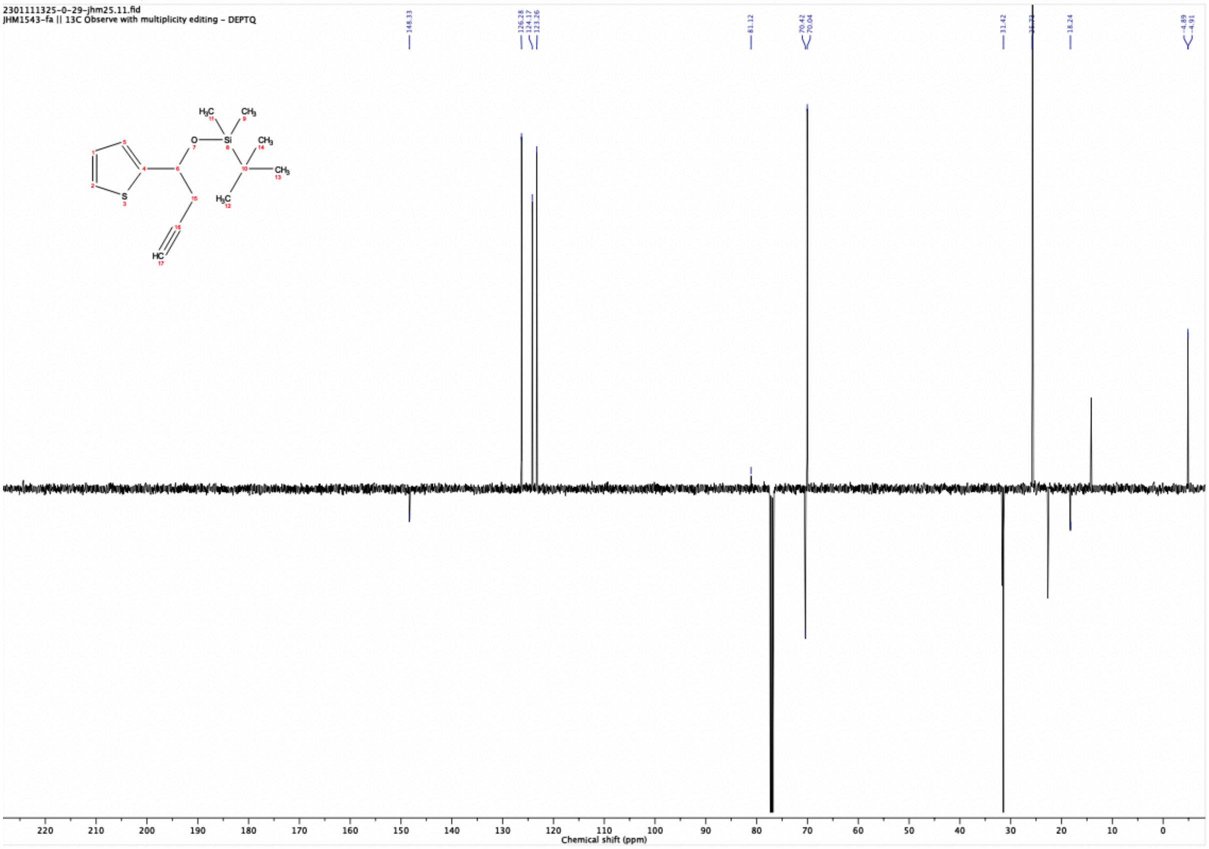

(S36)

^{1}H

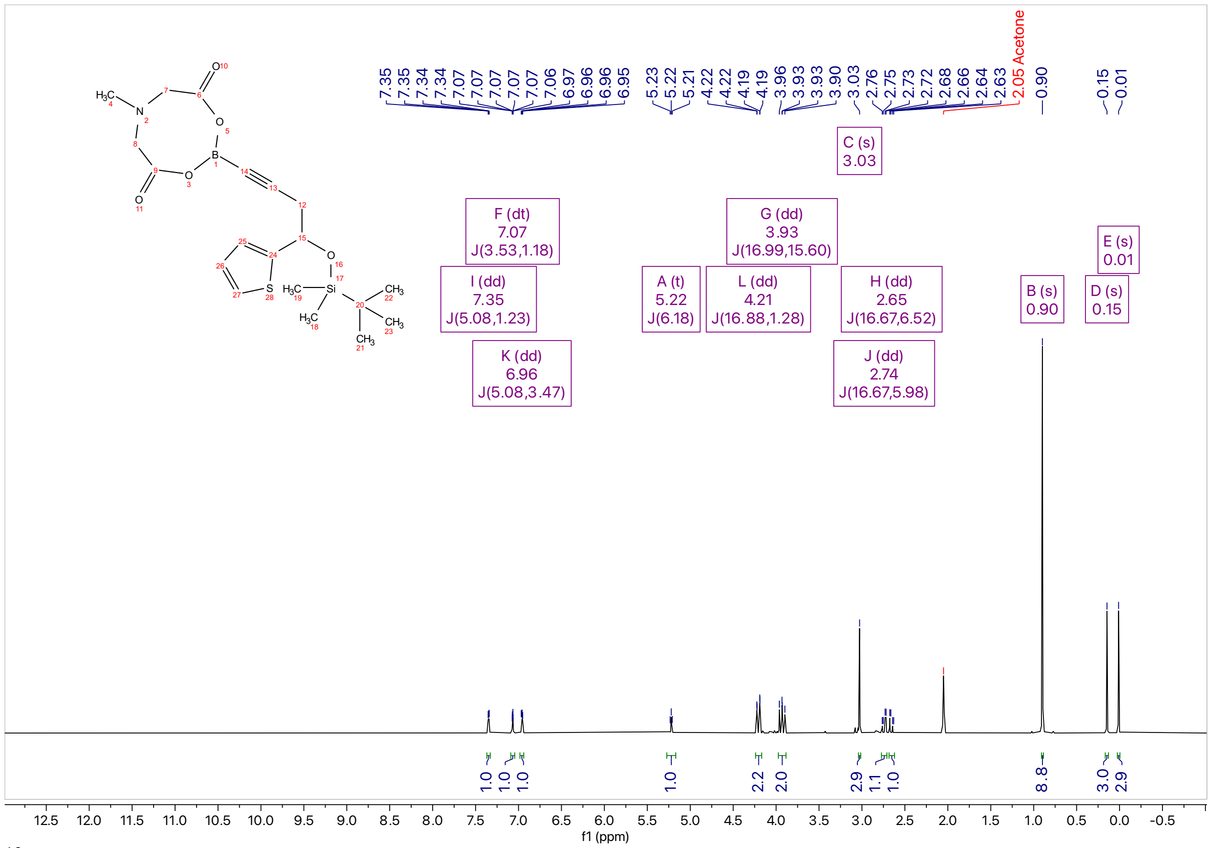

7.35
7.35
7.34
7.34
7.07
7.07
7.07
7.07
7.07
7.06
6.97
6.96
6.96
6.95
5.23
5.22
5.21
4.22
4.22
4.19
4.19
3.96
3.93
3.93
3.90
3.03
2.76
2.75
2.73
2.72
2.68
2.66
2.64
2.63
2.05 Acetone
0.90
0.15
0.01
C (s) 3.03
F (dt) 7.07 J(3.53,1.18)
G (dd) 3.93 J(16.99,15.60)
E (s) 0.01
I (dd) 7.35 J(5.08,1.23)
A (t) 5.22 J(6.18)
L (dd) 4.21 J(16.88,1.28)
H (dd) 2.65 J(16.67,6.52)
B (s) 0.90
D (s) 0.15
K (dd) 6.96 J(5.08,3.47)
J (dd) 2.74 J(16.67,5.98)
1.0
1.0
1.0
1.0
2.2
2.0
2.9
1.1
1.0
8.8
3.0
2.9
f1 (ppm)

^{13}C

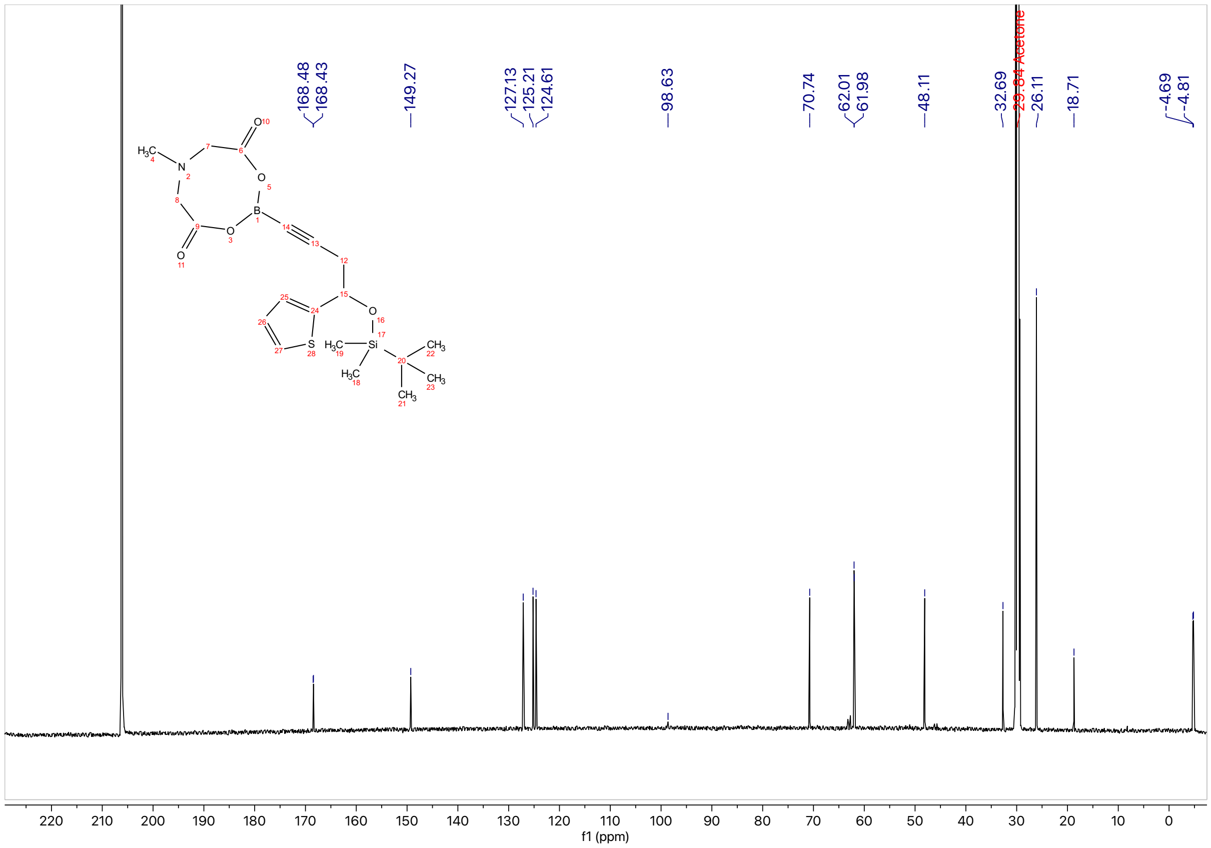

168.48
168.43
149.27
127.13
125.21
124.61
98.63
70.74
62.01
61.98
48.11
32.69
29.84 Acetone
26.11
18.71
-4.69
-4.81
f1 (ppm)

(S38)

^{1}H

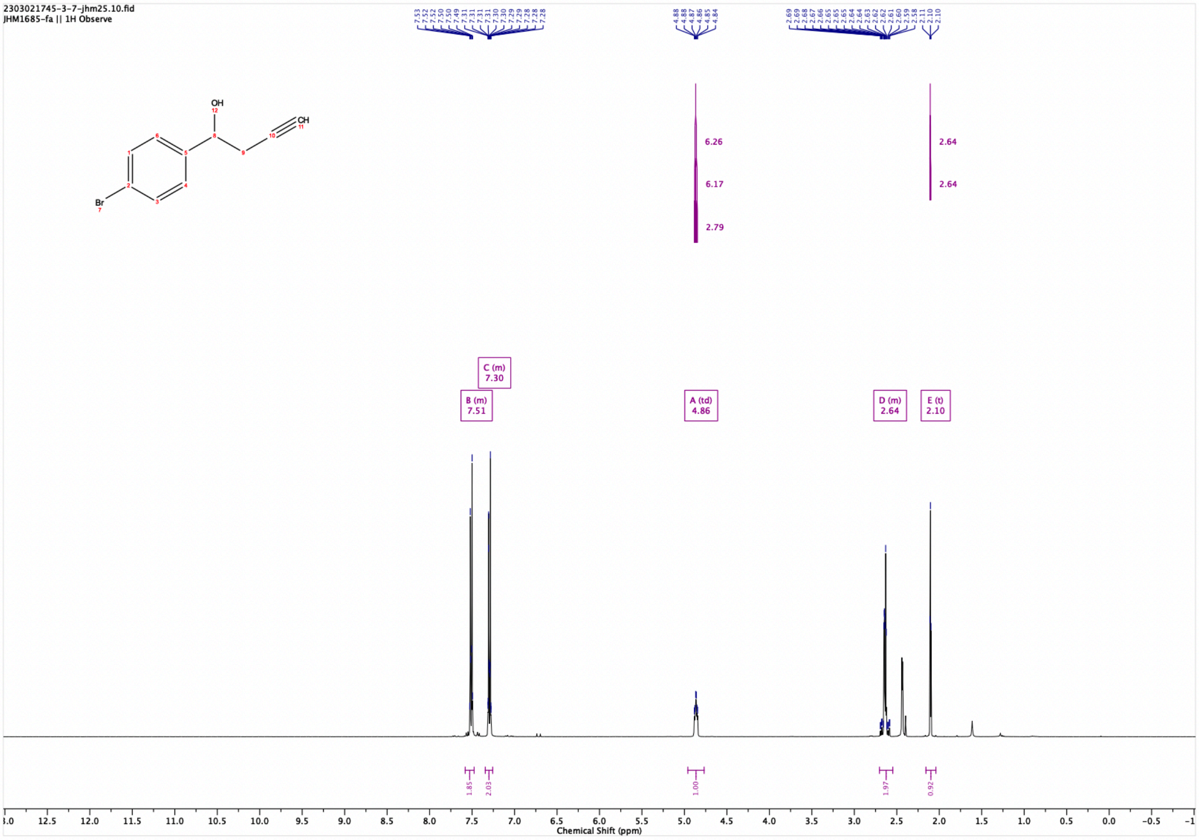

^{13}C DEPTQ

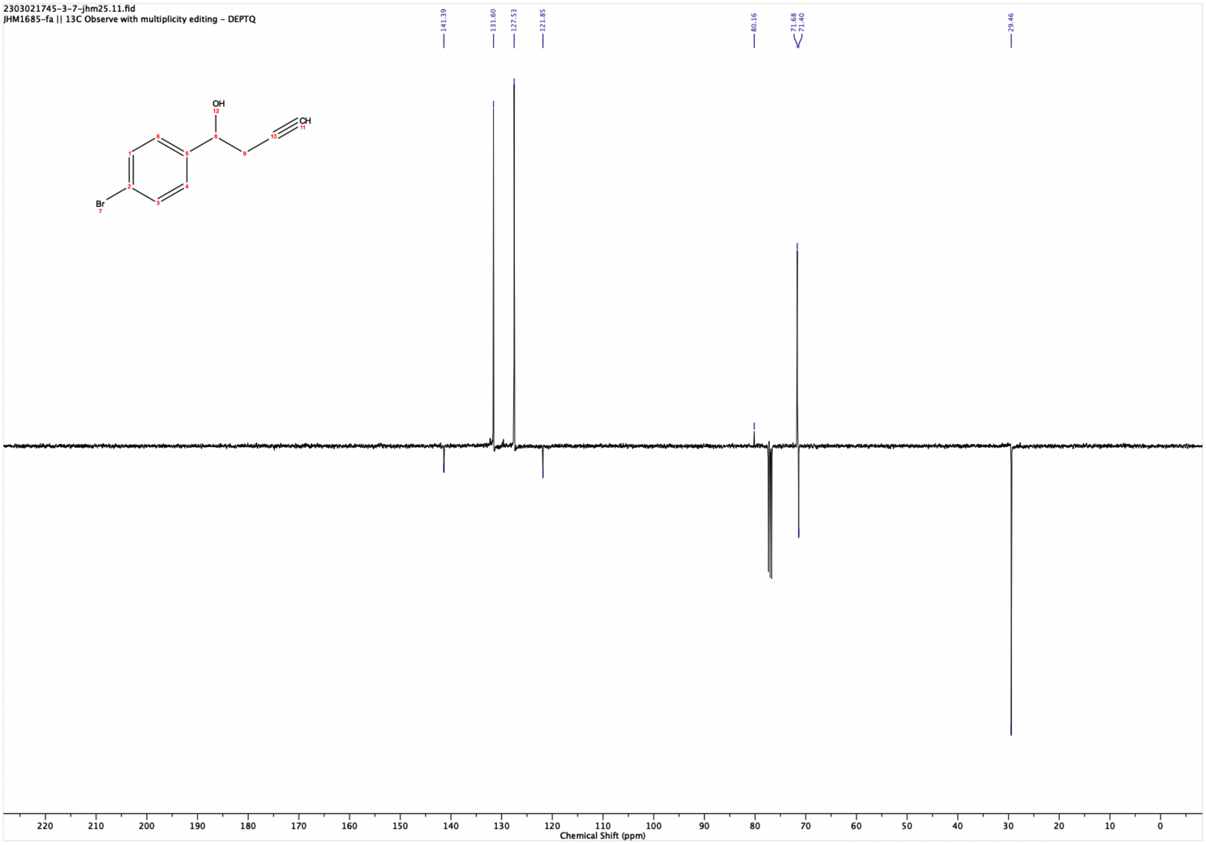

(S39)

^{1}H

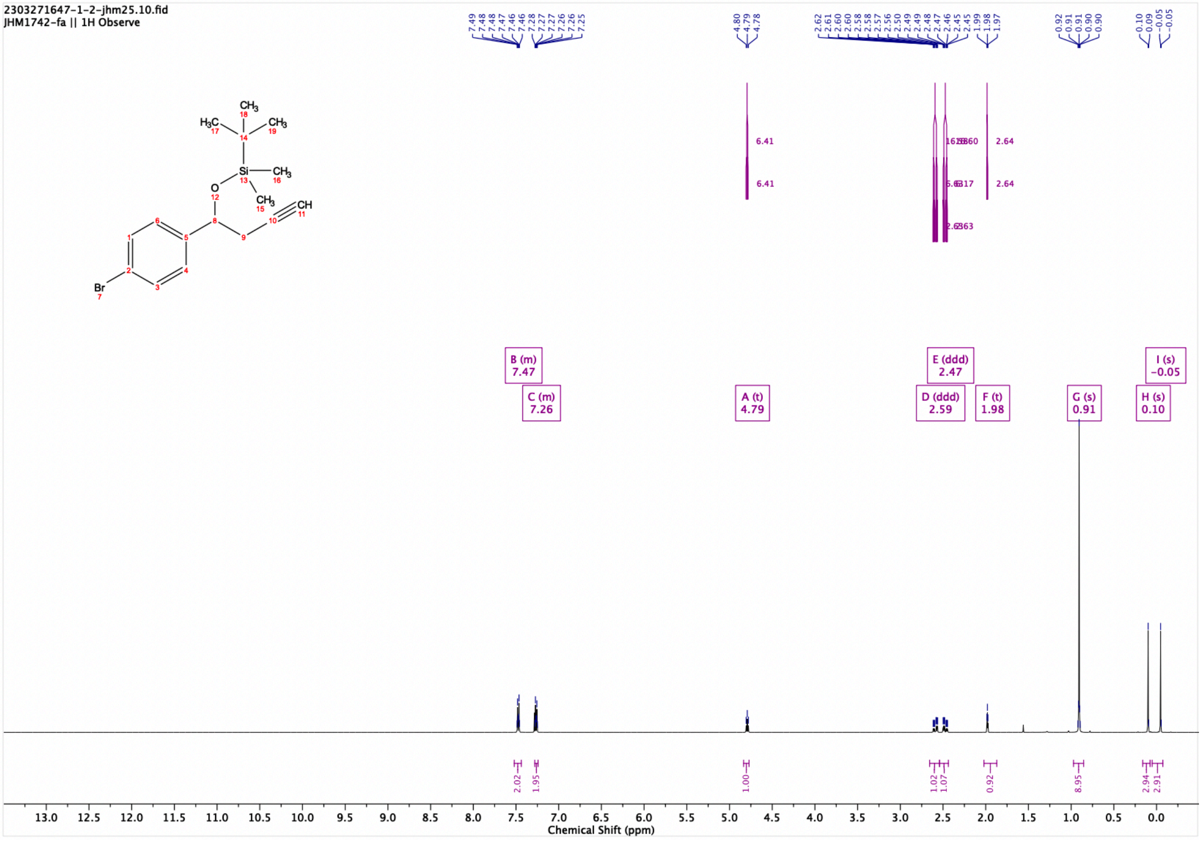

^{13}C DEPTQ

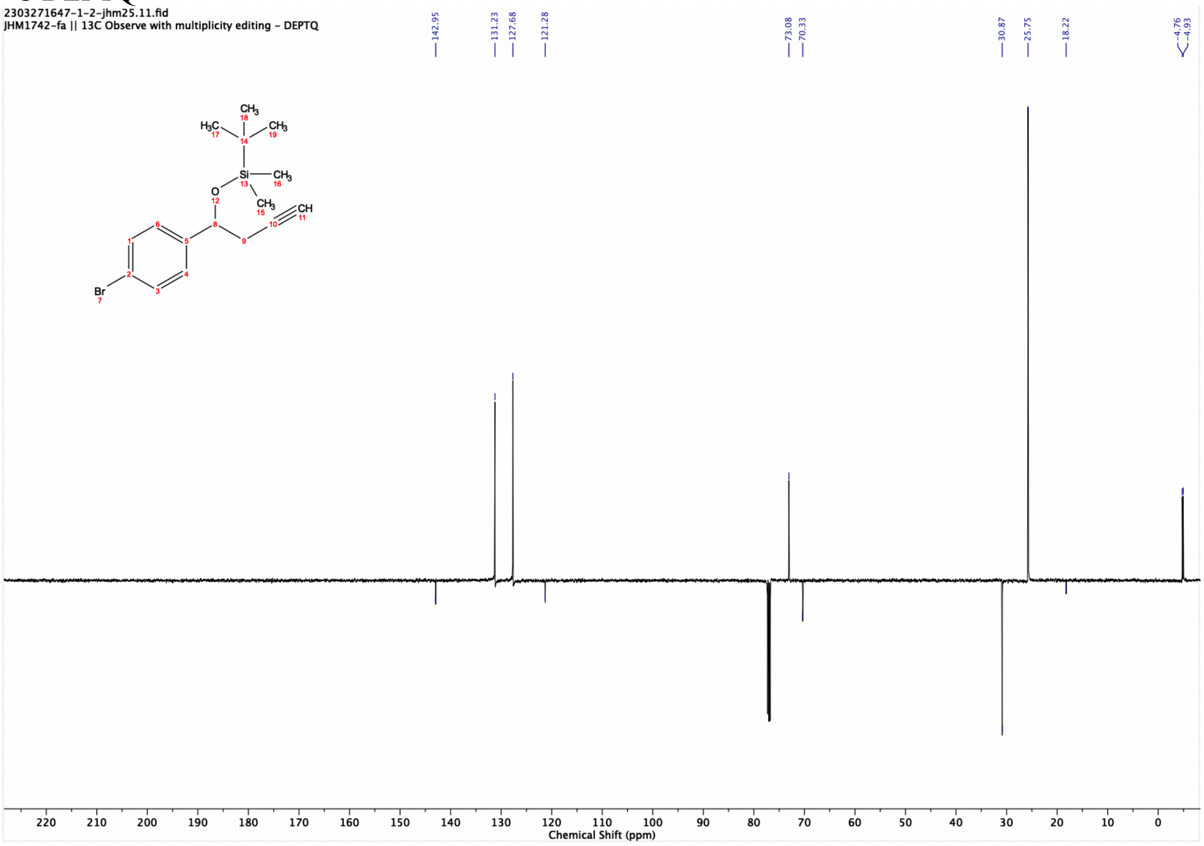

(S40)

^{1}H

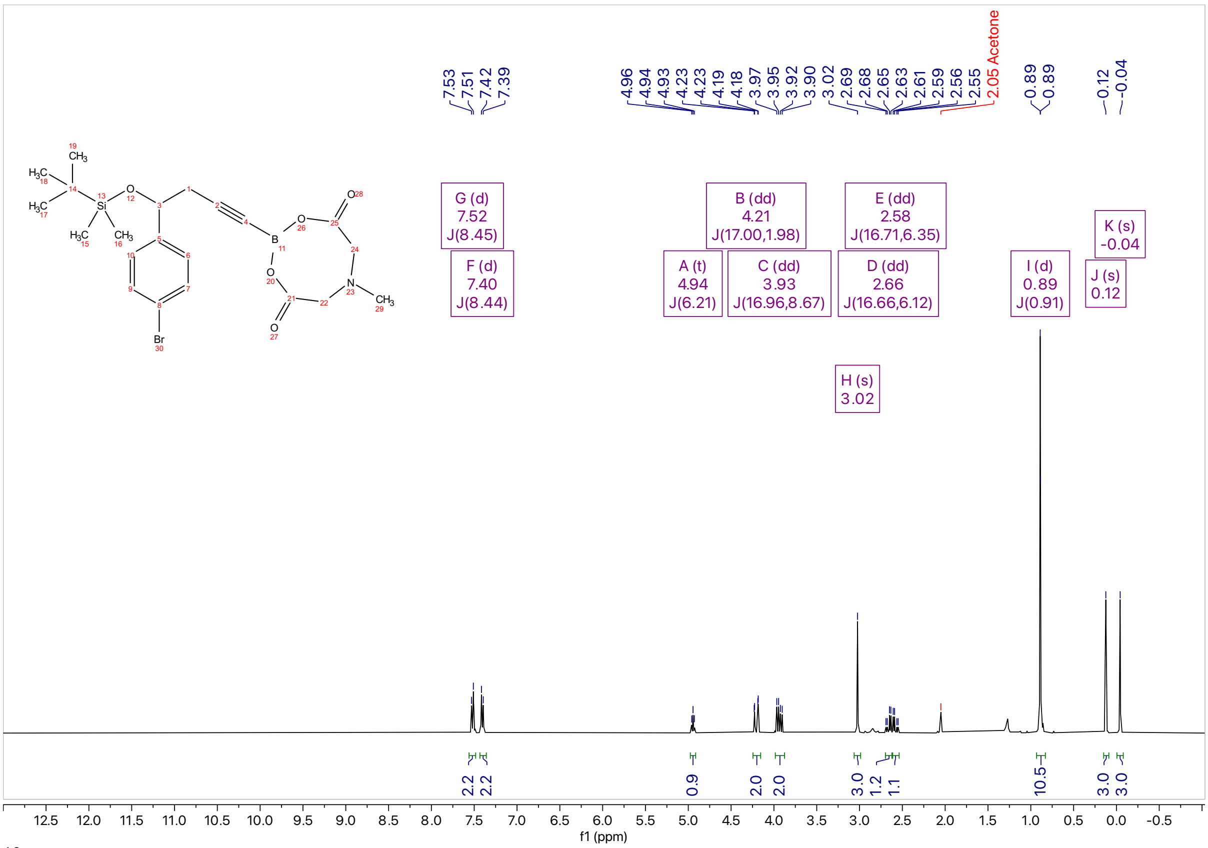
7.53
7.51
7.42
7.39
4.96
4.94
4.93
4.23
4.23
4.19
4.18
3.97
3.95
3.92
3.90
3.02
2.69
2.68
2.65
2.63
2.61
2.59
2.56
2.55
2.05 Acetone
0.89
0.89
0.12
-0.04
G (d) 7.52 J(8.45)
F (d) 7.40 J(8.44)
B (dd) 4.21 J(17.00,1.98)
A (t) 4.94 J(6.21)
C (dd) 3.93 J(16.96,8.67)
E (dd) 2.58 J(16.71,6.35)
D (dd) 2.66 J(16.66,6.12)
H (s) 3.02
I (d) 0.89 J(0.91)
J (s) 0.12
K (s) -0.04
2.2
2.2
0.9
2.0
2.0
3.0
1.2
1.1
10.5
3.0
3.0
f1 (ppm)

^{13}C

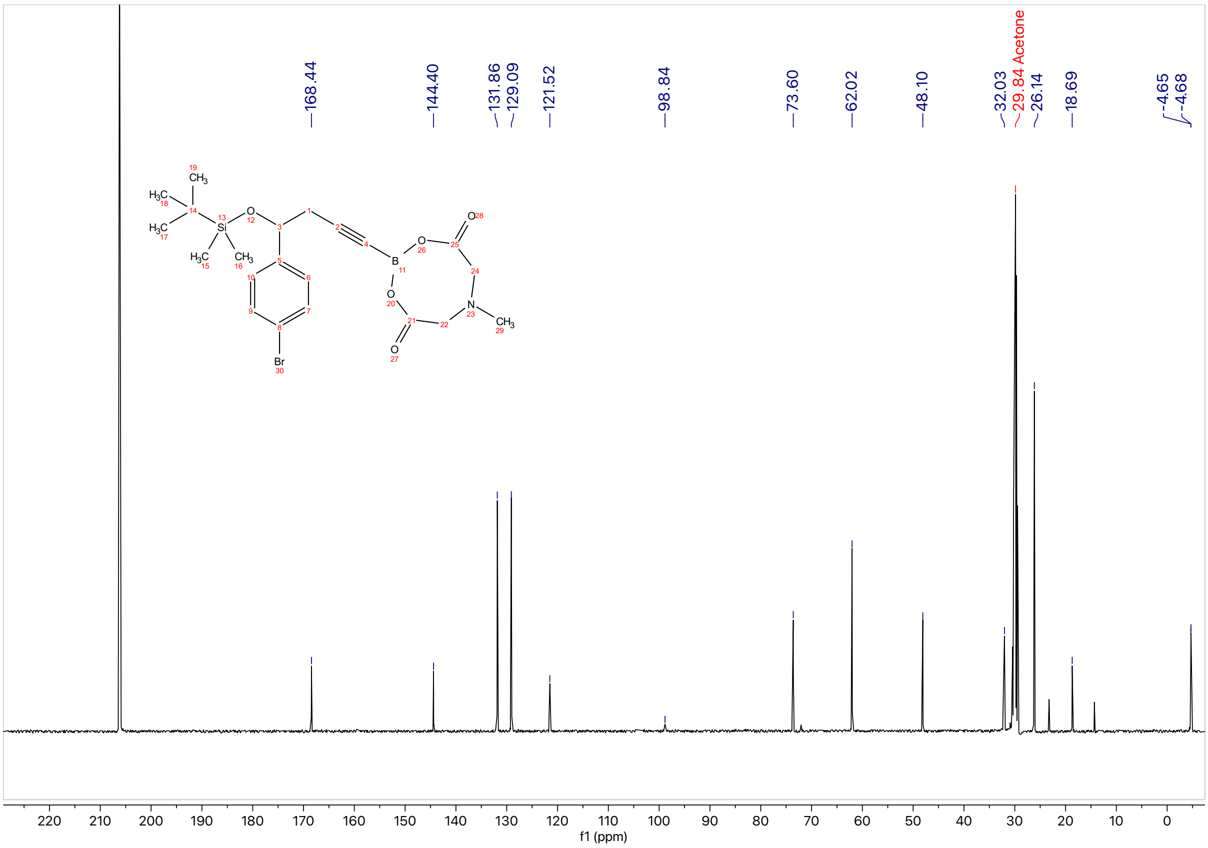
168.44
144.40
131.86
129.09
121.52
98.84
73.60
62.02
48.10
32.03
29.84 Acetone
26.14
18.69
-4.65
-4.68
f1 (ppm)

(S41)

^{1}H

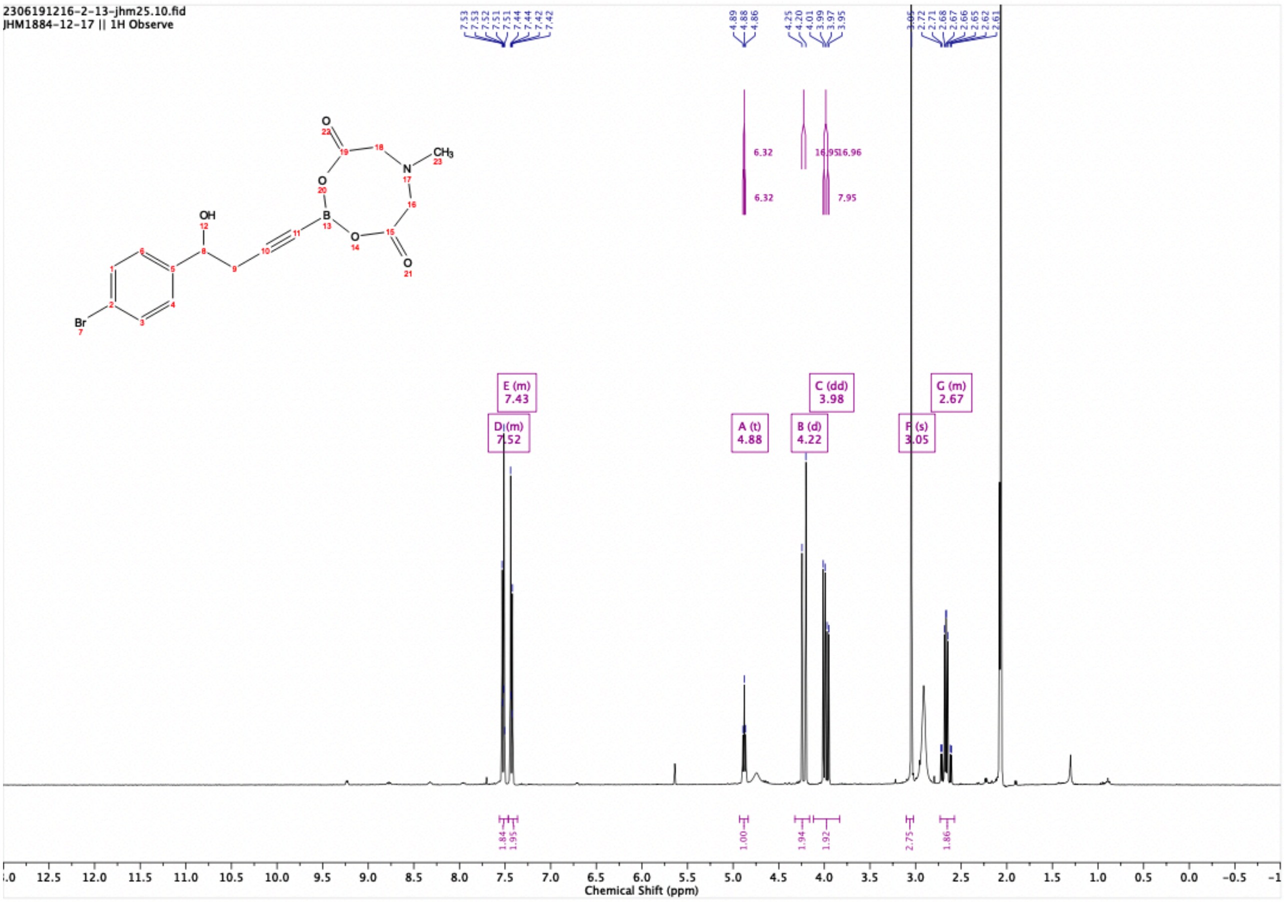

^{13}C DEPTQ

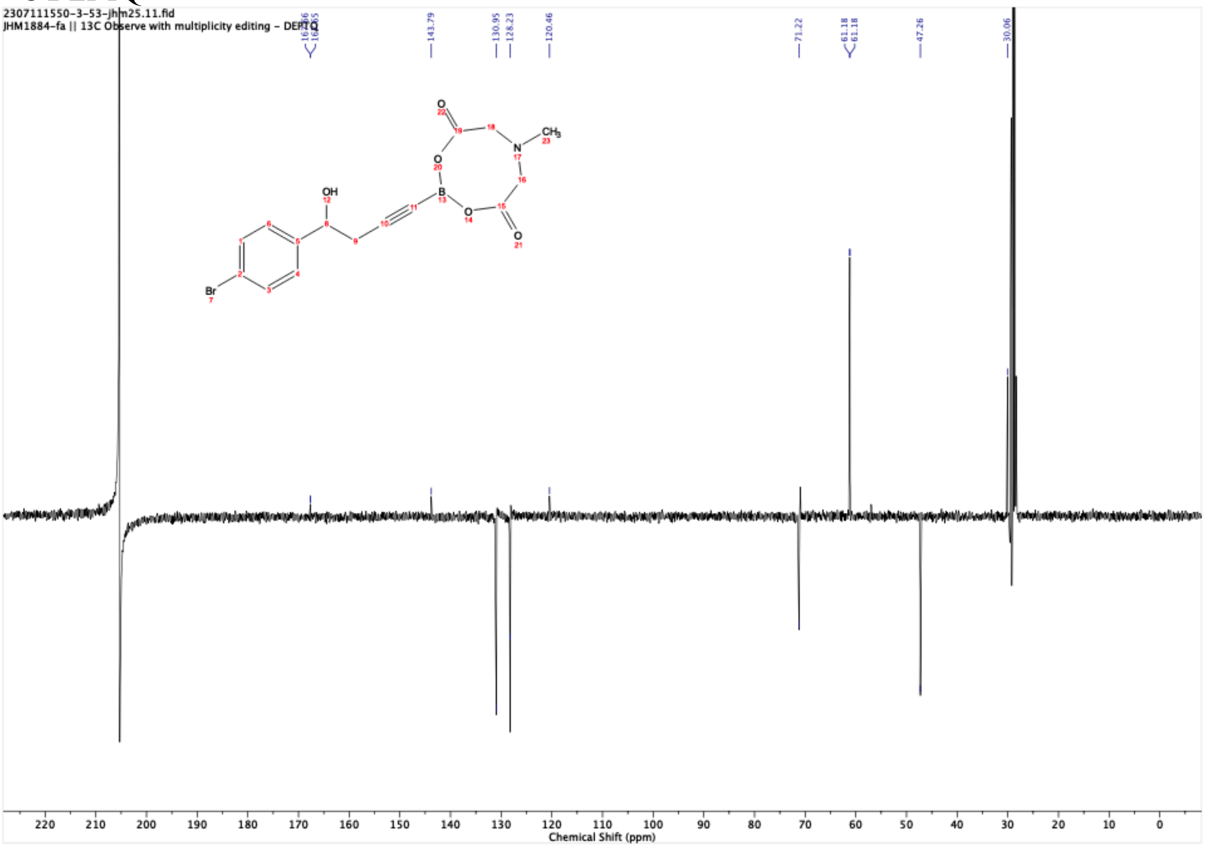

(S42)

^{1}H

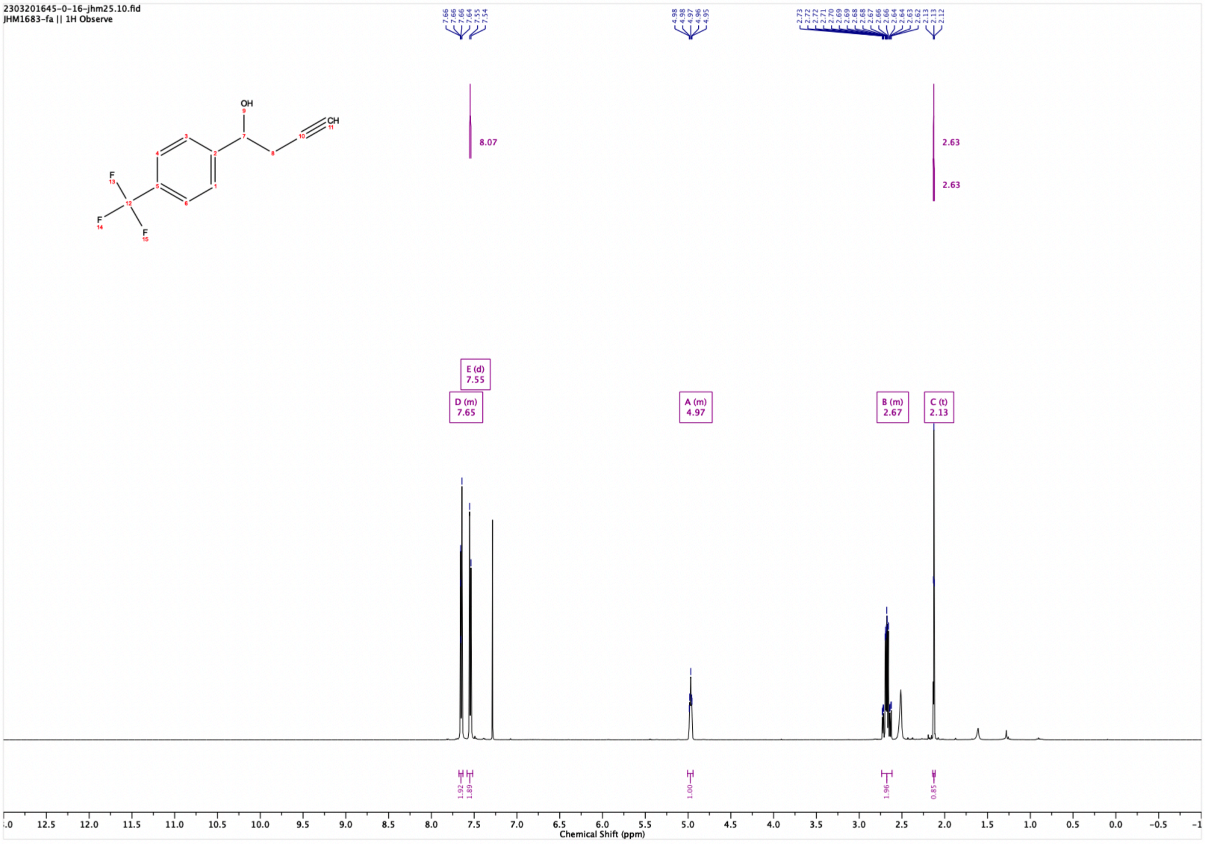

^{13}C DEPTQ

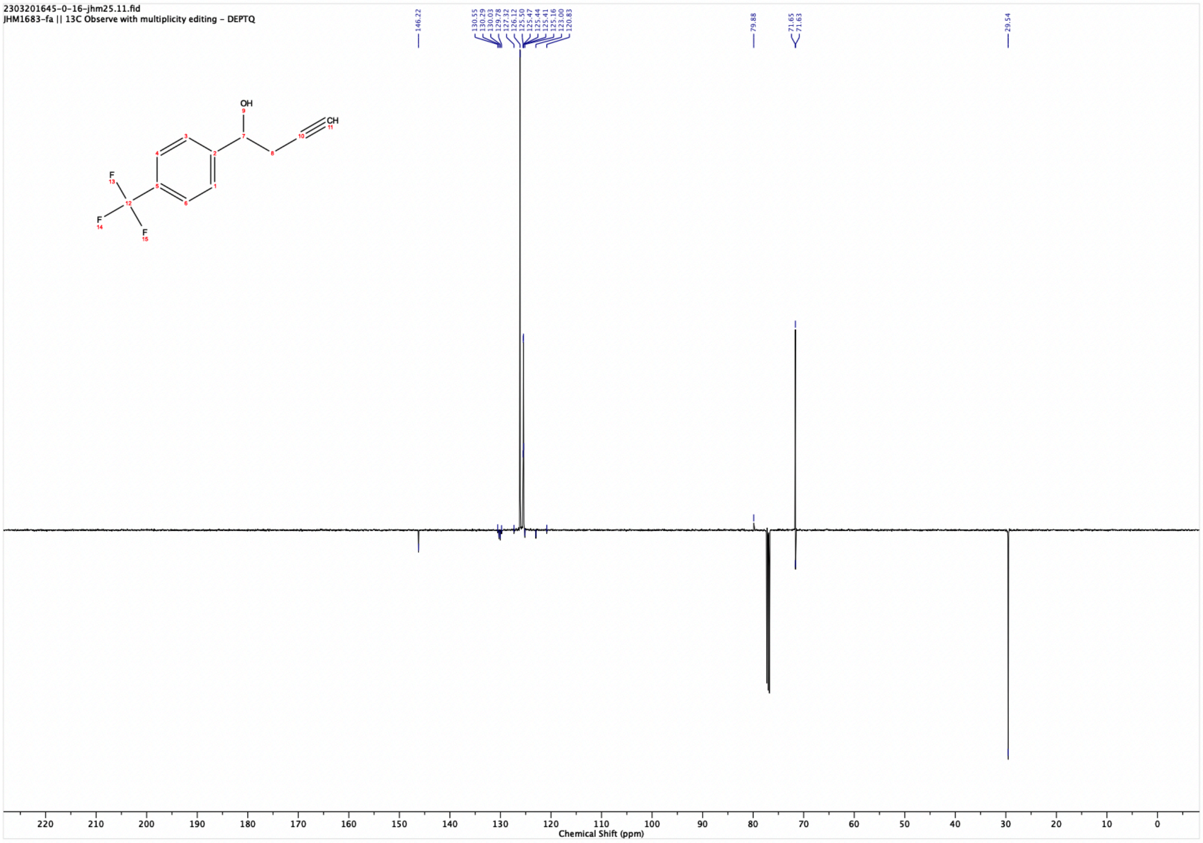

^{19}F

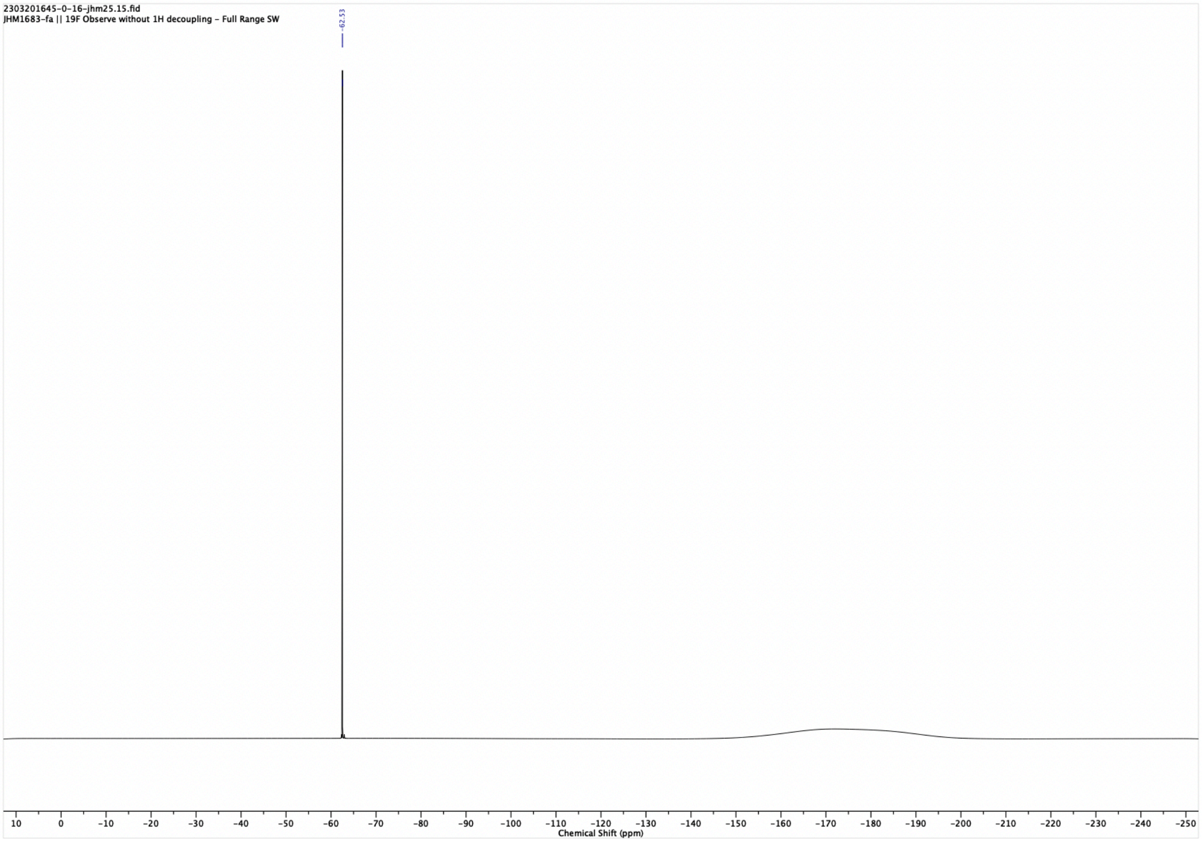

(S43)

^{1}H

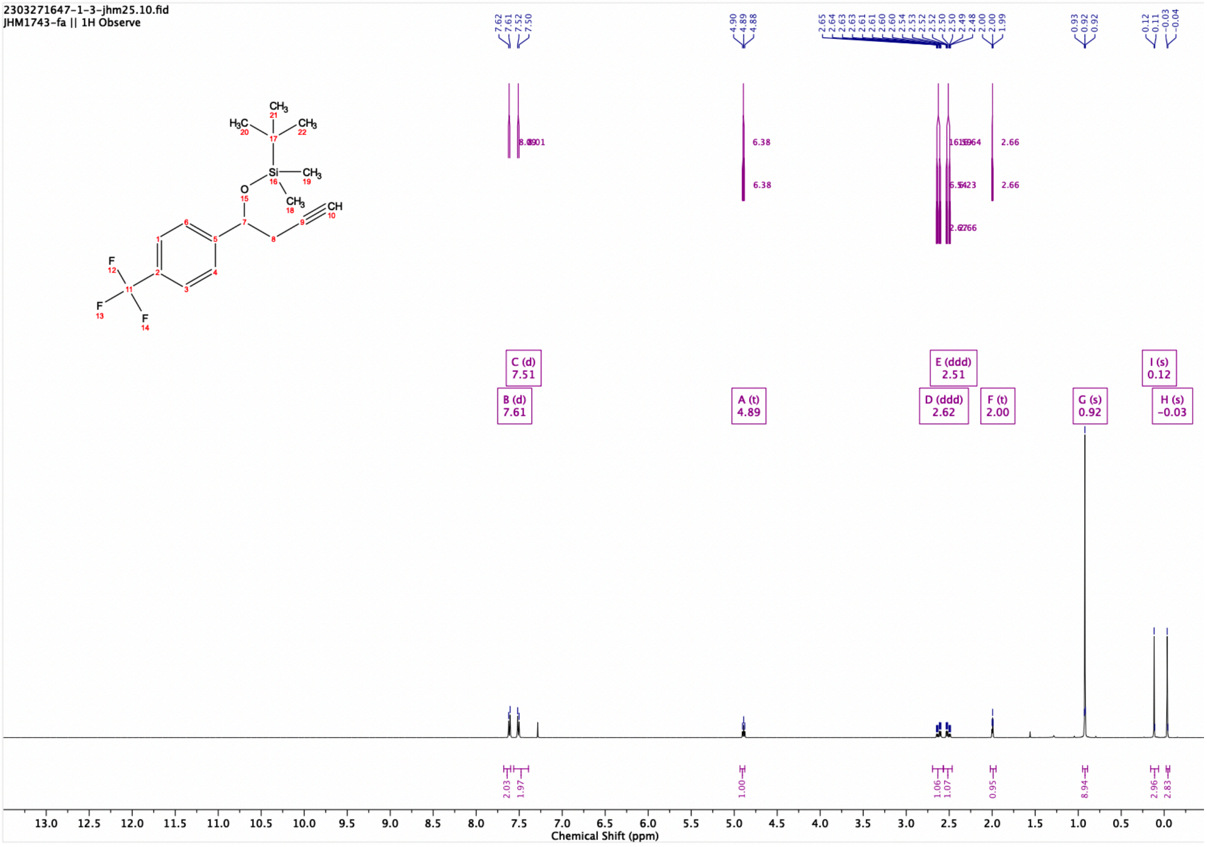

^{13}C DEPTQ

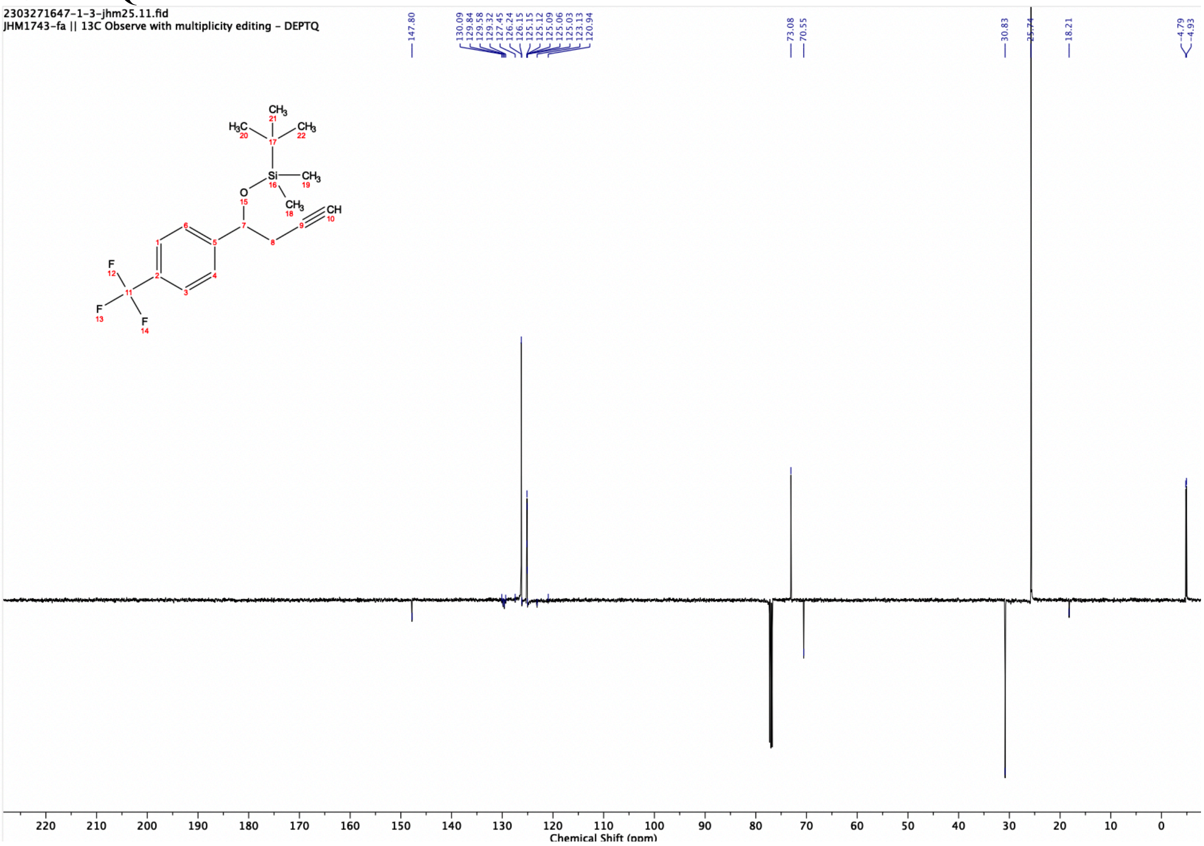

$^{19}F\{^{1}H\}$

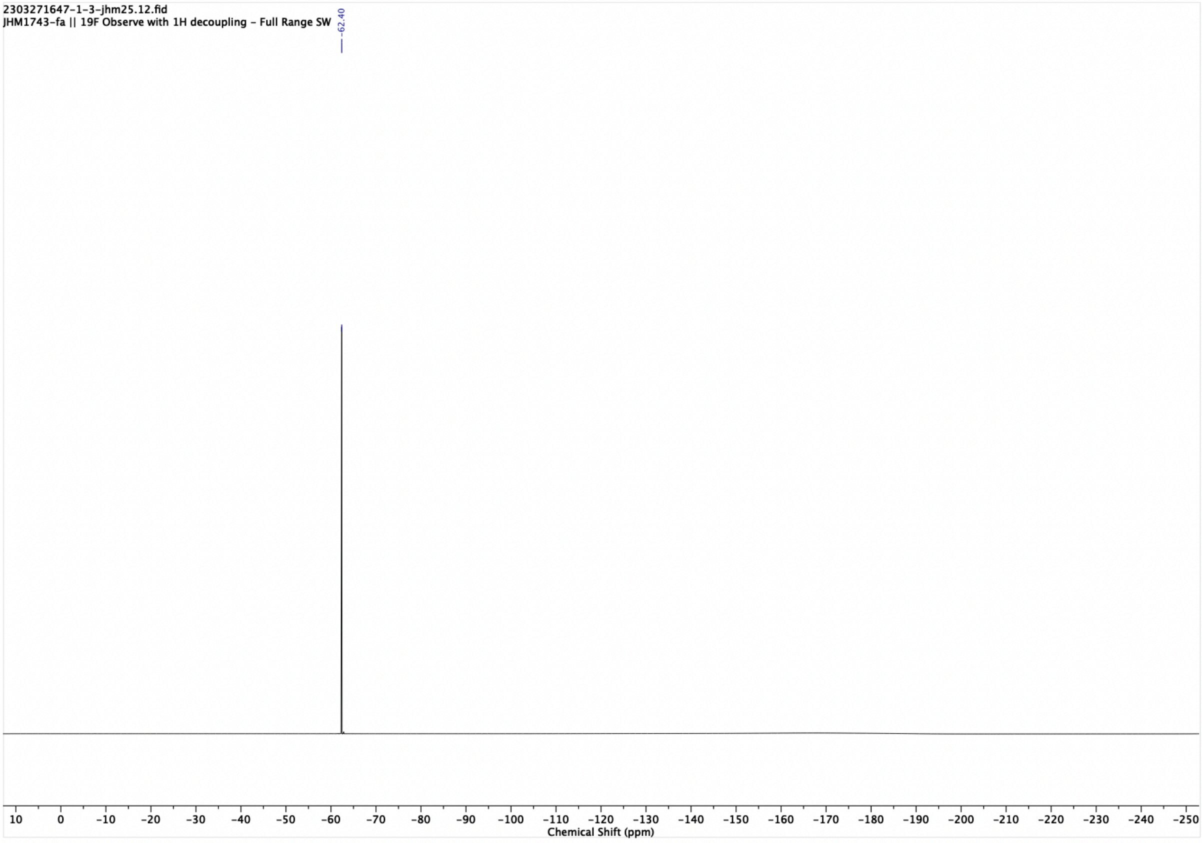

(S44)

^{1}H

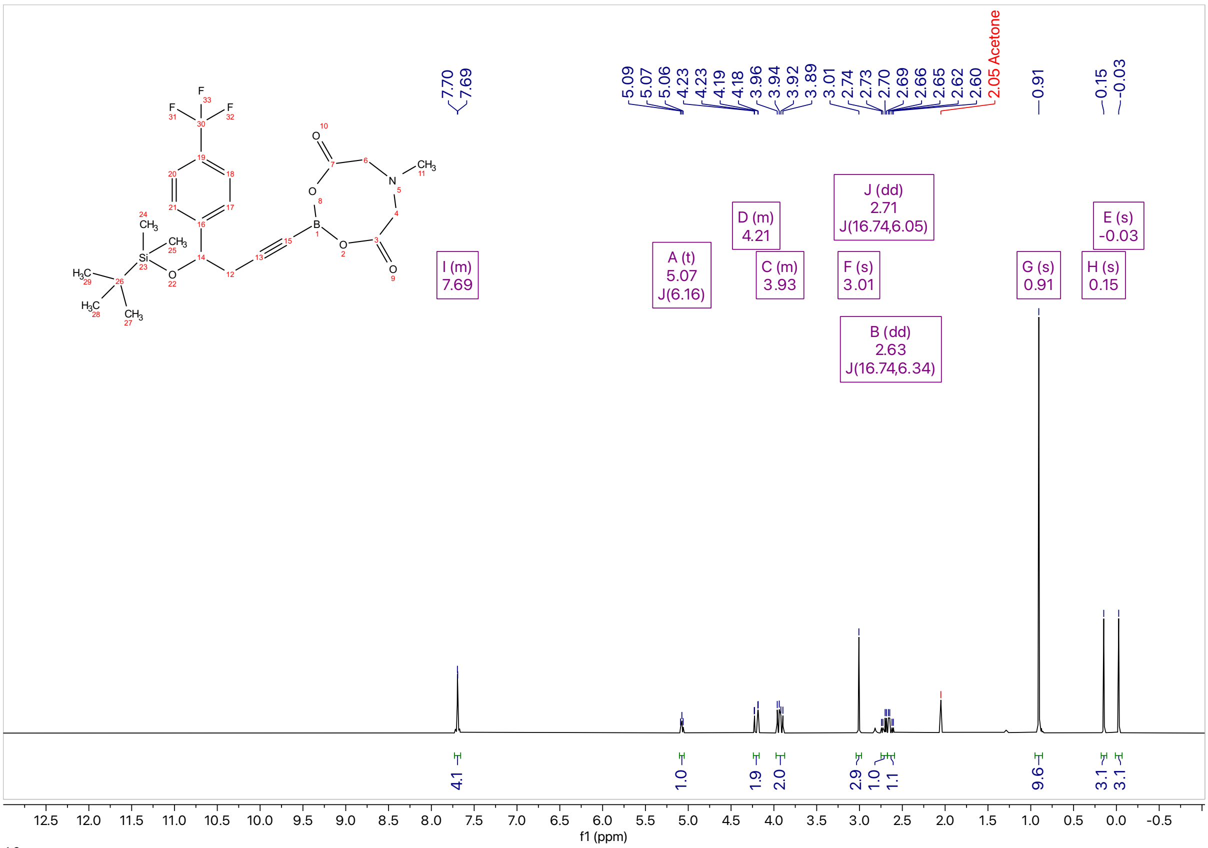

^{13}C DEPTQ

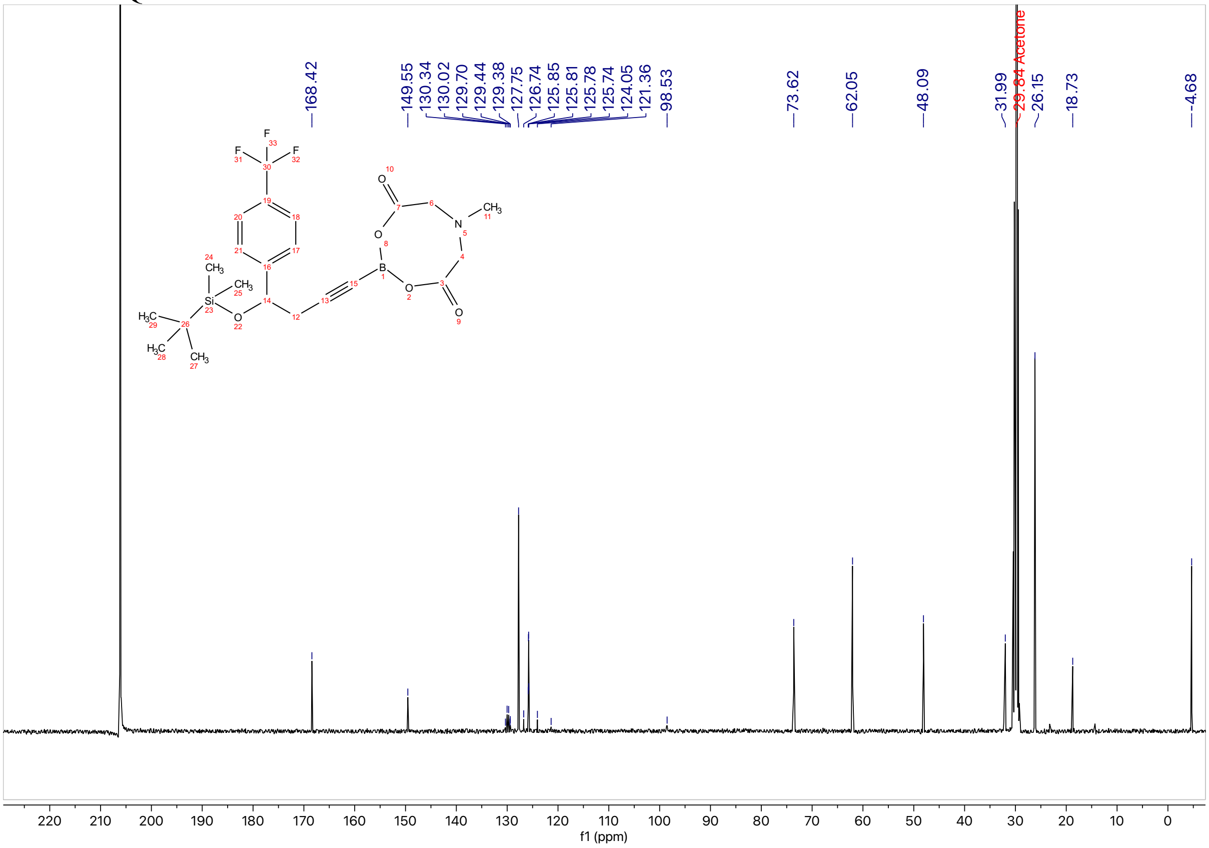

$^{19}F\{^1H\}$

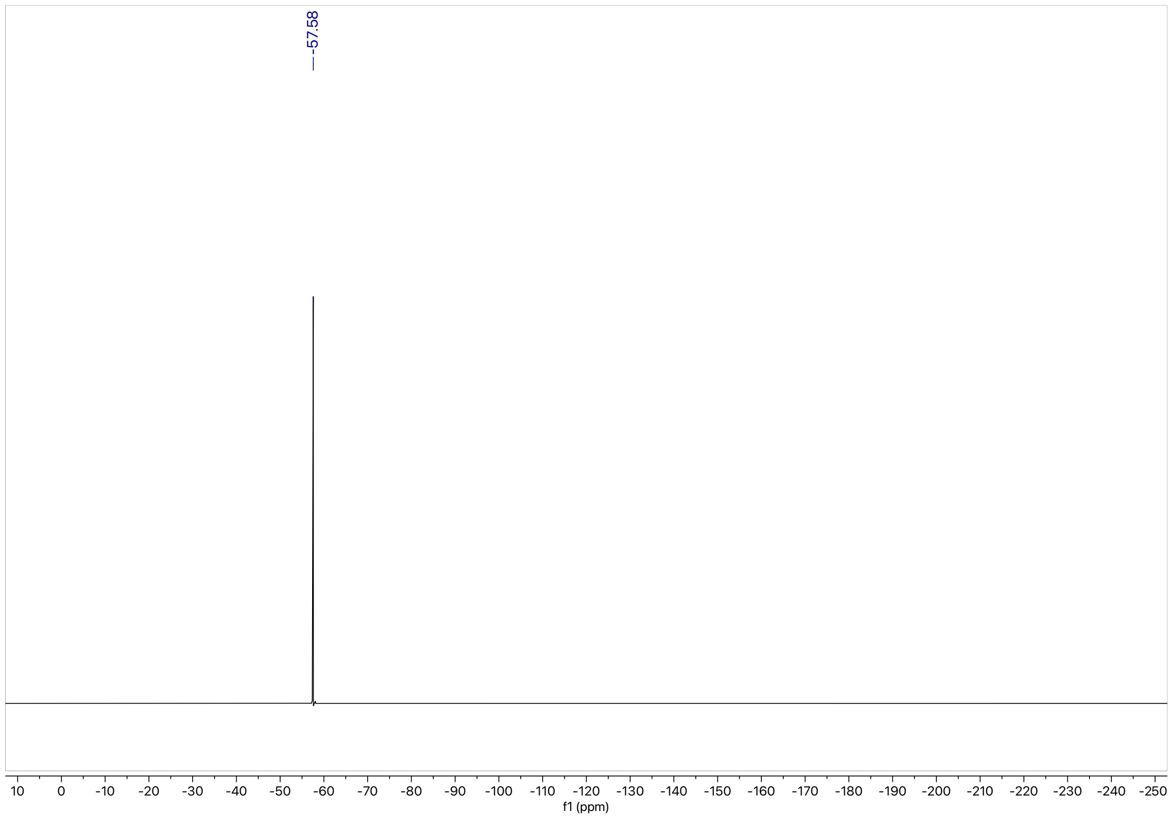
-57.58
10
0
-10
-20
-30
-40
-50
-60
-70
-80
-90
-100
-110
-120
-130
-140
-150
-160
-170
-180
-190
-200
-210
-220
-230
-240
-250
f1 (ppm)

(S45)

^{1}H

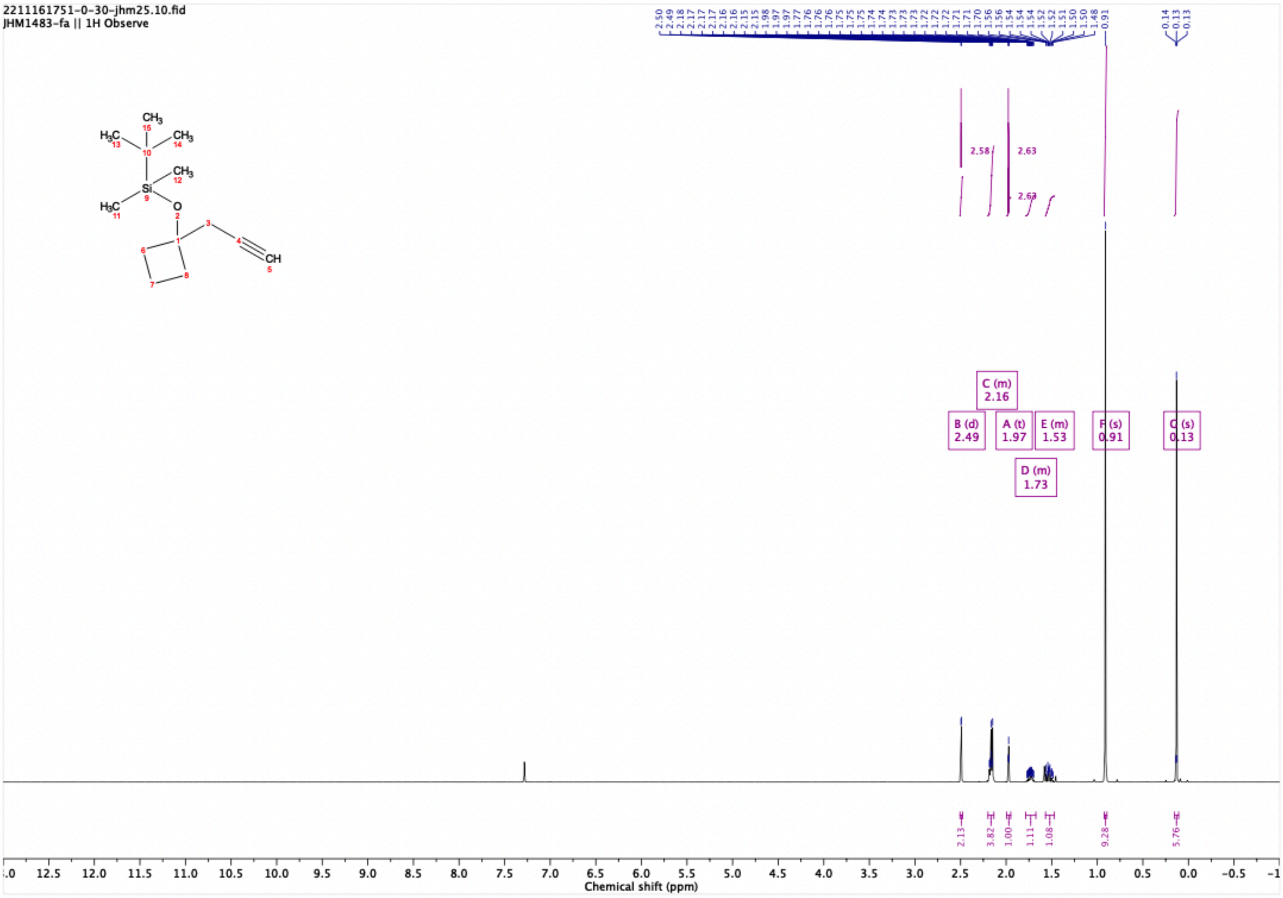

^{13}C DEPTQ

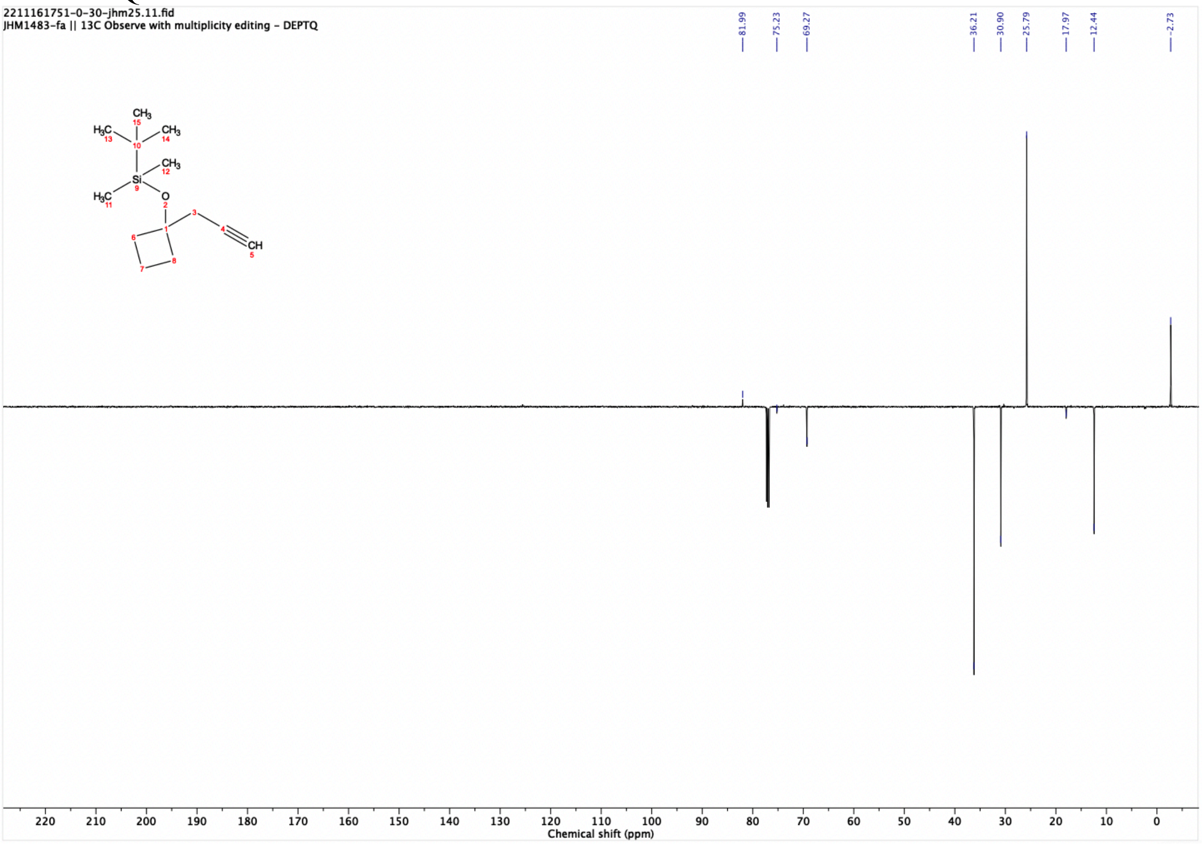

(S46)

^{1}H

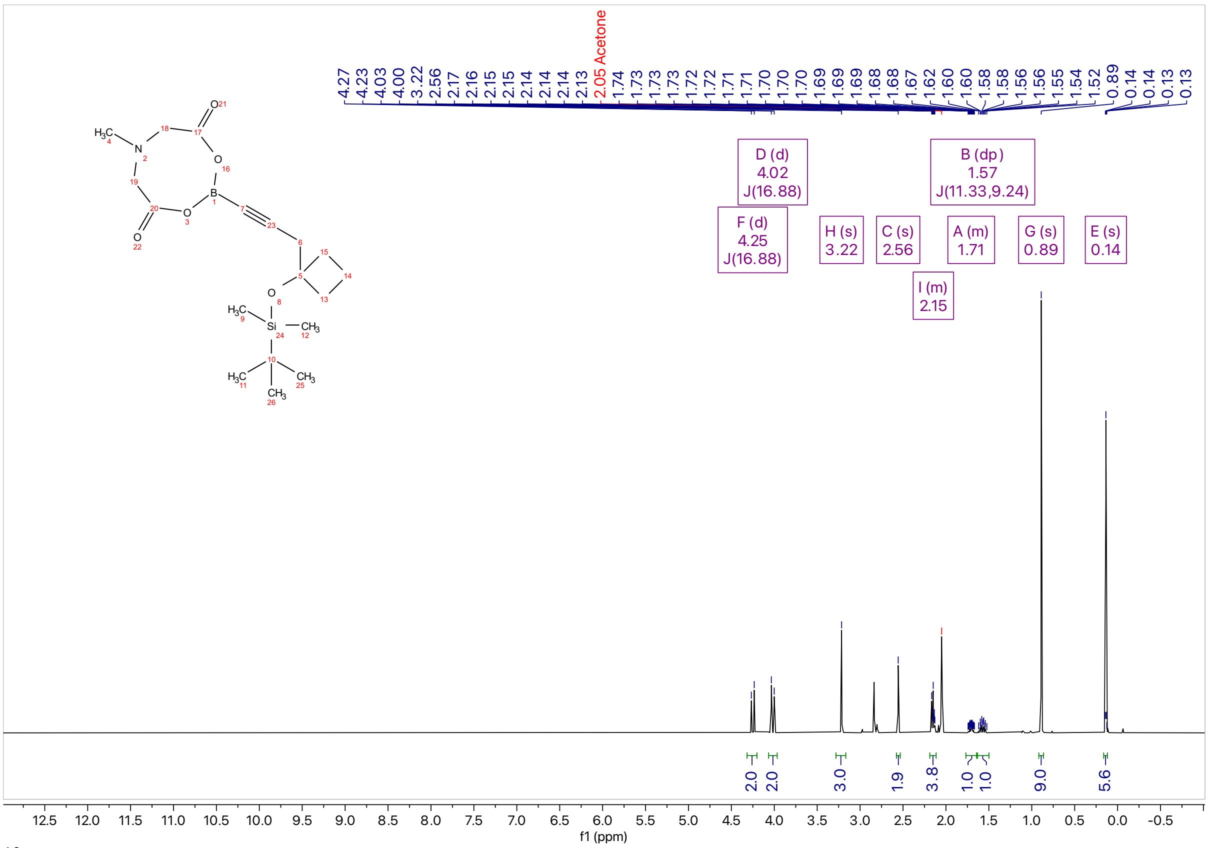
4.27 4.23 4.03 4.00 3.22 2.56 2.17 2.16 2.15 2.15 2.14 2.14 2.14 2.13 2.05 Acetone 1.74 1.73 1.73 1.73 1.72 1.72 1.71 1.71 1.70 1.70 1.70 1.69 1.69 1.69 1.68 1.68 1.67 1.62 1.60 1.60 1.58 1.58 1.56 1.56 1.55 1.54 1.52 0.89 0.14 0.14 0.13 0.13
D (d) 4.02 J(16.88)
B (dp) 1.57 J(11.33,9.24)
F (d) 4.25 J(16.88)
H (s) 3.22
C (s) 2.56
A (m) 1.71
G (s) 0.89
E (s) 0.14
I (m) 2.15
2.0 2.0 3.0 1.9 3.8 1.0 1.0 9.0 5.6
f1 (ppm)

^{13}C

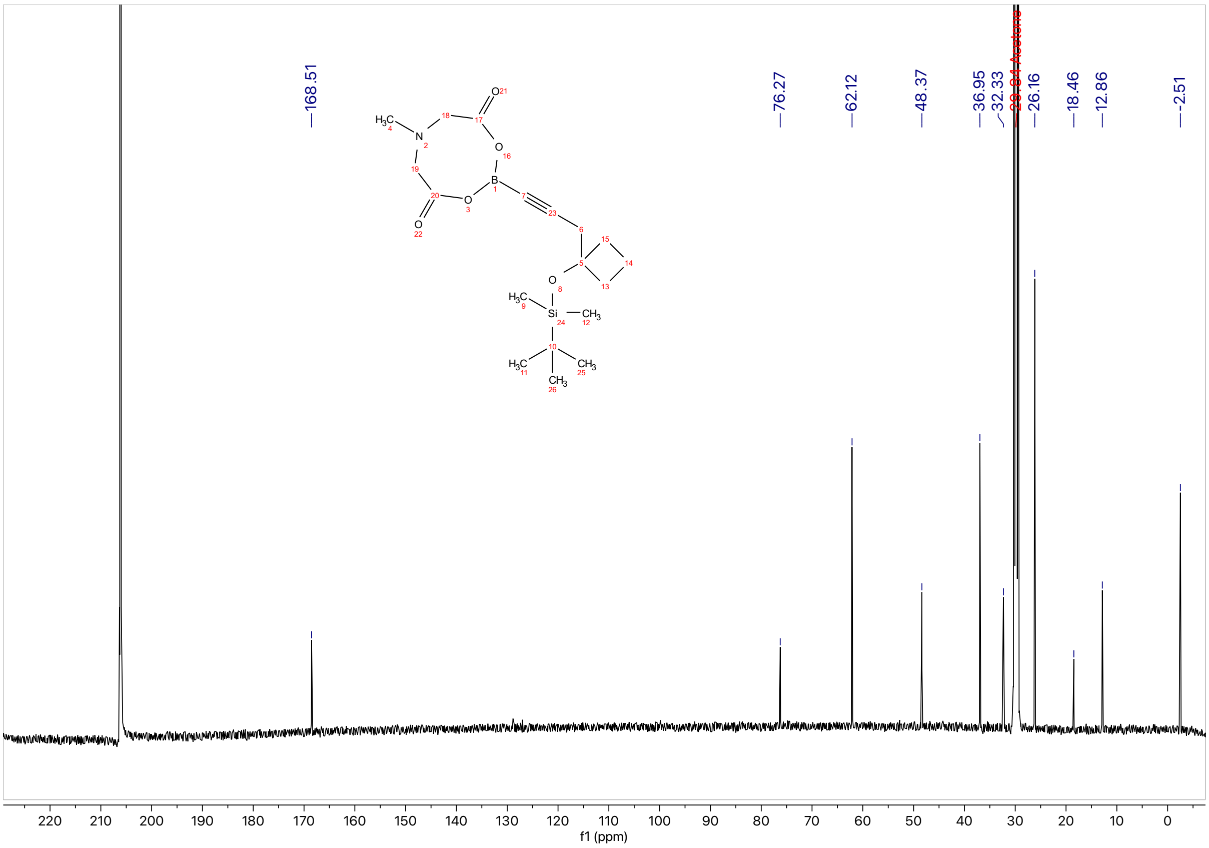
168.51
76.27
62.12
48.37
36.95
32.33
29.84 Acetone
26.16
18.46
12.86
-2.51
f1 (ppm)

(S47)

^{1}H

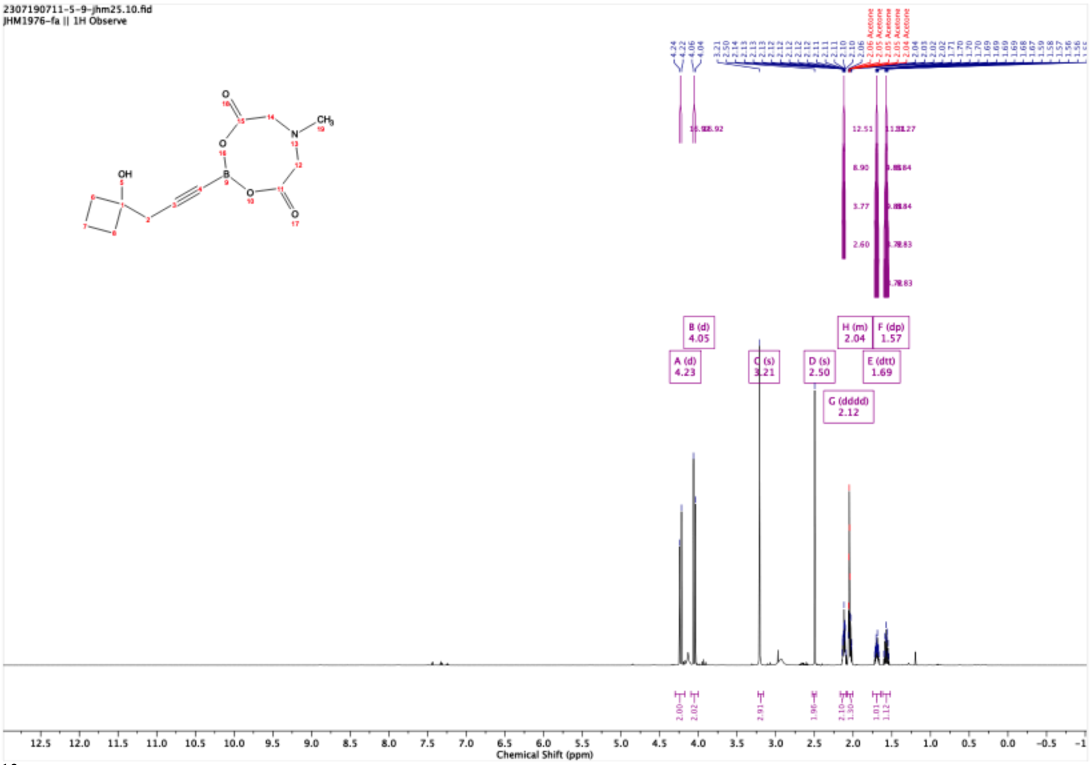

^{13}C DEPTQ

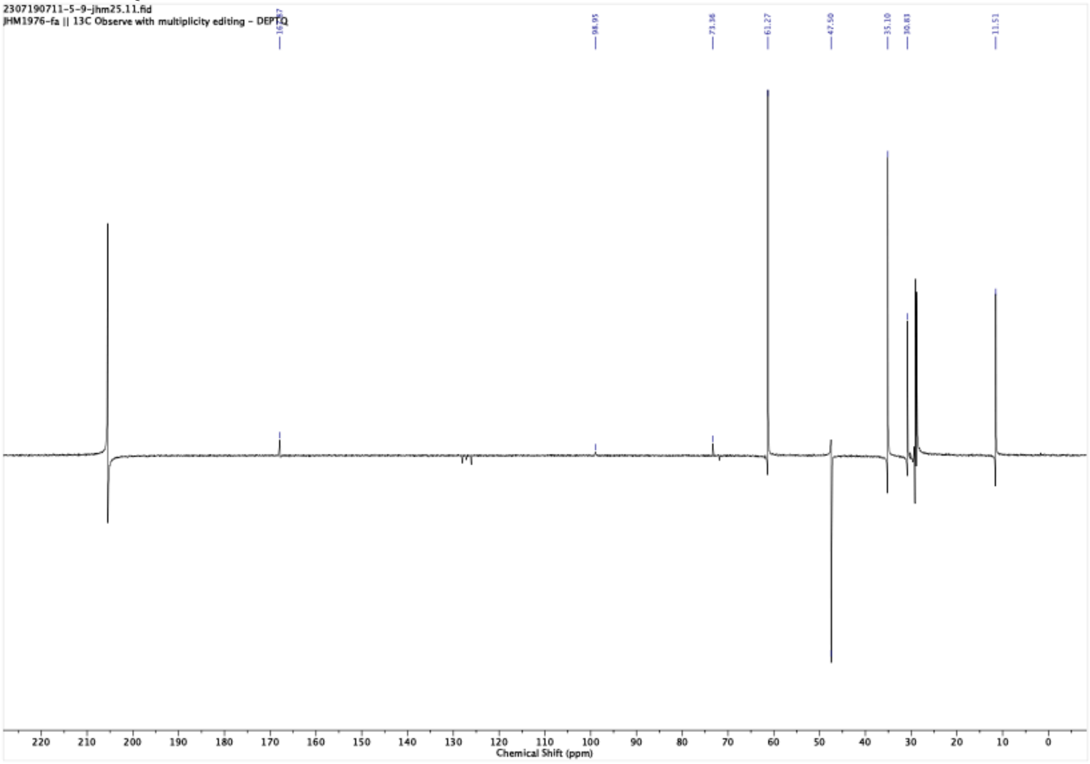

(S49)

^{1}H

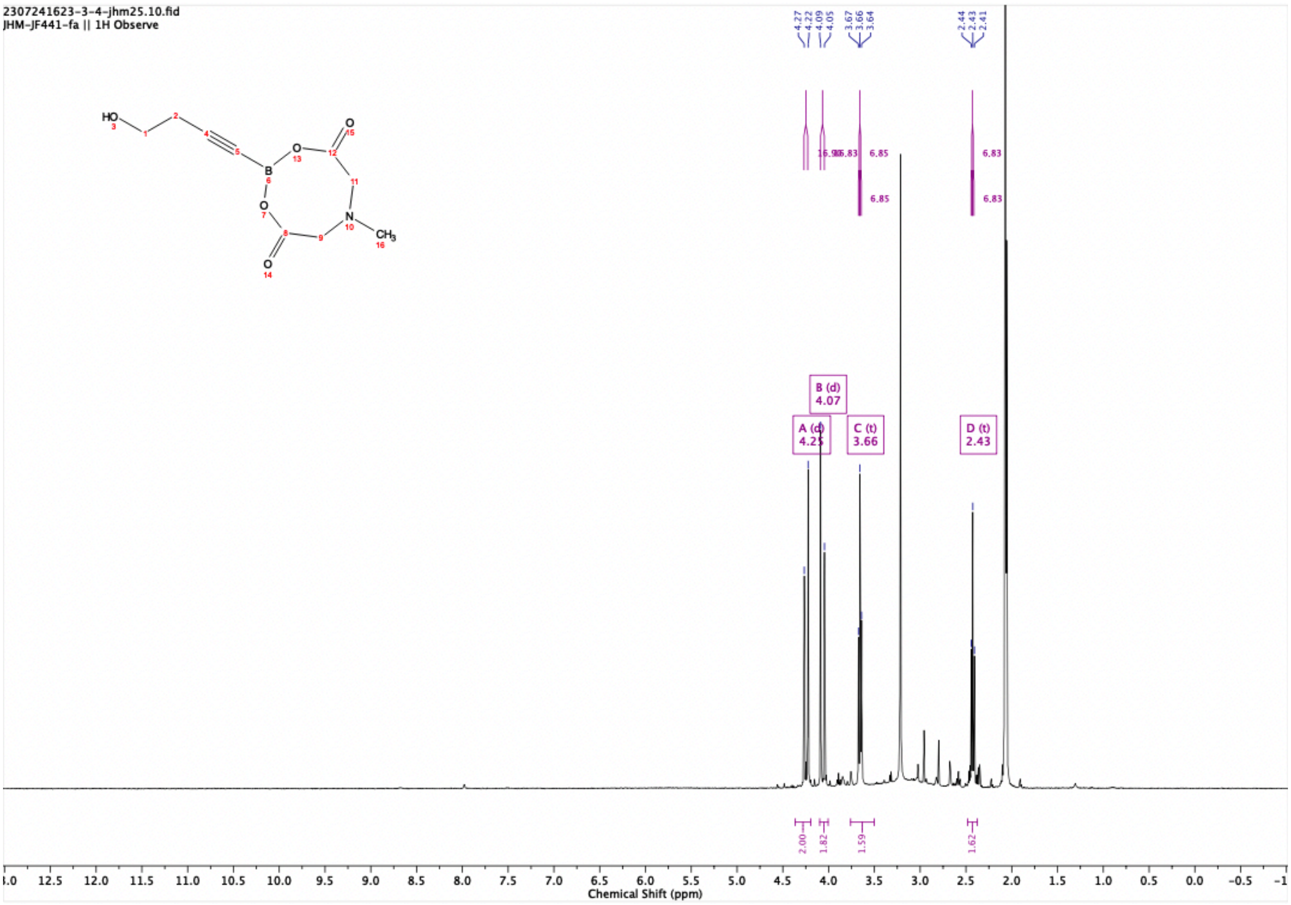

^{13}C DEPTQ

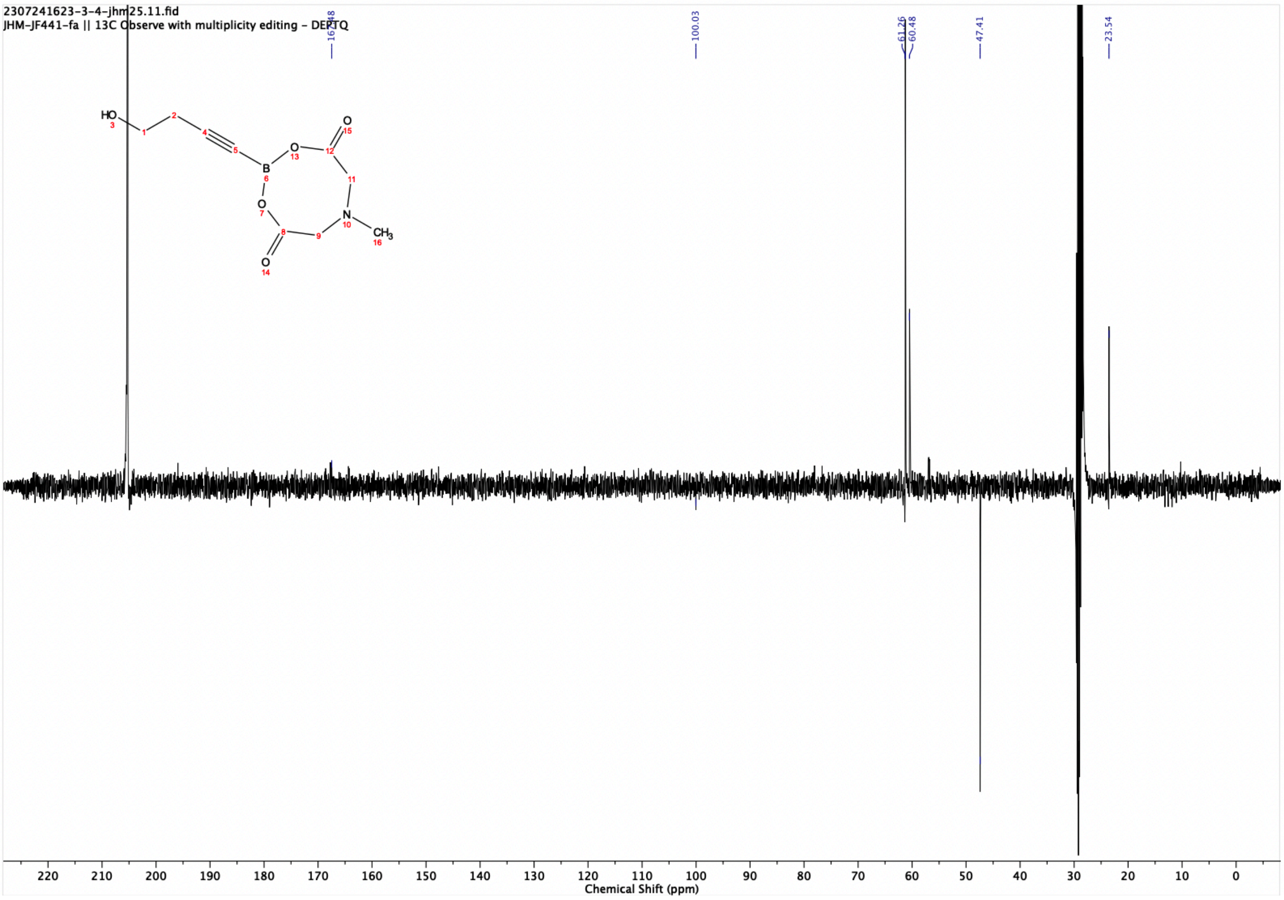

(S50)

^{1}H

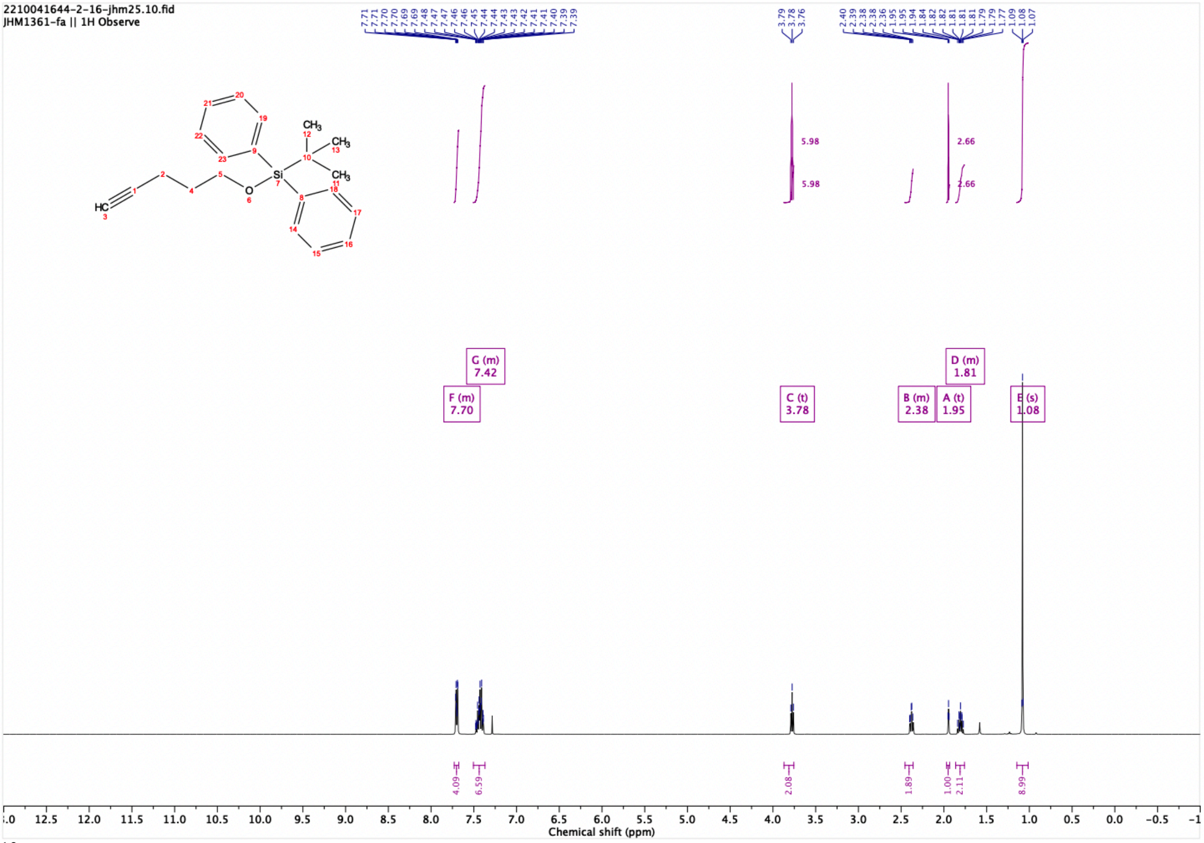

^{13}C DEPTQ

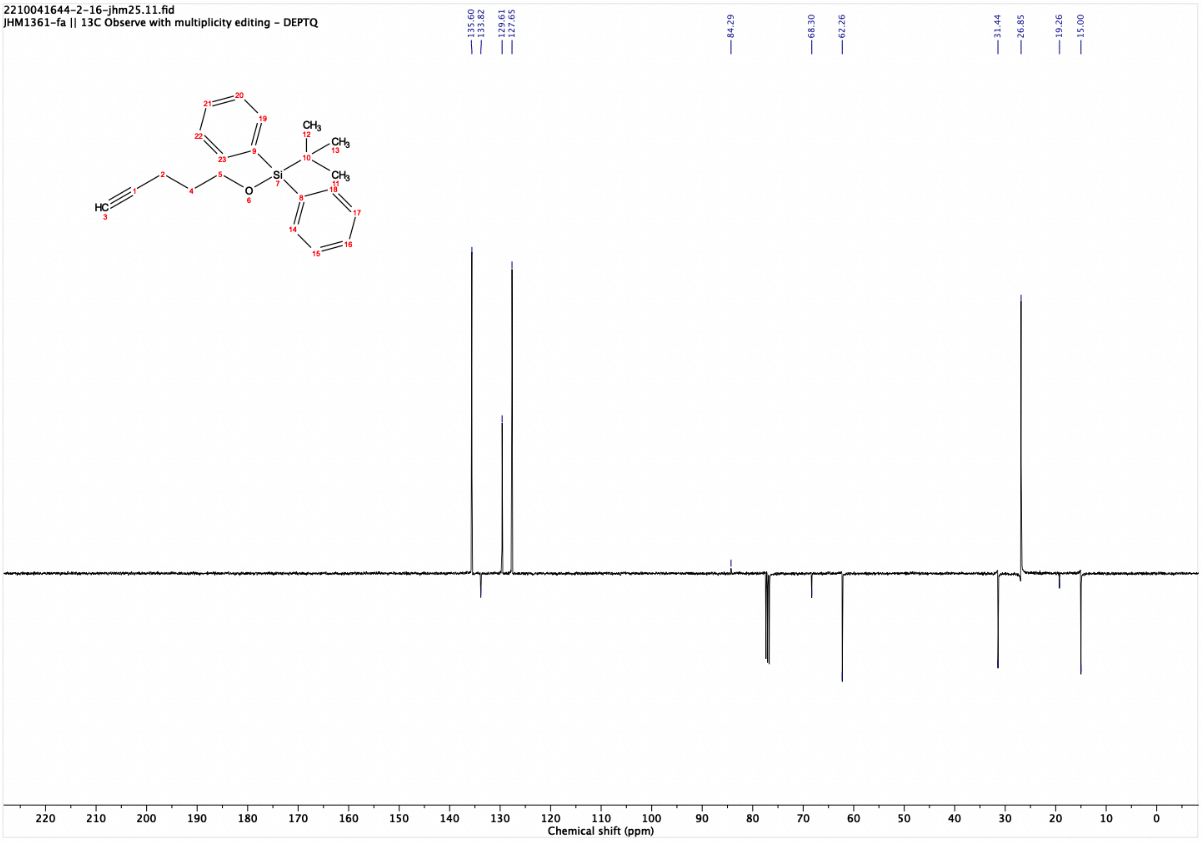

(S51)

^{1}H

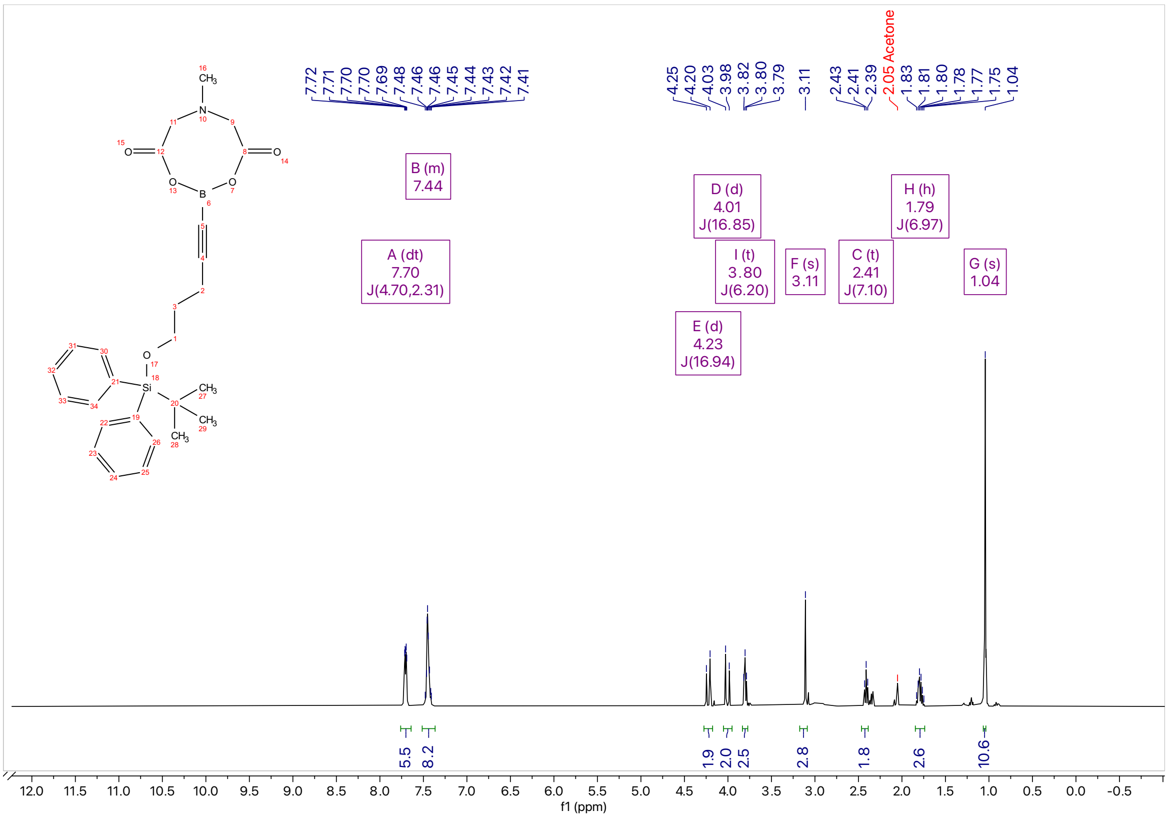

^{13}C

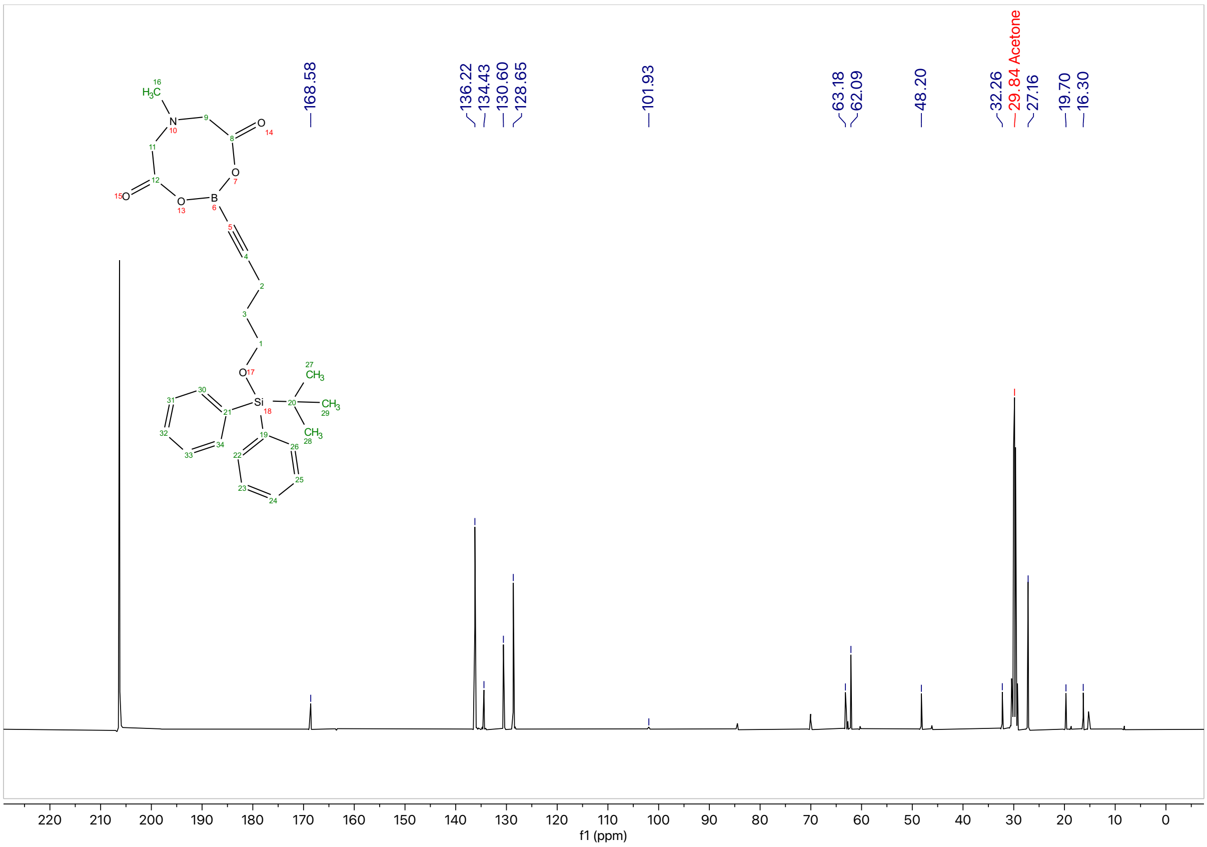

(S52)

^{1}H

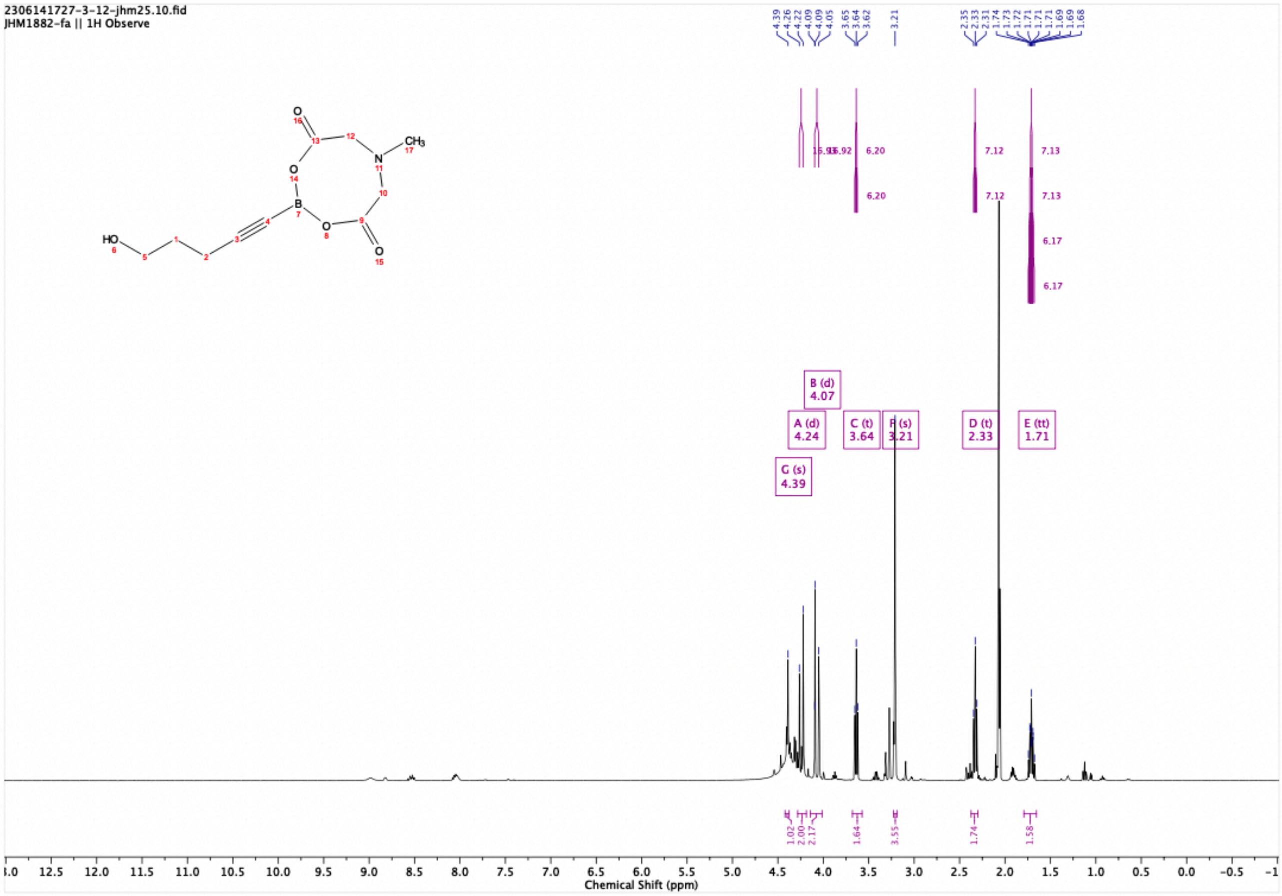

^{13}C DEPTQ

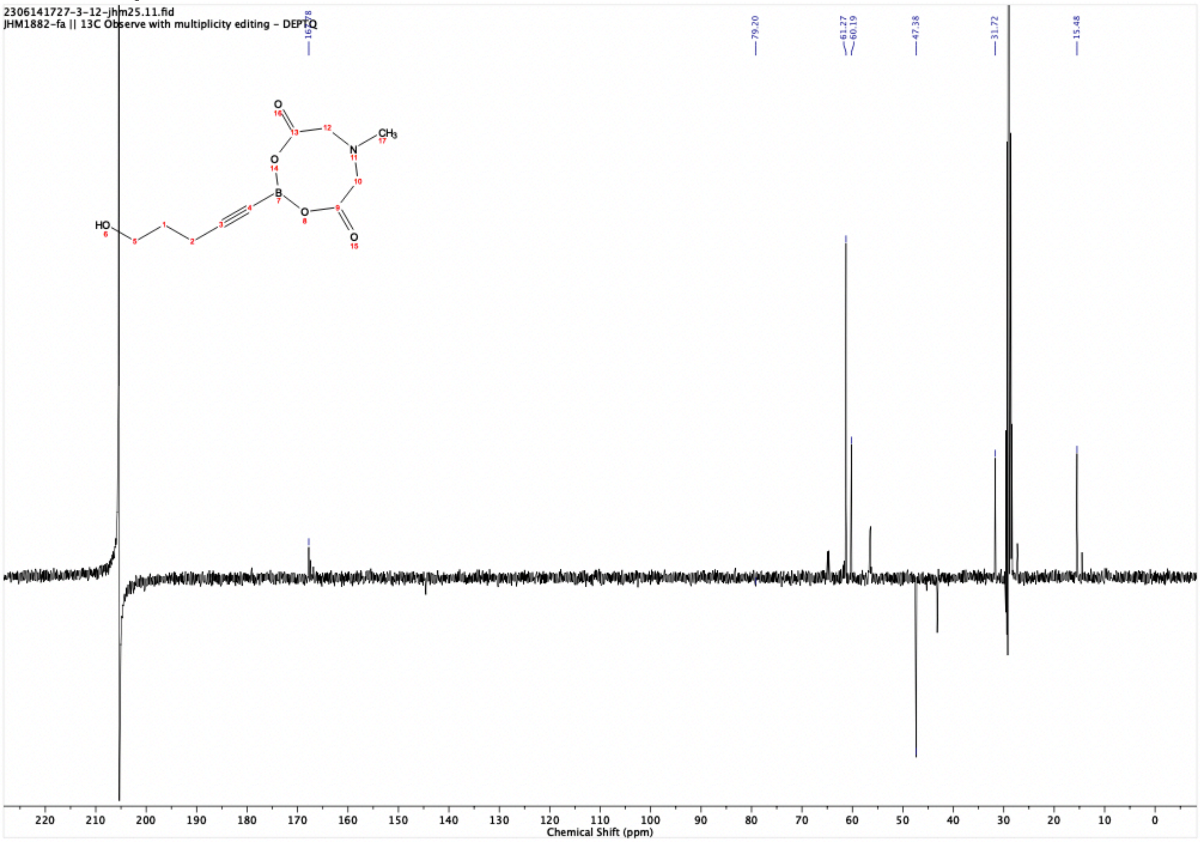

(S54)

^{1}H

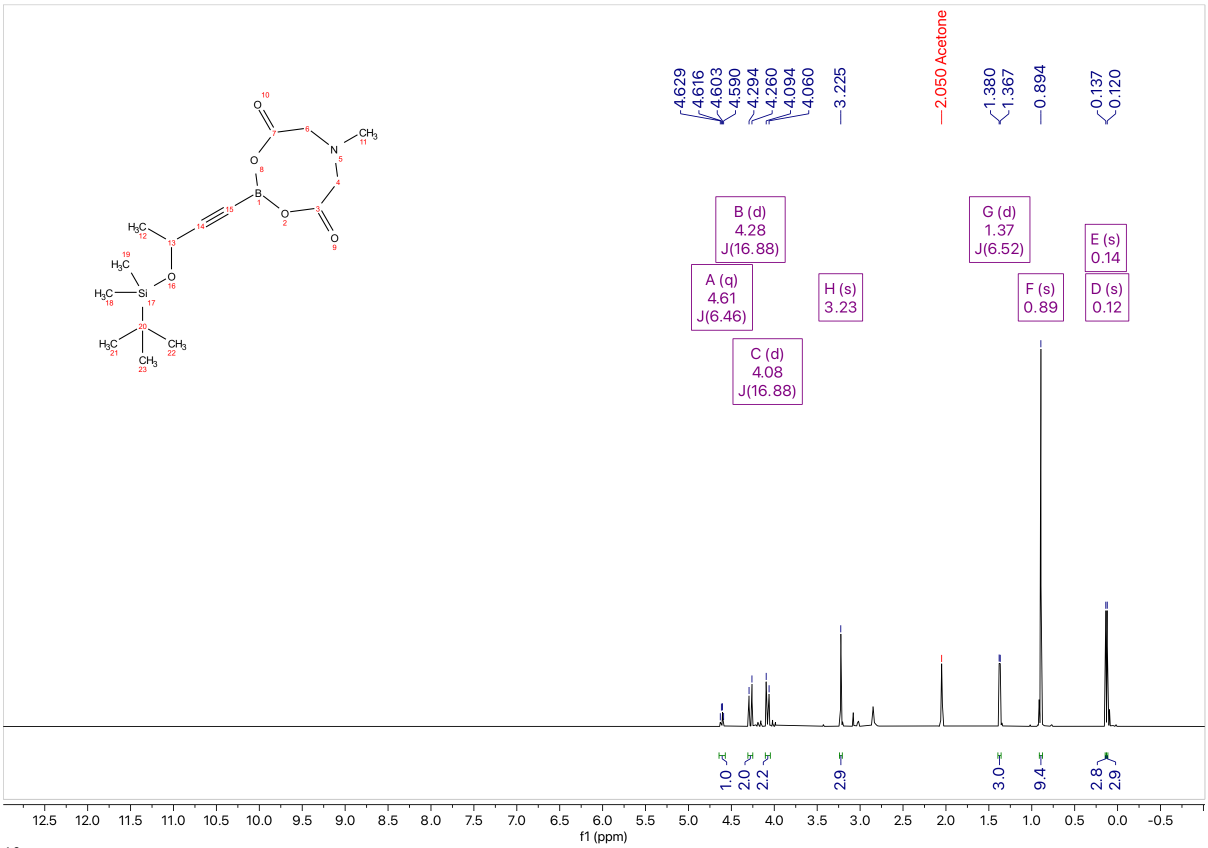
4.629
4.616
4.603
4.590
4.294
4.260
4.094
4.060
3.225
2.050 Acetone
1.380
1.367
0.894
0.137
0.120
B (d)
4.28
J(16.88)
G (d)
1.37
J(6.52)
E (s)
0.14
A (q)
4.61
J(6.46)
H (s)
3.23
F (s)
0.89
D (s)
0.12
C (d)
4.08
J(16.88)
1.0
2.0
2.2
2.9
3.0
9.4
2.8
2.9
f1 (ppm)

^{13}C

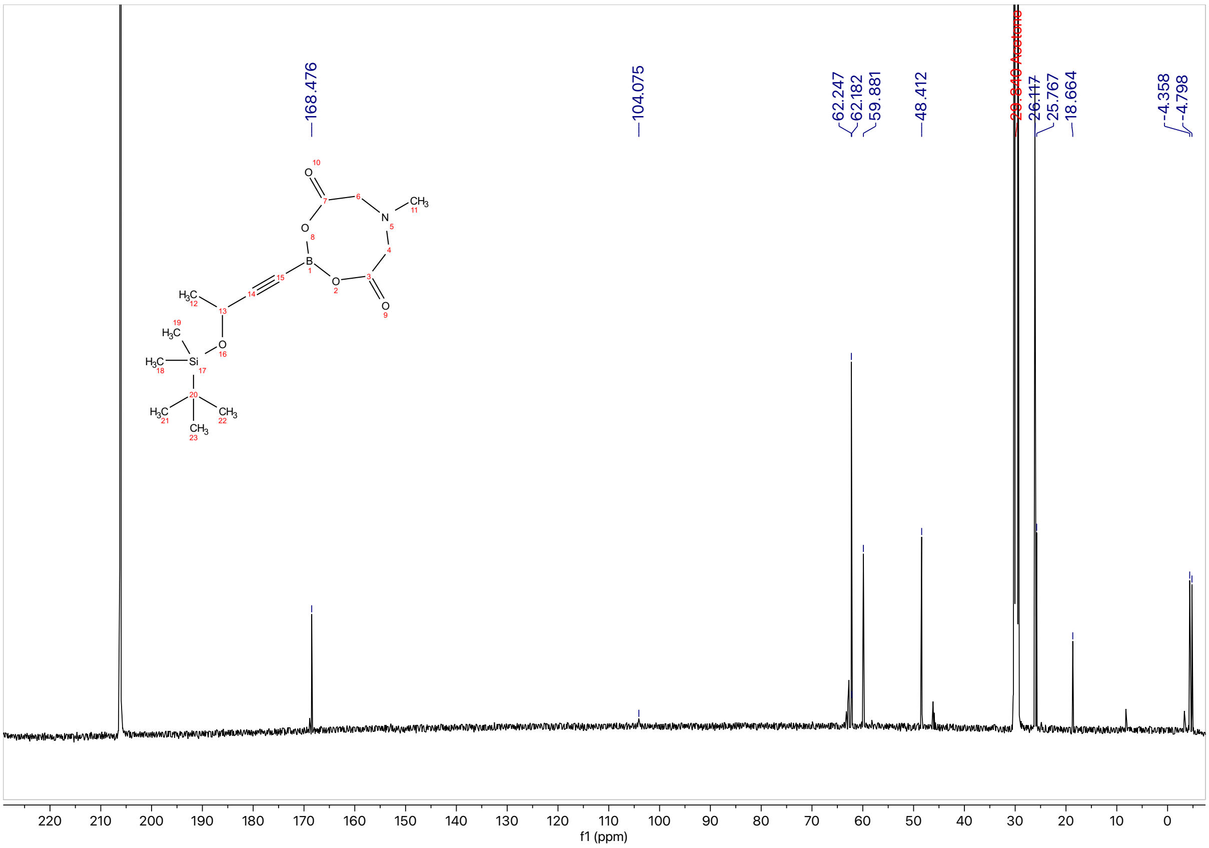
168.476
104.075
62.247
62.182
59.881
48.412
26.117
25.767
18.664
-4.358
-4.798
f1 (ppm)

(S55)

^{1}H

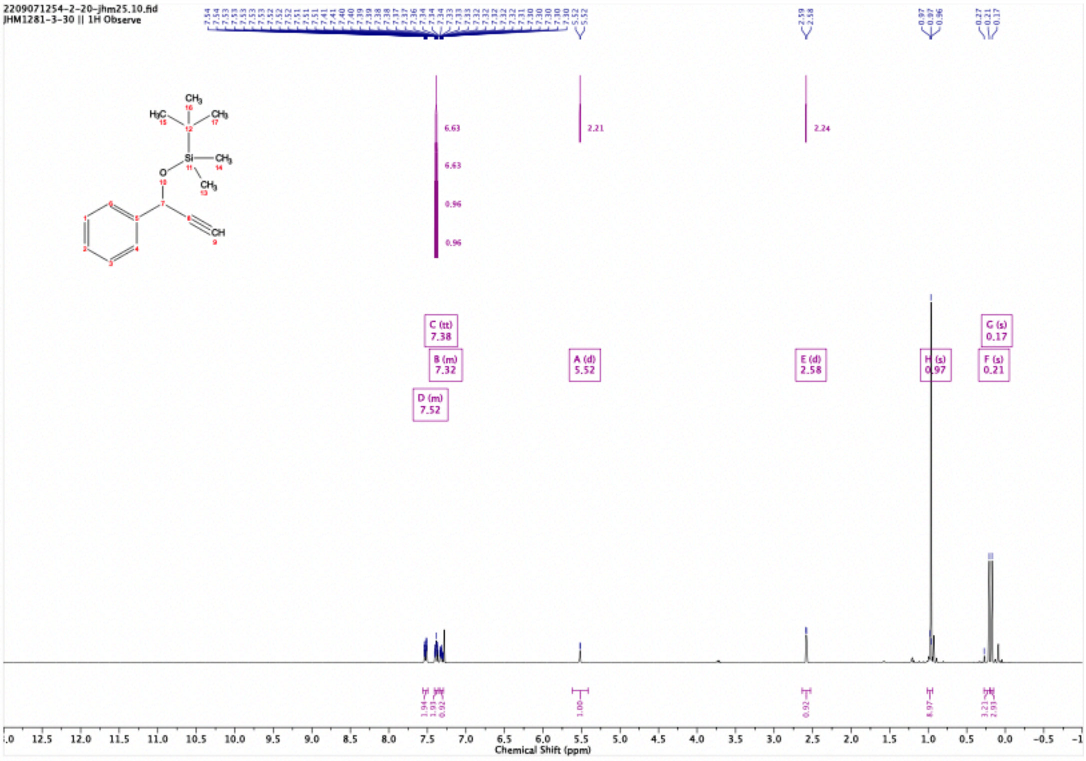

^{13}C DEPTQ

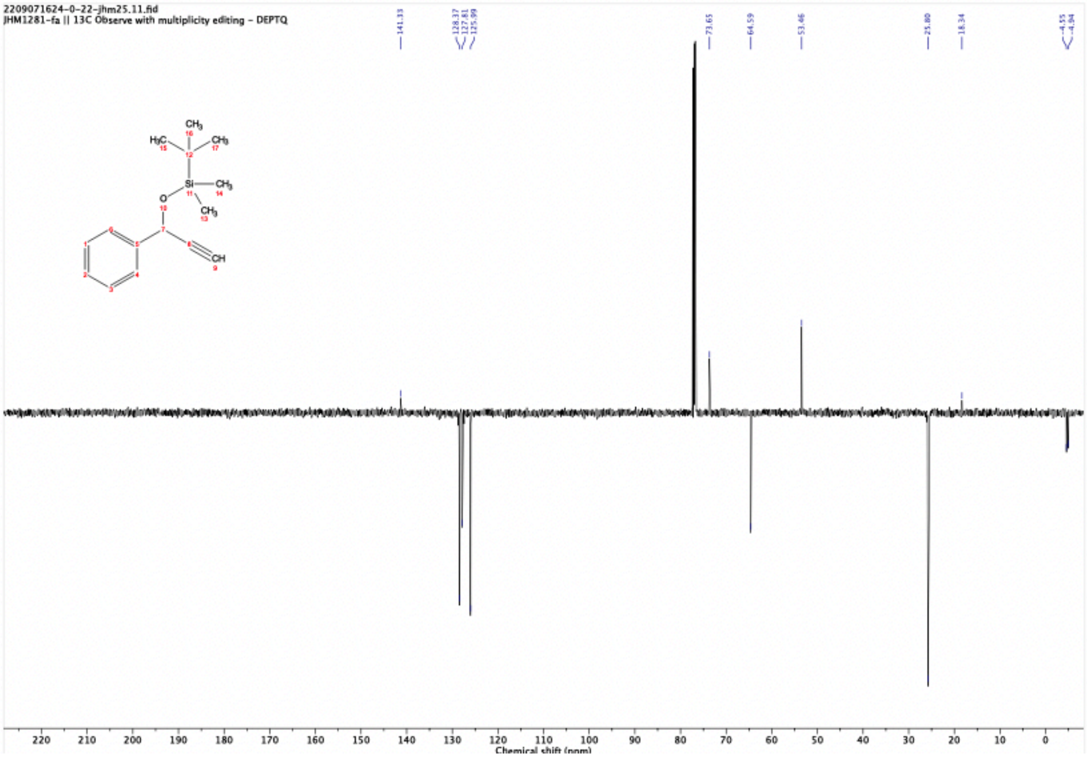

(S56)

^{1}H

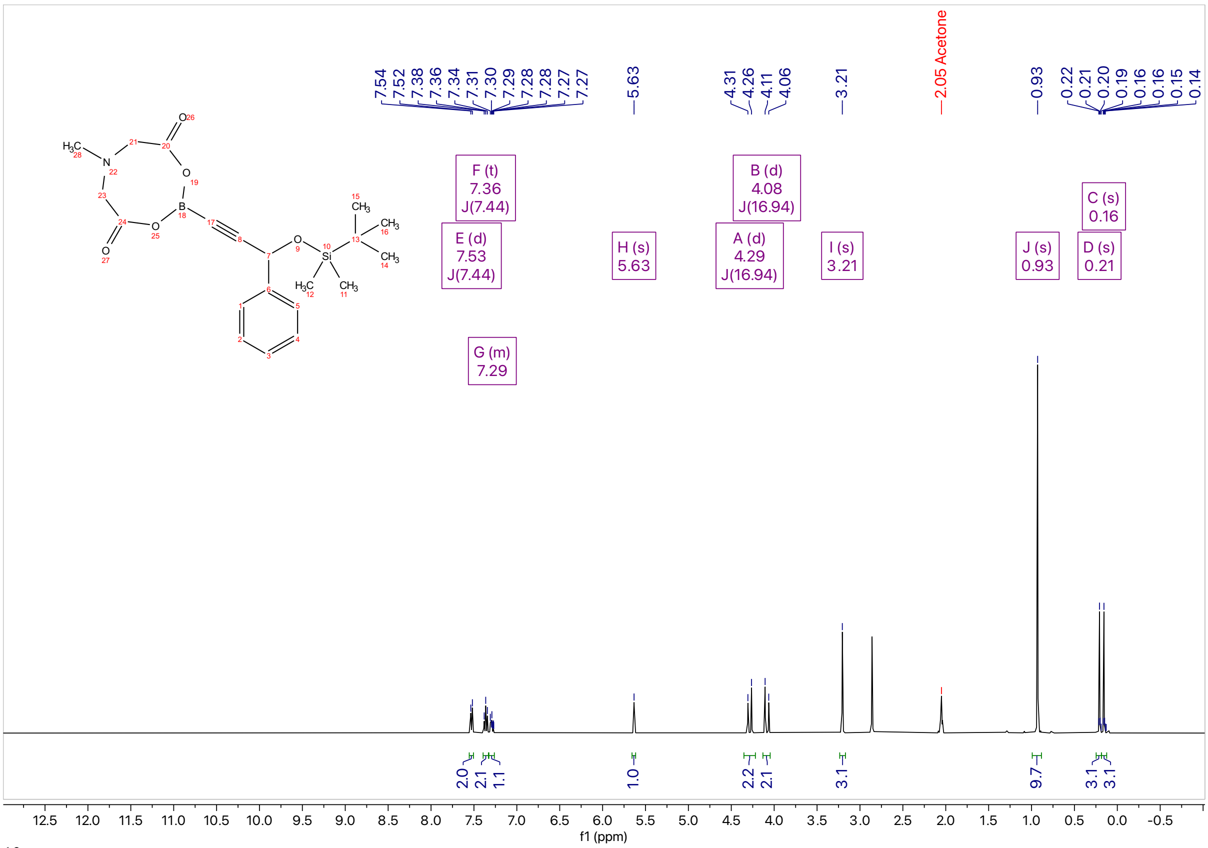

7.54
7.52
7.38
7.36
7.34
7.31
7.30
7.29
7.28
7.28
7.27
7.27
5.63
4.31
4.26
4.11
4.06
3.21
2.05 Acetone
0.93
0.22
0.21
0.20
0.19
0.16
0.16
0.15
0.14
F (t) 7.36 J(7.44)
B (d) 4.08 J(16.94)
C (s) 0.16
E (d) 7.53 J(7.44)
H (s) 5.63
A (d) 4.29 J(16.94)
I (s) 3.21
J (s) 0.93
D (s) 0.21
G (m) 7.29
2.0
2.1
1.1
1.0
2.2
2.1
3.1
9.7
3.1
3.1
f1 (ppm)

^{13}C

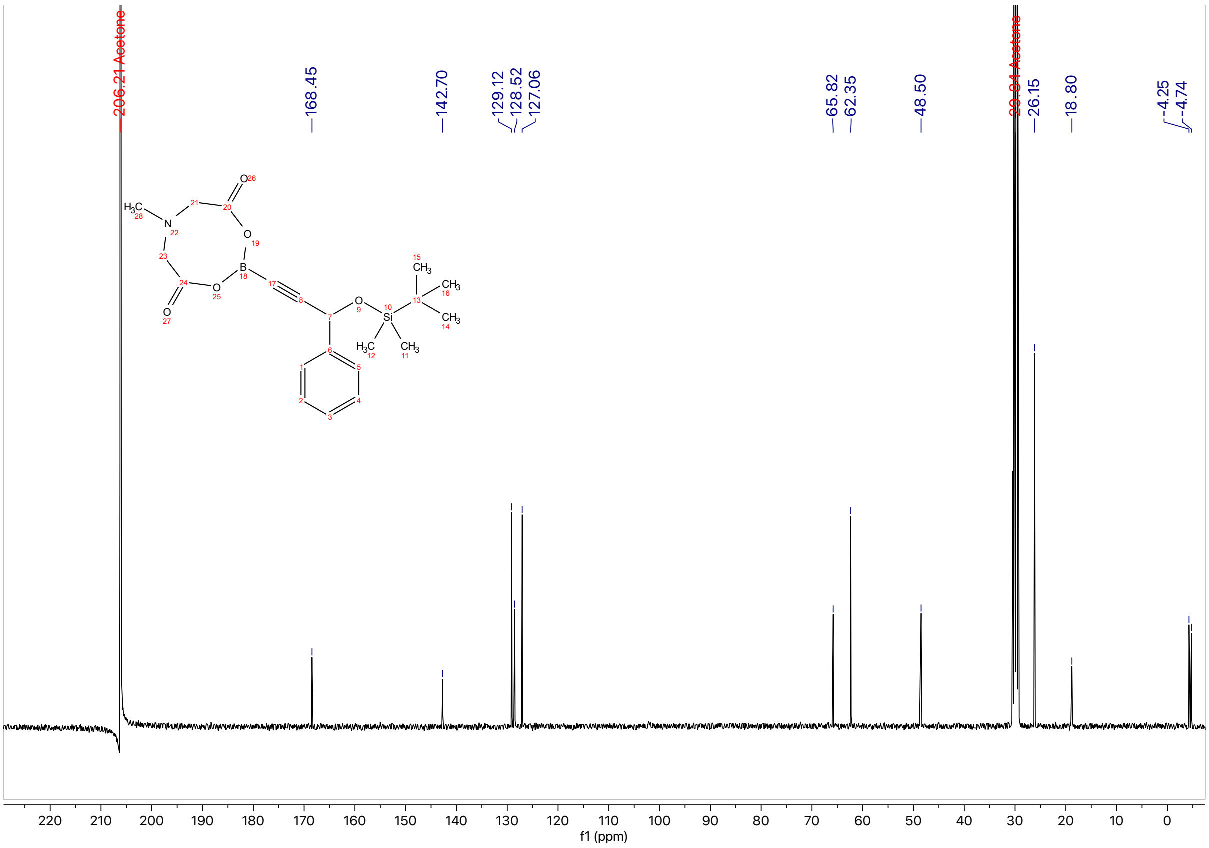

206.21 Acetone
168.45
142.70
129.12
128.52
127.06
65.82
62.35
48.50
29.84 Acetone
26.15
18.80
-4.25
-4.74
f1 (ppm)

(S57)

^{1}H

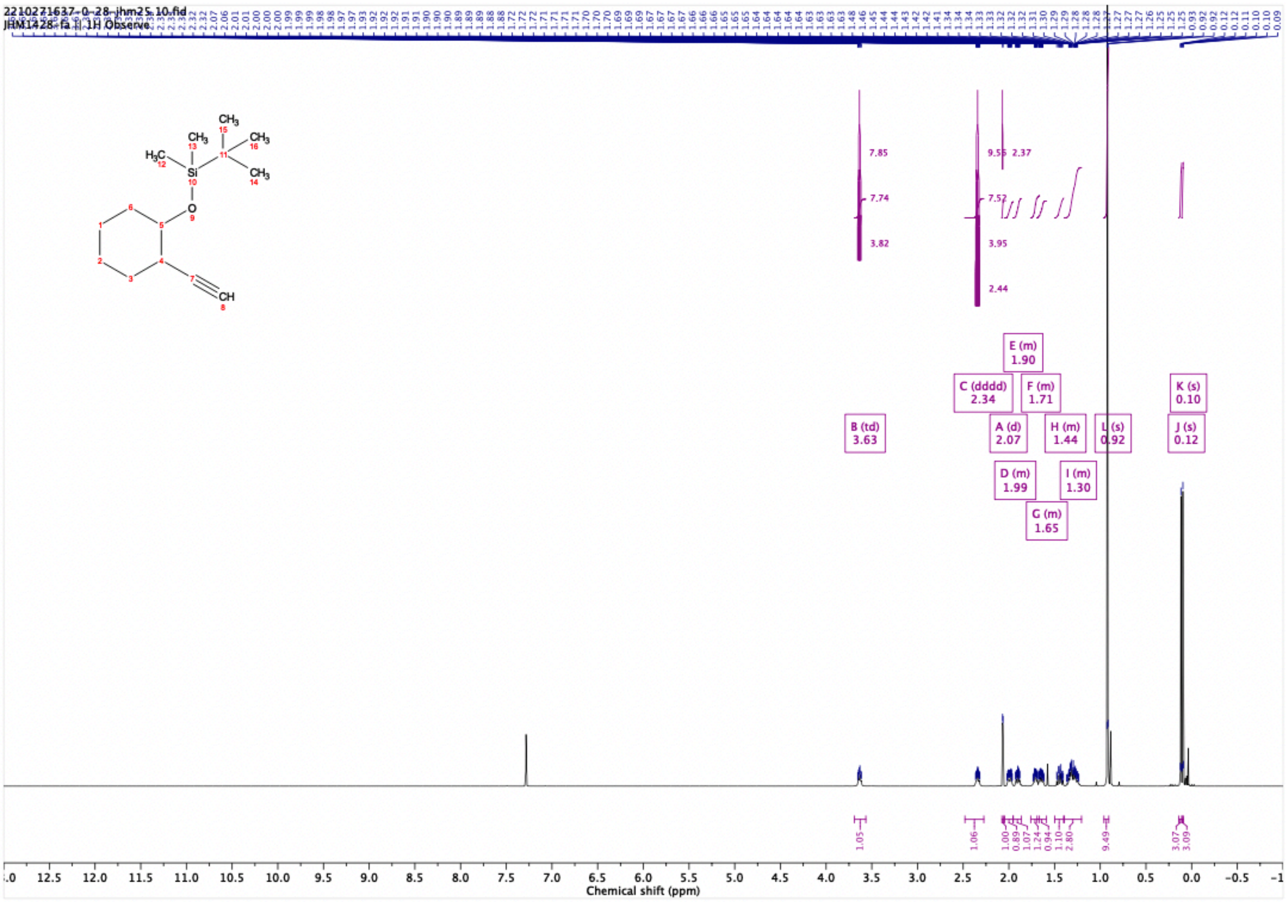

^{13}C DEPTQ

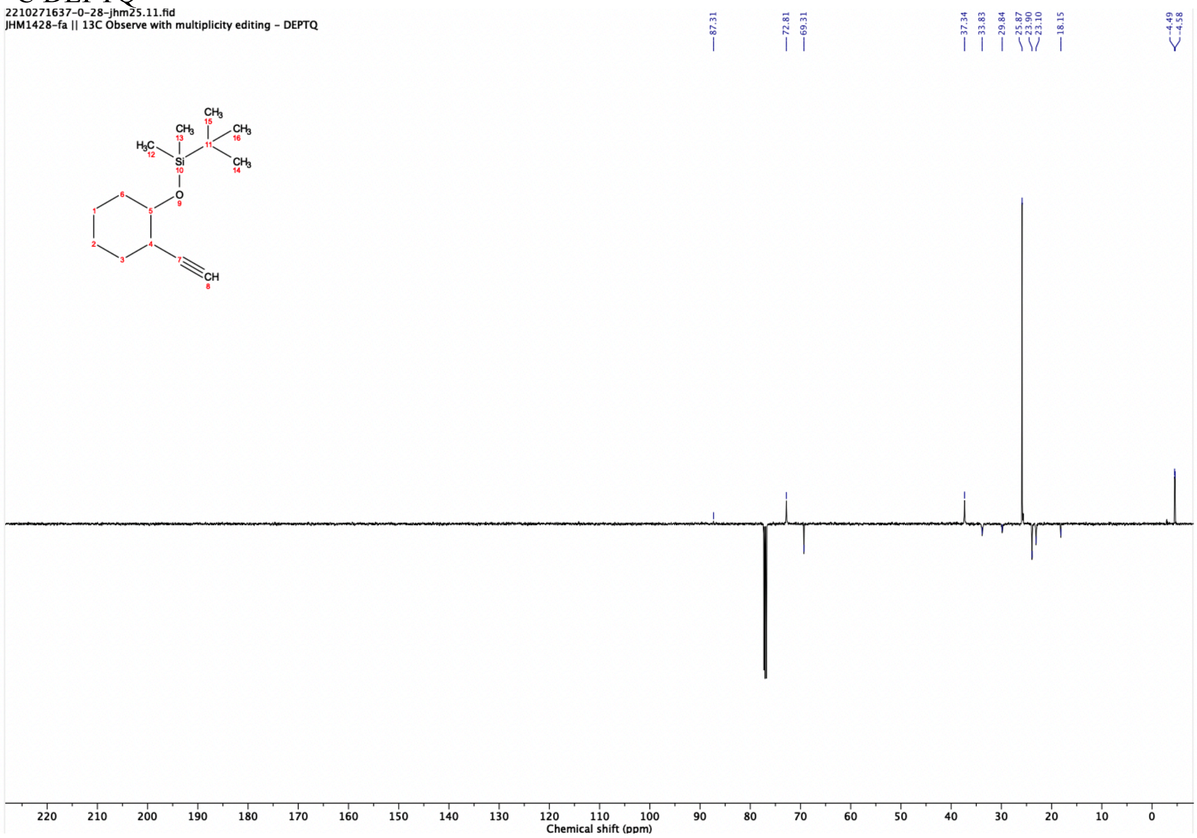

(S58)

^{1}H

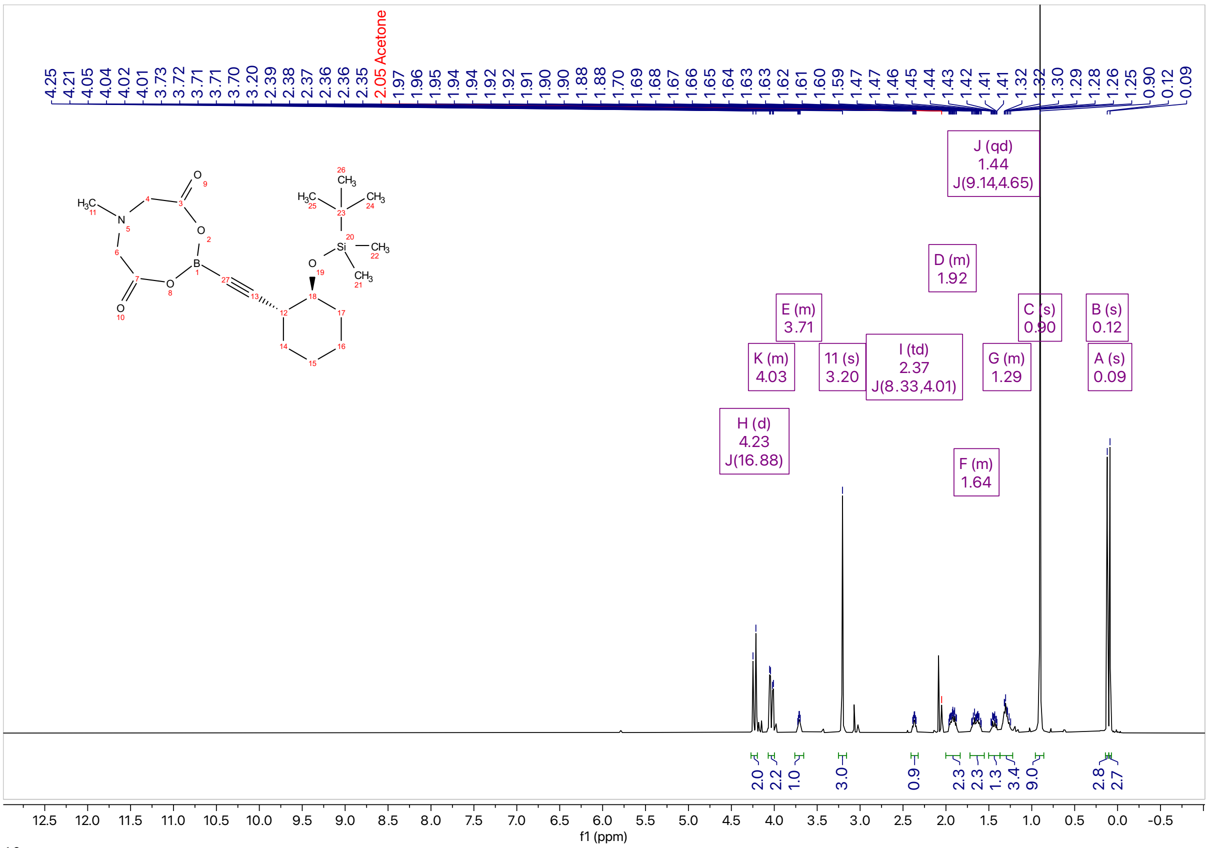
4.25 4.21 4.05 4.04 4.02 4.01 3.73 3.72 3.71 3.71 3.70 3.20 2.39 2.38 2.37 2.36 2.36 2.35 2.05 Acetone 1.97 1.96 1.95 1.94 1.94 1.92 1.92 1.91 1.90 1.90 1.88 1.88 1.70 1.69 1.68 1.67 1.66 1.65 1.64 1.63 1.63 1.62 1.61 1.60 1.59 1.47 1.46 1.45 1.44 1.43 1.42 1.41 1.41 1.32 1.32 1.30 1.29 1.28 1.26 1.25 0.90 0.12 0.09
J (qd) 1.44 J(9.14,4.65)
D (m) 1.92
E (m) 3.71
C (s) 0.90
B (s) 0.12
K (m) 4.03
11 (s) 3.20
I (td) 2.37 J(8.33,4.01)
G (m) 1.29
A (s) 0.09
H (d) 4.23 J(16.88)
F (m) 1.64
2.0 2.2 1.0 3.0 0.9 2.3 2.3 1.3 3.4 9.0 2.8 2.7
12.5 12.0 11.5 11.0 10.5 10.0 9.5 9.0 8.5 8.0 7.5 7.0 6.5 6.0 5.5 5.0 4.5 4.0 3.5 3.0 2.5 2.0 1.5 1.0 0.5 0.0 -0.5
f1 (ppm)

^{13}C

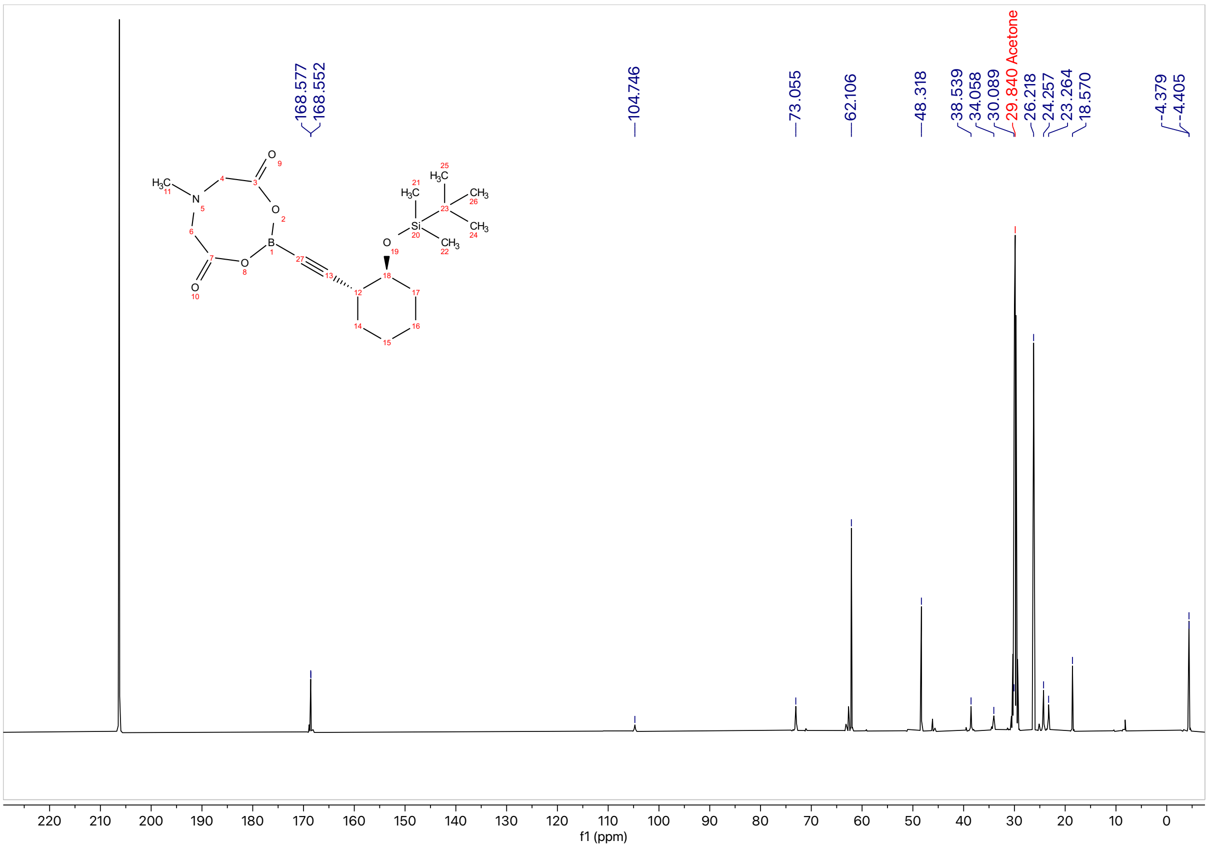
168.577 168.552
104.746
73.055
62.106
48.318
38.539 34.058 30.089 29.840 Acetone 26.218 24.257 23.264 18.570
-4.379 -4.405
220 210 200 190 180 170 160 150 140 130 120 110 100 90 80 70 60 50 40 30 20 10 0
f1 (ppm)

(S59)

^{1}H

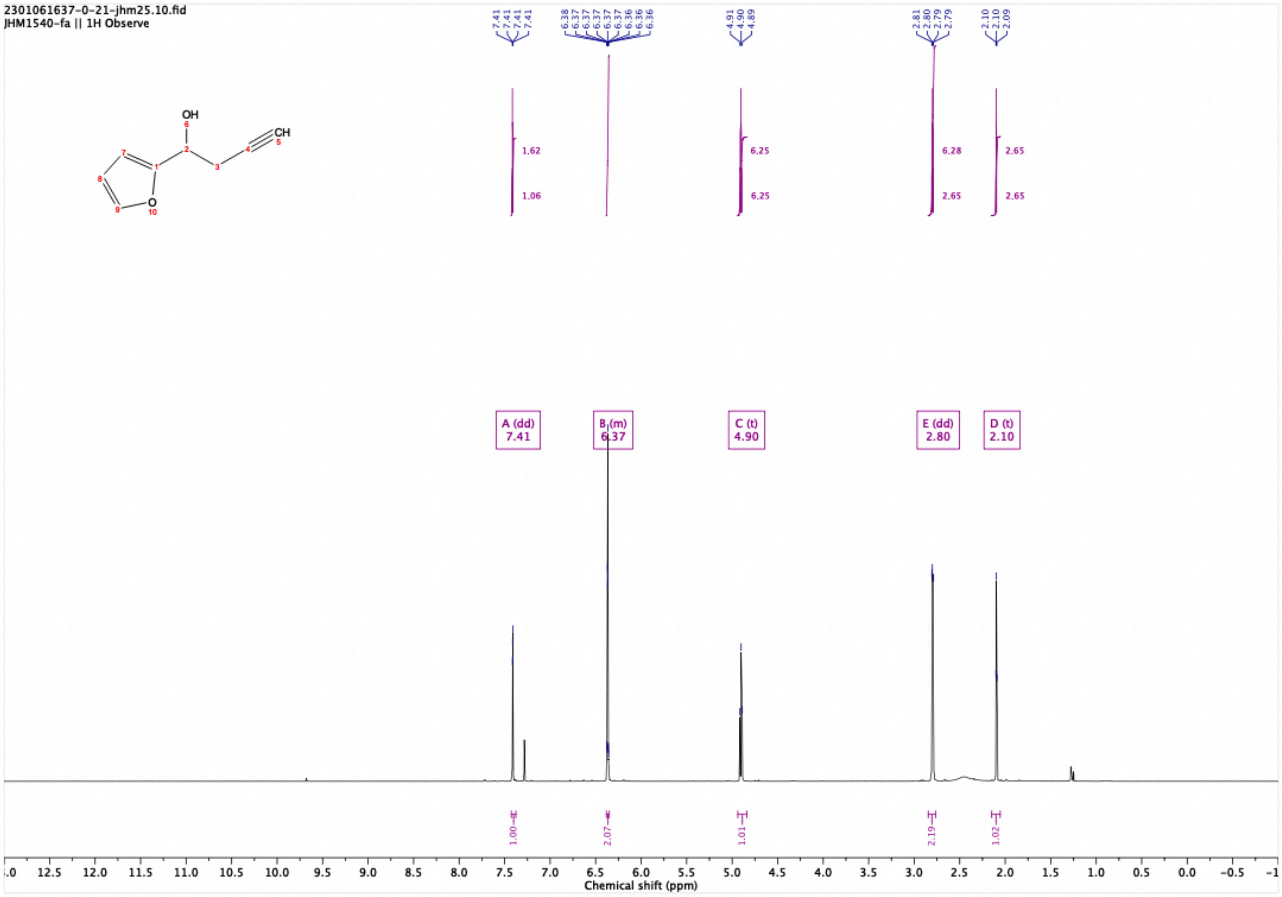

^{13}C DEPTQ

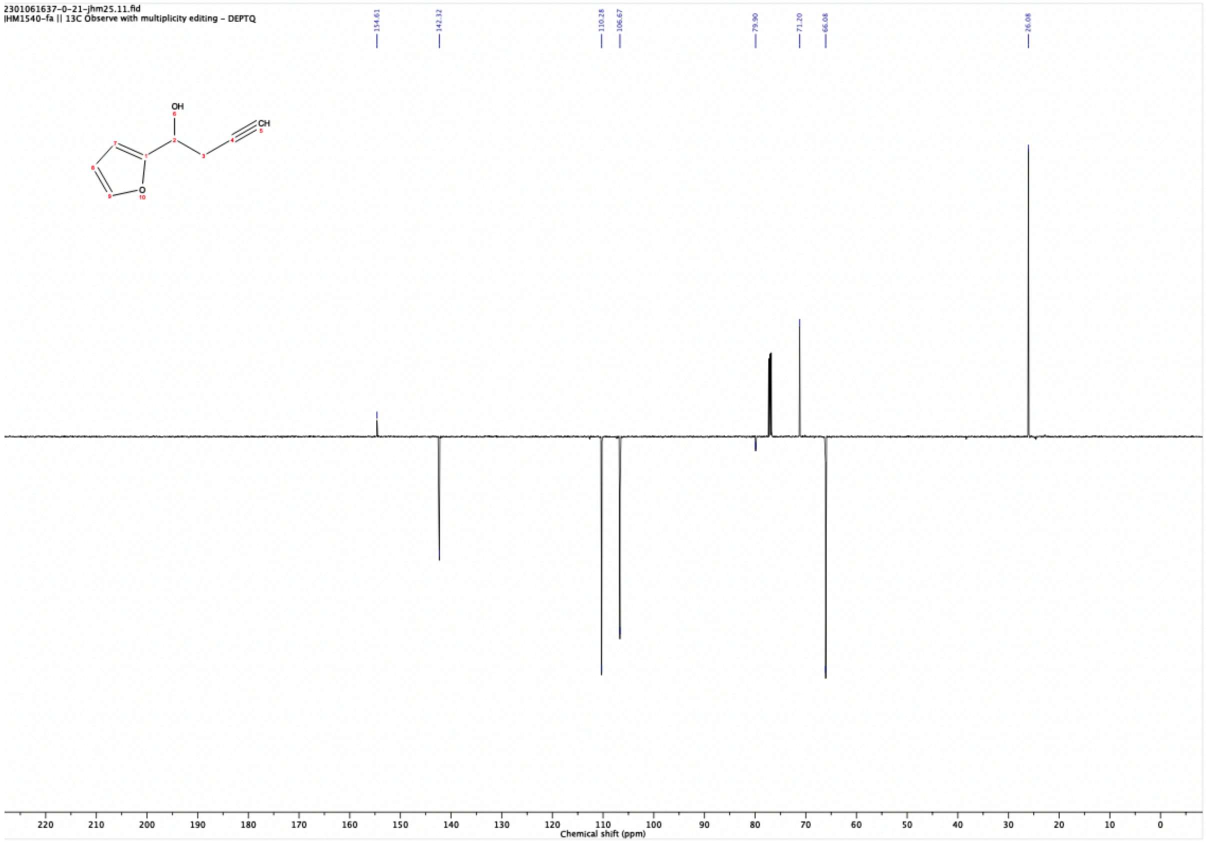

(S60)

[1]H

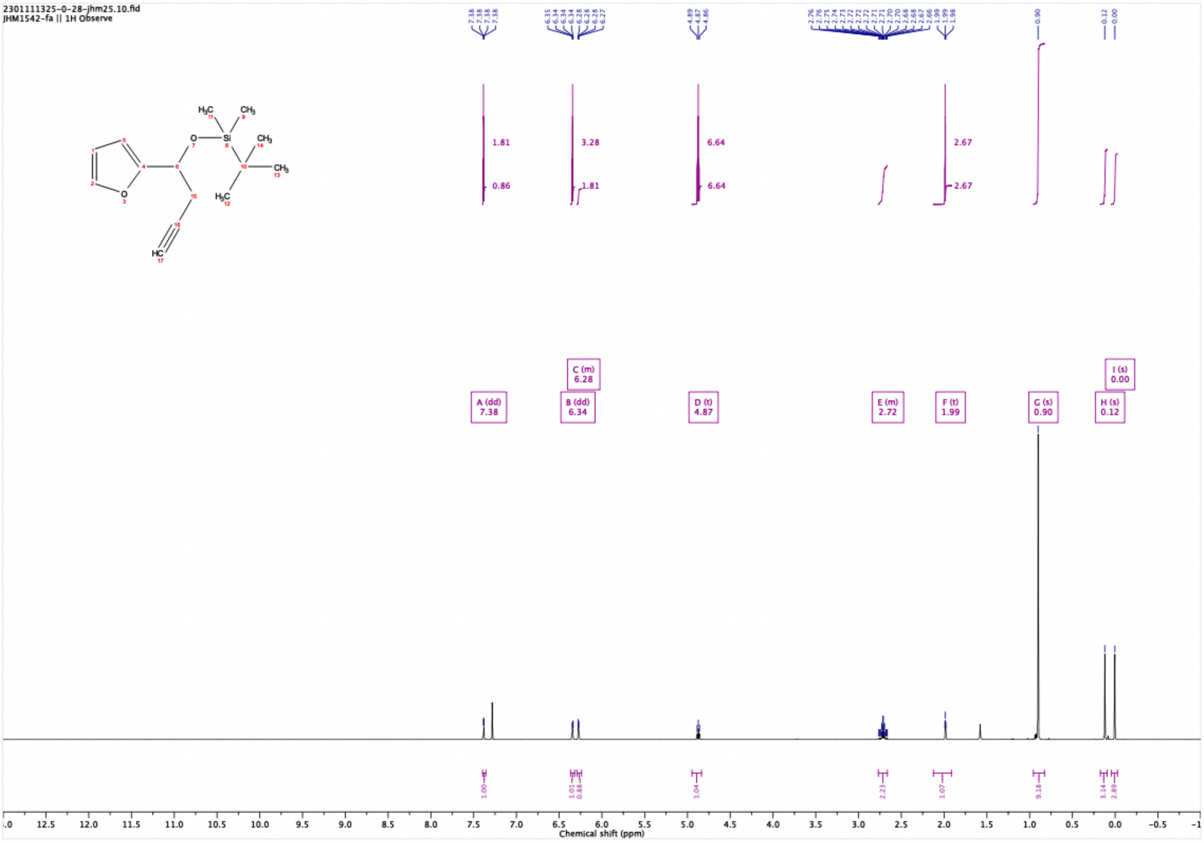

^{13}C DEPTQ

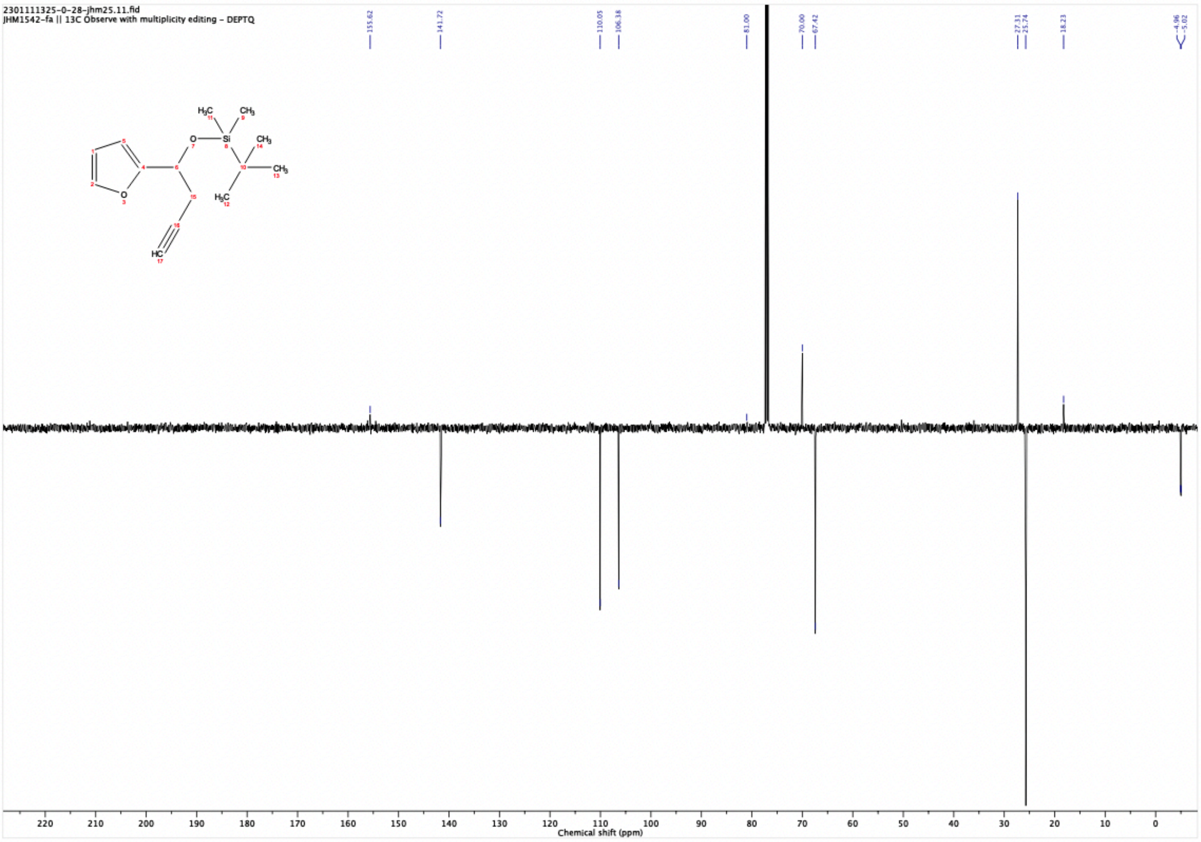

(S61)

^{1}H

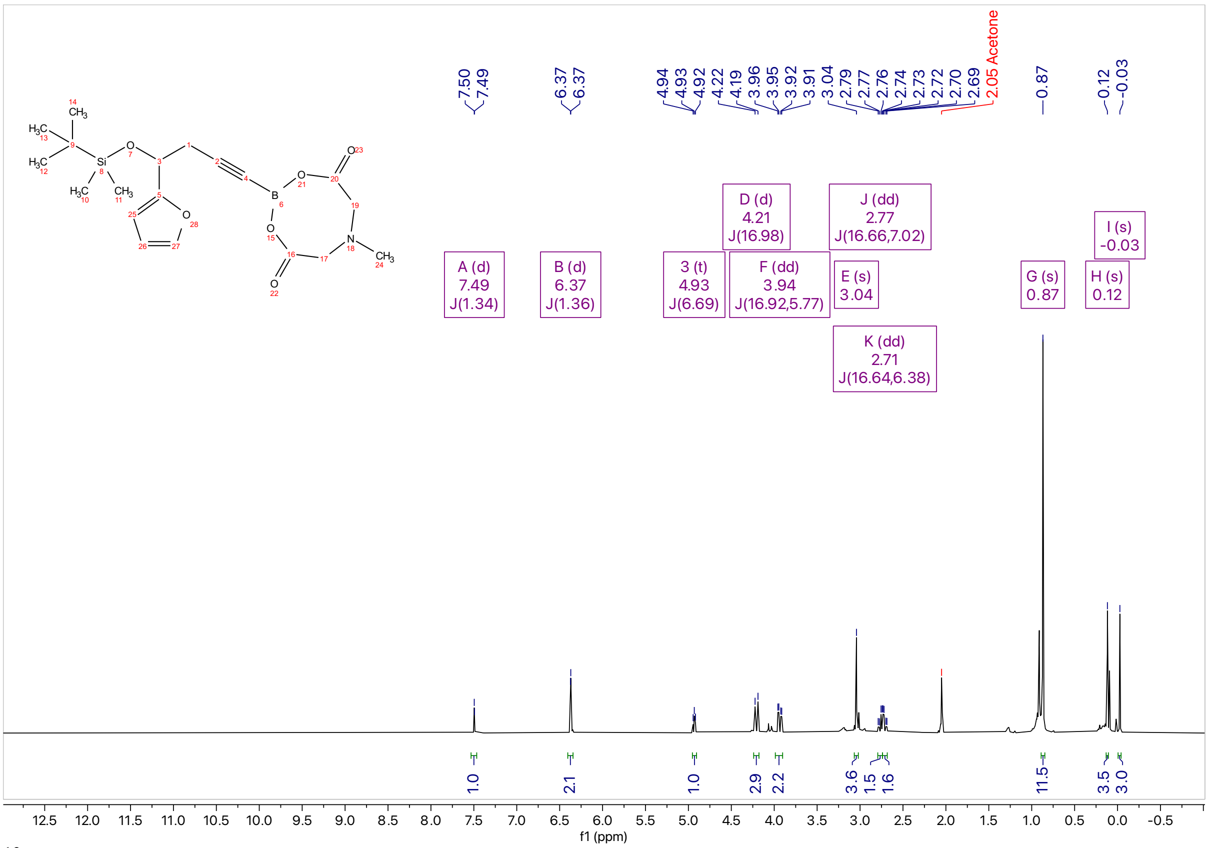
7.50
7.49
6.37
6.37
4.94
4.93
4.92
4.22
4.19
3.96
3.95
3.92
3.91
3.04
2.79
2.77
2.76
2.74
2.73
2.72
2.70
2.69
2.05 Acetone
0.87
0.12
-0.03
D (d) 4.21 J(16.98)
J (dd) 2.77 J(16.66,7.02)
I (s) -0.03
A (d) 7.49 J(1.34)
B (d) 6.37 J(1.36)
3 (t) 4.93 J(6.69)
F (dd) 3.94 J(16.92,5.77)
E (s) 3.04
G (s) 0.87
H (s) 0.12
K (dd) 2.71 J(16.64,6.38)
1.0
2.1
1.0
2.9
2.2
3.6
1.5
1.6
11.5
3.5
3.0
f1 (ppm)

^{13}C

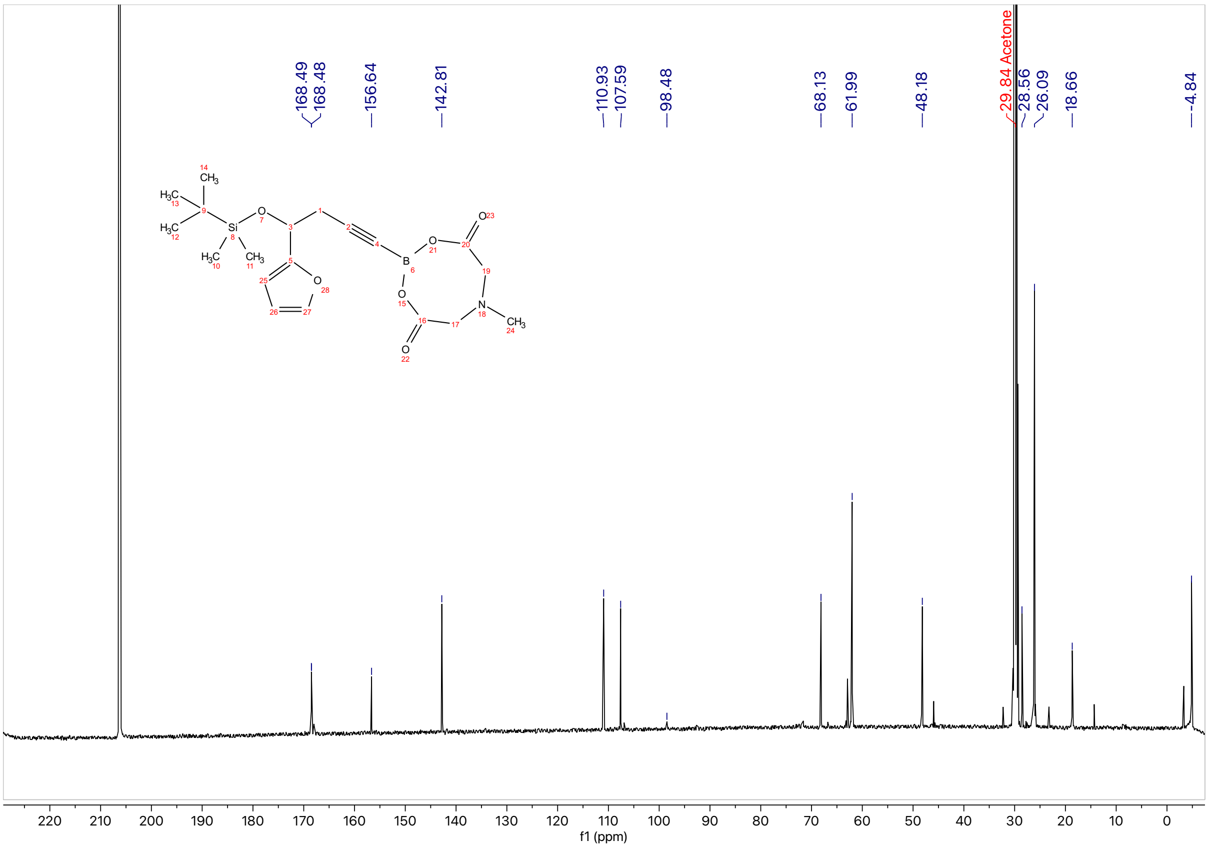
168.49
168.48
156.64
142.81
110.93
107.59
98.48
68.13
61.99
48.18
29.84 Acetone
28.56
26.09
18.66
-4.84
f1 (ppm)

(S62)

^{1}H

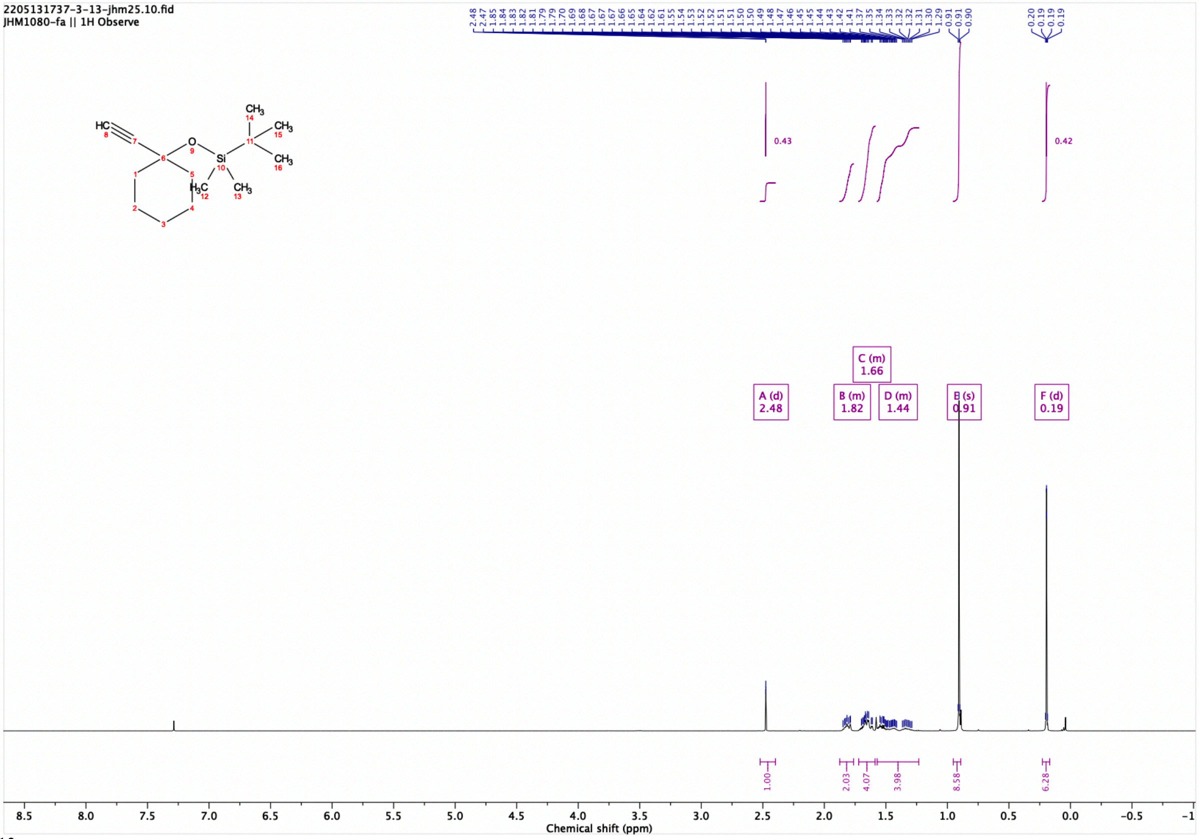

^{13}C DEPTQ

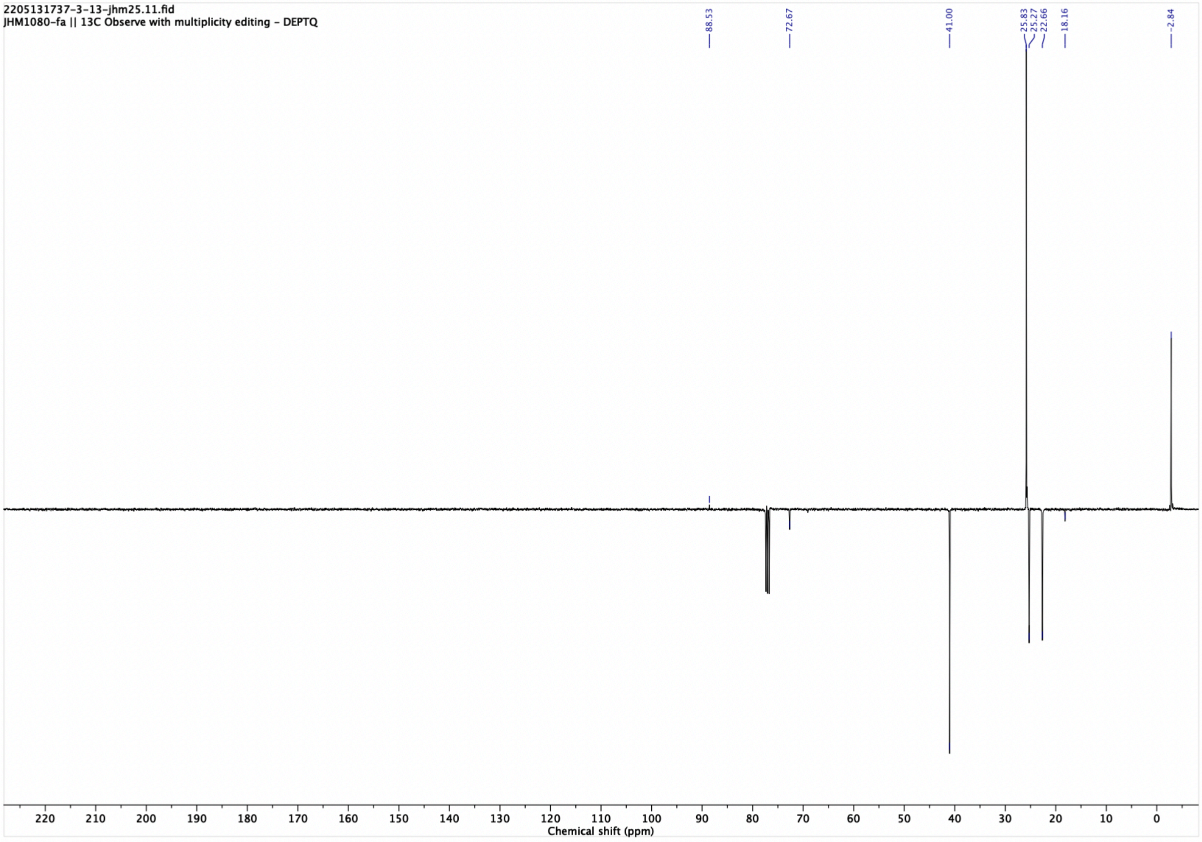

(S63)

^{1}H

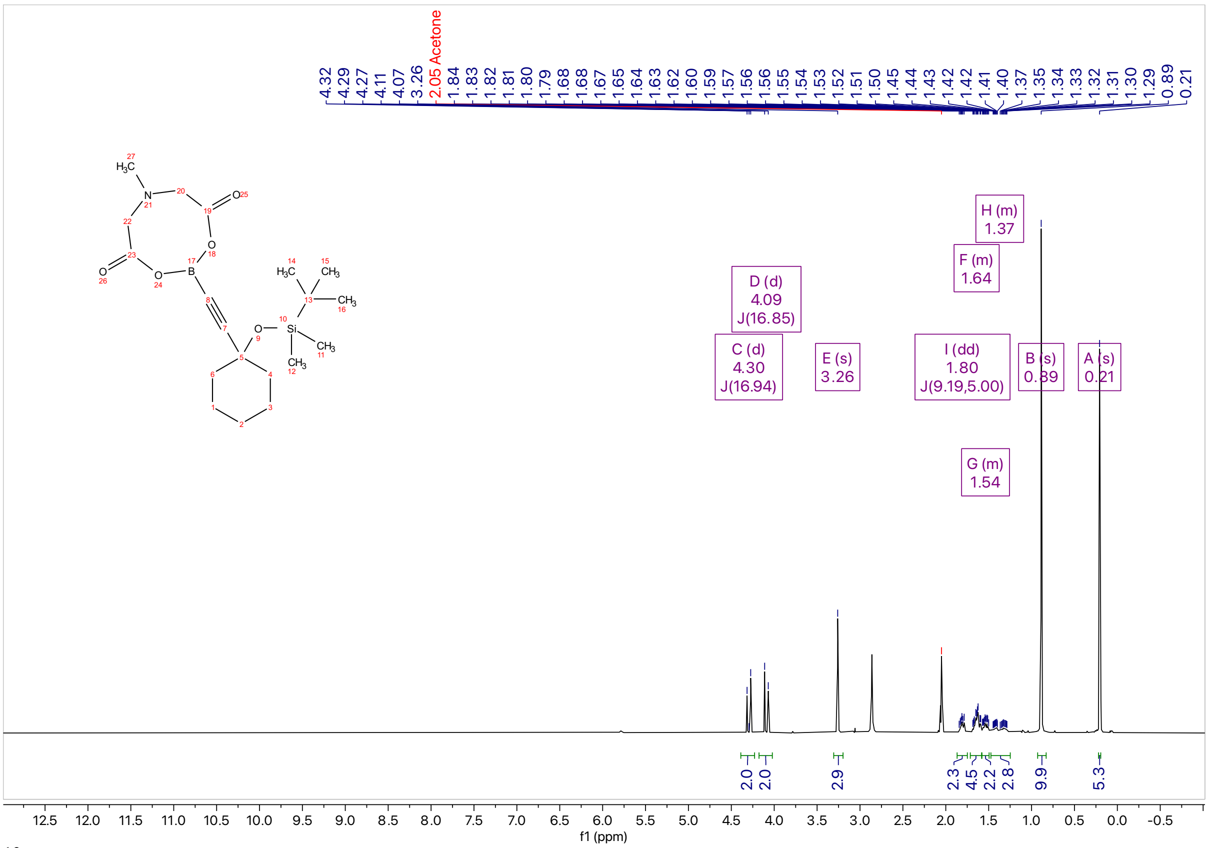

4.32
4.29
4.27
4.11
4.07
3.26
2.05 Acetone
1.84
1.83
1.82
1.81
1.80
1.79
1.68
1.68
1.67
1.65
1.64
1.63
1.62
1.60
1.59
1.57
1.56
1.56
1.55
1.54
1.53
1.52
1.51
1.50
1.45
1.44
1.43
1.42
1.42
1.41
1.40
1.37
1.35
1.34
1.33
1.32
1.31
1.30
1.29
0.89
0.21
H (m) 1.37
F (m) 1.64
D (d) 4.09 J(16.85)
C (d) 4.30 J(16.94)
E (s) 3.26
I (dd) 1.80 J(9.19,5.00)
B (s) 0.89
A (s) 0.21
G (m) 1.54
2.0
2.0
2.9
2.3
4.5
2.2
2.8
9.9
5.3
f1 (ppm)

^{13}C

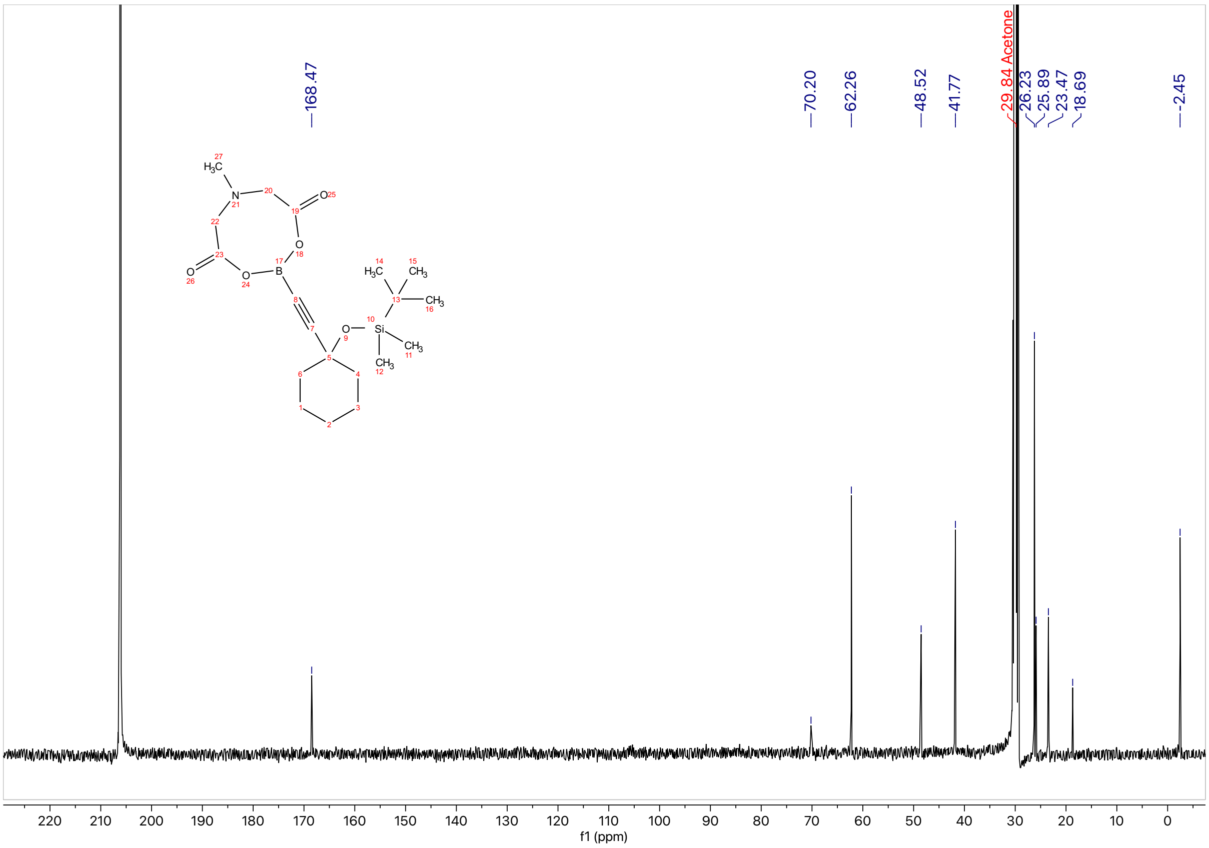

168.47
70.20
62.26
48.52
41.77
29.84 Acetone
26.23
25.89
23.47
18.69
-2.45
f1 (ppm)

(S65)

^{1}H

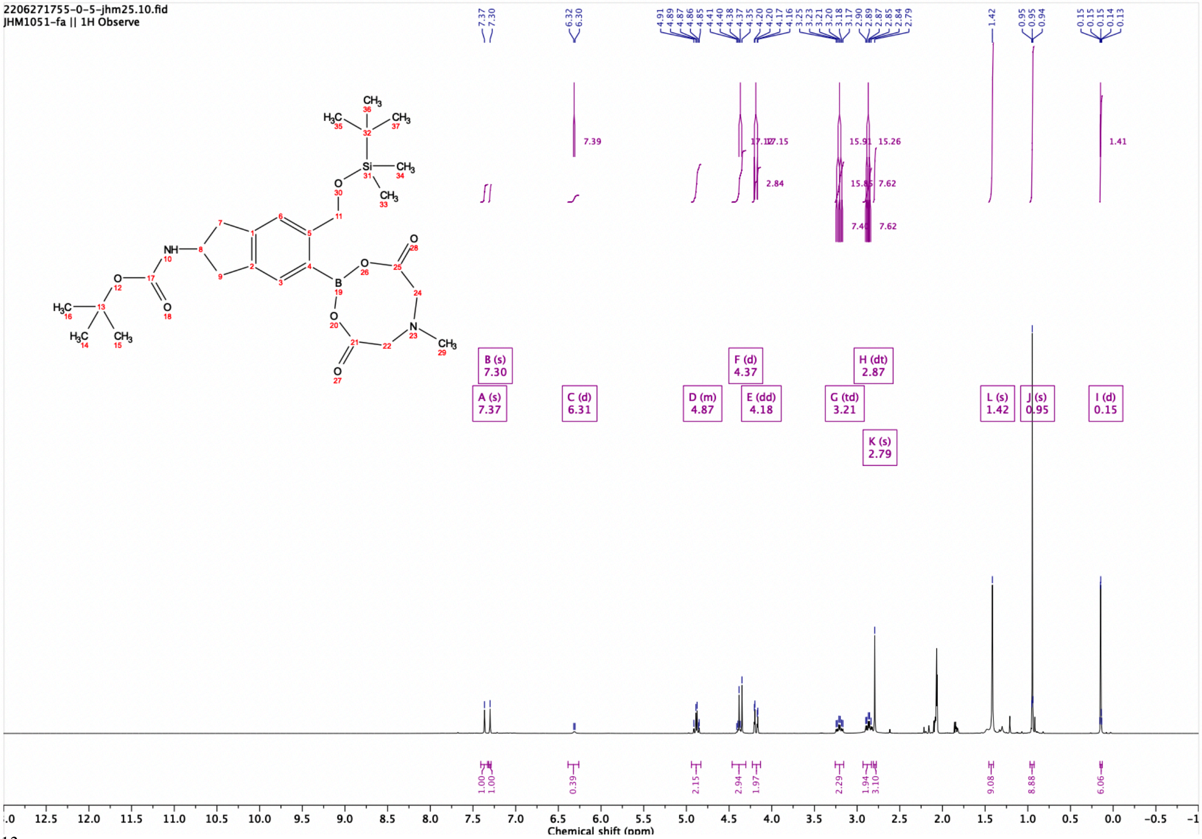

^{13}C DEPTQ

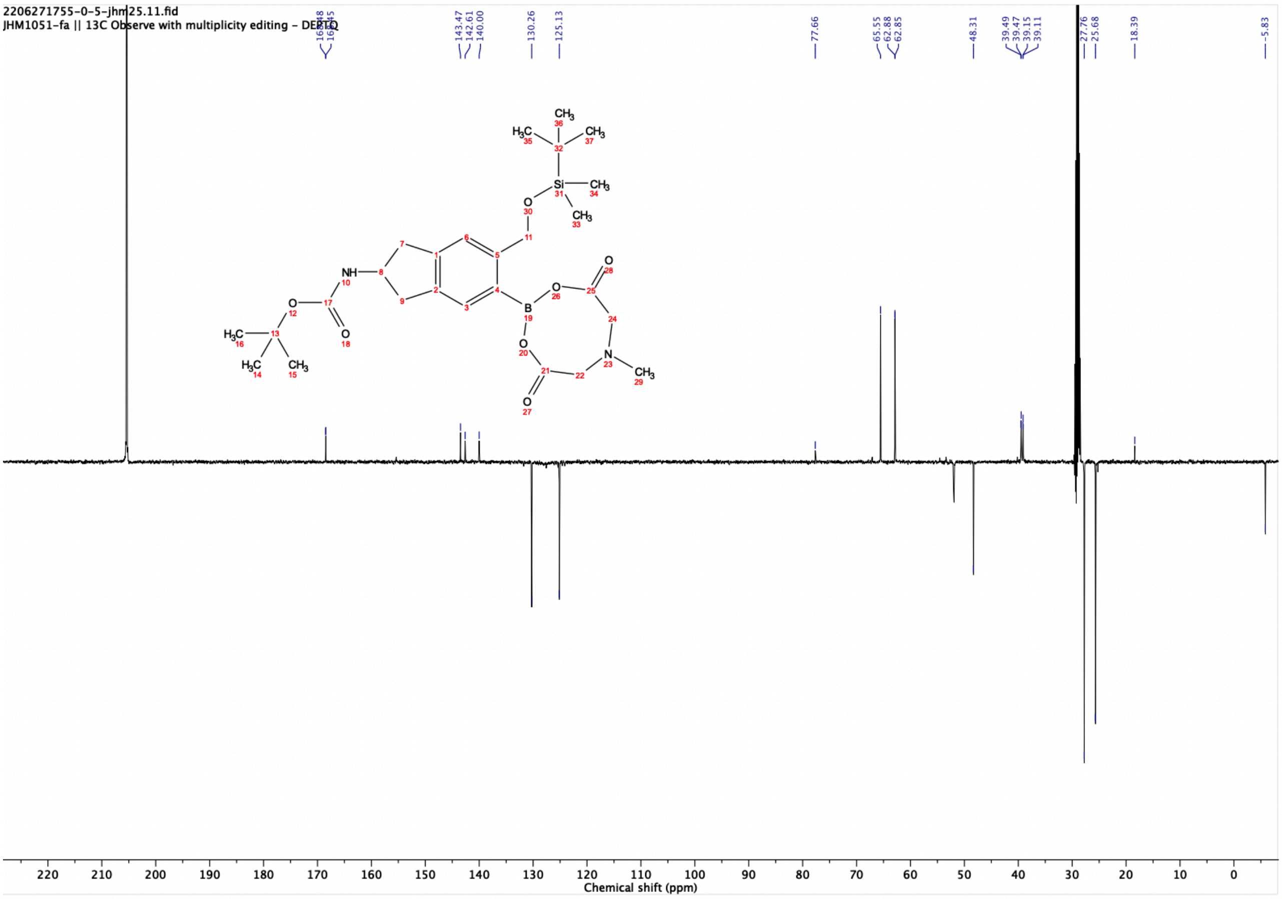

(S66)

^{1}H

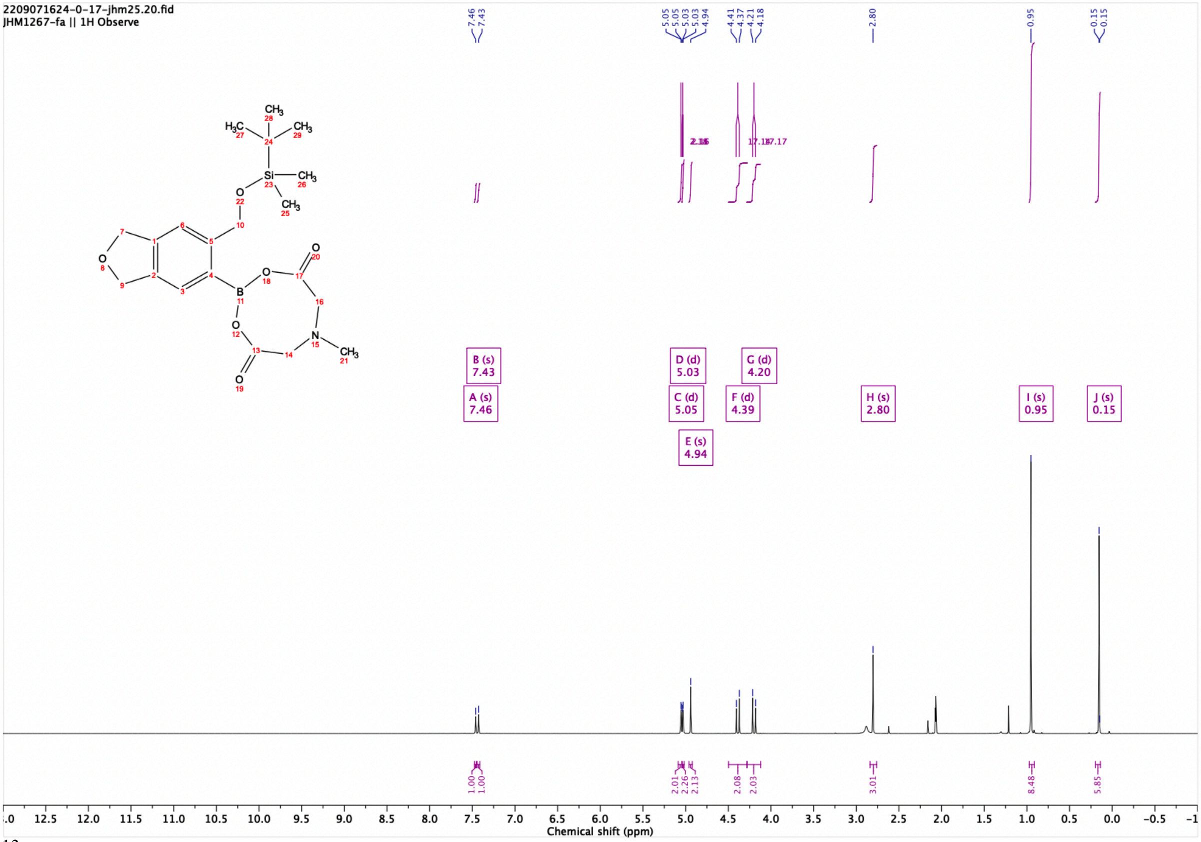

2209071624-0-17-jhm25.20.fid
JHM1267-fa || 1H Observe
A (s) 7.46
B (s) 7.43
C (d) 5.05
D (d) 5.03
E (s) 4.94
F (d) 4.39
G (d) 4.20
H (s) 2.80
I (s) 0.95
J (s) 0.15
Chemical shift (ppm)

^{13}C

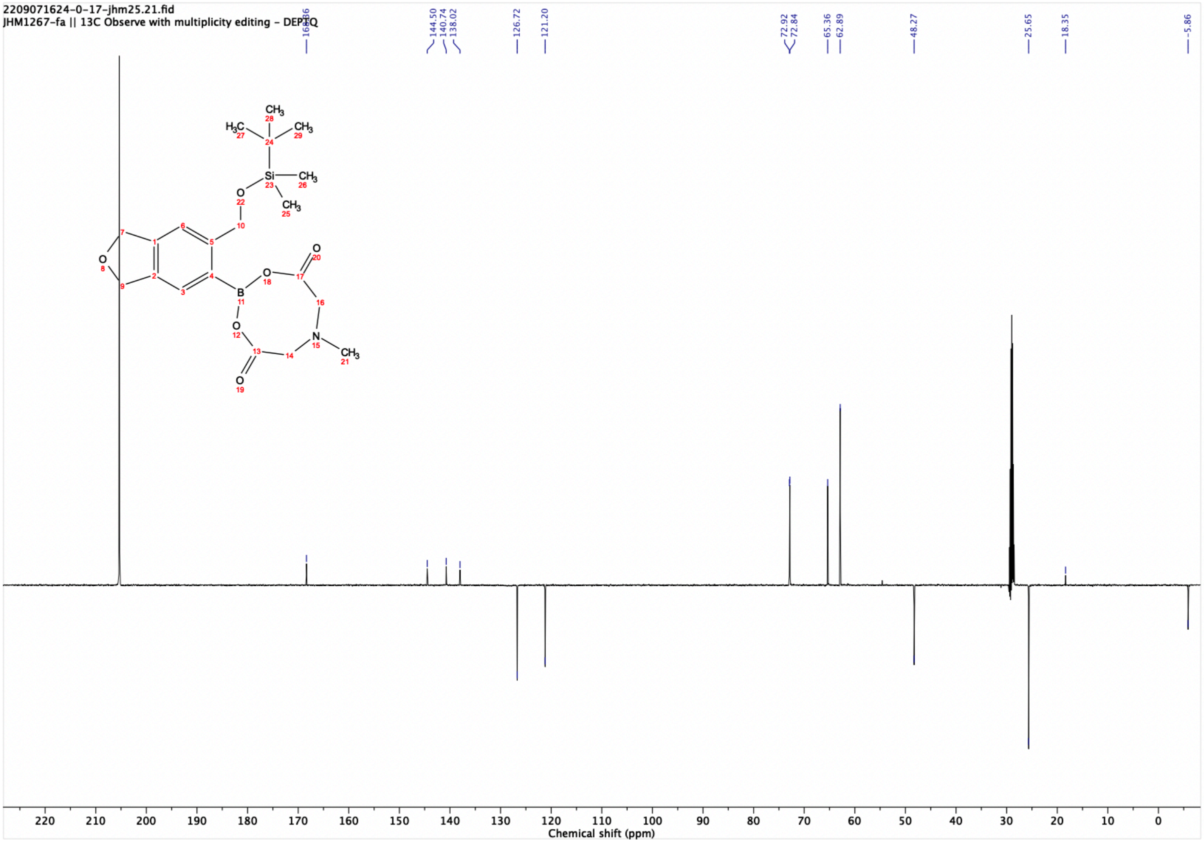

2209071624-0-17-jhm25.21.fid
JHM1267-fa || 13C Observe with multiplicity editing - DEPTQ
144.50
140.74
138.02
126.72
121.20
72.92
72.84
65.36
62.89
48.27
25.65
18.35
-5.86
Chemical shift (ppm)

(S67)

^{1}H

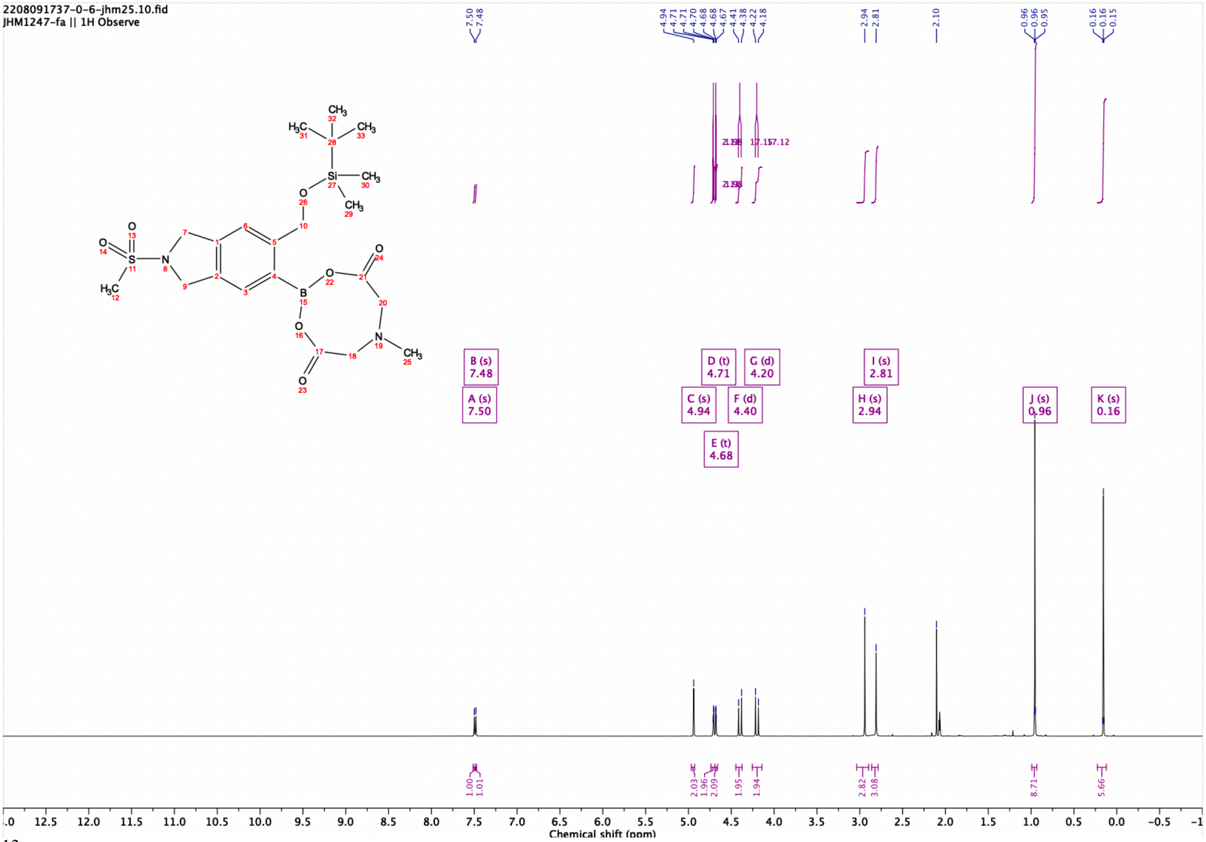

^{13}C DEPTQ

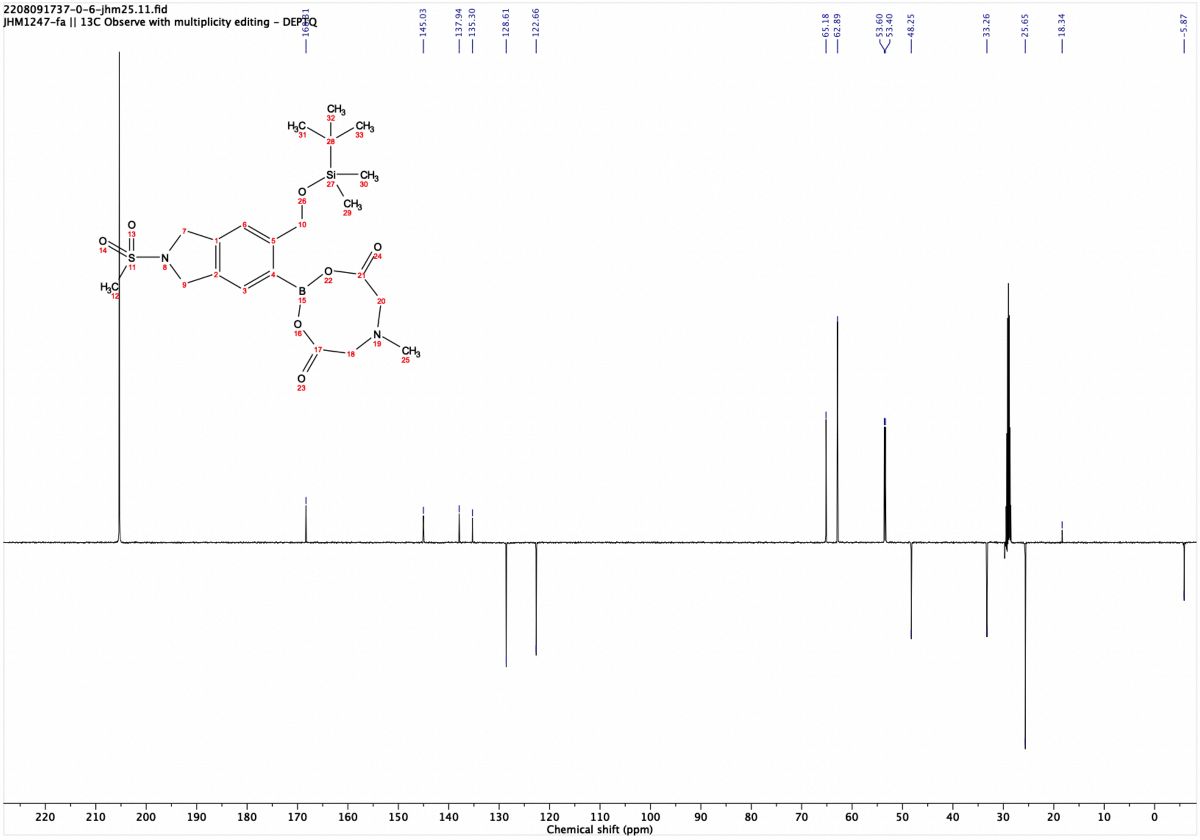

(S68)

^{1}H

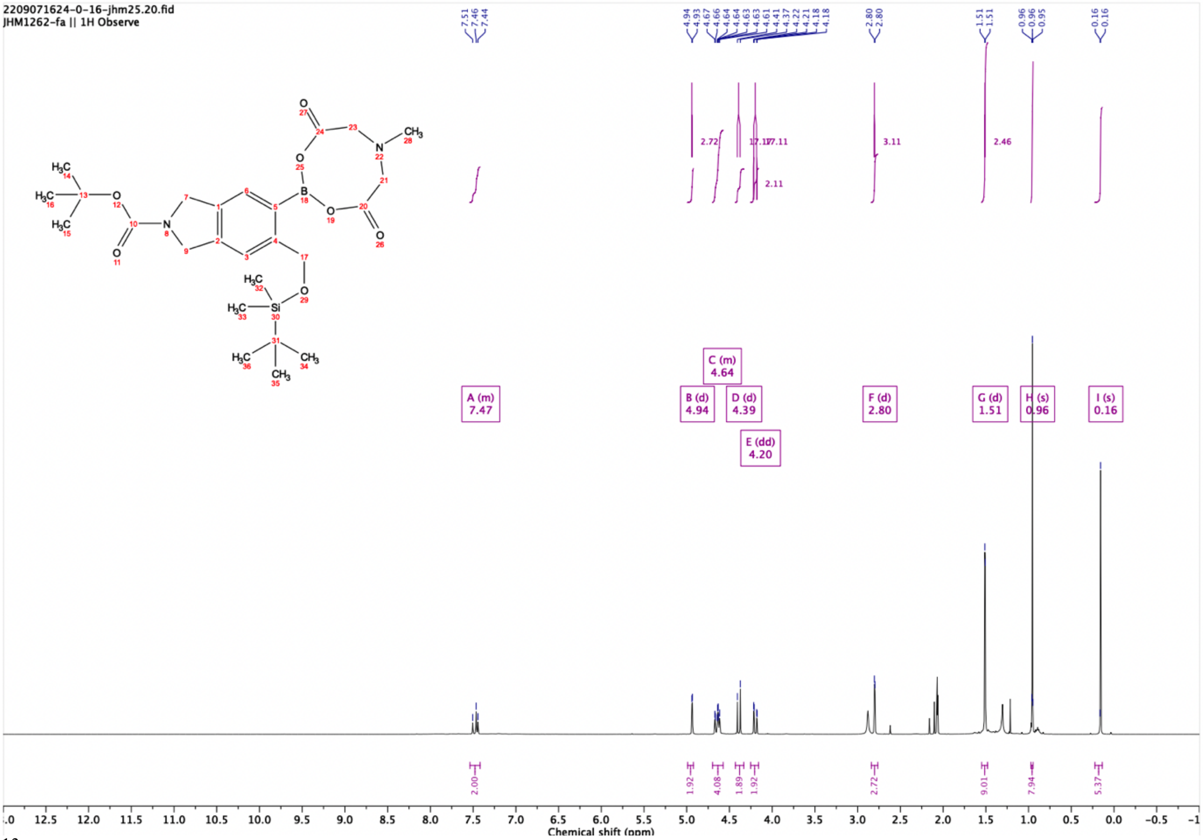

^{13}C DEPTQ

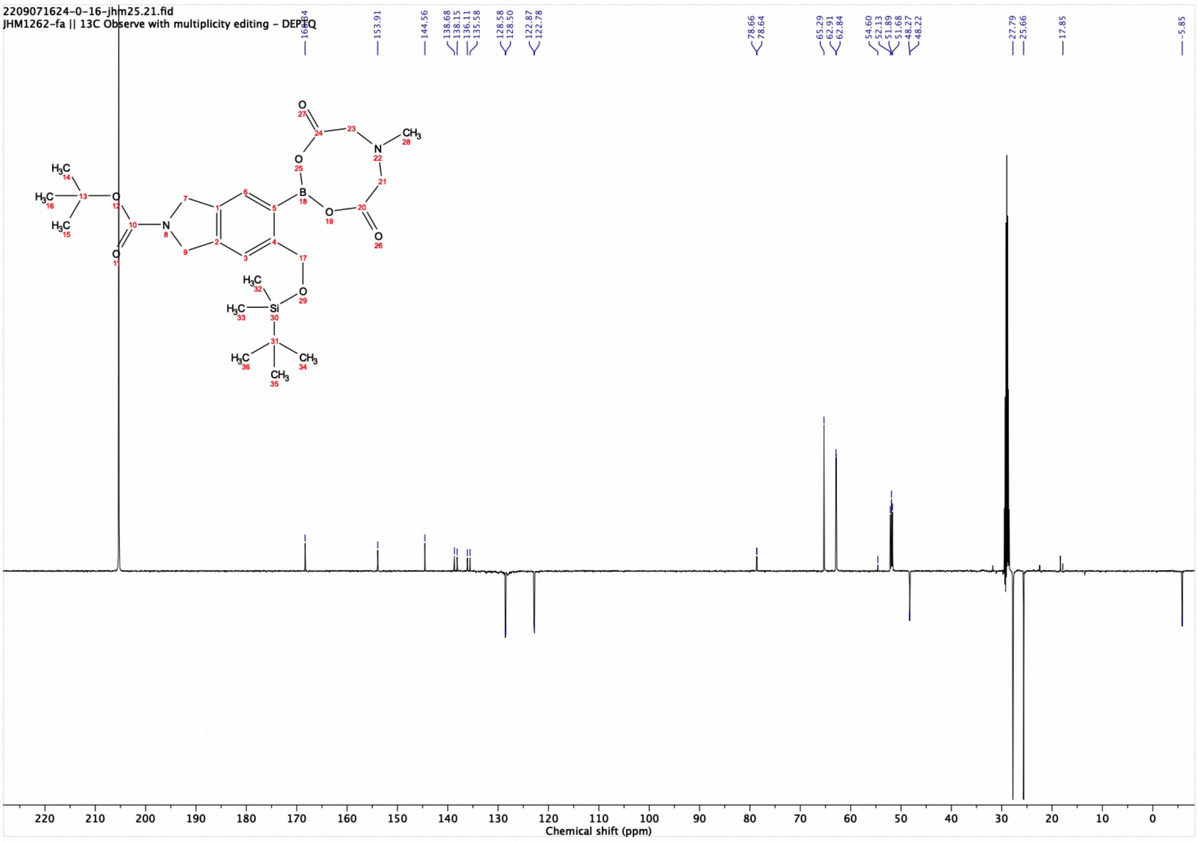

(S69)

^{1}H

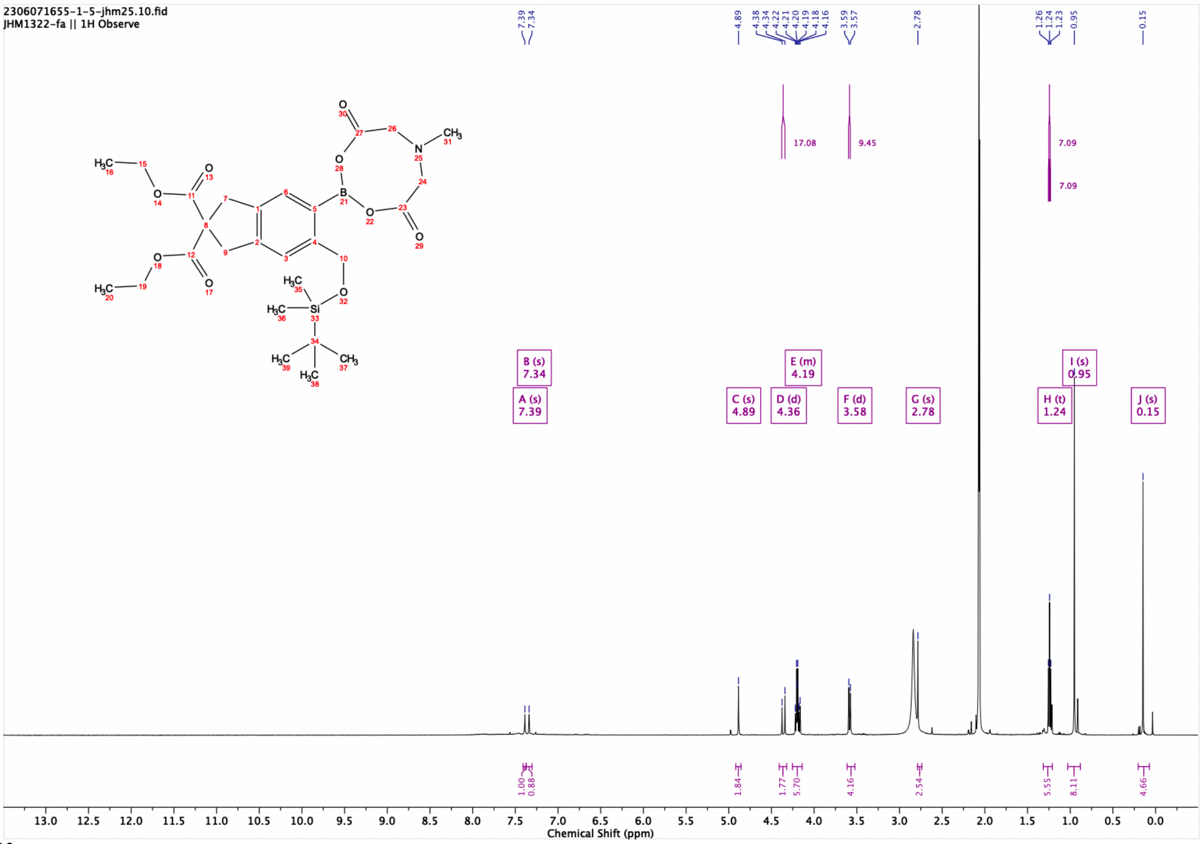

^{13}C DEPTQ

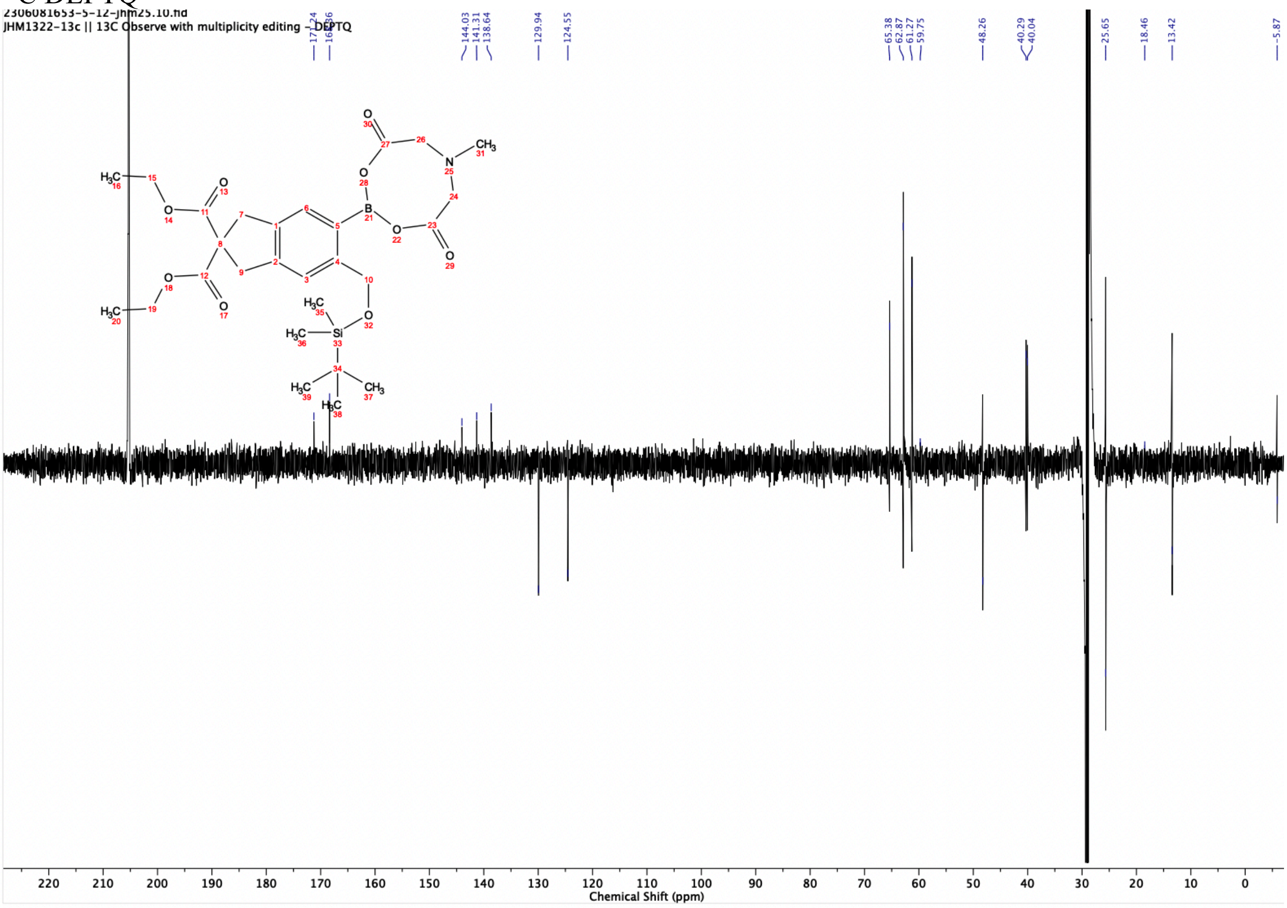

(S70)

^{1}H

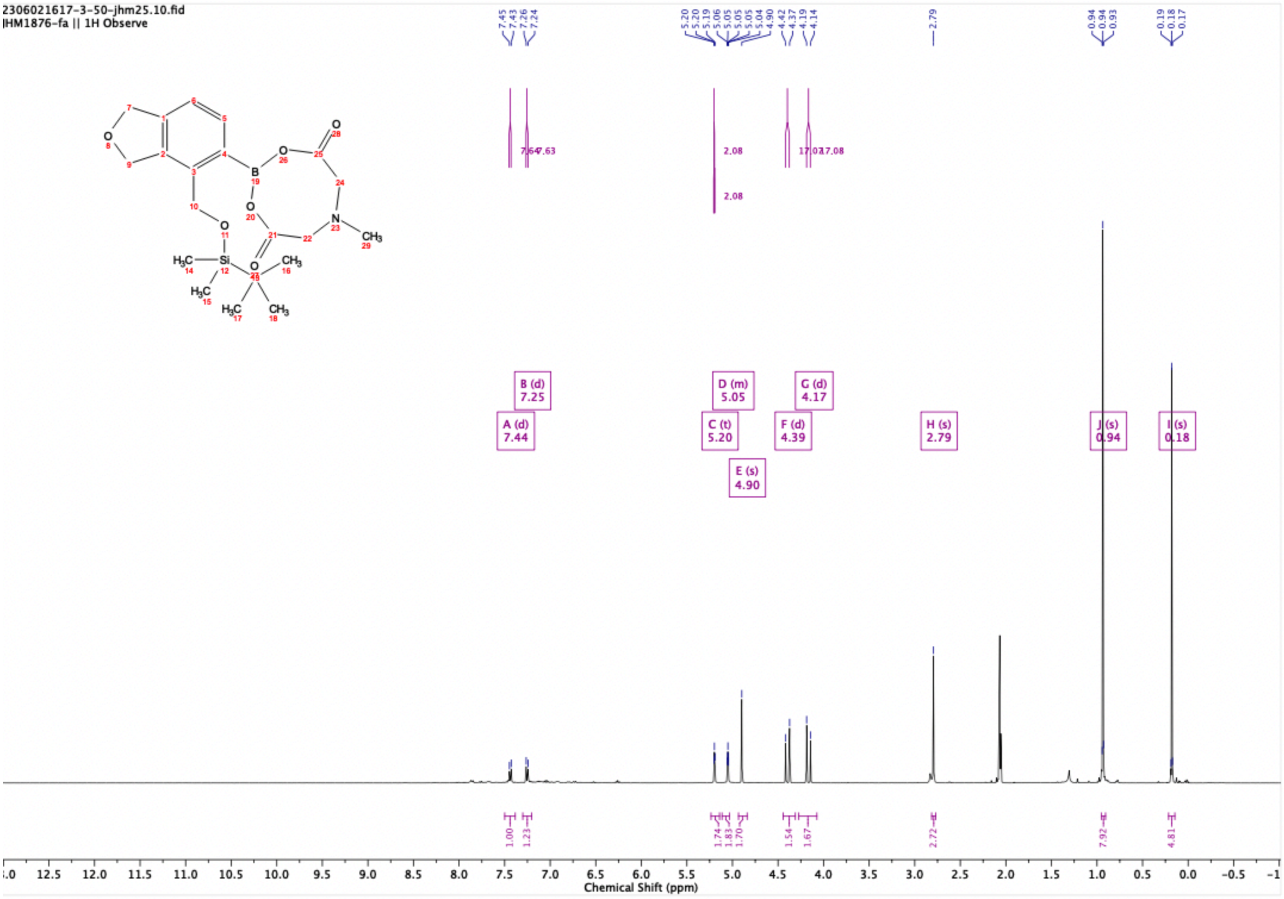

^{13}C DEPTQ

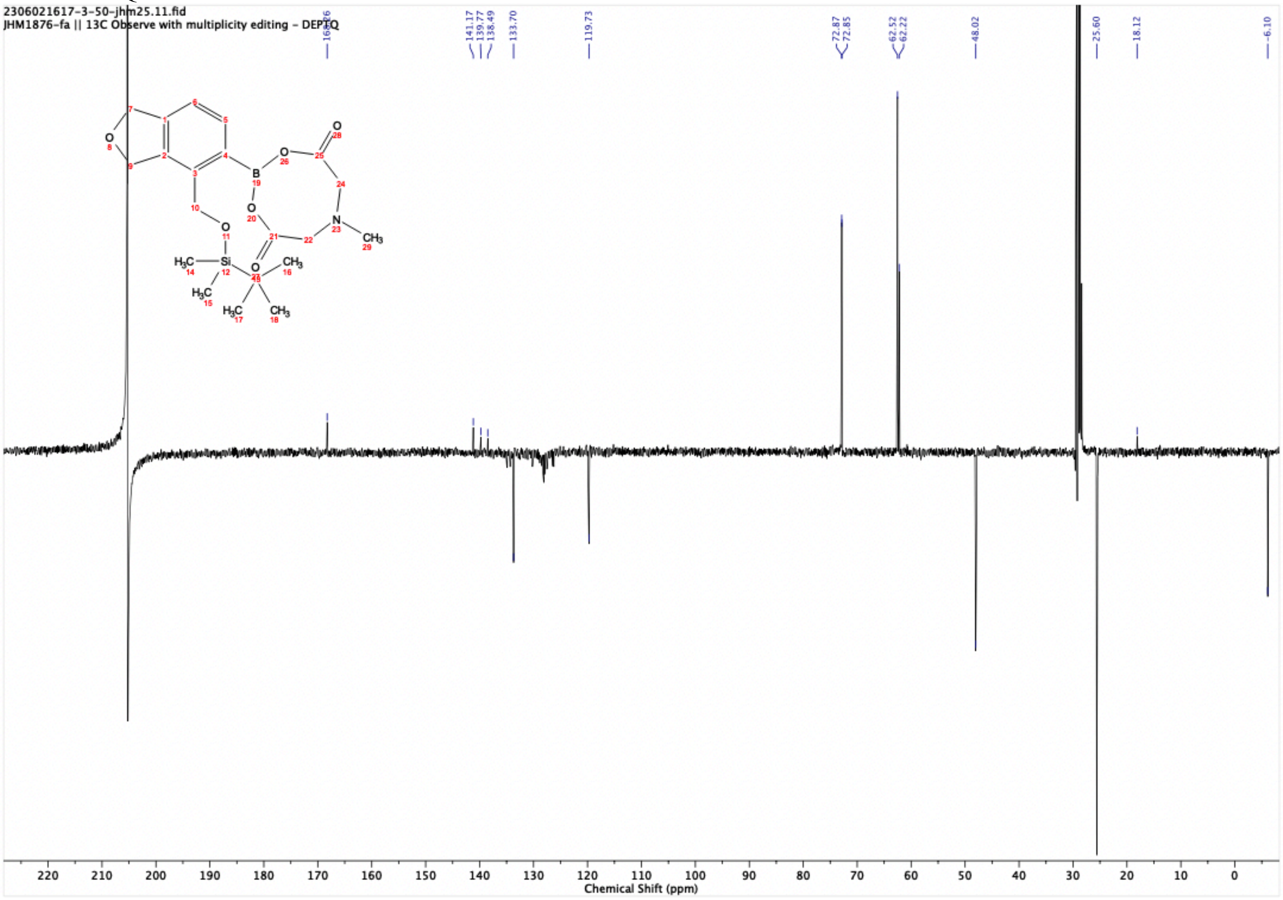

(S71)

^{1}H

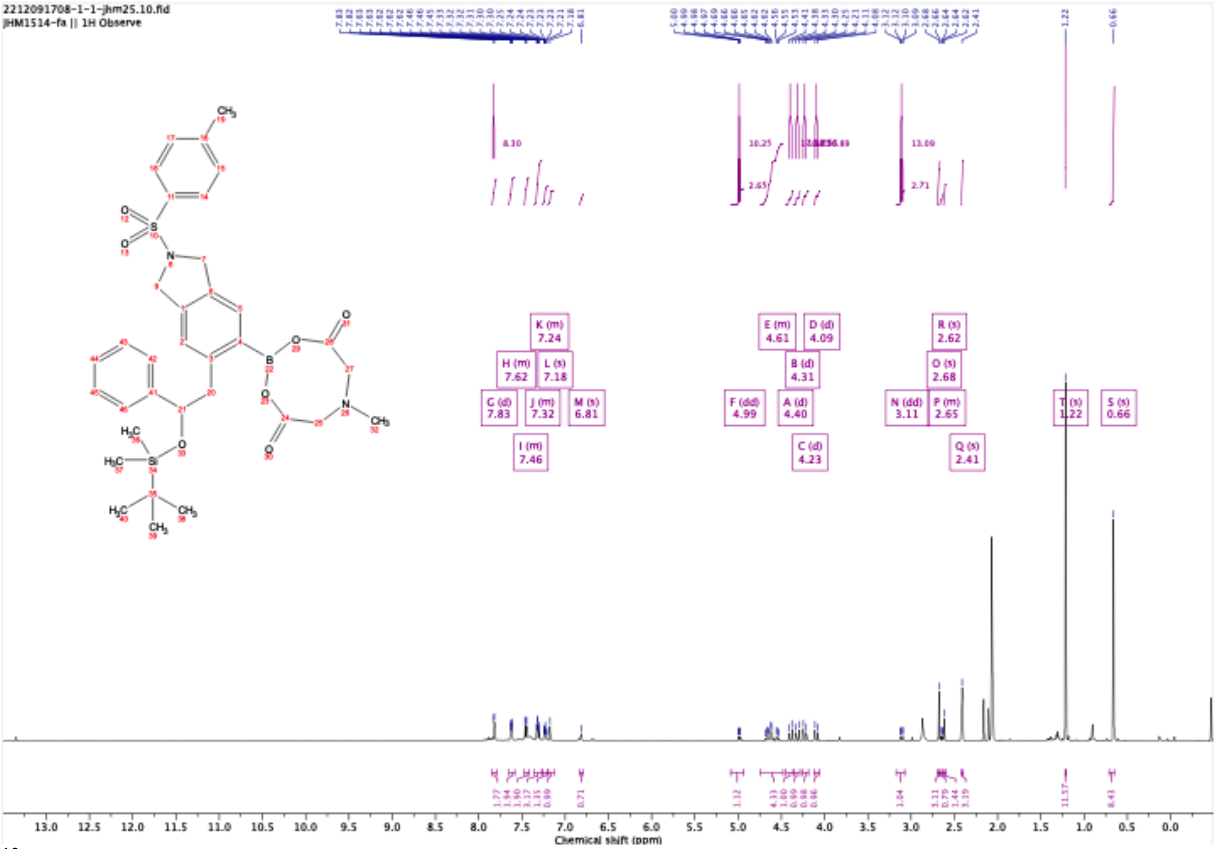

^{13}C DEPTQ

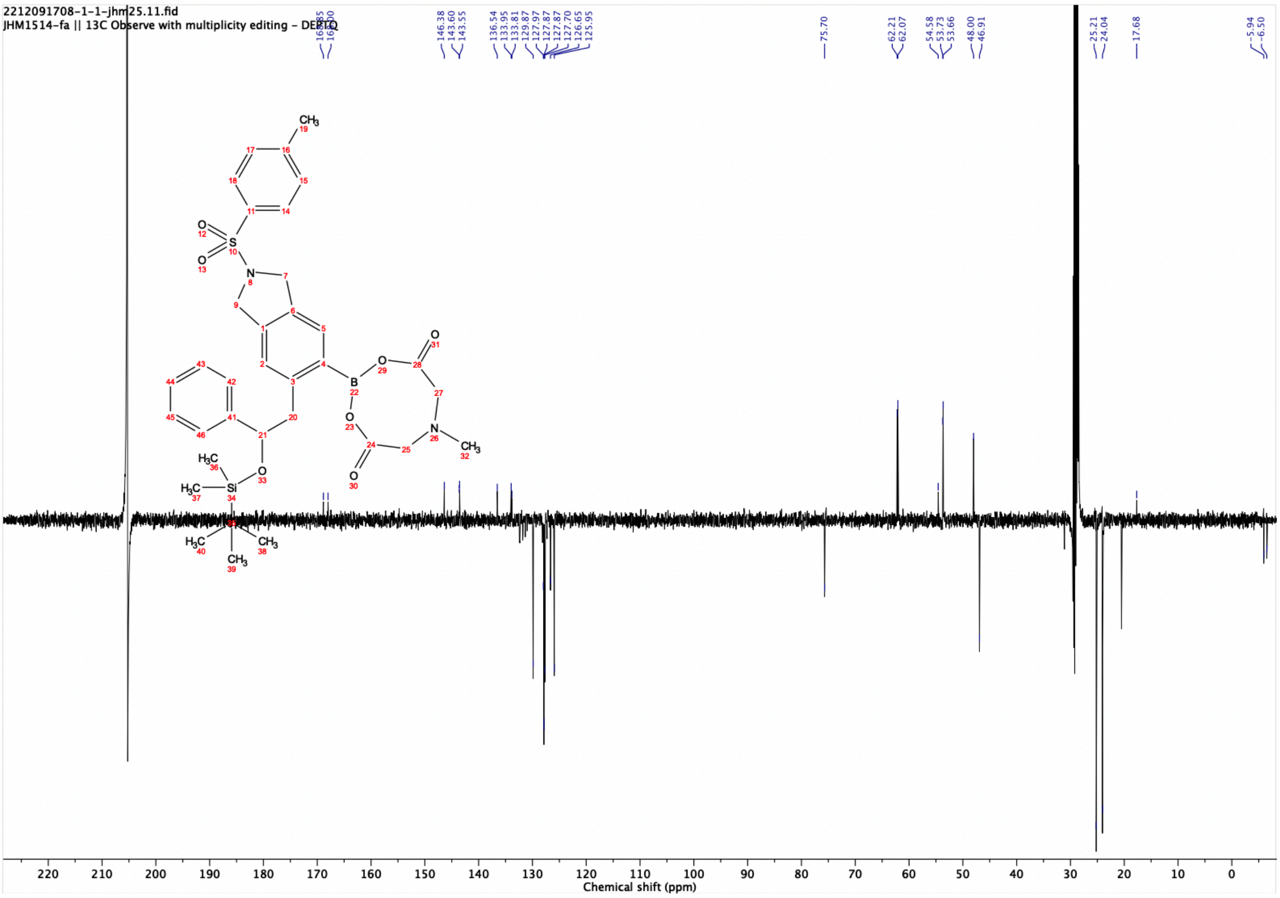

(S72)

^{1}H

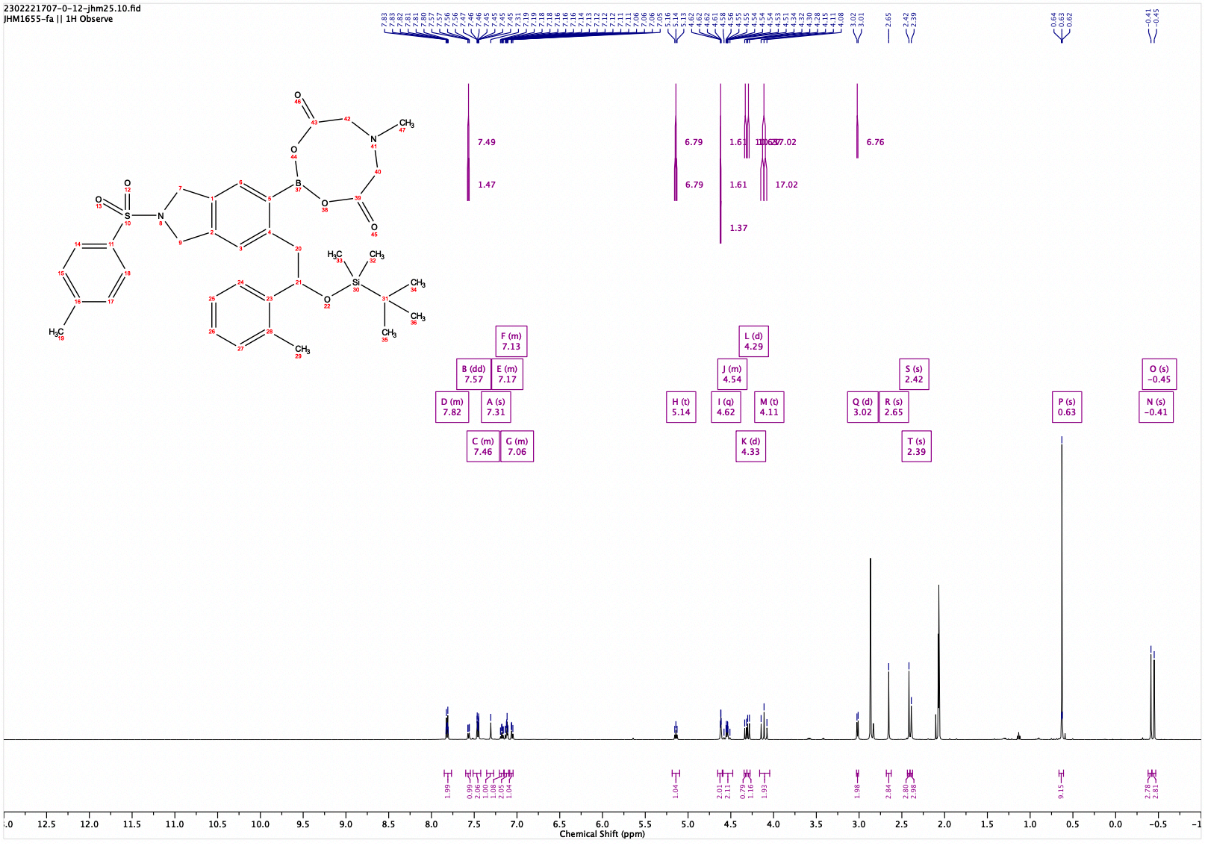

^{13}C DEPTQ

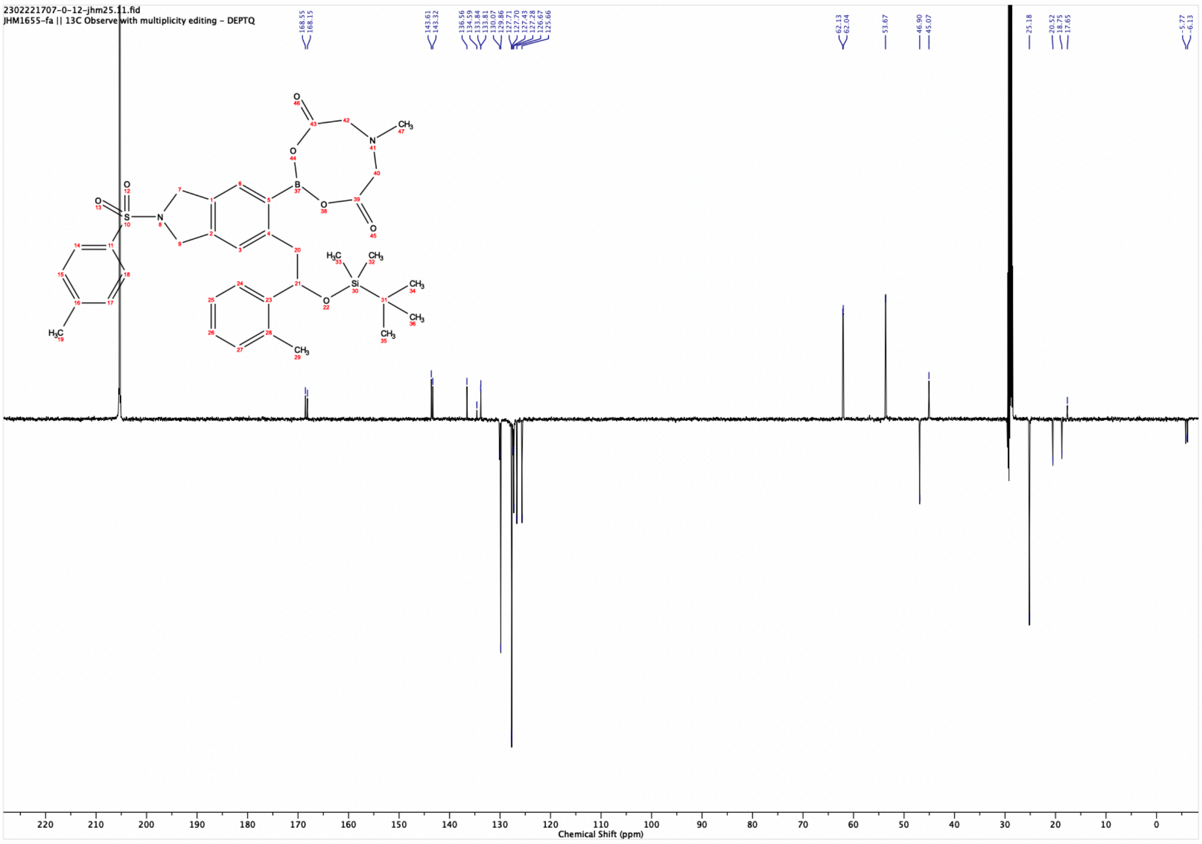

(S73)

^{1}H

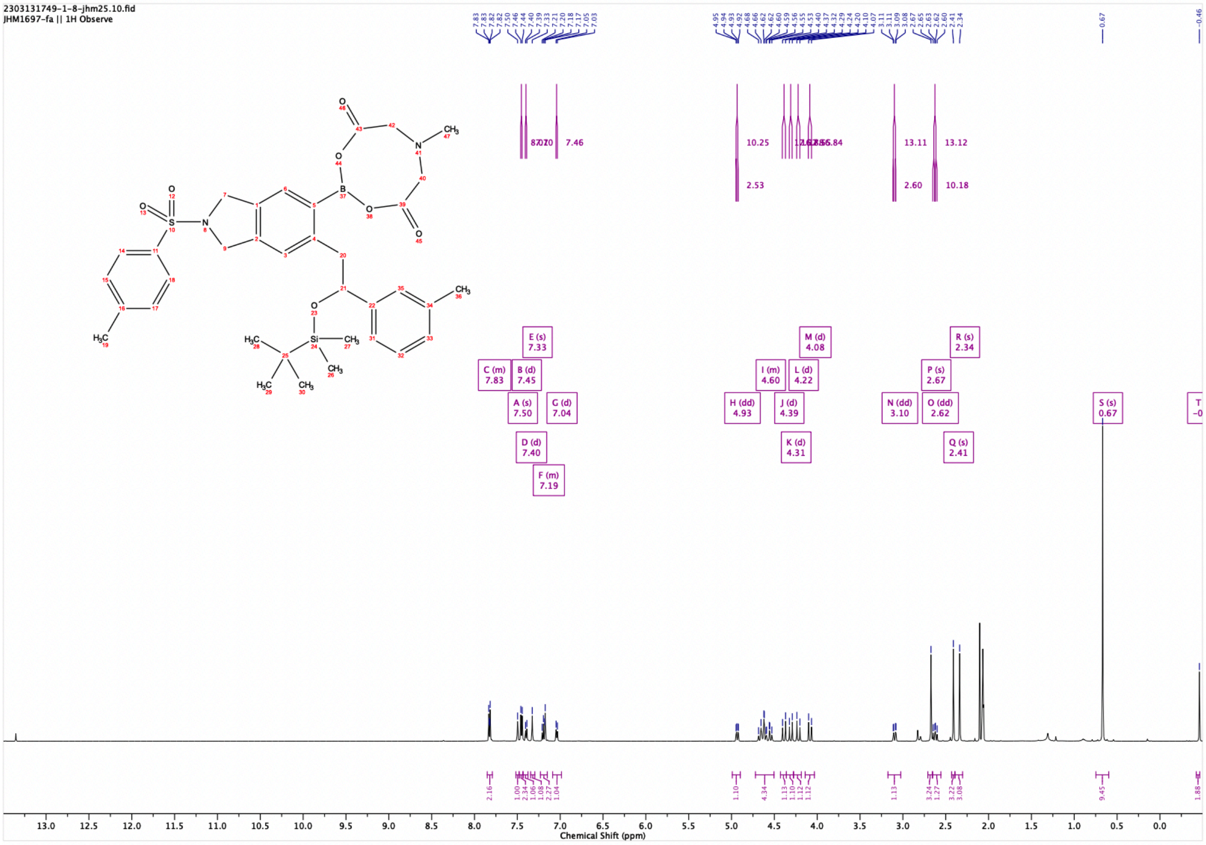

^{13}C DEPTQ

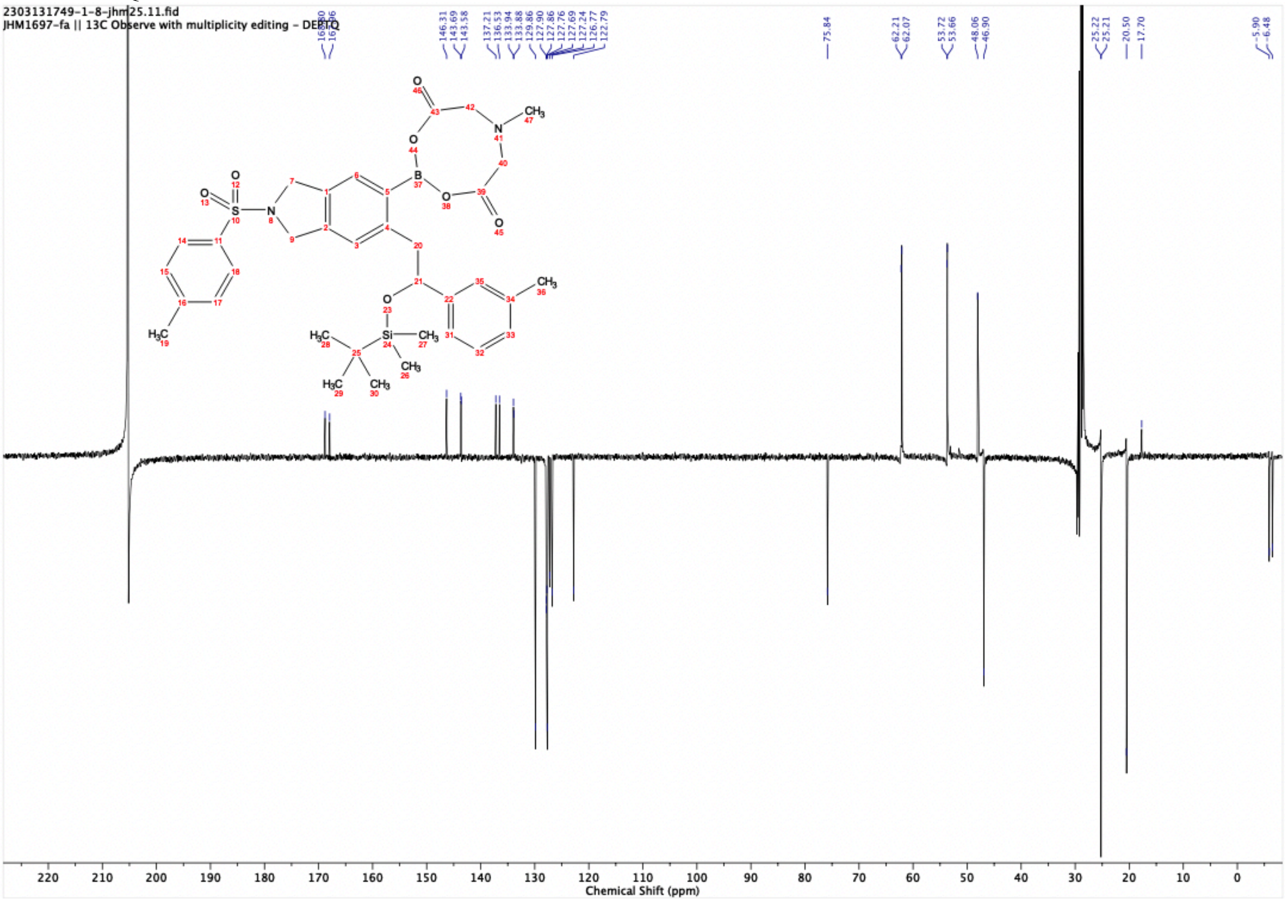

(S74)

^{1}H

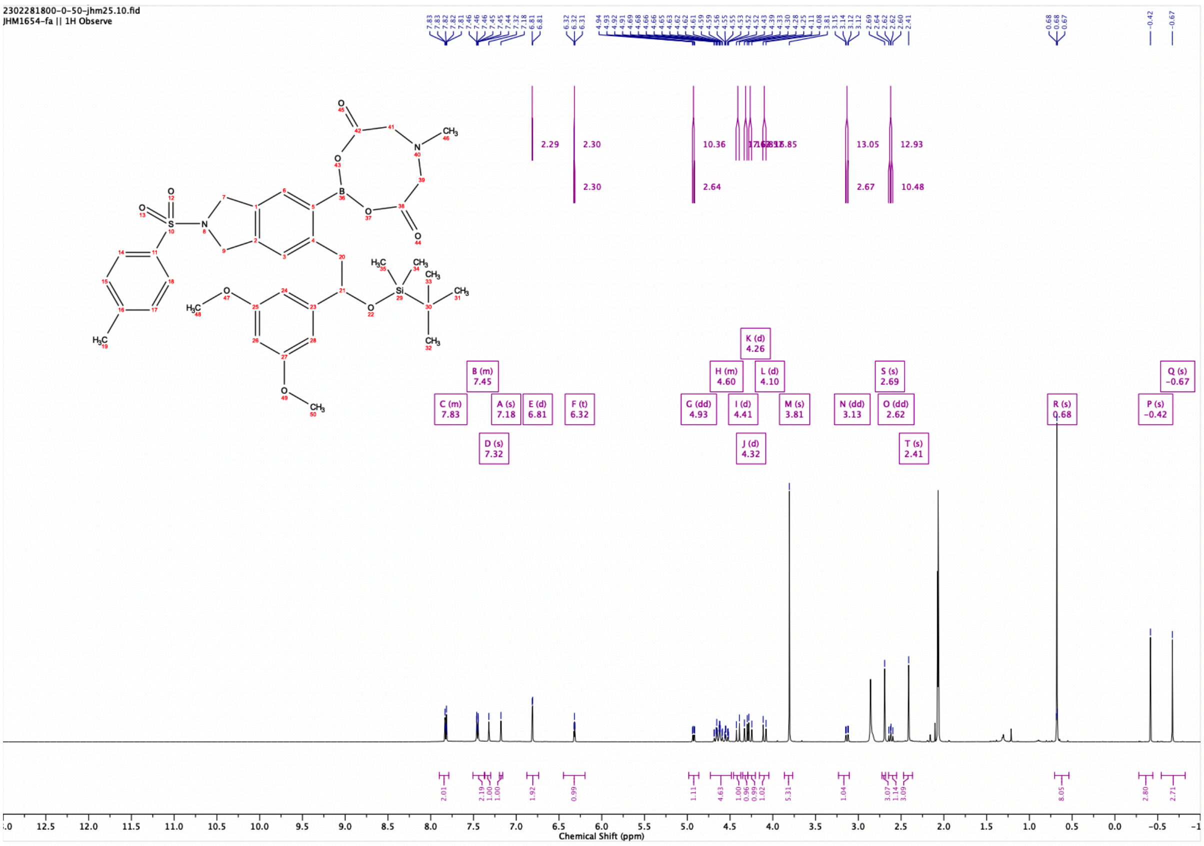

^{13}C DEPTQ

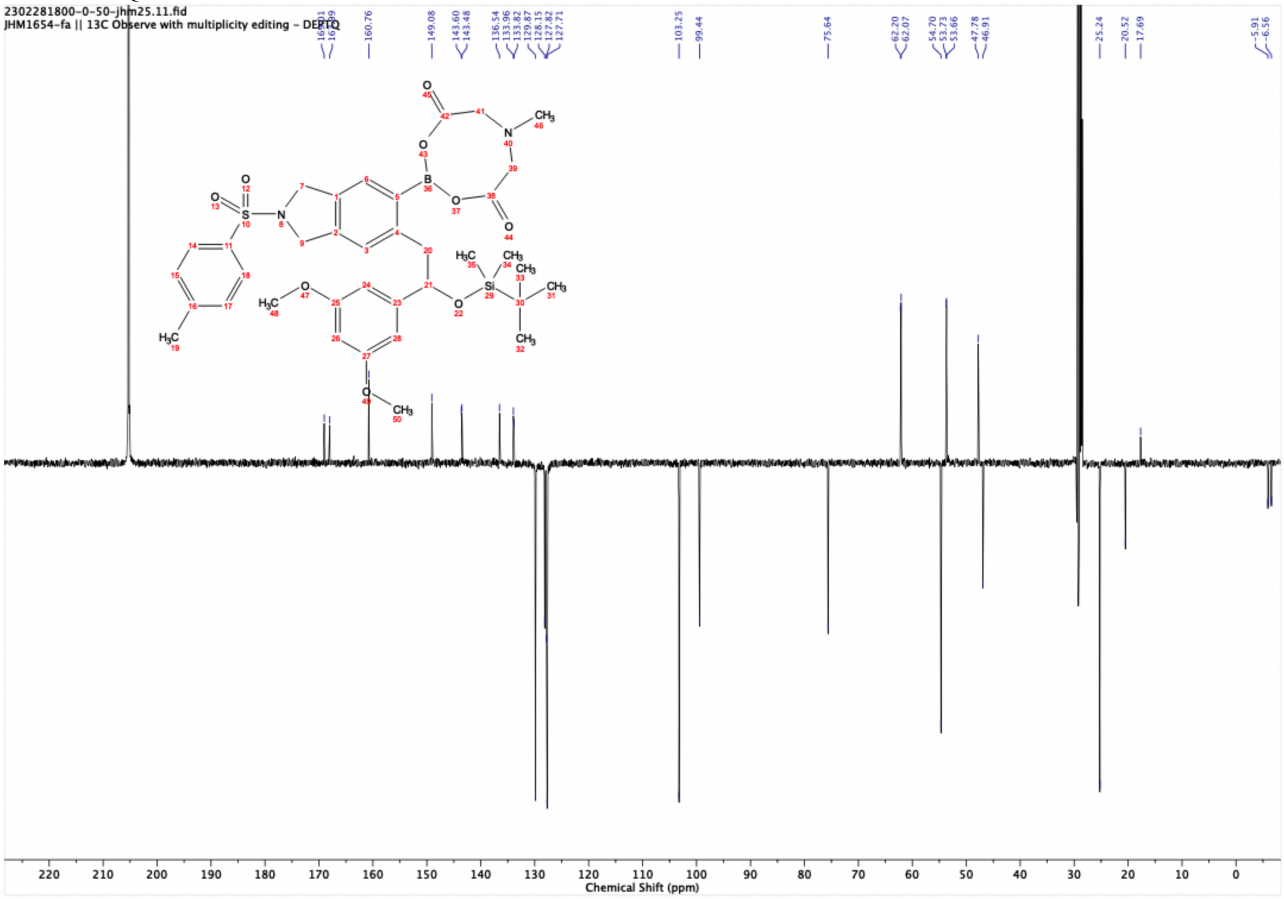

(S75)

^{1}H

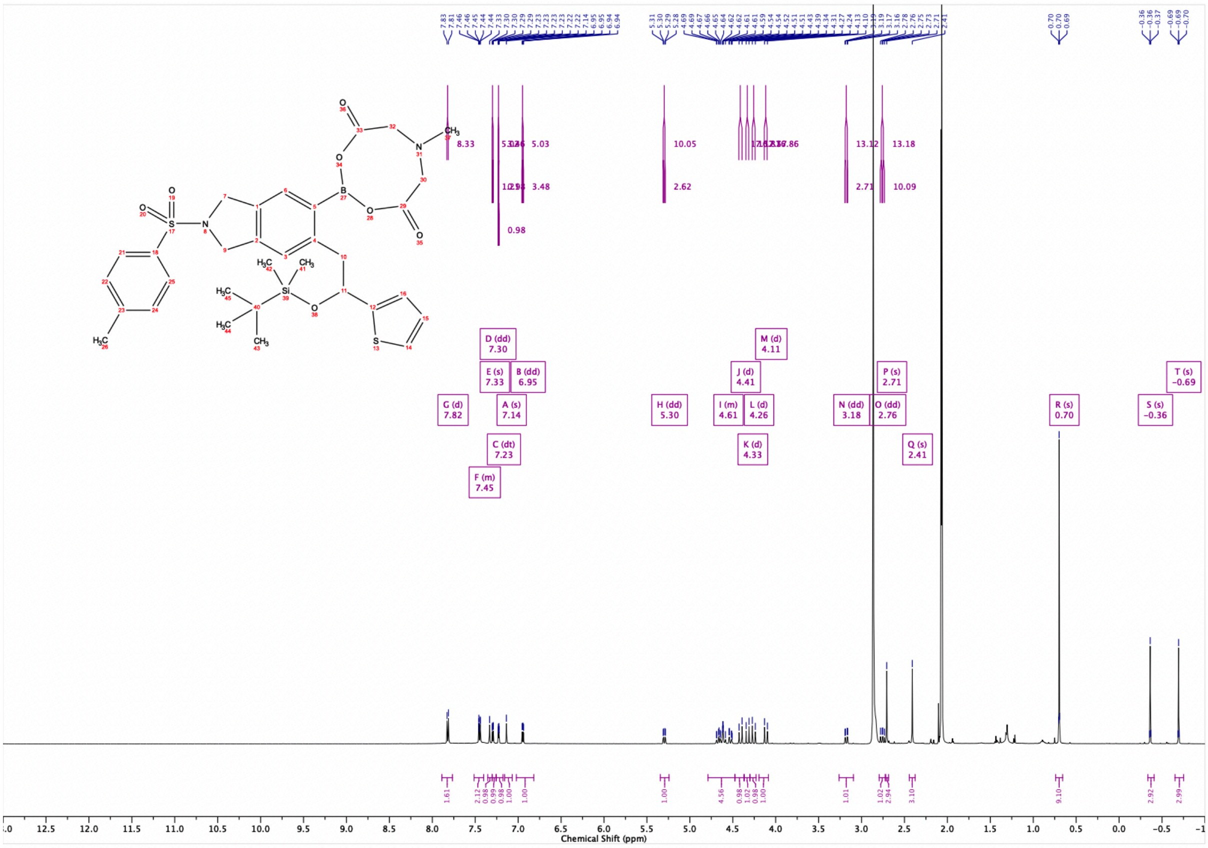

^{13}C DEPTQ

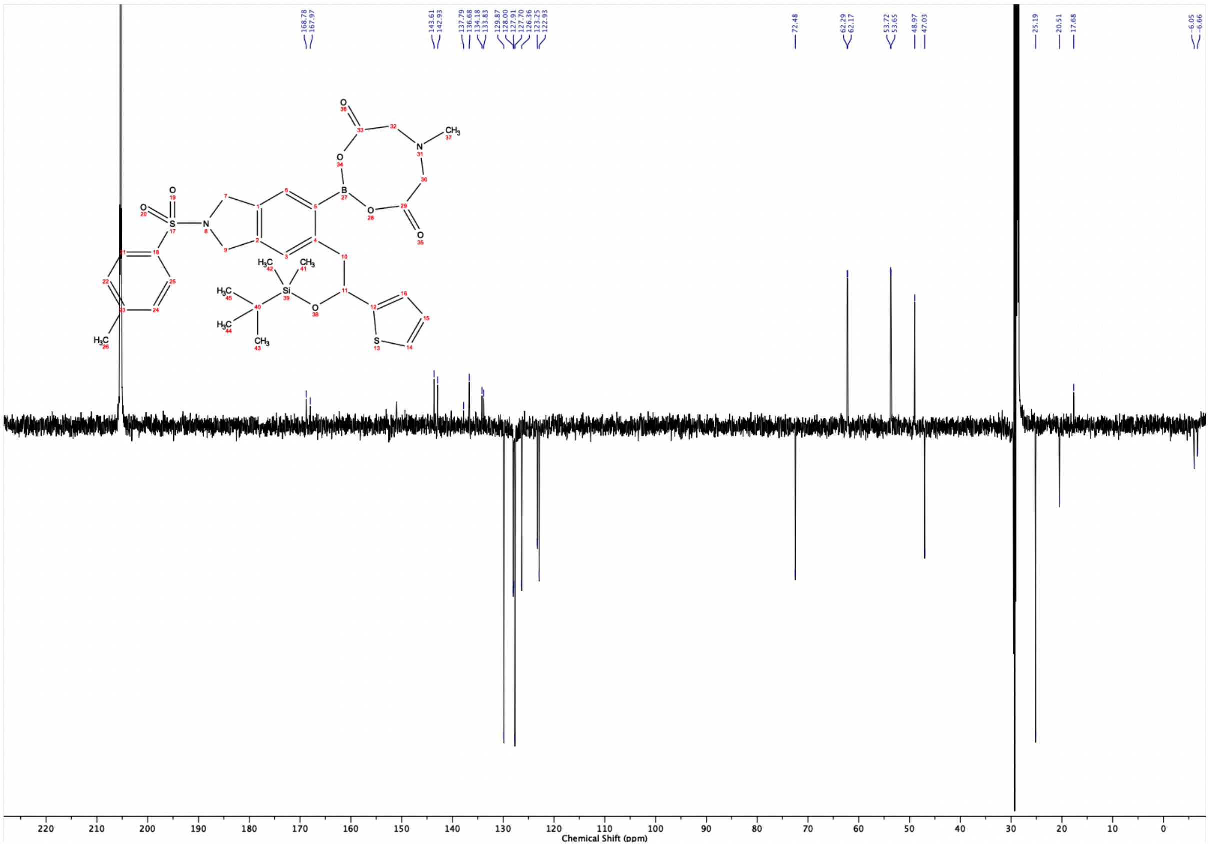

(S76)

^{1}H

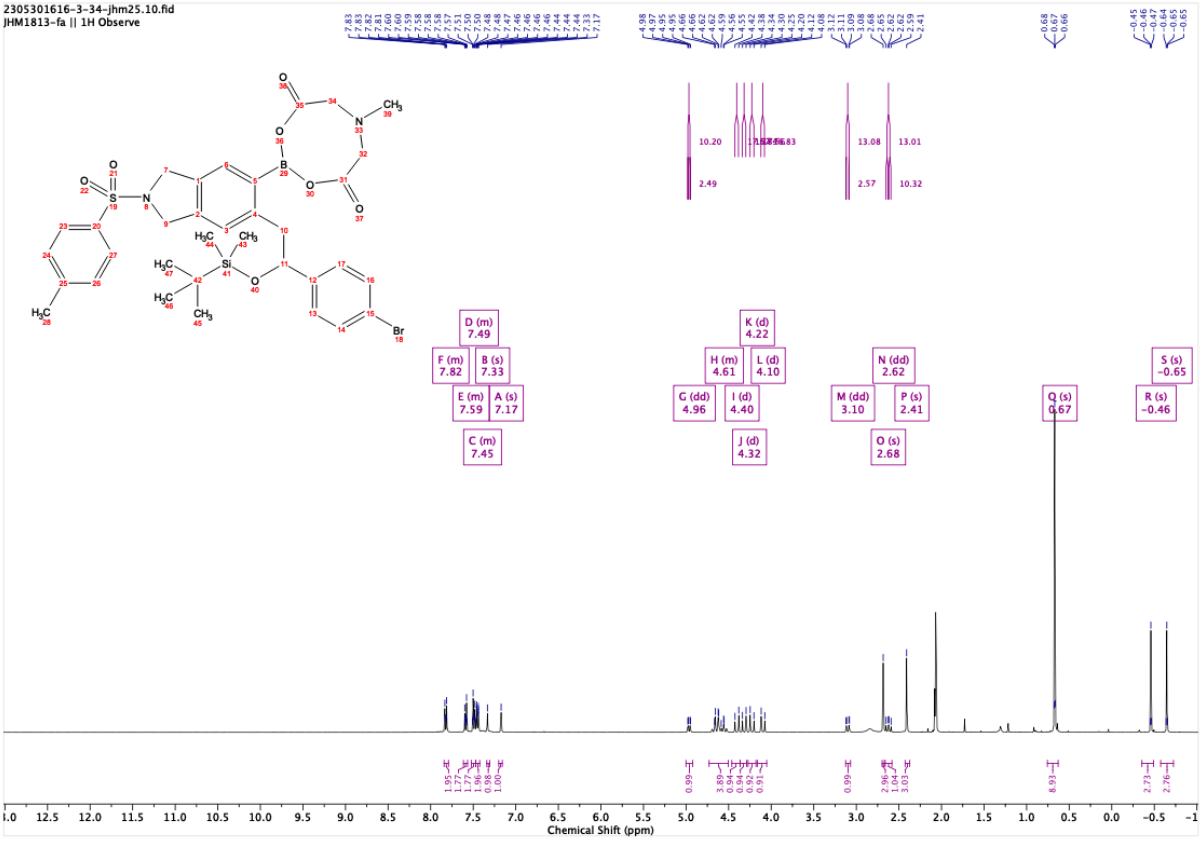

^{13}C DEPTQ

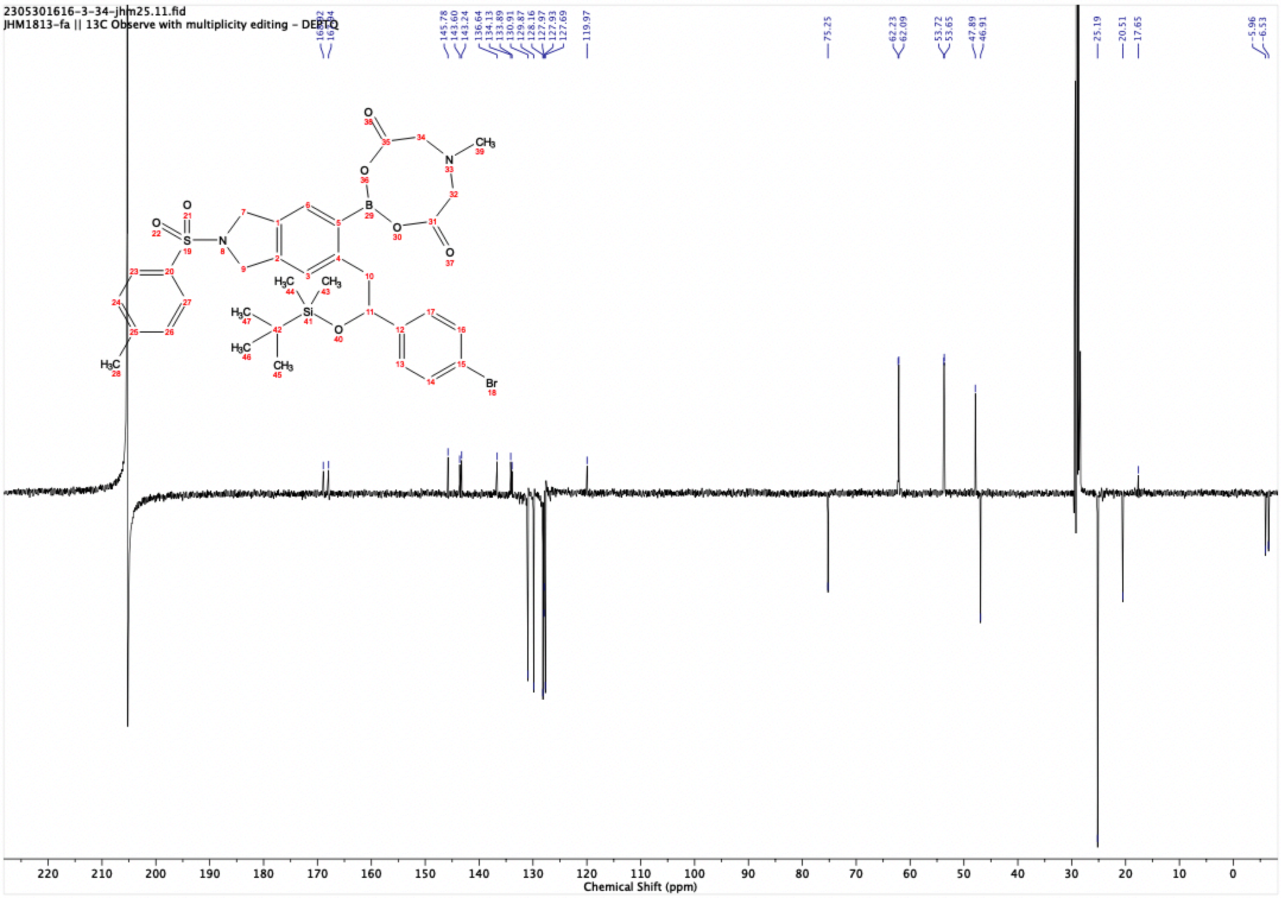

(S77)

^{1}H

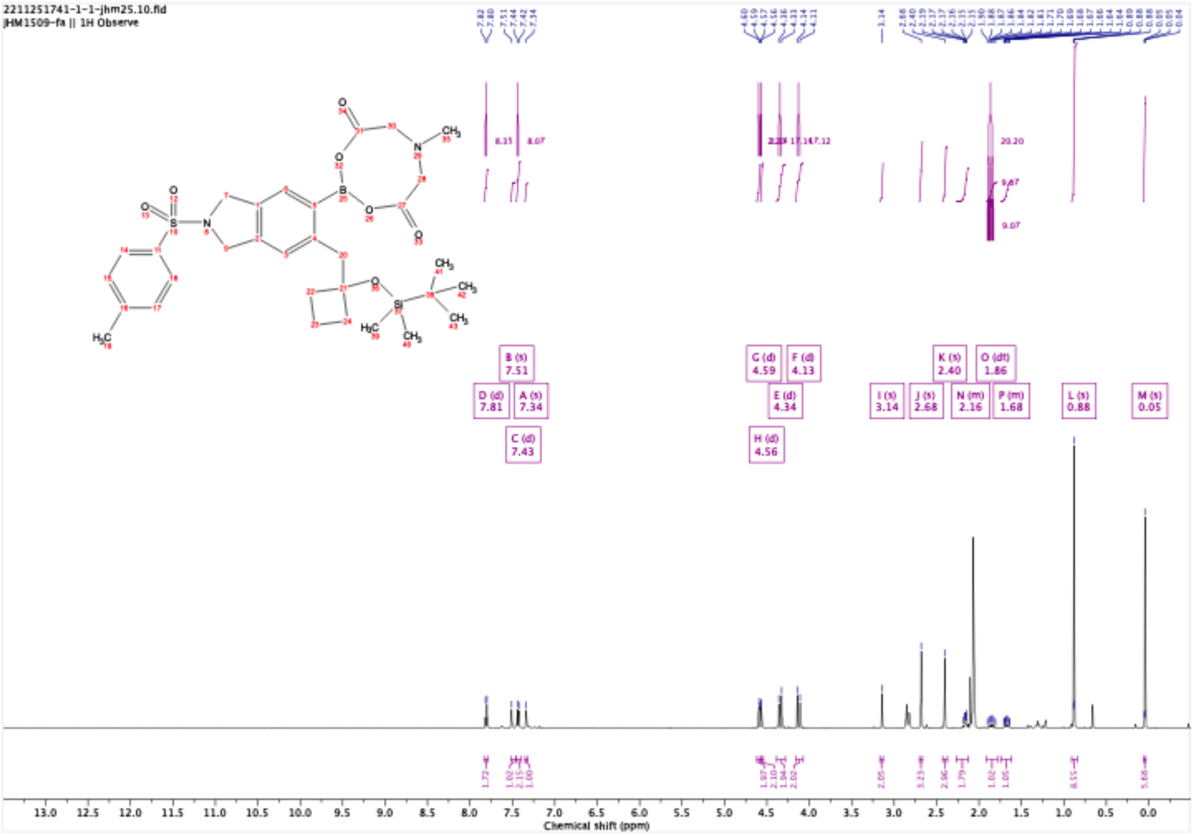

^{13}C DEPTQ

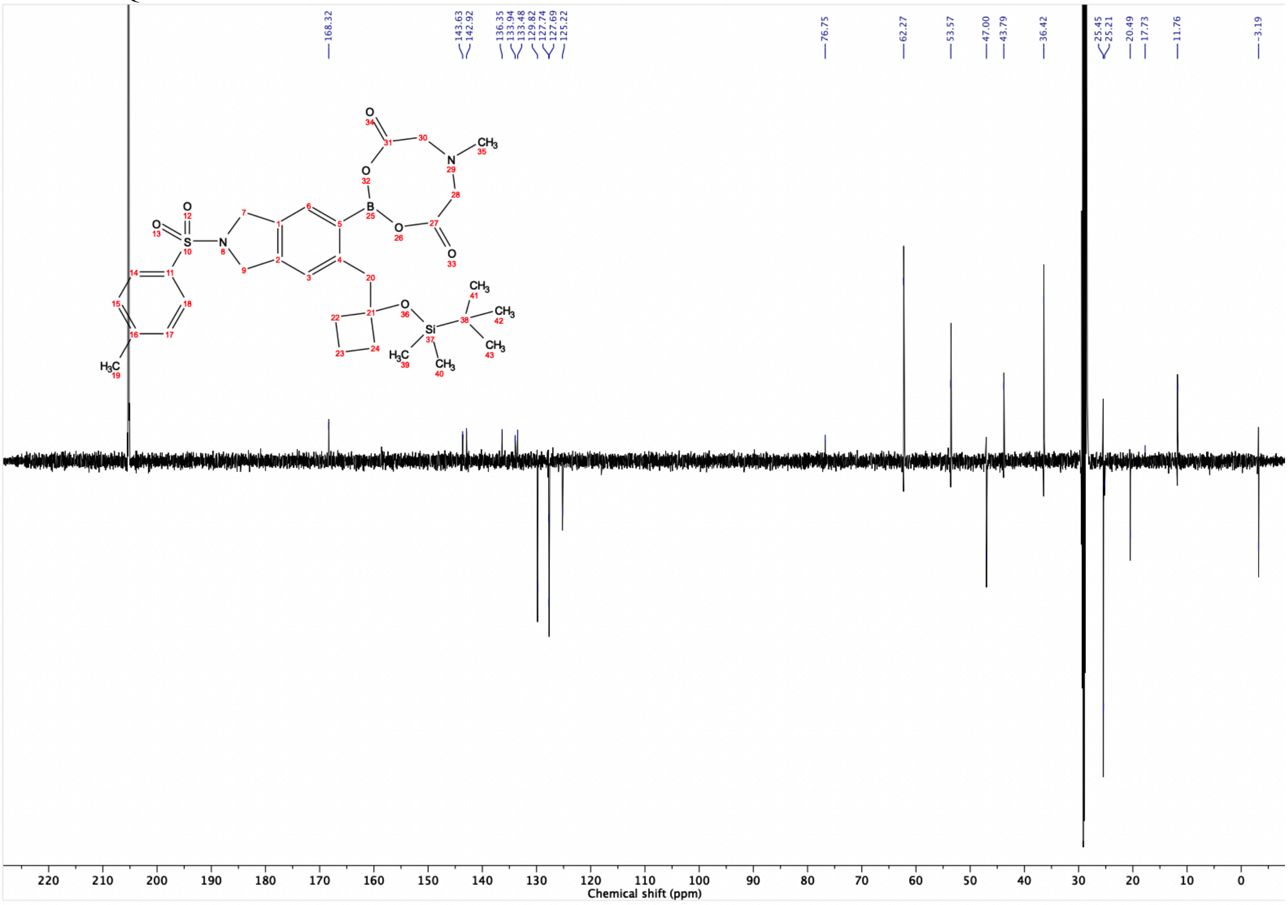

(S27)

^{1}H

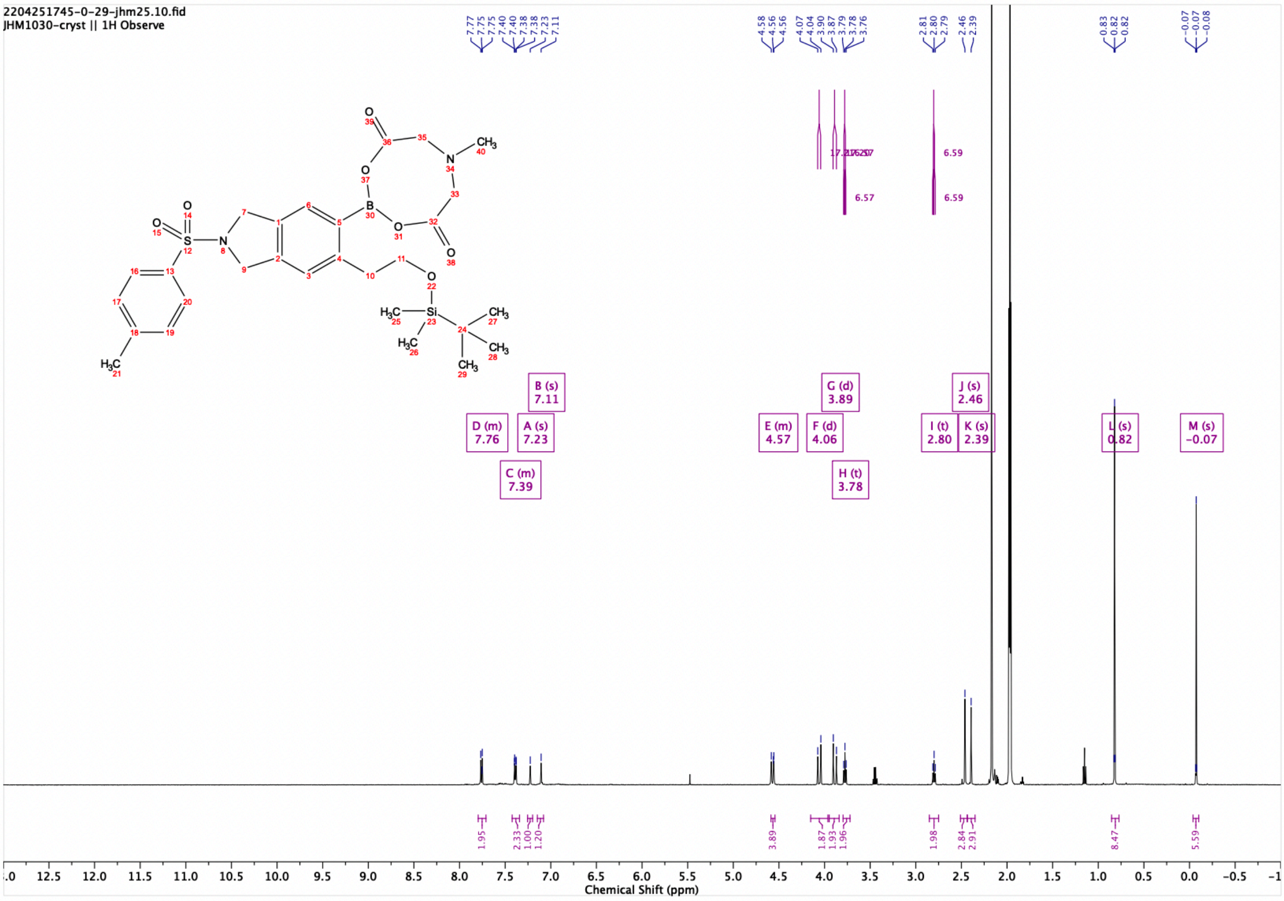

^{13}C DEPTQ

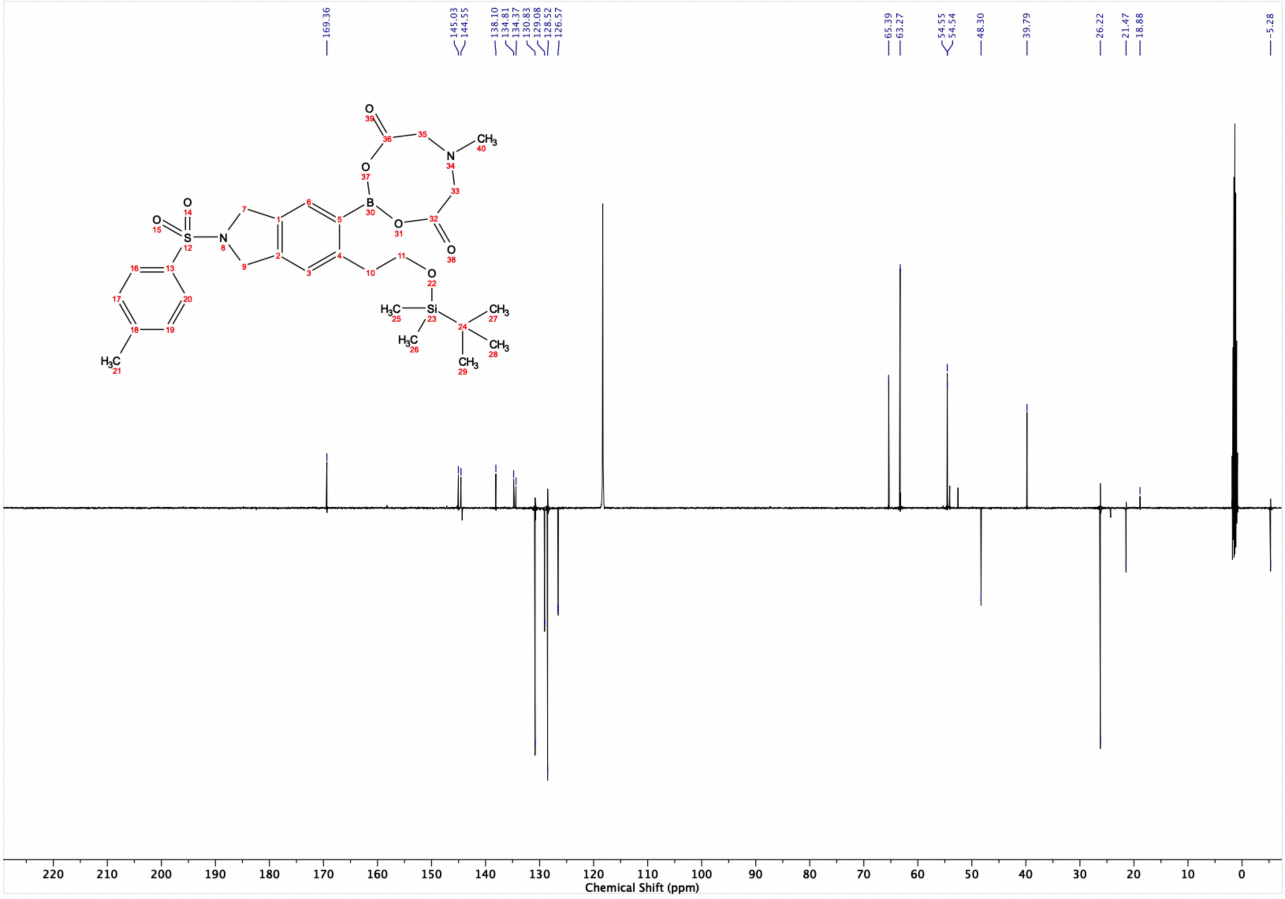

(S79)

^{1}H

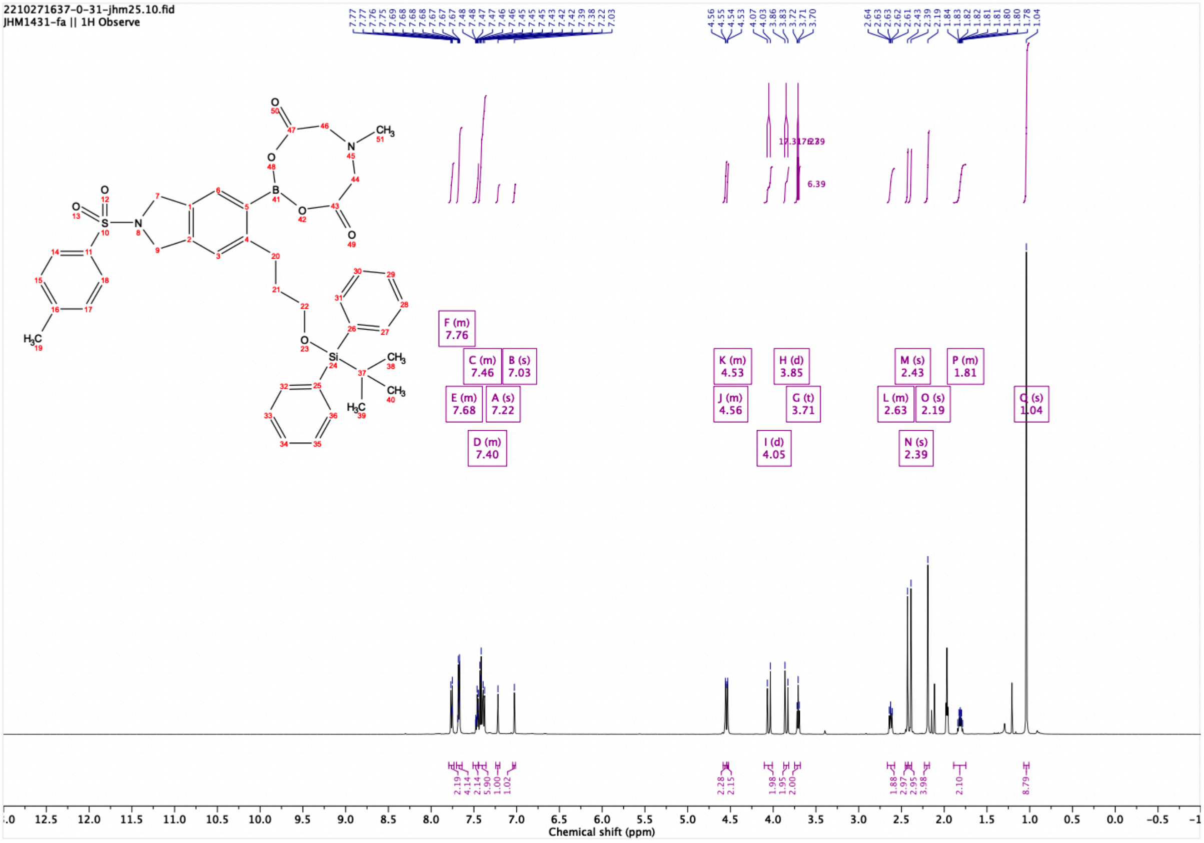

^{13}C DEPTQ

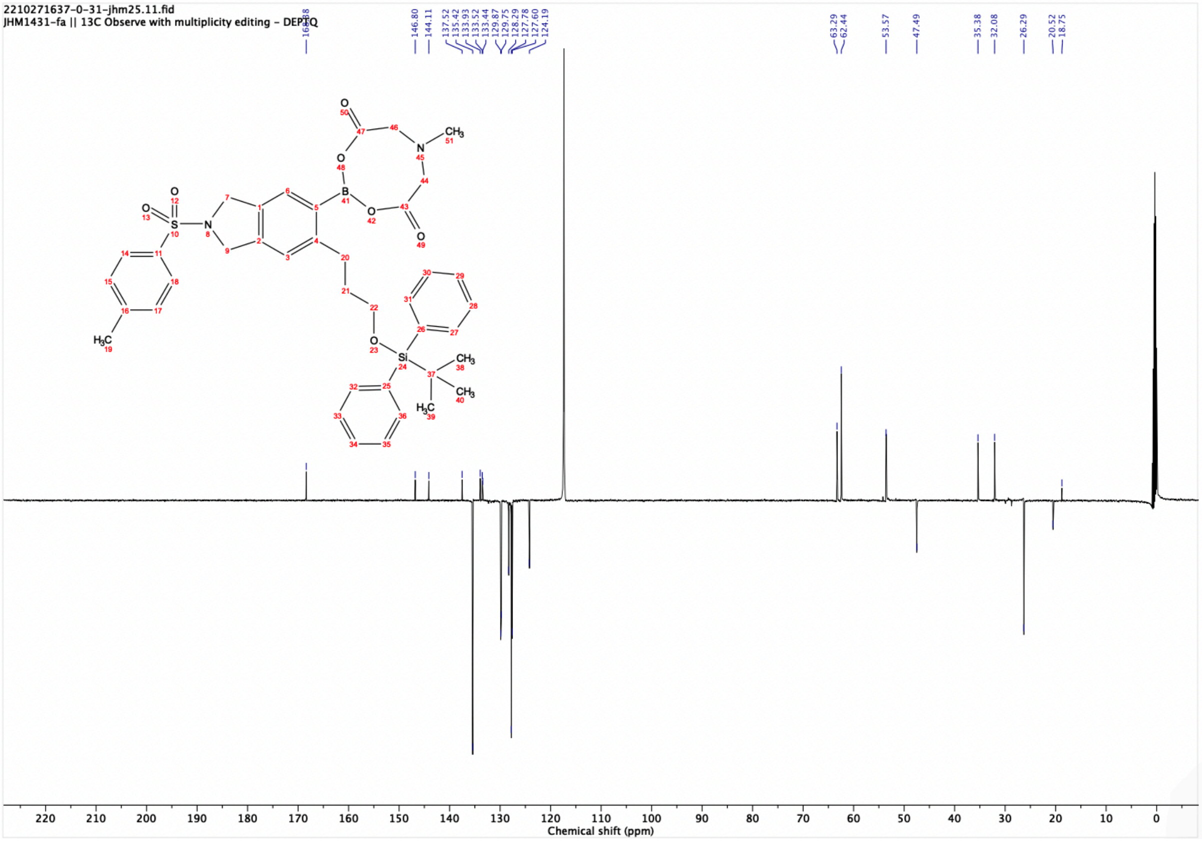

(S80)

^{1}H

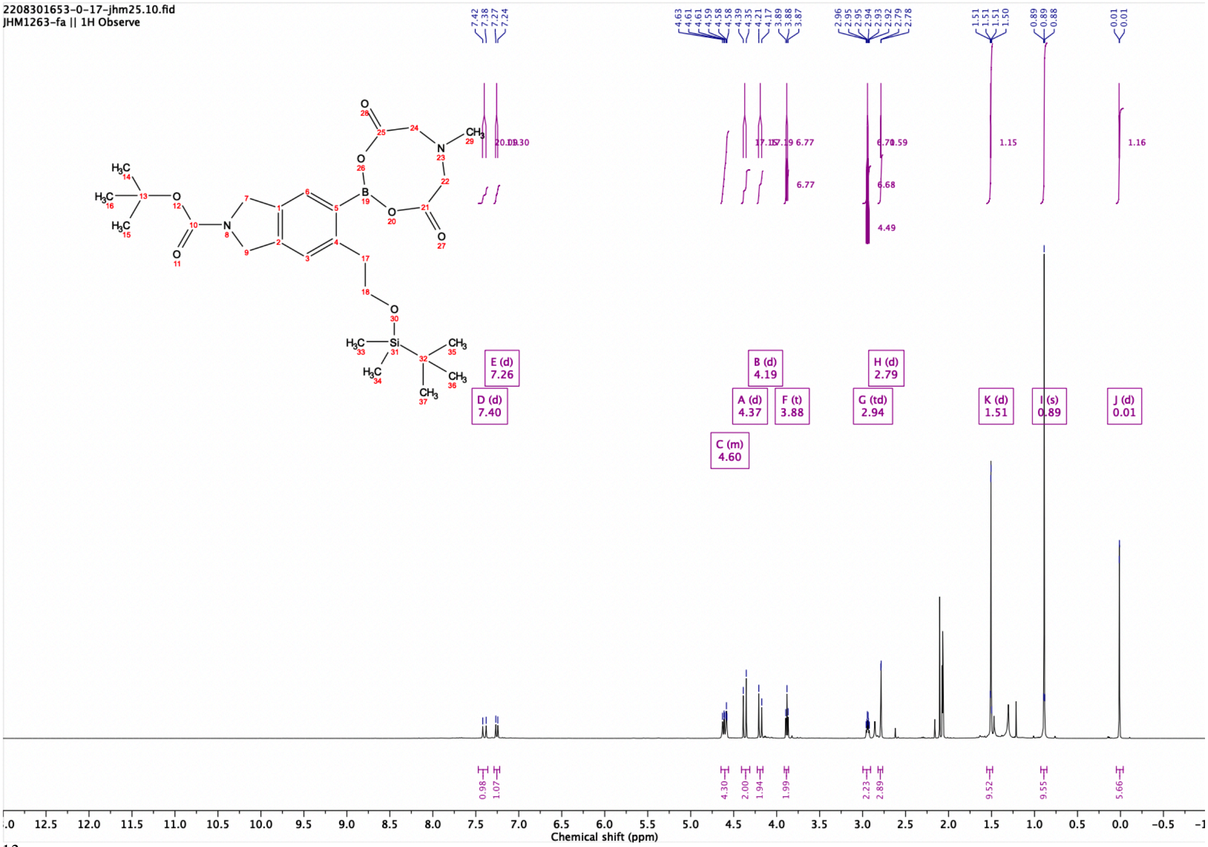

2208301653-0-17-jhm25.10.fid
JHM1263-fa || 1H Observe
E (d) 7.26
D (d) 7.40
B (d) 4.19
A (d) 4.37
F (t) 3.88
C (m) 4.60
H (d) 2.79
G (td) 2.94
K (d) 1.51
I (s) 0.89
J (d) 0.01
Chemical shift (ppm)

^{13}C

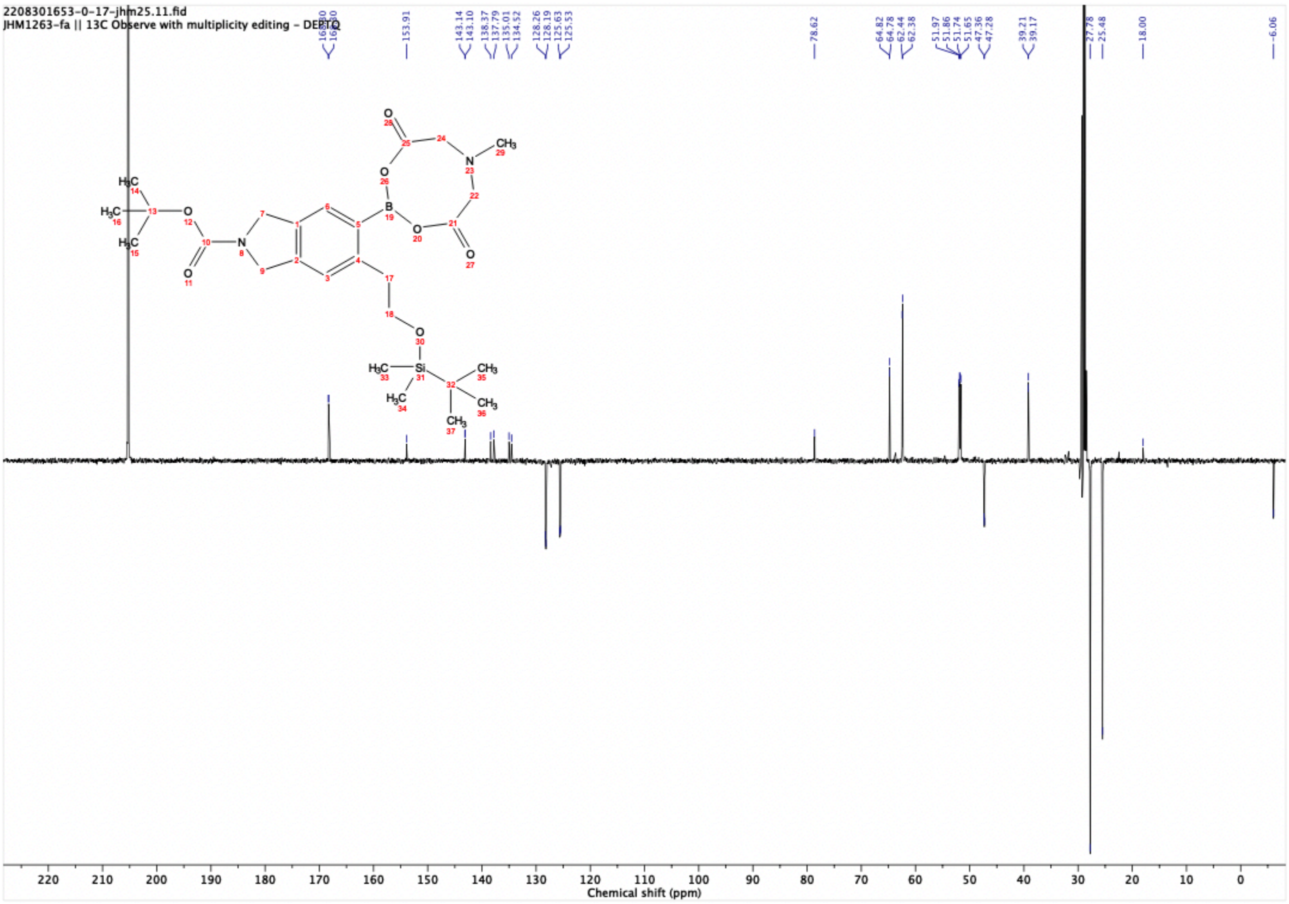

2208301653-0-17-jhm25.11.fid
JHM1263-fa || 13C Observe with multiplicity editing - DEPTQ
Chemical shift (ppm)

(S81)

^{1}H

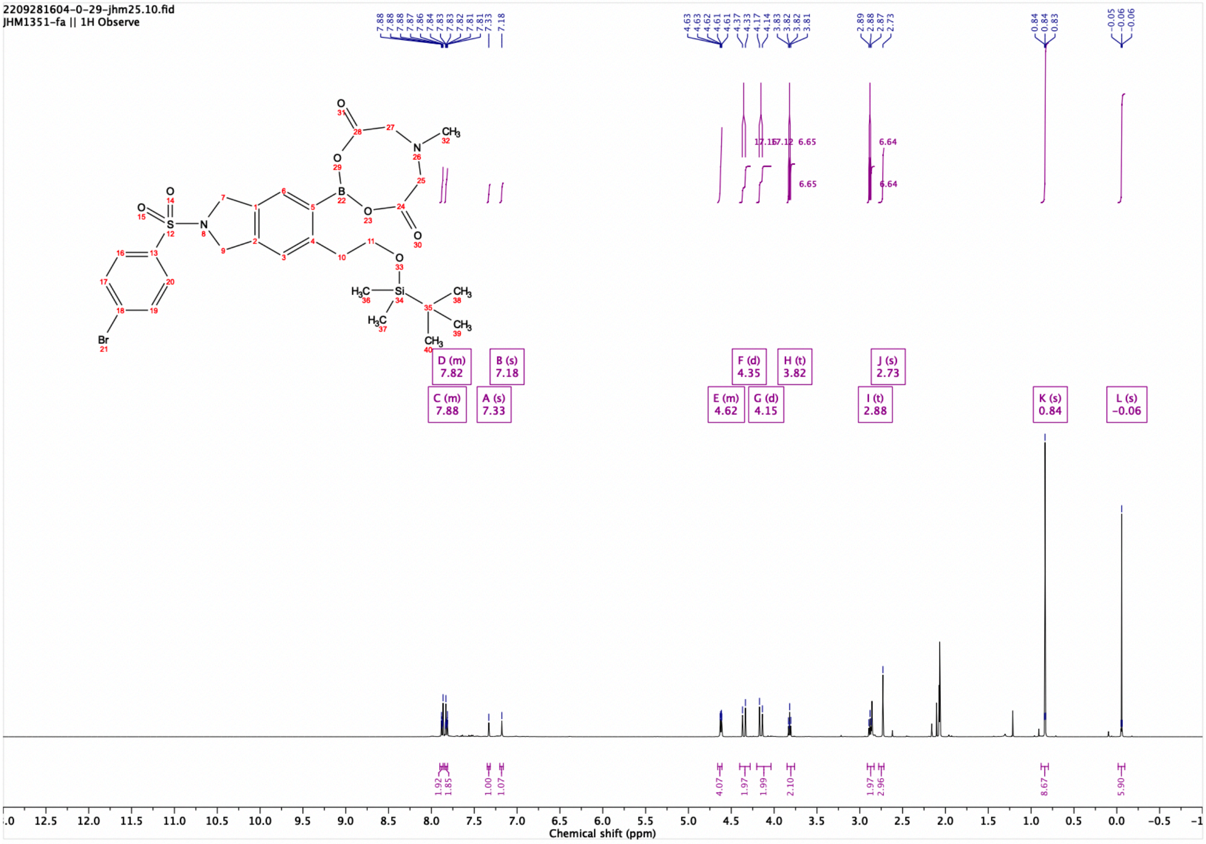

2209281604-0-29-jhm25.10.fid
JHM1351-fa || 1H Observe
Chemical shift (ppm)

^{13}C

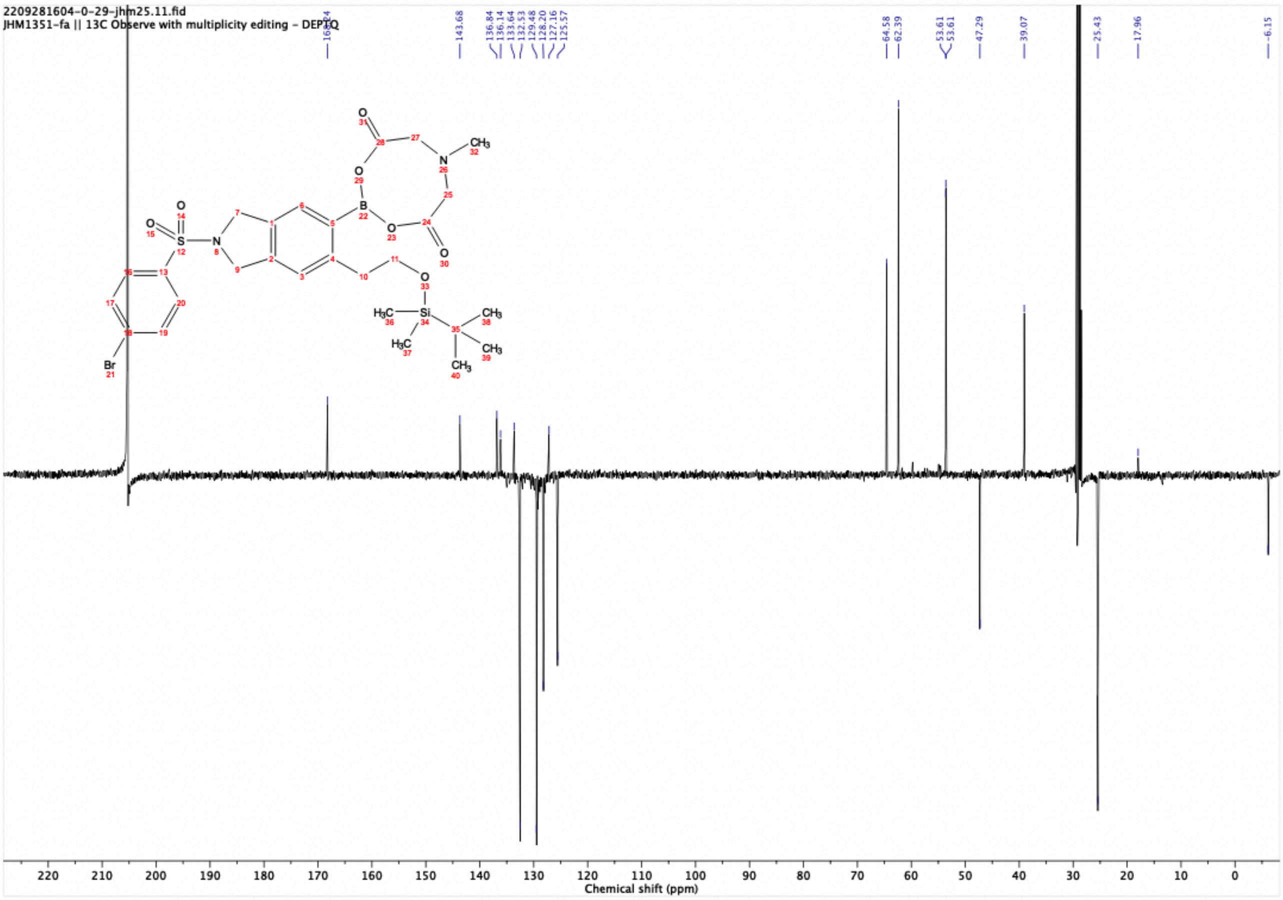

2209281604-0-29-jhm25.11.fid
JHM1351-fa || 13C Observe with multiplicity editing - DEPTQ
Chemical shift (ppm)

(S82)

^{1}H

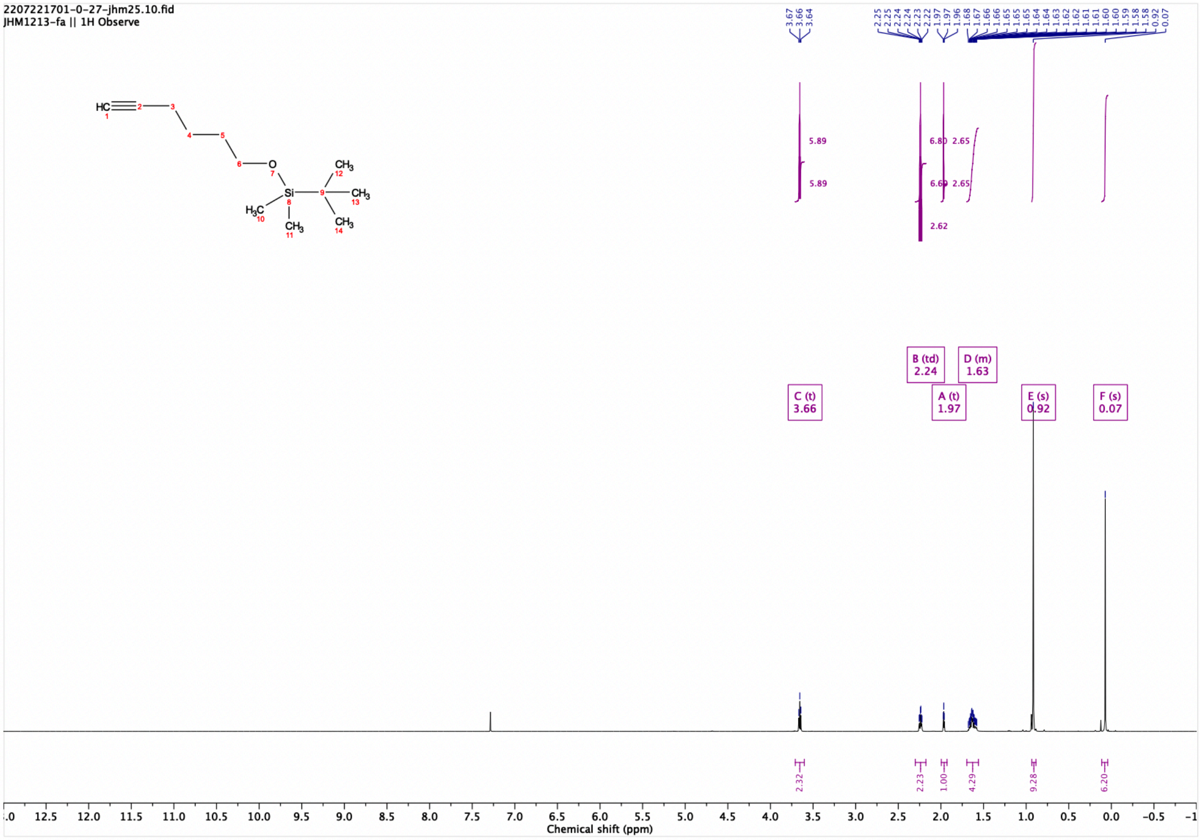

^{13}C DEPTQ

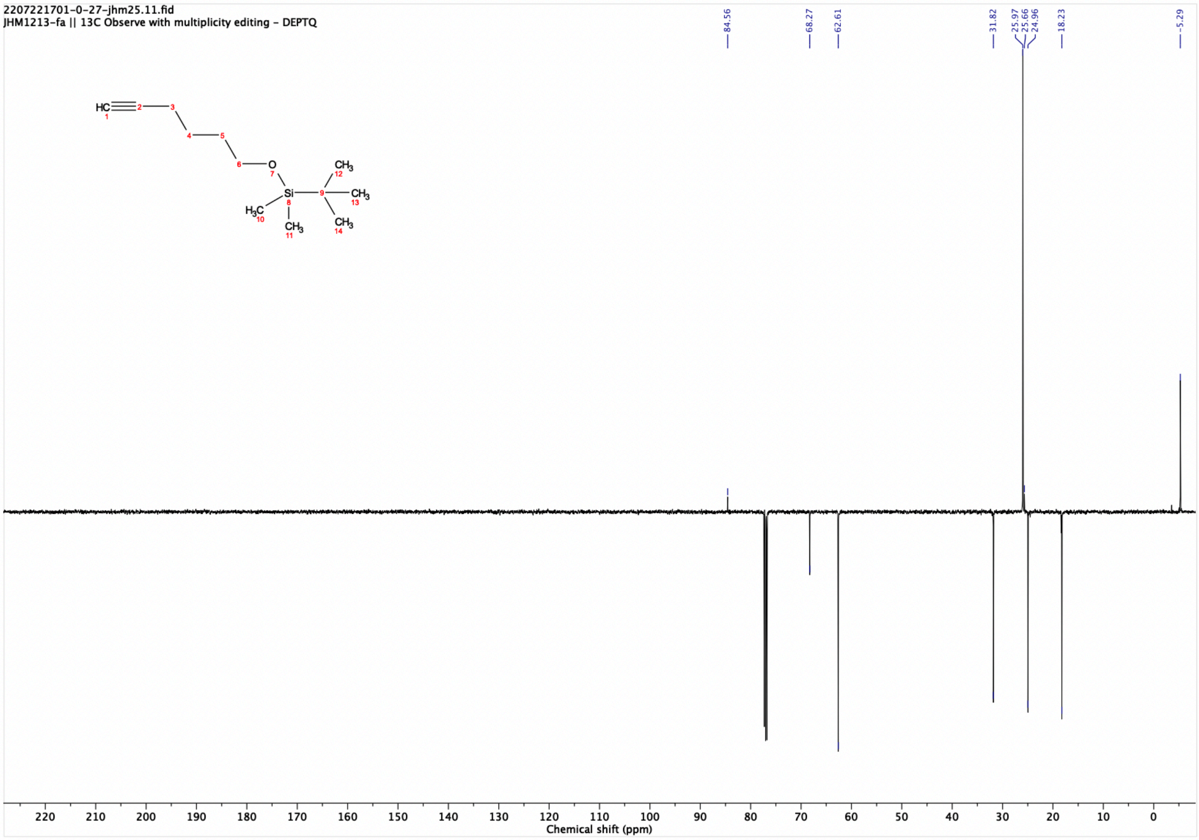

(S83)

^{1}H

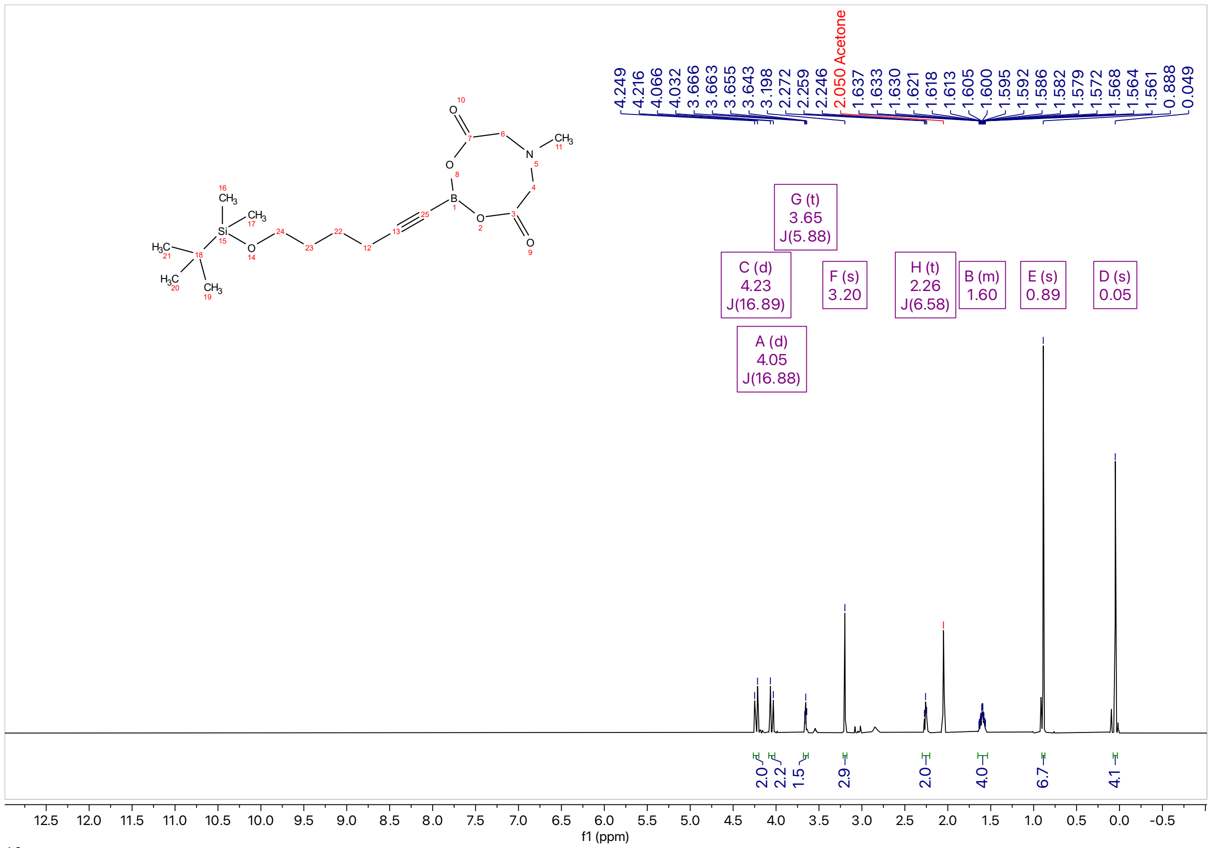
4.249
4.216
4.066
4.032
3.666
3.663
3.655
3.643
3.198
2.272
2.259
2.246
2.050 Acetone
1.637
1.633
1.630
1.621
1.618
1.613
1.605
1.600
1.595
1.592
1.586
1.582
1.579
1.572
1.568
1.564
1.561
0.888
0.049
G (t) 3.65 J(5.88)
C (d) 4.23 J(16.89)
F (s) 3.20
H (t) 2.26 J(6.58)
B (m) 1.60
E (s) 0.89
D (s) 0.05
A (d) 4.05 J(16.88)
2.0
2.2
1.5
2.9
2.0
4.0
6.7
4.1
f1 (ppm)

^{13}C

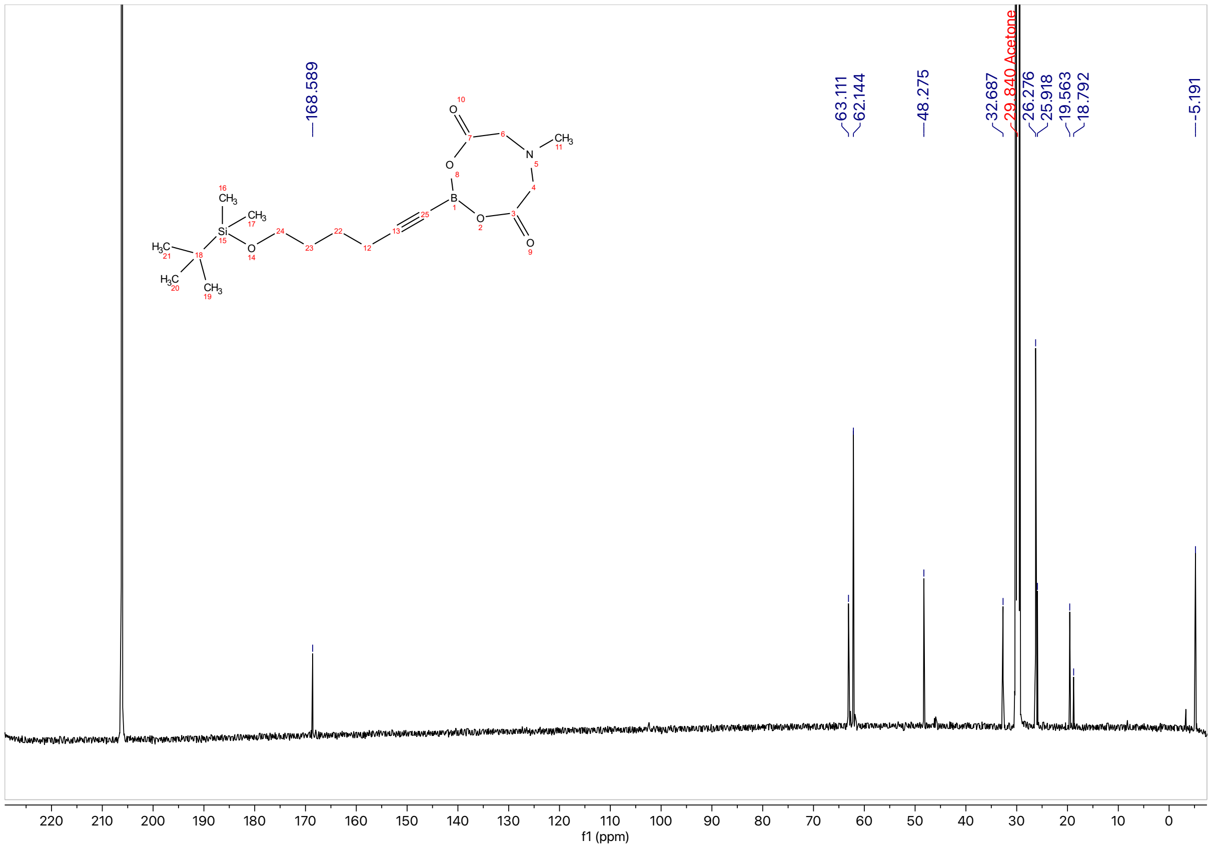
168.589
63.111
62.144
48.275
32.687
29.840 Acetone
26.276
25.918
19.563
18.792
-5.191
f1 (ppm)

(S84)

^{1}H

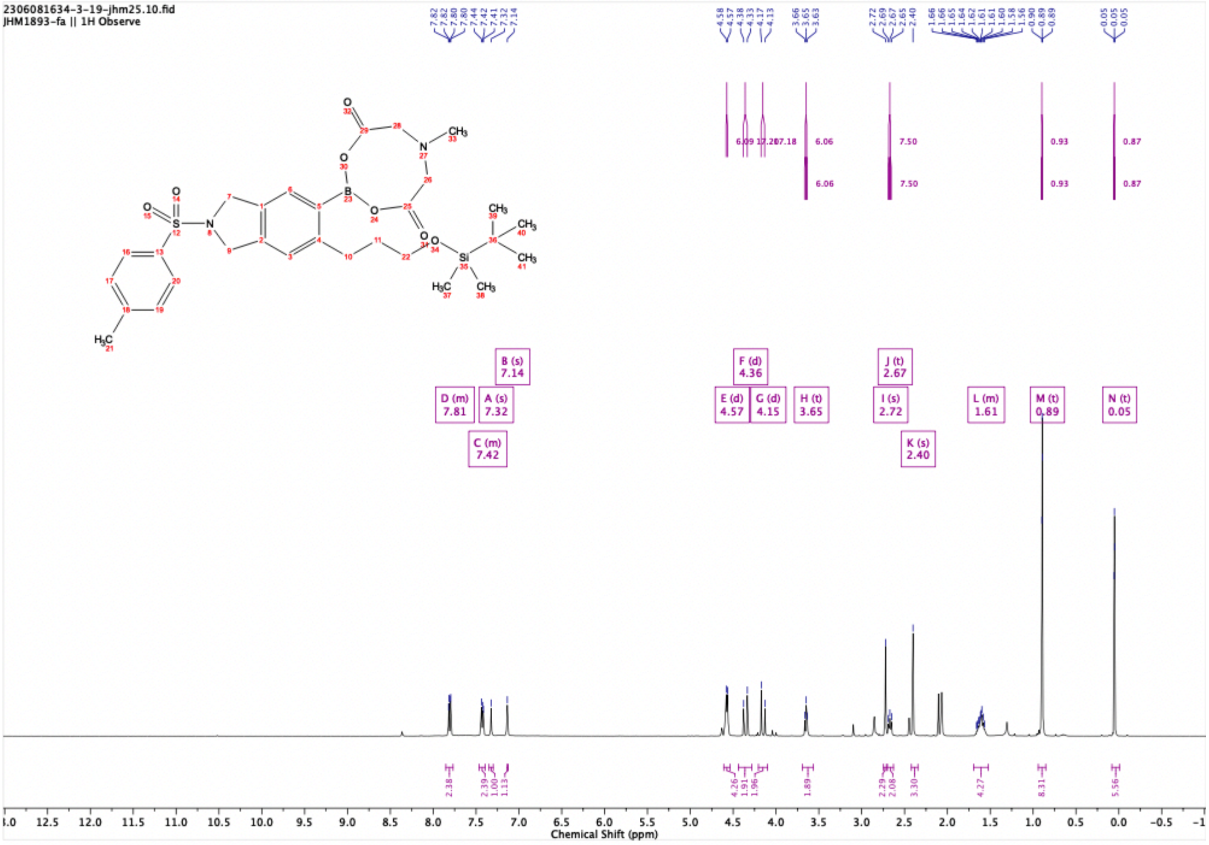

^{13}C DEPTQ

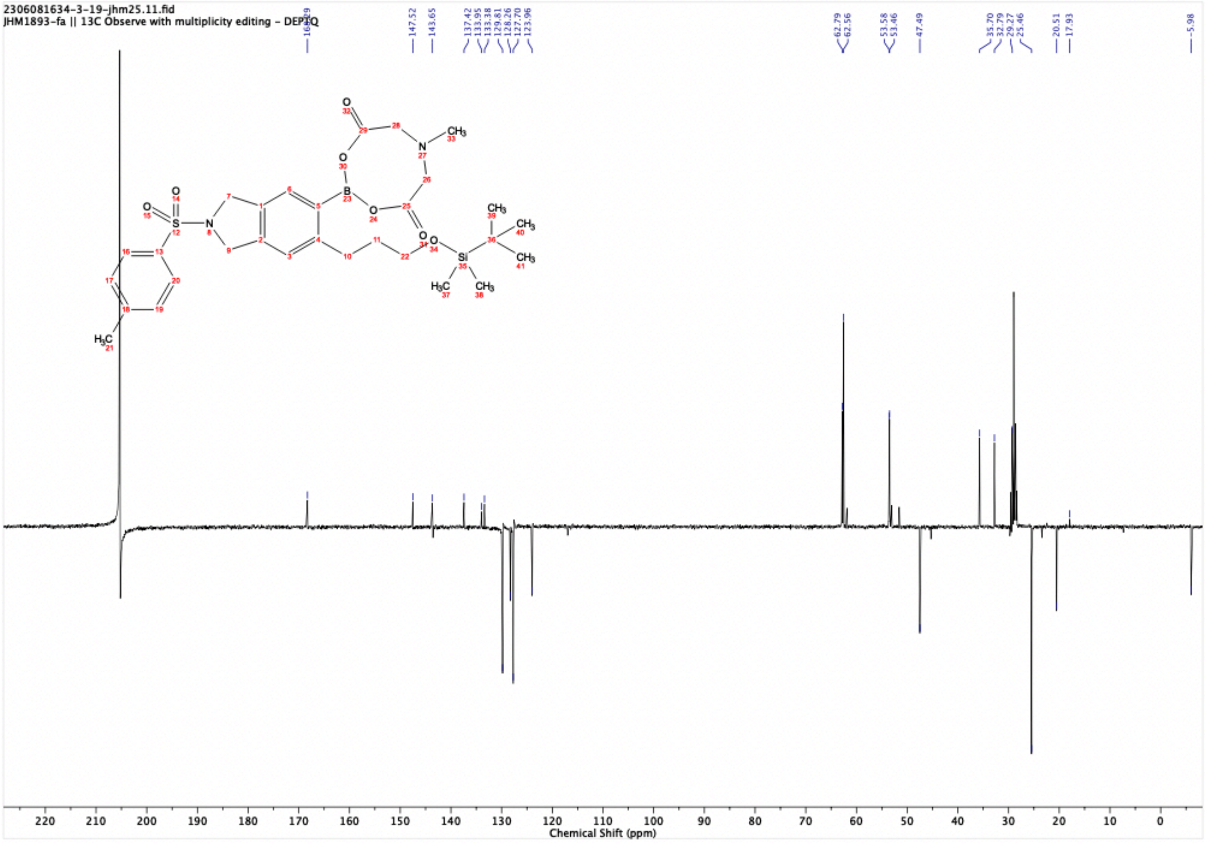

(1)

^{1}H

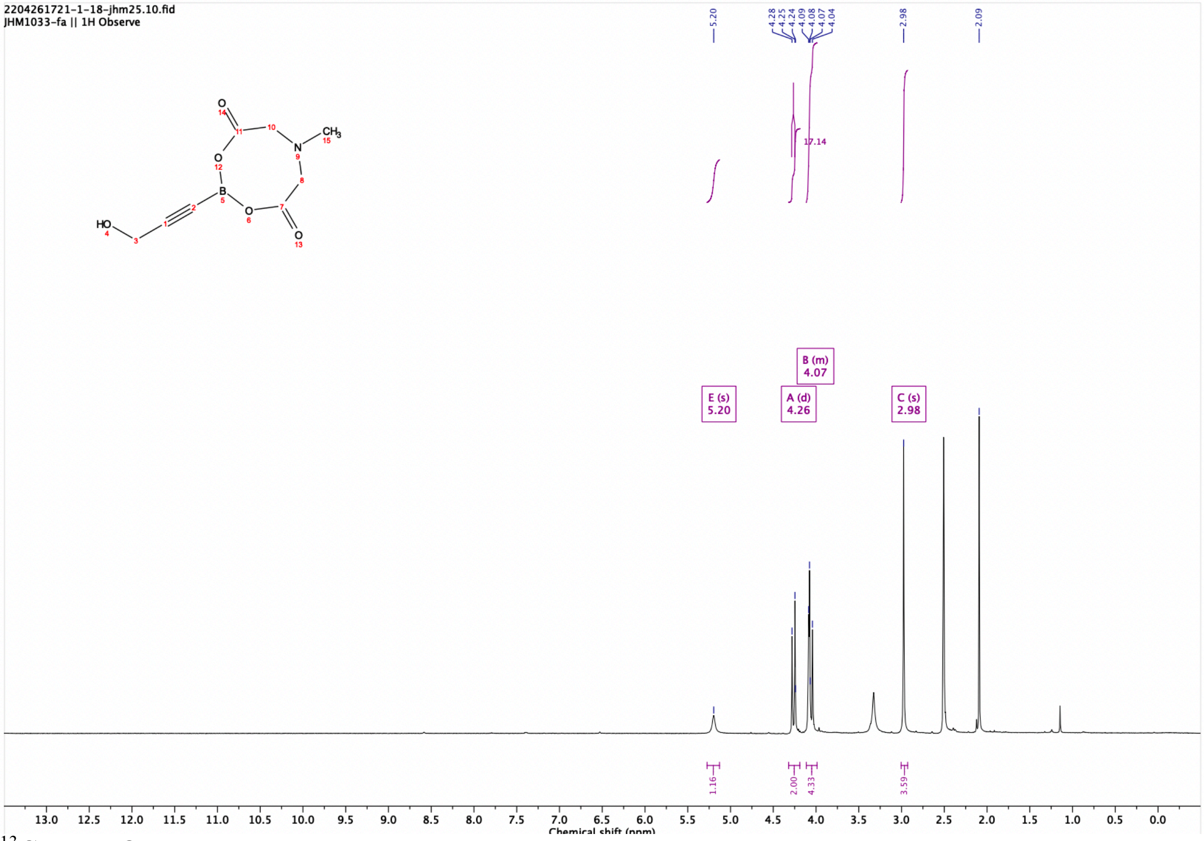

^{13}C DEPTQ

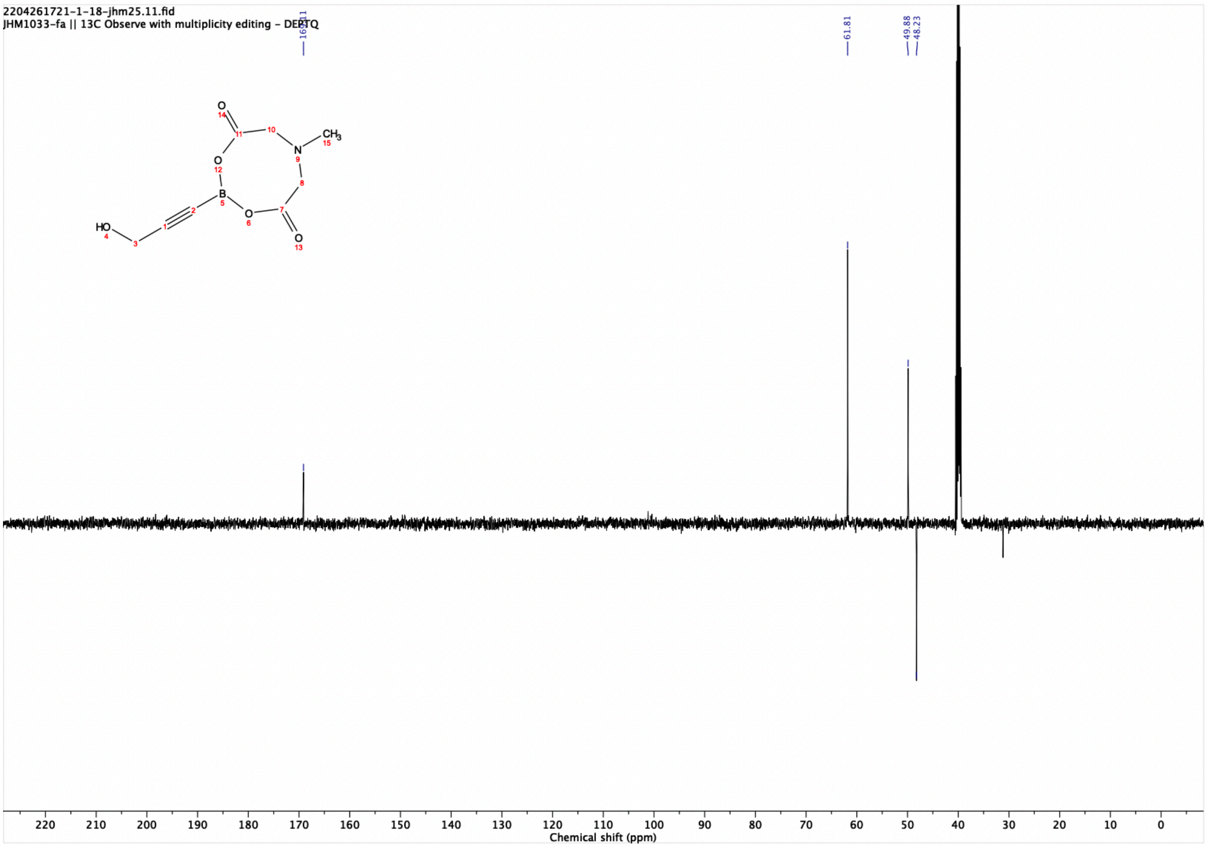

(4)

^{1}H

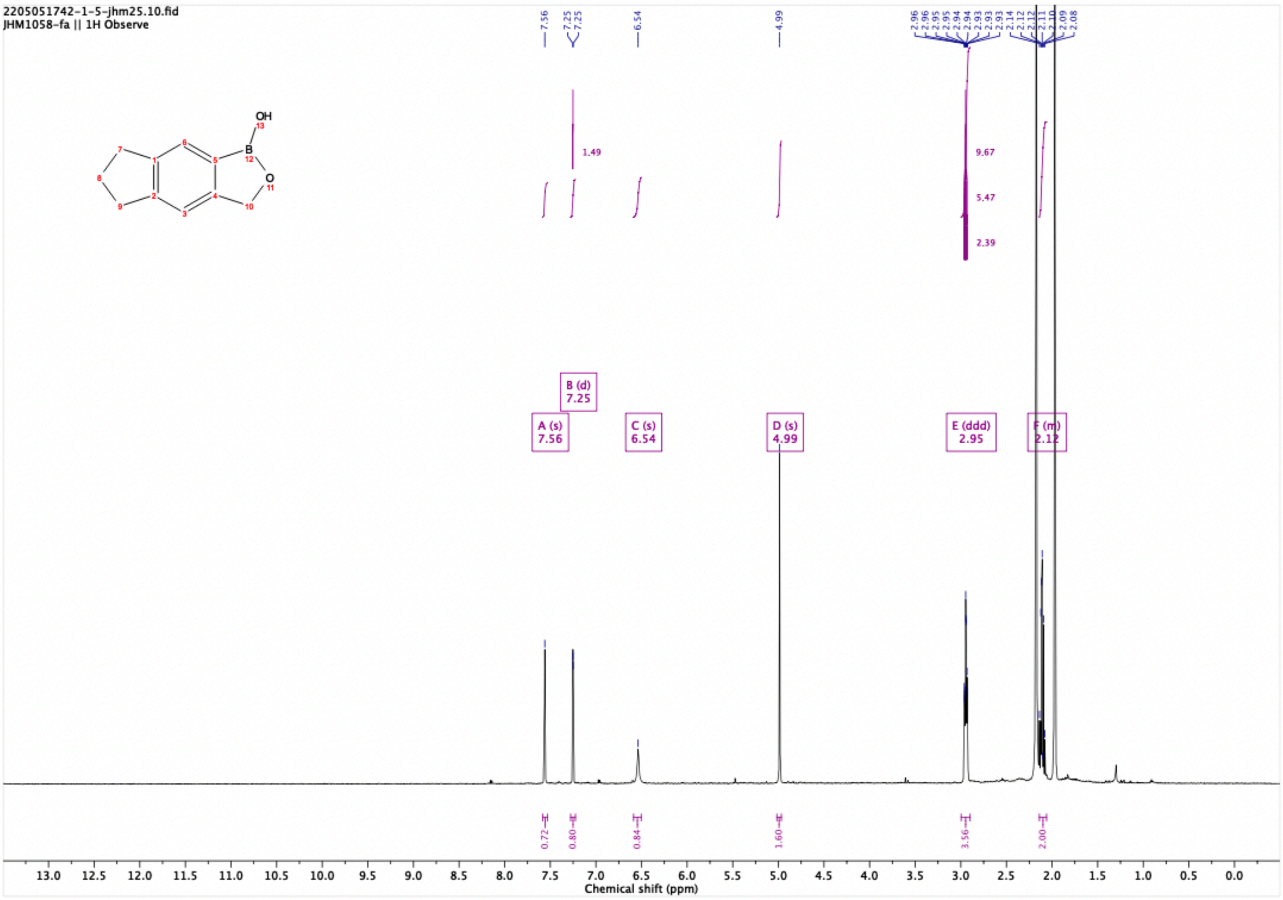

^{13}C DEPTQ

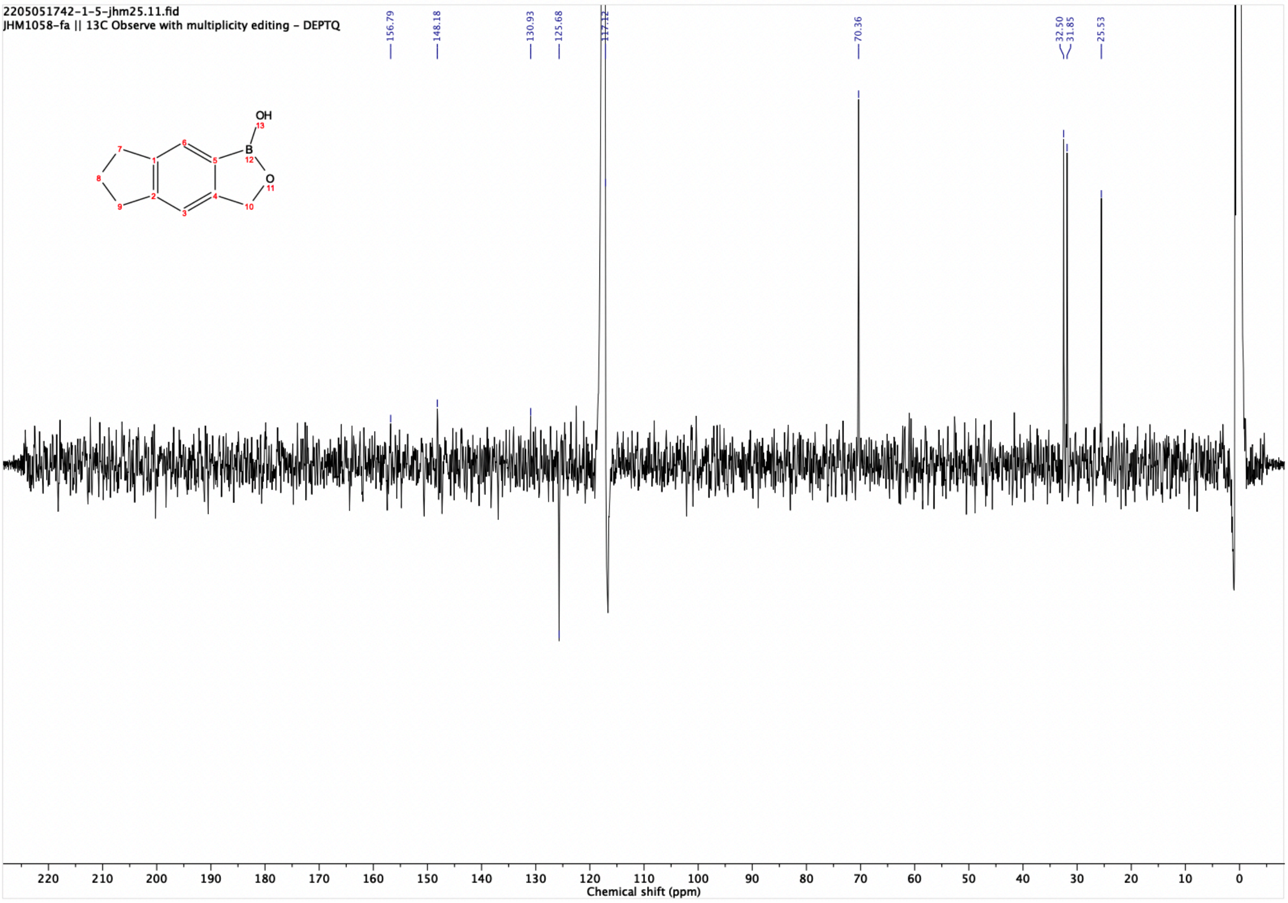

(5)

^{1}H

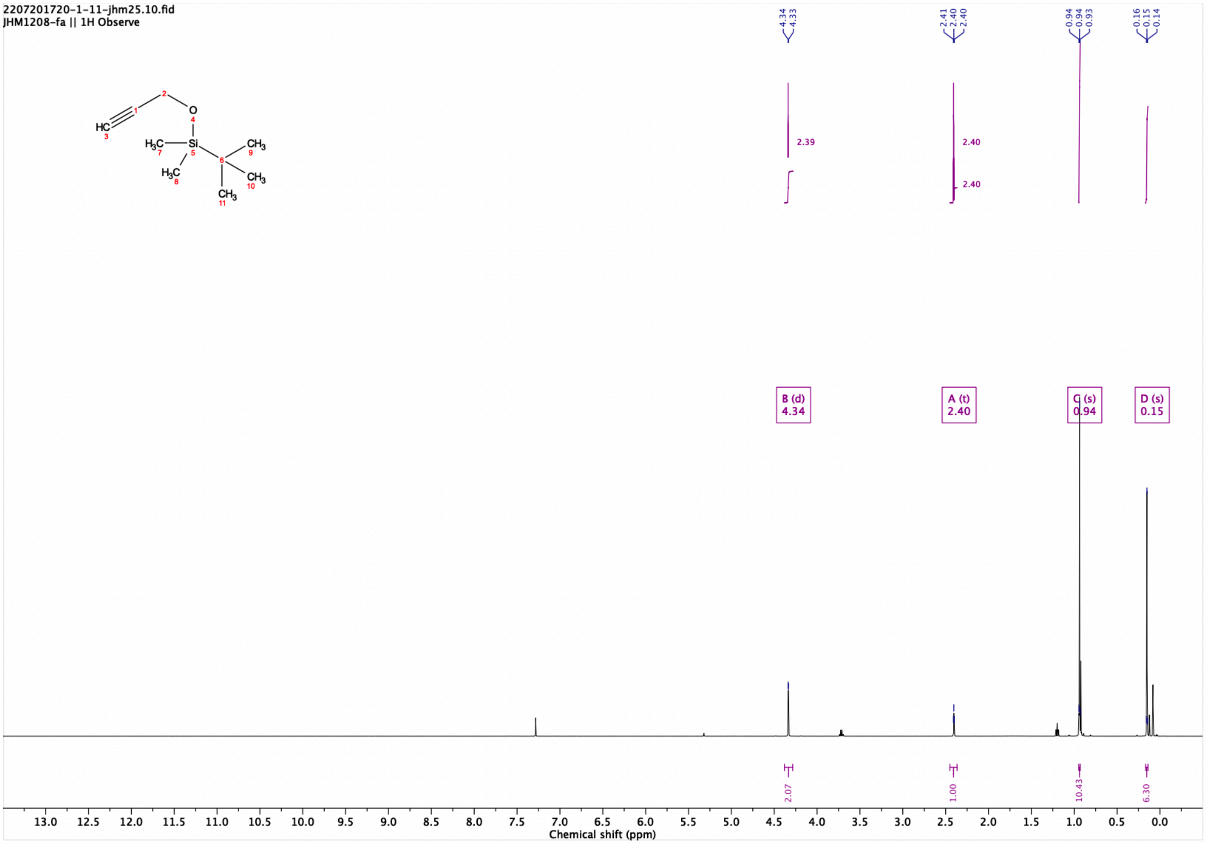

^{13}C DEPTQ

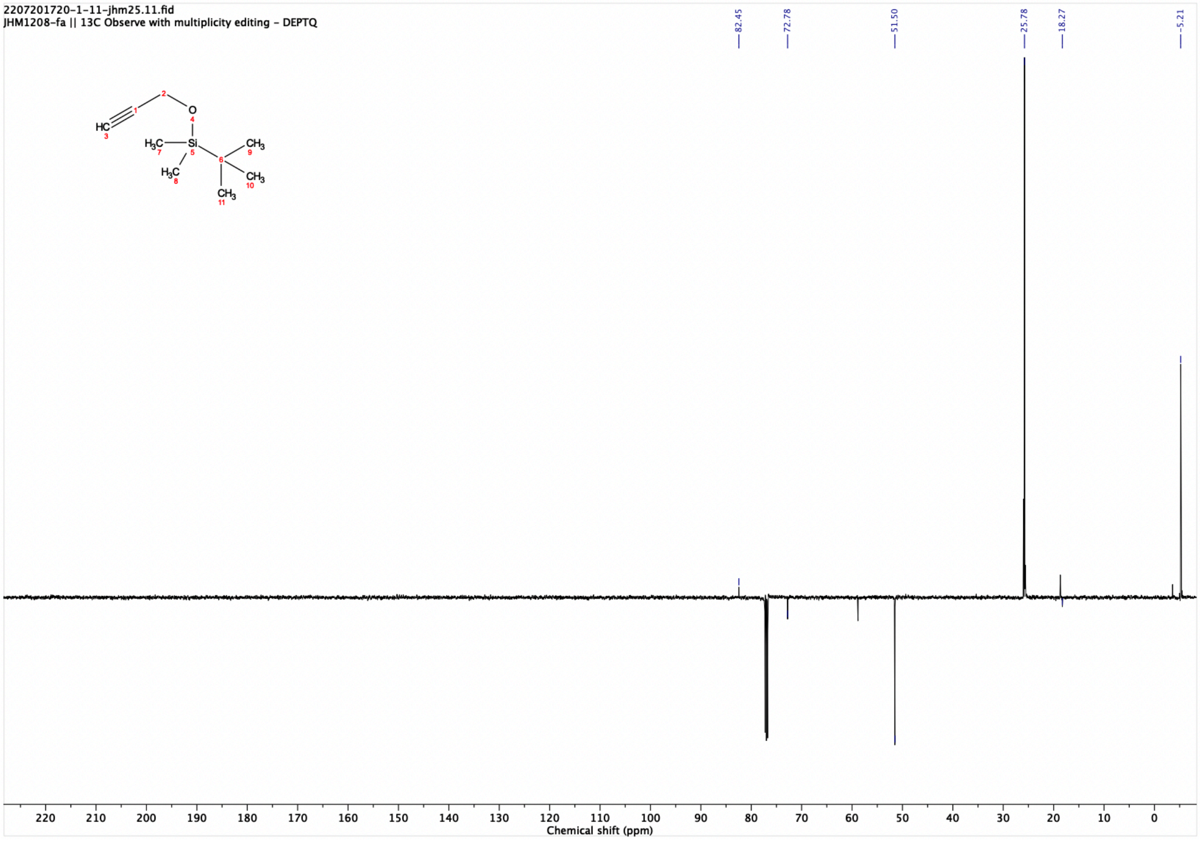

(8)

^{1}H

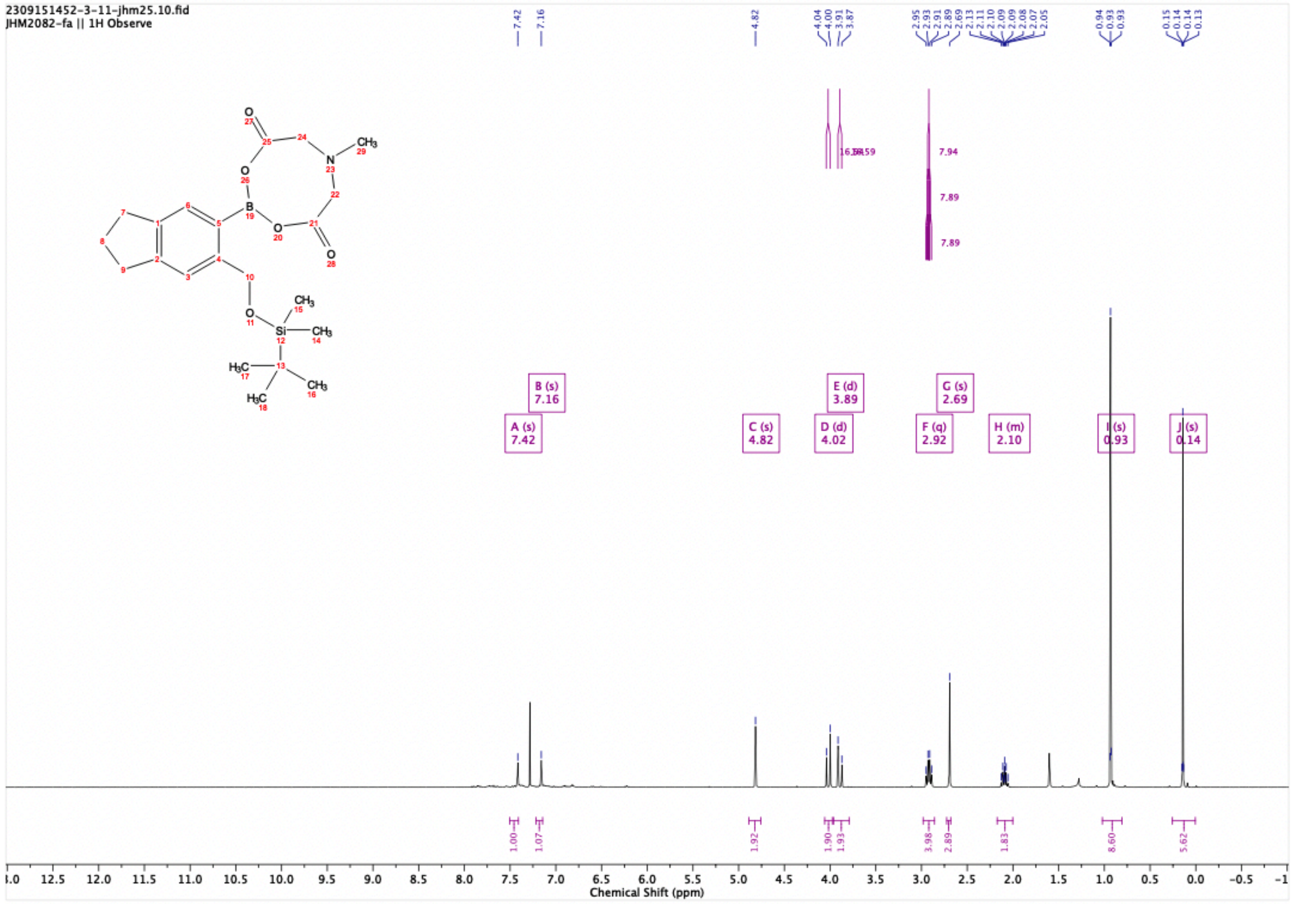

^{13}C DEPTQ

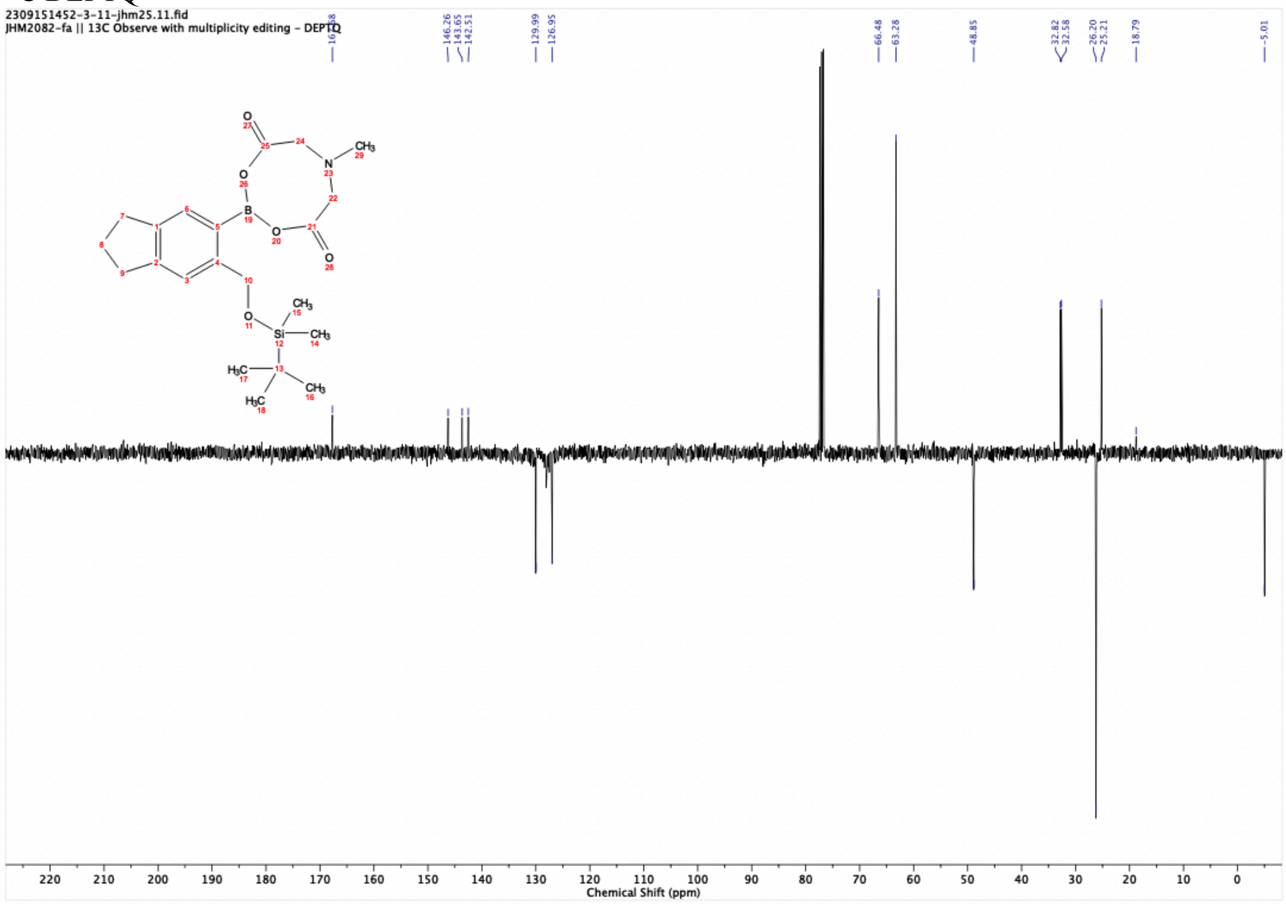

(10)

^{1}H

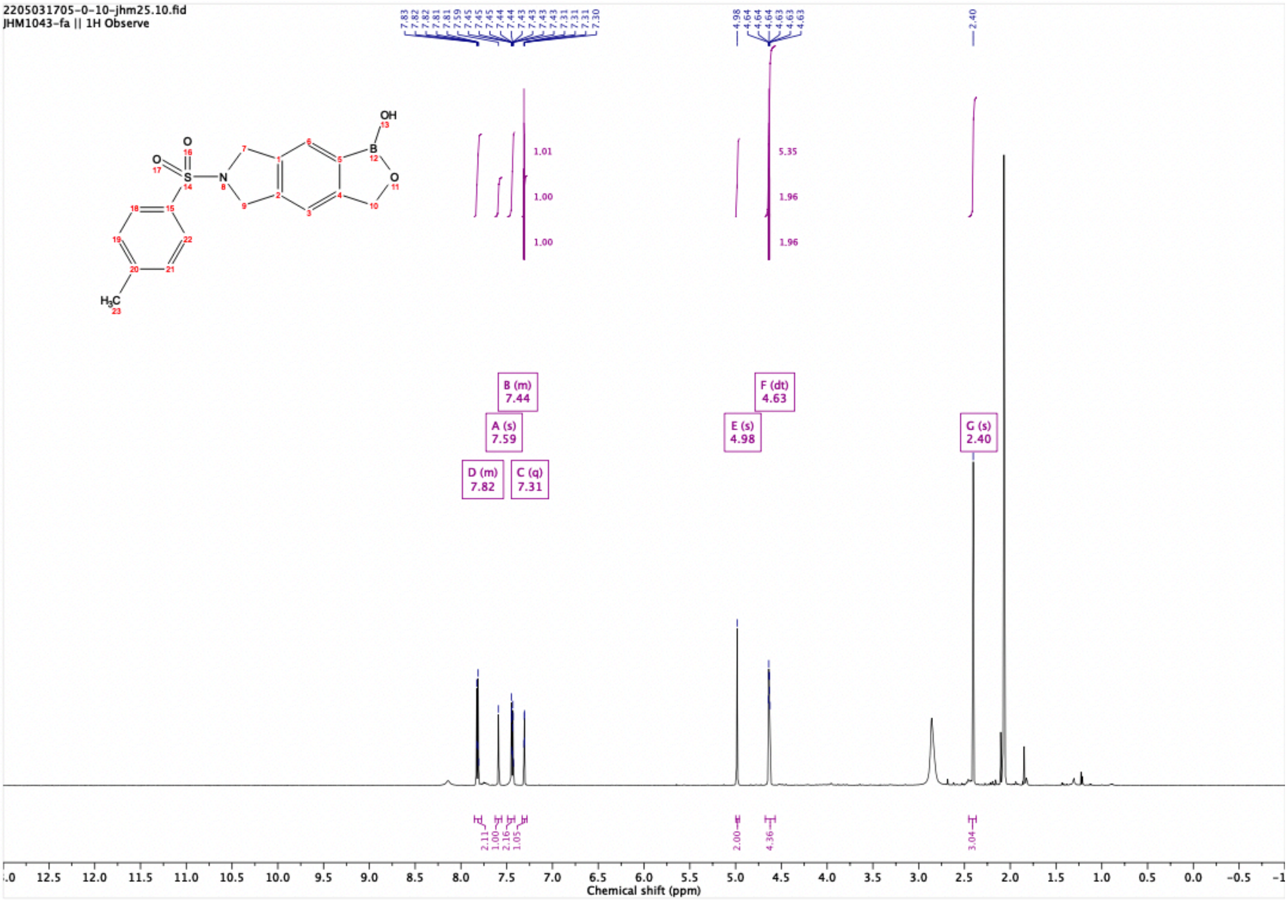

^{13}C DEPTQ

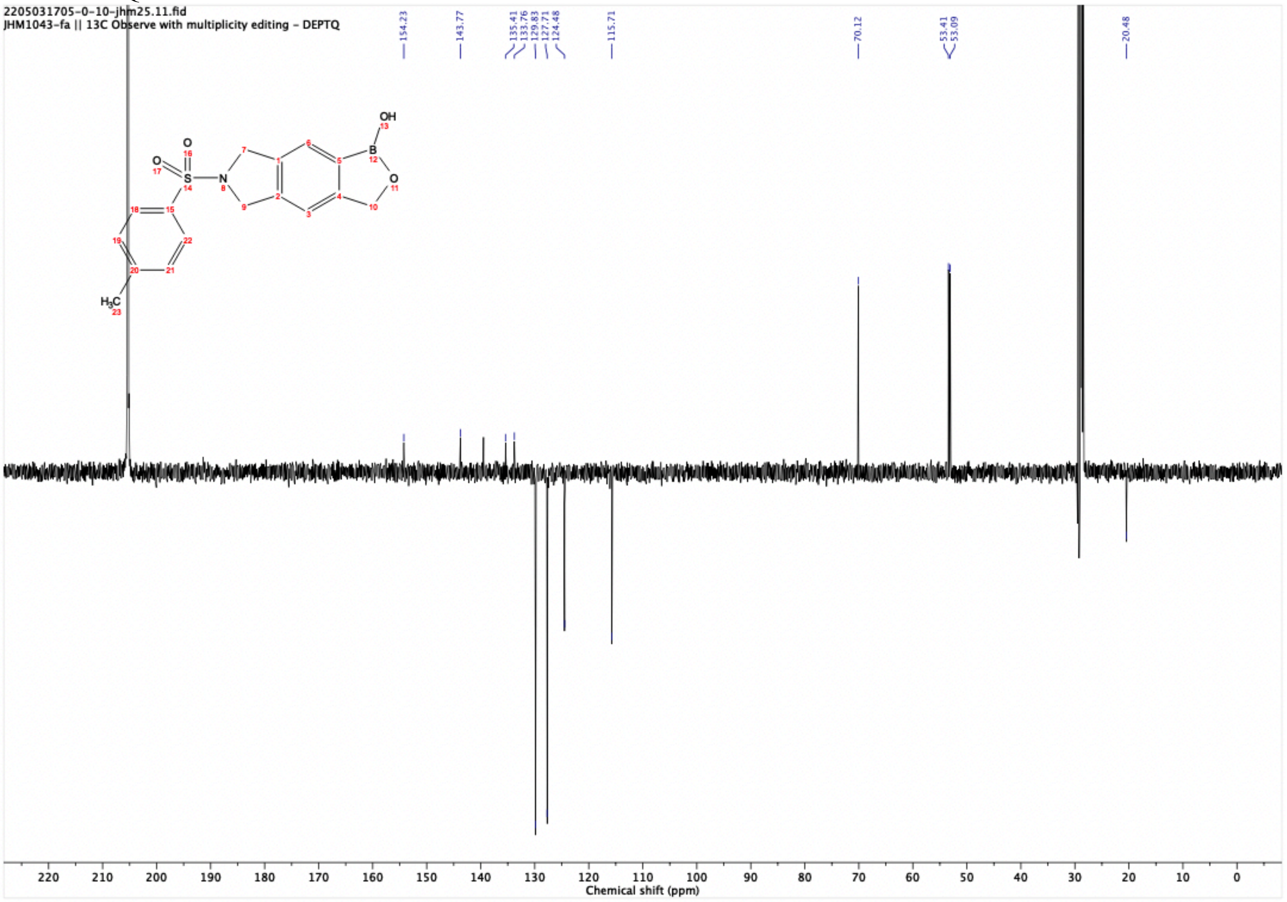

(11)

^{1}H

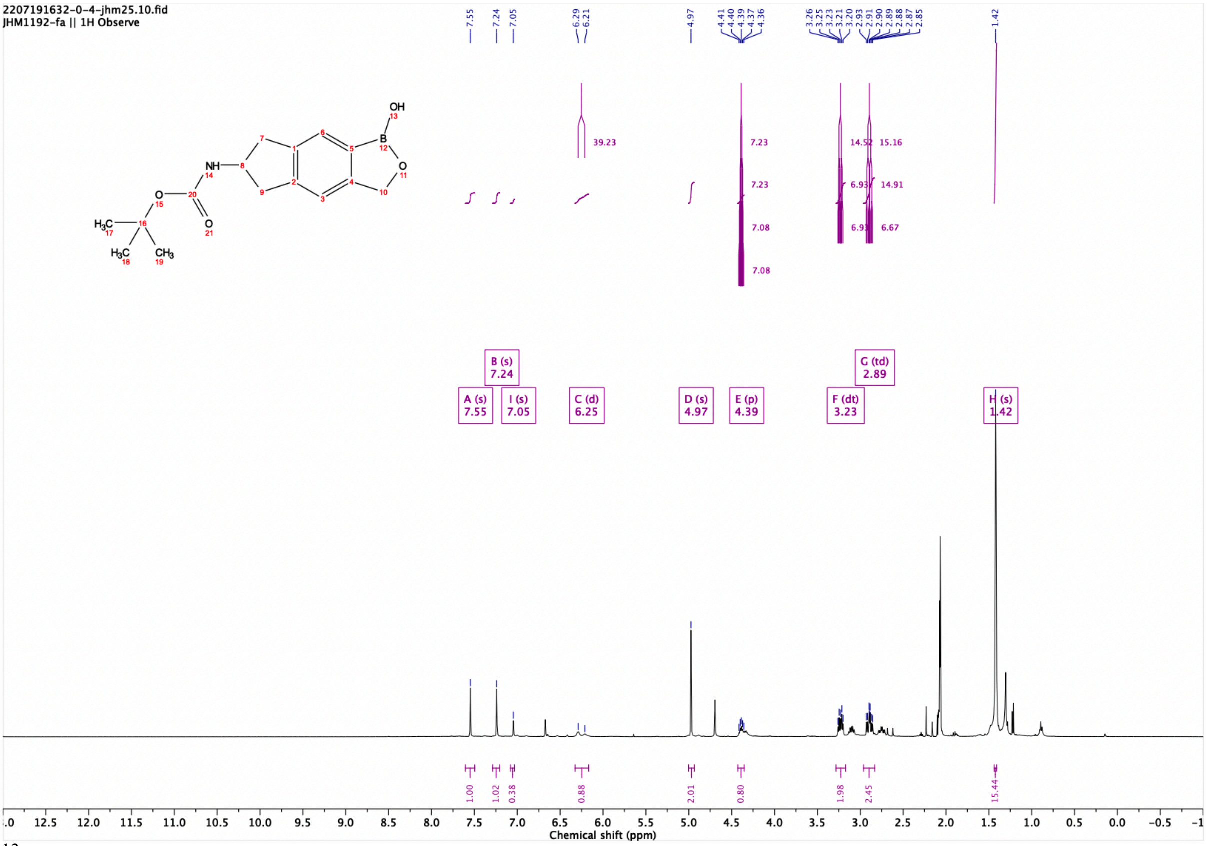

^{13}C DEPTQ

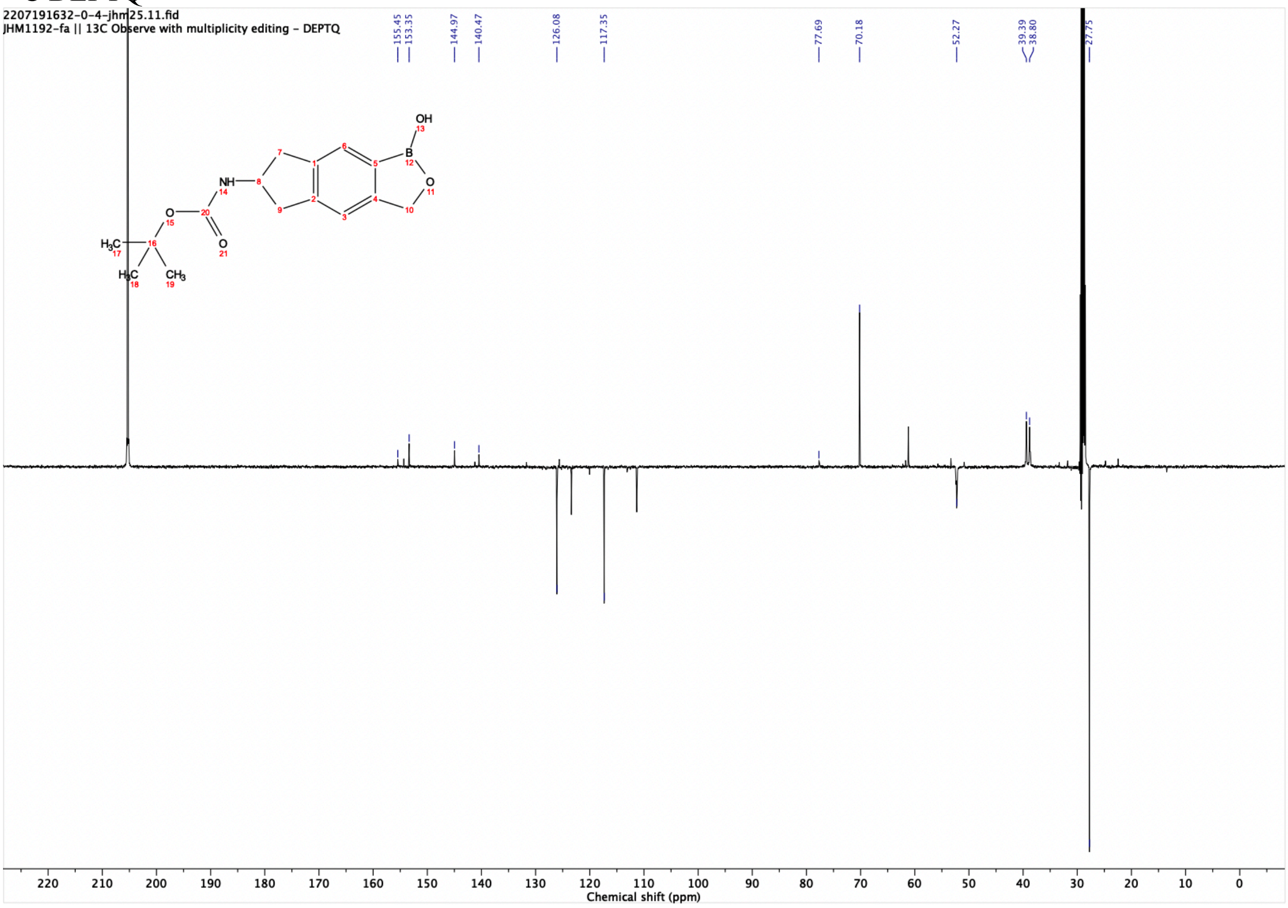

(12)

^{1}H

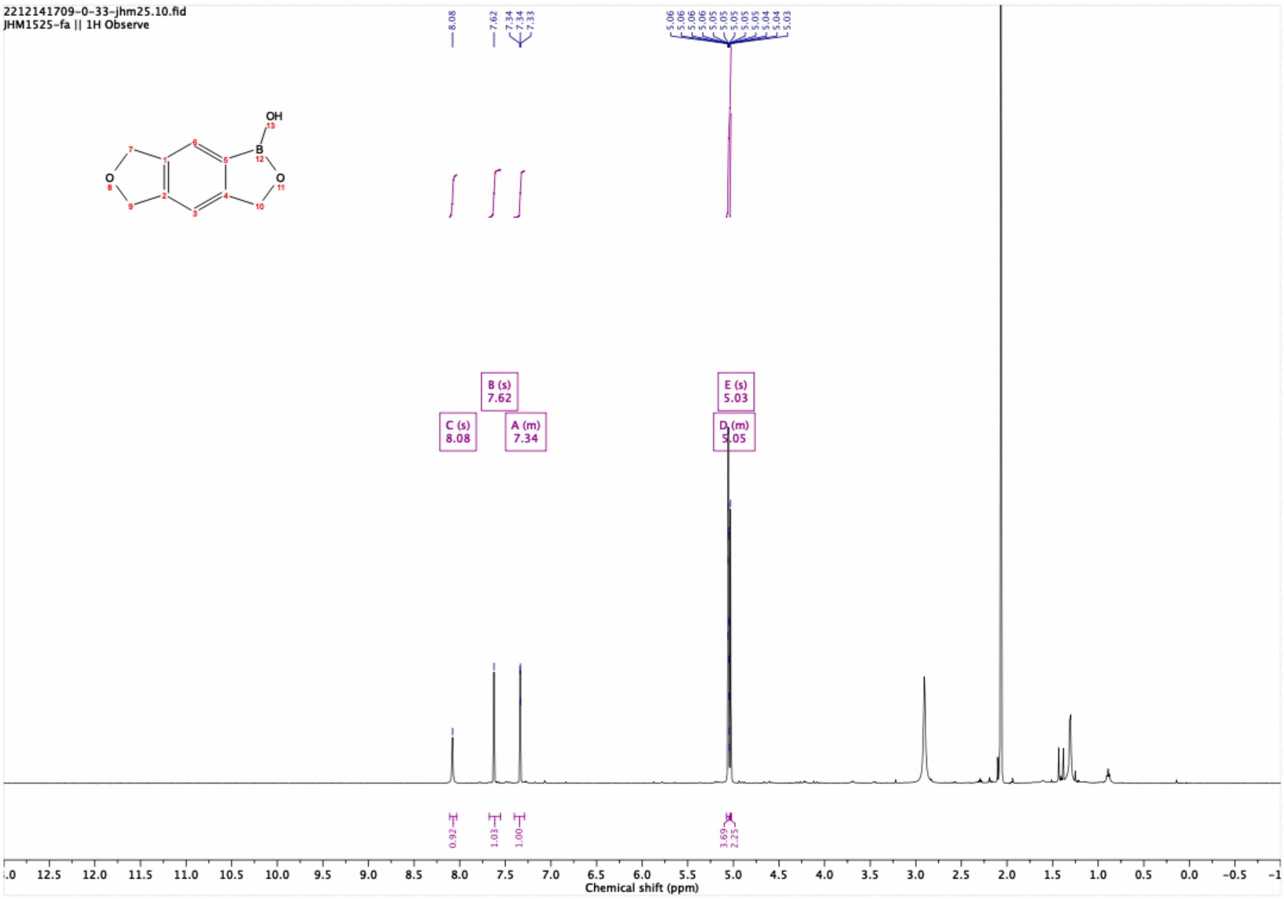

^{13}C DEPTQ

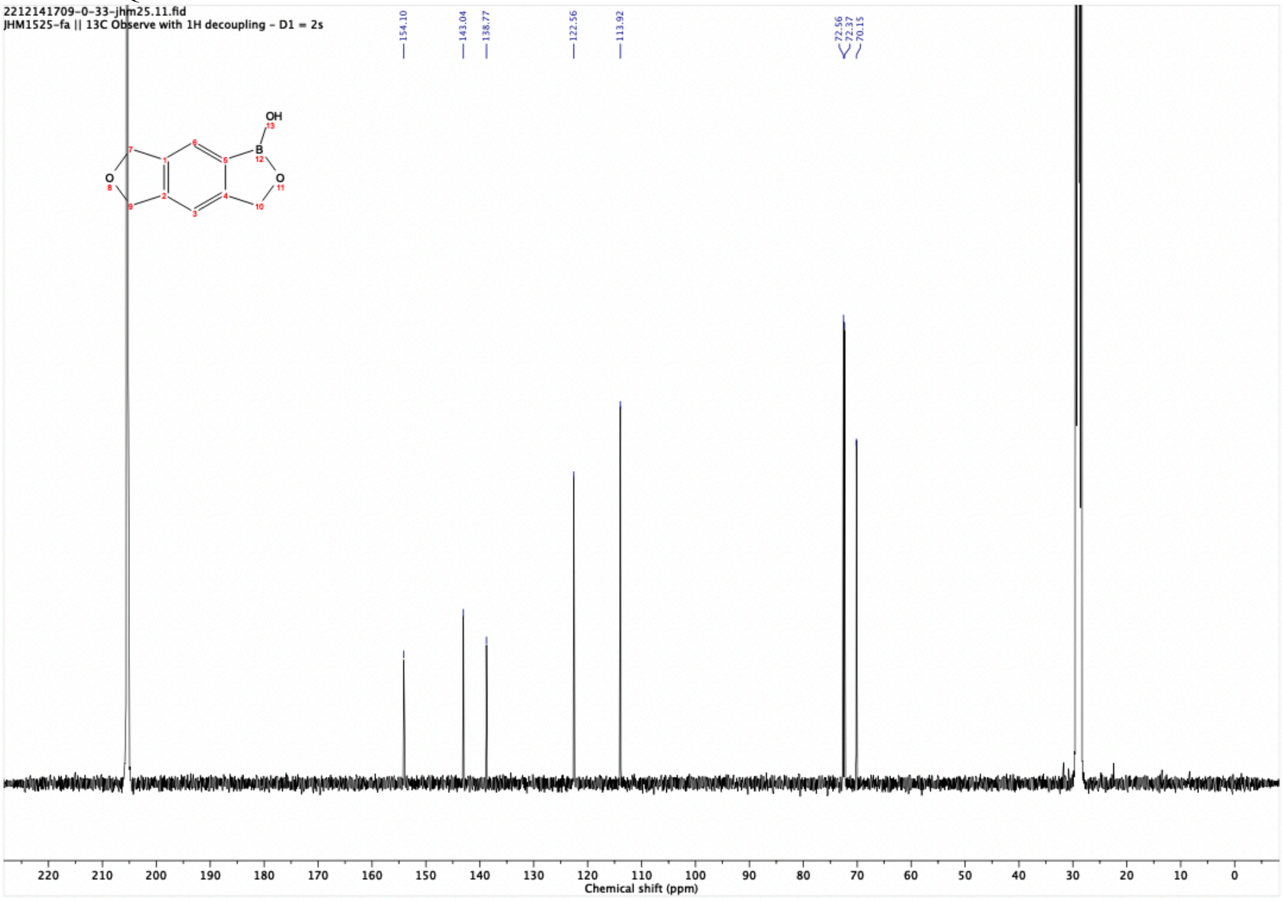

(13)

^{1}H

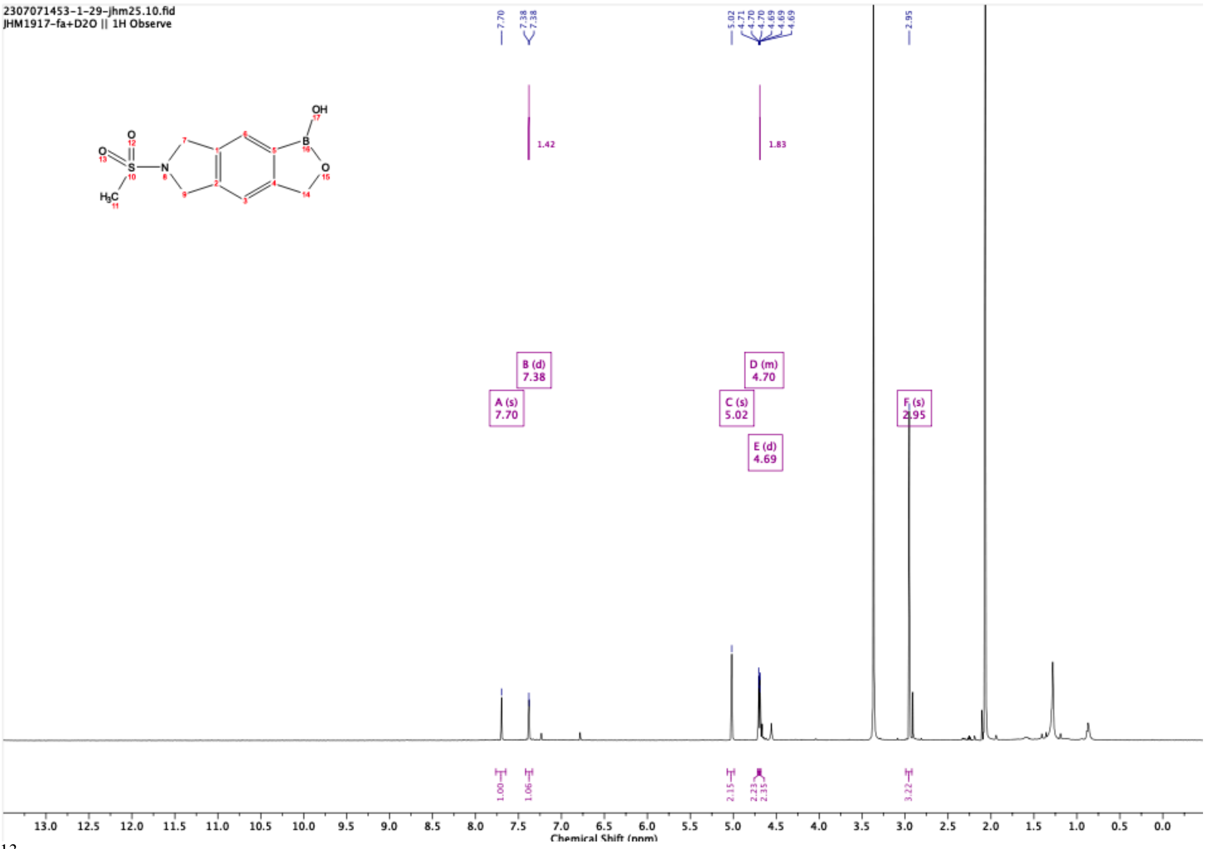

^{13}C DEPTQ

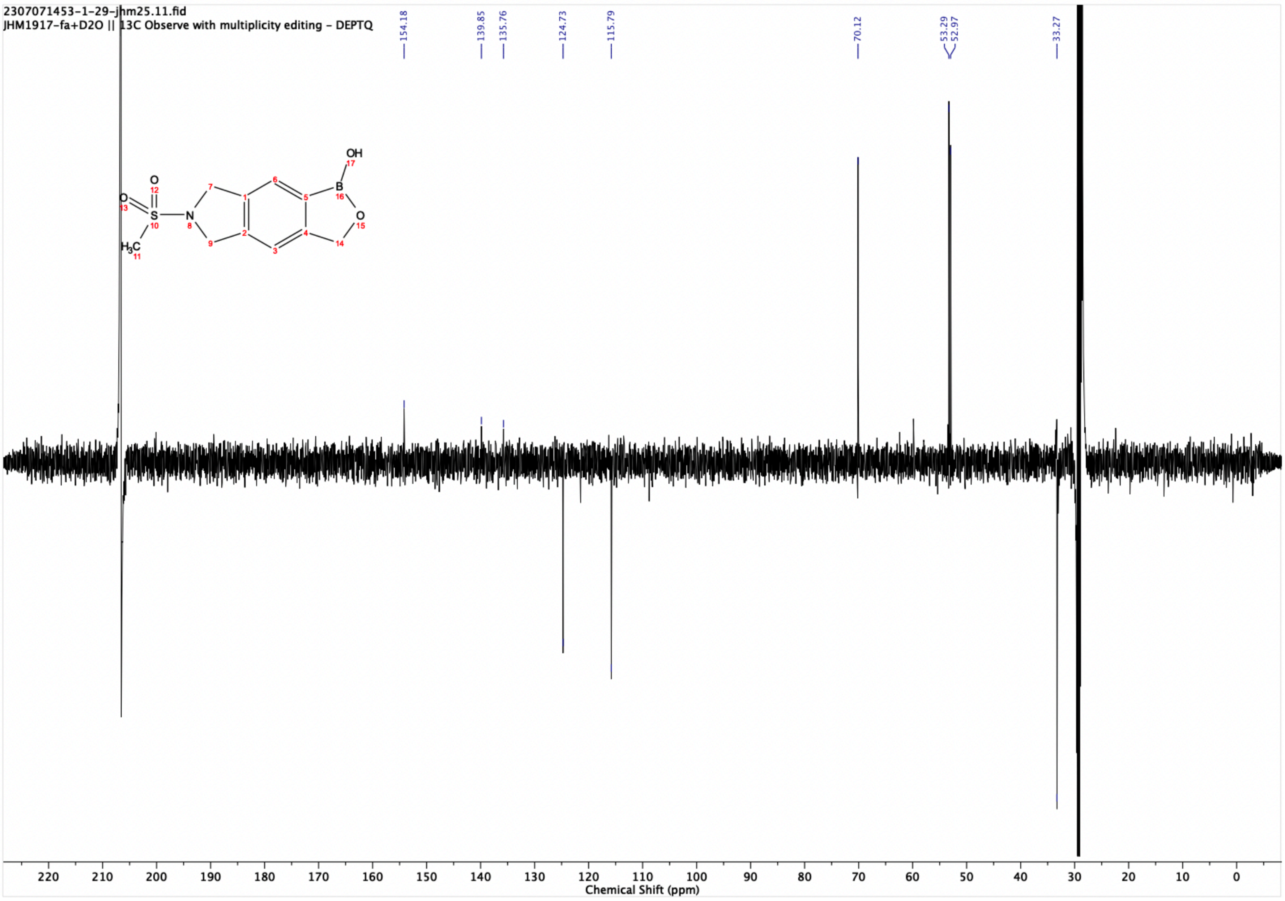

(14)

^{1}H

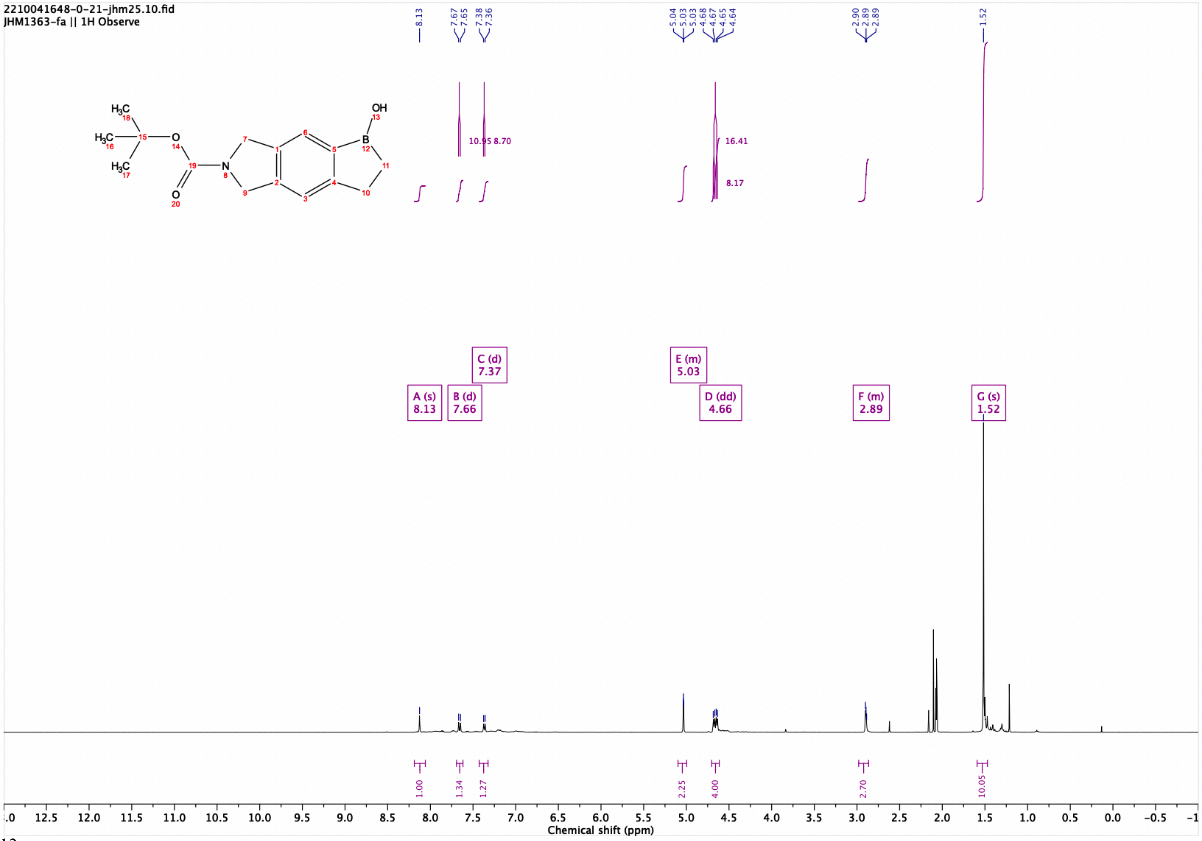

^{13}C DEPTQ

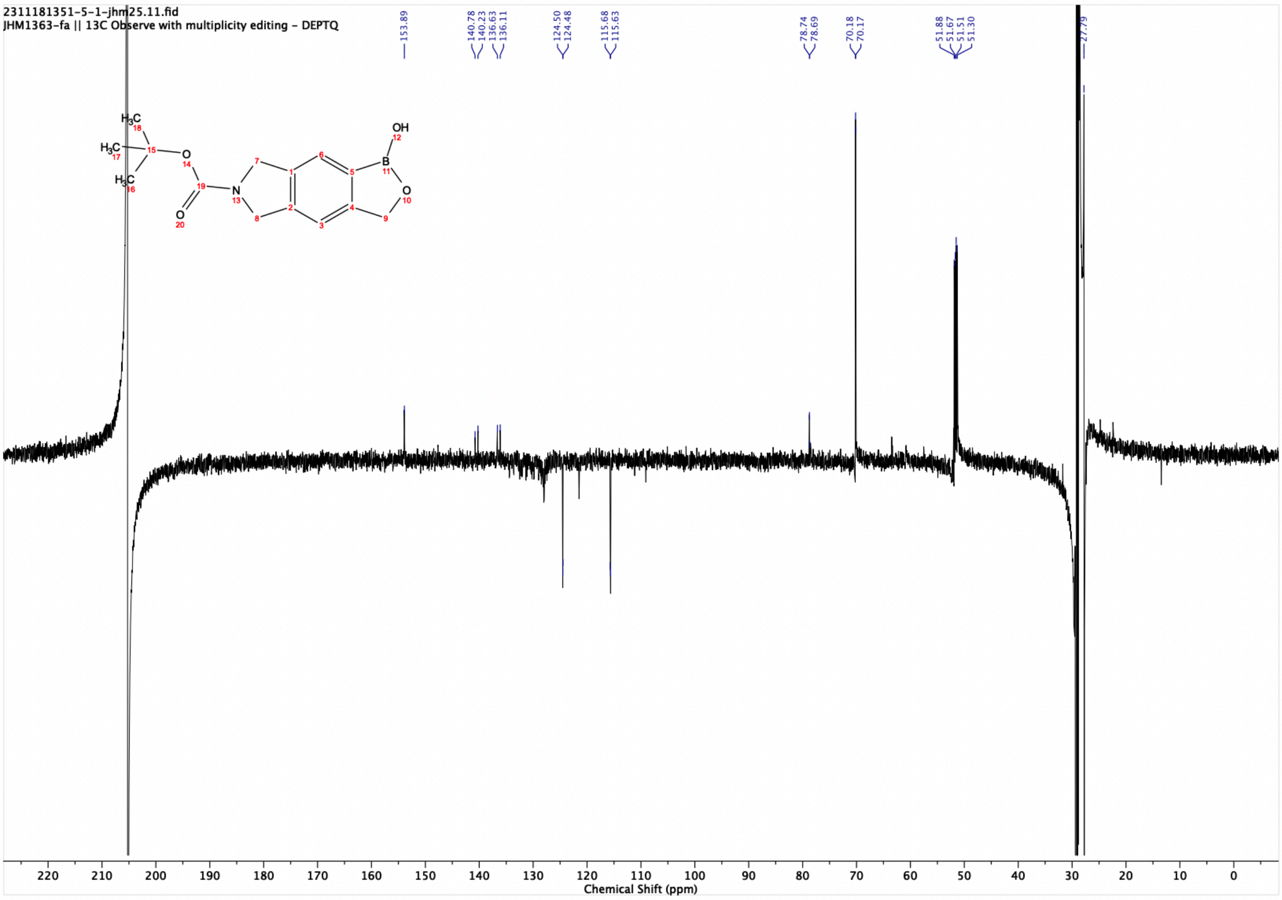

(15)

^{1}H

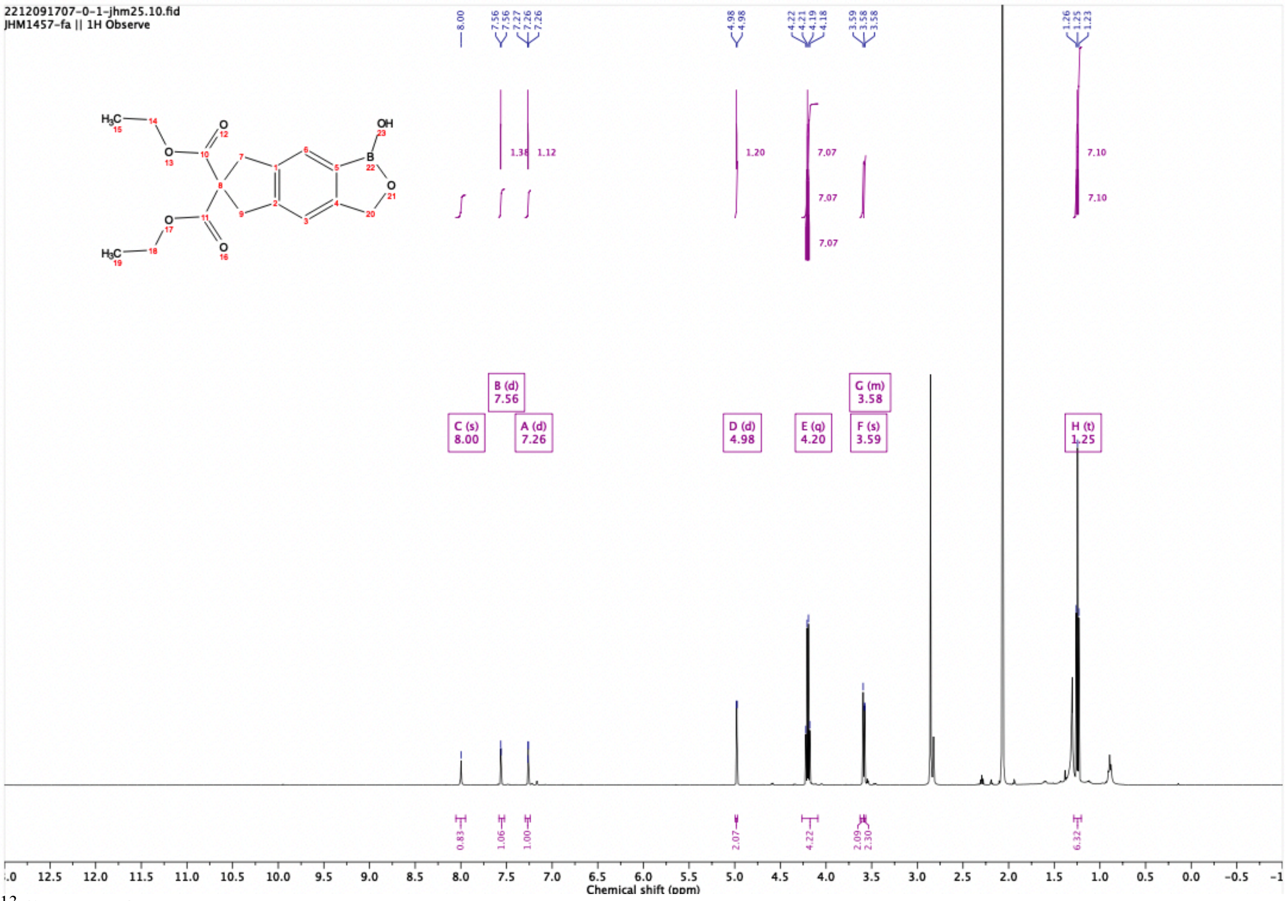

^{13}C DEPTQ

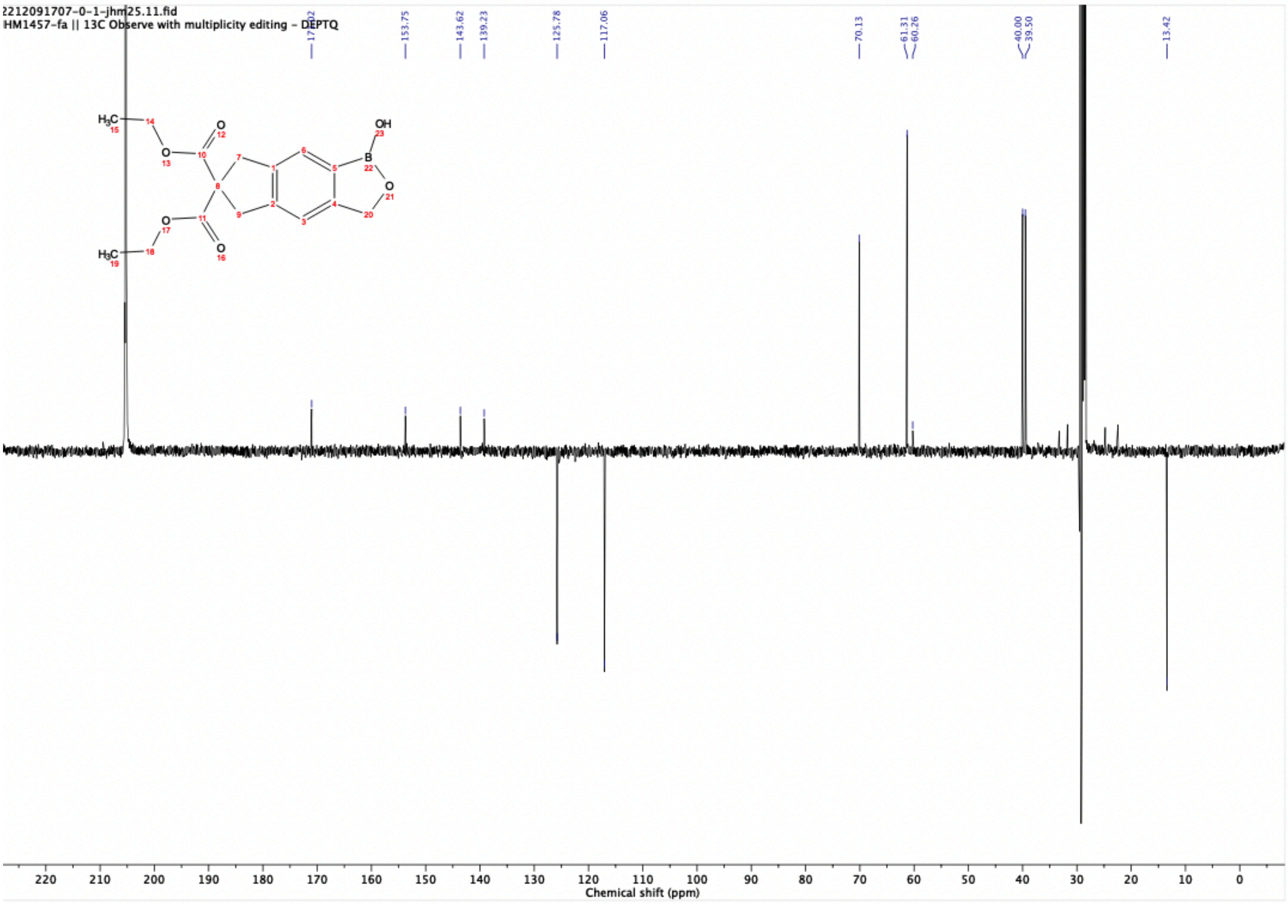

(16)

^{1}H

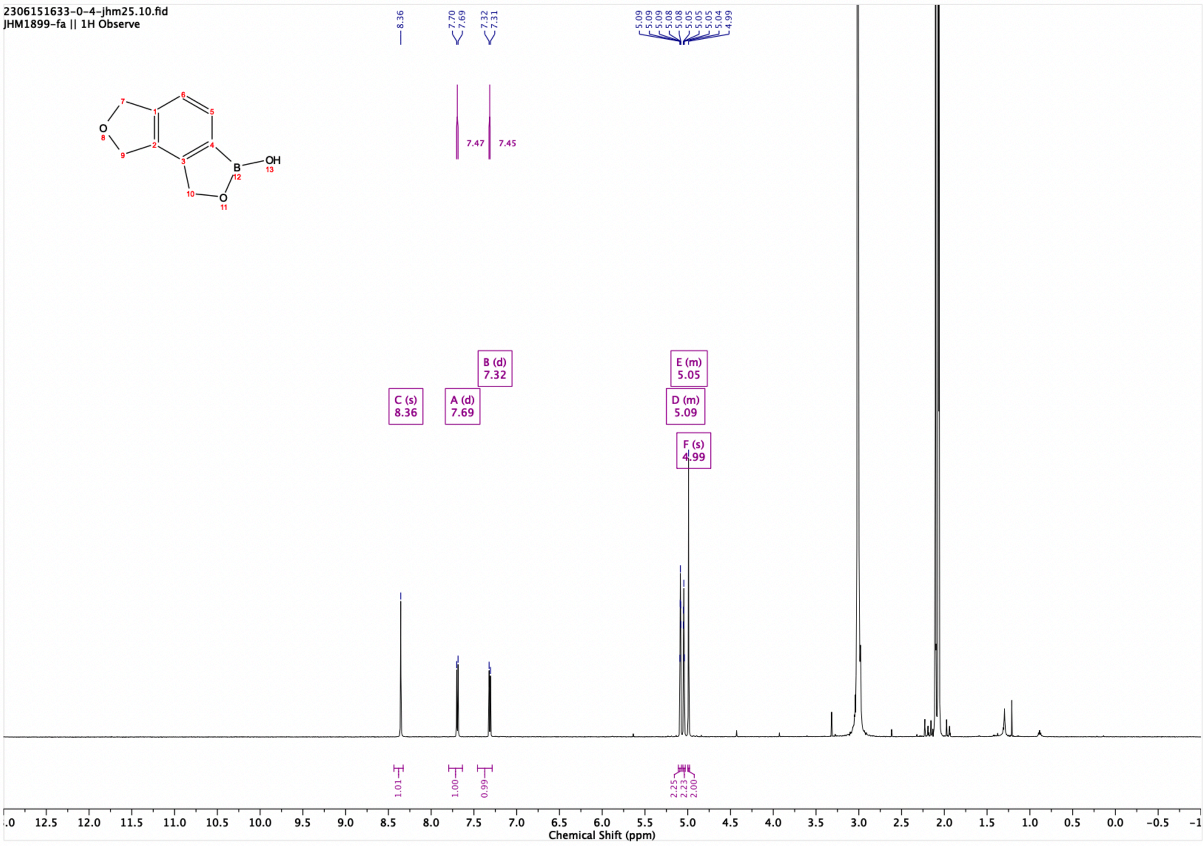

^{13}C DEPTQ

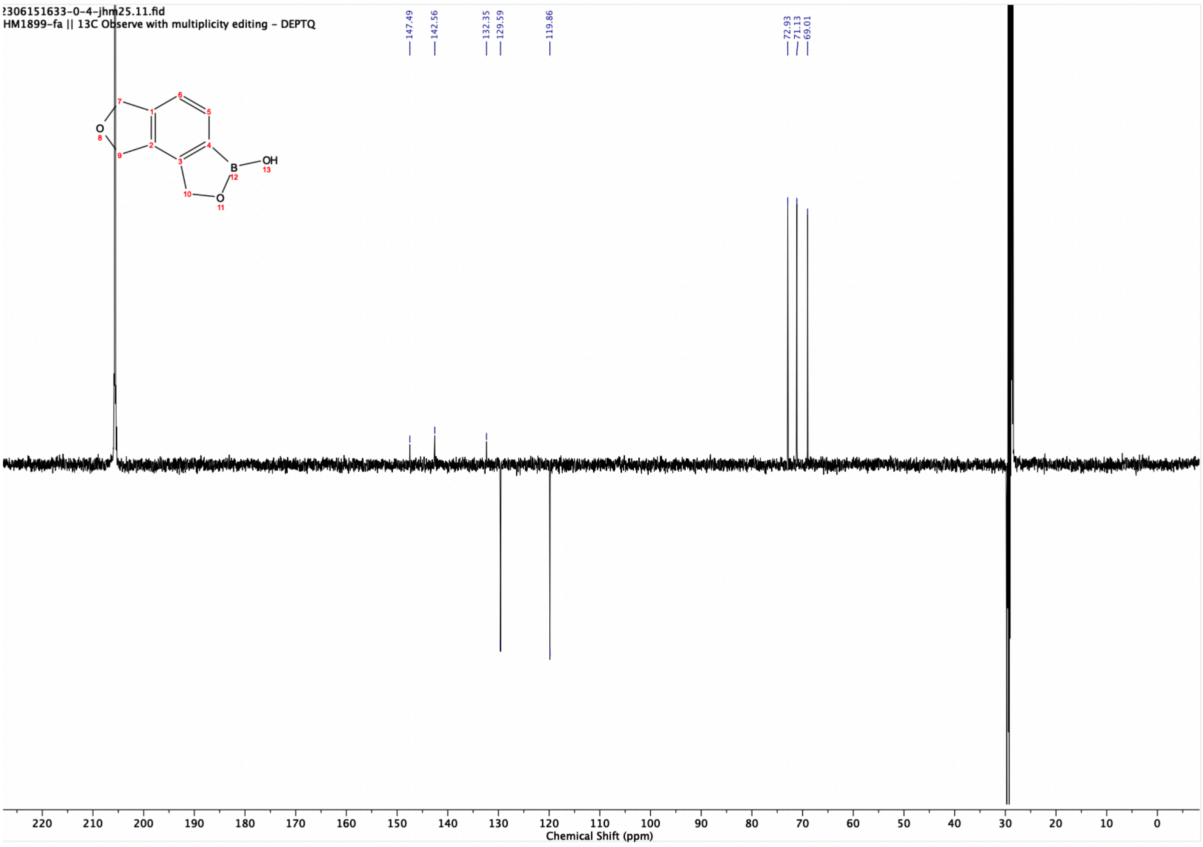

(17)

^{1}H

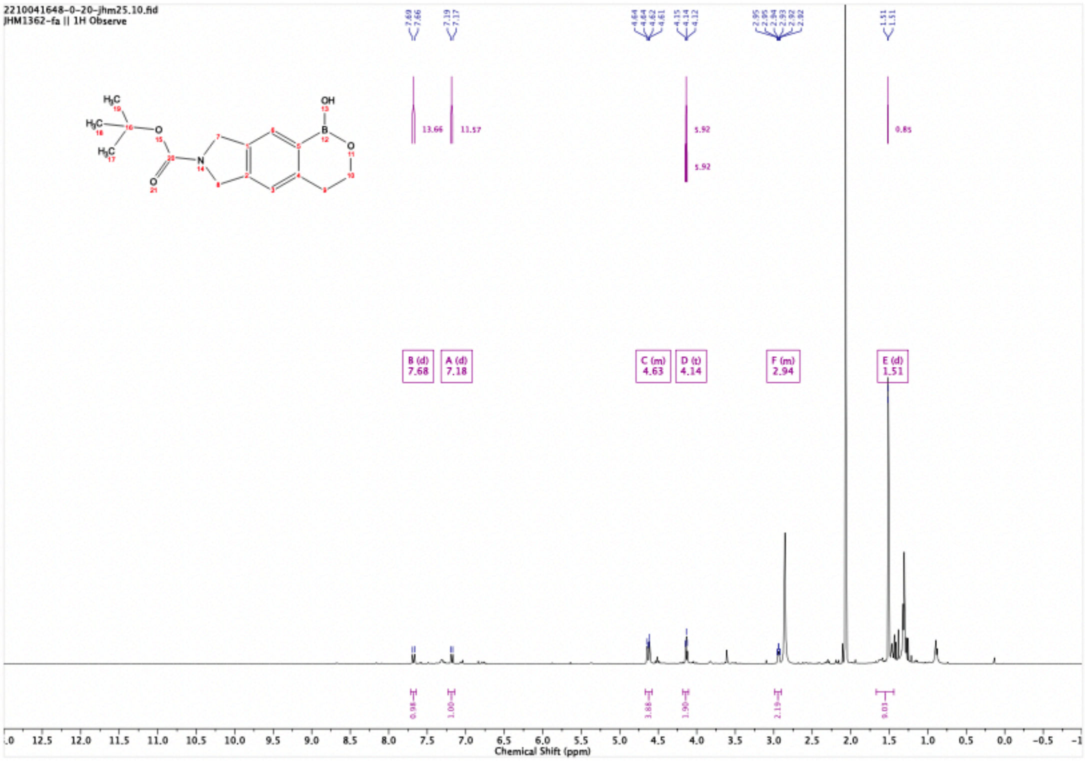

^{13}C DEPTQ

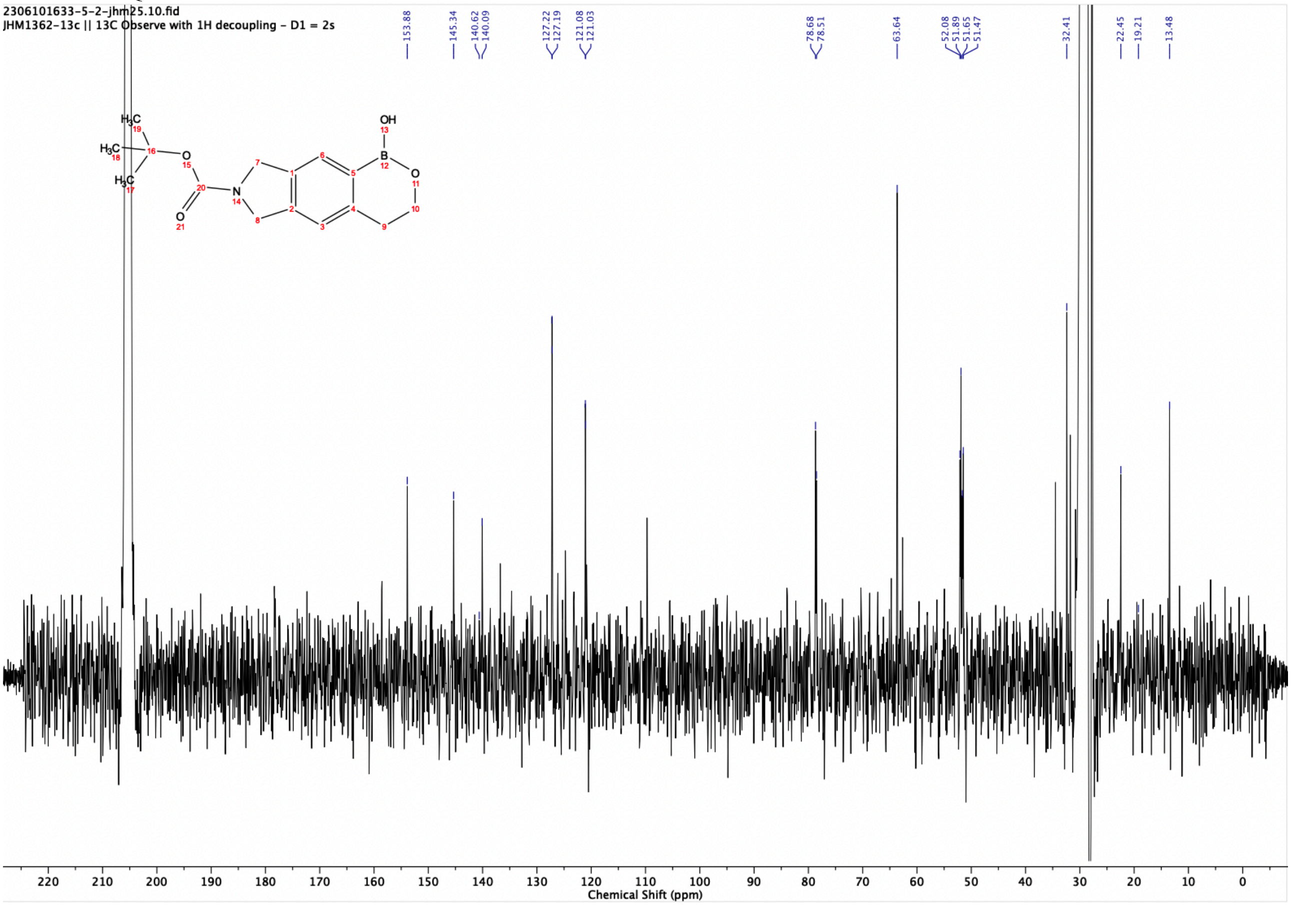

(18)

^{1}H

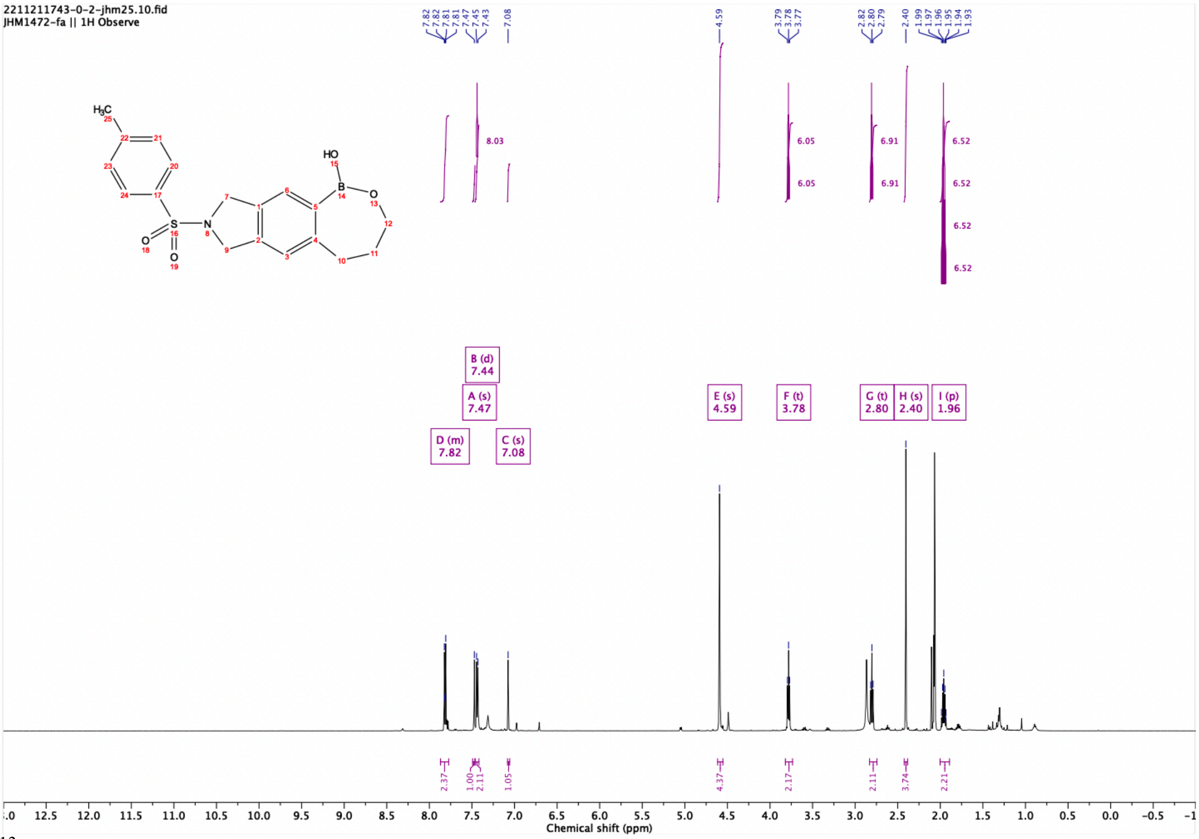

^{13}C DEPTQ

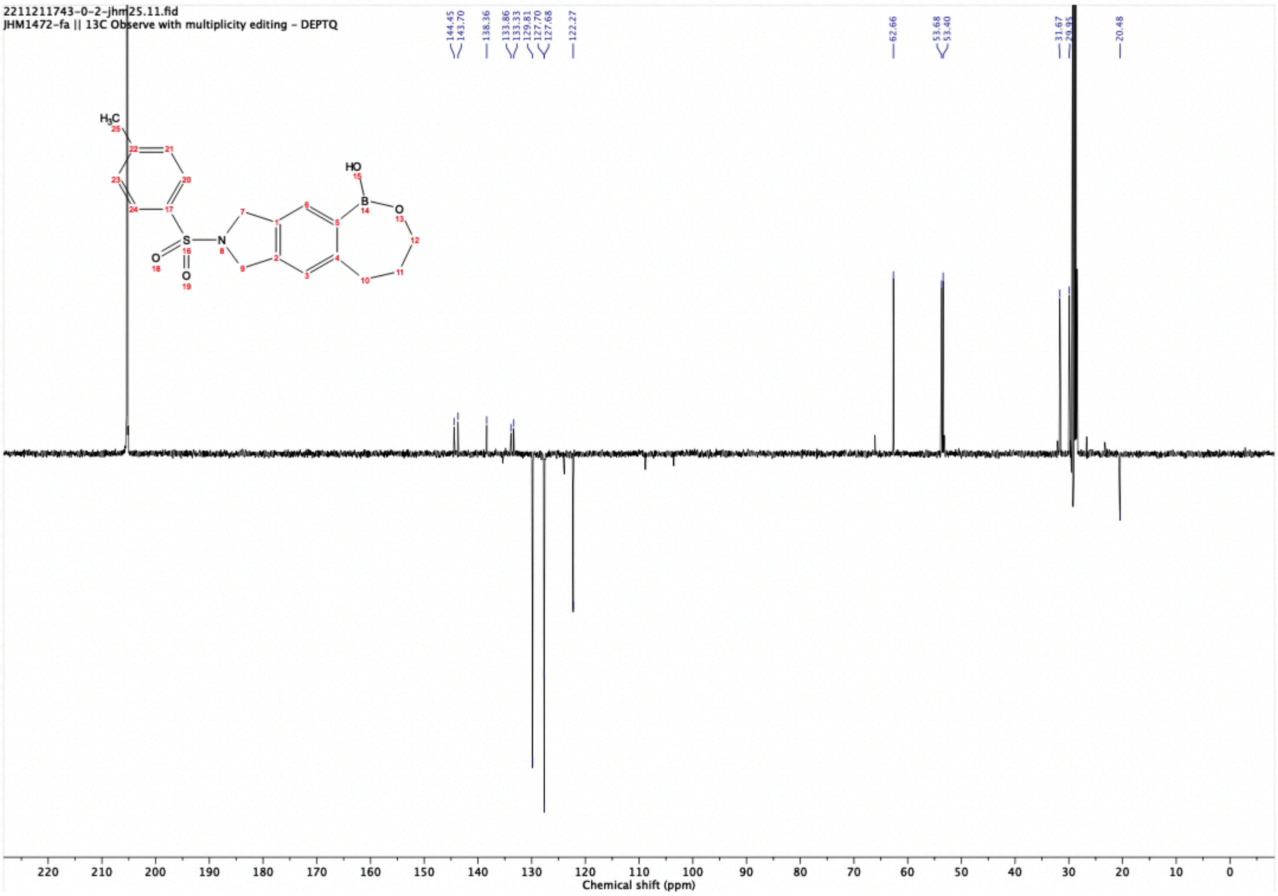

(19)

^{1}H

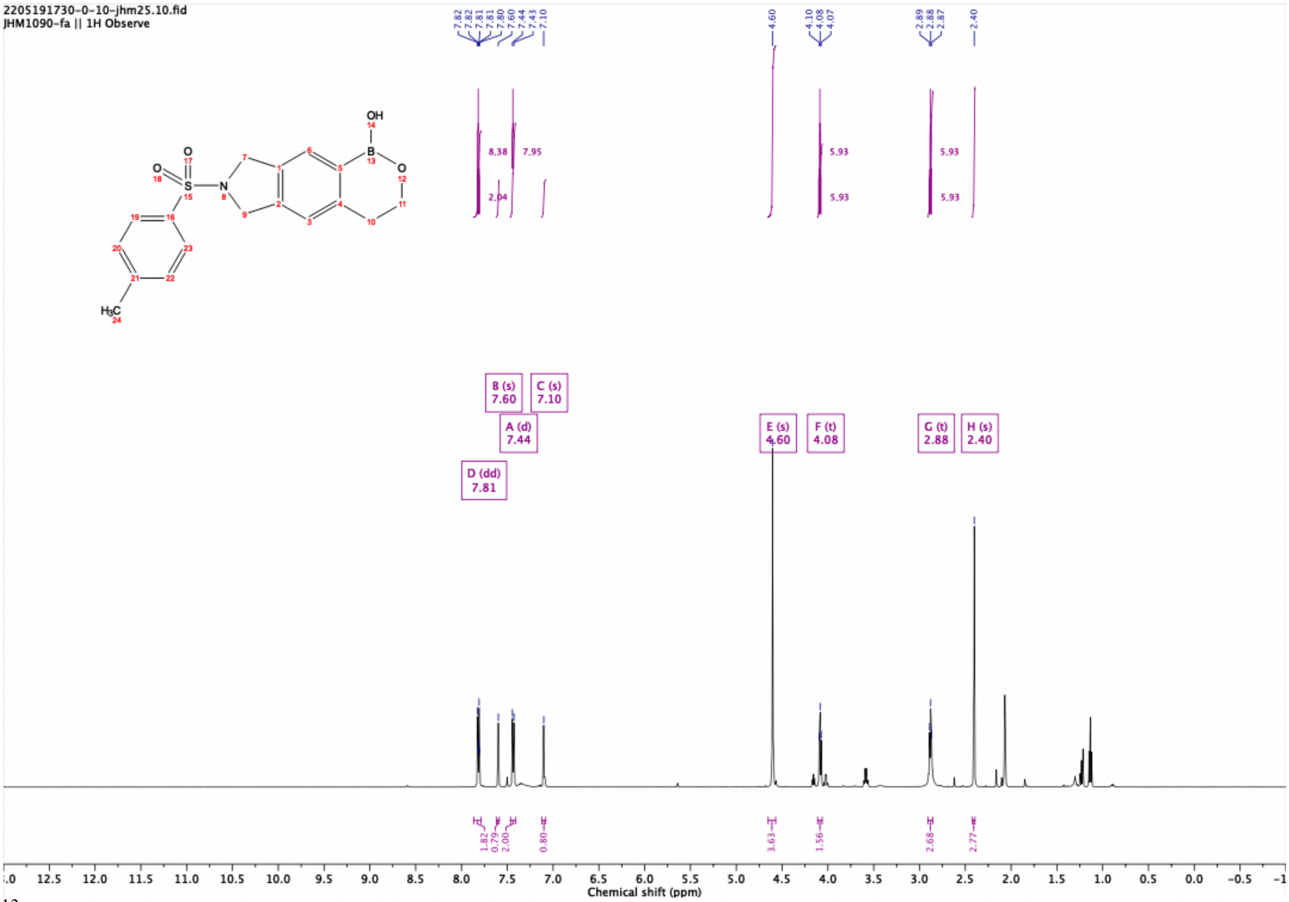

^{13}C DEPTQ

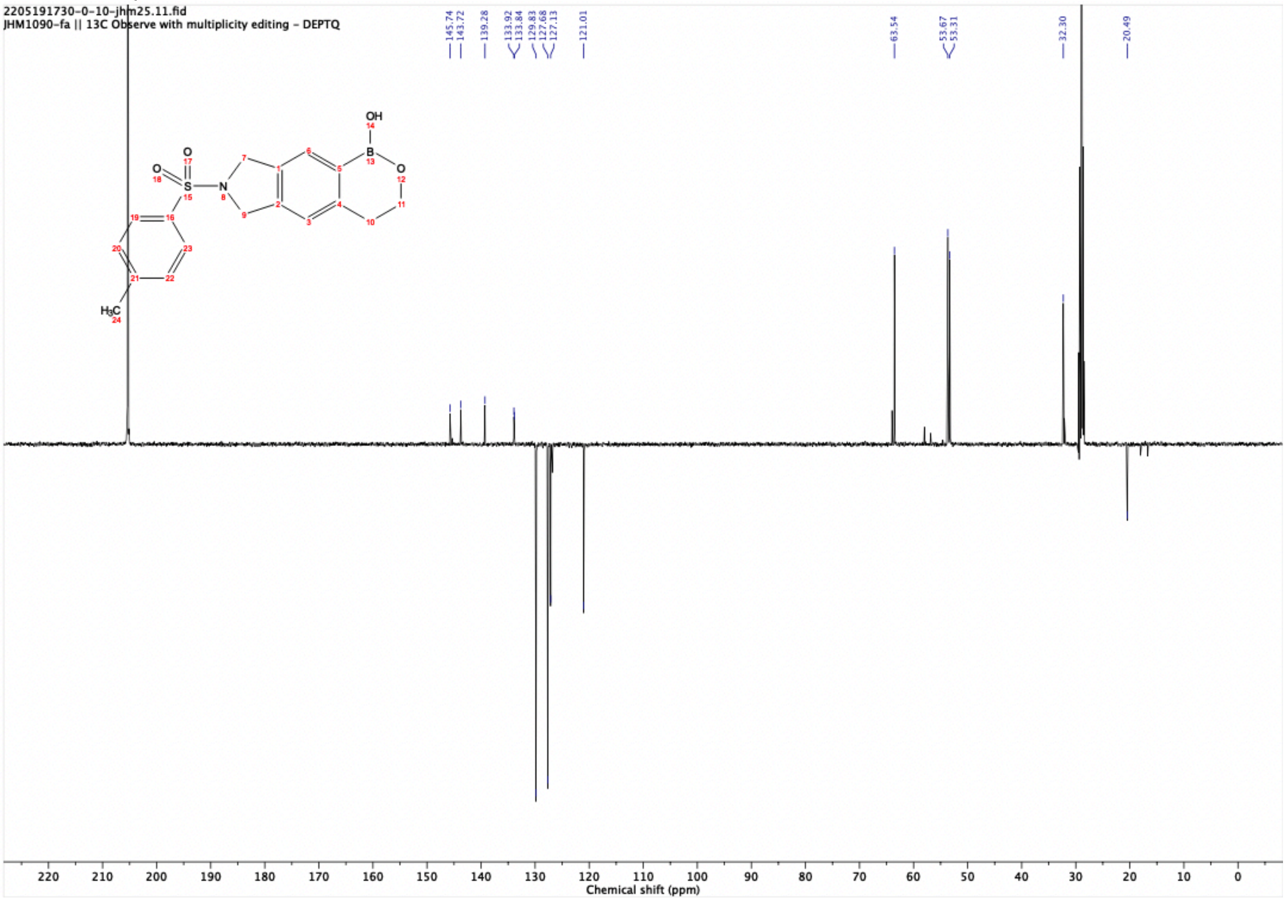

(20)

^{1}H

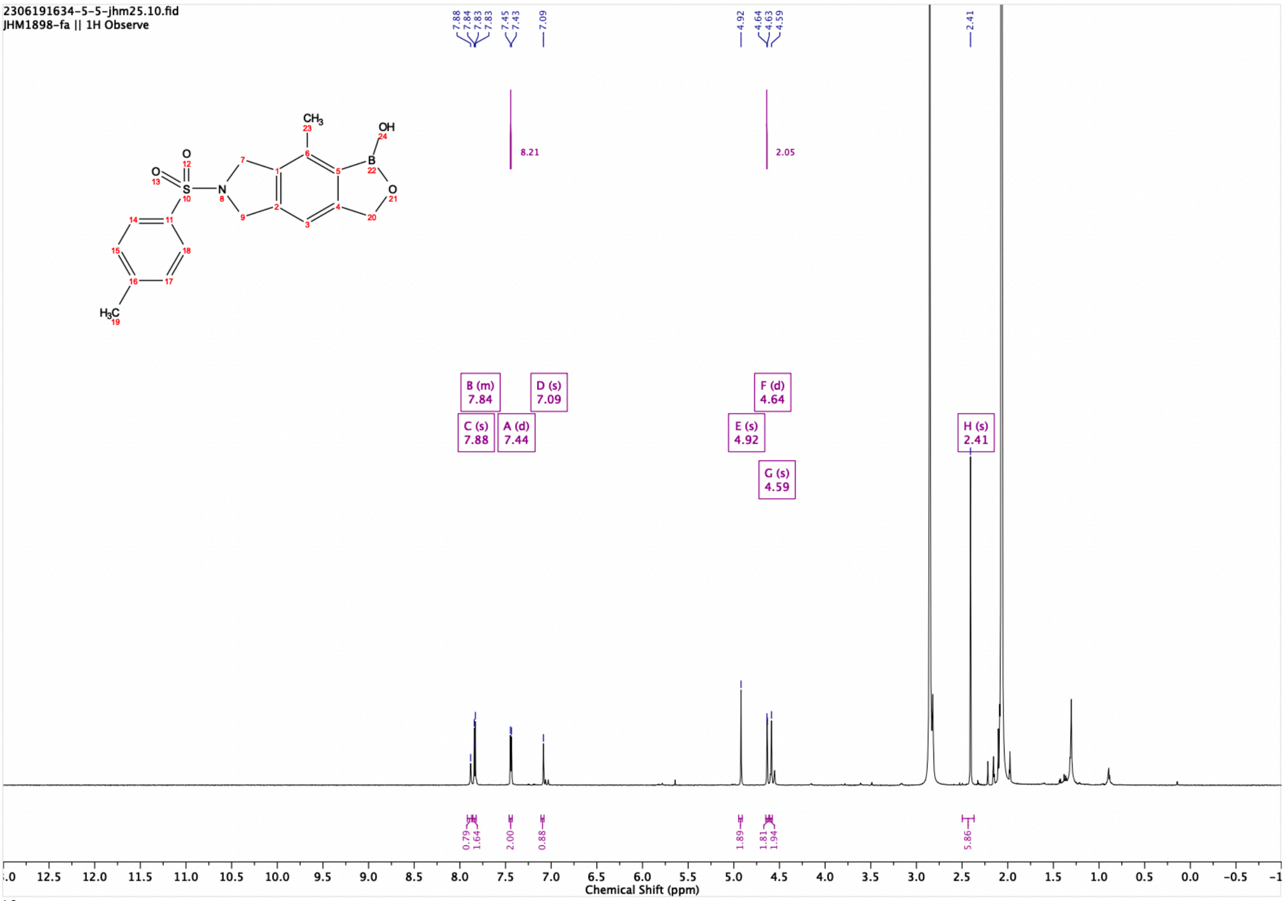

^{13}C DEPTQ

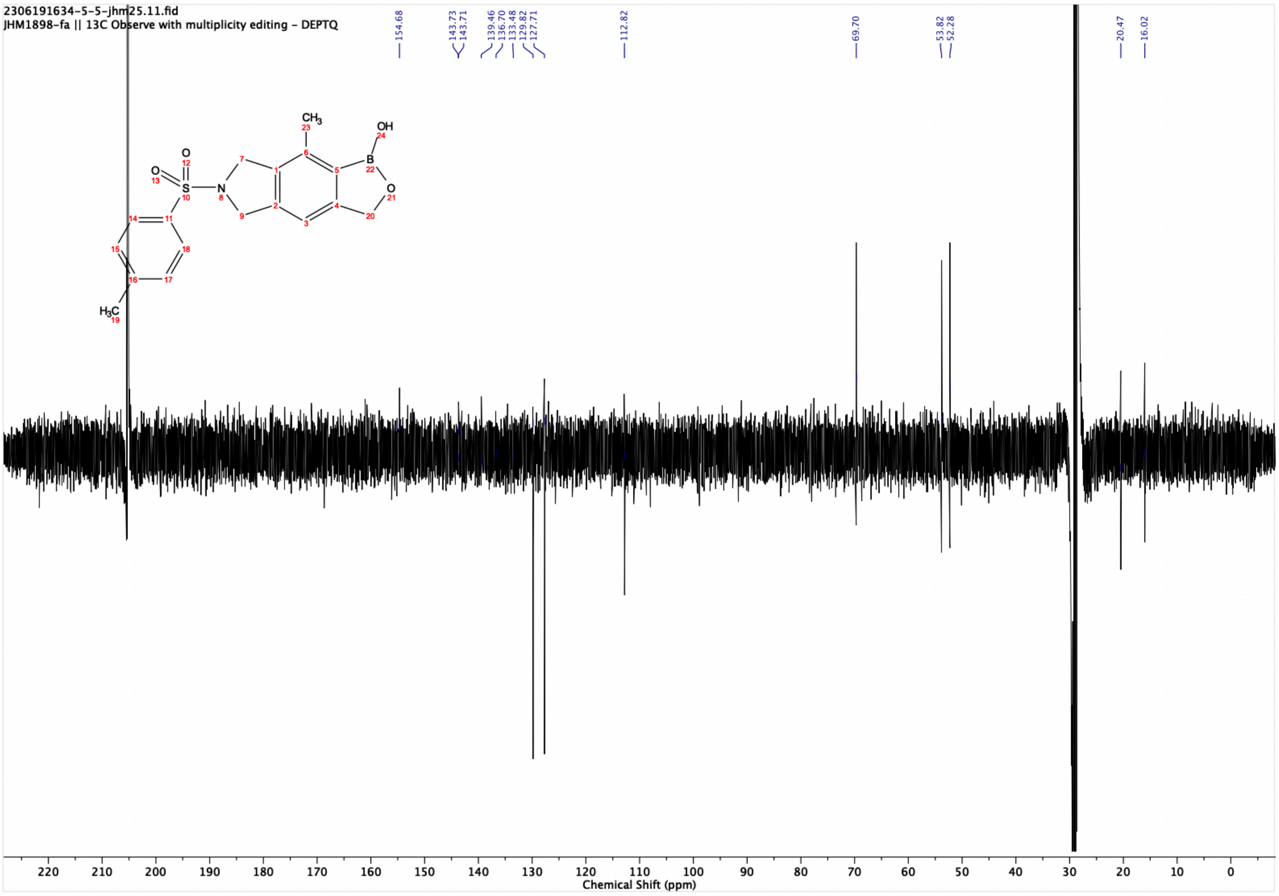

^{1}H-^{13}C HMBC

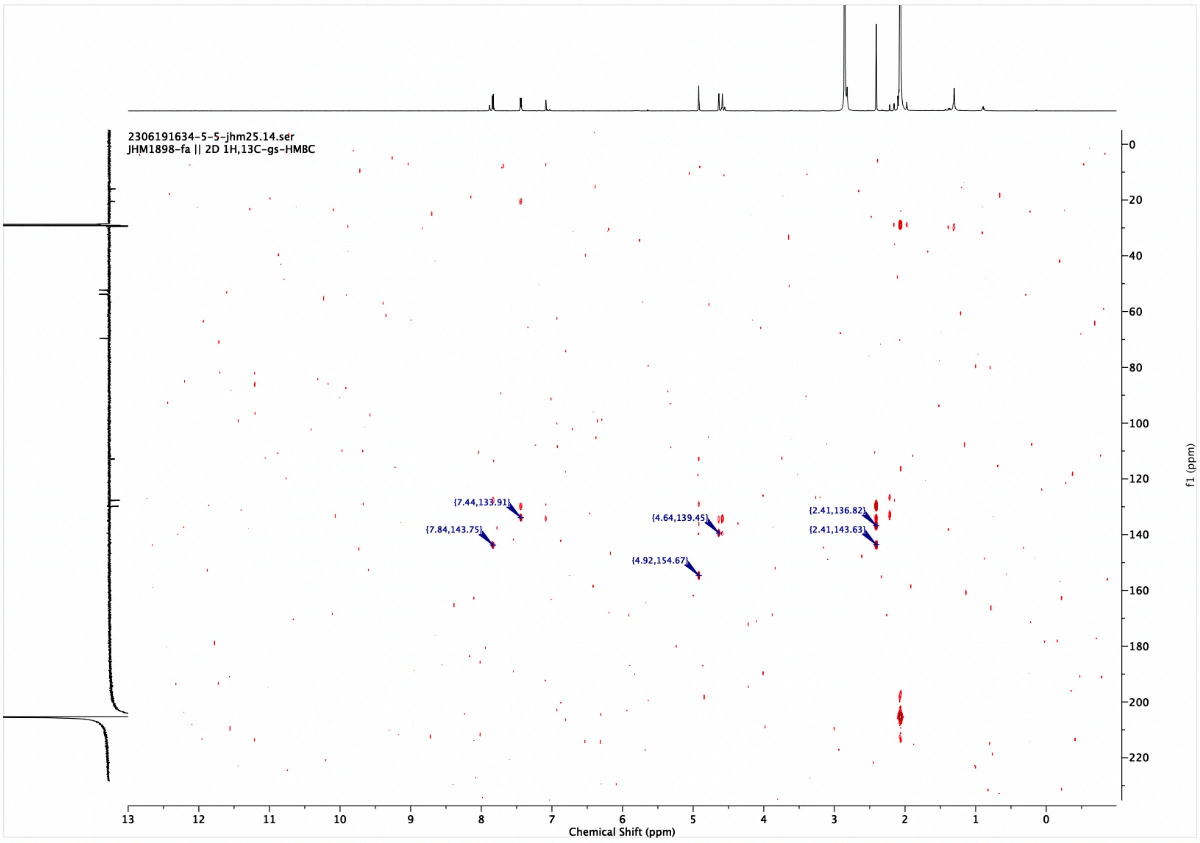

2306191634-5-5-jhm25.14.ser
JHM1898-fa || 2D 1H,13C-gs-HMBC
(7.44,133.91)
(7.84,143.75)
(4.64,139.45)
(4.92,154.67)
(2.41,136.82)
(2.41,143.63)
Chemical Shift (ppm)
f1 (ppm)

(21)

^{1}H

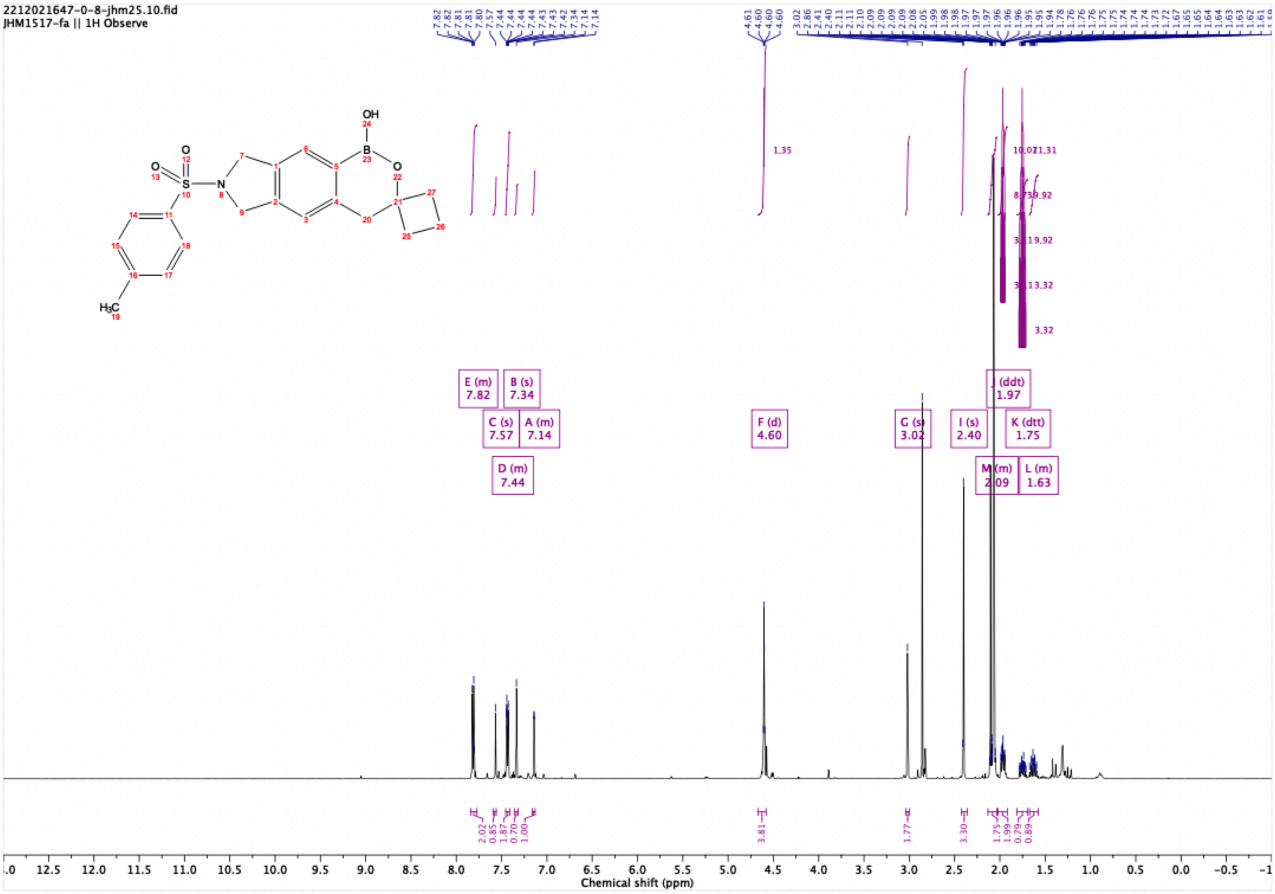

^{13}C DEPTQ

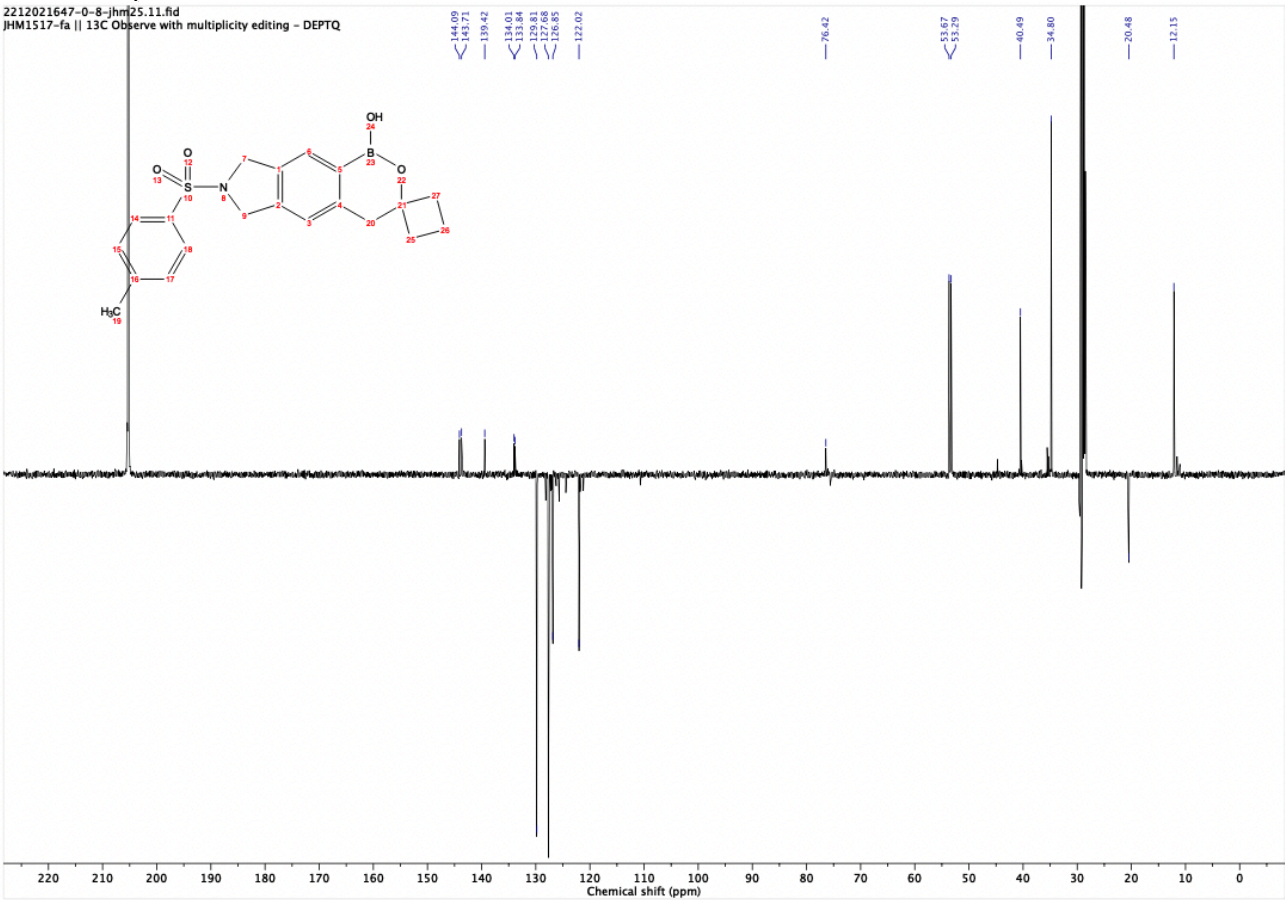

(22)

^{1}H

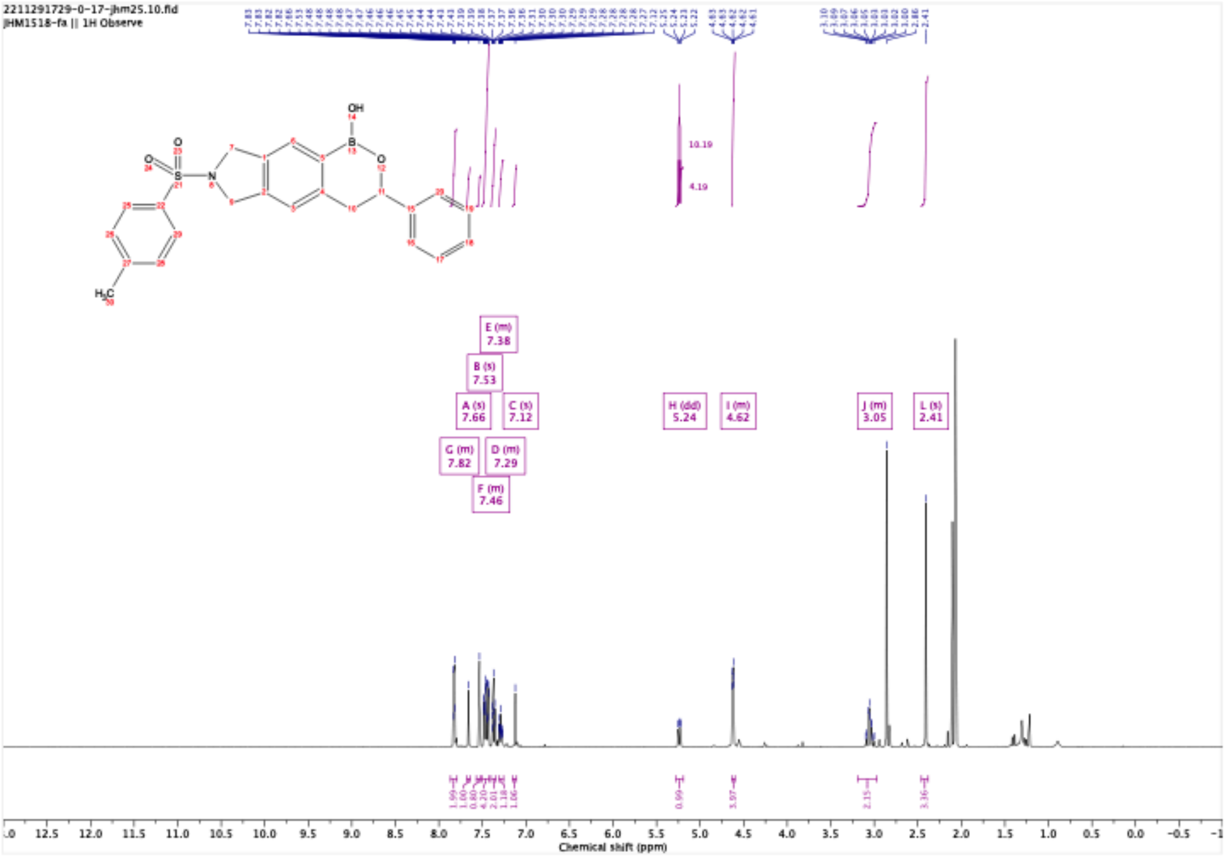

^{13}C DEPTQ

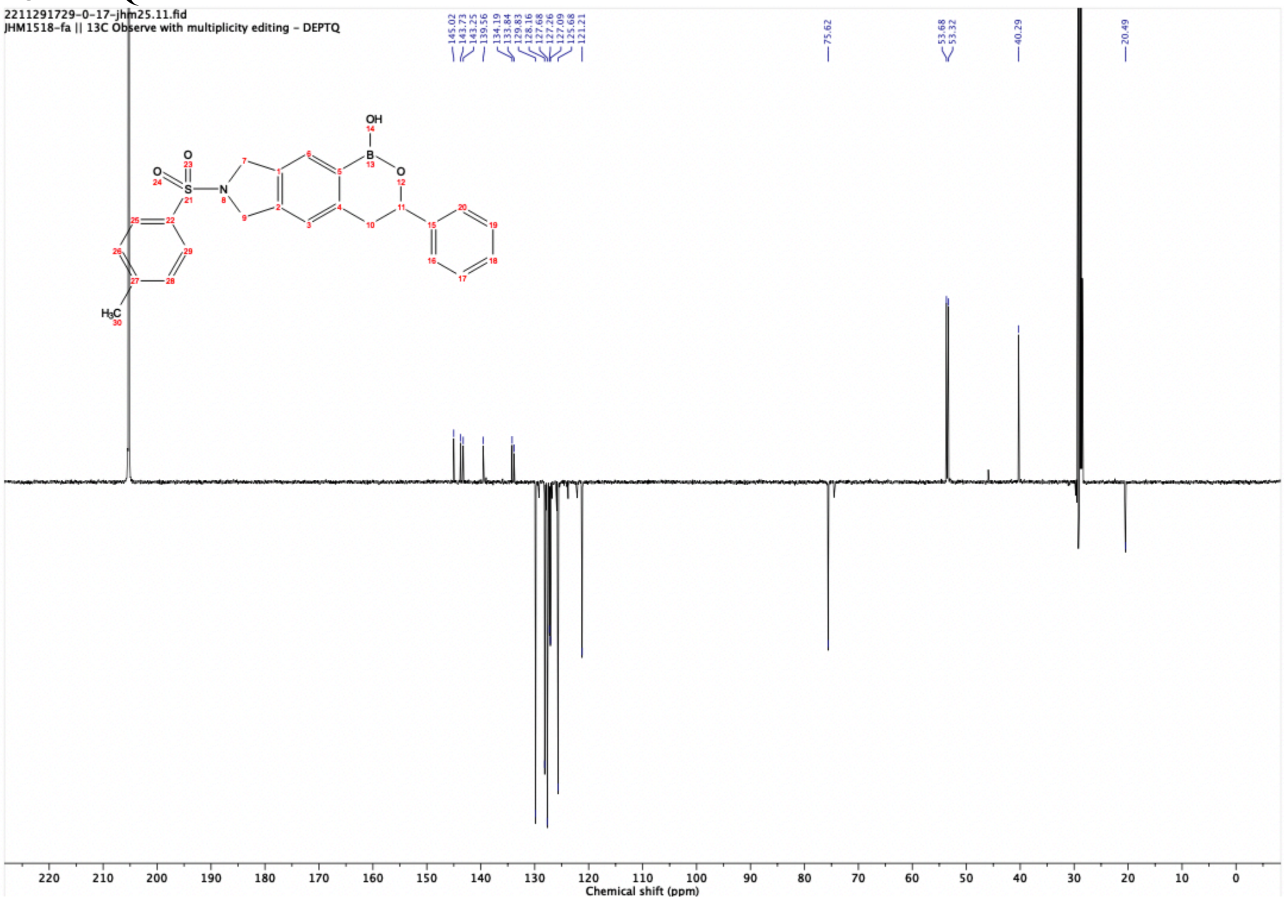

(23)

^{1}H

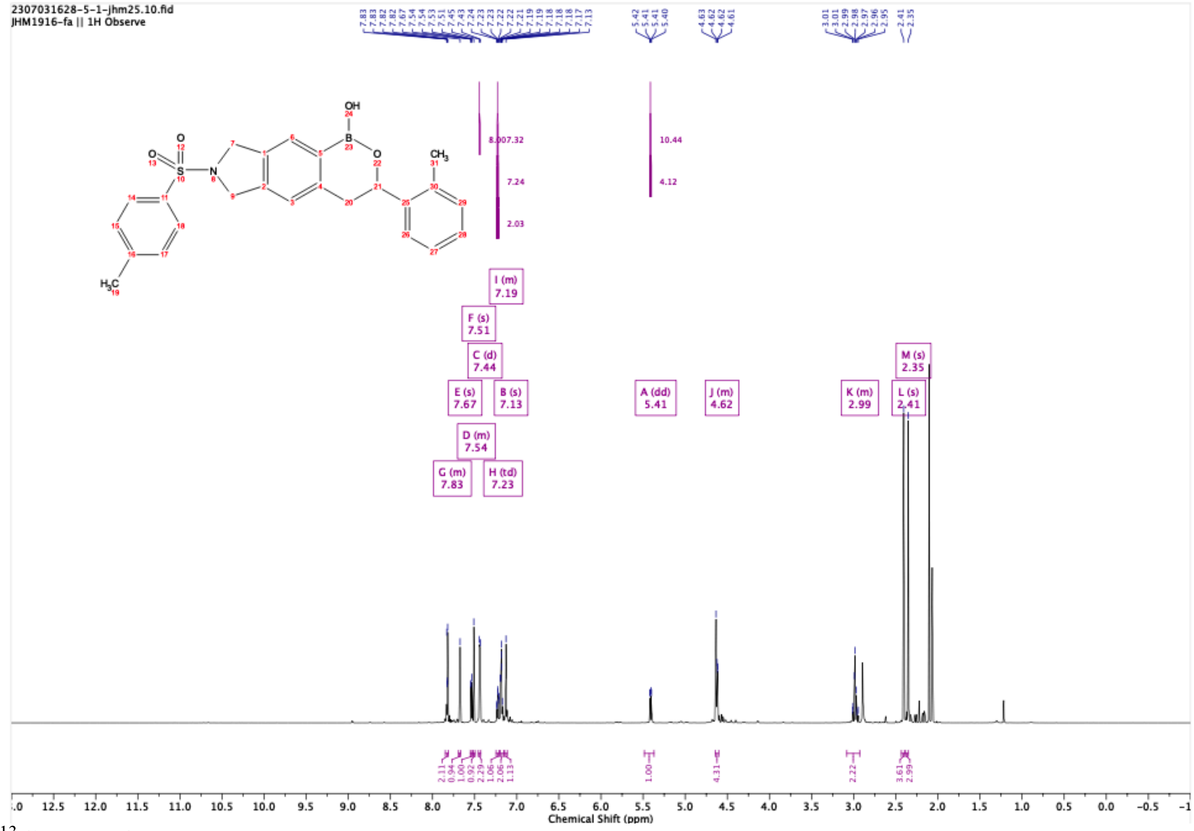

^{13}C DEPTQ

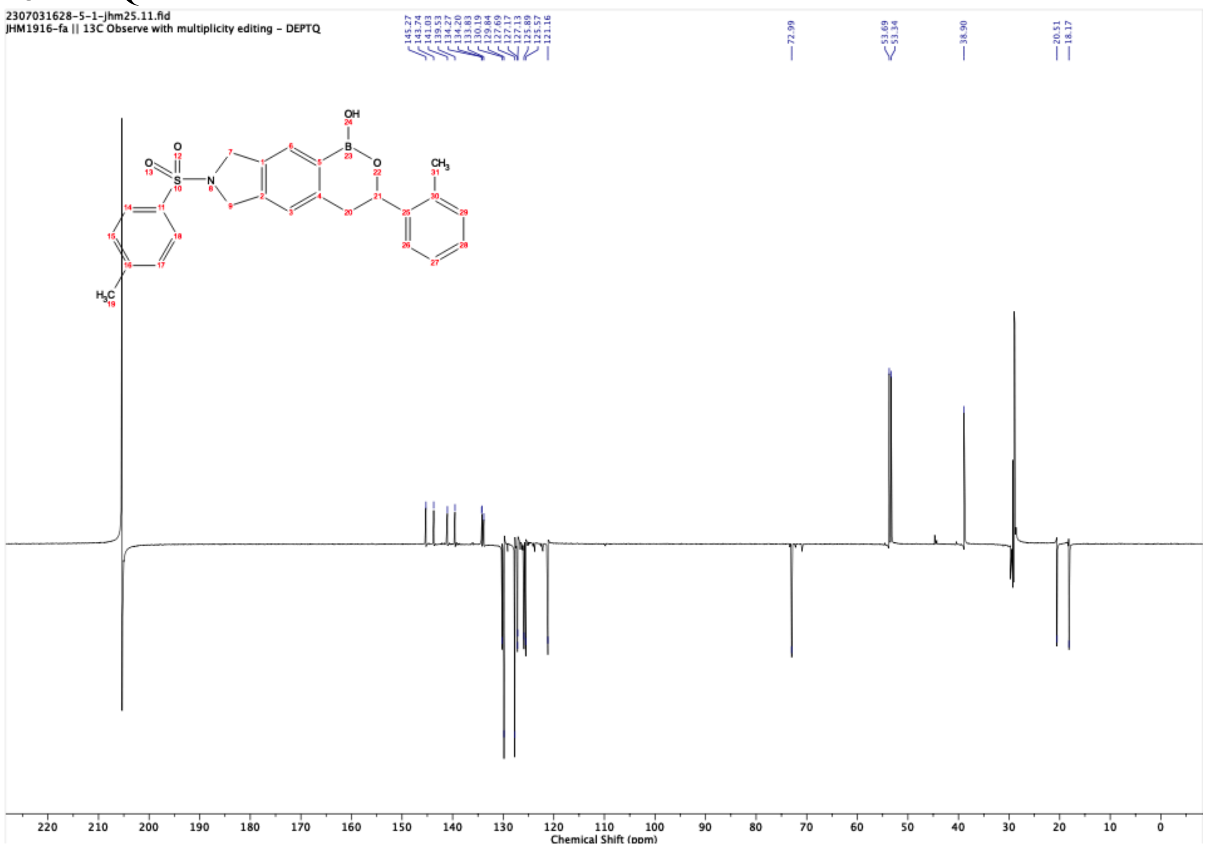

(24)

^{1}H

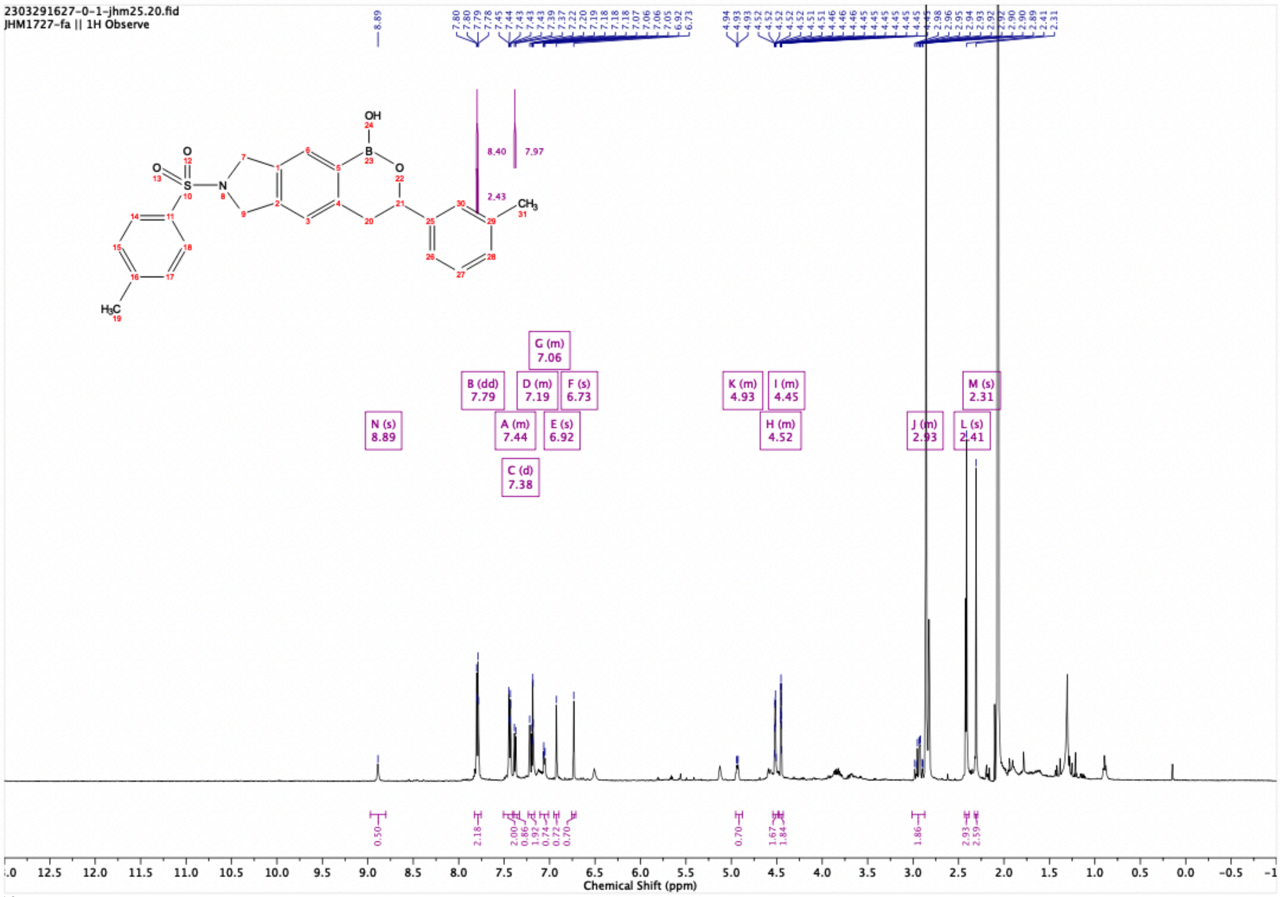

^{13}C DEPTQ

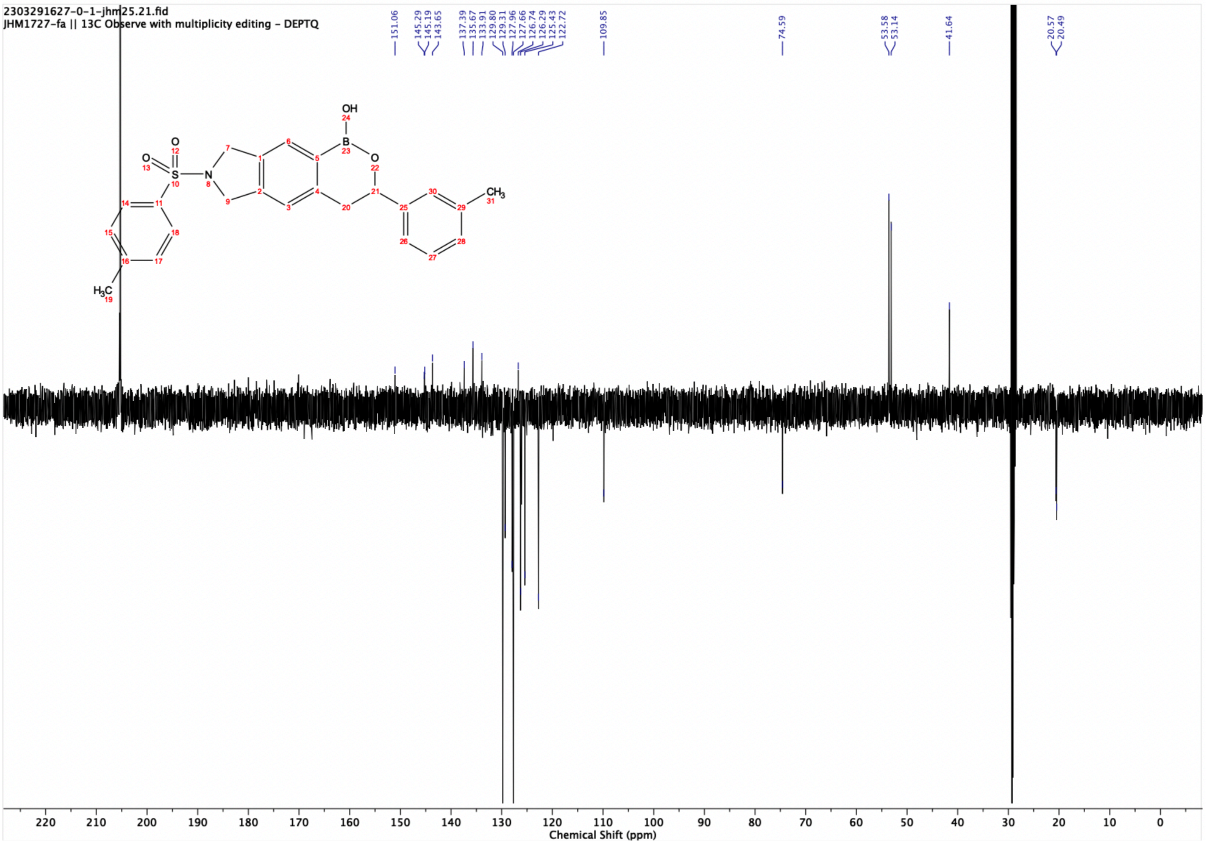

(25)

^{1}H

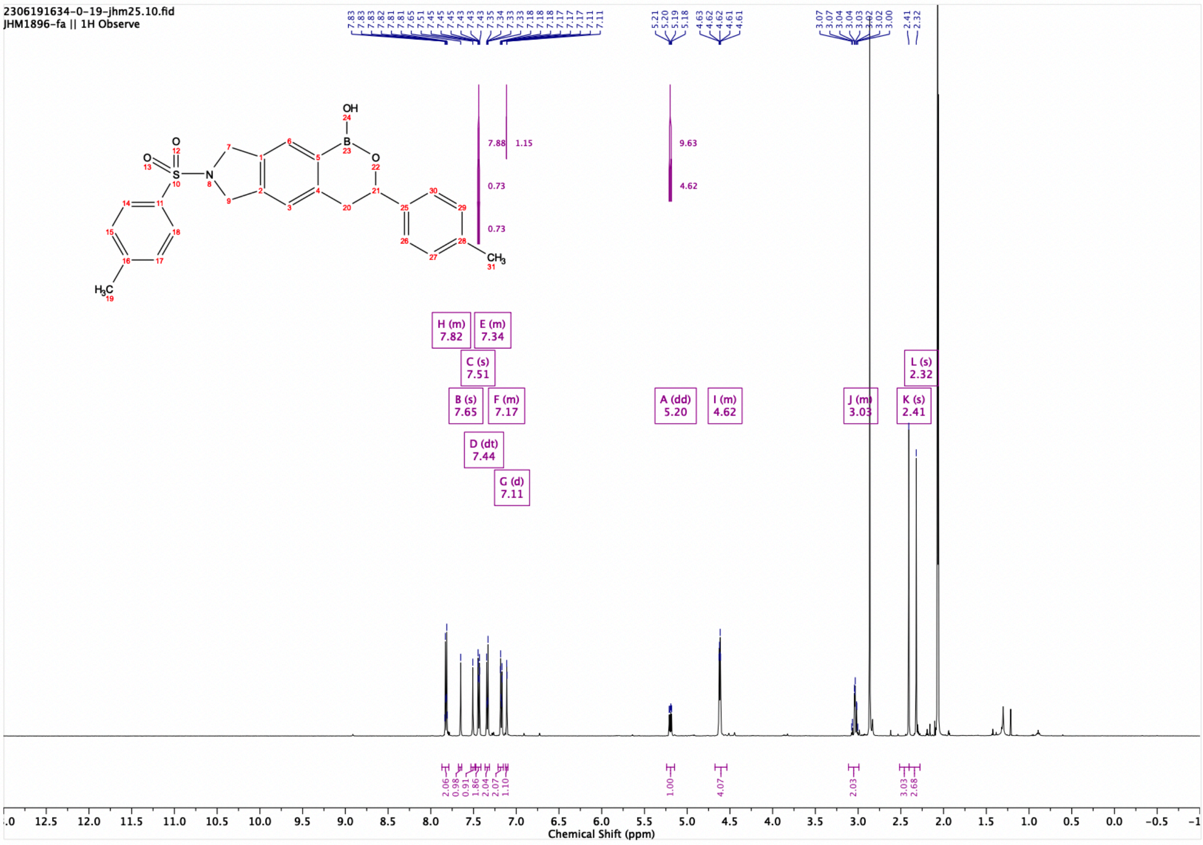

^{13}C DEPTQ

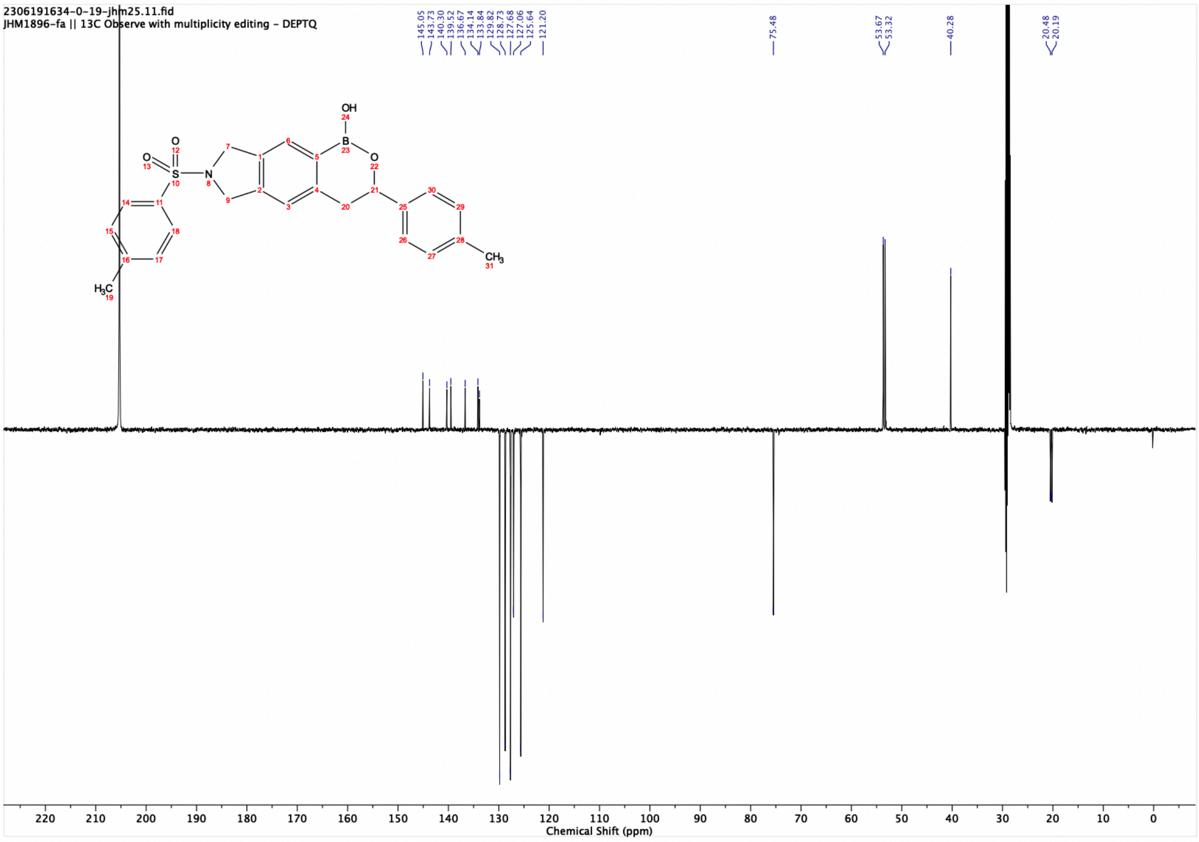

(26)

^{1}H

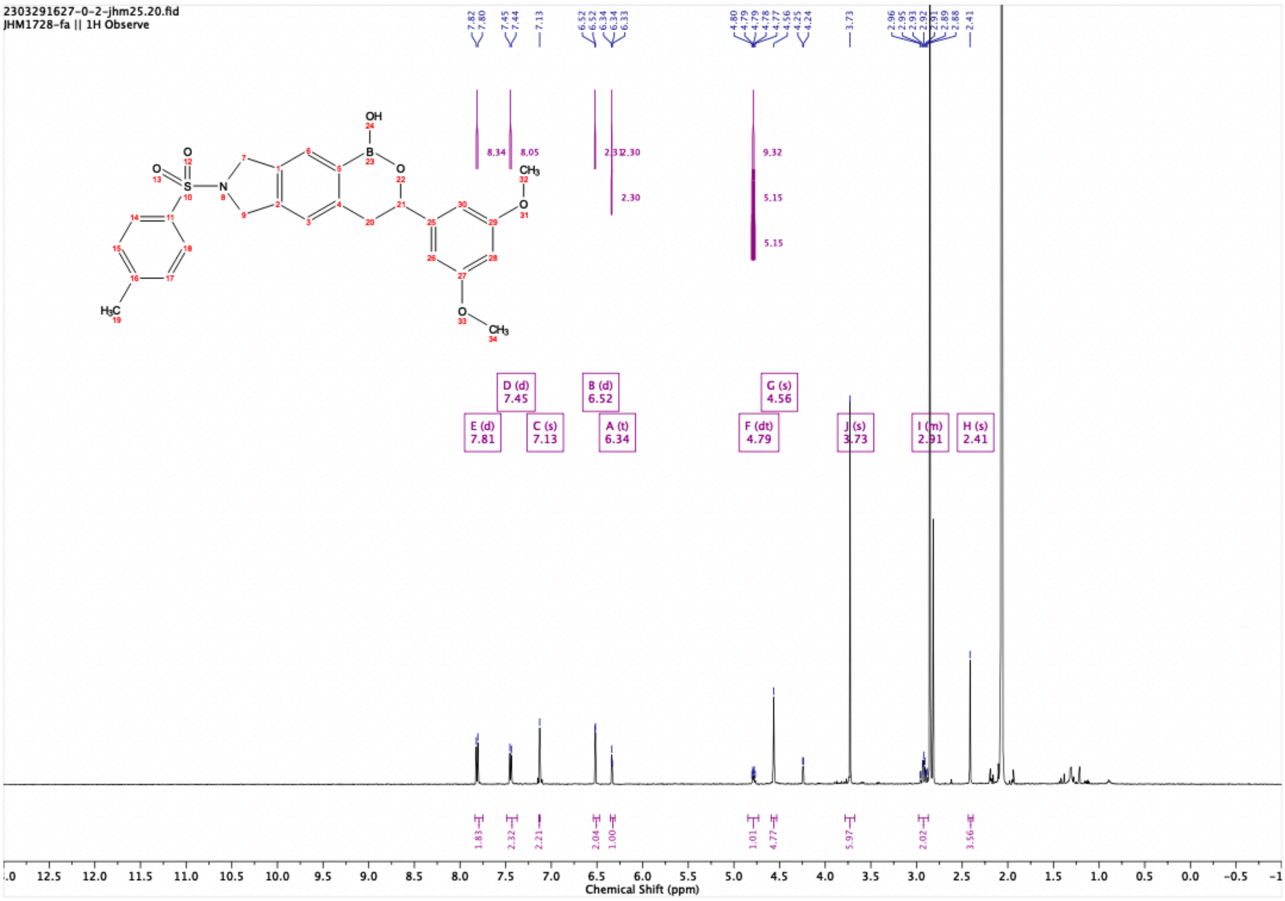

^{13}C DEPTQ

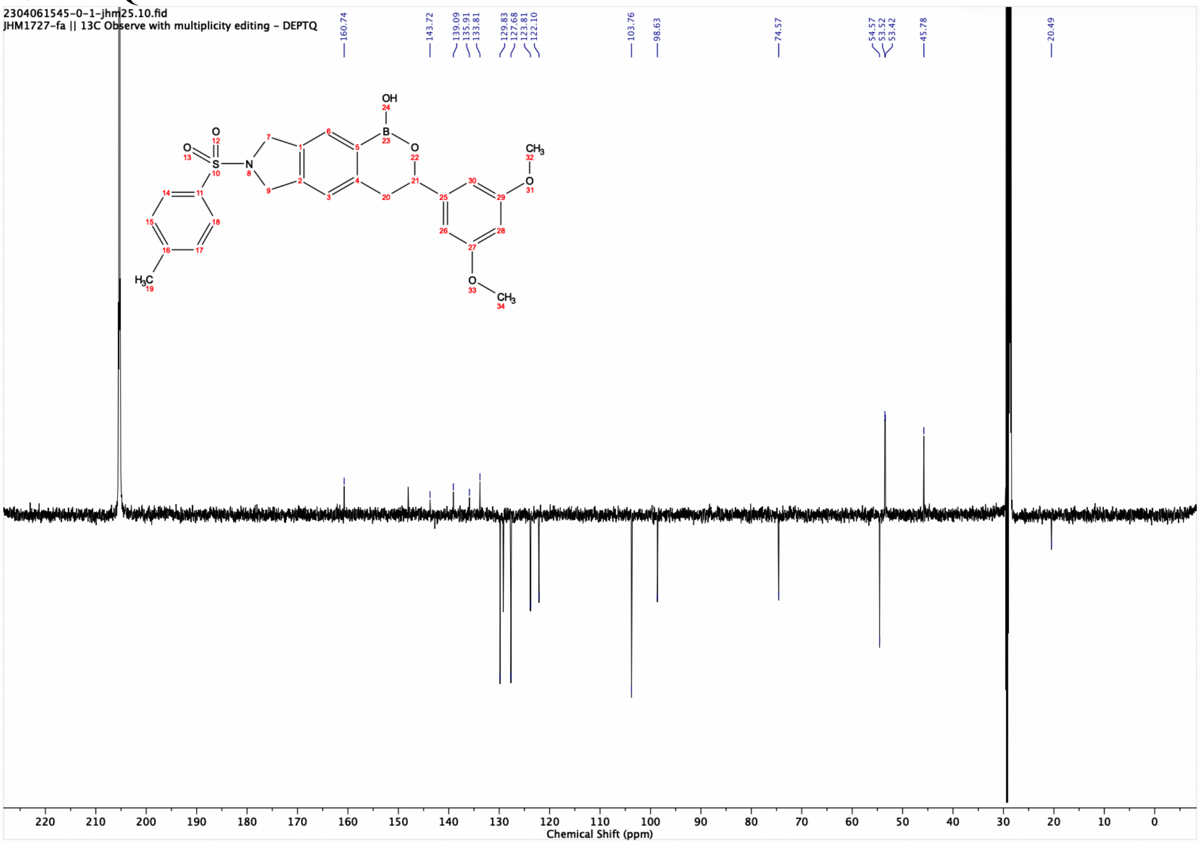

(27)

^{1}H

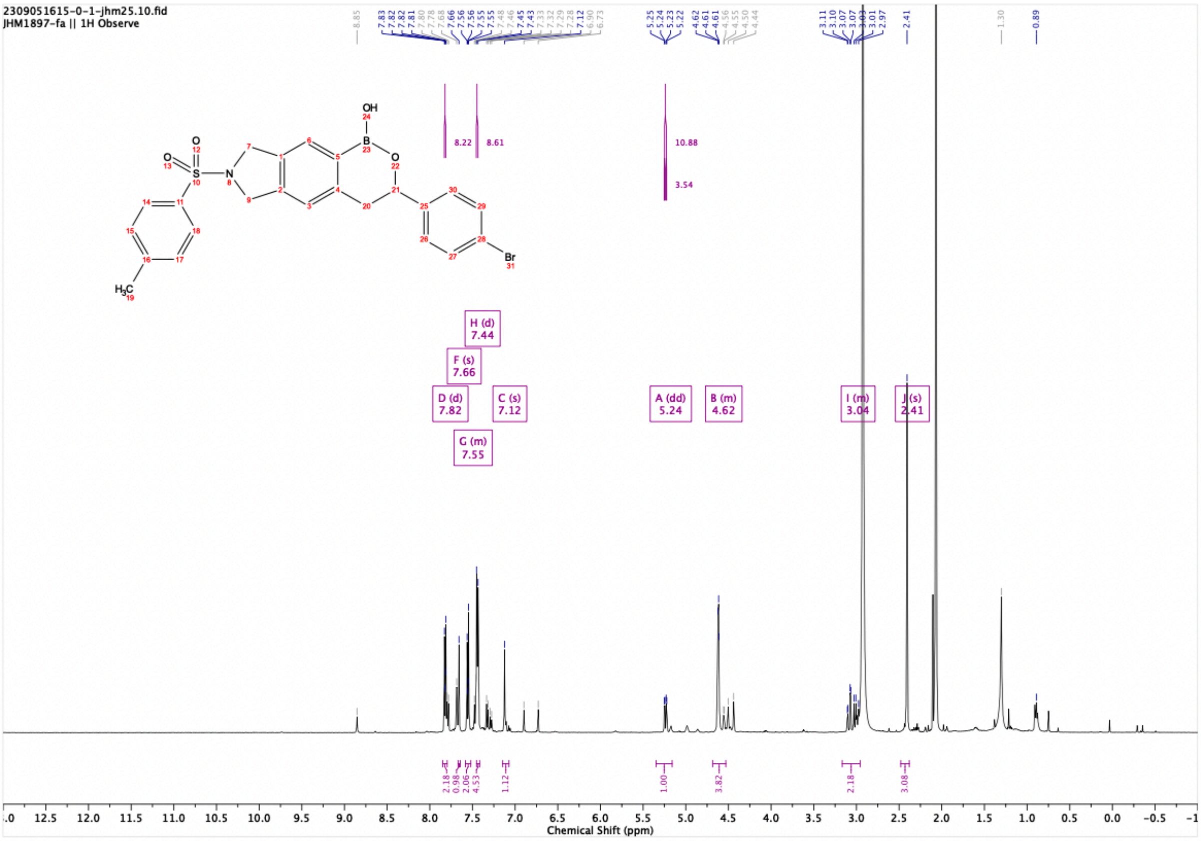

^{13}C DEPTQ

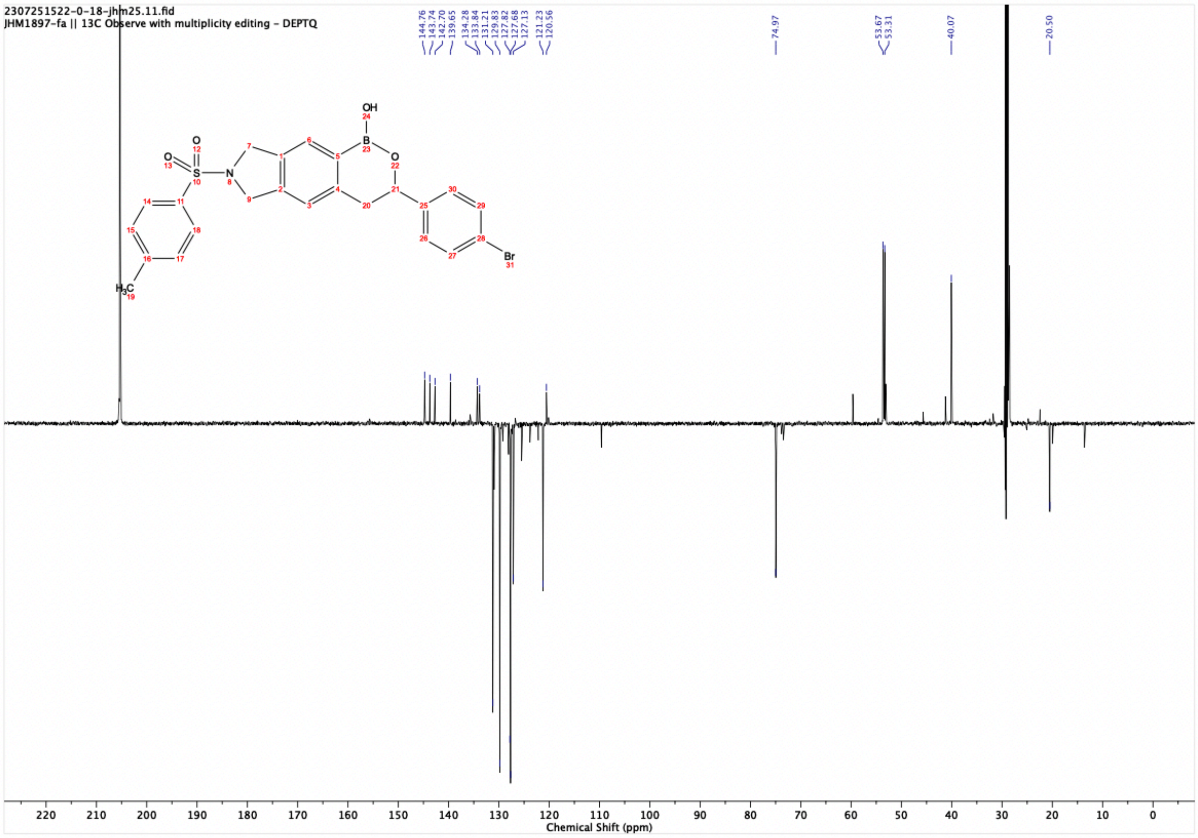

(28)

^{1}H

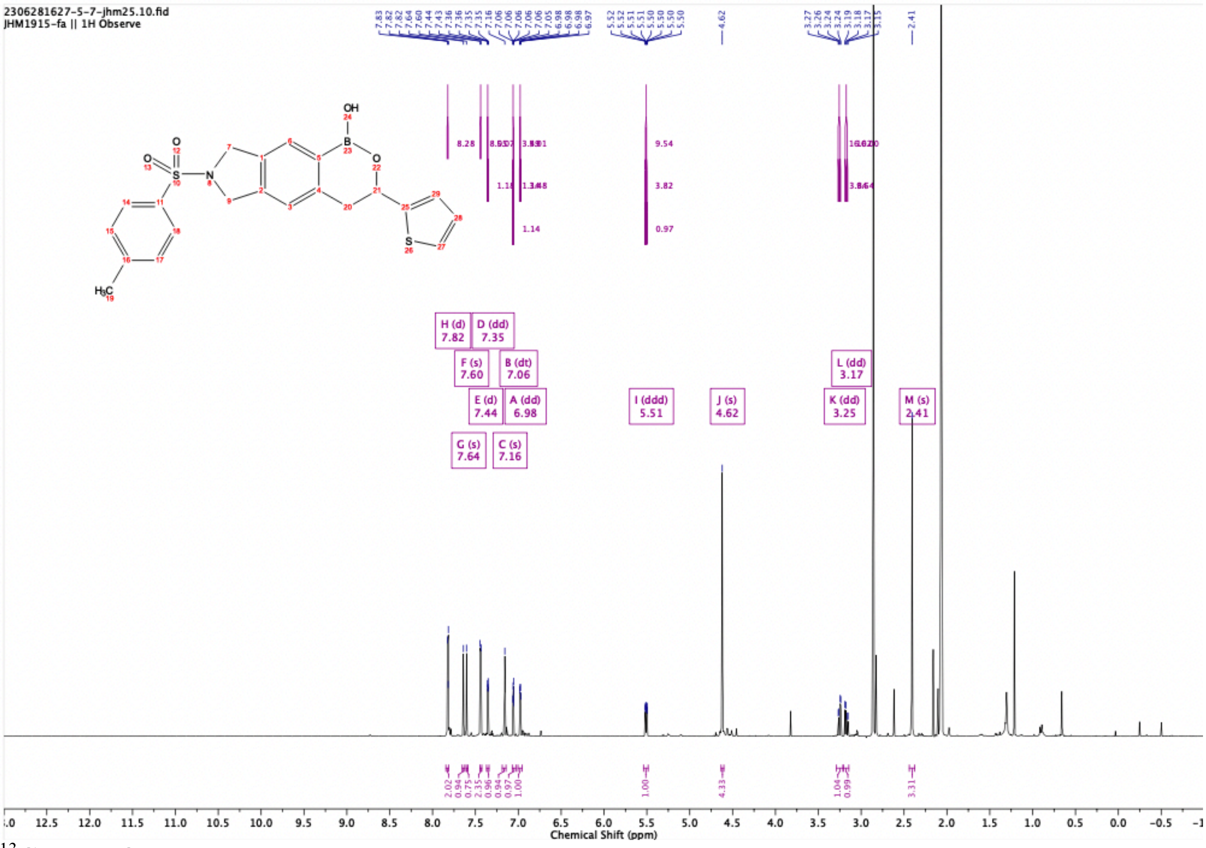

^{13}C DEPTQ

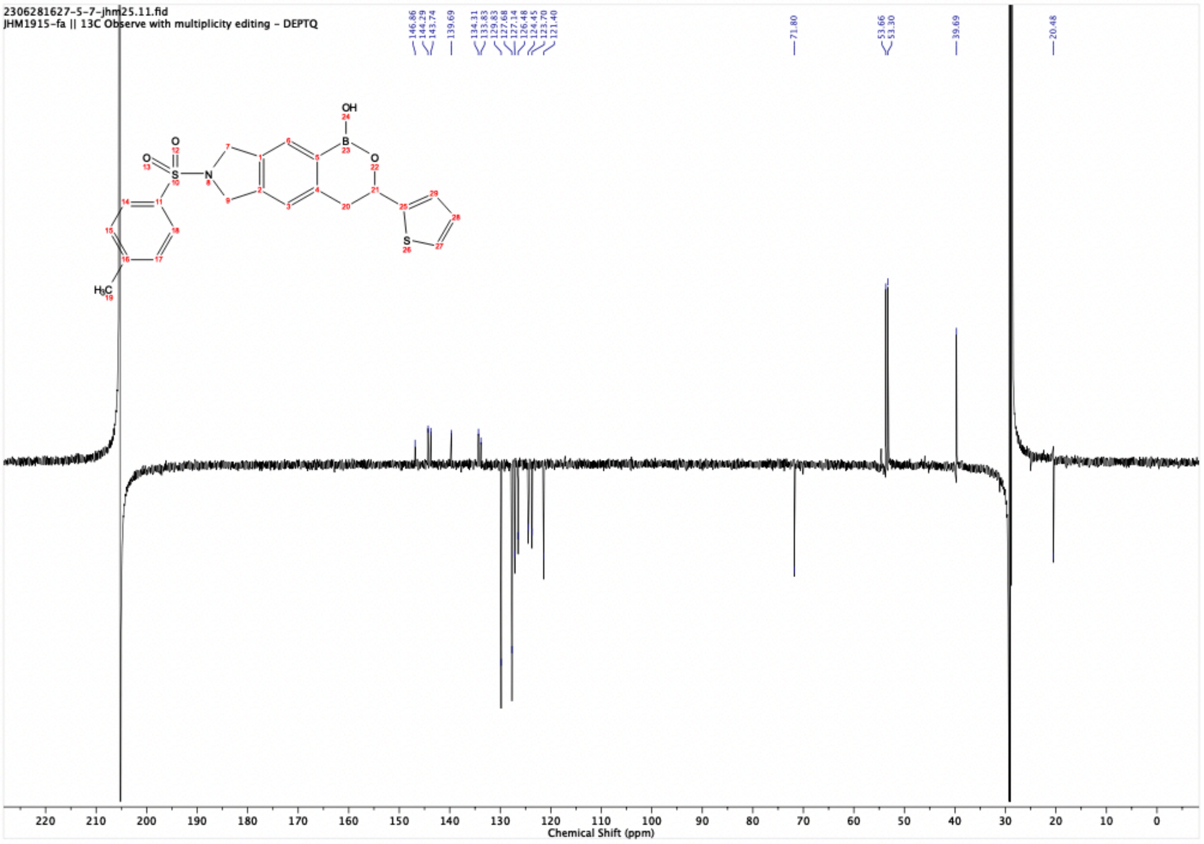

(29)

^{1}H

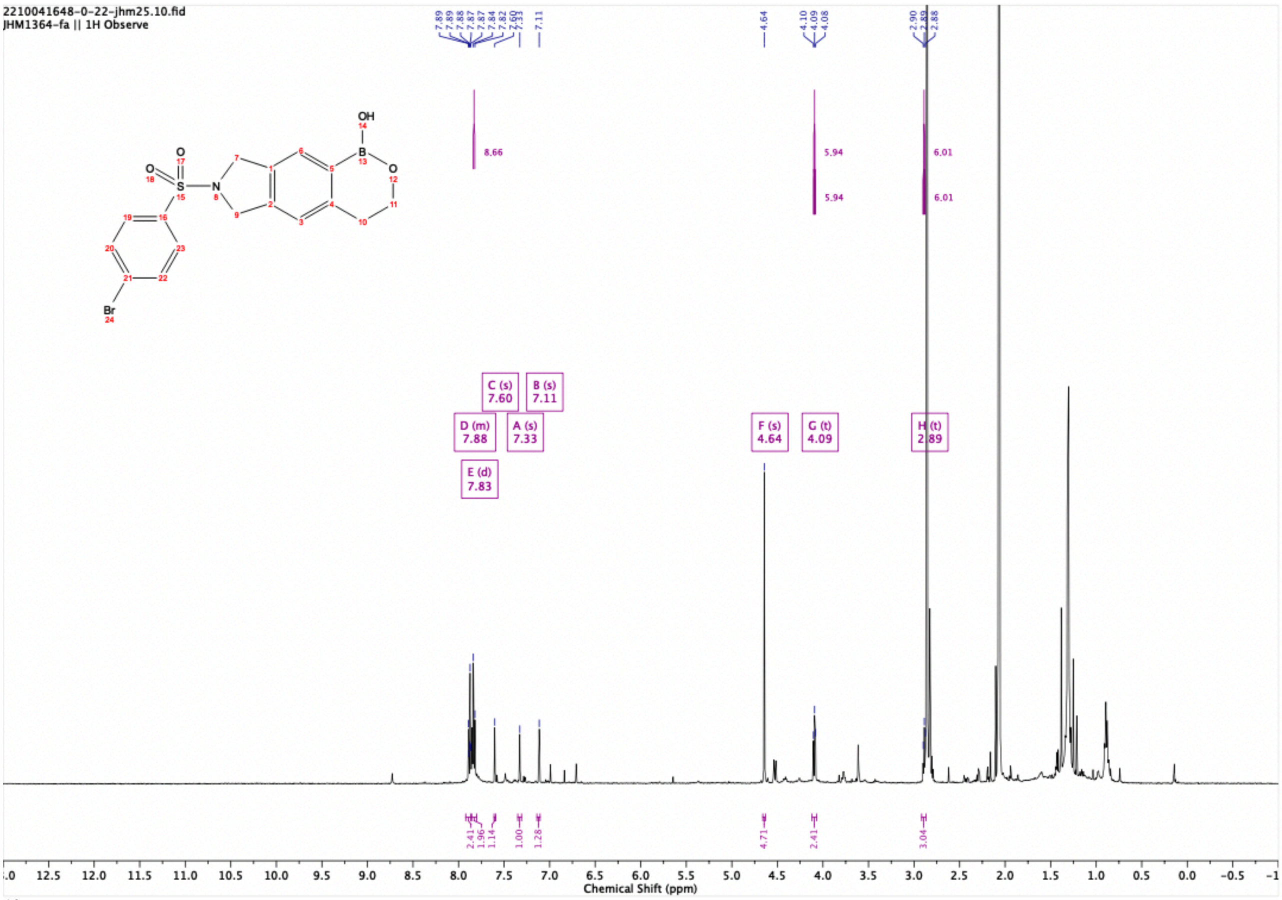
2210041648-0-22-jhm25.10.fid
JHM1364-fa || 1H Observe
Chemical Shift (ppm)

^{13}C

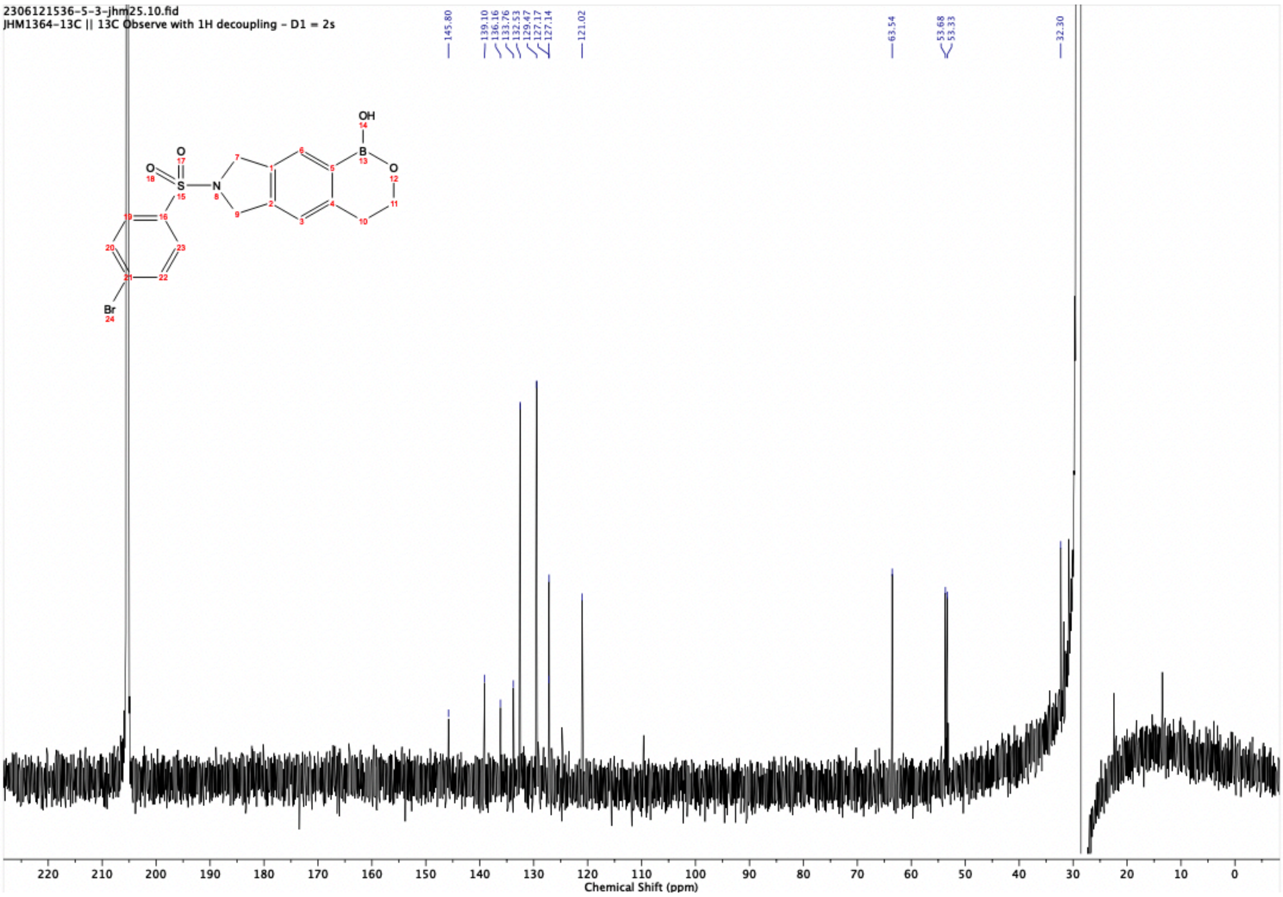
2306121536-5-3-jhm25.10.fid
JHM1364-13C || 13C Observe with 1H decoupling - D1 = 2s
Chemical Shift (ppm)

(38)

^{1}H

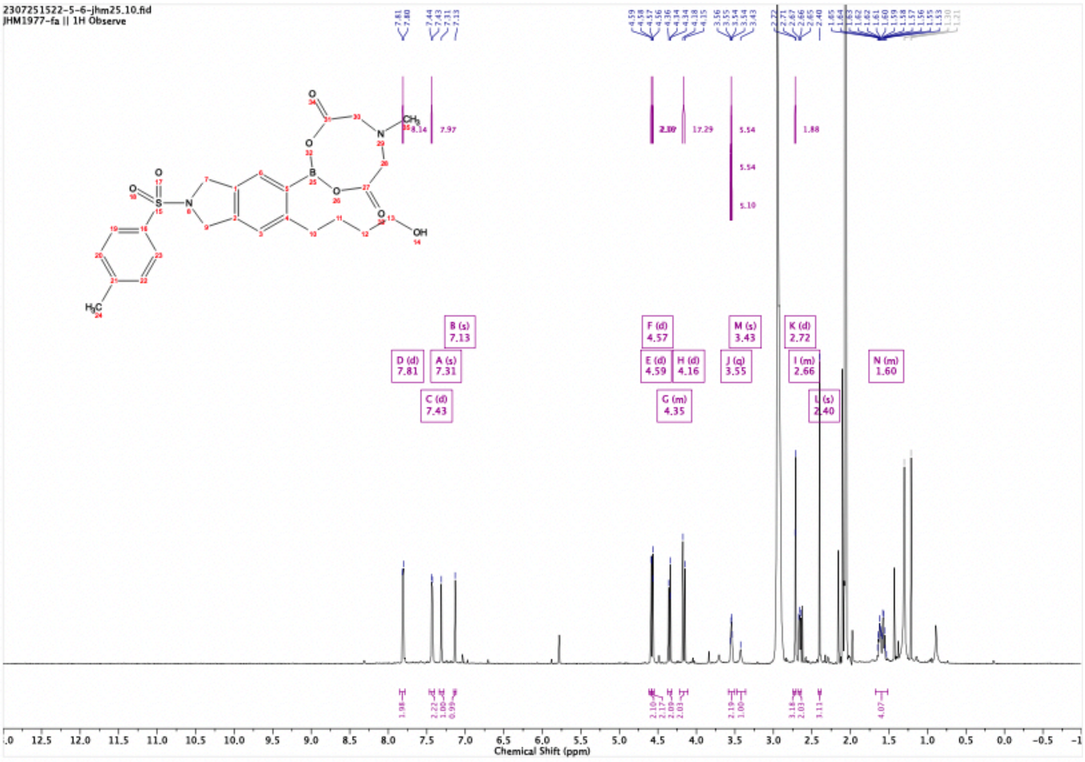

2307251522-5-6-jhm25.10.fid
JHM1977-fa || 1H Observe
D (d) 7.81
A (s) 7.31
B (s) 7.13
C (d) 7.43
F (d) 4.57
E (d) 4.59
H (d) 4.16
G (m) 4.35
M (s) 3.43
J (q) 3.55
K (d) 2.72
I (m) 2.66
L (s) 2.40
N (m) 1.60
Chemical Shift (ppm)

^{13}C

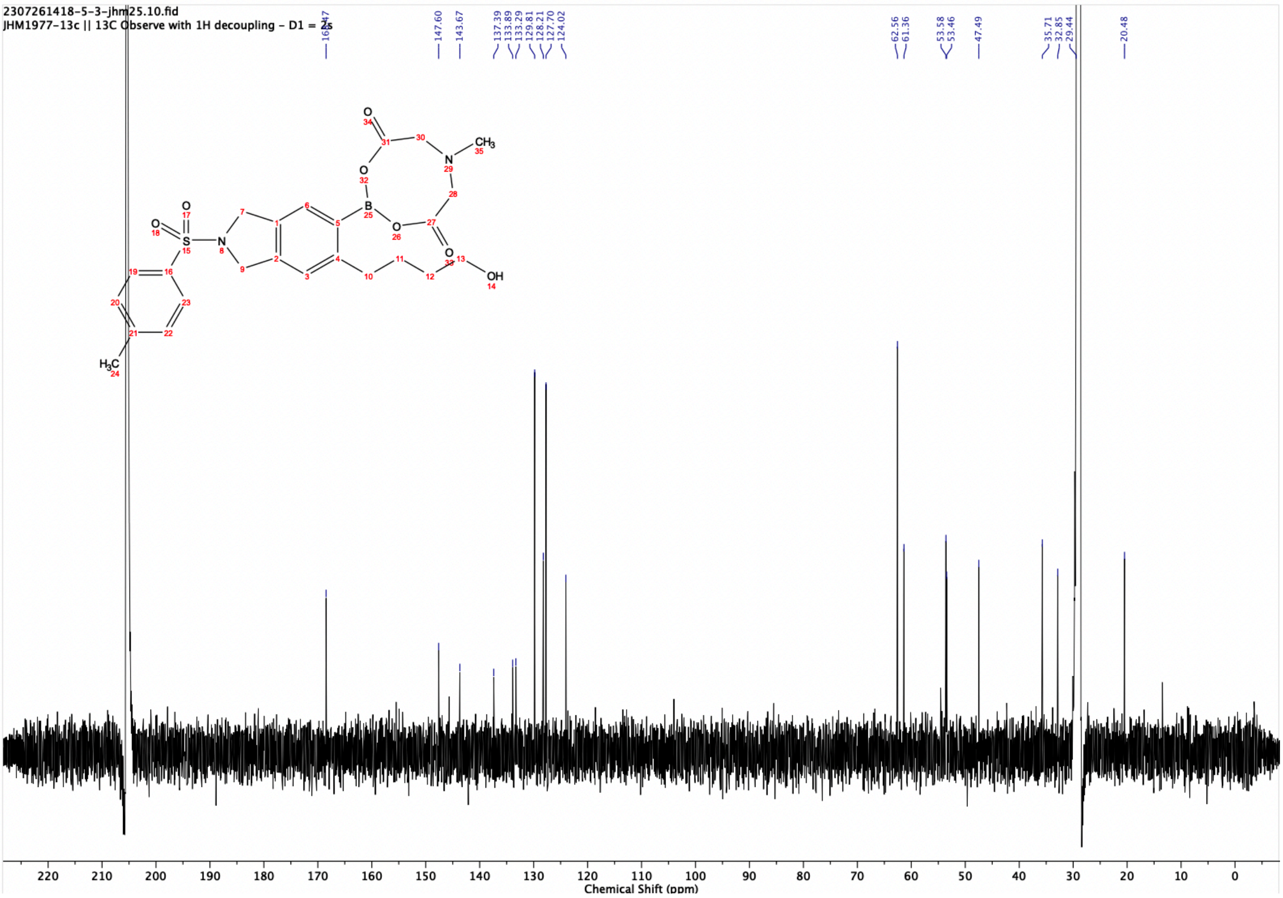

2307261418-5-3-jhm25.10.fid
JHM1977-13c || 13C Observe with 1H decoupling - D1 = 2s
147.60
143.67
137.39
133.89
133.29
129.81
128.21
127.70
124.02
62.56
61.36
53.58
53.46
47.49
35.71
32.85
29.44
20.48
Chemical Shift (ppm)

9. References

1. W. L. F. Armarego and C. Chai, *Purification of Laboratory Chemicals*, Elsevier, Burlington, MA/Oxford, UK, 6 edn., 2009.
2. Miller, E. W.; Albers, A. E.; Pralle, A.; Isacoff, E. Y.; Chang, C. J. Boronate-Based Fluorescent Probes for Imaging Cellular Hydrogen Peroxide. *J. Am. Chem. Soc.* **2005**, *127*, 16652–16659.
3. António, J. P. M.; Carvalho, J. I.; André, A. S.; Sias, J. N. R.; Aguiar, S. I.; Faustino, H.; Lopes, R. M. R. M.; Veiros, L. F.; Bernardes, G. J. L.; da Silva, F. A.; Gois, P. M. P. Diazaborines Are a Versatile Platform to Develop ROS-Responsive Antibody Drug Conjugates. *Angew. Chem. Int. Ed.* **2021**, *60*, 25914–25921.
4. Wang, R.; Bian, Z.; Zhan, D.; Wu, Z.; Yao, Q.; Zhang, G. Boronic acid-based sensors for small-molecule reactive species: A review. *Dyes Pigms.* **2021**, *185*, 108885.
5. Artigas, A.; Vila, J.; Lledó, A.; Solà, M.; Pla-Quintana A.; Roglans, A. A Rh-Catalyzed Cycloisomerization/Diels-Alder Cascade Reaction of 1,5-Bisallenes for the Synthesis of Polycyclic Heterocycles. *Org. Lett.* **2019**, *21*, 6608–6613.
6. Ruhl, K. E.; Rovis, T. Visible Light Gated-Cobalt Catalysis for a Spatially and Tempoally Resolved [2+2+2] Cycloaddition. *J. Am. Chem. Soc.* **2016**, *138*, 15527–15530.
7. Wilking, M.; Daniliuc C. G.; Hennecke, U. Monomeric Cinchona Alkaloid-Based Catalysts for Highly Enantioselective Bromolactonisation of Alkynes. *Chem. Eur. J.* **2016**, *22*, 18601–18607.
8. Ross S. P.; Hoye, T. R. Multiheterocyclic Motifs via Three-Component Reactions of Benzynes, Cyclic Amines, and Protic Nucleophiles. *Org. Lett.* **2018**, *20*, 100–103.
9. Artigas, A.; Castanyer, C.; Lledó, A.; Pla-Quintana, A.; Roglans, A.; Roig N.; Solà, M. Synthesis of Fused Dihydroazepine Derivatives of Fullerenes by a Rh-Catalyzed Cascade Process. *Adv. Synth. Catal.* **2021**, *363*, 3835–3844.
10. Vila, J.; Solà, M.; Pla-Quintana A.; Roglans, A. Highly Selective Synthesis of Seven-Membered Azaspiro Compounds by a Rh(I)-Catalyzed Cycloisomerization/Diels–Alder Cascade of 1,5-Bisallenes. *J. Org. Chem.* **2022**, *87*, 5279–5286.
11. Corr M. J.; O'Hagan, D. Fluorosugars: An improved synthesis of the 2,3,4-trideoxy-2,3,4-trifluoro hexose analogue of D-glucose *J. Fluor. Chem.* **2013**, *155*, 72–77.
12. Sasaki, M.; Hamzik, P. J.; Ikemoto, H.; Bartko, S. G.; Danheiser, R. L. Formal Bimolecular [2 + 2 + 2] Cycloaddition Strategy for the Synthesis of Pyridines: Intramolecular Propargylic Ene Reaction/Aza Diels–Alder Reaction Cascades. *Org. Lett.* **2018**, *20*, 6244–6249.
13. Kramm, F.; Teske, J.; Ullwer, F.; Frey, W.; Plietker, B. Annelated Cyclobutanes by Fe-Catalyzed Cycloisomerization of Enyne Acetates *Angew. Chem. Int. Ed.* **2018**, *57*, 13335–13338.
14. Tan, J.-F. ; Bormann, C. T.; Perrin, F. G.; Chadwick, M. F.; Severin, K.; Cramer, N. Divergent Synthesis of Densely Substituted Arenes and Pyridines via Cyclotrimerization Reactions of Alkynyl Triazenes. *J. Am. Chem. Soc.* **2019**, *141*, 10372–10383.
15. Guo, L.-N. ; Gao, H.; Mayer, P.; Knochel, P. Preparation of Organoaluminum Reagents from Propargylic Bromides and Aluminum Activated by PbCl2 and Their Regio- and Diastereoselective Addition to Carbonyl Derivatives. *Chem. Eur. J.* **2010**, *16*, 9829-9834.
16. Kribber, T.; Labonne A.; Hintermann, L. Iterative Synthesis of Oligo-1,4-diols via Catalytic Anti-Markovnikov Hydration of Terminal Alkynes. *Synthesis* **2007**, *18*, 2809-2818.
17. Li, Y.; Brand J. P.; Waser, J. Gold-Catalyzed Regioselective Synthesis of 2- and 3-Alkynyl Furans. *Angew. Chem. Int. Ed.* **2013**, *52*, 6743-6747.
18. Liang, T.; Woo S. K.; Krische, M. J. *C*-Propargylation Overrides O-Propargylation in Reactions of Propargyl Chloride with Primary Alcohols: Rhodium-Catalyzed Transfer Hydrogenation. *Angew. Chem. Int. Ed.* **2016**, *55*, 9207–9211.
19. Hashmi, A. S. K.; Yang, W.; Rominger, F. Gold(I)-Catalyzed Rearrangement of 3-Silyloxy-1,5-enynes: An Efficient Synthesis of Benzo[b]thiophenes, Dibenzothiophenes, Dibenzofurans, and Indole Derivatives. *Chem. Eur. J.* **2012**, *18*, 6576-6580.
20. Borowiecki P.; Dranka, M. A facile lipase-catalyzed KR approach toward enantiomerically enriched homopropargyl alcohols. *Bioorg. Chem.* **2019**, *93*, 102754.
21. Halford-McGuff, J. M.; Cordes, D. B.; Watson, A. J. B. Synthesis of complex aryl MIDA boronates by Rh-catalyzed [2+2+2] cycloaddition. *Chem. Commun.* **2023**, *59*, 7759-7762.
22. Barrett, R. R. G. ; Campbell, D. A.; Gleason, J. L. An Organocatalytic Oxy-Cope/Michael Cascade Reaction. *Org. Lett.* **2023**, *25*, 777-781.

23. Spencer J. A.; Jamieson, C.; Talbot, E. P. A. One-Pot, Three-Step Synthesis of Cyclopropylboronic Acid Pinacol Esters from Synthetically Tractable Propargylic Silyl Ethers, *Org. Lett.* **2017**, *19*, 3891–3894.
24. Wang, L.; Seiders, J. R.; Floreancig, P. E. Structure−Reactivity Relationships in Oxidative Carbon−Carbon Bond Forming Reactions: A Mild and Efficient Approach to Stereoselective Syntheses of 2,6-Disubstituted Tetrahydropyrones *J. Am. Chem. Soc.* **2004**, *126*, 12596-12603.
25. Peil, S.; Gutherz, A.; Biberger, T.; Fürstner, A. Hydrogenative Cyclopropanation and Hydrogenative Metathesis. *Angew. Chem. Int. Ed.* **2019**, *58*, 8851-8856.
26. Li, X.; He, S.; Song, Q. Diethylzinc-Mediated Radical 1,2-Addition of Alkenes and Alkynes. *Org. Lett.* **2021**, *23*, 2994-2999.
27. Cao, C.; Deitch, J.; Linton, E. C.; Kozlowski, M. C. Asymmetric Synthesis of Allenyl Oxindoles and Spirooxindoles by a Catalytic Enantioselective Saucy–Marbet Claisen Rearrangement. *Angew. Chem. Int. Ed.* **2012**, *51*, 2448–2451.
28. Ojo, O. S.; Hughes D. L.; Richards, C. J. An expedient copper-catalysed asymmetric synthesis of γ-lactones and γ-lactams. Application to the synthesis of lucidulactone A. *Org. Biomol. Chem*. **2023**, *21*, 4144-4149.
29. Bell, G. E.; Fyfe, J. W. B.; Israel, E. M.; Slawin, A. M. Z.; Campbell, M.; Watson, A. J. B. Synthesis of 2-BMIDA Indoles via Heteroannulation: Applications in Drug Scaffold and Natural Product Synthesis. *Org. Lett.* **2022**, *24*, 3024-3027.
30. Bérubé, M.; Dowlut, M.; Hall, D. G. Benzoboroxoles as Efficient Glycopyranoside-Binding Agents in Physiological Conditions: Structure and Selectivity of Complex Formation. *J. Org. Chem.* **2008**, *73*, 6471-6479.
31. Dowlut, M.; Hall, D. G. An Improved Class of Sugar-Binding Boronic Acids, Soluble and Capable of Complexing Glycosides in Neutral Water. *J. Am. Chem. Soc.* **2006**, *128*, 4226-4227.
32. *CrysAlisPro* v1.171.42.53a, 1.171.42.74a, 1.171.42.96a Rigaku Oxford Diffraction, Rigaku Corporation, *Tokyo,* Japan, 2022-2023.
33. Burla, M. C.; Caliandro, R.; Camalli, M.; Carrozzini, B.; Cascarano, G. L.; Giacovazzo, C.; Mallamo, M.; Mazzone, A.; Polidori, G.; Spagna, R. SIR2011: a new package for crystal structure determination and refinement. *J. Appl. Crystallogr.* **2012**, *45*, 357-361.
34. G. M. Sheldrick. SHELXT – Integrated space-group and crystal-structure determination. *Acta Crystallogr., Sect. A: Found Adv.* **2015**, *71*, 3-8.
35. G. M. Sheldrick. Crystal structure refinement with SHELXL. *Acta Crystallogr., Sect. C: Struct. Chem.* **2015**, *71*, 3-8.
36. A. L. Spek. Structure validation in chemical crystallography. *Acta Crystallogr., Sect D: Biol. Crystallogr.* **2009**, *65*, 148-155.
37. Dolomanov, O. V. ; Bourhis, L. J.; Gildea, R. J.; Howard, J. A. K.; Pushmann, H. *OLEX2*: a complete structure solution, refinement and analysis program. *J. Appl. Crystallogr.* **2009**, *42*, 339-341.
